# Comparing Four Question Formats in Five Languages for On-Line Consumer Surveys

**DOI:** 10.3390/mps3030049

**Published:** 2020-07-14

**Authors:** Denis Richard Seninde, Edgar Chambers

**Affiliations:** Center for Sensory Analysis and Consumer Behavior, Kansas State University, Manhattan, KS 66506, USA; seninde@ksu.edu

**Keywords:** Check-All-That-Apply, Check-All-Statements, Rate-All-That-Apply, Rate-All-Statements, CATA, RATA, Eating Motivations Survey, EMS, sensory, marketing

## Abstract

Question formats are critical to the collection of consumer health attitudes, food product characterizations, and perceptions. The information from those surveys provides important insights in the product development process. Four formats based on the same concept have been used for prior studies: Check-All-That-Apply (CATA), Check-All-Statements (CAS), Rate-All-That-Apply (RATA), and Rate-All-Statements (RAS). Data can vary depending on what question format is used in the research, and this can affect the interpretation of the findings and subsequent decisions. This survey protocol compares the four question formats. Using a modified version of the Eating Motivation Survey (EMS) to test consumer eating motivations for five food items, each question format was translated and randomly assigned to respondents (*N* = 200 per country per format) from Brazil (Portuguese), China (Mandarin Chinese), India (Hindi or English), Spain (Spanish), and the USA (English). The results of this survey should provide more understanding of the differences and similarities in distribution of data for the four scale formats. Also, the translations and findings of this survey can guide marketers, sensory scientists, product developers, dieticians, and nutritionists when designing future consumer studies that will use these question formats.

## 1. Introduction

To improve the health of people in our communities, it is important to understand the motivations that drive food choices, the perceptions of foods, such as liking or sensory qualities, and consumer’s reactions to products such as their emotional or attitudinal responses. This information is essential to nutritionists and dietitians as they develop sustainable meal plans for their communities. It also is beneficial to product developers, sensory scientists, and marketing researchers, as it guides them in producing and promoting food products that meet the needs of consumers. Various formats of questions have been used in consumer surveys to collect food product characterizations based on perceptions, opinions, beliefs, and attitudes of target group consumers [1,2,3,4,5,6,7,8]. Some commonly used ones include Check-All-That-Apply (CATA), Check-All-Statements (CAS), Rate-All-That-Apply (RATA), and Rate-All-Statements (RAS). These question formats are commonly used in consumer central location studies [2,7,9,10,11,12], phone interview surveys [13,14], self-administered studies (home-use tests [15,16] and on-line surveys [17,18]), and printed surveys [8] for a number of different types of studies related to consumer perception.

### 1.1. Check-All-That-Apply (CATA)

With CATA, consumers are asked to check all items that are of importance from a list of options [8,19]. The items provided are usually product sensory characteristics [9,20] and physiological and psychographic variables [16]. Despite its prevalence, the CATA question format has faced criticism for uncertainty in the interpretation of the unchecked items. The unchecked items could be explained in three different ways: Either (a) the items do not apply, (b) there is indecision of respondents, or (c) items were intentionally or non-intentionally left unchecked.

As the CATA question asks the respondent to check all items that apply, it would then be expected that the unmarked items are not important or do not apply. However, it is also likely that respondents who are undecided on whether a particular item applies could opt not to check the item. This lack of a neutral option in the CATA question format could impact on the accuracy and reliability of the collected responses that are intended to guide decision making when developing products. Also, whether intentionally or unintentionally, some items may remain unchecked by the respondent. It is possible that some unchecked items were not seen by the respondent as they speedily answered the question [5,18] or it could be that the respondent intentionally did not bother reading the entire list of options to save on time [21]. Such outcomes are brought on by the non-compulsory nature of the conventional CATA question format that does not require a response from each of the listed items. It is no wonder that the CATA question format has been described by respondents as fast and non-tedious as evidenced by the significantly shorter survey or study mean durations and higher respondent liking as compared to other diagnostic attribute rating questions such as the Just-About-Right (JAR) rating questions [1,10,22]. The JAR rating questions are popularly used in the product development process to optimize product sensory characteristics. Consumers are asked to rate the strength of an attribute based a 3 point or 5 point bi-polar scale with JAR at the center point, too weak on one end and too strong on the other end [10,23].

This, however, highlights the fact that a typical CATA question demands less cognitive effort from the respondents as compared to other question variations, such as the CAS, and other diagnostic rating questions such as Just-About-Right (JAR) [7,10,22]. Consequently, the amount of detail that is collected by the conventional CATA question format could be substantially less than that collected by similar question formats that require more cognitive effort from the respondents for each listed item. Hence, the level of thought that respondents accord to questions could have an impact on the accuracy of the information collected by the question format and this warrants more investigation [24].

Even more, a tendency for respondents to mark items that appear at the top of the list more than items that appear in the middle or at the bottom of the list (primacy bias) has been associated with print and online surveys [18] and central location studies [4] that employed the CATA question format. For phone interview surveys, where a list of CATA items are read out to the respondent, there is a likelihood of the items that were read last to be selected more as compared to items that were read at the beginning of the list, because they are more memorable (recency bias) [25]. For instance, telephone surveys that have long lists of CATA items or complicated CATA items could increase the cognitive burden, as respondents need to remember both the question and CATA items to form an accurate response [26].

### 1.2. Check-All-Statements (CAS)

In phone interview surveys, a different question format that is known as the Check-All-Statements (CAS) has been used extensively [14]. This question format has been applied also in on-line [18] and print surveys [8]. With CAS, respondents are presented the same CATA items but this time a “Yes” or “No” response is required for each item. For phone surveys with long lists of CATA items, the CAS question format would appear to be more feasible as respondents are not troubled with remembering all the items when making a selection of which apply but rather would provide a “yes” or “no” to each item as the interviewer reads them out. The CAS question format has been shown to result in more detailed responses in terms of a mean number of affirmative or positive checked responses per respondent as compared to the CATA format [2,8,18,27].

Thomas and Klein [27] showed that more detailed responses were consistent in various behavioral studies conducted in different languages and countries of residence. According to Sudman [19], Smyth et al. [18] and Nicolaas et al. [14], respondents apply more cognitive effort when answering CAS as compared to the CATA format. Nicolaas et al. [14] reported that respondents took longer to complete CAS questions when they were offered across in-person interviews and on-line surveys as compared to corresponding CATA questions. Smyth et al. [13] found that the data collected by CAS in phone interview surveys and online surveys were similar. That confirmed Smyth et al.’s [18] earlier claim that CAS questions collected more detail as compared to CATA. Also, while responses from CATA questions could be susceptible to primacy bias, responses from CAS are not affected by this effect [13,19,25].

It is worth noting that the CAS question format can be limited by the tendency of respondents to select affirmative or positive responses more frequently (acquiescence bias) [14]. However, the findings of Nicolaas et al. [14] and Smyth et al. [18] had no such effects, but they had reasonably short questionnaires of 8–15 questions. Nicolaas et al. [14] also showed that that the lack of a neutral option in CAS, when applied to online surveys, could prompt respondents to select “yes” as the next alternative option when faced with indecision. This could influence the accuracy and reliability of the findings collected. According to Best and Krueger [28], requiring an answer for each of the items could upset respondents and could lead to a high number of partial completes or “drop-offs” as respondents quit the survey before it is completed. Typically, studies in the sensory and marketing literature have longer questionnaires than those in the survey studies that have been conducted, which could impact findings.

### 1.3. Rate-All-That-Apply (RATA)

In some cases, knowing that an item is important or applies is not enough and researchers want to gain more understanding of the level of importance or “how much” the item applies to the study question. To do this, researchers sometimes use the Rate-All-That Apply (RATA) question format where, if the item is checked as applying, respondents are then asked to rate how much the selected items apply based on a given scale. Usually, a Likert type scale (3, 5, 7, or 9 point) can be used for example; a 5 pt scale anchored at “Not at All Important” and “Extremely Important” can be used to rate the applicability or importance of each selected CATA item. More discrimination in product liking was realized by Jaeger et al. [22,29] in a total of five and eight consumer sensory studies when RATA was compared with the CATA question format.

However, Vidal et al. [30] refuted this claim and showed that there was no significant difference between RATA and CATA responses but noted that the use of either format was depended on the objective of the study and characteristics of the product category being investigated. According to Jaeger et al. [22], CATA and RATA were employed in emoji questionnaires that investigated the emotions consumers experienced when they consumed food products. Researchers found that while CATA and RATA questions produced similar proportions of emojis in central location tests, RATA questions produced a significantly greater proportion of emojis as compared to CATA in the online surveys. This study suggested that the reliability of data for consumer testing of foods that is collected using either CATA, CAS, or RATA questions could depend on whether the survey or study was conducted at a central location or via on-line testing.

### 1.4. Rate-All-Statements (RAS)

Another CATA format is the Rate-All-Statements (RAS) where instead of having the respondents check all items that apply and subsequently rate how much the selected items apply; they are directly asked to rate all CATA items. A similar Likert-type scale for RATA is usually used. This question format may collect more detail as compared to either CATA or CAS or RATA. The RAS question format would be expected to require a higher level of thought process and likely would result in longer mean survey duration as compared to the other aforementioned three question formats. Little literature was found on RAS as applied to consumer product characterizations in sensory analysis surveys and study questionnaires. Thus, there is a research gap for exploration into the results and distribution of responses collected by these four question formats (i.e., CAS, CATA, RAS, and RATA) across different survey questionnaire fielding platforms (i.e., online, print via mail, central location testing and via telephone). Also, there is little research on the impact of demographic aspects such as age and gender and location on CATA data.

The overall objective of this survey was to compare the CATA, CAS, RATA, and RAS question formats. The specific questionnaire used for the comparison was an on-line eating motivations survey. Specific objectives for the questionnaire comparisons were to (a) compare the number (percentage) of items identified as positively motivating the eating of specific foods by either of the four formats; (b) the length of time taken to complete questionnaires in the four formats; (c) compare liking and just about right questions for the four formats of questionnaires; and (d) compare completion rates for the four formats. Specific objectives for the eating motivations survey were to use results from the surveys to determine specific eating motivations that can guide marketing, sensory, product development, and nutrition intervention for each country. This writing provides a step-by-step description of how and what materials, methods, and protocols were used during the preparation and fielding stages of the on-line survey in five languages.

## 2. Methods and Design

### 2.1. Questionnaire Development

The Eating Motivations Survey (EMS) was developed to compare four question formats (i.e., CATA, CAS, RATA, RAS) across five food groups in five countries. The questions investigated the respondents’ motivations for eating or not eating varying food items that belong to five different food groups (Starches, proteins, dairy, fruits, and desserts foods or desserts) [17,31] (Table 1). Differences in product availability and potential preferences among respondents in different countries were considered and food items commonly consumed in respective countries were used for the various question formats. For example, in the USA, respondents were asked about their motivations for eating baked potatoes (starches), hamburgers (proteins), cheese (milk and dairy), bananas (fruit and vegetables), and chocolate cake with frosting (desserts), all commonly eaten foods in the US. In Brazil, the baked potato was replaced with white rice as a starchy food, Feijao (a type of bean stew) replaced hamburger for protein foods, milk replaced cheese in the diary category, and brigadeiro (fudge cake balls) replaced chocolate cake with frosting. Bananas were commonly consumed by respondents from all five countries.

Based on previous research, a total of 16 motivation constructs, 15 from Renner et al. [32] and one added by Phan and Chambers [17], were used as motivation items for eating the different foods. The survey included motivation constructs such as Liking, Habits, Need and hunger, Health, Convenience, Pleasure, Traditional eating, Natural concerns, Sociability, Price, Visual Appeal, Weight Control, Affect Regulation, Social norms, Social Image, and Choice. Except for the Choice construct, which had two positive sub-items, the rest of the motivation constructs each consisted of three positive sub-items (Table 2). For example, for the Liking construct, respondents could have been motivated to eat a certain food either because they liked it and or because it tasted good and or because they had an appetite for it. As for the Choice construct, responses were collected using two sub-items that is either the respondent wanted to eat the food every day and or because the food was the only choice. On the other hand, in cases where respondents did not eat a particular food item, a different list of negative CATA items (sub-items) was presented (Table 3). Except for the Sociability construct all 16 motivation constructs had at least one negative sub item option presented to the respondents. For example, for the Liking construct, respondents either may not like the food item or the food item may not have been something they had desire to eat at the time. Overall, the number of positive CATA items were 47 while the negative CATA items were 20.

Additionally, the survey questionnaire included the food involvement scale [33] (13 questions), health and taste and attitudes scale [34] (14 questions), and neophobia scale [35] (10 questions) and the demographic questions [36] (5 questions). Furthermore, at the end of EMS, respondents were asked to rate how much they disliked or liked taking the survey (one question) and also to rate how long or short they found the survey (one question). This survey was designed following an approved protocol for conducting research that involves human subjects (IRB #7297.2) that were approved by the designated committee at Kansas State University, Manhattan.

### 2.2. Questionnaire Translation

The use of consumer surveys is widespread and testing question formats in only one culture or language does not answer whether findings apply solely for that country/culture or are more generalizable. Thus, the consumer eating motivations survey using the four question formats were translated and tested in five countries: Brazil, China, India, Spain, and the USA. The questionnaire was initially written in English (Figure S1) for the respondents in the USA and was translated into Portuguese (Figure S2), Simplified Mandarin (Figure S3), Spanish (Figure S4), and Hindi (Figure S5) for respondents in Brazil, China, Spain, and India, respectively. The survey translation process used a variation of the translation, review, adjudication, retesting, and documentation (TRAPD) approach [37,38]. First, the surveys were written in English and pre-tested to determine ease of use and to ensure that the language matched from questionnaire to questionnaire for each questionnaire (the format of the question changed, but not the question itself). Then, the questionnaires were translated by an expert in the subject area who is a native speaker of the language who also spoke/read English and then back translated by another subject area expert native speaker who made alternations, if needed. Various authors have pointed out that differences between the original questionnaire and the back translated questionnaire can be ascribed to errors in the forward translation and can be emanating from the back translations and other errors [39,40]. In the modified TRAPD process both translators worked together (either face to face or online) to check the final translation and ensure the meanings were as intended. If there was disagreement the plan was to bring in a third party to adjudicate, but in this study, the two translators were able to reach agreement in every case. This procedure has been used for other surveys across multiple languages [36,41,42,43]. After this “adjudication” step, a “soft launch” in each country with 50 consumers was conducted to test each translated questionnaire [37] to determine if the questionnaires could be successfully understood and completed in the allotted time by the contacted on-line subjects. Data from the soft launch were tracked, no missing data were found, all data were found to be reasonable, and data from screening and validity checks questions included to determine if consumers were paying attention were similar across countries. Information from all steps in the process was documented.

### 2.3. Respondents and Recruitment

Respondents in each of the five countries (i.e., USA, China, Brazil, Spain, and India) were recruited by Qualtrics, Provo, UT, USA using its or its partners existing databases. Qualtrics or its partners maintain proprietary databases of consumers in each country (usually with more than 1 million respondents per country and many more in some countries such as the USA). The databases have a range of demographics, which can be parsed based on age, sex, purchasing habits, etc. Each survey question format (CAS, CATA, RAS, and RATA) was assigned randomly to ~200 respondents per country meaning ~800 respondents per country were used in the test. Each of the 200 respondents per format were divided into 4 age groups with ~50 respondents per group for the study: (a) Generation Z (born in the years 1995 to 2001), (b) Millennials (born in the years 1980 to 1994), (c) Generation X (born in the years 1965 to 1979), and (d) Baby boomers (born in the years 1944 to 1964). Within each age, 50% were female and 50% were male. For recruiting, Qualtrics sends an e-mail to a percentage (e.g., 200% of the target sample size) of random members of its panel that a survey is available. Those members who volunteer to take the survey complete a screening questionnaire to determine if they qualify (for this survey they had to fit within a particular age, gender quota). If they qualify, they take the survey. If they complete the survey, they receive compensation usually based on a points system for the country they live in. If they fail to complete the survey within a specific period of time, complete the survey too fast, or answer questions incorrectly that are intended to check if the respondent is paying attention, the respondent is exited from the survey. It must be noted that although the databases are populated with consumers from a broad range of consumers in each country, only those with access to the internet are included. Although China, India, the USA, and Brazil had the highest number of internet users in 2015 and Spain had one of the highest percentages of users [44] some individuals are not accessible using this method and, therefore, are excluded from this type of survey.

## 3. Procedure

### 3.1. Data Collection

#### 3.1.1. Panel Screening Process

**Figure 1:**
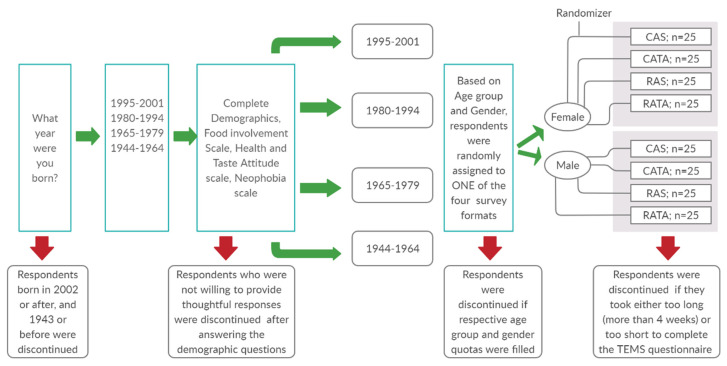
Process flowchart of the recruitment process for respondents who completed the entire survey questionnaire. Green arrows represent respondents who were selected to continue to complete the survey while the Red arrows represent respondents who were discontinued from participating in the survey. A total of 200 respondents per country per question format were recruited.

**Figure 2:**
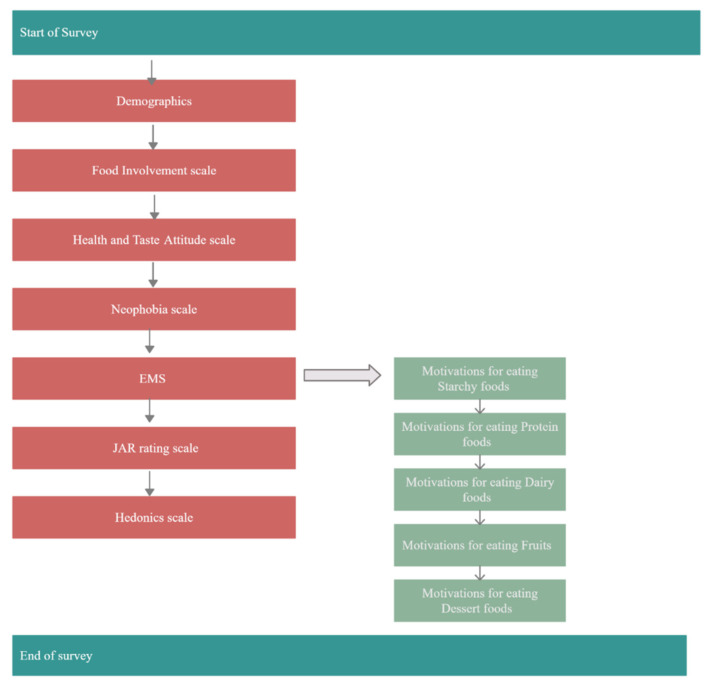
Flowchart showing the flow of questions of the entire survey questionnaire. EMS = Eating Motivation Survey; JAR = Just-About-Right.

This survey fielded during the summer of 2019. Respondents were required to be 18 years or older (born in 2001 or before) but not older than 75 years (born in 1944 or after). Respondents that did not meet the required age criteria were discontinued from completing soon after starting the survey. Another trigger was positioned after the completion of the demographic questions but before the start of the food involvement questions (Figure 1). Respondents who were not willing to provide thoughtful responses were discontinued. Also, if after completion of the demographic questions, and consequent questions on food involvement, heath and taste attitude and neophobia (Figure 2), a respondent was randomly assigned to a quota that had been filled, they too were discontinued from the survey. Furthermore, after the close of the soft launch, a speed check (half of the median time taken by respondents to complete the survey during the soft launch) was added. This inclusion allowed for the automatic termination of responses from people who went so fast through the questionnaire that they likely did not provide thoughtful responses but instead hurriedly completed the survey.

#### 3.1.2. Survey Testing Design

Respondents first completed questions on demographics, food involvement, health, and taste attitudes, and food neophobia. Then they were asked to complete the EMS using one of the four question format variations. The computerized randomizer tool that was used took into consideration the gender (female, male) and age group (four age groups) of the respondents in order to ensure approximately equal numbers of respondents in each gender and age group for each question format. Initially, a pilot test with about 100+ respondents was conducted to verify that the survey questionnaire was collecting data as designed. The initial responses were carefully examined to check for any missing data and identify any corrections that needed to be made before final data were collected. This is the last point at which researchers can revise questions and flow to ensure the appropriate data were being collected. In this study, for example, researchers noticed that the randomizer assigned all four question format variations to each respondent. Thus, those data were discarded, and the randomizer tool was reprogrammed to randomly present only one question format to each respondent. Incomplete or partial responses for cases where the respondent did not complete answering the entire questionnaire within 4 weeks were recorded but not included in the respective quota fulfillment. That allows comparison of survey completion rates among the question formats to provide more understanding of how each format influences the willingness of respondents to complete the survey. Such information is critical in guiding researchers when designing self-administered surveys on consumer behavior. The time that was taken by each respondent to complete the entire survey was recorded in seconds.

### 3.2. Survey Timeline

There was no specific length of time that was anticipated for the respondent recruitment and completion of fielding phases of the survey. This can be explained by the fact that both the recruitment and survey questionnaire completion occurred simultaneously. It can further be attributed to the complexity and number of required quotas (2 genders*4 age groups*4 questionnaires; =32) of this survey. However, from the start of respondent recruitment to the fulfillment of all quotas, it took on average five weeks to complete fielding in each country. Survey fielding began in the USA and continued to other countries as the respective translations became available. As expected for each country, certain quotas filled up quickly as compared to others. For instance, for China, the generation X, and the boomers quotas took a longer time to fill up as compared to the quotas of the younger people.

### 3.3. Data Analysis

Chi-square will be used to compare the CAS, CATA, RAS, and RATA data for each food group in each of the five countries. The ANOVA will be used to assess the effect of survey format on survey liking, mean duration, and respondent JAR for EMS in each country. Percentages of completion rates for each of the four question formats will be calculated. All analyses will be run using XLSTAT (a Microsoft Excel data analysis add-on tool).

## 4. Expected Results

The results of this survey protocol can help to make decisions related to the best choices for determining survey question formats for future studies based on whole sample, age group and gender subsamples. The number (percentage) of items identified as positively motivating the eating of specific foods by the various formats will be compared in order to determine which format provides the most in-depth information. In addition, the completion rate and length of time respondents take to complete questionnaires in the four formats can be compared to determine if one or another format is reducing respondent participation or taking excessively long to conduct the survey. For on-line surveys, time is money; longer surveys cost more to conduct because respondent incentives must be higher. If one format takes much longer to complete than another format, the cost could be too high unless the data provides significantly more robust information. Respondent acceptance of the survey and their beliefs in it being too short, just right, or too long also can be assessed. In addition, eating motivations for various food groups in different countries with both males and females and different age groups can be determined. The questionnaires are available and translated to be used directly or with additional modification by other researchers. The research results using this protocol can be used to guide nutrition and health interventions and assist marketing, sensory, and product development professionals in each country.

## 5. Discussion

### 5.1. Survey Timeline

#### 5.1.1. Development of Survey Questionnaire (s)—Time for Completion: 3 Weeks in this Study

Based on the objective(s) and purpose of the survey, items or terms to be included in questions, target population, and other items must be identified and established as part of the survey. Buy-in from stakeholders who will use the survey results must be obtained in order to make the data useful to them. In this project we discussed the project with a wide range of stakeholders before committing to the final design. Depending on the complexity of the project this timeline could be from a few days to more than a month.

Determining the questions should be done by review of available literature (e.g., the 15 motivation constructs from Renner et al. [32] and one construct from Phan and Chambers [17]). Also, qualitative approaches such as focus groups or one-on-one interviews with consumers and subject matter experts can be used to identify the appropriate terms [45] that will be used in the questions. Additional questionnaires were added to this survey to obtain information on food involvement [33], health and taste attitudes [34], and food neophobia [35]. Because this survey was to compare survey question formats (not known to consumers), respondents also were asked to rate the survey questionnaire based on how long or short it was (7 point JAR scale). Respondents also used a five-point hedonic scale to rate their experience of taking the survey (survey acceptance). Those two questions provide more understanding of respondents’ questionnaire acceptance and perceptions on the duration of the survey.

The target population for the survey must be determined based on demographic, psychographic, or behavioral criteria of interest. Demographic questions such as gender, age group of respondents, education level, number of adults, and children who live in the respondents’ home can be added to the questionnaire [36]. For international research, considerations should be made for several factors such as the culture and traditions of the people, official language, government restrictions, and policies on research involving human subjects [46].

For this survey, we also included a survey respondent “quality” or “trap” question to catch respondents who do not providing thoughtful responses. Questions with an obvious incorrect response or that require the consumer to do something that they may miss if they are not actually paying attention to the questions can help to minimize poor quality data [47].

#### 5.1.2. Test Design, Questionnaire, and Survey Flow Verification—Time for Completion: 1 Week in this Study

Based on previous experience in conducting on-line surveys and because all the different versions of the questionnaire, including all language versions, should be tested to ensure that they run smoothly as required using the on-line survey tool (e.g., Qualtrics survey software) a pre-test in actual field trials with 10% of the sample was conducted. The survey flowed from demographic questions to food involvement scale to health and taste attitude scale to neophobia scale and then to EMS questions (Figure 2). For EMS, respondents answer CATA questions on starches, proteins, dairy, fruits, and desserts foods in that order [17,31]. After completion of EMS, respondents answered the JAR and hedonic questions.

#### 5.1.3. Respondents Recruitment and Survey Fielding—Time for Completion: 4–6 Weeks Depending on the Availability of Target Population

For this study, recruitment and survey fielding was conducted simultaneously. Potential respondents in existing Qualtrics panel databases were screened, but that screening happens at different paces in various countries and cultures. In some countries where consumers of all ages regularly check computer communication (e-mail, text, etc.) screening and testing happened quickly. In other countries or for some demographic groups, recruitment and fielding took longer. In this case, we also checked the data after “completion” of the survey and added additional respondents when needed. The initial fielding took approximately 4 weeks with checks and additional recruitment taking 2 more weeks.

#### 5.1.4. Data Analysis and Reporting—Time for Completion: On-Going

Responses were recorded in real-time as the respondents completed the survey questionnaire. The responses were coded and downloaded as Microsoft Excel datasets. Although simple analyses of percentage responses for items, such as positive or negative motivations and mean values of time and questionnaire acceptance, can be analyzed quickly, more in-depth analysis by gender, age group, and other survey data clusters take much longer to analyze and understand. Timing also is affected by other work streams the researchers are working on.

## 6. Limitations

On-line surveys only test those consumers who are on-line and accessible, an increasingly large part of the population but still only a portion of the global population. In some parts of the world such testing is impossible, and those sections are missed in on-line testing regardless of the question format used.

For the large population that can use on-line testing, the complexity of surveys such as this one that have a large number of recruitment categories, numerous question items (47 positive and 20 negative), additional questionnaires used (e.g., food involvement, taste and health attitudes) and various survey flows for question formats, specialized computer programs are required to set up the surveys and ensure they are properly fielded. For example, for the RATA format, the Qualtrics system provided a pop-up question when respondents rated an item as important. Other survey computerized systems or survey methods (for example, in person paper ballots) may not have the same abilities to adjust the formatting or flow of questions. Adjustments must be considered if researchers conduct similar surveys using different survey methods.

Continuous, near real-time careful examination of collected responses and updates on quota fulfillment is required with this on-line survey approach. This ensures that quotas are not overfilled (increased cost beyond planned budget) but also prevents cases of unfulfilled quotas. For example, we noticed that the USA-RATA questionnaire for the female Generation Z quota had missing responses for four consumers and we were able to recruit and field additional respondents.

## Figures and Tables

**Table 1:** Food items that were used for each food group in each of the five countries.

Countries	Starchy Foods (Carbohydrates)	Proteins Foods (Meat, Fish, Eggs)	Milk and Dairy Foods	Fruit and Vegetables	Desserts(Fats and Sugars)
Brazil	White Rice	Feijao	Milk	Bananas	Brigadeiro
China	White rice	Red braised pork belly	Soy Milk	Bananas	Pan-fried red bean paste cakes
India	White rice	Toor Dal	Milk	Bananas	Gulab Jamun
Spain	Paella	Jamón Serrano	Milk	Bananas	Turrón
USA	Baked Potato	Hamburger	Cheese	Bananas	Chocolate cake with frosting

**Table 2:** 16 eating motivation constructs and their corresponding ***positive*** sub-items that were used in the Eating Motivation Survey (EMS).

Liking	Sociability
Because it tastes good	Because it is social
Because I like it	So that I can spend time with other people
Because I have an appetite for it	Because it makes social gatherings more comfortable
**Habits**	**Price**
Because I usually eat it	Because it is inexpensive
Because I am familiar with it	Because it is on sale
Because I’m accustomed to eating it	Because I don’t want to spend any more money
**Need and Hunger**	**Visual Appeal**
Because I’m hungry	Because it spontaneously appeals to me
Because it is pleasantly filling	Because the presentation is appealing (e.g., packaging)
Because I need energy	Because I recognize it from advertisements or have seen it on TV
**Health**	**Weight Control**
Because it is healthy	Because it is low in calories
To maintain a balanced diet	Because it is low in fat
Because it keeps me in shape (e.g., energetic, motivated)	Because I watch my weight
**Convenience**	**Affect Regulation**
Because it is quick to prepare	Because I am sad
Because it is the most convenient	Because I feel lonely
Because it is easy to prepare	Because I am frustrated
**Pleasure**	**Social Norms**
Because I enjoy it	Because I am supposed to eat it
In order to indulge myself	To avoid disappointing someone who is trying to make me happy
In order to reward myself	Because it would be impolite not to eat it
**Traditional Eating**	**Social Image**
Because I grew up with it	Because others like it
Because it belongs to certain situations	Because it is trendy
Out of traditions (e.g., family traditions, special occasions)	Because it makes me look good in front of others
**Natural Concerns**	**Choice**
Because it is natural (e.g., not genetically modified)	I want to eat it every day
Because it contains no harmful substances	Because it is the only choice
Because it is organic	

**Table 3:** 16 eating motivation constructs and their corresponding ***negative*** sub-items that were used in EMS.

Liking	Sociability
I don’t like it	**Price**
It is not something I have the desire to eat at this time	The price was too high
**Habits**	**Visual Appeal**
I don’t usually eat it	I don’t like the way it looked
**Need and Hunger**	**Weight Control**
It is not filling enough	It is too high in calories
The portion size was not suitable	**Affect Regulation**
**Health**	This food makes me feel sad, lonely, or frustrated
It is not healthy	**Social Norms**
**Convenience**	I am not supposed to eat it
It is not convenient	**Social Image**
**Pleasure**	It is not a food I eat around other people
I do not want to indulge myself	Eating it makes me seem “behind the times”
**Traditional Eating**	**Choice**
I don’t think it is a snack	I had it recently and I don’t want to eat the same food too often
It is not appropriate for the situation	I would never choose this because I like to eat the same food every day
**Natural Concerns**	
It is not organic	

## Supplementary Materials

The following are available online at https://www.mdpi.com/2409-9279/3/3/49/s1, Figure S1: English-USA (Questionnaire), Figure S2: Portuguese-Brazil (Questionnaire), Figure S3: Simplified Mandarin- China (Questionnaire), Figure S4: Spanish-Spain (Questionnaire), and Figure S5: Hindi-India (Questionnaire).
