# Supplementary material for: Comparing Four Question Formats in Five Languages for On-Line Consumer Surveys
Source: Methods Protoc. 2020 Jul 14;3(3):49. doi: 10.3390/mps3030049 (PMC7563457; doi:10.3390/mps3030049)
Supplement: Supplementary file 1 [file mps-03-00049-s001.zip › supplementary/Figure S1 (USA-English).pdf]

NSAS STATE
IVERSITY

English ▾

Demographics

What is your gender?

- ◯ Male
- ◯ Female

What year were you born?

- ◯ 1943 or before
- ◯ 1944- 1964
- ◯ 1965 - 1979
- ◯ 1980 -1994
- ◯ 1995 - 2001
- ◯ 2002 or after

What is the highest degree you completed?

- ◯ Primary School or less
- ◯ High School
- ◯ College or University

How many adults live in your household including yourself?

- 1
- 2
- 3
- 4
- 5 or more

How many children under the age of 18 are in your household?

- 0
- 1
- 2
- 3
- 4 or more

We care about the quality of our survey data and hope to receive the most accurate measures of your opinions, so it is important to us that you thoughtfully provide your best answer to each question in the survey. Do you commit to providing your thoughtful and honest answers to the questions in this survey?

- I will provide my best answers
- I will not provide my best answers
- I can't promise either way

Food Involvement

The survey that you are going to take is about Eating behavior.

Please indicate your agreement to the following questions.

I do not think much about food each day.

- Strongly Disagree
- Disagree
- Disagree somewhat
- Neither agree nor disagree
- Agree somewhat
- Agree
- Strongly Agree

Cooking is not much fun.

- Strongly Disagree
- Disagree
- Disagree somewhat
- Neither agree nor disagree
- Agree somewhat
- Agree
- Strongly Agree

Talking about what I ate or am going to eat is something I like to do.

- Strongly Disagree
- Disagree
- Disagree somewhat
- Neither agree nor disagree
- Agree somewhat
- Agree
- Strongly Agree

Compared with other daily decisions, my food choices are not very important.

- Strongly Disagree

- Disagree
- Disagree somewhat
- Neither agree nor disagree
- Agree somewhat
- Agree
- Strongly Agree

When I travel, one of the things I anticipate most is eating the food there.

- Strongly Disagree
- Disagree
- Disagree somewhat
- Neither agree nor disagree
- Agree somewhat
- Agree
- Strongly Agree

I do most or all the clean up after eating.

- Strongly Disagree
- Disagree
- Disagree somewhat
- Neither agree nor disagree
- Agree somewhat
- Agree
- Strongly Agree

I enjoy cooking for others and myself.

- Strongly Disagree
- Disagree
- Disagree somewhat

- Neither agree nor disagree
- Agree somewhat
- Agree
- Strongly Agree

When I eat out, I don’t think or talk much about how the food tastes.

- Strongly Disagree
- Disagree
- Disagree somewhat
- Neither agree nor disagree
- Agree somewhat
- Agree
- Strongly Agree

I do not like to mix or chop food.

- Strongly Disagree
- Disagree
- Disagree somewhat
- Neither agree nor disagree
- Agree somewhat
- Agree
- Strongly Agree

I do most or all of my own food shopping.

- Strongly Disagree
- Disagree
- Disagree somewhat
- Neither agree nor disagree
- Agree somewhat

- Agree
- Strongly Agree

I do not wash dishes or clean the table.

- Strongly Disagree
- Disagree
- Disagree somewhat
- Neither agree nor disagree
- Agree somewhat
- Agree
- Strongly Agree

I care whether or not a table is nicely set.

- Strongly Disagree
- Disagree
- Disagree somewhat
- Neither agree nor disagree
- Agree somewhat
- Agree
- Strongly Agree

Health and Taste Attitude

I am very particular about the healthiness of food.

- Strongly Disagree
- Disagree
- Disagree somewhat
- Neither agree nor disagree

- Agree somewhat
- Agree
- Strongly Agree

I always follow a healthy and balanced diet.

- Strongly Disagree
- Disagree
- Disagree somewhat
- Neither agree nor disagree
- Agree somewhat
- Agree
- Strongly Agree

It is important to me that my diet is low in fat.

- Strongly Disagree
- Disagree
- Disagree somewhat
- Neither agree nor disagree
- Agree somewhat
- Agree
- Strongly Agree

It is important to me that my daily diet contains a lot of vitamins and minerals.

- Strongly Disagree
- Disagree
- Disagree somewhat
- Neither agree nor disagree
- Agree somewhat
- Agree

- Strongly Agree

I eat what I like and I do not worry about the healthiness of food.

- Strongly Disagree
- Disagree
- Disagree somewhat
- Neither agree nor disagree
- Agree somewhat
- Agree
- Strongly Agree

I do not avoid any foods, even if they may rise my cholesterol.

- Strongly Disagree
- Disagree
- Disagree somewhat
- Neither agree nor disagree
- Agree somewhat
- Agree
- Strongly Agree

The healthiness of food has little impact on my food choices.

- Strongly Disagree
- Disagree
- Disagree somewhat
- Neither agree nor disagree
- Agree somewhat
- Agree
- Strongly Agree

The healthiness of snack makes no difference to me.

- Strongly Disagree
- Disagree
- Disagree somewhat
- Neither agree nor disagree
- Agree somewhat
- Agree
- Strongly Agree

I do not believe that food should always be a source of pleasure.

- Strongly Disagree
- Disagree
- Disagree somewhat
- Neither agree nor disagree
- Agree somewhat
- Agree
- Strongly Agree

The appearance of food makes no difference to me.

- Strongly Disagree
- Disagree
- Disagree somewhat
- Neither agree nor disagree
- Agree somewhat
- Agree
- Strongly Agree

It is important to me to eat delicious food weekdays as well as weekends.

- Strongly Disagree
- Disagree
- Disagree somewhat
- Neither agree nor disagree
- Agree somewhat
- Agree
- Strongly Agree

When I eat, I concentrate on enjoying the taste of food.

- Strongly Disagree
- Disagree
- Disagree somewhat
- Neither agree nor disagree
- Agree somewhat
- Agree
- Strongly Agree

I finish my meal even when I do not like the taste of food.

- Strongly Disagree
- Disagree
- Disagree somewhat
- Neither agree nor disagree
- Agree somewhat
- Agree
- Strongly Agree

An essential part of my weekend is eating delicious food.

- Strongly Disagree

- Disagree
- Disagree somewhat
- Neither agree nor disagree
- Agree somewhat
- Agree
- Strongly Agree

Neophobia Questions

I am constantly sampling new and different foods.

- Strongly Disagree
- Disagree
- Disagree somewhat
- Neither agree nor disagree
- Agree somewhat
- Agree
- Strongly Agree

I don't trust new foods.

- Strongly Disagree
- Disagree
- Disagree somewhat
- Neither agree nor disagree
- Agree somewhat
- Agree
- Strongly Agree

If I don't know what is in a food, I won't try it.

- Strongly Disagree
- Disagree
- Disagree somewhat
- Neither agree nor disagree
- Agree somewhat
- Agree
- Strongly Agree

I like foods from different countries.

- Strongly Disagree
- Disagree
- Disagree somewhat
- Neither agree nor disagree
- Agree somewhat
- Agree
- Strongly Agree

Ethnic food looks too weird to eat.

- Strongly Disagree
- Disagree
- Disagree somewhat
- Neither agree nor disagree
- Agree somewhat
- Agree
- Strongly Agree

At parties, I will try a new food.

- Strongly Disagree
- Disagree

- Disagree somewhat
- Neither agree nor disagree
- Agree somewhat
- Agree
- Strongly Agree

I am afraid to eat things I have never had before.

- Strongly Disagree
- Disagree
- Disagree somewhat
- Neither agree nor disagree
- Agree somewhat
- Agree
- Strongly Agree

I am very particular about the foods I will eat.

- Strongly Disagree
- Disagree
- Disagree somewhat
- Neither agree nor disagree
- Agree somewhat
- Agree
- Strongly Agree

I will eat almost anything.

- Strongly Disagree
- Disagree
- Disagree somewhat
- Neither agree nor disagree

- Agree somewhat
- Agree
- Strongly Agree

I like to try new ethnic restaurants.

- Strongly Disagree
- Disagree
- Disagree somewhat
- Neither agree nor disagree
- Agree somewhat
- Agree
- Strongly Agree

Reasons for Starchy Food Choices (CATA)

Do you eat baked potatoes?

- Yes
- No

What are the reasons you eat baked potatoes? (check all that apply)

- ... because it contains no harmful substances (e.g. pesticides, pollutants, antibiotics)
- ... because I am frustrated
- ... to avoid disappointing someone who is trying to make me happy
- ... in order to indulge myself
- ... because I recognize it from advertisements or have seen it on TV
- ... because it is trendy
- ... because it is easy to prepare
- ... because I am supposed to eat it

- [ ] ... because I feel lonely
- [ ] ... because I feel sad
- [ ] ... because it is the most convenient
- [ ] ... because it is healthy
- [ ] ... because it is natural (e.g. not genetically modified)
- [ ] ... because I usually eat it
- [ ] ... because I watch my weight
- [ ] ... because it tastes good
- [ ] ... because I enjoy it
- [ ] ... because it makes me look good in front of others
- [ ] ... because it is social
- [ ] ... to maintain a balanced diet
- [ ] ... because I want to eat it every day
- [ ] ... because I am familiar with it
- [ ] ... because I have an appetite for it
- [ ] ... because I need energy
- [ ] ... because it is organic
- [ ] ... because it spontaneously appealed to me (e.g. situated at eye level, appealing colors)
- [ ] ... because the presentation is appealing (e.g. packaging)
- [ ] ... because it is quick to prepare
- [ ] ... because that was the only choice
- [ ] ... out of traditions (e.g. family traditions, special occasions)
- [ ] ... because it is inexpensive
- [ ] ... because I grew up with it
- [ ] ... so that I can spend time with other people
- [ ] ... because I am accustomed to eating it
- [ ] ... because it is pleasantly filling
- [ ] ... because it belongs to certain situations
- [ ] ... in order to reward myself
- [ ] ... because it makes social gatherings more comfortable
- [ ] ... because it would be impolite not to eat it
- [ ] ... because I was hungry

- ☐ ... because I don't want to spend any more money
- ☐ ... because it is on sale
- ☐ ... because it is low in calories
- ☐ ... because it is low in fat
- ☐ ... because others like it
- ☐ ... because I like it
- ☐ ... because it keeps me in shape (e.g. energetic, motivated)

What are the reasons you do not eat baked potatoes (check all that apply)

- ☐ I don't usually eat it
- ☐ the portion size was not suitable
- ☐ I do not want to indulge myself
- ☐ I don't think it is a snack
- ☐ it is too high in calories
- ☐ the price is too high
- ☐ this food makes me feel sad, lonely, or frustrated
- ☐ it is not appropriate for the situation
- ☐ it was not something I have desire to eat at this time
- ☐ I had it recently and I don't want to eat the same food too often
- ☐ I don't like it
- ☐ eating it makes me seem "behind the times"
- ☐ I would never choose this because I like to eat the same food every day.
- ☐ I am not supposed to eat it
- ☐ it is not filling enough
- ☐ it is not organic
- ☐ I don't like the way it looks
- ☐ it is not a food I eat around other people
- ☐ it is not healthy
- ☐ it is not convenient

Reasons for Protein foods Choices(CATA)

Do you eat hamburgers?

- ○ Yes
- ○ No

What are the reasons you eat hamburgers? (check all that apply)

- ☐ ... because I don't want to spend any more money
- ☐ ... because I grew up with it
- ☐ ... because it would be impolite not to eat it
- ☐ ... because I feel lonely
- ☐ ... because it keeps me in shape (e.g. energetic, motivated)
- ☐ ... in order to indulge myself
- ☐ ... because it spontaneously appealed to me (e.g. situated at eye level, appealing colors)
- ☐ ... because it is trendy
- ☐ ... because the presentation is appealing (e.g. packaging)
- ☐ ... because it is organic
- ☐ ... because others like it
- ☐ ... because it is low in fat
- ☐ ... to maintain a balanced diet
- ☐ ... because it is pleasantly filling
- ☐ ... because it is social
- ☐ ... because it contains no harmful substances (e.g. pesticides, pollutants, antibiotics)
- ☐ ... because I am accustomed to eating it
- ☐ ... because it belongs to certain situations
- ☐ ... so that I can spend time with other people
- ☐ ... out of traditions (e.g. family traditions, special occasions)
- ☐ ... because I want to eat it every day
- ☐ ... because I am familiar with it

- [ ] ... because it is low in calories
- [ ] ... because I am supposed to eat it
- [ ] ... because it is natural (e.g. not genetically modified)
- [ ] ... because I need energy
- [ ] ... because it is inexpensive
- [ ] ... because I was hungry
- [ ] ... because it makes social gatherings more comfortable
- [ ] ... because I am frustrated
- [ ] ... because I feel sad
- [ ] ... because I usually eat it
- [ ] ... because I have an appetite for it
- [ ] ... because I like it
- [ ] ... because I watch my weight
- [ ] ... because it tastes good
- [ ] ... because I recognize it from advertisements or have seen it on TV
- [ ] ... because it makes me look good in front of others
- [ ] ... because that was the only choice
- [ ] ... to avoid disappointing someone who is trying to make me happy
- [ ] ... in order to reward myself
- [ ] ... because it is on sale
- [ ] ... because it is easy to prepare
- [ ] ... because I enjoy it
- [ ] ... because it is quick to prepare
- [ ] ... because it is healthy
- [ ] ... because it is the most convenient

What are the reasons you do not eat hamburgers (check all that apply)

- [ ] I am not supposed to eat it
- [ ] it is not healthy
- [ ] the portion size was not suitable
- [ ] it is not organic

- ☐ eating it makes me seem "behind the times"
- ☐ it is not appropriate for the situation
- ☐ the price was too high
- ☐ it is not something I have desire to eat at this time
- ☐ I don't usually eat it
- ☐ it is not filling enough
- ☐ it is too high in calories
- ☐ I don't like the way it looked
- ☐ I would never choose this because I like to eat the same food every day.
- ☐ I had it recently and I don't want to eat the same food too often
- ☐ it is not convenient
- ☐ I don't think it is a snack
- ☐ I do not want to indulge myself
- ☐ I don't like it
- ☐ this food makes me feel sad, lonely, or frustrated
- ☐ it is not a food I eat around other people

Reasons for Dairy Food Choices(CATA)

Do you eat american cheese?

- ○ Yes
- ○ No

What are the reasons you eat american cheese? (check all that apply)

- ☐ ... because I have an appetite for it
- ☐ ... to avoid disappointing someone who is trying to make me happy
- ☐ ... in order to indulge myself
- ☐ ... because that was the only choice
- ☐ ... because it is easy to prepare

- [ ] ... because it is natural (e.g. not genetically modified)
- [ ] ... because it is quick to prepare
- [ ] ... because it is healthy
- [ ] ... because it is trendy
- [ ] ... because I need energy
- [ ] ... because I recognize it from advertisements or have seen it on TV
- [ ] ... because I am supposed to eat it
- [ ] ... because I am frustrated
- [ ] ... because it is organic
- [ ] ... because it is inexpensive
- [ ] ... because I grew up with it
- [ ] ... because I like it
- [ ] ... because I don't want to spend any more money
- [ ] ... because I am accustomed to eating it
- [ ] ... because it is on sale
- [ ] ... because it makes social gatherings more comfortable
- [ ] ... out of traditions (e.g. family traditions, special occasions)
- [ ] ... because it belongs to certain situations
- [ ] ... because I am familiar with it
- [ ] ... because the presentation is appealing (e.g. packaging)
- [ ] ... because it tastes good
- [ ] ... because I enjoy it
- [ ] ... because I watch my weight
- [ ] ... because it is the most convenient
- [ ] ... because I feel lonely
- [ ] ... because it is low in fat
- [ ] ... because it spontaneously appealed to me (e.g. situated at eye level, appealing colors)
- [ ] ... because it is pleasantly filling
- [ ] ... because it makes me look good in front of others
- [ ] ... because I was hungry
- [ ] ... in order to reward myself
- [ ] ... to maintain a balanced diet

- ☐ ... because it is low in calories
- ☐ ... so that I can spend time with other people
- ☐ ... because it keeps me in shape (e.g. energetic, motivated)
- ☐ ... because it is social
- ☐ ... because it contains no harmful substances (e.g. pesticides, pollutants, antibiotics)
- ☐ ... because I usually eat it
- ☐ ... because others like it
- ☐ ... because I feel sad
- ☐ ... because I want to eat it every day
- ☐ ... because it would be impolite not to eat it

What are the reasons you do not eat american cheese (check all that apply)

- ☐ I don't think it is a snack
- ☐ I don't usually eat it
- ☐ I don't like the way it looks
- ☐ this food makes me feel sad, lonely, or frustrated
- ☐ the price is too high
- ☐ it is too high in calories
- ☐ the portion size was not suitable
- ☐ it is not convenient
- ☐ it is not a food I eat around other people
- ☐ it is not organic
- ☐ I do not want to indulge myself
- ☐ I would never choose this because I like to eat the same food every day.
- ☐ it is not something I have desire to eat at this time
- ☐ I had it recently and I don't want to eat the same food too often
- ☐ it is not filling enough
- ☐ eating it makes me seem "behind the times"
- ☐ it is not appropriate for the situation
- ☐ I am not supposed to eat it

- [ ] I don't like it
- [ ] it is not healthy

Reasons for Fruit Choices(CATA)

Do you eat bananas?

- ( ) Yes
- ( ) No

What are the reasons you eat bananas? (check all that apply)

- [ ] ... because it is low in calories
- [ ] ... because it is the most convenient
- [ ] ... because I am supposed to eat it
- [ ] ... because it would be impolite not to eat it
- [ ] ... because it makes me look good in front of others
- [ ] ... because I want to eat it every day
- [ ] ... because I am accustomed to eating it
- [ ] ... because others like it
- [ ] ... because I like it
- [ ] ... because it is natural (e.g. not genetically modified)
- [ ] ... in order to indulge myself
- [ ] ... because it spontaneously appealed to me (e.g. situated at eye level, appealing colors)
- [ ] ... because I usually eat it
- [ ] ... because it is low in fat
- [ ] ... because it keeps me in shape (e.g. energetic, motivated)
- [ ] ... to maintain a balanced diet
- [ ] ... because I watch my weight
- [ ] ... because I need energy

- ☐ ... because it contains no harmful substances (e.g. pesticides, pollutants, antibiotics)
- ☐ ... because it makes social gatherings more comfortable
- ☐ ... in order to reward myself
- ☐ ... out of traditions (e.g. family traditions, special occasions)
- ☐ ... because I recognize it from advertisements or have seen it on TV
- ☐ ... because I was hungry
- ☐ ... because I feel lonely
- ☐ ... because it is quick to prepare
- ☐ ... because I am frustrated
- ☐ ... because it is healthy
- ☐ ... because I have an appetite for it
- ☐ ... because it is on sale
- ☐ ... because that was the only choice
- ☐ ... because I enjoy it
- ☐ ... so that I can spend time with other people
- ☐ ... because it is pleasantly filling
- ☐ ... because I don't want to spend any more money
- ☐ ... because the presentation is appealing (e.g. packaging)
- ☐ ... because it is organic
- ☐ ... because it is trendy
- ☐ ... to avoid disappointing someone who is trying to make me happy
- ☐ ... because it is easy to prepare
- ☐ ... because it belongs to certain situations
- ☐ ... because I am familiar with it
- ☐ ... because it tastes good
- ☐ ... because it is inexpensive
- ☐ ... because I grew up with it
- ☐ ... because it is social
- ☐ ... because I feel sad

What are the reasons you do not eat bananas (check all that apply)

- [ ] I would never choose this because I like to eat the same food every day.
- [ ] it is not organic
- [ ] it is not convenient
- [ ] I don't usually eat it
- [ ] I don't think it is a snack
- [ ] it is not filling enough
- [ ] it is not appropriate for the situation
- [ ] I don't like the way it looks
- [ ] the portion size was not suitable
- [ ] I had it recently and I don't want to eat the same food too often
- [ ] I am not supposed to eat it
- [ ] it is not a food I eat around other people
- [ ] this food makes me feel sad, lonely, or frustrated
- [ ] eating it makes me seem "behind the times"
- [ ] it is not something I have desire to eat at this time
- [ ] it is not healthy
- [ ] I do not want to indulge myself
- [ ] the price is too high
- [ ] it is too high in calories
- [ ] I don't like it

Reasons for Sweet food Choices(CATA)

Do you eat chocolate cake with frosting?

- ○ Yes
- ○ No

What are the reasons you ate chocolate cake with frosting? (check all that apply)

- [ ] ... in order to reward myself

- ☐ ... because it is inexpensive
- ☐ ... because it is pleasantly filling
- ☐ ... because it is low in fat
- ☐ ... because others like it
- ☐ ... because that was the only choice
- ☐ ... because it is quick to prepare
- ☐ ... because I grew up with it
- ☐ ... because I feel sad
- ☐ ... because it is organic
- ☐ ... because I recognize it from advertisements or have seen it on TV
- ☐ ... because it keeps me in shape (e.g. energetic, motivated)
- ☐ ... out of traditions (e.g. family traditions, special occasions)
- ☐ ... because it is the most convenient
- ☐ ... because I am supposed to eat it
- ☐ ... because I like it
- ☐ ... because it tastes good
- ☐ ... because I was hungry
- ☐ ... because I watch my weight
- ☐ ... because it is on sale
- ☐ ... because I am accustomed to eating it
- ☐ ... because I usually eat it
- ☐ ... because it belongs to certain situations
- ☐ ... because it is easy to prepare
- ☐ ... to avoid disappointing someone who is trying to make me happy
- ☐ ... in order to indulge myself
- ☐ ... because the presentation is appealing (e.g. packaging)
- ☐ ... because I want to eat it every day
- ☐ ... to maintain a balanced diet
- ☐ ... because I don't want to spend any more money
- ☐ ... because it is trendy
- ☐ ... because it contains no harmful substances (e.g. pesticides, pollutants, antibiotics)
- ☐ ... because it is natural (e.g. not genetically modified)

- [ ] ... because it is social
- [ ] ... because I am frustrated
- [ ] ... because I enjoy it
- [ ] ... because it is healthy
- [ ] ... because I have an appetite for it
- [ ] ... because I need energy
- [ ] ... because it makes social gatherings more comfortable
- [ ] ... because I feel lonely
- [ ] ... because it is low in calories
- [ ] ... because it spontaneously appealed to me (e.g. situated at eye level, appealing colors)
- [ ] ... because I am familiar with it
- [ ] ... because it would be impolite not to eat it
- [ ] ... because it makes me look good in front of others
- [ ] ... so that I can spend time with other people

What are the reasons you do not eat chocolate cake with frosting (check all that apply)

- [ ] I do not not want to indulge myself
- [ ] I don't like it
- [ ] it is not something I have desire to eat at this time
- [ ] I would never choose this because I like to eat the same food every day.
- [ ] the price is too high
- [ ] it is not filling enough
- [ ] I don't think it is a snack
- [ ] it is not a food I eat around other people
- [ ] the portion size was not suitable
- [ ] it is not appropriate for the situation
- [ ] I am not supposed to eat it
- [ ] it is not organic
- [ ] eating it makes me seem "behind the times"

- ☐ I don't like the way it looks
- ☐ I don't usually eat it
- ☐ it is not convenient
- ☐ I had it recently and I don't want to eat the same food too often
- ☐ it is too high in calories
- ☐ it is not healthy
- ☐ this food makes me feel sad, lonely, or frustrated

JAR & Liking

How short/long did you think this survey was?

- ○ Much Too Short
- ○ Too Short
- ○ A Little Too Short
- ○ Just About Right
- ○ A Little Too Long
- ○ Too Long
- ○ Much Too Long

How did you feel about taking this survey after you were finished?

- ○ I liked it a lot
- ○ I liked taking it
- ○ I have no feelings either way
- ○ I did not like taking it
- ○ I hated taking it

Reasons for Starchy Food Choices(Full CATA)

Do you eat baked potatoes?

- Yes
- No

Do you eat baked potatoes ...because you are accustomed to eating it?

- Yes
- No

Do you eat baked potatoes ...because it is healthy?

- Yes
- No

Do you eat baked potatoes ...because it is quick to prepare?

- Yes
- No

Do you eat baked potatoes ...because it tastes good ?

- Yes
- No

Do you eat baked potatoes...in order to indulge yourself?

- Yes
- No

D you eat baked potatoes...because it is natural (e.g. not genetically modified)?

- Yes
- No

Do you eat baked potatoes...because you don't want to spend any more money?

- Yes
- No

Do you eat baked potatoes...because it is low in calories?

- Yes
- No

Do you eat baked potatoes...because you are frustrated?

- Yes
- No

Do you eat baked potatoes...because it makes you look good in front of others?

- Yes
- No

Do you eat baked potatoes...because it would be impolite not to eat it?

- Yes
- No

Do you eat baked potatoes...because the presentation is appealing (e.g. packaging)?

- Yes
- No

Do you eat baked potatoes...so that you can spend time with other people?

- Yes
- No

Do you eat baked potatoes...because it is organic?

- Yes
- No

Do you eat baked potatoes...because you grew up with it?

- Yes
- No

Do you eat baked potatoes...because you enjoy it?

- Yes
- No

Do you eat baked potatoes...because you are hungry?

- Yes
- No

Do you eat baked potatoes...because you usually eat it?

- Yes
- No

Do you eat baked potatoes...because you have an appetite for it?

- Yes
- No

Do you eat baked potatoes...because it is easy to prepare?

- Yes
- No

Do you eat baked potatoes...because you feel sad?

- Yes
- No

Do you eat baked potatoes...because it is low in fat?

- Yes
- No

Do you eat baked potatoes...because you recognize it from advertisements or have seen it on TV?

- Yes
- No

Do you eat baked potatoes...because it makes social gatherings more comfortable?

- Yes
- No

Do you eat baked potatoes...because you are supposed to eat it?

- Yes
- No

Do you eat baked potatoes...because others like it?

- Yes
- No

Do you eat baked potatoes...because you feel lonely?

- Yes
- No

Do you eat baked potatoes...because you watch your weight?

- Yes
- No

Do you eat baked potatoes...because it contains no harmful substances (e.g. pesticides, pollutants, antibiotics)?

- Yes
- No

Do you eat baked potatoes...because it is the most convenient?

- Yes
- No

Do you eat baked potatoes...because you need energy?

- Yes
- No

Do you eat baked potatoes...because you are familiar with it?

- Yes
- No

Do you eat baked potatoes...because you like it?

- Yes
- No

Do you eat baked potatoes...because it is pleasantly filling?

- Yes
- No

Do you eat baked potatoes...in order to reward yourself?

- Yes
- No

Do you eat baked potatoes...out of traditions (e.g. family traditions, special occasions)?

- Yes
- No

Do you eat baked potatoes...because it belongs to certain situations?

- Yes
- No

Do you eat baked potatoes...to maintain a balanced diet?

- Yes
- No

Do you eat baked potatoes...because it is social?

- Yes
- No

Do you eat baked potatoes...because it is inexpensive?

- Yes
- No

Do you eat baked potatoes...because it spontaneously appeals to you (e.g. situated at eye level, appealing colors)?

- Yes

- No

Do you eat baked potatoes...to avoid disappointing someone who is trying to make you happy?

- Yes
- No

Do you eat baked potatoes...because it is trendy?

- Yes
- No

Do you eat baked potatoes...because it is on sale?

- Yes
- No

Do you eat baked potatoes...because it keeps you in shape (e.g. energetic, motivated)?

- Yes
- No

Do you eat baked potatoes...because that is the only choice?

- Yes
- No

Do you eat baked potatoes...because you want to eat it every day?

- Yes
- No

Do you not eat baked potatoes...because you don't think it is a snack?

- Yes
- No

Do you not eat baked potatoes ...because you don't like it?

- Yes
- No

Do you not eat baked potatoes ...because you don't usually eat it?

- Yes
- No

Do you not eat baked potatoes ...because the portion size is not suitable?

- Yes
- No

Do you not eat baked potatoes ...because it is not filling enough?

- Yes
- No

Do you not eat baked potatoes ...because it is not healthy?

- Yes
- No

Do you not eat baked potatoes ...because it is not convenient?

- Yes
- No

Do you not eat baked potatoes ...because you do not want to indulge yourself?

- Yes
- No

Do you not eat baked potatoes ...because it is not appropriate for the situation?

- Yes
- No

Do you not eat baked potatoes ...because it is not a food you eat around other people?

- Yes
- No

Do you not eat baked potatoes ...because the price is too high?

- Yes
- No

Do you not eat baked potatoes ...because you don't like the way it looks?

- Yes
- No

Do you not eat baked potatoes ...because it is too high in calories?

- Yes
- No

Do you not eat baked potatoes ...because you are not supposed to eat it?

- Yes
- No

Do you not eat baked potatoes ...because eating it makes you seem "behind the times"?

- Yes
- No

Do you not eat baked potatoes ...because it is not something you have desire to eat at this time?

- Yes

- No

Do you not eat baked potatoes ...because you had it recently and don't want to eat the same food too often?

- Yes
- No

Do you not eat baked potatoes ...because you like to eat the same food every day?

- Yes
- No

Do you not eat baked potatoes ...because this food makes you feel sad, lonely, or frustrated?

- Yes
- No

Do you not eat baked potatoes ...because it is not organic?

- Yes
- No

Reasons for Protein foods Choices(Full CATA)

Do you eat hamburgers?

- Yes
- No

Do you eat hamburgers ...because you are accustomed to eating it?

- Yes
- No

Do you eat hamburgers ...because it is healthy?

- Yes
- No

Do you eat hamburgers ...because it is quick to prepare?

- Yes
- No

Do you eat hamburgers ...because it tastes good ?

- Yes
- No

Do you eat hamburgers...in order to indulge yourself?

- Yes
- No

D you eat hamburgers...because it is natural (e.g. not genetically modified)?

- Yes
- No

Do you eat hamburgers...because you don't want to spend any more money?

- Yes
- No

Do you eat hamburgers...because it is low in calories?

- Yes
- No

Do you eat hamburgers...because you are frustrated?

- Yes
- No

Do you eat hamburgers...because it makes you look good in front of others?

- Yes
- No

Do you eat hamburgers...because it would be impolite not to eat it?

- Yes
- No

Do you eat hamburgers...because the presentation is appealing (e.g. packaging)?

- Yes
- No

Do you eat hamburgers...so that you can spend time with other people?

- Yes
- No

Do you eat hamburgers...because it is organic?

- Yes
- No

Do you eat hamburgers...because you grew up with it?

- Yes
- No

Do you eat hamburgers...because you enjoy it?

- Yes
- No

Do you eat hamburgers...because you are hungry?

- Yes
- No

Do you eat hamburgers...because you usually eat it?

- Yes
- No

Do you eat hamburgers...because you have an appetite for it?

- Yes
- No

Do you eat hamburgers...because it is easy to prepare?

- Yes
- No

Do you eat hamburgers...because you feel sad?

- Yes
- No

Do you eat hamburgers...because it is low in fat?

- Yes
- No

Do you eat hamburgers...because you recognize it from advertisements or have seen it on TV?

- Yes
- No

Do you eat hamburgers...because it makes social gatherings more comfortable?

- Yes
- No

Do you eat hamburgers...because you are supposed to eat it?

- Yes
- No

Do you eat hamburgers...because others like it?

- Yes
- No

Do you eat hamburgers...because you feel lonely?

- Yes
- No

Do you eat hamburgers...because you watch your weight?

- Yes
- No

Do you eat hamburgers...because it contains no harmful substances (e.g. pesticides, pollutants, antibiotics)?

- Yes
- No

Do you eat hamburgers...because it is the most convenient?

- Yes
- No

Do you eat hamburgers...because you need energy?

- Yes
- No

Do you eat hamburgers...because you are familiar with it?

- Yes
- No

Do you eat hamburgers...because you like it?

- Yes
- No

Do you eat hamburgers...because it is pleasantly filling?

- Yes
- No

Do you eat hamburgers...in order to reward yourself?

- Yes
- No

Do you eat hamburgers...out of traditions (e.g. family traditions, special occasions)?

- Yes
- No

Do you eat hamburgers...because it belongs to certain situations?

- Yes
- No

Do you eat hamburgers...to maintain a balanced diet?

- Yes
- No

Do you eat hamburgers...because it is social?

- Yes
- No

Do you eat hamburgers...because it is inexpensive?

- Yes
- No

Do you eat hamburgers...because it spontaneously appeals to you (e.g. situated at eye level, appealing colors)?

- Yes
- No

Do you eat hamburgers...to avoid disappointing someone who is trying to make you happy?

- Yes
- No

Do you eat hamburgers...because it is trendy?

- Yes
- No

Do you eat hamburgers...because it is on sale?

- Yes
- No

Do you eat hamburgers...because it keeps you in shape (e.g. energetic, motivated)?

- Yes
- No

Do you eat hamburgers...because that is the only choice?

- Yes
- No

Do you eat hamburgers...because you want to eat it every day?

- Yes
- No

Do you not eat hamburgers...because you don't think it is a snack?

- Yes
- No

Do you not eat hamburgers ...because you don't like it?

- Yes
- No

Do you not eat hamburgers ...because you don't usually eat it?

- Yes
- No

Do you not eat hamburgers ...because the portion size is not suitable?

- Yes
- No

Do you not eat hamburgers ...because it is not filling enough?

- Yes
- No

Do you not eat hamburgers ...because it is not healthy?

- Yes
- No

Do you not eat hamburgers ...because it is not convenient?

- Yes
- No

Do you not eat hamburgers ...because you do not want to indulge yourself?

- Yes
- No

Do you not eat hamburgers ...because it is not appropriate for the situation?

- Yes
- No

Do you not eat hamburgers ...because it is not a food you eat around other people?

- Yes
- No

Do you not eat hamburgers ...because the price is too high?

- Yes
- No

Do you not eat hamburgers ...because you don't like the way it looks?

- Yes
- No

Do you not eat hamburgers ...because it is too high in calories?

- Yes
- No

Do you not eat hamburgers ...because you are not supposed to eat it?

- Yes
- No

Do you not eat hamburgers ...because eating it makes you seem "behind the times"?

- Yes
- No

Do you not eat hamburgers ...because it is not something you have desire to eat at this time?

- Yes
- No

Do you not eat hamburgers ...because you had it recently and don't want to eat the same food too often?

- Yes

○ No

Do you not eat hamburgers ...because you like to eat the same food every day?

○ Yes
○ No

Do you not eat hamburgers ...because this food makes you feel sad, lonely, or frustrated?

○ Yes
○ No

Do you not eat hamburgers ...because it is not organic?

○ Yes
○ No

Reasons for Dairy Food Choices(Full CATA)

Do you eat american cheese?

○ Yes
○ No

Do you eat american cheese ...because you are accustomed to eating it?

○ Yes
○ No

Do you eat american cheese ...because it is healthy?

- Yes
- No

Do you eat american cheese ...because it is quick to prepare?

- Yes
- No

Do you eat american cheese ...because it tastes good ?

- Yes
- No

Do you eat american cheese...in order to indulge yourself?

- Yes
- No

D you eat american cheese...because it is natural (e.g. not genetically modified)?

- Yes
- No

Do you eat american cheese...because you don't want to spend any more money?

- Yes
- No

Do you eat american cheese...because it is low in calories?

- Yes
- No

Do you eat american cheese...because you are frustrated?

- Yes
- No

Do you eat american cheese...because it makes you look good in front of others?

- Yes
- No

Do you eat american cheese...because it would be impolite not to eat it?

- Yes
- No

Do you eat american cheese...because the presentation is appealing (e.g. packaging)?

- Yes
- No

Do you eat american cheese...so that you can spend time with other people?

- Yes
- No

Do you eat american cheese...because it is organic?

- Yes
- No

Do you eat american cheese...because you grew up with it?

- Yes
- No

Do you eat american cheese...because you enjoy it?

- Yes
- No

Do you eat american cheese...because you are hungry?

- Yes
- No

Do you eat american cheese...because you usually eat it?

- Yes
- No

Do you eat american cheese...because you have an appetite for it?

- Yes
- No

Do you eat american cheese...because it is easy to prepare?

- Yes
- No

Do you eat american cheese...because you feel sad?

- Yes
- No

Do you eat american cheese...because it is low in fat?

- Yes
- No

Do you eat american cheese...because you recognize it from advertisements or have seen it on TV?

- Yes
- No

Do you eat american cheese...because it makes social gatherings more comfortable?

- Yes
- No

Do you eat american cheese...because you are supposed to eat it?

- Yes
- No

Do you eat american cheese...because others like it?

- Yes
- No

Do you eat american cheese...because you feel lonely?

- Yes
- No

Do you eat american cheese...because you watch your weight?

- Yes
- No

Do you eat american cheese...because it contains no harmful substances (e.g. pesticides, pollutants, antibiotics)?

- Yes
- No

Do you eat american cheese...because it is the most convenient?

- Yes
- No

Do you eat american cheese...because you need energy?

- Yes
- No

Do you eat american cheese...because you are familiar with it?

- Yes
- No

Do you eat american cheese...because you like it?

- Yes
- No

Do you eat american cheese...because it is pleasantly filling?

- Yes
- No

Do you eat american cheese...in order to reward yourself?

- Yes
- No

Do you eat american cheese...out of traditions (e.g. family traditions, special occasions)?

- Yes
- No

Do you eat american cheese...because it belongs to certain situations?

- Yes
- No

Do you eat american cheese...to maintain a balanced diet?

- Yes
- No

Do you eat american cheese...because it is social?

- Yes
- No

Do you eat american cheese...because it is inexpensive?

- Yes
- No

Do you eat american cheese...because it spontaneously appeals to you (e.g. situated at eye level, appealing colors)?

- Yes
- No

Do you eat american cheese...to avoid disappointing someone who is trying to make you happy?

- Yes
- No

Do you eat american cheese...because it is trendy?

- Yes
- No

Do you eat american cheese...because it is on sale?

- Yes
- No

Do you eat american cheese...because it keeps you in shape (e.g. energetic, motivated)?

- Yes
- No

Do you eat american cheese...because that is the only choice?

- Yes
- No

Do you eat american cheese...because you want to eat it every day?

- Yes
- No

Do you not eat american cheese...because you don't think it is a snack?

- Yes
- No

Do you not eat american cheese ...because you don't like it?

- Yes
- No

Do you not eat american cheese ...because you don't usually eat it?

- Yes
- No

Do you not eat american cheese ...because the portion size is not suitable?

- Yes
- No

Do you not eat american cheese ...because it is not filling enough?

- Yes
- No

Do you not eat american cheese ...because it is not healthy?

- Yes
- No

Do you not eat american cheese ...because it is not convenient?

- Yes
- No

Do you not eat american cheese ...because you do not want to indulge yourself?

- Yes
- No

Do you not eat american cheese ...because it is not appropriate for the situation?

- Yes
- No

Do you not eat american cheese ...because it is not a food you eat around other people?

- Yes
- No

Do you not eat american cheese ...because the price is too high?

- Yes
- No

Do you not eat american cheese ...because you don't like the way it looks?

- Yes
- No

Do you not eat american cheese ...because it is too high in calories?

- Yes
- No

Do you not eat american cheese ...because you are not supposed to eat it?

- Yes
- No

Do you not eat american cheese ...because eating it makes you seem "behind the times"?

- Yes
- No

Do you not eat american cheese ...because it is not something you have desire to eat at this time?

- Yes
- No

Do you not eat american cheese ...because you had it recently and don't want to eat the same food too often?

- Yes
- No

Do you not eat american cheese ...because you like to eat the same food every day?

- Yes
- No

Do you not eat american cheese ...because this food makes you feel sad, lonely, or frustrated?

- Yes
- No

Do you not eat american cheese ...because it is not organic?

- Yes
- No

Reasons for Fruit Choices(Full CATA)

Do you eat bananas?

- Yes
- No

Do you eat bananas ...because you are accustomed to eating it?

- Yes
- No

Do you eat bananas ...because it is healthy?

- Yes
- No

Do you eat bananas ...because it is quick to prepare?

- Yes
- No

Do you eat bananas ...because it tastes good ?

- Yes
- No

Do you eat bananas...in order to indulge yourself?

- Yes
- No

D you eat bananas...because it is natural (e.g. not genetically modified)?

- Yes
- No

Do you eat bananas...because you don't want to spend any more money?

- Yes
- No

Do you eat bananas...because it is low in calories?

- Yes
- No

Do you eat bananas...because you are frustrated?

- Yes
- No

Do you eat bananas...because it makes you look good in front of others?

- Yes
- No

Do you eat bananas...because it would be impolite not to eat it?

- Yes
- No

Do you eat bananas...because the presentation is appealing (e.g. packaging)?

- Yes
- No

Do you eat bananas...so that you can spend time with other people?

- Yes
- No

Do you eat bananas...because it is organic?

- Yes
- No

Do you eat bananas...because you grew up with it?

- Yes
- No

Do you eat bananas...because you enjoy it?

- Yes
- No

Do you eat bananas...because you are hungry?

- Yes
- No

Do you eat bananas...because you usually eat it?

- Yes
- No

Do you eat bananas...because you have an appetite for it?

- Yes
- No

Do you eat bananas...because it is easy to prepare?

- Yes
- No

Do you eat bananas...because you feel sad?

- Yes
- No

Do you eat bananas...because it is low in fat?

- Yes
- No

Do you eat bananas...because you recognize it from advertisements or have seen it on TV?

- Yes
- No

Do you eat bananas...because it makes social gatherings more comfortable?

- Yes
- No

Do you eat bananas...because you are supposed to eat it?

- Yes
- No

Do you eat bananas...because others like it?

- Yes
- No

Do you eat bananas...because you feel lonely?

- Yes
- No

Do you eat bananas...because you watch your weight?

- Yes
- No

Do you eat bananas...because it contains no harmful substances (e.g. pesticides, pollutants, antibiotics)?

- Yes
- No

Do you eat bananas...because it is the most convenient?

- Yes
- No

Do you eat bananas...because you need energy?

- Yes
- No

Do you eat bananas...because you are familiar with it?

- Yes
- No

Do you eat bananas...because you like it?

- Yes
- No

Do you eat bananas...because it is pleasantly filling?

- Yes
- No

Do you eat bananas...in order to reward yourself?

- Yes
- No

Do you eat bananas...out of traditions (e.g. family traditions, special occasions)?

- Yes
- No

Do you eat bananas...because it belongs to certain situations?

- Yes
- No

Do you eat bananas...to maintain a balanced diet?

- Yes
- No

Do you eat bananas...because it is social?

- Yes
- No

Do you eat bananas...because it is inexpensive?

- Yes
- No

Do you eat bananas...because it spontaneously appeals to you (e.g. situated at eye level, appealing colors)?

- Yes
- No

Do you eat bananas...to avoid disappointing someone who is trying to make you happy?

- Yes
- No

Do you eat bananas...because it is trendy?

- Yes
- No

Do you eat bananas...because it is on sale?

- Yes
- No

Do you eat bananas...because it keeps you in shape (e.g. energetic, motivated)?

- Yes
- No

Do you eat bananas...because that is the only choice?

- Yes
- No

Do you eat bananas...because you want to eat it every day?

- Yes
- No

Do you not eat bananas...because you don't think it is a snack?

- Yes
- No

Do you not eat bananas ...because you don't like it?

- Yes
- No

Do you not eat bananas ...because you don't usually eat it?

- Yes
- No

Do you not eat bananas ...because the portion size is not suitable?

- Yes
- No

Do you not eat bananas ...because it is not filling enough?

- Yes
- No

Do you not eat bananas ...because it is not healthy?

- Yes
- No

Do you not eat bananas ...because it is not convenient?

- Yes
- No

Do you not eat bananas ...because you do not want to indulge yourself?

- Yes
- No

Do you not eat bananas ...because it is not appropriate for the situation?

- Yes
- No

Do you not eat bananas ...because it is not a food you eat around other people?

- Yes
- No

Do you not eat bananas ...because the price is too high?

- Yes
- No

Do you not eat bananas ...because you don't like the way it looks?

- Yes
- No

Do you not eat bananas ...because it is too high in calories?

- Yes
- No

Do you not eat bananas ...because you are not supposed to eat it?

- Yes

- No

Do you not eat bananas ...because eating it makes you seem "behind the times"?

- Yes
- No

Do you not eat bananas ...because it is not something you have desire to eat at this time?

- Yes
- No

Do you not eat bananas ...because you had it recently and don't want to eat the same food too often?

- Yes
- No

Do you not eat bananas ...because you like to eat the same food every day?

- Yes
- No

Do you not eat bananas ...because this food makes you feel sad, lonely, or frustrated?

- Yes
- No

Do you not eat bananas ...because it is not organic?

- Yes
- No

Reasons for Sweet food Choices(Full CATA)

Do you eat chocolate cake with frosting ?

- Yes
- No

Do you eat chocolate cake with frosting ...because you are accustomed to eating it?

- Yes
- No

Do you eat chocolate cake with frosting ...because it is healthy?

- Yes
- No

Do you eat chocolate cake with frosting ...because it is quick to prepare?

- Yes
- No

Do you eat chocolate cake with frosting ...because it tastes good ?

- Yes

- No

Do you eat chocolate cake with frosting ...in order to indulge yourself?

- Yes
- No

D you eat chocolate cake with frosting ...because it is natural (e.g. not genetically modified)?

- Yes
- No

Do you eat chocolate cake with frosting ...because you don't want to spend any more money?

- Yes
- No

Do you eat chocolate cake with frosting ...because it is low in calories?

- Yes
- No

Do you eat chocolate cake with frosting ...because you are frustrated?

- Yes
- No

Do you eat chocolate cake with frosting ...because it makes you look good in front of others?

- Yes
- No

Do you eat chocolate cake with frosting ...because it would be impolite not to eat it?

- Yes
- No

Do you eat chocolate cake with frosting ...because the presentation is appealing (e.g. packaging)?

- Yes
- No

Do you eat chocolate cake with frosting ...so that you can spend time with other people?

- Yes
- No

Do you eat chocolate cake with frosting ...because it is organic?

- Yes
- No

Do you eat chocolate cake with frosting ...because you grew up with it?

- Yes
- No

Do you eat chocolate cake with frosting ...because you enjoy it?

- Yes
- No

Do you eat chocolate cake with frosting ...because you are hungry?

- Yes
- No

Do you eat chocolate cake with frosting ...because you usually eat it?

- Yes
- No

Do you eat chocolate cake with frosting ...because you have an appetite for it?

- Yes
- No

Do you eat chocolate cake with frosting ...because it is easy to prepare?

- Yes
- No

Do you eat chocolate cake with frosting ...because you feel sad?

- Yes

No

Do you eat chocolate cake with frosting ...because it is low in fat?

Yes

No

Do you eat chocolate cake with frosting ...because you recognize it from advertisements or have seen it on TV?

Yes

No

Do you eat chocolate cake with frosting ...because it makes social gatherings more comfortable?

Yes

No

Do you eat chocolate cake with frosting ...because you are supposed to eat it?

Yes

No

Do you eat chocolate cake with frosting ...because others like it?

Yes

No

Do you eat chocolate cake with frosting ...because you feel lonely?

- Yes
- No

Do you eat chocolate cake with frosting ...because you watch your weight?

- Yes
- No

Do you eat chocolate cake with frosting ...because it contains no harmful substances (e.g. pesticides, pollutants, antibiotics)?

- Yes
- No

Do you eat chocolate cake with frosting ...because it is the most convenient?

- Yes
- No

Do you eat chocolate cake with frosting ...because you need energy?

- Yes
- No

Do you eat chocolate cake with frosting ...because you are familiar with it?

- Yes
- No

Do you eat chocolate cake with frosting ...because you like it?

- Yes
- No

Do you eat chocolate cake with frosting ...because it is pleasantly filling?

- Yes
- No

Do you eat chocolate cake with frosting ...in order to reward yourself?

- Yes
- No

Do you eat chocolate cake with frosting ...out of traditions (e.g. family traditions, special occasions)?

- Yes
- No

Do you eat chocolate cake with frosting ...because it belongs to certain situations?

- Yes
- No

Do you eat chocolate cake with frosting ...to maintain a balanced diet?

- Yes
- No

Do you eat chocolate cake with frosting ...because it is social?

- Yes
- No

Do you eat chocolate cake with frosting ...because it is inexpensive?

- Yes
- No

Do you eat chocolate cake with frosting ...because it spontaneously appeals to you (e.g. situated at eye level, appealing colors)?

- Yes
- No

Do you eat chocolate cake with frosting ...to avoid disappointing someone who is trying to make you happy?

- Yes
- No

Do you eat chocolate cake with frosting ...because it is trendy?

- Yes
- No

Do you eat chocolate cake with frosting ...because it is on sale?

- Yes
- No

Do you eat chocolate cake with frosting ...because it keeps you in shape (e.g. energetic, motivated)?

- Yes
- No

Do you eat chocolate cake with frosting ...because that is the only choice?

- Yes
- No

Do you eat chocolate cake with frosting ...because you want to eat it every day?

- Yes
- No

Do you not eat chocolate cake with frosting ...because you don't think it is a snack?

- Yes
- No

Do you not eat chocolate cake with frosting ...because you don't like it?

- Yes
- No

Do you not eat chocolate cake with frosting ...because you don't usually eat it?

- Yes
- No

Do you not eat chocolate cake with frosting ...because the portion size is not suitable?

- Yes
- No

Do you not eat chocolate cake with frosting ...because it is not filling enough?

- Yes
- No

Do you not eat chocolate cake with frosting ...because it is not healthy?

- Yes
- No

Do you not eat chocolate cake with frosting ...because it is not convenient?

- Yes
- No

Do you not eat chocolate cake with frosting ...because you do not want to indulge yourself?

- Yes
- No

Do you not eat chocolate cake with frosting ...because it is not appropriate for the situation?

- Yes
- No

Do you not eat chocolate cake with frosting ...because it is not a food you eat around other people?

- Yes
- No

Do you not eat chocolate cake with frosting ...because the price is too high?

- Yes
- No

Do you not eat chocolate cake with frosting ...because you don't like the way it looks?

- Yes
- No

Do you not eat chocolate cake with frosting ...because it is too high in calories?

- Yes
- No

Do you not eat chocolate cake with frosting ...because you are not supposed to eat it?

- Yes
- No

Do you not eat chocolate cake with frosting ...because eating it makes you seem "behind the times"?

- Yes
- No

Do you not eat chocolate cake with frosting ...because it is not something you have desire to eat at this time?

- Yes
- No

Do you not eat chocolate cake with frosting ...because you had it recently and don't want to eat the same food too often?

- Yes
- No

Do you not eat chocolate cake with frosting ...because you like to eat the same food every day?

- Yes

- No

Do you not eat chocolate cake with frosting ...because this food makes you feel sad, lonely, or frustrated?

- Yes
- No

Do you not eat chocolate cake with frosting ...because it is not organic?

- Yes
- No

Reasons for Starchy Food Choices(RATA)

Do you eat baked potatoes?

- Yes
- No

What are the reasons you eat baked potatoes? (check all that apply)

- ... because it is social
- ... in order to indulge myself
- ... because I am accustomed to eating it
- ... because I have an appetite for it
- ... because it is healthy
- ... because I am hungry
- ... in order to reward myself
- ... because it keeps me in shape (e.g. energetic, motivated)
- ... out of traditions (e.g. family traditions, special occasions)

- [ ] ... because I usually eat it
- [ ] ... because it belongs to certain situations
- [ ] ... because I want to eat it every day
- [ ] ... because I feel sad
- [ ] ... because I grew up with it
- [ ] ... because I am frustrated
- [ ] ... because it is on sale
- [ ] ... because I feel lonely
- [ ] ... because the presentation is appealing (e.g. packaging)
- [ ] ... because I am supposed to eat it
- [ ] ... because it would be impolite not to eat it
- [ ] ... because it is natural (e.g. not genetically modified)
- [ ] ... because it contains no harmful substances (e.g. pesticides, pollutants, antibiotics)
- [ ] ... because it is the most convenient
- [ ] ... because I recognize it from advertisements or have seen it on TV
- [ ] ... because it is inexpensive
- [ ] ... because that is the only choice
- [ ] ... because I watch my weight
- [ ] ... because I am familiar with it
- [ ] ... because it is organic
- [ ] ... so that I can spend time with other people
- [ ] ... because it tastes good
- [ ] ... because it spontaneously appealed to me (e.g. situated at eye level, appealing colors)
- [ ] ... to avoid disappointing someone who is trying to make me happy
- [ ] ... because I like it
- [ ] ... because I enjoy it
- [ ] ... because it is low in fat
- [ ] ... because it is pleasantly filling
- [ ] ... because I need energy
- [ ] ... because others like it
- [ ] ... because it is low in calories

- ☐ ... because it makes me look good in front of others
- ☐ ... because it is easy to prepare
- ☐ ... to maintain a balanced diet
- ☐ ... because I don't want to spend any more money
- ☐ ... because it is quick to prepare
- ☐ ... because it is trendy
- ☐ ... because it makes social gatherings more comfortable

You selected " ... because I am accustomed to eating it" How important is this statement to you?

- ○ Extremely Important
- ○ Very Important
- ○ Moderately Important
- ○ Slightly Important
- ○ Not At All Important

You selected "... because it is healthy"How important is this statement to you?

- ○ Extremely Important
- ○ Very Important
- ○ Moderately Important
- ○ Slightly Important
- ○ Not At All Important

You selected "... because it is quick to prepare"How important is this statement to you?

- ○ Extremely Important
- ○ Very Important
- ○ Moderately Important
- ○ Slightly Important

- Not At All Important

You selected "... because it tastes good"How important is this statement to you?

- Extremely Important
- Very Important
- Moderately Important
- Slightly Important
- Not At All Important

You selected "... in order to indulge myself"How important is this statement to you?

- Extremely Important
- Very Important
- Moderately Important
- Slightly Important
- Not At All Important

You Selected "... because it is natural (e.g. not genetically modified)"How important is this statement to you?

- Extremely Important
- Very Important
- Moderately Important
- Slightly Important
- Not At All Important

You selected "... because I don't want to spend any more money"How important is this statement to you?

- Extremely Important

- Very Important
- Moderately Important
- Slightly Important
- Not At All Important

You selected "... because it is low in calories"How important is this statement to you?

- Extremely Important
- Very Important
- Moderately Important
- Slightly Important
- Not At All Important

You selected "... because I am frustrated"How important is this statement to you?

- Extremely Important
- Very Important
- Moderately Important
- Slightly Important
- Not At All Important

You selected "... because it makes me look good in front of others"How important is this statement to you?

- Extremely Important
- Very Important
- Moderately Important
- Slightly Important
- Not At All Important

You selected "... because it would be impolite not to eat it"How important is this statement to you?

- Extremely Important
- Very Important
- Moderately Important
- Slightly Important
- Not At All Important

You selected "... because the presentation is appealing (e.g. packaging)"How important is this statement to you?

- Extremely Important
- Very Important
- Moderately Important
- Slightly Important
- Not At All Important

You selected "... so that I can spend time with other people"How important is this statement to you?

- Extremely Important
- Very Important
- Moderately Important
- Slightly Important
- Not At All Important

You selected "... because it is organic"How important is this statement to you?

- Extremely Important
- Very Important
- Moderately Important

- Slightly Important
- Not At All Important

You selected "... because I grew up with it"How important is this statement to you?

- Extremely Important
- Very Important
- Moderately Important
- Slightly Important
- Not At All Important

You selected "... because I enjoy it"How important is this statement to you?

- Extremely Important
- Very Important
- Moderately Important
- Slightly Important
- Not At All Important

You selected "... because I am hungry"How important is this statement to you?

- Extremely Important
- Very Important
- Moderately Important
- Slightly Important
- Not At All Important

You selected "... because I usually eat it"How important is this statement to you?

- Extremely Important
- Very Important

- Moderately Important
- Slightly Important
- Not At All Important

You selected "... because I have an appetite for it"How important is this statement to you?

- Extremely Important
- Very Important
- Moderately Important
- Slightly Important
- Not At All Important

You selected "... because it is easy to prepare"How important is this statement to you?

- Extremely Important
- Very Important
- Moderately Important
- Slightly Important
- Not At All Important

You selected "... because I feel sad"How important is this statement to you?

- Extremely Important
- Very Important
- Moderately Important
- Slightly Important
- Not At All Important

You selected "... because it is low in fat"How important is this statement to you?

- Extremely Important
- Very Important
- Moderately Important
- Slightly Important
- Not At All Important

You selected "... because I recognize it from advertisements or have seen it on TV"How important is this statement to you?

- Extremely Important
- Very Important
- Moderately Important
- Slightly Important
- Not At All Important

You selected "... because it makes social gatherings more comfortable"How important is this statement to you?

- Extremely Important
- Very Important
- Moderately Important
- Slightly Important
- Not At All Important

You selected "... because I am supposed to eat it"How important is this statement to you?

- Extremely Important
- Very Important
- Moderately Important
- Slightly Important

- Not At All Important

You selected "... because others like it"How important is this statement to you?

- Extremely Important
- Very Important
- Moderately Important
- Slightly Important
- Not At All Important

You selected "... because I feel lonely"How important is this statement to you?

- Extremely Important
- Very Important
- Moderately Important
- Slightly Important
- Not At All Important

You selected "... because I watch my weightHow important is this statement to you?

- Extremely Important
- Very Important
- Moderately Important
- Slightly Important
- Not At All Important

You selected "... because it contains no harmful substances (e.g. pesticides, pollutants, antibiotics)}"How important is this statement to you?

- Extremely Important
- Very Important

- Moderately Important
- Slightly Important
- Not At All Important

You selected "... because it is the most convenient"How important is this statement to you?

- Extremely Important
- Very Important
- Moderately Important
- Slightly Important
- Not At All Important

You selected "... because I need energy"How important is this statement to you?

- Extremely Important
- Very Important
- Moderately Important
- Slightly Important
- Not At All Important

You selected "... because I am familiar with it"How important is this statement to you?

- Extremely Important
- Very Important
- Moderately Important
- Slightly Important
- Not At All Important

You selected "... because I like it"How important is this statement to you?

- Extremely Important
- Very Important
- Moderately Important
- Slightly Important
- Not At All Important

You selected "... because it is pleasantly filling"How important is this statement to you?

- Extremely Important
- Very Important
- Moderately Important
- Slightly Important
- Not At All Important

You selected "... in order to reward myself"How important is this statement to you?

- Extremely Important
- Very Important
- Moderately Important
- Slightly Important
- Not At All Important

You selected "... out of traditions (e.g. family traditions, special occasions)"How important is this statement to you?

- Extremely Important
- Very Important
- Moderately Important
- Slightly Important
- Not At All Important

You selected "... because it belongs to certain situations"How important is this statement to you?

- Extremely Important
- Very Important
- Moderately Important
- Slightly Important
- Not At All Important

You selected "... to maintain a balanced diet"How important is this statement to you?

- Extremely Important
- Very Important
- Moderately Important
- Slightly Important
- Not At All Important

You selected "... because it is social"How important is this statement to you?

- Extremely Important
- Very Important
- Moderately Important
- Slightly Important
- Not At All Important

You selected "... because it is inexpensive"How important is this statement to you?

- Extremely Important
- Very Important
- Moderately Important

- Slightly Important
- Not At All Important

You selected "... because it spontaneously appealed to me (e.g. situated at eye level, appealing colors)"How important is this statement to you?

- Extremely Important
- Very Important
- Moderately Important
- Slightly Important
- Not At All Important

You selected "... to avoid disappointing someone who is trying to make me happy"How important is this statement to you?

- Extremely Important
- Very Important
- Moderately Important
- Slightly Important
- Not At All Important

You selected "... because it is trendy"How important is this statement to you?

- Extremely Important
- Very Important
- Moderately Important
- Slightly Important
- Not At All Important

You selected "... because it is on sale"How important is this statement to you?

- Extremely Important
- Very Important
- Moderately Important
- Slightly Important
- Not At All Important

You selected ... because it keeps me in shape (e.g. energetic, motivated)How important is this statement to you?

- Extremely Important
- Very Important
- Moderately Important
- Slightly Important
- Not At All Important

?You selected "... because that is the only choice"How important is this statement to you

- Extremely Important
- Very Important
- Moderately Important
- Slightly Important
- Not At All Important

You selected "... because I want to eat it every day"How important is this statement to you?

- Extremely Important
- Very Important
- Moderately Important
- Slightly Important
- Not At All Important

Please tell us all the reasons you did not eat baked potatoes (check all that apply)

- [ ] I don't like the way it looks
- [ ] it is not organic
- [ ] I would never choose this because I like to eat the same food every day.
- [ ] it is not healthy
- [ ] it is not not something I have desire to eat at this time
- [ ] I do not want to indulge myself
- [ ] it is not filling enough
- [ ] I don't like it
- [ ] I had it recently and I don't want to eat the same food too often
- [ ] it is too high in calories
- [ ] it is not a food I eat around other people
- [ ] it is not convenient
- [ ] the price is too high
- [ ] it is not appropriate for the situation
- [ ] I don't usually eat it
- [ ] I don't think it is a snack
- [ ] I am not supposed to eat it
- [ ] eating it makes me seem "behind the times"
- [ ] this food makes me feel sad, lonely, or frustrated
- [ ] the portion size was not suitable

You selected "I don't think it is a snack"How important is this statement to you?

- ○ Extremely Important
- ○ Very Important
- ○ Moderately Important
- ○ Slightly Important
- ○ Not At All Important

You selected "I don't like it"How important is this statement to you?

- Extremely Important
- Very Important
- Moderately Important
- Slightly Important
- Not At All Important

You selected "I don't usually eat it"How important is this statement to you?

- Extremely Important
- Very Important
- Moderately Important
- Slightly Important
- Not At All Important

You selected "the portion size was not suitable"How important is this statement to you?

- Extremely Important
- Very Important
- Moderately Important
- Slightly Important
- Not At All Important

You selected "it is not filling enough"How important is this statement to you?

- Extremely Important
- Very Important
- Moderately Important
- Slightly Important
- Not At All Important

You selected "it is not healthy"How important is this statement to you?

- Extremely Important
- Very Important
- Moderately Important
- Slightly Important
- Not At All Important

You selected "it is not convenient"How important is this statement to you?

- Extremely Important
- Very Important
- Moderately Important
- Slightly Important
- Not At All Important

You selected "I do not want to indulge myself"How important is this statement to you?

- Extremely Important
- Very Important
- Moderately Important
- Slightly Important
- Not At All Important

You selected "it is not appropriate for the situation"How important is this statement to you?

- Extremely Important
- Very Important

- Moderately Important
- Slightly Important
- Not At All Important

You selected "it is not a food I eat around other people"How important is this statement to you?

- Extremely Important
- Very Important
- Moderately Important
- Slightly Important
- Not At All Important

You selected "the price is too high"How important is this statement to you?

- Extremely Important
- Very Important
- Moderately Important
- Slightly Important
- Not At All Important

You selected "I don't like the way it looks"How important is this statement to you?

- Extremely Important
- Very Important
- Moderately Important
- Slightly Important
- Not At All Important

You selected "it is too high in calories"How important is this statement to you?

- Extremely Important
- Very Important
- Moderately Important
- Slightly Important
- Not At All Important

You selected "I am not supposed to eat it"How important is this statement to you?

- Extremely Important
- Very Important
- Moderately Important
- Slightly Important
- Not At All Important

You selected "eating it makes me seem "behind the times""How important is this statement to you?

- Extremely Important
- Very Important
- Moderately Important
- Slightly Important
- Not At All Important

You selected "it is not not something I have desire to eat at this time"How important is this statement to you?

- Extremely Important
- Very Important
- Moderately Important
- Slightly Important
- Not At All Important

You selected "I had it recently and I don't want to eat the same food too often"How important is this statement to you?

- Extremely Important
- Very Important
- Moderately Important
- Slightly Important
- Not At All Important

You selected "I would never choose this because I like to eat the same food every day."How important is this statement to you?

- Extremely Important
- Very Important
- Moderately Important
- Slightly Important
- Not At All Important

You selected "this food makes me feel sad, lonely, or frustrated"How important is this statement to you?

- Extremely Important
- Very Important
- Moderately Important
- Slightly Important
- Not At All Important

You selected "it is not organic"How important is this statement to you?

- Extremely Important
- Very Important

- Moderately Important
- Slightly Important
- Not At All Important

Reasons for Protein foods Choices(RATA)

Do you eat hamburgers?

- Yes
- No

What are the reasons you eat hamburgers? (check all that apply)

- ... because the presentation is appealing (e.g. packaging)
- ... because I feel lonely
- ... because it keeps me in shape (e.g. energetic, motivated)
- ... in order to reward myself
- ... because it tastes good
- ... because I have an appetite for it
- ... because I am frustrated
- ... because it is inexpensive
- ... because I am supposed to eat it
- ... to avoid disappointing someone who is trying to make me happy
- ... because it is organic
- ... because it is the most convenient
- ... because that is the only choice
- ... to maintain a balanced diet
- ... because I don't want to spend any more money
- ... because I am familiar with it
- ... because it is social
- ... because I like it

- ☐ ... because it is pleasantly filling
- ☐ ... because it is healthy
- ☐ ... because it would be impolite not to eat it
- ☐ ... because it makes social gatherings more comfortable
- ☐ ... because I am hungry
- ☐ ... because I enjoy it
- ☐ ... because it is natural (e.g. not genetically modified)
- ☐ ... because I want to eat it every day
- ☐ ... because I need energy
- ☐ ... because it contains no harmful substances (e.g. pesticides, pollutants, antibiotics)
- ☐ ... because I recognize it from advertisements or have seen it on TV
- ☐ ... because it is quick to prepare
- ☐ ... because it is easy to prepare
- ☐ ... because it makes me look good in front of others
- ☐ ... because it belongs to certain situations
- ☐ ... because others like it
- ☐ ... because I feel sad
- ☐ ... because I usually eat it
- ☐ ... because it is low in calories
- ☐ ... because it is trendy
- ☐ ... out of traditions (e.g. family traditions, special occasions)
- ☐ ... because it spontaneously appealed to me (e.g. situated at eye level, appealing colors)
- ☐ ... because I watch my weight
- ☐ ... because I grew up with it
- ☐ ... because it is low in fat
- ☐ ... in order to indulge myself
- ☐ ... so that I can spend time with other people
- ☐ ... because I am accustomed to eating it
- ☐ ... because it is on sale

You selected " ... because I am accustomed to eating it" How important is this statement to you?

- Extremely Important
- Very Important
- Moderately Important
- Slightly Important
- Not At All Important

You selected "... because it is healthy"How important is this statement to you?

- Extremely Important
- Very Important
- Moderately Important
- Slightly Important
- Not At All Important

You selected "... because it is quick to prepare"How important is this statement to you?

- Extremely Important
- Very Important
- Moderately Important
- Slightly Important
- Not At All Important

You selected "... because it tastes good"How important is this statement to you?

- Extremely Important
- Very Important
- Moderately Important
- Slightly Important

- Not At All Important

You selected "... in order to indulge myself"How important is this statement to you?

- Extremely Important
- Very Important
- Moderately Important
- Slightly Important
- Not At All Important

You Selected "... because it is natural (e.g. not genetically modified)"How important is this statement to you?

- Extremely Important
- Very Important
- Moderately Important
- Slightly Important
- Not At All Important

You selected "... because I don't want to spend any more money"How important is this statement to you?

- Extremely Important
- Very Important
- Moderately Important
- Slightly Important
- Not At All Important

You selected "... because it is low in calories"How important is this statement to you?

- Extremely Important
- Very Important
- Moderately Important
- Slightly Important
- Not At All Important

You selected "... because I am frustrated"How important is this statement to you?

- Extremely Important
- Very Important
- Moderately Important
- Slightly Important
- Not At All Important

You selected "... because it makes me look good in front of others"How important is this statement to you?

- Extremely Important
- Very Important
- Moderately Important
- Slightly Important
- Not At All Important

You selected "... because it would be impolite not to eat it"How important is this statement to you?

- Extremely Important
- Very Important
- Moderately Important
- Slightly Important
- Not At All Important

You selected "... because the presentation is appealing (e.g. packaging)"How important is this statement to you?

- Extremely Important
- Very Important
- Moderately Important
- Slightly Important
- Not At All Important

You selected "... so that I can spend time with other people"How important is this statement to you?

- Extremely Important
- Very Important
- Moderately Important
- Slightly Important
- Not At All Important

You selected "... because it is organic"How important is this statement to you?

- Extremely Important
- Very Important
- Moderately Important
- Slightly Important
- Not At All Important

You selected "... because I grew up with it"How important is this statement to you?

- Extremely Important
- Very Important
- Moderately Important

- Slightly Important
- Not At All Important

You selected "... because I enjoy it"How important is this statement to you?

- Extremely Important
- Very Important
- Moderately Important
- Slightly Important
- Not At All Important

You selected "... because I am hungry"How important is this statement to you?

- Extremely Important
- Very Important
- Moderately Important
- Slightly Important
- Not At All Important

You selected "... because I usually eat it"How important is this statement to you?

- Extremely Important
- Very Important
- Moderately Important
- Slightly Important
- Not At All Important

You selected "... because I have an appetite for it"How important is this statement to you?

- Extremely Important

- Very Important
- Moderately Important
- Slightly Important
- Not At All Important

You selected "... because it is easy to prepare"How important is this statement to you?

- Extremely Important
- Very Important
- Moderately Important
- Slightly Important
- Not At All Important

You selected "... because I feel sad"How important is this statement to you?

- Extremely Important
- Very Important
- Moderately Important
- Slightly Important
- Not At All Important

You selected "... because it is low in fat"How important is this statement to you?

- Extremely Important
- Very Important
- Moderately Important
- Slightly Important
- Not At All Important

You selected "... because I recognize it from advertisements or have seen it on TV"How important is this statement to you?

- Extremely Important
- Very Important
- Moderately Important
- Slightly Important
- Not At All Important

You selected "... because it makes social gatherings more comfortable"How important is this statement to you?

- Extremely Important
- Very Important
- Moderately Important
- Slightly Important
- Not At All Important

You selected "... because I am supposed to eat it"How important is this statement to you?

- Extremely Important
- Very Important
- Moderately Important
- Slightly Important
- Not At All Important

You selected "... because others like it"How important is this statement to you?

- Extremely Important
- Very Important
- Moderately Important

- Slightly Important
- Not At All Important

You selected "... because I feel lonely"How important is this statement to you?

- Extremely Important
- Very Important
- Moderately Important
- Slightly Important
- Not At All Important

You selected "... because I watch my weightHow important is this statement to you?

- Extremely Important
- Very Important
- Moderately Important
- Slightly Important
- Not At All Important

You selected "... because it contains no harmful substances (e.g. pesticides, pollutants, antibiotics)}"How important is this statement to you?

- Extremely Important
- Very Important
- Moderately Important
- Slightly Important
- Not At All Important

You selected "... because it is the most convenient"How important is this statement to you?

- Extremely Important
- Very Important
- Moderately Important
- Slightly Important
- Not At All Important

You selected "... because I need energy"How important is this statement to you?

- Extremely Important
- Very Important
- Moderately Important
- Slightly Important
- Not At All Important

You selected "... because I am familiar with it"How important is this statement to you?

- Extremely Important
- Very Important
- Moderately Important
- Slightly Important
- Not At All Important

You selected "... because I like it"How important is this statement to you?

- Extremely Important
- Very Important
- Moderately Important
- Slightly Important
- Not At All Important

You selected "... because it is pleasantly filling"How important is this statement to you?

- Extremely Important
- Very Important
- Moderately Important
- Slightly Important
- Not At All Important

You selected "... in order to reward myself"How important is this statement to you?

- Extremely Important
- Very Important
- Moderately Important
- Slightly Important
- Not At All Important

You selected "... out of traditions (e.g. family traditions, special occasions)"How important is this statement to you?

- Extremely Important
- Very Important
- Moderately Important
- Slightly Important
- Not At All Important

You selected "... because it belongs to certain situations"How important is this statement to you?

- Extremely Important
- Very Important
- Moderately Important

- Slightly Important
- Not At All Important

You selected "... to maintain a balanced diet"How important is this statement to you?

- Extremely Important
- Very Important
- Moderately Important
- Slightly Important
- Not At All Important

You selected "... because it is social"How important is this statement to you?

- Extremely Important
- Very Important
- Moderately Important
- Slightly Important
- Not At All Important

You selected "... because it is inexpensive"How important is this statement to you?

- Extremely Important
- Very Important
- Moderately Important
- Slightly Important
- Not At All Important

You selected "... because it spontaneously appealed to me (e.g. situated at eye level, appealing colors)"How important is this statement to you?

- Extremely Important
- Very Important
- Moderately Important
- Slightly Important
- Not At All Important

You selected "... to avoid disappointing someone who is trying to make me happy"How important is this statement to you?

- Extremely Important
- Very Important
- Moderately Important
- Slightly Important
- Not At All Important

You selected "... because it is trendy"How important is this statement to you?

- Extremely Important
- Very Important
- Moderately Important
- Slightly Important
- Not At All Important

You selected "... because it is on sale"How important is this statement to you?

- Extremely Important
- Very Important
- Moderately Important
- Slightly Important
- Not At All Important

You selected ... because it keeps me in shape (e.g. energetic, motivated)How important is this statement to you?

- Extremely Important
- Very Important
- Moderately Important
- Slightly Important
- Not At All Important

?You selected "... because that is the only choice"How important is this statement to you

- Extremely Important
- Very Important
- Moderately Important
- Slightly Important
- Not At All Important

You selected "... because I want to eat it every day"How important is this statement to you?

- Extremely Important
- Very Important
- Moderately Important
- Slightly Important
- Not At All Important

Please tell us all the reasons you did not eat hamburgers (check all that apply)

- [ ] it is not convenient
- [ ] eating it makes me seem "behind the times"
- [ ] it is not appropriate for the situation

- [ ] it is too high in calories
- [ ] it is not healthy
- [ ] I had it recently and I don't want to eat the same food too often
- [ ] I don't think it is a snack
- [ ] the portion size was not suitable
- [ ] this food makes me feel sad, lonely, or frustrated
- [ ] I don't like the way it looks
- [ ] I would never choose this because I like to eat the same food every day.
- [ ] I do not want to indulge myself
- [ ] it is not filling enough
- [ ] I don't usually eat it
- [ ] the price is too high
- [ ] it is not organic
- [ ] it is not not something I have desire to eat at this time
- [ ] I don't like it
- [ ] I am not supposed to eat it
- [ ] it is not a food I eat around other people

You selected "I don't think it is a snack"How important is this statement to you?

- Extremely Important
- Very Important
- Moderately Important
- Slightly Important
- Not At All Important

You selected "I don't like it"How important is this statement to you?

- Extremely Important
- Very Important
- Moderately Important
- Slightly Important

- Not At All Important

You selected "I don't usually eat it"How important is this statement to you?

- Extremely Important
- Very Important
- Moderately Important
- Slightly Important
- Not At All Important

You selected "the portion size was not suitable"How important is this statement to you?

- Extremely Important
- Very Important
- Moderately Important
- Slightly Important
- Not At All Important

You selected "it is not filling enough"How important is this statement to you?

- Extremely Important
- Very Important
- Moderately Important
- Slightly Important
- Not At All Important

You selected "it is not healthy"How important is this statement to you?

- Extremely Important
- Very Important

- Moderately Important
- Slightly Important
- Not At All Important

You selected "it is not convenient"How important is this statement to you?

- Extremely Important
- Very Important
- Moderately Important
- Slightly Important
- Not At All Important

You selected "I do not want to indulge myself"How important is this statement to you?

- Extremely Important
- Very Important
- Moderately Important
- Slightly Important
- Not At All Important

You selected "it is not appropriate for the situation"How important is this statement to you?

- Extremely Important
- Very Important
- Moderately Important
- Slightly Important
- Not At All Important

You selected "it is not a food I eat around other people"How important is this statement to you?

- Extremely Important
- Very Important
- Moderately Important
- Slightly Important
- Not At All Important

You selected "the price is too high"How important is this statement to you?

- Extremely Important
- Very Important
- Moderately Important
- Slightly Important
- Not At All Important

You selected "I don't like the way it looks"How important is this statement to you?

- Extremely Important
- Very Important
- Moderately Important
- Slightly Important
- Not At All Important

You selected "it is too high in calories"How important is this statement to you?

- Extremely Important
- Very Important
- Moderately Important
- Slightly Important
- Not At All Important

You selected "I am not supposed to eat it"How important is this statement to you?

- Extremely Important
- Very Important
- Moderately Important
- Slightly Important
- Not At All Important

You selected "eating it makes me seem "behind the times""How important is this statement to you?

- Extremely Important
- Very Important
- Moderately Important
- Slightly Important
- Not At All Important

You selected "it is not not something I have desire to eat at this time"How important is this statement to you?

- Extremely Important
- Very Important
- Moderately Important
- Slightly Important
- Not At All Important

You selected "I had it recently and I don't want to eat the same food too often"How important is this statement to you?

- Extremely Important

- Very Important
- Moderately Important
- Slightly Important
- Not At All Important

You selected "I would never choose this because I like to eat the same food every day."How important is this statement to you?

- Extremely Important
- Very Important
- Moderately Important
- Slightly Important
- Not At All Important

You selected "this food makes me feel sad, lonely, or frustrated"How important is this statement to you?

- Extremely Important
- Very Important
- Moderately Important
- Slightly Important
- Not At All Important

You selected "it is not organic"How important is this statement to you?

- Extremely Important
- Very Important
- Moderately Important
- Slightly Important
- Not At All Important

Reasons for Dairy Food Choices(RATA)

Do you eat american cheese?

- Yes
- No

What are the reasons you eat american cheese? (check all that apply)

- ... because I feel sad
- ... because it makes social gatherings more comfortable
- ... because I recognize it from advertisements or have seen it on TV
- ... to maintain a balanced diet
- ... out of traditions (e.g. family traditions, special occasions)
- ... because I don't want to spend any more money
- ... because I watch my weight
- ... because it belongs to certain situations
- ... because others like it
- ... because I feel lonely
- ... in order to indulge myself
- ... because I need energy
- ... in order to reward myself
- ... because I usually eat it
- ... so that I can spend time with other people
- ... because it spontaneously appealed to me (e.g. situated at eye level, appealing colors)
- ... because it makes me look good in front of others
- ... because it is low in fat
- ... because I grew up with it
- ... because it is trendy
- ... because I am familiar with it
- ... because it contains no harmful substances (e.g. pesticides, pollutants, antibiotics)

- ☐ ... because I am frustrated
- ☐ ... because it is organic
- ☐ ... because I am supposed to eat it
- ☐ ... to avoid disappointing someone who is trying to make me happy
- ☐ ... because I enjoy it
- ☐ ... because it is low in calories
- ☐ ... because the presentation is appealing (e.g. packaging)
- ☐ ... because I have an appetite for it
- ☐ ... because it is pleasantly filling
- ☐ ... because it is healthy
- ☐ ... because I am hungry
- ☐ ... because I like it
- ☐ ... because I am accustomed to eating it
- ☐ ... because it would be impolite not to eat it
- ☐ ... because it is the most convenient
- ☐ ... because that is the only choice
- ☐ ... because it is quick to prepare
- ☐ ... because I want to eat it every day
- ☐ ... because it is natural (e.g. not genetically modified)
- ☐ ... because it is social
- ☐ ... because it tastes good
- ☐ ... because it is inexpensive
- ☐ ... because it is on sale
- ☐ ... because it is easy to prepare
- ☐ ... because it keeps me in shape (e.g. energetic, motivated)

You selected " ... because I am accustomed to eating it" How important is this statement to you?

- ○ Extremely Important
- ○ Very Important
- ○ Moderately Important

- Slightly Important
- Not At All Important

You selected "... because it is healthy"How important is this statement to you?

- Extremely Important
- Very Important
- Moderately Important
- Slightly Important
- Not At All Important

You selected "... because it is quick to prepare"How important is this statement to you?

- Extremely Important
- Very Important
- Moderately Important
- Slightly Important
- Not At All Important

You selected "... because it tastes good"How important is this statement to you?

- Extremely Important
- Very Important
- Moderately Important
- Slightly Important
- Not At All Important

You selected "... in order to indulge myself"How important is this statement to you?

- Extremely Important

- Very Important
- Moderately Important
- Slightly Important
- Not At All Important

You Selected "... because it is natural (e.g. not genetically modified)"How important is this statement to you?

- Extremely Important
- Very Important
- Moderately Important
- Slightly Important
- Not At All Important

You selected "... because I don't want to spend any more money"How important is this statement to you?

- Extremely Important
- Very Important
- Moderately Important
- Slightly Important
- Not At All Important

You selected "... because it is low in calories"How important is this statement to you?

- Extremely Important
- Very Important
- Moderately Important
- Slightly Important
- Not At All Important

You selected "... because I am frustrated"How important is this statement to you?

- Extremely Important
- Very Important
- Moderately Important
- Slightly Important
- Not At All Important

You selected "... because it makes me look good in front of others"How important is this statement to you?

- Extremely Important
- Very Important
- Moderately Important
- Slightly Important
- Not At All Important

You selected "... because it would be impolite not to eat it"How important is this statement to you?

- Extremely Important
- Very Important
- Moderately Important
- Slightly Important
- Not At All Important

You selected "... because the presentation is appealing (e.g. packaging)"How important is this statement to you?

- Extremely Important
- Very Important

- Moderately Important
- Slightly Important
- Not At All Important

You selected "... so that I can spend time with other people"How important is this statement to you?

- Extremely Important
- Very Important
- Moderately Important
- Slightly Important
- Not At All Important

You selected "... because it is organic"How important is this statement to you?

- Extremely Important
- Very Important
- Moderately Important
- Slightly Important
- Not At All Important

You selected "... because I grew up with it"How important is this statement to you?

- Extremely Important
- Very Important
- Moderately Important
- Slightly Important
- Not At All Important

You selected "... because I enjoy it"How important is this statement to you?

- Extremely Important
- Very Important
- Moderately Important
- Slightly Important
- Not At All Important

You selected "... because I am hungry"How important is this statement to you?

- Extremely Important
- Very Important
- Moderately Important
- Slightly Important
- Not At All Important

You selected "... because I usually eat it"How important is this statement to you?

- Extremely Important
- Very Important
- Moderately Important
- Slightly Important
- Not At All Important

You selected "... because I have an appetite for it"How important is this statement to you?

- Extremely Important
- Very Important
- Moderately Important
- Slightly Important
- Not At All Important

You selected "... because it is easy to prepare"How important is this statement to you?

- Extremely Important
- Very Important
- Moderately Important
- Slightly Important
- Not At All Important

You selected "... because I feel sad"How important is this statement to you?

- Extremely Important
- Very Important
- Moderately Important
- Slightly Important
- Not At All Important

You selected "... because it is low in fat"How important is this statement to you?

- Extremely Important
- Very Important
- Moderately Important
- Slightly Important
- Not At All Important

You selected "... because I recognize it from advertisements or have seen it on TV"How important is this statement to you?

- Extremely Important
- Very Important
- Moderately Important
- Slightly Important

- Not At All Important

You selected "... because it makes social gatherings more comfortable"How important is this statement to you?

- Extremely Important
- Very Important
- Moderately Important
- Slightly Important
- Not At All Important

You selected "... because I am supposed to eat it"How important is this statement to you?

- Extremely Important
- Very Important
- Moderately Important
- Slightly Important
- Not At All Important

You selected "... because others like it"How important is this statement to you?

- Extremely Important
- Very Important
- Moderately Important
- Slightly Important
- Not At All Important

You selected "... because I feel lonely"How important is this statement to you?

- Extremely Important

- Very Important
- Moderately Important
- Slightly Important
- Not At All Important

You selected "... because I watch my weightHow important is this statement to you?

- Extremely Important
- Very Important
- Moderately Important
- Slightly Important
- Not At All Important

You selected "... because it contains no harmful substances (e.g. pesticides, pollutants, antibiotics)}"How important is this statement to you?

- Extremely Important
- Very Important
- Moderately Important
- Slightly Important
- Not At All Important

You selected "... because it is the most convenient"How important is this statement to you?

- Extremely Important
- Very Important
- Moderately Important
- Slightly Important
- Not At All Important

You selected "... because I need energy"How important is this statement to you?

- Extremely Important
- Very Important
- Moderately Important
- Slightly Important
- Not At All Important

You selected "... because I am familiar with it"How important is this statement to you?

- Extremely Important
- Very Important
- Moderately Important
- Slightly Important
- Not At All Important

You selected "... because I like it"How important is this statement to you?

- Extremely Important
- Very Important
- Moderately Important
- Slightly Important
- Not At All Important

You selected "... because it is pleasantly filling"How important is this statement to you?

- Extremely Important
- Very Important
- Moderately Important
- Slightly Important

- Not At All Important

You selected "... in order to reward myself"How important is this statement to you?

- Extremely Important
- Very Important
- Moderately Important
- Slightly Important
- Not At All Important

You selected "... out of traditions (e.g. family traditions, special occasions)"How important is this statement to you?

- Extremely Important
- Very Important
- Moderately Important
- Slightly Important
- Not At All Important

You selected "... because it belongs to certain situations"How important is this statement to you?

- Extremely Important
- Very Important
- Moderately Important
- Slightly Important
- Not At All Important

You selected "... to maintain a balanced diet"How important is this statement to you?

- Extremely Important
- Very Important
- Moderately Important
- Slightly Important
- Not At All Important

You selected "... because it is social"How important is this statement to you?

- Extremely Important
- Very Important
- Moderately Important
- Slightly Important
- Not At All Important

You selected "... because it is inexpensive"How important is this statement to you?

- Extremely Important
- Very Important
- Moderately Important
- Slightly Important
- Not At All Important

You selected "... because it spontaneously appealed to me (e.g. situated at eye level, appealing colors)"How important is this statement to you?

- Extremely Important
- Very Important
- Moderately Important
- Slightly Important
- Not At All Important

You selected "... to avoid disappointing someone who is trying to make me happy"How important is this statement to you?

- Extremely Important
- Very Important
- Moderately Important
- Slightly Important
- Not At All Important

You selected "... because it is trendy"How important is this statement to you?

- Extremely Important
- Very Important
- Moderately Important
- Slightly Important
- Not At All Important

You selected "... because it is on sale"How important is this statement to you?

- Extremely Important
- Very Important
- Moderately Important
- Slightly Important
- Not At All Important

You selected ... because it keeps me in shape (e.g. energetic, motivated)How important is this statement to you?

- Extremely Important
- Very Important
- Moderately Important
- Slightly Important

- Not At All Important

?You selected "... because that is the only choice"How important is this statement to you

- Extremely Important
- Very Important
- Moderately Important
- Slightly Important
- Not At All Important

You selected "... because I want to eat it every day"How important is this statement to you?

- Extremely Important
- Very Important
- Moderately Important
- Slightly Important
- Not At All Important

Please tell us all the reasons you did not eat american cheese (check all that apply)

- [ ] I don't usually eat it
- [ ] I don't think it is a snack
- [ ] it is not healthy
- [ ] I don't like it
- [ ] it is not appropriate for the situation
- [ ] I am not supposed to eat it
- [ ] the portion size was not suitable
- [ ] eating it makes me seem "behind the times"
- [ ] it is not organic

- ☐ it is not not something I have desire to eat at this time
- ☐ the price is too high
- ☐ I would never choose this because I like to eat the same food every day.
- ☐ this food makes me feel sad, lonely, or frustrated
- ☐ it is not convenient
- ☐ it is not a food I eat around other people
- ☐ I don't like the way it looks
- ☐ I do not want to indulge myself
- ☐ it is not filling enough
- ☐ I had it recently and I don't want to eat the same food too often
- ☐ it is too high in calories

You selected "I don't think it is a snack"How important is this statement to you?

- ○ Extremely Important
- ○ Very Important
- ○ Moderately Important
- ○ Slightly Important
- ○ Not At All Important

You selected "I don't like it"How important is this statement to you?

- ○ Extremely Important
- ○ Very Important
- ○ Moderately Important
- ○ Slightly Important
- ○ Not At All Important

You selected "I don't usually eat it"How important is this statement to you?

- ○ Extremely Important
- ○ Very Important

- Moderately Important
- Slightly Important
- Not At All Important

You selected "the portion size was not suitable"How important is this statement to you?

- Extremely Important
- Very Important
- Moderately Important
- Slightly Important
- Not At All Important

You selected "it is not filling enough"How important is this statement to you?

- Extremely Important
- Very Important
- Moderately Important
- Slightly Important
- Not At All Important

You selected "it is not healthy"How important is this statement to you?

- Extremely Important
- Very Important
- Moderately Important
- Slightly Important
- Not At All Important

You selected "it is not convenient"How important is this statement to you?

- Extremely Important
- Very Important
- Moderately Important
- Slightly Important
- Not At All Important

You selected "I do not want to indulge myself"How important is this statement to you?

- Extremely Important
- Very Important
- Moderately Important
- Slightly Important
- Not At All Important

You selected "it is not appropriate for the situation"How important is this statement to you?

- Extremely Important
- Very Important
- Moderately Important
- Slightly Important
- Not At All Important

You selected "it is not a food I eat around other people"How important is this statement to you?

- Extremely Important
- Very Important
- Moderately Important
- Slightly Important
- Not At All Important

You selected "the price is too high"How important is this statement to you?

- Extremely Important
- Very Important
- Moderately Important
- Slightly Important
- Not At All Important

You selected "I don't like the way it looks"How important is this statement to you?

- Extremely Important
- Very Important
- Moderately Important
- Slightly Important
- Not At All Important

You selected "it is too high in calories"How important is this statement to you?

- Extremely Important
- Very Important
- Moderately Important
- Slightly Important
- Not At All Important

You selected "I am not supposed to eat it"How important is this statement to you?

- Extremely Important
- Very Important
- Moderately Important
- Slightly Important

- Not At All Important

You selected "eating it makes me seem "behind the times""How important is this statement to you?

- Extremely Important
- Very Important
- Moderately Important
- Slightly Important
- Not At All Important

You selected "it is not not something I have desire to eat at this time"How important is this statement to you?

- Extremely Important
- Very Important
- Moderately Important
- Slightly Important
- Not At All Important

You selected "I had it recently and I don't want to eat the same food too often"How important is this statement to you?

- Extremely Important
- Very Important
- Moderately Important
- Slightly Important
- Not At All Important

You selected "I would never choose this because I like to eat the same food every day."How important is this statement to you?

- Extremely Important
- Very Important
- Moderately Important
- Slightly Important
- Not At All Important

You selected "this food makes me feel sad, lonely, or frustrated"How important is this statement to you?

- Extremely Important
- Very Important
- Moderately Important
- Slightly Important
- Not At All Important

You selected "it is not organic"How important is this statement to you?

- Extremely Important
- Very Important
- Moderately Important
- Slightly Important
- Not At All Important

Reasons for Fruit Choices(RATA)

Do you eat bananas?

- Yes
- No

What are the reasons you eat bananas? (check all that apply)

- ☐ ... because I usually eat it
- ☐ ... out of traditions (e.g. family traditions, special occasions)
- ☐ ... because it contains no harmful substances (e.g. pesticides, pollutants, antibiotics)
- ☐ ... because I want to eat it every day
- ☐ ... because I don't want to spend any more money
- ☐ ... because it is healthy
- ☐ ... because it is quick to prepare
- ☐ ... because I am accustomed to eating it
- ☐ ... because others like it
- ☐ ... because it is low in fat
- ☐ ... because it is on sale
- ☐ ... because I feel lonely
- ☐ ... because I like it
- ☐ ... because I grew up with it
- ☐ ... because that is the only choice
- ☐ ... because I am supposed to eat it
- ☐ ... because it is low in calories
- ☐ ... because I am hungry
- ☐ ... because I need energy
- ☐ ... because it is the most convenient
- ☐ ... because I am frustrated
- ☐ ... because I feel sad
- ☐ ... because it spontaneously appealed to me (e.g. situated at eye level, appealing colors)
- ☐ ... because it belongs to certain situations
- ☐ ... because I watch my weight
- ☐ ... because it is easy to prepare
- ☐ ... because it makes social gatherings more comfortable
- ☐ ... because it is social
- ☐ ... because it is organic

- ☐ ... because it keeps me in shape (e.g. energetic, motivated)
- ☐ ... because it tastes good
- ☐ ... because it is trendy
- ☐ ... because it makes me look good in front of others
- ☐ ... because I enjoy it
- ☐ ... to maintain a balanced diet
- ☐ ... because I have an appetite for it
- ☐ ... because the presentation is appealing (e.g. packaging)
- ☐ ... so that I can spend time with other people
- ☐ ... because I recognize it from advertisements or have seen it on TV
- ☐ ... in order to reward myself
- ☐ ... in order to indulge myself
- ☐ ... because it is inexpensive
- ☐ ... because it is pleasantly filling
- ☐ ... to avoid disappointing someone who is trying to make me happy
- ☐ ... because it is natural (e.g. not genetically modified)
- ☐ ... because it would be impolite not to eat it
- ☐ ... because I am familiar with it

You selected " ... because I am accustomed to eating it" How important is this statement to you?

- ○ Extremely Important
- ○ Very Important
- ○ Moderately Important
- ○ Slightly Important
- ○ Not At All Important

You selected "... because it is healthy"How important is this statement to you?

- ○ Extremely Important
- ○ Very Important

- Moderately Important
- Slightly Important
- Not At All Important

You selected "... because it is quick to prepare"How important is this statement to you?

- Extremely Important
- Very Important
- Moderately Important
- Slightly Important
- Not At All Important

You selected "... because it tastes good"How important is this statement to you?

- Extremely Important
- Very Important
- Moderately Important
- Slightly Important
- Not At All Important

You selected "... in order to indulge myself"How important is this statement to you?

- Extremely Important
- Very Important
- Moderately Important
- Slightly Important
- Not At All Important

You Selected "... because it is natural (e.g. not genetically modified)"How important is this statement to you?

- Extremely Important
- Very Important
- Moderately Important
- Slightly Important
- Not At All Important

You selected "... because I don't want to spend any more money"How important is this statement to you?

- Extremely Important
- Very Important
- Moderately Important
- Slightly Important
- Not At All Important

You selected "... because it is low in calories"How important is this statement to you?

- Extremely Important
- Very Important
- Moderately Important
- Slightly Important
- Not At All Important

You selected "... because I am frustrated"How important is this statement to you?

- Extremely Important
- Very Important
- Moderately Important
- Slightly Important
- Not At All Important

You selected "... because it makes me look good in front of others"How important is this statement to you?

- Extremely Important
- Very Important
- Moderately Important
- Slightly Important
- Not At All Important

You selected "... because it would be impolite not to eat it"How important is this statement to you?

- Extremely Important
- Very Important
- Moderately Important
- Slightly Important
- Not At All Important

You selected "... because the presentation is appealing (e.g. packaging)"How important is this statement to you?

- Extremely Important
- Very Important
- Moderately Important
- Slightly Important
- Not At All Important

You selected "... so that I can spend time with other people"How important is this statement to you?

- Extremely Important

- Very Important
- Moderately Important
- Slightly Important
- Not At All Important

You selected "... because it is organic"How important is this statement to you?

- Extremely Important
- Very Important
- Moderately Important
- Slightly Important
- Not At All Important

You selected "... because I grew up with it"How important is this statement to you?

- Extremely Important
- Very Important
- Moderately Important
- Slightly Important
- Not At All Important

You selected "... because I enjoy it"How important is this statement to you?

- Extremely Important
- Very Important
- Moderately Important
- Slightly Important
- Not At All Important

You selected "... because I am hungry"How important is this statement to you?

- Extremely Important
- Very Important
- Moderately Important
- Slightly Important
- Not At All Important

You selected "... because I usually eat it"How important is this statement to you?

- Extremely Important
- Very Important
- Moderately Important
- Slightly Important
- Not At All Important

You selected "... because I have an appetite for it"How important is this statement to you?

- Extremely Important
- Very Important
- Moderately Important
- Slightly Important
- Not At All Important

You selected "... because it is easy to prepare"How important is this statement to you?

- Extremely Important
- Very Important
- Moderately Important
- Slightly Important
- Not At All Important

You selected "... because I feel sad"How important is this statement to you?

- Extremely Important
- Very Important
- Moderately Important
- Slightly Important
- Not At All Important

You selected "... because it is low in fat"How important is this statement to you?

- Extremely Important
- Very Important
- Moderately Important
- Slightly Important
- Not At All Important

You selected "... because I recognize it from advertisements or have seen it on TV"How important is this statement to you?

- Extremely Important
- Very Important
- Moderately Important
- Slightly Important
- Not At All Important

You selected "... because it makes social gatherings more comfortable"How important is this statement to you?

- Extremely Important
- Very Important
- Moderately Important

- Slightly Important
- Not At All Important

You selected "... because I am supposed to eat it"How important is this statement to you?

- Extremely Important
- Very Important
- Moderately Important
- Slightly Important
- Not At All Important

You selected "... because others like it"How important is this statement to you?

- Extremely Important
- Very Important
- Moderately Important
- Slightly Important
- Not At All Important

You selected "... because I feel lonely"How important is this statement to you?

- Extremely Important
- Very Important
- Moderately Important
- Slightly Important
- Not At All Important

You selected "... because I watch my weightHow important is this statement to you?

- Extremely Important

- Very Important
- Moderately Important
- Slightly Important
- Not At All Important

You selected "... because it contains no harmful substances (e.g. pesticides, pollutants, antibiotics)}"How important is this statement to you?

- Extremely Important
- Very Important
- Moderately Important
- Slightly Important
- Not At All Important

You selected "... because it is the most convenient"How important is this statement to you?

- Extremely Important
- Very Important
- Moderately Important
- Slightly Important
- Not At All Important

You selected "... because I need energy"How important is this statement to you?

- Extremely Important
- Very Important
- Moderately Important
- Slightly Important
- Not At All Important

You selected "... because I am familiar with it"How important is this statement to you?

- Extremely Important
- Very Important
- Moderately Important
- Slightly Important
- Not At All Important

You selected "... because I like it"How important is this statement to you?

- Extremely Important
- Very Important
- Moderately Important
- Slightly Important
- Not At All Important

You selected "... because it is pleasantly filling"How important is this statement to you?

- Extremely Important
- Very Important
- Moderately Important
- Slightly Important
- Not At All Important

You selected "... in order to reward myself"How important is this statement to you?

- Extremely Important
- Very Important
- Moderately Important
- Slightly Important

- Not At All Important

You selected "... out of traditions (e.g. family traditions, special occasions)"How important is this statement to you?

- Extremely Important
- Very Important
- Moderately Important
- Slightly Important
- Not At All Important

You selected "... because it belongs to certain situations"How important is this statement to you?

- Extremely Important
- Very Important
- Moderately Important
- Slightly Important
- Not At All Important

You selected "... to maintain a balanced diet"How important is this statement to you?

- Extremely Important
- Very Important
- Moderately Important
- Slightly Important
- Not At All Important

You selected "... because it is social"How important is this statement to you?

- Extremely Important
- Very Important
- Moderately Important
- Slightly Important
- Not At All Important

You selected "... because it is inexpensive"How important is this statement to you?

- Extremely Important
- Very Important
- Moderately Important
- Slightly Important
- Not At All Important

You selected "... because it spontaneously appealed to me (e.g. situated at eye level, appealing colors)"How important is this statement to you?

- Extremely Important
- Very Important
- Moderately Important
- Slightly Important
- Not At All Important

You selected "... to avoid disappointing someone who is trying to make me happy"How important is this statement to you?

- Extremely Important
- Very Important
- Moderately Important
- Slightly Important
- Not At All Important

You selected "... because it is trendy"How important is this statement to you?

- Extremely Important
- Very Important
- Moderately Important
- Slightly Important
- Not At All Important

You selected "... because it is on sale"How important is this statement to you?

- Extremely Important
- Very Important
- Moderately Important
- Slightly Important
- Not At All Important

You selected ... because it keeps me in shape (e.g. energetic, motivated)How important is this statement to you?

- Extremely Important
- Very Important
- Moderately Important
- Slightly Important
- Not At All Important

?You selected "... because that is the only choice"How important is this statement to you

- Extremely Important
- Very Important
- Moderately Important

- ○ Slightly Important
- ○ Not At All Important

You selected "... because I want to eat it every day"How important is this statement to you?

- ○ Extremely Important
- ○ Very Important
- ○ Moderately Important
- ○ Slightly Important
- ○ Not At All Important

Please tell us all the reasons you did not eat bananas (check all that apply)

- ☐ the price is too high
- ☐ I don't like it
- ☐ it is not healthy
- ☐ it is too high in calories
- ☐ it is not not something I have desire to eat at this time
- ☐ I would never choose this because I like to eat the same food every day.
- ☐ I don't think it is a snack
- ☐ it is not organic
- ☐ I do not want to indulge myself
- ☐ it is not convenient
- ☐ I don't like the way it looks
- ☐ it is not a food I eat around other people
- ☐ the portion size was not suitable
- ☐ I had it recently and I don't want to eat the same food too often
- ☐ it is not filling enough
- ☐ it is not appropriate for the situation
- ☐ I am not supposed to eat it
- ☐ eating it makes me seem "behind the times"

- ☐ this food makes me feel sad, lonely, or frustrated
- ☐ I don't usually eat it

You selected "I don't think it is a snack"How important is this statement to you?

- ○ Extremely Important
- ○ Very Important
- ○ Moderately Important
- ○ Slightly Important
- ○ Not At All Important

You selected "I don't like it"How important is this statement to you?

- ○ Extremely Important
- ○ Very Important
- ○ Moderately Important
- ○ Slightly Important
- ○ Not At All Important

You selected "I don't usually eat it"How important is this statement to you?

- ○ Extremely Important
- ○ Very Important
- ○ Moderately Important
- ○ Slightly Important
- ○ Not At All Important

You selected "the portion size was not suitable"How important is this statement to you?

- ○ Extremely Important

- Very Important
- Moderately Important
- Slightly Important
- Not At All Important

You selected "it is not filling enough"How important is this statement to you?

- Extremely Important
- Very Important
- Moderately Important
- Slightly Important
- Not At All Important

You selected "it is not healthy"How important is this statement to you?

- Extremely Important
- Very Important
- Moderately Important
- Slightly Important
- Not At All Important

You selected "it is not convenient"How important is this statement to you?

- Extremely Important
- Very Important
- Moderately Important
- Slightly Important
- Not At All Important

You selected "I do not want to indulge myself"How important is this statement to you?

- Extremely Important
- Very Important
- Moderately Important
- Slightly Important
- Not At All Important

You selected "it is not appropriate for the situation"How important is this statement to you?

- Extremely Important
- Very Important
- Moderately Important
- Slightly Important
- Not At All Important

You selected "it is not a food I eat around other people"How important is this statement to you?

- Extremely Important
- Very Important
- Moderately Important
- Slightly Important
- Not At All Important

You selected "the price is too high"How important is this statement to you?

- Extremely Important
- Very Important
- Moderately Important
- Slightly Important
- Not At All Important

You selected "I don't like the way it looks"How important is this statement to you?

- Extremely Important
- Very Important
- Moderately Important
- Slightly Important
- Not At All Important

You selected "it is too high in calories"How important is this statement to you?

- Extremely Important
- Very Important
- Moderately Important
- Slightly Important
- Not At All Important

You selected "I am not supposed to eat it"How important is this statement to you?

- Extremely Important
- Very Important
- Moderately Important
- Slightly Important
- Not At All Important

You selected "eating it makes me seem "behind the times""How important is this statement to you?

- Extremely Important
- Very Important
- Moderately Important
- Slightly Important

- Not At All Important

You selected "it is not not something I have desire to eat at this time"How important is this statement to you?

- Extremely Important
- Very Important
- Moderately Important
- Slightly Important
- Not At All Important

You selected "I had it recently and I don't want to eat the same food too often"How important is this statement to you?

- Extremely Important
- Very Important
- Moderately Important
- Slightly Important
- Not At All Important

You selected "I would never choose this because I like to eat the same food every day."How important is this statement to you?

- Extremely Important
- Very Important
- Moderately Important
- Slightly Important
- Not At All Important

You selected "this food makes me feel sad, lonely, or frustrated"How important is this statement to you?

- Extremely Important
- Very Important
- Moderately Important
- Slightly Important
- Not At All Important

You selected "it is not organic"How important is this statement to you?

- Extremely Important
- Very Important
- Moderately Important
- Slightly Important
- Not At All Important

Reasons for Sweet food Choices(RATA)

Do you eat chocolate cake with frosting ?

- Yes
- No

What are the reasons you eat chocolate cake with frosting ? (check all that apply)

- ☐ ... because it is on sale
- ☐ ... because it is low in fat
- ☐ ... because it is inexpensive
- ☐ ... because I feel lonely
- ☐ ... to avoid disappointing someone who is trying to make me happy
- ☐ ... because I grew up with it
- ☐ ... because it is social
- ☐ ... in order to indulge myself

- [ ] ... because I want to eat it every day
- [ ] ... in order to reward myself
- [ ] ... because I am hungry
- [ ] ... because I enjoy it
- [ ] ... because it tastes good
- [ ] ... because it makes me look good in front of others
- [ ] ... so that I can spend time with other people
- [ ] ... because I am frustrated
- [ ] ... because I like it
- [ ] ... because I watch my weight
- [ ] ... because it is easy to prepare
- [ ] ... because I usually eat it
- [ ] ... because it belongs to certain situations
- [ ] ... because I am supposed to eat it
- [ ] ... because I don't want to spend any more money
- [ ] ... because it is the most convenient
- [ ] ... because it is quick to prepare
- [ ] ... because it makes social gatherings more comfortable
- [ ] ... because it contains no harmful substances (e.g. pesticides, pollutants, antibiotics)
- [ ] ... because it would be impolite not to eat it
- [ ] ... to maintain a balanced diet
- [ ] ... because it is pleasantly filling
- [ ] ... because it is trendy
- [ ] ... because I recognize it from advertisements or have seen it on TV
- [ ] ... because it is low in calories
- [ ] ... because I have an appetite for it
- [ ] ... because the presentation is appealing (e.g. packaging)
- [ ] ... because it is organic
- [ ] ... out of traditions (e.g. family traditions, special occasions)
- [ ] ... because it spontaneously appealed to me (e.g. situated at eye level, appealing colors)
- [ ] ... because I am familiar with it

- ☐ ... because that is the only choice
- ☐ ... because I feel sad
- ☐ ... because I am accustomed to eating it
- ☐ ... because others like it
- ☐ ... because I need energy
- ☐ ... because it is natural (e.g. not genetically modified)
- ☐ ... because it keeps me in shape (e.g. energetic, motivated)
- ☐ ... because it is healthy

You selected " ... because I am accustomed to eating it" How important is this statement to you?

- ○ Extremely Important
- ○ Very Important
- ○ Moderately Important
- ○ Slightly Important
- ○ Not At All Important

You selected "... because it is healthy"How important is this statement to you?

- ○ Extremely Important
- ○ Very Important
- ○ Moderately Important
- ○ Slightly Important
- ○ Not At All Important

You selected "... because it is quick to prepare"How important is this statement to you?

- ○ Extremely Important
- ○ Very Important
- ○ Moderately Important

- Slightly Important
- Not At All Important

You selected "... because it tastes good"How important is this statement to you?

- Extremely Important
- Very Important
- Moderately Important
- Slightly Important
- Not At All Important

You selected "... in order to indulge myself"How important is this statement to you?

- Extremely Important
- Very Important
- Moderately Important
- Slightly Important
- Not At All Important

You Selected "... because it is natural (e.g. not genetically modified)"
How important is this statement to you?

- Extremely Important
- Very Important
- Moderately Important
- Slightly Important
- Not At All Important

You selected "... because I don't want to spend any more money"
How important is this statement to you?

- Extremely Important
- Very Important
- Moderately Important
- Slightly Important
- Not At All Important

You selected "... because it is low in calories"
How important is this statement to you?

- Extremely Important
- Very Important
- Moderately Important
- Slightly Important
- Not At All Important

You selected "... because I am frustrated"
How important is this statement to you?

- Extremely Important
- Very Important
- Moderately Important
- Slightly Important
- Not At All Important

You selected "... because it makes me look good in front of others"
How important is this statement to you?

- Extremely Important
- Very Important
- Moderately Important
- Slightly Important
- Not At All Important

You selected "... because it would be impolite not to eat it"
How important is this statement to you?

- Extremely Important
- Very Important
- Moderately Important
- Slightly Important
- Not At All Important

You selected "... because the presentation is appealing (e.g. packaging)"
How important is this statement to you?

- Extremely Important
- Very Important
- Moderately Important
- Slightly Important
- Not At All Important

You selected "... so that I can spend time with other people"
How important is this statement to you?

- Extremely Important
- Very Important
- Moderately Important
- Slightly Important
- Not At All Important

You selected "... because it is organic"
How important is this statement to you?

- Extremely Important
- Very Important
- Moderately Important
- Slightly Important
- Not At All Important

You selected "... because I grew up with it"
How important is this statement to you?

- Extremely Important
- Very Important
- Moderately Important
- Slightly Important
- Not At All Important

You selected "... because I enjoy it"
How important is this statement to you?

- Extremely Important
- Very Important
- Moderately Important
- Slightly Important
- Not At All Important

You selected "... because I am hungry"
How important is this statement to you?

- Extremely Important
- Very Important
- Moderately Important
- Slightly Important
- Not At All Important

You selected "... because I usually eat it"
How important is this statement to you?

- Extremely Important
- Very Important
- Moderately Important
- Slightly Important
- Not At All Important

You selected "... because I have an appetite for it"
How important is this statement to you?

- Extremely Important
- Very Important
- Moderately Important
- Slightly Important
- Not At All Important

You selected "... because it is easy to prepare"
How important is this statement to you?

- Extremely Important
- Very Important
- Moderately Important
- Slightly Important
- Not At All Important

You selected "... because I feel sad"
How important is this statement to you?

- Extremely Important
- Very Important
- Moderately Important
- Slightly Important
- Not At All Important

You selected "... because it is low in fat"
How important is this statement to you?

- Extremely Important
- Very Important
- Moderately Important
- Slightly Important
- Not At All Important

You selected "... because I recognize it from advertisements or have seen it on TV"
How important is this statement to you?

- Extremely Important
- Very Important
- Moderately Important
- Slightly Important
- Not At All Important

You selected "... because it makes social gatherings more comfortable"
How important is this statement to you?

- Extremely Important
- Very Important
- Moderately Important
- Slightly Important
- Not At All Important

You selected "... because I am supposed to eat it"
How important is this statement to you?

- Extremely Important
- Very Important
- Moderately Important
- Slightly Important
- Not At All Important

You selected "... because others like it"
How important is this statement to you?

- Extremely Important
- Very Important
- Moderately Important
- Slightly Important
- Not At All Important

You selected "... because I feel lonely"
How important is this statement to you?

- Extremely Important
- Very Important
- Moderately Important
- Slightly Important
- Not At All Important

You selected "... because I watch my weight
How important is this statement to you?

- Extremely Important
- Very Important
- Moderately Important
- Slightly Important
- Not At All Important

You selected "... because it contains no harmful substances (e.g. pesticides, pollutants, antibiotics)}"
How important is this statement to you?

- Extremely Important
- Very Important
- Moderately Important
- Slightly Important
- Not At All Important

You selected "... because it is the most convenient"
How important is this statement to you?

- Extremely Important
- Very Important
- Moderately Important
- Slightly Important
- Not At All Important

You selected "... because I need energy"
How important is this statement to you?

- Extremely Important
- Very Important
- Moderately Important
- Slightly Important

- Not At All Important

You selected "... because I am familiar with it"
How important is this statement to you?

- Extremely Important
- Very Important
- Moderately Important
- Slightly Important
- Not At All Important

You selected "... because I like it"
How important is this statement to you?

- Extremely Important
- Very Important
- Moderately Important
- Slightly Important
- Not At All Important

You selected "... because it is pleasantly filling"
How important is this statement to you?

- Extremely Important
- Very Important
- Moderately Important
- Slightly Important
- Not At All Important

You selected "... in order to reward myself"
How important is this statement to you?

- Extremely Important
- Very Important
- Moderately Important
- Slightly Important
- Not At All Important

You selected "... out of traditions (e.g. family traditions, special occasions)"
How important is this statement to you?

- Extremely Important
- Very Important
- Moderately Important
- Slightly Important
- Not At All Important

You selected "... because it belongs to certain situations"
How important is this statement to you?

- Extremely Important
- Very Important
- Moderately Important
- Slightly Important
- Not At All Important

You selected "... to maintain a balanced diet"
How important is this statement to you?

- Extremely Important
- Very Important
- Moderately Important
- Slightly Important

- Not At All Important

You selected "... because it is social"
How important is this statement to you?

- Extremely Important
- Very Important
- Moderately Important
- Slightly Important
- Not At All Important

You selected "... because it is inexpensive"
How important is this statement to you?

- Extremely Important
- Very Important
- Moderately Important
- Slightly Important
- Not At All Important

You selected "... because it spontaneously appealed to me (e.g. situated at eye level, appealing colors)"
How important is this statement to you?

- Extremely Important
- Very Important
- Moderately Important
- Slightly Important
- Not At All Important

You selected "... to avoid disappointing someone who is trying to make me happy"

How important is this statement to you?

- Extremely Important
- Very Important
- Moderately Important
- Slightly Important
- Not At All Important

You selected "... because it is trendy"
How important is this statement to you?

- Extremely Important
- Very Important
- Moderately Important
- Slightly Important
- Not At All Important

You selected "... because it is on sale"
How important is this statement to you?

- Extremely Important
- Very Important
- Moderately Important
- Slightly Important
- Not At All Important

You selected ... because it keeps me in shape (e.g. energetic, motivated)
How important is this statement to you?

- Extremely Important
- Very Important
- Moderately Important

- Slightly Important
- Not At All Important

?You selected "... because that is the only choice"
How important is this statement to you

- Extremely Important
- Very Important
- Moderately Important
- Slightly Important
- Not At All Important

You selected "... because I want to eat it every day"
How important is this statement to you?

- Extremely Important
- Very Important
- Moderately Important
- Slightly Important
- Not At All Important

Please tell us all the reasons you did not eat chocolate cake with frosting
(check all that apply)

- I don't usually eat it
- I had it recently and I don't want to eat the same food too often
- the price is too high
- I am not supposed to eat it
- it is not organic
- it is not healthy
- eating it makes me seem "behind the times"
- it is too high in calories

- [ ] it is not a food I eat around other people
- [ ] it is not not something I have desire to eat at this time
- [ ] I would never choose this because I like to eat the same food every day.
- [ ] this food makes me feel sad, lonely, or frustrated
- [ ] it is not appropriate for the situation
- [ ] the portion size was not suitable
- [ ] I don't think it is a snack
- [ ] it is not convenient
- [ ] I don't like it
- [ ] I don't like the way it looks
- [ ] it is not filling enough
- [ ] I do not want to indulge myself

You selected "I don't think it is a snack"
How important is this statement to you?

- ○ Extremely Important
- ○ Very Important
- ○ Moderately Important
- ○ Slightly Important
- ○ Not At All Important

You selected "I don't like it"
How important is this statement to you?

- ○ Extremely Important
- ○ Very Important
- ○ Moderately Important
- ○ Slightly Important
- ○ Not At All Important

You selected "I don't usually eat it"
How important is this statement to you?

- Extremely Important
- Very Important
- Moderately Important
- Slightly Important
- Not At All Important

You selected "the portion size was not suitable"
How important is this statement to you?

- Extremely Important
- Very Important
- Moderately Important
- Slightly Important
- Not At All Important

You selected "it is not filling enough"
How important is this statement to you?

- Extremely Important
- Very Important
- Moderately Important
- Slightly Important
- Not At All Important

You selected "it is not healthy"
How important is this statement to you?

- Extremely Important
- Very Important

- Moderately Important
- Slightly Important
- Not At All Important

You selected "it is not convenient"
How important is this statement to you?

- Extremely Important
- Very Important
- Moderately Important
- Slightly Important
- Not At All Important

You selected "I do not want to indulge myself"
How important is this statement to you?

- Extremely Important
- Very Important
- Moderately Important
- Slightly Important
- Not At All Important

You selected "it is not appropriate for the situation"
How important is this statement to you?

- Extremely Important
- Very Important
- Moderately Important
- Slightly Important
- Not At All Important

You selected "it is not a food I eat around other people"
How important is this statement to you?

- Extremely Important
- Very Important
- Moderately Important
- Slightly Important
- Not At All Important

You selected "the price is too high"
How important is this statement to you?

- Extremely Important
- Very Important
- Moderately Important
- Slightly Important
- Not At All Important

You selected "I don't like the way it looks"
How important is this statement to you?

- Extremely Important
- Very Important
- Moderately Important
- Slightly Important
- Not At All Important

You selected "it is too high in calories"
How important is this statement to you?

- Extremely Important
- Very Important

- Moderately Important
- Slightly Important
- Not At All Important

You selected "I am not supposed to eat it"
How important is this statement to you?

- Extremely Important
- Very Important
- Moderately Important
- Slightly Important
- Not At All Important

You selected "eating it makes me seem "behind the times""
How important is this statement to you?

- Extremely Important
- Very Important
- Moderately Important
- Slightly Important
- Not At All Important

You selected "it is not not something I have desire to eat at this time"
How important is this statement to you?

- Extremely Important
- Very Important
- Moderately Important
- Slightly Important
- Not At All Important

You selected "I had it recently and I don't want to eat the same food too often"
How important is this statement to you?

- Extremely Important
- Very Important
- Moderately Important
- Slightly Important
- Not At All Important

You selected "I would never choose this because I like to eat the same food every day."
How important is this statement to you?

- Extremely Important
- Very Important
- Moderately Important
- Slightly Important
- Not At All Important

You selected "this food makes me feel sad, lonely, or frustrated"
How important is this statement to you?

- Extremely Important
- Very Important
- Moderately Important
- Slightly Important
- Not At All Important

You selected "it is not organic"
How important is this statement to you?

- Extremely Important

- Very Important
- Moderately Important
- Slightly Important
- Not At All Important

Reasons for Starchy Food Choices(Full RATA)

Do you eat baked potatoes?

- Yes
- No

Thinking about the reasons you eat baked potatoes.

How important is this statement to you? **...**because I am accustomed to eating it

- Extremely Important
- Very Important
- Moderately Important
- Slightly Important
- Not At All Important

Thinking about the reasons you eat baked potatoes.

How important is this statement to you? ... because it is healthy

- Extremely Important
- Very Important
- Moderately Important
- Slightly Important
- Not At All Important

Thinking about the reasons you eat baked potatoes.

How important is this statement to you? ... because it is quick to prepare

- Extremely Important
- Very Important
- Moderately Important
- Slightly Important
- Not At All Important

Thinking about the reasons you eat baked potatoes.

How important is this statement to you? ... because it tastes good

- Extremely Important
- Very Important
- Moderately Important
- Slightly Important
- Not At All Important

Thinking about the reasons you eat baked potatoes.

How important is this statement to you? ... in order to indulge myself

- Extremely Important
- Very Important
- Moderately Important
- Slightly Important
- Not At All Important

Thinking about the reasons you eat baked potatoes.

How important is this statement to you? ... because it is natural (e.g. not genetically modified)

- Extremely Important
- Very Important

- Moderately Important
- Slightly Important
- Not At All Important

Thinking about the reasons you eat baked potatoes.

How important is this statement to you? ... because I don't want to spend any more money

- Extremely Important
- Very Important
- Moderately Important
- Slightly Important
- Not At All Important

Thinking about the reasons you eat baked potatoes.

How important is this statement to you? ... because it is low in calories

- Extremely Important
- Very Important
- Moderately Important
- Slightly Important
- Not At All Important

Thinking about the reasons you eat baked potatoes.

How important is this statement to you? ... because I am frustrated

- Extremely Important
- Very Important
- Moderately Important
- Slightly Important

- Not At All Important

Thinking about the reasons you eat baked potatoes.

How important is this statement to you? ... because it makes me look good in front of others

- Extremely Important
- Very Important
- Moderately Important
- Slightly Important
- Not At All Important

Thinking about the reasons you eat baked potatoes.

How important is this statement to you? ... because it would be impolite not to eat it

- Extremely Important
- Very Important
- Moderately Important
- Slightly Important
- Not At All Important

Thinking about the reasons you eat baked potatoes.

How important is this statement to you? ... because the presentation is appealing (e.g. packaging)

- Extremely Important
- Very Important
- Moderately Important
- Slightly Important
- Not At All Important

Thinking about the reasons you eat baked potatoes.

How important is this statement to you? ... so that I can spend time with other people

- Extremely Important
- Very Important
- Moderately Important
- Slightly Important
- Not At All Important

Thinking about the reasons you eat baked potatoes.

How important is this statement to you? ... because it is organic

- Extremely Important
- Very Important
- Moderately Important
- Slightly Important
- Not At All Important

Thinking about the reasons you eat baked potatoes.

How important is this statement to you? ... because I grew up with it

- Extremely Important
- Very Important
- Moderately Important
- Slightly Important
- Not At All Important

Thinking about the reasons you eat baked potatoes.

How important is this statement to you? ... because I enjoy it

- Extremely Important
- Very Important
- Moderately Important
- Slightly Important

- Not At All Important

Thinking about the reasons you eat baked potatoes.

How important is this statement to you? ... because I was hungry

- Extremely Important
- Very Important
- Moderately Important
- Slightly Important
- Not At All Important

Thinking about the reasons you eat baked potatoes.

How important is this statement to you? ... because I usually eat it

- Extremely Important
- Very Important
- Moderately Important
- Slightly Important
- Not At All Important

Thinking about the reasons you eat baked potatoes.

How important is this statement to you?... because I have an appetite for it

- Extremely Important
- Very Important
- Moderately Important
- Slightly Important
- Not At All Important

Thinking about the reasons you eat baked potatoes.

How important is this statement to you?... because it is easy to prepare

- Extremely Important
- Very Important
- Moderately Important
- Slightly Important
- Not At All Important

Thinking about the reasons you eat baked potatoes.

How important is this statement to you?... because I feel sad

- Extremely Important
- Very Important
- Moderately Important
- Slightly Important
- Not At All Important

Thinking about the reasons you eat baked potatoes.

How important is this statement to you?... because it is low in fat

- Extremely Important
- Very Important
- Moderately Important
- Slightly Important
- Not At All Important

Thinking about the reasons you eat baked potatoes.

How important is this statement to you?... because I recognize it from advertisements or have seen it on TV

- Extremely Important
- Very Important
- Moderately Important
- Slightly Important
- Not At All Important

Thinking about the reasons you eat baked potatoes.

How important is this statement to you?... because it makes social gatherings more comfortable

- Extremely Important
- Very Important
- Moderately Important
- Slightly Important
- Not At All Important

Thinking about the reasons you eat baked potatoes.

How important is this statement to you?... because I am supposed to eat it

- Extremely Important
- Very Important
- Moderately Important
- Slightly Important
- Not At All Important

Thinking about the reasons you eat baked potatoes.

How important is this statement to you?... because others like it

- Extremely Important
- Very Important
- Moderately Important
- Slightly Important
- Not At All Important

Thinking about the reasons you eat baked potatoes.

How important is this statement to you?... because I feel lonely

- Extremely Important
- Very Important
- Moderately Important
- Slightly Important
- Not At All Important

Thinking about the reasons you eat baked potatoes.

How important is this statement to you?... because I watch my weight

- Extremely Important
- Very Important
- Moderately Important
- Slightly Important
- Not At All Important

Thinking about the reasons you eat baked potatoes.

How important is this statement to you?... because it contains no harmful substances (e.g. pesticides, pollutants, antibiotics)

- Extremely Important
- Very Important
- Moderately Important
- Slightly Important
- Not At All Important

Thinking about the reasons you eat baked potatoes.

How important is this statement to you?... because it is the most convenient

- Extremely Important
- Very Important
- Moderately Important
- Slightly Important
- Not At All Important

Thinking about the reasons you eat baked potatoes.

How important is this statement to you?... because I need energy

- Extremely Important
- Very Important
- Moderately Important
- Slightly Important
- Not At All Important

Thinking about the reasons you eat baked potatoes.

How important is this statement to you?... because I am familiar with it

- Extremely Important
- Very Important
- Moderately Important
- Slightly Important
- Not At All Important

Thinking about the reasons you eat baked potatoes.

How important is this statement to you?... because I like it

- Extremely Important
- Very Important
- Moderately Important
- Slightly Important
- Not At All Important

Thinking about the reasons you eat baked potatoes.

How important is this statement to you?... because it is pleasantly filling

- Extremely Important
- Very Important
- Moderately Important
- Slightly Important
- Not At All Important

Thinking about the reasons you eat baked potatoes.

How important is this statement to you?... in order to reward myself

- Extremely Important
- Very Important
- Moderately Important
- Slightly Important
- Not At All Important

Thinking about the reasons you eat baked potatoes.

How important is this statement to you?... out of traditions (e.g. family traditions, special occasions)

- Extremely Important
- Very Important
- Moderately Important
- Slightly Important
- Not At All Important

Thinking about the reasons you eat baked potatoes.

How important is this statement to you?... because it belongs to certain situations

- Extremely Important
- Very Important
- Moderately Important
- Slightly Important
- Not At All Important

Thinking about the reasons you eat baked potatoes.

How important is this statement to you?... to maintain a balanced diet

- Extremely Important
- Very Important
- Moderately Important
- Slightly Important
- Not At All Important

Thinking about the reasons you eat baked potatoes.

How important is this statement to you?... because it is social

- Extremely Important
- Very Important
- Moderately Important
- Slightly Important
- Not At All Important

Thinking about the reasons you eat baked potatoes.

How important is this statement to you?... because it is inexpensive

- Extremely Important
- Very Important
- Moderately Important
- Slightly Important
- Not At All Important

Thinking about the reasons you eat baked potatoes.

How important is this statement to you?... because it spontaneously appealed to me (e.g. situated at eye level, appealing colors)

- Extremely Important
- Very Important
- Moderately Important
- Slightly Important
- Not At All Important

Thinking about the reasons you eat baked potatoes.

How important is this statement to you?... to avoid disappointing someone who is trying to make me happy

- Extremely Important
- Very Important
- Moderately Important
- Slightly Important
- Not At All Important

Thinking about the reasons you eat baked potatoes.

How important is this statement to you?... because it is trendy

- Extremely Important
- Very Important
- Moderately Important
- Slightly Important
- Not At All Important

Thinking about the reasons you eat baked potatoes.

How important is this statement to you?... because it is on sale

- Extremely Important
- Very Important
- Moderately Important
- Slightly Important
- Not At All Important

Thinking about the reasons you eat baked potatoes.

How important is this statement to you?... because it keeps me in shape (e.g. energetic, motivated)

- Extremely Important
- Very Important
- Moderately Important
- Slightly Important
- Not At All Important

Thinking about the reasons you eat baked potatoes.

How important is this statement to you?... because that was the only choice

- Extremely Important
- Very Important
- Moderately Important
- Slightly Important
- Not At All Important

Thinking about the reasons you eat baked potatoes.

How important is this statement to you?... because I want to eat it every day

- Extremely Important
- Very Important
- Moderately Important
- Slightly Important
- Not At All Important

Thinking about the reasons you do not eat baked potatoes.

How important is this statement to you?... I don't think it is a snack

- Extremely Important
- Very Important
- Moderately Important
- Slightly Important
- Not At All Important

Thinking about the reasons you do not eat baked potatoes.

How important is this statement to you?... I don't like it

- Extremely Important
- Very Important
- Moderately Important
- Slightly Important
- Not At All Important

Thinking about the reasons you do not eat baked potatoes.

How important is this statement to you?... I don't usually eat it

- Extremely Important
- Very Important
- Moderately Important
- Slightly Important
- Not At All Important

Thinking about the reasons you do not eat baked potatoes.

How important is this statement to you?...the portion size was not suitable

- Extremely Important
- Very Important
- Moderately Important
- Slightly Important
- Not At All Important

Thinking about the reasons you do not eat baked potatoes.

How important is this statement to you?...it is not filling enough

- Extremely Important
- Very Important
- Moderately Important
- Slightly Important
- Not At All Important

Thinking about the reasons you do not eat baked potatoes.

How important is this statement to you?...it is not healthy

- Extremely Important
- Very Important
- Moderately Important
- Slightly Important
- Not At All Important

Thinking about the reasons you do not eat baked potatoes.

How important is this statement to you?...it is not convenient

- Extremely Important
- Very Important
- Moderately Important
- Slightly Important
- Not At All Important

Thinking about the reasons you do not eat baked potatoes.

How important is this statement to you?...I did not want to indulge myself

- Extremely Important
- Very Important
- Moderately Important
- Slightly Important
- Not At All Important

Thinking about the reasons you do not eat baked potatoes.

How important is this statement to you?...it was not appropriate for the situation

- Extremely Important
- Very Important
- Moderately Important
- Slightly Important
- Not At All Important

Thinking about the reasons you do not eat baked potatoes.

How important is this statement to you?...it is not a food I eat around other people

- Extremely Important
- Very Important
- Moderately Important
- Slightly Important
- Not At All Important

Thinking about the reasons you do not eat baked potatoes.

How important is this statement to you?...the price was too high

- Extremely Important
- Very Important
- Moderately Important
- Slightly Important
- Not At All Important

Thinking about the reasons you do not eat baked potatoes.

How important is this statement to you?...I didn't like the way it looked

- Extremely Important
- Very Important
- Moderately Important
- Slightly Important
- Not At All Important

Thinking about the reasons you do not eat baked potatoes.

How important is this statement to you?...it is too high in calories

- Extremely Important
- Very Important
- Moderately Important
- Slightly Important
- Not At All Important

Thinking about the reasons you do not eat baked potatoes.

How important is this statement to you?...I am not supposed to eat it

- Extremely Important
- Very Important
- Moderately Important
- Slightly Important
- Not At All Important

Thinking about the reasons you do not eat baked potatoes.

How important is this statement to you?...eating it makes me seem "behind the times"

- Extremely Important
- Very Important
- Moderately Important
- Slightly Important
- Not At All Important

Thinking about the reasons you do not eat baked potatoes.

How important is this statement to you?...it was not something I had desire to eat at that time

- Extremely Important
- Very Important
- Moderately Important
- Slightly Important
- Not At All Important

Thinking about the reasons you do not eat baked potatoes.

How important is this statement to you?...I had it recently and I don't want to eat the same food too often

- Extremely Important
- Very Important
- Moderately Important
- Slightly Important
- Not At All Important

Thinking about the reasons you do not eat baked potatoes.

How important is this statement to you?...I would never choose this. I like to eat the same food every day

- Extremely Important
- Very Important
- Moderately Important
- Slightly Important
- Not At All Important

Thinking about the reasons you do not eat baked potatoes.

How important is this statement to you?...this food makes me feel sad, lonely, or frustrated

- Extremely Important
- Very Important
- Moderately Important
- Slightly Important
- Not At All Important

Thinking about the reasons you do not eat baked potatoes.

How important is this statement to you?...it is not organic

- Extremely Important
- Very Important
- Moderately Important
- Slightly Important
- Not At All Important

Reasons for Protein foods Choices(Full RATA)

Do you eat hamburgers?

- Yes
- No

Thinking about the reasons you eat hamburgers.

How important is this statement to you? ...because I am accustomed to eating it

- Extremely Important
- Very Important
- Moderately Important
- Slightly Important
- Not At All Important

Thinking about the reasons you eat hamburgers.

How important is this statement to you? ... because it is healthy

- Extremely Important
- Very Important
- Moderately Important
- Slightly Important
- Not At All Important

Thinking about the reasons you eat hamburgers.

How important is this statement to you? ... because it is quick to prepare

- Extremely Important
- Very Important
- Moderately Important
- Slightly Important
- Not At All Important

Thinking about the reasons you eat hamburgers.

How important is this statement to you? ... because it tastes good

- Extremely Important
- Very Important

- Moderately Important
- Slightly Important
- Not At All Important

Thinking about the reasons you eat hamburgers.

How important is this statement to you? ... in order to indulge myself

- Extremely Important
- Very Important
- Moderately Important
- Slightly Important
- Not At All Important

Thinking about the reasons you eat hamburgers.

How important is this statement to you? ... because it is natural (e.g. not genetically modified)

- Extremely Important
- Very Important
- Moderately Important
- Slightly Important
- Not At All Important

Thinking about the reasons you eat hamburgers.

How important is this statement to you? ... because I don't want to spend any more money

- Extremely Important
- Very Important
- Moderately Important
- Slightly Important
- Not At All Important

Thinking about the reasons you eat hamburgers.

How important is this statement to you? ... because it is low in calories

- Extremely Important
- Very Important
- Moderately Important
- Slightly Important
- Not At All Important

Thinking about the reasons you eat hamburgers.

How important is this statement to you? ... because I am frustrated

- Extremely Important
- Very Important
- Moderately Important
- Slightly Important
- Not At All Important

Thinking about the reasons you eat hamburgers.

How important is this statement to you? ... because it makes me look good in front of others

- Extremely Important
- Very Important
- Moderately Important
- Slightly Important
- Not At All Important

Thinking about the reasons you eat hamburgers.

How important is this statement to you? ... because it would be impolite not to eat it

- Extremely Important
- Very Important
- Moderately Important
- Slightly Important
- Not At All Important

Thinking about the reasons you eat hamburgers.

How important is this statement to you? ... because the presentation is appealing (e.g. packaging)

- Extremely Important
- Very Important
- Moderately Important
- Slightly Important
- Not At All Important

Thinking about the reasons you eat hamburgers.

How important is this statement to you? ... so that I can spend time with other people

- Extremely Important
- Very Important
- Moderately Important
- Slightly Important
- Not At All Important

Thinking about the reasons you eat hamburgers.

How important is this statement to you? ... because it is organic

- Extremely Important
- Very Important
- Moderately Important

- Slightly Important
- Not At All Important

Thinking about the reasons you eat hamburgers.

How important is this statement to you? ... because I grew up with it

- Extremely Important
- Very Important
- Moderately Important
- Slightly Important
- Not At All Important

Thinking about the reasons you eat hamburgers.

How important is this statement to you? ... because I enjoy it

- Extremely Important
- Very Important
- Moderately Important
- Slightly Important
- Not At All Important

Thinking about the reasons you eat hamburgers.

How important is this statement to you? ... because I was hungry

- Extremely Important
- Very Important
- Moderately Important
- Slightly Important
- Not At All Important

Thinking about the reasons you eat hamburgers.

How important is this statement to you? ... because I usually eat it

- Extremely Important
- Very Important
- Moderately Important
- Slightly Important
- Not At All Important

Thinking about the reasons you eat hamburgers.

How important is this statement to you?... because I have an appetite for it

- Extremely Important
- Very Important
- Moderately Important
- Slightly Important
- Not At All Important

Thinking about the reasons you eat hamburgers.

How important is this statement to you?... because it is easy to prepare

- Extremely Important
- Very Important
- Moderately Important
- Slightly Important
- Not At All Important

Thinking about the reasons you eat hamburgers.

How important is this statement to you?... because I feel sad

- Extremely Important
- Very Important
- Moderately Important
- Slightly Important
- Not At All Important

Thinking about the reasons you eat hamburgers.

How important is this statement to you?... because it is low in fat

- Extremely Important
- Very Important
- Moderately Important
- Slightly Important
- Not At All Important

Thinking about the reasons you eat hamburgers.

How important is this statement to you?... because I recognize it from advertisements or have seen it on TV

- Extremely Important
- Very Important
- Moderately Important
- Slightly Important
- Not At All Important

Thinking about the reasons you eat hamburgers.

How important is this statement to you?... because it makes social gatherings more comfortable

- Extremely Important
- Very Important
- Moderately Important

- Slightly Important
- Not At All Important

Thinking about the reasons you eat hamburgers.

How important is this statement to you?... because I am supposed to eat it

- Extremely Important
- Very Important
- Moderately Important
- Slightly Important
- Not At All Important

Thinking about the reasons you eat hamburgers.

How important is this statement to you?... because others like it

- Extremely Important
- Very Important
- Moderately Important
- Slightly Important
- Not At All Important

Thinking about the reasons you eat hamburgers.

How important is this statement to you?... because I feel lonely

- Extremely Important
- Very Important
- Moderately Important
- Slightly Important
- Not At All Important

Thinking about the reasons you eat hamburgers.

How important is this statement to you?... because I watch my weight

- Extremely Important
- Very Important
- Moderately Important
- Slightly Important
- Not At All Important

Thinking about the reasons you eat hamburgers.

How important is this statement to you?... because it contains no harmful substances (e.g. pesticides, pollutants, antibiotics)

- Extremely Important
- Very Important
- Moderately Important
- Slightly Important
- Not At All Important

Thinking about the reasons you eat hamburgers.

How important is this statement to you?... because it is the most convenient

- Extremely Important
- Very Important
- Moderately Important
- Slightly Important
- Not At All Important

Thinking about the reasons you eat hamburgers.

How important is this statement to you?... because I need energy

- Extremely Important
- Very Important

- Moderately Important
- Slightly Important
- Not At All Important

Thinking about the reasons you eat hamburgers.

How important is this statement to you?... because I am familiar with it

- Extremely Important
- Very Important
- Moderately Important
- Slightly Important
- Not At All Important

Thinking about the reasons you eat hamburgers.

How important is this statement to you?... because I like it

- Extremely Important
- Very Important
- Moderately Important
- Slightly Important
- Not At All Important

Thinking about the reasons you eat hamburgers.

How important is this statement to you?... because it is pleasantly filling

- Extremely Important
- Very Important
- Moderately Important
- Slightly Important
- Not At All Important

Thinking about the reasons you eat hamburgers.

How important is this statement to you?... in order to reward myself

- Extremely Important
- Very Important
- Moderately Important
- Slightly Important
- Not At All Important

Thinking about the reasons you eat hamburgers.

How important is this statement to you?... out of traditions (e.g. family traditions, special occasions)

- Extremely Important
- Very Important
- Moderately Important
- Slightly Important
- Not At All Important

Thinking about the reasons you eat hamburgers.

How important is this statement to you?... because it belongs to certain situations

- Extremely Important
- Very Important
- Moderately Important
- Slightly Important
- Not At All Important

Thinking about the reasons you eat hamburgers.

How important is this statement to you?... to maintain a balanced diet

- Extremely Important
- Very Important

- Moderately Important
- Slightly Important
- Not At All Important

Thinking about the reasons you eat hamburgers.

How important is this statement to you?... because it is social

- Extremely Important
- Very Important
- Moderately Important
- Slightly Important
- Not At All Important

Thinking about the reasons you eat hamburgers.

How important is this statement to you?... because it is inexpensive

- Extremely Important
- Very Important
- Moderately Important
- Slightly Important
- Not At All Important

Thinking about the reasons you eat hamburgers.

How important is this statement to you?... because it spontaneously appealed to me (e.g. situated at eye level, appealing colors)

- Extremely Important
- Very Important
- Moderately Important
- Slightly Important
- Not At All Important

Thinking about the reasons you eat hamburgers.

How important is this statement to you?... to avoid disappointing someone who is trying to make me happy

- Extremely Important
- Very Important
- Moderately Important
- Slightly Important
- Not At All Important

Thinking about the reasons you eat hamburgers.

How important is this statement to you?... because it is trendy

- Extremely Important
- Very Important
- Moderately Important
- Slightly Important
- Not At All Important

Thinking about the reasons you eat hamburgers.

How important is this statement to you?... because it is on sale

- Extremely Important
- Very Important
- Moderately Important
- Slightly Important
- Not At All Important

Thinking about the reasons you eat hamburgers.

How important is this statement to you?... because it keeps me in shape (e.g. energetic, motivated)

- Extremely Important

- Very Important
- Moderately Important
- Slightly Important
- Not At All Important

Thinking about the reasons you eat hamburgers.

How important is this statement to you?... because that was the only choice

- Extremely Important
- Very Important
- Moderately Important
- Slightly Important
- Not At All Important

Thinking about the reasons you eat hamburgers.

How important is this statement to you?... because I want to eat it every day

- Extremely Important
- Very Important
- Moderately Important
- Slightly Important
- Not At All Important

Thinking about the reasons you do not eat hamburgers.

How important is this statement to you?... I don't think it is a snack

- Extremely Important
- Very Important
- Moderately Important
- Slightly Important
- Not At All Important

Thinking about the reasons you do not eat hamburgers.

How important is this statement to you?... I don't like it

- Extremely Important
- Very Important
- Moderately Important
- Slightly Important
- Not At All Important

Thinking about the reasons you do not eat hamburgers.

How important is this statement to you?... I don't usually eat it

- Extremely Important
- Very Important
- Moderately Important
- Slightly Important
- Not At All Important

Thinking about the reasons you do not eat hamburgers.

How important is this statement to you?...the portion size was not suitable

- Extremely Important
- Very Important
- Moderately Important
- Slightly Important
- Not At All Important

Thinking about the reasons you do not eat hamburgers.

How important is this statement to you?...it is not filling enough

- Extremely Important

- Very Important
- Moderately Important
- Slightly Important
- Not At All Important

Thinking about the reasons you do not eat hamburgers.

How important is this statement to you?...it is not healthy

- Extremely Important
- Very Important
- Moderately Important
- Slightly Important
- Not At All Important

Thinking about the reasons you do not eat hamburgers.

How important is this statement to you?...it is not convenient

- Extremely Important
- Very Important
- Moderately Important
- Slightly Important
- Not At All Important

Thinking about the reasons you do not eat hamburgers.

How important is this statement to you?...I did not want to indulge myself

- Extremely Important
- Very Important
- Moderately Important
- Slightly Important
- Not At All Important

Thinking about the reasons you do not eat hamburgers.

How important is this statement to you?...it was not appropriate for the situation

- Extremely Important
- Very Important
- Moderately Important
- Slightly Important
- Not At All Important

Thinking about the reasons you do not eat hamburgers.

How important is this statement to you?...it is not a food I eat around other people

- Extremely Important
- Very Important
- Moderately Important
- Slightly Important
- Not At All Important

Thinking about the reasons you do not eat hamburgers.

How important is this statement to you?...the price was too high

- Extremely Important
- Very Important
- Moderately Important
- Slightly Important
- Not At All Important

Thinking about the reasons you do not eat hamburgers.

How important is this statement to you?...I didn't like the way it looked

- Extremely Important

- Very Important
- Moderately Important
- Slightly Important
- Not At All Important

Thinking about the reasons you do not eat hamburgers.

How important is this statement to you?...it is too high in calories

- Extremely Important
- Very Important
- Moderately Important
- Slightly Important
- Not At All Important

Thinking about the reasons you do not eat hamburgers.

How important is this statement to you?...I am not supposed to eat it

- Extremely Important
- Very Important
- Moderately Important
- Slightly Important
- Not At All Important

Thinking about the reasons you do not eat hamburgers.

How important is this statement to you?...eating it makes me seem "behind the times"

- Extremely Important
- Very Important
- Moderately Important
- Slightly Important
- Not At All Important

Thinking about the reasons you do not eat hamburgers.

How important is this statement to you?...it was not something I had desire to eat at that time

- Extremely Important
- Very Important
- Moderately Important
- Slightly Important
- Not At All Important

Thinking about the reasons you do not eat hamburgers.

How important is this statement to you?...I had it recently and I don't want to eat the same food too often

- Extremely Important
- Very Important
- Moderately Important
- Slightly Important
- Not At All Important

Thinking about the reasons you do not eat hamburgers.

How important is this statement to you?...I would never choose this because I like to eat the same food every day

- Extremely Important
- Very Important
- Moderately Important
- Slightly Important
- Not At All Important

Thinking about the reasons you do not eat hamburgers.

How important is this statement to you?...this food makes me feel sad, lonely, or frustrated

- Extremely Important

- Very Important
- Moderately Important
- Slightly Important
- Not At All Important

Thinking about the reasons you do not eat hamburgers.

How important is this statement to you?...it is not organic

- Extremely Important
- Very Important
- Moderately Important
- Slightly Important
- Not At All Important

Reasons for Dairy foods Choices(Full RATA)

Do you eat american cheese?

- Yes
- No

Thinking about the reasons you eat american cheese.

How important is this statement to you? ...because I am accustomed to eating it

- Extremely Important
- Very Important
- Moderately Important
- Slightly Important
- Not At All Important

Thinking about the reasons you eat american cheese.

How important is this statement to you? ... because it is healthy

- Extremely Important
- Very Important
- Moderately Important
- Slightly Important
- Not At All Important

Thinking about the reasons you eat american cheese.

How important is this statement to you? ... because it is quick to prepare

- Extremely Important
- Very Important
- Moderately Important
- Slightly Important
- Not At All Important

Thinking about the reasons you eat american cheese.

How important is this statement to you? ... because it tastes good

- Extremely Important
- Very Important
- Moderately Important
- Slightly Important
- Not At All Important

Thinking about the reasons you eat american cheese.

How important is this statement to you? ... in order to indulge myself

- Extremely Important
- Very Important

- Moderately Important
- Slightly Important
- Not At All Important

Thinking about the reasons you eat american cheese.

How important is this statement to you? ... because it is natural (e.g. not genetically modified)

- Extremely Important
- Very Important
- Moderately Important
- Slightly Important
- Not At All Important

Thinking about the reasons you eat american cheese.

How important is this statement to you? ... because I don't want to spend any more money

- Extremely Important
- Very Important
- Moderately Important
- Slightly Important
- Not At All Important

Thinking about the reasons you eat american cheese.

How important is this statement to you? ... because it is low in calories

- Extremely Important
- Very Important
- Moderately Important
- Slightly Important
- Not At All Important

Thinking about the reasons you eat american cheese.

How important is this statement to you? ... because I am frustrated

- Extremely Important
- Very Important
- Moderately Important
- Slightly Important
- Not At All Important

Thinking about the reasons you eat american cheese.

How important is this statement to you? ... because it makes me look good in front of others

- Extremely Important
- Very Important
- Moderately Important
- Slightly Important
- Not At All Important

Thinking about the reasons you eat american cheese.

How important is this statement to you? ... because it would be impolite not to eat it

- Extremely Important
- Very Important
- Moderately Important
- Slightly Important
- Not At All Important

Thinking about the reasons you eat american cheese.

How important is this statement to you? ... because the presentation is appealing (e.g. packaging)

- Extremely Important
- Very Important
- Moderately Important
- Slightly Important
- Not At All Important

Thinking about the reasons you eat american cheese.

How important is this statement to you? ... so that I can spend time with other people

- Extremely Important
- Very Important
- Moderately Important
- Slightly Important
- Not At All Important

Thinking about the reasons you eat american cheese.

How important is this statement to you? ... because it is organic

- Extremely Important
- Very Important
- Moderately Important
- Slightly Important
- Not At All Important

Thinking about the reasons you eat american cheese.

How important is this statement to you? ... because I grew up with it

- Extremely Important
- Very Important
- Moderately Important
- Slightly Important
- Not At All Important

Thinking about the reasons you eat american cheese.

How important is this statement to you? ... because I enjoy it

- Extremely Important
- Very Important
- Moderately Important
- Slightly Important
- Not At All Important

Thinking about the reasons you eat american cheese.

How important is this statement to you? ... because I was hungry

- Extremely Important
- Very Important
- Moderately Important
- Slightly Important
- Not At All Important

Thinking about the reasons you eat american cheese.

How important is this statement to you? ... because I usually eat it

- Extremely Important
- Very Important
- Moderately Important
- Slightly Important
- Not At All Important

Thinking about the reasons you eat american cheese.

How important is this statement to you?... because I have an appetite for it

- Extremely Important
- Very Important
- Moderately Important
- Slightly Important
- Not At All Important

Thinking about the reasons you eat american cheese.

How important is this statement to you?... because it is easy to prepare

- Extremely Important
- Very Important
- Moderately Important
- Slightly Important
- Not At All Important

Thinking about the reasons you eat american cheese.

How important is this statement to you?... because I feel sad

- Extremely Important
- Very Important
- Moderately Important
- Slightly Important
- Not At All Important

Thinking about the reasons you eat american cheese.

How important is this statement to you?... because it is low in fat

- Extremely Important
- Very Important
- Moderately Important
- Slightly Important
- Not At All Important

Thinking about the reasons you eat american cheese.

How important is this statement to you?... because I recognize it from advertisements or have seen it on TV

- Extremely Important
- Very Important
- Moderately Important
- Slightly Important
- Not At All Important

Thinking about the reasons you eat american cheese.

How important is this statement to you?... because it makes social gatherings more comfortable

- Extremely Important
- Very Important
- Moderately Important
- Slightly Important
- Not At All Important

Thinking about the reasons you eat american cheese.

How important is this statement to you?... because I am supposed to eat it

- Extremely Important
- Very Important
- Moderately Important
- Slightly Important
- Not At All Important

Thinking about the reasons you eat american cheese.

How important is this statement to you?... because others like it

- Extremely Important
- Very Important
- Moderately Important
- Slightly Important
- Not At All Important

Thinking about the reasons you eat american cheese.

How important is this statement to you?... because I feel lonely

- Extremely Important
- Very Important
- Moderately Important
- Slightly Important
- Not At All Important

Thinking about the reasons you eat american cheese.

How important is this statement to you?... because I watch my weight

- Extremely Important
- Very Important
- Moderately Important
- Slightly Important
- Not At All Important

Thinking about the reasons you eat american cheese.

How important is this statement to you?... because it contains no harmful substances (e.g. pesticides, pollutants, antibiotics)

- Extremely Important
- Very Important
- Moderately Important
- Slightly Important
- Not At All Important

Thinking about the reasons you eat american cheese.

How important is this statement to you?... because it is the most convenient

- Extremely Important
- Very Important
- Moderately Important
- Slightly Important
- Not At All Important

Thinking about the reasons you eat american cheese.

How important is this statement to you?... because I need energy

- Extremely Important
- Very Important
- Moderately Important
- Slightly Important
- Not At All Important

Thinking about the reasons you eat american cheese.

How important is this statement to you?... because I am familiar with it

- Extremely Important
- Very Important
- Moderately Important
- Slightly Important
- Not At All Important

Thinking about the reasons you eat american cheese.

How important is this statement to you?... because I like it

- Extremely Important
- Very Important
- Moderately Important
- Slightly Important
- Not At All Important

Thinking about the reasons you eat american cheese.

How important is this statement to you?... because it is pleasantly filling

- Extremely Important
- Very Important
- Moderately Important
- Slightly Important
- Not At All Important

Thinking about the reasons you eat american cheese.

How important is this statement to you?... in order to reward myself

- Extremely Important
- Very Important
- Moderately Important
- Slightly Important
- Not At All Important

Thinking about the reasons you eat american cheese.

How important is this statement to you?... out of traditions (e.g. family traditions, special occasions)

- Extremely Important
- Very Important
- Moderately Important
- Slightly Important
- Not At All Important

Thinking about the reasons you eat american cheese.

How important is this statement to you?... because it belongs to certain situations

- Extremely Important
- Very Important
- Moderately Important
- Slightly Important
- Not At All Important

Thinking about the reasons you eat american cheese.

How important is this statement to you?... to maintain a balanced diet

- Extremely Important
- Very Important
- Moderately Important
- Slightly Important
- Not At All Important

Thinking about the reasons you eat american cheese.

How important is this statement to you?... because it is social

- Extremely Important
- Very Important
- Moderately Important
- Slightly Important
- Not At All Important

Thinking about the reasons you eat american cheese.

How important is this statement to you?... because it is inexpensive

- Extremely Important
- Very Important
- Moderately Important
- Slightly Important
- Not At All Important

Thinking about the reasons you eat american cheese.

How important is this statement to you?... because it spontaneously appealed to me (e.g. situated at eye level, appealing colors)

- Extremely Important
- Very Important
- Moderately Important
- Slightly Important
- Not At All Important

Thinking about the reasons you eat american cheese.

How important is this statement to you?... to avoid disappointing someone who is trying to make me happy

- Extremely Important
- Very Important
- Moderately Important
- Slightly Important
- Not At All Important

Thinking about the reasons you eat american cheese.

How important is this statement to you?... because it is trendy

- Extremely Important
- Very Important
- Moderately Important
- Slightly Important
- Not At All Important

Thinking about the reasons you eat american cheese.

How important is this statement to you?... because it is on sale

- Extremely Important
- Very Important
- Moderately Important
- Slightly Important
- Not At All Important

Thinking about the reasons you eat american cheese.

How important is this statement to you?... because it keeps me in shape (e.g. energetic, motivated)

- Extremely Important
- Very Important
- Moderately Important
- Slightly Important
- Not At All Important

Thinking about the reasons you eat american cheese.

How important is this statement to you?... because that was the only choice

- Extremely Important
- Very Important
- Moderately Important
- Slightly Important
- Not At All Important

Thinking about the reasons you eat american cheese.

How important is this statement to you?... because I want to eat it every day

- Extremely Important
- Very Important
- Moderately Important
- Slightly Important
- Not At All Important

Thinking about the reasons you do not eat american cheese.

How important is this statement to you?... I don't think it is a snack

- Extremely Important
- Very Important
- Moderately Important
- Slightly Important
- Not At All Important

Thinking about the reasons you do not eat american cheese.

How important is this statement to you?... I don't like it

- Extremely Important
- Very Important
- Moderately Important
- Slightly Important
- Not At All Important

Thinking about the reasons you do not eat american cheese.

How important is this statement to you?... I don't usually eat it

- Extremely Important
- Very Important
- Moderately Important
- Slightly Important
- Not At All Important

Thinking about the reasons you do not eat american cheese.

How important is this statement to you?...the portion size was not suitable

- Extremely Important
- Very Important
- Moderately Important
- Slightly Important
- Not At All Important

Thinking about the reasons you do not eat american cheese.

How important is this statement to you?...it is not filling enough

- Extremely Important
- Very Important
- Moderately Important
- Slightly Important
- Not At All Important

Thinking about the reasons you do not eat american cheese.

How important is this statement to you?...it is not healthy

- Extremely Important
- Very Important
- Moderately Important
- Slightly Important
- Not At All Important

Thinking about the reasons you do not eat american cheese.

How important is this statement to you?...it is not convenient

- Extremely Important
- Very Important
- Moderately Important
- Slightly Important
- Not At All Important

Thinking about the reasons you do not eat american cheese.

How important is this statement to you?...I did not want to indulge myself

- Extremely Important
- Very Important
- Moderately Important
- Slightly Important
- Not At All Important

Thinking about the reasons you do not eat american cheese.

How important is this statement to you?...it was not appropriate for the situation

- Extremely Important
- Very Important
- Moderately Important
- Slightly Important
- Not At All Important

Thinking about the reasons you do not eat american cheese.

How important is this statement to you?...it is not a food I eat around other people

- Extremely Important
- Very Important
- Moderately Important
- Slightly Important
- Not At All Important

Thinking about the reasons you do not eat american cheese.

How important is this statement to you?...the price was too high

- Extremely Important
- Very Important
- Moderately Important
- Slightly Important
- Not At All Important

Thinking about the reasons you do not eat american cheese.

How important is this statement to you?...I didn't like the way it looked

- Extremely Important
- Very Important
- Moderately Important
- Slightly Important
- Not At All Important

Thinking about the reasons you do not eat american cheese.

How important is this statement to you?...it is too high in calories

- Extremely Important
- Very Important
- Moderately Important
- Slightly Important
- Not At All Important

Thinking about the reasons you do not eat american cheese.

How important is this statement to you?...I am not supposed to eat it

- Extremely Important
- Very Important
- Moderately Important
- Slightly Important
- Not At All Important

Thinking about the reasons you do not eat american cheese.

How important is this statement to you?...eating it makes me seem "behind the times"

- Extremely Important
- Very Important
- Moderately Important
- Slightly Important
- Not At All Important

Thinking about the reasons you do not eat american cheese.

How important is this statement to you?...it was not something I had desire to eat at that time

- Extremely Important
- Very Important
- Moderately Important
- Slightly Important
- Not At All Important

Thinking about the reasons you do not eat american cheese.

How important is this statement to you?...I had it recently and I don't want to eat the same food too often

- Extremely Important
- Very Important
- Moderately Important
- Slightly Important
- Not At All Important

Thinking about the reasons you do not eat american cheese.

How important is this statement to you?...I would never choose this because I like to eat the same food every day

- Extremely Important
- Very Important
- Moderately Important
- Slightly Important
- Not At All Important

Thinking about the reasons you do not eat american cheese.

How important is this statement to you?...this food makes me feel sad, lonely, or frustrated

- Extremely Important
- Very Important
- Moderately Important
- Slightly Important
- Not At All Important

Thinking about the reasons you do not eat american cheese.

How important is this statement to you?...it is not organic

- Extremely Important
- Very Important
- Moderately Important
- Slightly Important

- Not At All Important

Reasons for Fruit Choices(Full RATA)

Do you eat bananas?

- Yes
- No

Thinking about the reasons you eat bananas.

How important is this statement to you? ...because I am accustomed to eating it

- Extremely Important
- Very Important
- Moderately Important
- Slightly Important
- Not At All Important

Thinking about the reasons you eat bananas.

How important is this statement to you? ... because it is healthy

- Extremely Important
- Very Important
- Moderately Important
- Slightly Important
- Not At All Important

Thinking about the reasons you eat bananas.

How important is this statement to you? ... because it is quick to prepare

- Extremely Important

- Very Important
- Moderately Important
- Slightly Important
- Not At All Important

Thinking about the reasons you eat bananas.

How important is this statement to you? ... because it tastes good

- Extremely Important
- Very Important
- Moderately Important
- Slightly Important
- Not At All Important

Thinking about the reasons you eat bananas.

How important is this statement to you? ... in order to indulge myself

- Extremely Important
- Very Important
- Moderately Important
- Slightly Important
- Not At All Important

Thinking about the reasons you eat bananas.

How important is this statement to you? ... because it is natural (e.g. not genetically modified)

- Extremely Important
- Very Important
- Moderately Important
- Slightly Important
- Not At All Important

Thinking about the reasons you eat bananas.

How important is this statement to you? ... because I don't want to spend any more money

- Extremely Important
- Very Important
- Moderately Important
- Slightly Important
- Not At All Important

Thinking about the reasons you eat bananas.

How important is this statement to you? ... because it is low in calories

- Extremely Important
- Very Important
- Moderately Important
- Slightly Important
- Not At All Important

Thinking about the reasons you eat bananas.

How important is this statement to you? ... because I am frustrated

- Extremely Important
- Very Important
- Moderately Important
- Slightly Important
- Not At All Important

Thinking about the reasons you eat bananas.

How important is this statement to you? ... because it makes me look good in front of others

- Extremely Important
- Very Important
- Moderately Important
- Slightly Important
- Not At All Important

Thinking about the reasons you eat bananas.

How important is this statement to you? ... because it would be impolite not to eat it

- Extremely Important
- Very Important
- Moderately Important
- Slightly Important
- Not At All Important

Thinking about the reasons you eat bananas.

How important is this statement to you? ... because the presentation is appealing (e.g. packaging)

- Extremely Important
- Very Important
- Moderately Important
- Slightly Important
- Not At All Important

Thinking about the reasons you eat bananas.

How important is this statement to you? ... so that I can spend time with other people

- Extremely Important
- Very Important
- Moderately Important

- Slightly Important
- Not At All Important

Thinking about the reasons you eat bananas.

How important is this statement to you? ... because it is organic

- Extremely Important
- Very Important
- Moderately Important
- Slightly Important
- Not At All Important

Thinking about the reasons you eat bananas.

How important is this statement to you? ... because I grew up with it

- Extremely Important
- Very Important
- Moderately Important
- Slightly Important
- Not At All Important

Thinking about the reasons you eat bananas.

How important is this statement to you? ... because I enjoy it

- Extremely Important
- Very Important
- Moderately Important
- Slightly Important
- Not At All Important

Thinking about the reasons you eat bananas.

How important is this statement to you? ... because I was hungry

- Extremely Important
- Very Important
- Moderately Important
- Slightly Important
- Not At All Important

Thinking about the reasons you eat bananas.

How important is this statement to you? ... because I usually eat it

- Extremely Important
- Very Important
- Moderately Important
- Slightly Important
- Not At All Important

Thinking about the reasons you eat bananas.

How important is this statement to you?... because I have an appetite for it

- Extremely Important
- Very Important
- Moderately Important
- Slightly Important
- Not At All Important

Thinking about the reasons you eat bananas.

How important is this statement to you?... because it is easy to prepare

- Extremely Important
- Very Important
- Moderately Important
- Slightly Important
- Not At All Important

Thinking about the reasons you eat bananas.

How important is this statement to you?... because I feel sad

- Extremely Important
- Very Important
- Moderately Important
- Slightly Important
- Not At All Important

Thinking about the reasons you eat bananas.

How important is this statement to you?... because it is low in fat

- Extremely Important
- Very Important
- Moderately Important
- Slightly Important
- Not At All Important

Thinking about the reasons you eat bananas.

How important is this statement to you?... because I recognize it from advertisements or have seen it on TV

- Extremely Important
- Very Important
- Moderately Important

- Slightly Important
- Not At All Important

Thinking about the reasons you eat bananas.

How important is this statement to you?... because it makes social gatherings more comfortable

- Extremely Important
- Very Important
- Moderately Important
- Slightly Important
- Not At All Important

Thinking about the reasons you eat bananas.

How important is this statement to you?... because I am supposed to eat it

- Extremely Important
- Very Important
- Moderately Important
- Slightly Important
- Not At All Important

Thinking about the reasons you eat bananas.

How important is this statement to you?... because others like it

- Extremely Important
- Very Important
- Moderately Important
- Slightly Important
- Not At All Important

Thinking about the reasons you eat bananas.

How important is this statement to you?... because I feel lonely

- Extremely Important
- Very Important
- Moderately Important
- Slightly Important
- Not At All Important

Thinking about the reasons you eat bananas.

How important is this statement to you?... because I watch my weight

- Extremely Important
- Very Important
- Moderately Important
- Slightly Important
- Not At All Important

Thinking about the reasons you eat bananas.

How important is this statement to you?... because it contains no harmful substances (e.g. pesticides, pollutants, antibiotics)

- Extremely Important
- Very Important
- Moderately Important
- Slightly Important
- Not At All Important

Thinking about the reasons you eat bananas.

How important is this statement to you?... because it is the most convenient

- Extremely Important
- Very Important

- Moderately Important
- Slightly Important
- Not At All Important

Thinking about the reasons you eat bananas.

How important is this statement to you?... because I need energy

- Extremely Important
- Very Important
- Moderately Important
- Slightly Important
- Not At All Important

Thinking about the reasons you eat bananas.

How important is this statement to you?... because I am familiar with it

- Extremely Important
- Very Important
- Moderately Important
- Slightly Important
- Not At All Important

Thinking about the reasons you eat bananas.

How important is this statement to you?... because I like it

- Extremely Important
- Very Important
- Moderately Important
- Slightly Important
- Not At All Important

Thinking about the reasons you eat bananas.

How important is this statement to you?... because it is pleasantly filling

- Extremely Important
- Very Important
- Moderately Important
- Slightly Important
- Not At All Important

Thinking about the reasons you eat bananas.

How important is this statement to you?... in order to reward myself

- Extremely Important
- Very Important
- Moderately Important
- Slightly Important
- Not At All Important

Thinking about the reasons you eat bananas.

How important is this statement to you?... out of traditions (e.g. family traditions, special occasions)

- Extremely Important
- Very Important
- Moderately Important
- Slightly Important
- Not At All Important

Thinking about the reasons you eat bananas.

How important is this statement to you?... because it belongs to certain situations

- Extremely Important
- Very Important

- Moderately Important
- Slightly Important
- Not At All Important

Thinking about the reasons you eat bananas.

How important is this statement to you?... to maintain a balanced diet

- Extremely Important
- Very Important
- Moderately Important
- Slightly Important
- Not At All Important

Thinking about the reasons you eat bananas.

How important is this statement to you?... because it is social

- Extremely Important
- Very Important
- Moderately Important
- Slightly Important
- Not At All Important

Thinking about the reasons you eat bananas.

How important is this statement to you?... because it is inexpensive

- Extremely Important
- Very Important
- Moderately Important
- Slightly Important
- Not At All Important

Thinking about the reasons you eat bananas.

How important is this statement to you?... because it spontaneously appealed to me (e.g. situated at eye level, appealing colors)

- Extremely Important
- Very Important
- Moderately Important
- Slightly Important
- Not At All Important

Thinking about the reasons you eat bananas.

How important is this statement to you?... to avoid disappointing someone who is trying to make me happy

- Extremely Important
- Very Important
- Moderately Important
- Slightly Important
- Not At All Important

Thinking about the reasons you eat bananas.

How important is this statement to you?... because it is trendy

- Extremely Important
- Very Important
- Moderately Important
- Slightly Important
- Not At All Important

Thinking about the reasons you eat bananas.

How important is this statement to you?... because it is on sale

- Extremely Important

- Very Important
- Moderately Important
- Slightly Important
- Not At All Important

Thinking about the reasons you eat bananas.

How important is this statement to you?... because it keeps me in shape (e.g. energetic, motivated)

- Extremely Important
- Very Important
- Moderately Important
- Slightly Important
- Not At All Important

Thinking about the reasons you eat bananas.

How important is this statement to you?... because that was the only choice

- Extremely Important
- Very Important
- Moderately Important
- Slightly Important
- Not At All Important

Thinking about the reasons you eat bananas.

How important is this statement to you?... because I want to eat it every day

- Extremely Important
- Very Important
- Moderately Important
- Slightly Important
- Not At All Important

Thinking about the reasons you do not eat bananas.

How important is this statement to you?... I don't think it is a snack

- Extremely Important
- Very Important
- Moderately Important
- Slightly Important
- Not At All Important

Thinking about the reasons you do not eat bananas.

How important is this statement to you?... I don't like it

- Extremely Important
- Very Important
- Moderately Important
- Slightly Important
- Not At All Important

Thinking about the reasons you do not eat bananas.

How important is this statement to you?... I don't usually eat it

- Extremely Important
- Very Important
- Moderately Important
- Slightly Important
- Not At All Important

Thinking about the reasons you do not eat bananas.

How important is this statement to you?...the portion size was not suitable

- Extremely Important

- Very Important
- Moderately Important
- Slightly Important
- Not At All Important

Thinking about the reasons you do not eat bananas.

How important is this statement to you?...it is not filling enough

- Extremely Important
- Very Important
- Moderately Important
- Slightly Important
- Not At All Important

Thinking about the reasons you do not eat bananas.

How important is this statement to you?...it is not healthy

- Extremely Important
- Very Important
- Moderately Important
- Slightly Important
- Not At All Important

Thinking about the reasons you do not eat bananas.

How important is this statement to you?...it is not convenient

- Extremely Important
- Very Important
- Moderately Important
- Slightly Important
- Not At All Important

Thinking about the reasons you do not eat bananas.

How important is this statement to you?...I did not want to indulge myself

- Extremely Important
- Very Important
- Moderately Important
- Slightly Important
- Not At All Important

Thinking about the reasons you do not eat bananas.

How important is this statement to you?...it was not appropriate for the situation

- Extremely Important
- Very Important
- Moderately Important
- Slightly Important
- Not At All Important

Thinking about the reasons you do not eat bananas.

How important is this statement to you?...it is not a food I eat around other people

- Extremely Important
- Very Important
- Moderately Important
- Slightly Important
- Not At All Important

Thinking about the reasons you do not eat bananas.

How important is this statement to you?...the price was too high

- Extremely Important

- Very Important
- Moderately Important
- Slightly Important
- Not At All Important

Thinking about the reasons you do not eat bananas.

How important is this statement to you?...I didn't like the way it looked

- Extremely Important
- Very Important
- Moderately Important
- Slightly Important
- Not At All Important

Thinking about the reasons you do not eat bananas.

How important is this statement to you?...it is too high in calories

- Extremely Important
- Very Important
- Moderately Important
- Slightly Important
- Not At All Important

Thinking about the reasons you do not eat bananas.

How important is this statement to you?...I am not supposed to eat it

- Extremely Important
- Very Important
- Moderately Important
- Slightly Important
- Not At All Important

Thinking about the reasons you do not eat bananas.

How important is this statement to you?...eating it makes me seem "behind the times"

- Extremely Important
- Very Important
- Moderately Important
- Slightly Important
- Not At All Important

Thinking about the reasons you do not eat bananas.

How important is this statement to you?...it was not something I had desire to eat at that time

- Extremely Important
- Very Important
- Moderately Important
- Slightly Important
- Not At All Important

Thinking about the reasons you do not eat bananas.

How important is this statement to you?...I had it recently and I don't want to eat the same food too often

- Extremely Important
- Very Important
- Moderately Important
- Slightly Important
- Not At All Important

Thinking about the reasons you do not eat bananas.

How important is this statement to you?...I would never choose this because I like to eat the same food every day

- Extremely Important

- Very Important
- Moderately Important
- Slightly Important
- Not At All Important

Thinking about the reasons you do not eat bananas.

How important is this statement to you?...this food makes me feel sad, lonely, or frustrated

- Extremely Important
- Very Important
- Moderately Important
- Slightly Important
- Not At All Important

Thinking about the reasons you do not eat bananas.

How important is this statement to you?...it is not organic

- Extremely Important
- Very Important
- Moderately Important
- Slightly Important
- Not At All Important

Reasons for Sweet food Choices(Full RATA)

Do you eat chocolate cake with frosting ?

- Yes
- No

Thinking about the reasons you eat chocolate cake with frosting . How important is this statement to you? ...because I am accustomed to eating it

- Extremely Important
- Very Important
- Moderately Important
- Slightly Important
- Not At All Important

Thinking about the reasons you eat chocolate cake with frosting . How important is this statement to you? ... because it is healthy

- Extremely Important
- Very Important
- Moderately Important
- Slightly Important
- Not At All Important

Thinking about the reasons you eat chocolate cake with frosting . How important is this statement to you? ... because it is quick to prepare

- Extremely Important
- Very Important
- Moderately Important
- Slightly Important
- Not At All Important

Thinking about the reasons you eat chocolate cake with frosting . How important is this statement to you? ... because it tastes good

- Extremely Important
- Very Important

- Moderately Important
- Slightly Important
- Not At All Important

Thinking about the reasons you eat chocolate cake with frosting . How important is this statement to you? ... in order to indulge myself

- Extremely Important
- Very Important
- Moderately Important
- Slightly Important
- Not At All Important

Thinking about the reasons you eat chocolate cake with frosting . How important is this statement to you? ... because it is natural (e.g. not genetically modified)

- Extremely Important
- Very Important
- Moderately Important
- Slightly Important
- Not At All Important

Thinking about the reasons you eat chocolate cake with frosting . How important is this statement to you? ... because I don't want to spend any more money

- Extremely Important
- Very Important
- Moderately Important
- Slightly Important
- Not At All Important

Thinking about the reasons you eat chocolate cake with frosting . How important is this statement to you? ... because it is low in calories

- Extremely Important
- Very Important
- Moderately Important
- Slightly Important
- Not At All Important

Thinking about the reasons you eat chocolate cake with frosting . How important is this statement to you? ... because I am frustrated

- Extremely Important
- Very Important
- Moderately Important
- Slightly Important
- Not At All Important

Thinking about the reasons you eat chocolate cake with frosting . How important is this statement to you? ... because it makes me look good in front of others

- Extremely Important
- Very Important
- Moderately Important
- Slightly Important
- Not At All Important

Thinking about the reasons you eat chocolate cake with frosting . How important is this statement to you? ... because it would be impolite not to eat it

- Extremely Important

- Very Important
- Moderately Important
- Slightly Important
- Not At All Important

Thinking about the reasons you eat chocolate cake with frosting . How important is this statement to you? ... because the presentation is appealing (e.g. packaging)

- Extremely Important
- Very Important
- Moderately Important
- Slightly Important
- Not At All Important

Thinking about the reasons you eat chocolate cake with frosting . How important is this statement to you? ... so that I can spend time with other people

- Extremely Important
- Very Important
- Moderately Important
- Slightly Important
- Not At All Important

Thinking about the reasons you eat chocolate cake with frosting . How important is this statement to you? ... because it is organic

- Extremely Important
- Very Important
- Moderately Important
- Slightly Important
- Not At All Important

Thinking about the reasons you eat chocolate cake with frosting . How important is this statement to you? ... because I grew up with it

- Extremely Important
- Very Important
- Moderately Important
- Slightly Important
- Not At All Important

Thinking about the reasons you eat chocolate cake with frosting . How important is this statement to you? ... because I enjoy it

- Extremely Important
- Very Important
- Moderately Important
- Slightly Important
- Not At All Important

Thinking about the reasons you eat chocolate cake with frosting . How important is this statement to you? ... because I was hungry

- Extremely Important
- Very Important
- Moderately Important
- Slightly Important
- Not At All Important

Thinking about the reasons you eat chocolate cake with frosting . How important is this statement to you? ... because I usually eat it

- Extremely Important

- Very Important
- Moderately Important
- Slightly Important
- Not At All Important

Thinking about the reasons you eat chocolate cake with frosting . How important is this statement to you?... because I have an appetite for it

- Extremely Important
- Very Important
- Moderately Important
- Slightly Important
- Not At All Important

Thinking about the reasons you eat chocolate cake with frosting . How important is this statement to you?... because it is easy to prepare

- Extremely Important
- Very Important
- Moderately Important
- Slightly Important
- Not At All Important

Thinking about the reasons you eat chocolate cake with frosting . How important is this statement to you?... because I feel sad

- Extremely Important
- Very Important
- Moderately Important
- Slightly Important
- Not At All Important

Thinking about the reasons you eat chocolate cake with frosting . How important is this statement to you?... because it is low in fat

- Extremely Important
- Very Important
- Moderately Important
- Slightly Important
- Not At All Important

Thinking about the reasons you eat chocolate cake with frosting . How important is this statement to you?... because I recognize it from advertisements or have seen it on TV

- Extremely Important
- Very Important
- Moderately Important
- Slightly Important
- Not At All Important

Thinking about the reasons you eat chocolate cake with frosting . How important is this statement to you?... because it makes social gatherings more comfortable

- Extremely Important
- Very Important
- Moderately Important
- Slightly Important
- Not At All Important

Thinking about the reasons you eat chocolate cake with frosting . How important is this statement to you?... because I am supposed to eat it

- Extremely Important

- Very Important
- Moderately Important
- Slightly Important
- Not At All Important

Thinking about the reasons you eat chocolate cake with frosting . How important is this statement to you?... because others like it

- Extremely Important
- Very Important
- Moderately Important
- Slightly Important
- Not At All Important

Thinking about the reasons you eat chocolate cake with frosting . How important is this statement to you?... because I feel lonely

- Extremely Important
- Very Important
- Moderately Important
- Slightly Important
- Not At All Important

Thinking about the reasons you eat chocolate cake with frosting . How important is this statement to you?... because I watch my weight

- Extremely Important
- Very Important
- Moderately Important
- Slightly Important
- Not At All Important

Thinking about the reasons you eat chocolate cake with frosting . How important is this statement to you?... because it contains no harmful substances (e.g. pesticides, pollutants, antibiotics)

- Extremely Important
- Very Important
- Moderately Important
- Slightly Important
- Not At All Important

Thinking about the reasons you eat chocolate cake with frosting . How important is this statement to you?... because it is the most convenient

- Extremely Important
- Very Important
- Moderately Important
- Slightly Important
- Not At All Important

Thinking about the reasons you eat chocolate cake with frosting . How important is this statement to you?... because I need energy

- Extremely Important
- Very Important
- Moderately Important
- Slightly Important
- Not At All Important

Thinking about the reasons you eat chocolate cake with frosting . How important is this statement to you?... because I am familiar with it

- Extremely Important

- Very Important
- Moderately Important
- Slightly Important
- Not At All Important

Thinking about the reasons you eat chocolate cake with frosting . How important is this statement to you?... because I like it

- Extremely Important
- Very Important
- Moderately Important
- Slightly Important
- Not At All Important

Thinking about the reasons you eat chocolate cake with frosting . How important is this statement to you?... because it is pleasantly filling

- Extremely Important
- Very Important
- Moderately Important
- Slightly Important
- Not At All Important

Thinking about the reasons you eat chocolate cake with frosting . How important is this statement to you?... in order to reward myself

- Extremely Important
- Very Important
- Moderately Important
- Slightly Important
- Not At All Important

Thinking about the reasons you eat chocolate cake with frosting . How important is this statement to you?... out of traditions (e.g. family traditions, special occasions)

- Extremely Important
- Very Important
- Moderately Important
- Slightly Important
- Not At All Important

Thinking about the reasons you eat chocolate cake with frosting . How important is this statement to you?... because it belongs to certain situations

- Extremely Important
- Very Important
- Moderately Important
- Slightly Important
- Not At All Important

Thinking about the reasons you eat chocolate cake with frosting . How important is this statement to you?... to maintain a balanced diet

- Extremely Important
- Very Important
- Moderately Important
- Slightly Important
- Not At All Important

Thinking about the reasons you eat chocolate cake with frosting . How important is this statement to you?... because it is social

- Extremely Important

- Very Important
- Moderately Important
- Slightly Important
- Not At All Important

Thinking about the reasons you eat chocolate cake with frosting . How important is this statement to you?... because it is inexpensive

- Extremely Important
- Very Important
- Moderately Important
- Slightly Important
- Not At All Important

Thinking about the reasons you eat chocolate cake with frosting . How important is this statement to you?... because it spontaneously appealed to me (e.g. situated at eye level, appealing colors)

- Extremely Important
- Very Important
- Moderately Important
- Slightly Important
- Not At All Important

Thinking about the reasons you eat chocolate cake with frosting . How important is this statement to you?... to avoid disappointing someone who is trying to make me happy

- Extremely Important
- Very Important
- Moderately Important
- Slightly Important

- Not At All Important

Thinking about the reasons you eat chocolate cake with frosting . How important is this statement to you?... because it is trendy

- Extremely Important
- Very Important
- Moderately Important
- Slightly Important
- Not At All Important

Thinking about the reasons you eat chocolate cake with frosting . How important is this statement to you?... because it is on sale

- Extremely Important
- Very Important
- Moderately Important
- Slightly Important
- Not At All Important

Thinking about the reasons you eat chocolate cake with frosting . How important is this statement to you?... because it keeps me in shape (e.g. energetic, motivated)

- Extremely Important
- Very Important
- Moderately Important
- Slightly Important
- Not At All Important

Thinking about the reasons you eat chocolate cake with frosting . How important is this statement to you?... because that was the only choice

- Extremely Important
- Very Important
- Moderately Important
- Slightly Important
- Not At All Important

Thinking about the reasons you eat chocolate cake with frosting . How important is this statement to you?... because I want to eat it every day

- Extremely Important
- Very Important
- Moderately Important
- Slightly Important
- Not At All Important

Thinking about the reasons you do not eat chocolate cake with frosting . How important is this statement to you?... I don't think it is a snack

- Extremely Important
- Very Important
- Moderately Important
- Slightly Important
- Not At All Important

Thinking about the reasons you do not eat chocolate cake with frosting . How important is this statement to you?... I don't like it

- Extremely Important
- Very Important
- Moderately Important
- Slightly Important

- Not At All Important

Thinking about the reasons you do not eat chocolate cake with frosting . How important is this statement to you?... I don't usually eat it

- Extremely Important
- Very Important
- Moderately Important
- Slightly Important
- Not At All Important

Thinking about the reasons you do not eat chocolate cake with frosting . How important is this statement to you?...the portion size was not suitable

- Extremely Important
- Very Important
- Moderately Important
- Slightly Important
- Not At All Important

Thinking about the reasons you do not eat chocolate cake with frosting . How important is this statement to you?...it is not filling enough

- Extremely Important
- Very Important
- Moderately Important
- Slightly Important
- Not At All Important

Thinking about the reasons you do not eat chocolate cake with frosting . How important is this statement to you?...it is not healthy

- Extremely Important
- Very Important
- Moderately Important
- Slightly Important
- Not At All Important

Thinking about the reasons you do not eat chocolate cake with frosting . How important is this statement to you?...it is not convenient

- Extremely Important
- Very Important
- Moderately Important
- Slightly Important
- Not At All Important

Thinking about the reasons you do not eat chocolate cake with frosting . How important is this statement to you?...I did not want to indulge myself

- Extremely Important
- Very Important
- Moderately Important
- Slightly Important
- Not At All Important

Thinking about the reasons you do not eat chocolate cake with frosting . How important is this statement to you?...it was not appropriate for the situation

- Extremely Important
- Very Important
- Moderately Important
- Slightly Important

- Not At All Important

Thinking about the reasons you do not eat chocolate cake with frosting . How important is this statement to you?...it is not a food I eat around other people

- Extremely Important
- Very Important
- Moderately Important
- Slightly Important
- Not At All Important

Thinking about the reasons you do not eat chocolate cake with frosting . How important is this statement to you?...the price was too high

- Extremely Important
- Very Important
- Moderately Important
- Slightly Important
- Not At All Important

Thinking about the reasons you do not eat chocolate cake with frosting . How important is this statement to you?...I didn't like the way it looked

- Extremely Important
- Very Important
- Moderately Important
- Slightly Important
- Not At All Important

Thinking about the reasons you do not eat chocolate cake with frosting . How important is this statement to you?...it is too high in calories

- Extremely Important
- Very Important
- Moderately Important
- Slightly Important
- Not At All Important

Thinking about the reasons you do not eat chocolate cake with frosting . How important is this statement to you?...I am not supposed to eat it

- Extremely Important
- Very Important
- Moderately Important
- Slightly Important
- Not At All Important

Thinking about the reasons you do not eat chocolate cake with frosting . How important is this statement to you?...eating it makes me seem "behind the times"

- Extremely Important
- Very Important
- Moderately Important
- Slightly Important
- Not At All Important

Thinking about the reasons you do not eat chocolate cake with frosting . How important is this statement to you?...it was not something I had desire to eat at that time

- Extremely Important
- Very Important
- Moderately Important

- Slightly Important
- Not At All Important

Thinking about the reasons you do not eat chocolate cake with frosting . How important is this statement to you?...I had it recently and I don't want to eat the same food too often

- Extremely Important
- Very Important
- Moderately Important
- Slightly Important
- Not At All Important

Thinking about the reasons you do not eat chocolate cake with frosting . How important is this statement to you?...I would never choose this because I like to eat the same food every day

- Extremely Important
- Very Important
- Moderately Important
- Slightly Important
- Not At All Important

Thinking about the reasons you do not eat chocolate cake with frosting . How important is this statement to you?...this food makes me feel sad, lonely, or frustrated

- Extremely Important
- Very Important
- Moderately Important
- Slightly Important
- Not At All Important

Thinking about the reasons you do not eat chocolate cake with frosting . How important is this statement to you?...it is not organic

- Extremely Important
- Very Important
- Moderately Important
- Slightly Important
- Not At All Important

Powered by Qualtrics
