# Supplementary material for: Comparing Four Question Formats in Five Languages for On-Line Consumer Surveys
Source: Methods Protoc. 2020 Jul 14;3(3):49. doi: 10.3390/mps3030049 (PMC7563457; doi:10.3390/mps3030049)
Supplement: Supplementary file 1 [file mps-03-00049-s001.zip › supplementary/Figure S2 (Brazil-Portuguese).pdf]

Demographics

Qual seu gênero?

- ◯ Masculino
- ◯ Feminino

Que ano você nasceu?

- ◯ 1943 ou antes
- ◯ 1944 - 1964
- ◯ 1965 - 1979
- ◯ 1980 - 1994
- ◯ 1995 -2001
- ◯ 2002 ou depois

Nível acadêmico completo

- ◯ Escola primária ou inferior
- ◯ Ensino Medio
- ◯ Ensino Superior

Quantos adultos vivem em sua casa incluindo você?

- ◯ 1
- ◯ 2

- 3
- 4
- 5 +

Quantos menores de 18 anos vivem em sua casa?

- 0
- 1
- 2
- 3
- 4 +

Nós nos importamos com a qualidade dos dados da nossa pesquisa e esperamos receber suas opiniões da maneira mais acurada, portanto, é importante para nós que você responda a cada questão dessa pesquisa cuidadosamente.

Você se compromete em fornecer as respostas de maneira cuidadosa e honesta, nessa pequisa?

- Eu irei fornecer as minhas melhores respostas
- Eu não irei fornecer as minhas melhoras respostas
- Eu não posso prometer qualquer uma das duas formas

Food Involvement

A pesquisa que você realizará é sobre comida.

Por favor, indique o quanto você concorda/discorda com as afirmações abaixo:

Eu não fico pensando em comida todos os dias

- Discorda plenamente
- Discorda
- Discorda parcialmente
- Nem concorda ou discorda
- Concorda parcialmente
- Concorda
- Concorda plenamente

Cozinhar não é muito agradável

- Discorda plenamente
- Discorda
- Discorda parcialmente
- Nem concorda ou discorda
- Concorda parcialmente
- Concorda
- Concorda plenamente

Eu gosto de falar sobre o que comi e o que comerei

- Discorda plenamente
- Discorda
- Discorda parcialmente
- Nem concorda ou discorda
- Concorda parcialmente
- Concorda
- Concorda plenamente

Comparando-se com outras decisões diárias, minhas escolhas por determinados alimentos, não são tão importantes

- Discorda plenamente

- Discorda
- Discorda parcialmente
- Nem concorda ou discorda
- Concorda parcialmente
- Concorda
- Concorda plenamente

Quando viajo, uma das coisas que me deixa muito ansioso(a), é o que comerei no local

- Discorda plenamente
- Discorda
- Discorda parcialmente
- Nem concorda ou discorda
- Concorda parcialmente
- Concorda
- Concorda plenamente

Sempre, ou na maioria das vezes, eu limpo o prato e a mesa, após as refeições

- Discorda plenamente
- Discorda
- Discorda parcialmente
- Nem concorda ou discorda
- Concorda parcialmente
- Concorda
- Concorda plenamente

Eu gosto de cozinhar para os outros e para mim também

- Discorda plenamente
- Discorda
- Discorda parcialmente

- Nem concorda ou discorda
- Concorda parcialmente
- Concorda
- Concorda plenamente

Quando saio para comer, não fico pensando ou falando muito sobre o gosto da comida

- Discorda plenamente
- Discorda
- Discorda parcialmente
- Nem concorda ou discorda
- Concorda parcialmente
- Concorda
- Concorda plenamente

Eu não gosto de misturar ou picar alimentos

- Discorda plenamente
- Discorda
- Discorda parcialmente
- Nem concorda ou discorda
- Concorda parcialmente
- Concorda
- Concorda plenamente

Sou eu quem compro a maior parte dos meus alimentos

- Discorda plenamente
- Discorda
- Discorda parcialmente
- Nem concorda ou discorda
- Concorda parcialmente

- Concorda
- Concorda plenamente

Eu não lavo os talheres ou tiro a mesa

- Discorda plenamente
- Discorda
- Discorda parcialmente
- Nem concorda ou discorda
- Concorda parcialmente
- Concorda
- Concorda plenamente

Eu me importo se a mesa está bem arrumada

- Discorda plenamente
- Discorda
- Discorda parcialmente
- Nem concorda ou discorda
- Concorda parcialmente
- Concorda
- Concorda plenamente

Health and Taste Attitude

Eu me preocupo em relação ao alimento ser saudável

- Discorda plenamente
- Discorda
- Discorda parcialmente
- Nem concorda ou discorda

- Concorda parcialmente
- Concorda
- Concorda plenamente

Eu sempre sigo uma dieta saudável e balanceada

- Discorda plenamente
- Discorda
- Discorda parcialmente
- Nem concorda ou discorda
- Concorda parcialmente
- Concorda
- Concorda plenamente

Na minha opinião, é importante uma dieta com baixo teor de gordura

- Discorda plenamente
- Discorda
- Discorda parcialmente
- Nem concorda ou discorda
- Concorda parcialmente
- Concorda
- Concorda plenamente

É importante que a minha dieta diária tenha diversas vitaminas e minerais

- Discorda plenamente
- Discorda
- Discorda parcialmente
- Nem concorda ou discorda
- Concorda parcialmente
- Concorda

- Concorda plenamente

Eu como o que gosto sem me preocupar com o quanto o alimento é saudável!

- Discorda plenamente
- Discorda
- Discorda parcialmente
- Nem concorda ou discorda
- Concorda parcialmente
- Concorda
- Concorda plenamente

Mesmo que um alimento aumente o meu colesterol, eu não o evito

- Discorda plenamente
- Discorda
- Discorda parcialmente
- Nem concorda ou discorda
- Concorda parcialmente
- Concorda
- Concorda plenamente

O quanto um alimento é saudável, pouco influencia nas minhas escolhas alimentares

- Discorda plenamente
- Discorda
- Discorda parcialmente
- Nem concorda ou discorda
- Concorda parcialmente
- Concorda
- Concorda plenamente

O quanto um lanche ou aperitivo é saudável, não faz diferença para mim

- Discorda plenamente
- Discorda
- Discorda parcialmente
- Nem concorda ou discorda
- Concorda parcialmente
- Concorda
- Concorda plenamente

Eu não acredito que o alimento tenha que ser sempre uma fonte de prazer

- Discorda plenamente
- Discorda
- Discorda parcialmente
- Nem concorda ou discorda
- Concorda parcialmente
- Concorda
- Concorda plenamente

A aparência do alimento não faz diferença, na minha opinão

- Discorda plenamente
- Discorda
- Discorda parcialmente
- Nem concorda ou discorda
- Concorda parcialmente
- Concorda
- Concorda plenamente

Na minha opinião é importante comer alimentos deliciosos, tanto durante a semana como nos finais de semana

- Discorda plenamente
- Discorda
- Discorda parcialmente
- Nem concorda ou discorda
- Concorda parcialmente
- Concorda
- Concorda plenamente

Quando eu como um alimento, concentro-me para apreciar o seu gosto

- Discorda plenamente
- Discorda
- Discorda parcialmente
- Nem concorda ou discorda
- Concorda parcialmente
- Concorda
- Concorda plenamente

Mesmo que não goste do sabor da comida, eu a como

- Discorda plenamente
- Discorda
- Discorda parcialmente
- Nem concorda ou discorda
- Concorda parcialmente
- Concorda
- Concorda plenamente

Utilizo boa parte do meu final de semana para me alimentar de comidas deliciosas

- Discorda plenamente
- Discorda
- Discorda parcialmente
- Nem concorda ou discorda
- Concorda parcialmente
- Concorda
- Concorda plenamente

Neophobia

Frequentemente eu experimento comidas novas e diferentes

- Discorda plenamente
- Discorda
- Discorda parcialmente
- Nem concorda ou discorda
- Concorda parcialmente
- Concorda
- Concorda plenamente

Eu desconfio de novos alimentos

- Discorda plenamente
- Discorda
- Discorda parcialmente
- Nem concorda ou discorda
- Concorda parcialmente
- Concorda
- Concorda plenamente

Se desconheço o que tem na comida, eu não experimento

- Discorda plenamente
- Discorda
- Discorda parcialmente
- Nem concorda ou discorda
- Concorda parcialmente
- Concorda
- Concorda plenamente

Eu gosto de comidas de diferentes países

- Discorda plenamente
- Discorda
- Discorda parcialmente
- Nem concorda ou discorda
- Concorda parcialmente
- Concorda
- Concorda plenamente

Comidas típicas de outros países parecem muito estranhas para se comer

- Discorda plenamente
- Discorda
- Discorda parcialmente
- Nem concorda ou discorda
- Concorda parcialmente
- Concorda
- Concorda plenamente

Nas festas, eu experimento novos alimentos

- Discorda plenamente
- Discorda
- Discorda parcialmente
- Nem concorda ou discorda
- Concorda parcialmente
- Concorda
- Concorda plenamente

Eu tenho medo de comer coisas sobre as quais nunca ouvi falar

- Discorda plenamente
- Discorda
- Discorda parcialmente
- Nem concorda ou discorda
- Concorda parcialmente
- Concorda
- Concorda plenamente

Eu me preocupo muito com os alimentos que vou comer

- Discorda plenamente
- Discorda
- Discorda parcialmente
- Nem concorda ou discorda
- Concorda parcialmente
- Concorda
- Concorda plenamente

Eu como de tudo.

- Discorda plenamente

- Discorda
- Discorda parcialmente
- Nem concorda ou discorda
- Concorda parcialmente
- Concorda
- Concorda plenamente

Eu gosto de provar novos restaurantes de comidas típicas ou estrangeiras

- Discorda plenamente
- Discorda
- Discorda parcialmente
- Nem concorda ou discorda
- Concorda parcialmente
- Concorda
- Concorda plenamente

Reasons for Starchy food choices(CATA)

Você consome arroz?

- Sim
- Não

Quais as razões pelas quais você come arroz? (Marque todas as respostas que correspondem)

- ☐ ... porque eu vejo propaganda em algum lugar ou na TV
- ☐ ... porque me faz parecer melhor na frente dos outros
- ☐ ... porque deixa as reuniões sociais mais agradáveis

- ☐ ... porque é orgânico
- ☐ ... porque estou faminto
- ☐ ... porque eu curto
- ☐ ... porque me atrai aparentemente, por exemplo cores atraentes
- ☐ ... para compensar
- ☐ ... porque eu estou triste
- ☐ ... porque é mais conveniente
- ☐ ... porque eu tenho vontade de comer isso
- ☐ ... porque me mantém em forma exemplo, vigoroso, dinâmico
- ☐ ... porque tem pouca gordura
- ☐ ... porque é rápido de preparar
- ☐ ... porque não contém substâncias perigosas como agrotóxicos, poluentes, antibióticos
- ☐ ... porque estou frustrado
- ☐ ... porque é fácil de preparar
- ☐ ... porque me dou ao luxo
- ☐ ... porque eu me sinto sozinho
- ☐ ... porque é natural (exemplo, não geneticamente modificado)
- ☐ ... porque está em promoção
- ☐ ... porque eu cresci comendo isso
- ☐ ... porque eu devo comer
- ☐ ... porque eu posso desfrutar o momento com outras pessoas
- ☐ ... porque eu gosto
- ☐ ... porque os outros gostam
- ☐ ... porque estou habituado
- ☐ ... porque seria falta de educação não comer isso
- ☐ ... porque eu preciso de energia
- ☐ ... porque tem baixas calorias
- ☐ ... porque me sacia de forma agradável
- ☐ ... porque é moda
- ☐ ... porque é sociável
- ☐ ... porque é a única escolha
- ☐ ... porque me é familiar

- ☐ ... para manter uma dieta balanceada
- ☐ ... porque é saudável
- ☐ ... porque a apresentação é atraente, por exemplo a embalagem
- ☐ ... porque eu controlo meu peso
- ☐ ... porque faz parte de certas situações
- ☐ ... porque é saboroso
- ☐ ... porque normalmente eu como isso
- ☐ ... porque eu não quero gastar muito dinheiro
- ☐ ... para não desapontar alguém que está tentando me agradar
- ☐ ... porque eu quero comer todos os dias
- ☐ ... porque não faz parte das tradições como Natal, Ano Novo e ocasiões especiais
- ☐ ... porque não é caro

Quais as razões pelas quais você não come arroz? (Marque todas as respostas que correspondem)

- ☐ ... Eu não devo comer isso
- ☐ ... Comer isso me faz parecer antiquado
- ☐ ... Eu não quero me dar ao luxo
- ☐ ... Não é saudável
- ☐ ... O tamanho da porção não é adequado
- ☐ ... Eu não gosto do jeito que parece
- ☐ ... O preço está muito alto
- ☐ ... Eu nunca escolheria isso e eu gosto de comer a mesma comida todos os dias
- ☐ .. Eu comi recentemente e não quero comer a mesma comida com tanta frequência
- ☐ ... Essa comida me faz sentir triste, solitário e frustrado
- ☐ ... Não sustenta o suficiente
- ☐ ... Eu não gosto disso
- ☐ ... Não é conveniente
- ☐ ... Não é apropriado para a situação
- ☐ ... Eu normalmente não como isso

- ☐ .. Não é nada que eu queira comer agora
- ☐ ... Muito rico em calorias
- ☐ ... Não é um alimento que eu como perto de outras pessoas
- ☐ ... Não é orgânico
- ☐ ... Eu não acho que seja um lanche

Reasons for Protein foods Choices(CATA)

Você consome feijão?

- ○ Sim
- ○ Não

Quais as razões pelas quais você come feijão? (Marque todas as respostas que correspondem)

- ☐ ... porque é saboroso
- ☐ ... porque não contém substâncias perigosas como agrotóxicos, poluentes, antibióticos
- ☐ ... porque a apresentação é atraente, por exemplo a embalagem
- ☐ ... porque eu me sinto sozinho
- ☐ ... porque é fácil de preparar
- ☐ ... porque não é caro
- ☐ ... porque eu quero comer todos os dias
- ☐ ... porque seria falta de educação não comer isso
- ☐ ... porque é moda
- ☐ ... porque tem pouca gordura
- ☐ ... porque eu gosto
- ☐ ... porque é saudável
- ☐ ... porque é natural (exemplo, não geneticamente modificado)
- ☐ ... porque me sacia de forma agradável

- [ ] ... porque eu não quero gastar muito dinheiro
- [ ] ... porque não faz parte das tradições como Natal, Ano Novo e ocasiões especiais
- [ ] ... porque eu vejo propaganda em algum lugar ou na TV
- [ ] ... porque os outros gostam
- [ ] ... porque faz parte de certas situações
- [ ] ... porque estou frustrado
- [ ] ... porque está em promoção
- [ ] ... porque é sociável
- [ ] ... porque eu curto
- [ ] ... porque me faz parecer melhor na frente dos outros
- [ ] ... porque eu cresci comendo isso
- [ ] ... porque eu posso desfrutar o momento com outras pessoas
- [ ] ... porque eu devo comer
- [ ] ... porque é mais conveniente
- [ ] ... porque normalmente eu como isso
- [ ] ... porque tem baixas calorias
- [ ] ... porque eu controlo meu peso
- [ ] ... porque é a única escolha
- [ ] ... porque eu preciso de energia
- [ ] ... porque eu estou triste
- [ ] ... para não desapontar alguém que está tentando me agradar
- [ ] ... porque é rápido de preparar
- [ ] ... porque eu tenho vontade de comer isso
- [ ] ... para compensar
- [ ] ... porque me dou ao luxo
- [ ] ... porque me mantém em forma exemplo, vigoroso, dinâmico
- [ ] ... porque estou habituado
- [ ] ... porque deixa as reuniões sociais mais agradáveis
- [ ] ... porque me é familiar
- [ ] ... porque estou faminto
- [ ] ... porque me atrai aparentemente, por exemplo cores atraentes
- [ ] ... porque é orgânico

- [ ] ... para manter uma dieta balanceada

Quais as razões pelas quais você não come feijão? (Marque todas as respostas que correspondem)

- [ ] ... Não é saudável
- [ ] ... O tamanho da porção não é adequado
- [ ] .. Não é nada que eu queira comer agora
- [ ] ... Eu não quero me dar ao luxo
- [ ] ... Não é apropriado para a situação
- [ ] ... Não sustenta o suficiente
- [ ] ... Eu não gosto disso
- [ ] ... Eu não gosto do jeito que parece
- [ ] ... Eu nunca escolheria isso e eu gosto de comer a mesma comida todos os dias
- [ ] ... Comer isso me faz parecer antiquado
- [ ] ... Não é orgânico
- [ ] ... Muito rico em calorias
- [ ] ... Não é um alimento que eu como perto de outras pessoas
- [ ] ... Eu normalmente não como isso
- [ ] ... O preço está muito alto
- [ ] .. Eu comi recentemente e não quero comer a mesma comida com tanta frequência
- [ ] ... Essa comida me faz sentir triste, solitário e frustrado
- [ ] ... Eu não acho que seja um lanche
- [ ] ... Não é conveniente
- [ ] ... Eu não devo comer isso

Reasons for Dairy Food Choices(CATA)

Você consome leite?

- Sim
- Não

Quais as razões pelas quais você come leite? (Marque todas as respostas que correspondem)

- ... porque tem pouca gordura
- ... porque eu devo comer
- ... para não desapontar alguém que está tentando me agradar
- ... porque é sociável
- ... porque me sacia de forma agradável
- ... porque me dou ao luxo
- ... para compensar
- ... porque é orgânico
- ... porque eu posso desfrutar o momento com outras pessoas
- ... porque está em promoção
- ... porque eu curto
- ... porque estou frustrado
- ... porque os outros gostam
- ... para manter uma dieta balanceada
- ... porque deixa as reuniões sociais mais agradáveis
- ... porque estou faminto
- ... porque é mais conveniente
- ... porque eu controlo meu peso
- ... porque seria falta de educação não comer isso
- ... porque é saboroso
- ... porque eu estou triste
- ... porque me mantém em forma exemplo, vigoroso, dinâmico
- ... porque eu vejo propaganda em algum lugar ou na TV
- ... porque a apresentação é atraente, por exemplo a embalagem
- ... porque é fácil de preparar
- ... porque eu tenho vontade de comer isso

- ... porque me atrai aparentemente, por exemplo cores atraentes
- ... porque eu preciso de energia
- ... porque eu não quero gastar muito dinheiro
- ... porque é a única escolha
- ... porque não faz parte das tradições como Natal, Ano Novo e ocasiões especiais
- ... porque me é familiar
- ... porque faz parte de certas situações
- ... porque eu cresci comendo isso
- ... porque é rápido de preparar
- ... porque tem baixas calorias
- ... porque não contém substâncias perigosas como agrotóxicos, poluentes, antibióticos
- ... porque eu quero comer todos os dias
- ... porque não é caro
- ... porque é moda
- ... porque é saudável
- ... porque eu me sinto sozinho
- ... porque normalmente eu como isso
- ... porque eu gosto
- ... porque estou habituado
- ... porque me faz parecer melhor na frente dos outros
- ... porque é natural (exemplo, não geneticamente modificado)

Quais as razões pelas quais você não come leite? (Marque todas as respostas que correspondem)

- ... Não é um alimento que eu como perto de outras pessoas
- ... Eu não quero me dar ao luxo
- ... Não sustenta o suficiente
- ... Não é conveniente
- ... Não é saudável
- ... Eu nunca escolheria isso e eu gosto de comer a mesma comida todos os dias

- ☐ ... Eu não acho que seja um lanche
- ☐ ... Não é apropriado para a situação
- ☐ ... Eu não devo comer isso
- ☐ ... O tamanho da porção não é adequado
- ☐ ... Essa comida me faz sentir triste, solitário e frustrado
- ☐ ... Muito rico em calorias
- ☐ ... Eu não gosto do jeito que parece
- ☐ ... Não é orgânico
- ☐ ... Eu normalmente não como isso
- ☐ ... Comer isso me faz parecer antiquado
- ☐ .. Eu comi recentemente e não quero comer a mesma comida com tanta frequência
- ☐ ... O preço está muito alto
- ☐ ... Eu não gosto disso
- ☐ .. Não é nada que eu queira comer agora

Reasons for Fruit Choices(CATA)

Você consome banana?

- ○ Sim
- ○ Não

Quais as razões pelas quais você come banana? (Marque todas as respostas que correspondem)

- ☐ ... porque eu posso desfrutar o momento com outras pessoas
- ☐ ... para manter uma dieta balanceada
- ☐ ... porque não faz parte das tradições como Natal, Ano Novo e ocasiões especiais
- ☐ ... porque deixa as reuniões sociais mais agradáveis
- ☐ ... porque não é caro

- ☐ ... porque é fácil de preparar
- ☐ ... porque estou habituado
- ☐ ... porque estou frustrado
- ☐ ... porque eu cresci comendo isso
- ☐ ... porque eu quero comer todos os dias
- ☐ ... porque é rápido de preparar
- ☐ ... porque é saudável
- ☐ ... porque a apresentação é atraente, por exemplo a embalagem
- ☐ ... porque me atrai aparentemente, por exemplo cores atraentes
- ☐ ... porque normalmente eu como isso
- ☐ ... porque eu curto
- ☐ ... porque é saboroso
- ☐ ... porque eu me sinto sozinho
- ☐ ... porque é mais conveniente
- ☐ ... porque me faz parecer melhor na frente dos outros
- ☐ ... porque eu devo comer
- ☐ ... porque é moda
- ☐ ... porque me dou ao luxo
- ☐ ... porque eu preciso de energia
- ☐ ... porque me mantém em forma exemplo, vigoroso, dinâmico
- ☐ ... porque estou faminto
- ☐ ... porque não contém substâncias perigosas como agrotóxicos, poluentes, antibióticos
- ☐ ... porque os outros gostam
- ☐ ... porque tem pouca gordura
- ☐ ... porque está em promoção
- ☐ ... porque é sociável
- ☐ ... porque eu gosto
- ☐ ... porque eu vejo propaganda em algum lugar ou na TV
- ☐ ... porque seria falta de educação não comer isso
- ☐ ... porque tem baixas calorias
- ☐ ... porque é orgânico
- ☐ ... porque faz parte de certas situações

- ☐ ... porque eu tenho vontade de comer isso
- ☐ ... porque eu controlo meu peso
- ☐ ... para não desapontar alguém que está tentando me agradar
- ☐ ... porque eu estou triste
- ☐ ... porque me sacia de forma agradável
- ☐ ... porque é a única escolha
- ☐ ... porque eu não quero gastar muito dinheiro
- ☐ ... porque é natural (exemplo, não geneticamente modificado)
- ☐ ... para compensar
- ☐ ... porque me é familiar

Quais as razões pelas quais você não come banana? (Marque todas as respostas que correspondem)

- ☐ ... Eu nunca escolheria isso e eu gosto de comer a mesma comida todos os dias
- ☐ .. Não é nada que eu queira comer agora
- ☐ ... Não é conveniente
- ☐ ... Essa comida me faz sentir triste, solitário e frustrado
- ☐ ... Não é apropriado para a situação
- ☐ ... Não sustenta o suficiente
- ☐ .. Eu comi recentemente e não quero comer a mesma comida com tanta frequência
- ☐ ... Eu não gosto do jeito que parece
- ☐ ... Comer isso me faz parecer antiquado
- ☐ ... O tamanho da porção não é adequado
- ☐ ... Eu não gosto disso
- ☐ ... O preço está muito alto
- ☐ ... Eu não quero me dar ao luxo
- ☐ ... Eu não acho que seja um lanche
- ☐ ... Eu não devo comer isso
- ☐ ... Muito rico em calorias
- ☐ ... Não é um alimento que eu como perto de outras pessoas

- ☐ ... Não é saudável
- ☐ ... Não é orgânico
- ☐ ... Eu normalmente não como isso

Reasons for Sweet food Choices(CATA)

Você consome brigadeiro?

- ○ Sim
- ○ Não

Quais as razões pelas quais você come brigadeiro? (Marque todas as respostas que correspondem)

- ☐ ... porque me mantém em forma exemplo, vigoroso, dinâmico
- ☐ ... porque faz parte de certas situações
- ☐ ... porque estou habituado
- ☐ ... porque a apresentação é atraente, por exemplo a embalagem
- ☐ ... porque os outros gostam
- ☐ ... porque eu não quero gastar muito dinheiro
- ☐ ... porque deixa as reuniões sociais mais agradáveis
- ☐ ... porque é moda
- ☐ ... porque estou faminto
- ☐ ... porque eu curto
- ☐ ... para compensar
- ☐ ... porque estou frustrado
- ☐ ... porque eu vejo propaganda em algum lugar ou na TV
- ☐ ... porque eu posso desfrutar o momento com outras pessoas
- ☐ ... porque eu controlo meu peso
- ☐ ... porque é mais conveniente
- ☐ ... porque tem pouca gordura

- ☐ ... porque é fácil de preparar
- ☐ ... porque é orgânico
- ☐ ... porque eu quero comer todos os dias
- ☐ ... porque é rápido de preparar
- ☐ ... porque normalmente eu como isso
- ☐ ... porque não é caro
- ☐ ... porque tem baixas calorias
- ☐ ... porque é saboroso
- ☐ ... porque é saudável
- ☐ ... porque me dou ao luxo
- ☐ ... porque eu me sinto sozinho
- ☐ ... porque me faz parecer melhor na frente dos outros
- ☐ ... porque me sacia de forma agradável
- ☐ ... porque eu devo comer
- ☐ ... porque não contém substâncias perigosas como agrotóxicos, poluentes, antibióticos
- ☐ ... porque me é familiar
- ☐ ... porque é sociável
- ☐ ... porque não faz parte das tradições como Natal, Ano Novo e ocasiões especiais
- ☐ ... porque seria falta de educação não comer isso
- ☐ ... porque está em promoção
- ☐ ... porque eu preciso de energia
- ☐ ... porque eu estou triste
- ☐ ... porque é natural (exemplo, não geneticamente modificado)
- ☐ ... porque me atrai aparentemente, por exemplo cores atraentes
- ☐ ... porque eu tenho vontade de comer isso
- ☐ ... porque é a única escolha
- ☐ ... para não desapontar alguém que está tentando me agradar
- ☐ ... para manter uma dieta balanceada
- ☐ ... porque eu cresci comendo isso
- ☐ ... porque eu gosto

Quais as razões pelas quais você não come brigadeiro? (Marque todas as respostas que correspondem)

- ☐ ... Eu não gosto disso
- ☐ ... O preço está muito alto
- ☐ ... Eu não quero me dar ao luxo
- ☐ ... Não é um alimento que eu como perto de outras pessoas
- ☐ ... Eu não gosto do jeito que parece
- ☐ .. Eu comi recentemente e não quero comer a mesma comida com tanta frequência
- ☐ ... Não sustenta o suficiente
- ☐ ... O tamanho da porção não é adequado
- ☐ ... Eu não acho que seja um lanche
- ☐ ... Não é conveniente
- ☐ ... Não é apropriado para a situação
- ☐ ... Não é orgânico
- ☐ ... Eu normalmente não como isso
- ☐ ... Comer isso me faz parecer antiquado
- ☐ ... Não é saudável
- ☐ ... Eu nunca escolheria isso e eu gosto de comer a mesma comida todos os dias
- ☐ ... Muito rico em calorias
- ☐ .. Não é nada que eu queira comer agora
- ☐ ... Essa comida me faz sentir triste, solitário e frustrado
- ☐ ... Eu não devo comer isso

JAR & Liking

O quanto você achou essa pesquisa curta ou longa?

- ○ Curta demais
- ○ Muito curta
- ○ Um pouco curta

- Ideal
- Um pouco longa
- Muito longa
- Longa demais

Como você se sentiu ao término dessa pesquisa?

- Eu adorei responder
- Eu gostei muito de responder
- Indiferente
- Eu não gostei muito de responder
- Eu odiei responder

Reasons for Starchy food choices(Full CATA)

Você come arroz?

- Sim
- Não

Você come arroz porque está habituado?

- Sim
- Não

Você come arroz porque é saudável?

- Sim
- Não

Você come arroz porque é rápido de preparar?

- Sim
- Não

Você come arroz porque é saboroso?

- Sim
- Não

Você come arroz porque se dá ao luxo de comer?

- Sim
- Não

Você come arroz porque é natural (exemplo, não geneticamente modificado)?

- Sim
- Não

Você come arroz porque não quer gastar muito dinheiro?

- Sim
- Não

Você come arroz porque tem baixas calorias?

- Sim
- Não

Você come arroz porque está frustrado?

- Sim
- Não

Você come arroz porque te faz parecer melhor na frente dos outros?

- Sim
- Não

Você come arroz porque seria falta de educação não comer?

- Sim
- Não

Você come arroz porque a apresentação é atraente, por exemplo a embalagem?

- Sim
- Não

Você come arroz para poder desfrutar o momento com outras pessoas?

- Sim
- Não

Você come arroz porque é orgânico?

- Sim
- Não

Você come arroz porque cresceu comendo isso?

- Sim
- Não

Você come arroz porque curte?

- Sim
- Não

Você come arroz porque está faminto?

- Sim
- Não

Você come arroz porque faz parte do hábito?

- Sim
- Não

Você come arroz porque tem vontade de comer isso?

- Sim
- Não

Você come arroz porque é fácil de preparar?

- Sim
- Não

Você come arroz porque fica triste?

- Sim
- Não

Você come arroz porque tem pouca gordura?

- Sim
- Não

Você come arroz porque vê propaganda em algum lugar ou na TV?

- Sim
- Não

Você come arroz porque deixa as reuniões sociais mais agradáveis?

- Sim
- Não

Você come arroz porque deve comer?

- Sim
- Não

Você come arroz porque os outros gostam?

- Sim
- Não

Você come arroz porque se sente sozinho?

- Sim
- Não

Você come arroz porque controla seu peso?

- Sim
- Não

Você come arroz porque não contém substâncias perigosas como agrotóxicos, poluentes e ou antibióticos?

- Sim
- Não

Você come arroz porque é mais conveniente?

- Sim
- Não

Você come arroz porque precisa de energia?

- Sim
- Não

Você come arroz porque lhe é familiar?

- Sim
- Não

Você come arroz porque gosta?

- Sim
- Não

Você come arroz porque te sacia de forma agradável?

- Sim
- Não

Você come arroz para compensar?

- Sim
- Não

Você come arroz porque não faz parte das tradições como Natal, Ano Novo ou outras ocasiões especiais?

- Sim
- Não

Você come arroz porque faz parte de certas situações?

- Sim
- Não

Você come arroz para manter uma dieta balanceada?

- Sim
- Não

Você come arroz porque é sociável?

- Sim
- Não

Você come arroz porque não é caro?

- Sim
- Não

Você come arroz porque te atrai aparentemente como por exemplo as cores atraentes?

- Sim
- Não

Você come arroz para não desapontar alguém que está tentando te agradar?

- Sim
- Não

Você come arroz porque é moda?

- Sim
- Não

Você come arroz porque está em promoção?

- Sim
- Não

Você come arroz porque te mantém em forma, vigoroso, dinâmico?

- Sim
- Não

Você come arroz porque é a única escolha?

- Sim
- Não

Você come arroz porque quer comer todos os dias?

- Sim
- Não

Você não come arroz porque pensa que não é um lanche?

- Sim
- Não

Você não come arroz porque não gosta?

- Sim
- Não

Você não come arroz porque normalmente não come?

- Sim
- Não

Você não come arroz porque o tamanho da porção não é adequado?

- Sim
- Não

Você não come arroz porque não sustenta o suficiente?

- Sim
- Não

Você não come arroz porque não é saudável?

- Sim
- Não

Você não come arroz porque não é conveniente?

- Sim
- Não

Você não come arroz porque não quer se dar ao luxo?

- Sim
- Não

Você não come arroz porque não é apropriado para a situação?

- Sim
- Não

Você não come arroz porque não é um alimento que se come perto de outras pessoas?

- Sim
- Não

Você não come arroz porque o preço está muito alto?

- Sim
- Não

Você não come arroz porque não gosta do jeito que parece?

- Sim
- Não

Você não come arroz porque é muito rico em calorias?

- Sim
- Não

Você não come arroz porque não deve comer isso?

- Sim
- Não

Você não come arroz porque comer isso te faz parecer antiquado?

- Sim
- Não

Você não come arroz porque não é algo que queira comer agora?

- Sim
- Não

Você não come arroz porque comeu recentemente e não quer comer a mesma comida com tanta frequência?

- Sim
- Não

Você não come arroz porque nunca escolheria isso, já que gosta de comer a mesma comida todo dia?

- Sim
- Não

Você não come arroz porque essa comida te faz se sentir triste, solitário e frustrado?

- Sim
- Não

Você não come arroz porque não é orgânico

- Sim
- Não

Reasons for Protein foods Choices(Full CATA)

Você come feijão?

- Sim
- Não

Você come feijão porque está habituado?

- Sim
- Não

Você come feijão porque é saudável?

- Sim
- Não

Você come feijão porque é rápido de preparar?

- Sim
- Não

Você come feijão porque é saboroso?

- Sim
- Não

Você come feijão porque se dá ao luxo de comer?

- Sim
- Não

Você come feijão porque é natural (exemplo, não geneticamente modificado)?

- Sim
- Não

Você come feijão porque não quer gastar muito dinheiro?

- Sim
- Não

Você come feijão porque tem baixas calorias?

- Sim
- Não

Você come feijão porque está frustrado?

- Sim
- Não

Você come feijão porque te faz parecer melhor na frente dos outros?

- Sim
- Não

Você come feijão porque seria falta de educação não comer?

- Sim
- Não

Você come feijão porque a apresentação é atraente, por exemplo a embalagem?

- Sim
- Não

Você come feijão para poder desfrutar o momento com outras pessoas?

- Sim
- Não

Você come feijão porque é orgânico?

- Sim
- Não

Você come feijão porque cresceu comendo isso?

- Sim
- Não

Você come feijão porque curte?

- Sim
- Não

Você come feijão porque está faminto?

- Sim
- Não

Você come feijão porque faz parte do hábito?

- Sim
- Não

Você come feijão porque tem vontade de comer isso?

- Sim
- Não

Você come feijão porque é fácil de preparar?

- Sim
- Não

Você come feijão porque fica triste?

- Sim
- Não

Você come feijão porque tem pouca gordura?

- Sim
- Não

Você come feijão porque vê propaganda em algum lugar ou na TV?

- Sim
- Não

Você come feijão porque deixa as reuniões sociais mais agradáveis?

- Sim
- Não

Você come feijão porque deve comer?

- Sim
- Não

Você come feijão porque os outros gostam?

- Sim
- Não

Você come feijão porque se sente sozinho?

- Sim
- Não

Você come feijão porque controla seu peso?

- Sim
- Não

Você come feijão porque não contém substâncias perigosas como agrotóxicos, poluentes e ou antibióticos?

- Sim
- Não

Você come feijão porque é mais conveniente?

- Sim
- Não

Você come feijão porque precisa de energia?

- Sim
- Não

Você come feijão porque lhe é familiar?

- Sim
- Não

Você come feijão porque gosta?

- Sim
- Não

Você come feijão porque te sacia de forma agradável?

- Sim
- Não

Você come feijão para compensar?

- Sim
- Não

Você come feijão porque não faz parte das tradições como Natal, Ano Novo ou outras ocasiões especiais?

- Sim
- Não

Você come feijão porque faz parte de certas situações?

- Sim
- Não

Você come feijão para manter uma dieta balanceada?

- Sim
- Não

Você come feijão porque é sociável?

- Sim
- Não

Você come feijão porque não é caro?

- Sim
- Não

Você come feijão porque te atrai aparentemente como por exemplo as cores atraentes?

- Sim
- Não

Você come feijão para não desapontar alguém que está tentando te agradar?

- Sim
- Não

Você come feijão porque é moda?

- Sim
- Não

Você come feijão porque está em promoção?

- Sim
- Não

Você come feijão porque te mantém em forma, vigoroso, dinâmico?

- Sim
- Não

Você come feijão porque é a única escolha?

- Sim
- Não

Você come feijão porque quer comer todos os dias?

- Sim
- Não

Você não come feijão porque pensa que não é um lanche?

- Sim
- Não

Você não come feijão porque não gosta?

- Sim
- Não

Você não come feijão porque normalmente não come?

- Sim
- Não

Você não come feijão porque o tamanho da porção não é adequado?

- Sim
- Não

Você não come feijão porque não sustenta o suficiente?

- Sim
- Não

Você não come feijão porque não é saudável?

- Sim
- Não

Você não come feijão porque não é conveniente?

- Sim
- Não

Você não come feijão porque não quer se dar ao luxo?

- Sim
- Não

Você não come feijão porque não é apropriado para a situação?

- Sim
- Não

Você não come feijão porque não é um alimento que se come perto de outras pessoas?

- Sim
- Não

Você não come feijão porque o preço está muito alto?

- Sim
- Não

Você não come feijão porque não gosta do jeito que parece?

- Sim
- Não

Você não come feijão porque é muito rico em calorias?

- Sim
- Não

Você não come feijão porque não deve comer isso?

- Sim
- Não

Você não come feijão porque comer isso te faz parecer antiquado?

- Sim
- Não

Você não come feijão porque não é algo que queira comer agora?

- Sim
- Não

Você não come feijão porque comeu recentemente e não quer comer a mesma comida com tanta frequência?

- Sim
- Não

Você não come feijão porque nunca escolheria isso, já que gosta de comer a mesma comida todo dia?

- Sim
- Não

Você não come feijão porque essa comida te faz se sentir triste, solitário e frustrado?

- Sim
- Não

Você não come feijão porque não é orgânico

- Sim
- Não

Reasons for Dairy Food Choices(Full CATA)

Você come leite?

- Sim
- Não

Você come leite porque está habituado?

- Sim
- Não

Você come leite porque é saudável?

- Sim
- Não

Você come leite porque é rápido de preparar?

- Sim
- Não

Você come leite porque é saboroso?

- Sim
- Não

Você come leite porque se dá ao luxo de comer?

- Sim
- Não

Você come leite porque é natural (exemplo, não geneticamente modificado)?

- Sim
- Não

Você come leite porque não quer gastar muito dinheiro?

- Sim
- Não

Você come leite porque tem baixas calorias?

- Sim
- Não

Você come leite porque está frustrado?

- Sim
- Não

Você come leite porque te faz parecer melhor na frente dos outros?

- Sim
- Não

Você come leite porque seria falta de educação não comer?

- Sim
- Não

Você come leite porque a apresentação é atraente, por exemplo a embalagem?

- Sim
- Não

Você come leite para poder desfrutar o momento com outras pessoas?

- Sim
- Não

Você come leite porque é orgânico?

- Sim
- Não

Você come leite porque cresceu comendo isso?

- Sim
- Não

Você come leite porque curte?

- Sim
- Não

Você come leite porque está faminto?

- Sim
- Não

Você come leite porque faz parte do hábito?

- Sim
- Não

Você come leite porque tem vontade de comer isso?

- Sim
- Não

Você come leite porque é fácil de preparar?

- Sim
- Não

Você come leite porque fica triste?

- Sim
- Não

Você come leite porque tem pouca gordura?

- Sim
- Não

Você come leite porque vê propaganda em algum lugar ou na TV?

- Sim
- Não

Você come leite porque deixa as reuniões sociais mais agradáveis?

- Sim
- Não

Você come leite porque deve comer?

- Sim
- Não

Você come leite porque os outros gostam?

- Sim
- Não

Você come leite porque se sente sozinho?

- Sim
- Não

Você come leite porque controla seu peso?

- Sim
- Não

Você come leite porque não contém substâncias perigosas como agrotóxicos, poluentes e ou antibióticos?

- Sim
- Não

Você come leite porque é mais conveniente?

- Sim
- Não

Você come leite porque precisa de energia?

- Sim
- Não

Você come leite porque lhe é familiar?

- Sim
- Não

Você come leite porque gosta?

- Sim
- Não

Você come leite porque te sacia de forma agradável?

- Sim
- Não

Você come leite para compensar?

- Sim
- Não

Você come leite porque não faz parte das tradições como Natal, Ano Novo ou outras ocasiões especiais?

- Sim
- Não

Você come leite porque faz parte de certas situações?

- Sim
- Não

Você come leite para manter uma dieta balanceada?

- Sim
- Não

Você come leite porque é sociável?

- Sim
- Não

Você come leite porque não é caro?

- Sim
- Não

Você come leite porque te atrai aparentemente como por exemplo as cores atraentes?

- Sim
- Não

Você come leite para não desapontar alguém que está tentando te agradar?

- Sim
- Não

Você come leite porque é moda?

- Sim
- Não

Você come leite porque está em promoção?

- Sim
- Não

Você come leite porque te mantém em forma, vigoroso, dinâmico?

- Sim
- Não

Você come leite porque é a única escolha?

- Sim
- Não

Você come leite porque quer comer todos os dias?

- Sim
- Não

Você não come leite porque pensa que não é um lanche?

- Sim
- Não

Você não come leite porque não gosta?

- Sim
- Não

Você não come leite porque normalmente não come?

- Sim
- Não

Você não come leite porque o tamanho da porção não é adequado?

- Sim
- Não

Você não come leite porque não sustenta o suficiente?

- Sim
- Não

Você não come leite porque não é saudável?

- Sim
- Não

Você não come leite porque não é conveniente?

- Sim
- Não

Você não come leite porque não quer se dar ao luxo?

- Sim
- Não

Você não come leite porque não é apropriado para a situação?

- Sim
- Não

Você não come leite porque não é um alimento que se come perto de outras pessoas?

- Sim
- Não

Você não come leite porque o preço está muito alto?

- Sim
- Não

Você não come leite porque não gosta do jeito que parece?

- Sim
- Não

Você não come leite porque é muito rico em calorias?

- Sim
- Não

Você não come leite porque não deve comer isso?

- Sim
- Não

Você não come leite porque comer isso te faz parecer antiquado?

- Sim
- Não

Você não come leite porque não é algo que queira comer agora?

- Sim
- Não

Você não come leite porque comeu recentemente e não quer comer a mesma comida com tanta frequência?

- Sim
- Não

Você não come leite porque nunca escolheria isso, já que gosta de comer a mesma comida todo dia?

- Sim
- Não

Você não come leite porque essa comida te faz se sentir triste, solitário e frustrado?

- Sim
- Não

Você não come leite porque não é orgânico

- Sim
- Não

Reasons for Fruit Choices(Full CATA)

Você come banana?

- Sim
- Não

Você come banana porque está habituado?

- Sim
- Não

Você come banana porque é saudável?

- Sim
- Não

Você come banana porque é rápido de preparar?

- Sim
- Não

Você come banana porque é saboroso?

- Sim
- Não

Você come banana porque se dá ao luxo de comer?

- Sim
- Não

Você come banana porque é natural (exemplo, não geneticamente modificado)?

- Sim
- Não

Você come banana porque não quer gastar muito dinheiro?

- Sim
- Não

Você come banana porque tem baixas calorias?

- Sim
- Não

Você come banana porque está frustrado?

- Sim
- Não

Você come banana porque te faz parecer melhor na frente dos outros?

- Sim
- Não

Você come banana porque seria falta de educação não comer?

- Sim
- Não

Você come banana porque a apresentação é atraente, por exemplo a embalagem?

- Sim
- Não

Você come banana para poder desfrutar o momento com outras pessoas?

- Sim
- Não

Você come banana porque é orgânico?

- Sim
- Não

Você come banana porque cresceu comendo isso?

- Sim
- Não

Você come banana porque curte?

- Sim
- Não

Você come banana porque está faminto?

- Sim
- Não

Você come banana porque faz parte do hábito?

- Sim
- Não

Você come banana porque tem vontade de comer isso?

- Sim
- Não

Você come banana porque é fácil de preparar?

- Sim
- Não

Você come banana porque fica triste?

- Sim
- Não

Você come banana porque tem pouca gordura?

- Sim
- Não

Você come banana porque vê propaganda em algum lugar ou na TV?

- Sim
- Não

Você come banana porque deixa as reuniões sociais mais agradáveis?

- Sim
- Não

Você come banana porque deve comer?

- Sim
- Não

Você come banana porque os outros gostam?

- Sim
- Não

Você come banana porque se sente sozinho?

- Sim
- Não

Você come banana porque controla seu peso?

- Sim
- Não

Você come banana porque não contém substâncias perigosas como agrotóxicos, poluentes e ou antibióticos?

- Sim
- Não

Você come banana porque é mais conveniente?

- Sim
- Não

Você come banana porque precisa de energia?

- Sim
- Não

Você come banana porque lhe é familiar?

- Sim
- Não

Você come banana porque gosta?

- Sim
- Não

Você come banana porque te sacia de forma agradável?

- Sim
- Não

Você come banana para compensar?

- Sim
- Não

Você come banana porque não faz parte das tradições como Natal, Ano Novo ou outras ocasiões especiais?

- Sim
- Não

Você come banana porque faz parte de certas situações?

- Sim
- Não

Você come banana para manter uma dieta balanceada?

- Sim
- Não

Você come banana porque é sociável?

- Sim
- Não

Você come banana porque não é caro?

- Sim
- Não

Você come banana porque te atrai aparentemente como por exemplo as cores atraentes?

- Sim
- Não

Você come banana para não desapontar alguém que está tentando te agradar?

- Sim
- Não

Você come banana porque é moda?

- Sim
- Não

Você come banana porque está em promoção?

- Sim
- Não

Você come banana porque te mantém em forma, vigoroso, dinâmico?

- Sim
- Não

Você come banana porque é a única escolha?

- Sim
- Não

Você come banana porque quer comer todos os dias?

- Sim
- Não

Você não come banana porque pensa que não é um lanche?

- Sim
- Não

Você não come banana porque não gosta?

- Sim
- Não

Você não come banana porque normalmente não come?

- Sim
- Não

Você não come banana porque o tamanho da porção não é adequado?

- Sim
- Não

Você não come banana porque não sustenta o suficiente?

- Sim
- Não

Você não come banana porque não é saudável?

- Sim
- Não

Você não come banana porque não é conveniente?

- Sim
- Não

Você não come banana porque não quer se dar ao luxo?

- Sim
- Não

Você não come banana porque não é apropriado para a situação?

- Sim
- Não

Você não come banana porque não é um alimento que se come perto de outras pessoas?

- Sim
- Não

Você não come banana porque o preço está muito alto?

- Sim
- Não

Você não come banana porque não gosta do jeito que parece?

- Sim
- Não

Você não come banana porque é muito rico em calorias?

- Sim
- Não

Você não come banana porque não deve comer isso?

- Sim
- Não

Você não come banana porque comer isso te faz parecer antiquado?

- Sim
- Não

Você não come banana porque não é algo que queira comer agora?

- Sim
- Não

Você não come banana porque comeu recentemente e não quer comer a mesma comida com tanta frequência?

- Sim
- Não

Você não come banana porque nunca escolheria isso, já que gosta de comer a mesma comida todo dia?

- Sim
- Não

Você não come banana porque essa comida te faz se sentir triste, solitário e frustrado?

- Sim
- Não

Você não come banana porque não é orgânico

- Sim
- Não

Reasons for Sweet food Choices(Full CATA)

Você come brigadeiro?

- Sim
- Não

Você come brigadeiro porque está habituado?

- Sim
- Não

Você come brigadeiro porque é saudável?

- Sim
- Não

Você come brigadeiro porque é rápido de preparar?

- Sim
- Não

Você come brigadeiro porque é saboroso?

- Sim
- Não

Você come brigadeiro porque se dá ao luxo de comer?

- Sim
- Não

Você come brigadeiro porque é natural (exemplo, não geneticamente modificado)?

- Sim
- Não

Você come brigadeiro porque não quer gastar muito dinheiro?

- Sim
- Não

Você come brigadeiro porque tem baixas calorias?

- Sim
- Não

Você come brigadeiro porque está frustrado?

- Sim
- Não

Você come brigadeiro porque te faz parecer melhor na frente dos outros?

- Sim
- Não

Você come brigadeiro porque seria falta de educação não comer?

- Sim
- Não

Você come brigadeiro porque a apresentação é atraente, por exemplo a embalagem?

- Sim
- Não

Você come brigadeiro para poder desfrutar o momento com outras pessoas?

- Sim
- Não

Você come brigadeiro porque é orgânico?

- Sim
- Não

Você come brigadeiro porque cresceu comendo isso?

- Sim
- Não

Você come brigadeiro porque curte?

- Sim
- Não

Você come brigadeiro porque está faminto?

- Sim
- Não

Você come brigadeiro porque faz parte do hábito?

- Sim
- Não

Você come brigadeiro porque tem vontade de comer isso?

- Sim
- Não

Você come brigadeiro porque é fácil de preparar?

- Sim
- Não

Você come brigadeiro porque fica triste?

- Sim
- Não

Você come brigadeiro porque tem pouca gordura?

- Sim
- Não

Você come brigadeiro porque vê propaganda em algum lugar ou na TV?

- Sim
- Não

Você come brigadeiro porque deixa as reuniões sociais mais agradáveis?

- Sim
- Não

Você come brigadeiro porque deve comer?

- Sim
- Não

Você come brigadeiro porque os outros gostam?

- Sim
- Não

Você come brigadeiro porque se sente sozinho?

- Sim
- Não

Você come brigadeiro porque controla seu peso?

- Sim
- Não

Você come brigadeiro porque não contém substâncias perigosas como agrotóxicos, poluentes e ou antibióticos?

- Sim
- Não

Você come brigadeiro porque é mais conveniente?

- Sim
- Não

Você come brigadeiro porque precisa de energia?

- Sim
- Não

Você come brigadeiro porque lhe é familiar?

- Sim
- Não

Você come brigadeiro porque gosta?

- Sim
- Não

Você come brigadeiro porque te sacia de forma agradável?

- Sim
- Não

Você come brigadeiro para compensar?

- Sim
- Não

Você come brigadeiro porque não faz parte das tradições como Natal, Ano Novo ou outras ocasiões especiais?

- Sim
- Não

Você come brigadeiro porque faz parte de certas situações?

- Sim
- Não

Você come brigadeiro para manter uma dieta balanceada?

- Sim
- Não

Você come brigadeiro porque é sociável?

- Sim
- Não

Você come brigadeiro porque não é caro?

- Sim
- Não

Você come brigadeiro porque te atrai aparentemente como por exemplo as cores atraentes?

- Sim
- Não

Você come brigadeiro para não desapontar alguém que está tentando te agradar?

- Sim
- Não

Você come brigadeiro porque é moda?

- Sim
- Não

Você come brigadeiro porque está em promoção?

- Sim
- Não

Você come brigadeiro porque te mantém em forma, vigoroso, dinâmico?

- Sim
- Não

Você come brigadeiro porque é a única escolha?

- Sim
- Não

Você come brigadeiro porque quer comer todos os dias?

- Sim
- Não

Você não come brigadeiro porque pensa que não é um lanche?

- Sim
- Não

Você não come brigadeiro porque não gosta?

- Sim
- Não

Você não come brigadeiro porque normalmente não come?

- Sim
- Não

Você não come brigadeiro porque o tamanho da porção não é adequado?

- Sim
- Não

Você não come brigadeiro porque não sustenta o suficiente?

- Sim
- Não

Você não come brigadeiro porque não é saudável?

- Sim
- Não

Você não come brigadeiro porque não é conveniente?

- Sim
- Não

Você não come brigadeiro porque não quer se dar ao luxo?

- Sim
- Não

Você não come brigadeiro porque não é apropriado para a situação?

- Sim
- Não

Você não come brigadeiro porque não é um alimento que se come perto de outras pessoas?

- Sim
- Não

Você não come brigadeiro porque o preço está muito alto?

- Sim
- Não

Você não come brigadeiro porque não gosta do jeito que parece?

- Sim
- Não

Você não come brigadeiro porque é muito rico em calorias?

- Sim
- Não

Você não come brigadeiro porque não deve comer isso?

- Sim
- Não

Você não come brigadeiro porque comer isso te faz parecer antiquado?

- Sim
- Não

Você não come brigadeiro porque não é algo que queira comer agora?

- Sim
- Não

Você não come brigadeiro porque comeu recentemente e não quer comer a mesma comida com tanta frequência?

- Sim
- Não

Você não come brigadeiro porque nunca escolheria isso, já que gosta de comer a mesma comida todo dia?

- Sim
- Não

Você não come brigadeiro porque essa comida te faz se sentir triste, solitário e frustrado?

- Sim
- Não

Você não come brigadeiro porque não é orgânico

- Sim
- Não

Reasons for Starchy food choices(RATA)

Você consome arroz?

- Sim
- Não

Quais as razões pelas quais você come arroz? (Marque todas as respostas que correspondem)

- ... porque não é caro
- ... para não desapontar alguém que está tentando me agradar
- ... porque tem pouca gordura
- ... porque eu posso desfrutar o momento com outras pessoas
- ... porque me sacia de forma agradável
- ... porque é mais conveniente
- ... porque seria falta de educação não comer isso
- ... porque é saboroso
- ... porque eu não quero gastar muito dinheiro
- ... porque eu me sinto sozinho
- ... porque faz parte de certas situações
- ... porque é rápido de preparar
- ... porque eu controlo meu peso
- ... porque estou faminto
- ... porque me atrai aparentemente, por exemplo cores atraentes

- ☐ ... porque estou frustrado
- ☐ ... porque eu preciso de energia
- ☐ ... porque a apresentação é atraente, por exemplo a embalagem
- ☐ ... porque é orgânico
- ☐ ... porque não contém substâncias perigosas como agrotóxicos, poluentes, antibióticos
- ☐ ... porque eu estou triste
- ☐ ... porque está em promoção
- ☐ ... porque me dou ao luxo
- ☐ ... porque é moda
- ☐ ... porque eu devo comer
- ☐ ... porque me mantém em forma exemplo, vigoroso, dinâmico
- ☐ ... porque tem baixas calorias
- ☐ ... porque é sociável
- ☐ ... porque estou habituado
- ☐ ... porque eu curto
- ☐ ... porque eu vejo propaganda em algum lugar ou vejo na TV
- ☐ ... porque eu cresci comendo isso
- ☐ ... porque é fácil de preparar
- ☐ ... porque os outros gostam
- ☐ ... porque me faz parecer melhor na frente dos outros
- ☐ ... porque me é familiar
- ☐ ... porque eu quero comer todos os dias
- ☐ ... porque não faz parte das tradições como Natal, Ano Novo e ocasiões especiais
- ☐ ... porque deixa as reuniões sociais mais agradáveis
- ☐ ... porque é natural (exemplo, não geneticamente modificado)
- ☐ ... porque eu tenho vontade de comer isso
- ☐ ... porque é saudável
- ☐ ... para manter uma dieta balanceada
- ☐ ... porque é a única escolha
- ☐ ... porque eu gosto
- ☐ ... para compensar
- ☐ ... porque normalmente eu como isso

Você selecionou "... porque estou habituado "
O quanto isso é importante para você?

- Extremamente importante
- Muito importante
- Moderadamente importante
- Levemente importante
- Nada importante

Você selecionou "... porque é saudável "
O quanto isso é importante para você?

- Extremamente importante
- Muito importante
- Moderadamente importante
- Levemente importante
- Nada importante

Você selecionou "... porque é rápido de preparar "
O quanto isso é importante para você?

- Extremamente importante
- Muito importante
- Moderadamente importante
- Levemente importante
- Nada importante

Você selecionou "... porque é saboroso "
O quanto isso é importante para você?

- Extremamente importante

- Muito importante
- Moderadamente importante
- Levemente importante
- Nada importante

Você selecionou "... porque me dou ao luxo "
O quanto isso é importante para você?

- Extremamente importante
- Muito importante
- Moderadamente importante
- Levemente importante
- Nada importante

Você selecionou "... porque é natural (exemplo, não geneticamente modificado) "
O quanto isso é importante para você?

- Extremamente importante
- Muito importante
- Moderadamente importante
- Levemente importante
- Nada importante

Você selecionou "... porque eu não quero gastar muito dinheiro "
O quanto isso é importante para você?

- Extremamente importante
- Muito importante
- Moderadamente importante
- Levemente importante
- Nada importante

Você selecionou "... porque tem baixas calorias "
O quanto isso é importante para você?

- Extremamente importante
- Muito importante
- Moderadamente importante
- Levemente importante
- Nada importante

Você selecionou "... porque estou frustrado "
O quanto isso é importante para você?

- Extremamente importante
- Muito importante
- Moderadamente importante
- Levemente importante
- Nada importante

Você selecionou "... porque me faz parecer melhor na frente dos outros "
O quanto isso é importante para você?

- Extremamente importante
- Muito importante
- Moderadamente importante
- Levemente importante
- Nada importante

Você selecionou "... porque seria falta de educação não comer isso "
O quanto isso é importante para você?

- Extremamente importante

- Muito importante
- Moderadamente importante
- Levemente importante
- Nada importante

Você selecionou "... porque a apresentação é atraente, por exemplo a embalagem "

O quanto isso é importante para você?

- Extremamente importante
- Muito importante
- Moderadamente importante
- Levemente importante
- Nada importante

Você selecionou "... porque eu posso desfrutar o momento com outras pessoas "
O quanto isso é importante para você?

- Extremamente importante
- Muito importante
- Moderadamente importante
- Levemente importante
- Nada importante

Você selecionou "... porque é orgânico "
O quanto isso é importante para você?

- Extremamente importante
- Muito importante
- Moderadamente importante
- Levemente importante
- Nada importante

Você selecionou "... porque eu cresci comendo isso "
O quanto isso é importante para você?

- Extremamente importante
- Muito importante
- Moderadamente importante
- Levemente importante
- Nada importante

Você selecionou "... porque eu curto "
O quanto isso é importante para você?

- Extremamente importante
- Muito importante
- Moderadamente importante
- Levemente importante
- Nada importante

Você selecionou "... porque estou faminto "
O quanto isso é importante para você?

- Extremamente importante
- Muito importante
- Moderadamente importante
- Levemente importante
- Nada importante

Você selecionou "... porque normalmente eu como isso "
O quanto isso é importante para você?

- Extremamente importante
- Muito importante
- Moderadamente importante
- Levemente importante
- Nada importante

Você selecionou "... porque eu tenho vontade de comer isso "
O quanto isso é importante para você?

- Extremamente importante
- Muito importante
- Moderadamente importante
- Levemente importante
- Nada importante

Você selecionou "... porque é fácil de preparar "
O quanto isso é importante para você?

- Extremamente importante
- Muito importante
- Moderadamente importante
- Levemente importante
- Nada importante

Você selecionou "... porque eu estou triste "
O quanto isso é importante para você?

- Extremamente importante
- Muito importante
- Moderadamente importante
- Levemente importante
- Nada importante

Você selecionou "... porque tem pouca gordura "
O quanto isso é importante para você?

- Extremamente importante
- Muito importante
- Moderadamente importante
- Levemente importante
- Nada importante

Você selecionou "... porque eu vejo propaganda em algum lugar ou vejo na TV "
O quanto isso é importante para você?

- Extremamente importante
- Muito importante
- Moderadamente importante
- Levemente importante
- Nada importante

Você selecionou "... porque deixa as reuniões sociais mais agradáveis "
O quanto isso é importante para você?

- Extremamente importante
- Muito importante
- Moderadamente importante
- Levemente importante
- Nada importante

Você selecionou "... porque eu devo comer "
O quanto isso é importante para você?

- Extremamente importante
- Muito importante
- Moderadamente importante
- Levemente importante
- Nada importante

Você selecionou "... porque os outros gostam "
O quanto isso é importante para você?

- Extremamente importante
- Muito importante
- Moderadamente importante
- Levemente importante
- Nada importante

Você selecionou "... porque eu me sinto sozinho "
O quanto isso é importante para você?

- Extremamente importante
- Muito importante
- Moderadamente importante
- Levemente importante
- Nada importante

Você selecionou "... porque eu controlo meu peso "
O quanto isso é importante para você?

- Extremamente importante
- Muito importante
- Moderadamente importante
- Levemente importante
- Nada importante

Você selecionou "... porque não contém substâncias perigosas como agrotóxicos, poluentes, antibióticos "
O quanto isso é importante para você?

- Extremamente importante
- Muito importante
- Moderadamente importante
- Levemente importante
- Nada importante

Você selecionou "... porque é mais conveniente "
O quanto isso é importante para você?

- Extremamente importante
- Muito importante
- Moderadamente importante
- Levemente importante
- Nada importante

Você selecionou "... porque eu preciso de energia "
O quanto isso é importante para você?

- Extremamente importante
- Muito importante
- Moderadamente importante
- Levemente importante
- Nada importante

Você selecionou "... porque me é familiar "
O quanto isso é importante para você?

- Extremamente importante
- Muito importante
- Moderadamente importante
- Levemente importante
- Nada importante

Você selecionou "... porque eu gosto "
O quanto isso é importante para você?

- Extremamente importante
- Muito importante
- Moderadamente importante
- Levemente importante
- Nada importante

Você selecionou "... porque me sacia de forma agradável "
O quanto isso é importante para você?

- Extremamente importante
- Muito importante
- Moderadamente importante
- Levemente importante
- Nada importante

Você selecionou "... para compensar "
O quanto isso é importante para você?

- Extremamente importante
- Muito importante
- Moderadamente importante
- Levemente importante
- Nada importante

Você selecionou "... porque não faz parte das tradições como Natal, Ano Novo e ocasiões especiais "
O quanto isso é importante para você?

- Extremamente importante
- Muito importante
- Moderadamente importante
- Levemente importante
- Nada importante

Você selecionou "... porque faz parte de certas situações "
O quanto isso é importante para você?

- Extremamente importante
- Muito importante
- Moderadamente importante
- Levemente importante
- Nada importante

Você selecionou "... para manter uma dieta balanceada "
O quanto isso é importante para você?

- Extremamente importante
- Muito importante
- Moderadamente importante
- Levemente importante
- Nada importante

Você selecionou "... porque é sociável "
O quanto isso é importante para você?

- Extremamente importante
- Muito importante
- Moderadamente importante
- Levemente importante
- Nada importante

Você selecionou "... porque não é caro "
O quanto isso é importante para você?

- Extremamente importante
- Muito importante
- Moderadamente importante
- Levemente importante
- Nada importante

Você selecionou "... porque me atrai aparentemente, por exemplo cores atraentes "
O quanto isso é importante para você?

- Extremamente importante
- Muito importante
- Moderadamente importante
- Levemente importante
- Nada importante

Você selecionou "... para não desapontar alguém que está tentando me agradar "
O quanto isso é importante para você?

- Extremamente importante
- Muito importante
- Moderadamente importante
- Levemente importante

- Nada importante

Você selecionou "... porque é moda "
O quanto isso é importante para você?

- Extremamente importante
- Muito importante
- Moderadamente importante
- Levemente importante
- Nada importante

Você selecionou "... porque está em promoção "
O quanto isso é importante para você?

- Extremamente importante
- Muito importante
- Moderadamente importante
- Levemente importante
- Nada importante

Você selecionou "... porque me mantém em forma exemplo, vigoroso, dinâmico "
O quanto isso é importante para você?

- Extremamente importante
- Muito importante
- Moderadamente importante
- Levemente importante
- Nada importante

Você selecionou "... porque é a única escolha "
O quanto isso é importante para você?

- Extremamente importante
- Muito importante
- Moderadamente importante
- Levemente importante
- Nada importante

Você selecionou "... porque eu quero comer todos os dias "
O quanto isso é importante para você?

- Extremamente importante
- Muito importante
- Moderadamente importante
- Levemente importante
- Nada importante

Quais as razões pelas quais você não come arroz? (Marque todas as respostas que correspondem)

- ... O preço está muito alto
- ... Não é um alimento que eu como perto de outras pessoas
- .. Não é nada que eu queira comer agora
- ... Eu não gosto do jeito que parece
- .. Eu comi recentemente e não quero comer a mesma comida com tanta frequência
- ... Não é apropriado para a situação
- ... Eu não quero me dar ao luxo
- ... Muito rico em calorias
- ... Eu nunca escolheria isso e eu gosto de comer a mesma comida todos os dias
- ... Essa comida me faz sentir triste, solitário e frustrado
- ... Eu normalmente não como isso
- ... Comer isso me faz parecer antiquado
- ... Não é conveniente

- ☐ ... Eu não devo comer isso
- ☐ ... Eu não gosto disso
- ☐ ... Eu não acho que seja um lanche
- ☐ ... O tamanho da porção não é adequado
- ☐ ... Não é saudável
- ☐ ... Não é orgânico
- ☐ ... Não sustenta o suficiente

Você selecionou "... Eu não acho que seja um lanche"
O quanto isso é importante para você?

- ○ Extremamente importante
- ○ Muito importante
- ○ Moderadamente importante
- ○ Levemente importante
- ○ Nada importante

Você selecionou "... Eu não gosto disso"
O quanto isso é importante para você?

- ○ Extremamente importante
- ○ Muito importante
- ○ Moderadamente importante
- ○ Levemente importante
- ○ Nada importante

Você selecionou "... Eu normalmente não como isso"
O quanto isso é importante para você?

- ○ Extremamente importante
- ○ Muito importante
- ○ Moderadamente importante

- Levemente importante
- Nada importante

Você selecionou "... O tamanho da porção não é adequado"
O quanto isso é importante para você?

- Extremamente importante
- Muito importante
- Moderadamente importante
- Levemente importante
- Nada importante

Você selecionou "... Não sustenta o suficiente"
O quanto isso é importante para você?

- Extremamente importante
- Muito importante
- Moderadamente importante
- Levemente importante
- Nada importante

Você selecionou "... Não é saudável"
O quanto isso é importante para você?

- Extremamente importante
- Muito importante
- Moderadamente importante
- Levemente importante
- Nada importante

Você selecionou "... Não é conveniente"

O quanto isso é importante para você?

- Extremamente importante
- Muito importante
- Moderadamente importante
- Levemente importante
- Nada importante

Você selecionou "... Eu não quero me dar ao luxo"
O quanto isso é importante para você?

- Extremamente importante
- Muito importante
- Moderadamente importante
- Levemente importante
- Nada importante

Você selecionou "... Não é apropriado para a situação"
O quanto isso é importante para você?

- Extremamente importante
- Muito importante
- Moderadamente importante
- Levemente importante
- Nada importante

Você selecionou "... Não é um alimento que eu como perto de outras pessoas"
O quanto isso é importante para você?

- Extremamente importante
- Muito importante
- Moderadamente importante

- Levemente importante
- Nada importante

Você selecionou "... O preço está muito alto"
O quanto isso é importante para você?

- Extremamente importante
- Muito importante
- Moderadamente importante
- Levemente importante
- Nada importante

Você selecionou "... Eu não gosto do jeito que parece"
O quanto isso é importante para você?

- Extremamente importante
- Muito importante
- Moderadamente importante
- Levemente importante
- Nada importante

Você selecionou "... Muito rico em calorias"
O quanto isso é importante para você?

- Extremamente importante
- Muito importante
- Moderadamente importante
- Levemente importante
- Nada importante

Você selecionou "... Eu não devo comer isso"

O quanto isso é importante para você?

- Extremamente importante
- Muito importante
- Moderadamente importante
- Levemente importante
- Nada importante

Você selecionou "... Comer isso me faz parecer antiquado"
O quanto isso é importante para você?

- Extremamente importante
- Muito importante
- Moderadamente importante
- Levemente importante
- Nada importante

Você selecionou ".. Não é nada que eu queira comer agora"
O quanto isso é importante para você?

- Extremamente importante
- Muito importante
- Moderadamente importante
- Levemente importante
- Nada importante

Você selecionou ".. Eu comi recentemente e não quero comer a mesma comida com tanta frequência"
O quanto isso é importante para você?

- Extremamente importante
- Muito importante

- Moderadamente importante
- Levemente importante
- Nada importante

Você selecionou "... Eu nunca escolheria isso e eu gosto de comer a mesma comida todos os dias"
O quanto isso é importante para você?

- Extremamente importante
- Muito importante
- Moderadamente importante
- Levemente importante
- Nada importante

Você selecionou "... Essa comida me faz sentir triste, solitário e frustrado"
O quanto isso é importante para você?

- Extremamente importante
- Muito importante
- Moderadamente importante
- Levemente importante
- Nada importante

Você selecionou "... Não é orgânico"
O quanto isso é importante para você?

- Extremamente importante
- Muito importante
- Moderadamente importante
- Levemente importante
- Nada importante

Reasons for Protein foods Choices(RATA)

Você consome feijão?

- Sim
- Não

Quais as razões pelas quais você come feijão? (Marque todas as respostas que correspondem)

- ... porque é sociável
- ... porque me sacia de forma agradável
- ... porque eu gosto
- ... porque estou habituado
- ... porque é a única escolha
- ... porque é orgânico
- ... porque me dou ao luxo
- ... porque me atrai aparentemente, por exemplo cores atraentes
- ... porque eu controlo meu peso
- ... porque é mais conveniente
- ... porque está em promoção
- ... porque deixa as reuniões sociais mais agradáveis
- ... porque é saboroso
- ... porque é natural (exemplo, não geneticamente modificado)
- ... porque eu preciso de energia
- ... porque seria falta de educação não comer isso
- ... porque é saudável
- ... porque estou frustrado
- ... porque eu devo comer
- ... porque eu vejo propaganda em algum lugar ou vejo na TV
- ... porque não faz parte das tradições como Natal, Ano Novo e ocasiões especiais

- ☐ ... porque eu me sinto sozinho
- ☐ ... porque a apresentação é atraente, por exemplo a embalagem
- ☐ ... porque estou faminto
- ☐ ... porque me é familiar
- ☐ ... porque eu estou triste
- ☐ ... porque tem baixas calorias
- ☐ ... porque me faz parecer melhor na frente dos outros
- ☐ ... para não desapontar alguém que está tentando me agradar
- ☐ ... porque normalmente eu como isso
- ☐ ... porque é fácil de preparar
- ☐ ... porque eu posso desfrutar o momento com outras pessoas
- ☐ ... para compensar
- ☐ ... porque eu não quero gastar muito dinheiro
- ☐ ... porque não contém substâncias perigosas como agrotóxicos, poluentes, antibióticos
- ☐ ... porque é moda
- ☐ ... porque faz parte de certas situações
- ☐ ... porque é rápido de preparar
- ☐ ... porque eu tenho vontade de comer isso
- ☐ ... para manter uma dieta balanceada
- ☐ ... porque eu quero comer todos os dias
- ☐ ... porque tem pouca gordura
- ☐ ... porque eu curto
- ☐ ... porque os outros gostam
- ☐ ... porque não é caro
- ☐ ... porque eu cresci comendo isso
- ☐ ... porque me mantém em forma exemplo, vigoroso, dinâmico

---

Você selecionou "... porque estou habituado "
O quanto isso é importante para você?

- ◯ Extremamente importante

- Muito importante
- Moderadamente importante
- Levemente importante
- Nada importante

Você selecionou "... porque é saudável "
O quanto isso é importante para você?

- Extremamente importante
- Muito importante
- Moderadamente importante
- Levemente importante
- Nada importante

Você selecionou "... porque é rápido de preparar "
O quanto isso é importante para você?

- Extremamente importante
- Muito importante
- Moderadamente importante
- Levemente importante
- Nada importante

Você selecionou "... porque é saboroso "
O quanto isso é importante para você?

- Extremamente importante
- Muito importante
- Moderadamente importante
- Levemente importante
- Nada importante

Você selecionou "... porque me dou ao luxo "
O quanto isso é importante para você?

- Extremamente importante
- Muito importante
- Moderadamente importante
- Levemente importante
- Nada importante

Você selecionou "... porque é natural (exemplo, não geneticamente modificado) "
O quanto isso é importante para você?

- Extremamente importante
- Muito importante
- Moderadamente importante
- Levemente importante
- Nada importante

Você selecionou "... porque eu não quero gastar muito dinheiro "
O quanto isso é importante para você?

- Extremamente importante
- Muito importante
- Moderadamente importante
- Levemente importante
- Nada importante

Você selecionou "... porque tem baixas calorias "
O quanto isso é importante para você?

- Extremamente importante

- Muito importante
- Moderadamente importante
- Levemente importante
- Nada importante

Você selecionou "... porque estou frustrado "
O quanto isso é importante para você?

- Extremamente importante
- Muito importante
- Moderadamente importante
- Levemente importante
- Nada importante

Você selecionou "... porque me faz parecer melhor na frente dos outros "
O quanto isso é importante para você?

- Extremamente importante
- Muito importante
- Moderadamente importante
- Levemente importante
- Nada importante

Você selecionou "... porque seria falta de educação não comer isso "
O quanto isso é importante para você?

- Extremamente importante
- Muito importante
- Moderadamente importante
- Levemente importante
- Nada importante

Você selecionou "... porque a apresentação é atraente, por exemplo a embalagem "

O quanto isso é importante para você?

- Extremamente importante
- Muito importante
- Moderadamente importante
- Levemente importante
- Nada importante

Você selecionou "... porque eu posso desfrutar o momento com outras pessoas "

O quanto isso é importante para você?

- Extremamente importante
- Muito importante
- Moderadamente importante
- Levemente importante
- Nada importante

Você selecionou "... porque é orgânico "

O quanto isso é importante para você?

- Extremamente importante
- Muito importante
- Moderadamente importante
- Levemente importante
- Nada importante

Você selecionou "... porque eu cresci comendo isso "

O quanto isso é importante para você?

- Extremamente importante

- Muito importante
- Moderadamente importante
- Levemente importante
- Nada importante

Você selecionou "... porque eu curto "
O quanto isso é importante para você?

- Extremamente importante
- Muito importante
- Moderadamente importante
- Levemente importante
- Nada importante

Você selecionou "... porque estou faminto "
O quanto isso é importante para você?

- Extremamente importante
- Muito importante
- Moderadamente importante
- Levemente importante
- Nada importante

Você selecionou "... porque normalmente eu como isso "
O quanto isso é importante para você?

- Extremamente importante
- Muito importante
- Moderadamente importante
- Levemente importante
- Nada importante

Você selecionou "... porque eu tenho vontade de comer isso "
O quanto isso é importante para você?

- Extremamente importante
- Muito importante
- Moderadamente importante
- Levemente importante
- Nada importante

Você selecionou "... porque é fácil de preparar "
O quanto isso é importante para você?

- Extremamente importante
- Muito importante
- Moderadamente importante
- Levemente importante
- Nada importante

Você selecionou "... porque eu estou triste "
O quanto isso é importante para você?

- Extremamente importante
- Muito importante
- Moderadamente importante
- Levemente importante
- Nada importante

Você selecionou "... porque tem pouca gordura "
O quanto isso é importante para você?

- Extremamente importante

- Muito importante
- Moderadamente importante
- Levemente importante
- Nada importante

Você selecionou "... porque eu vejo propaganda em algum lugar ou vejo na TV "
O quanto isso é importante para você?

- Extremamente importante
- Muito importante
- Moderadamente importante
- Levemente importante
- Nada importante

Você selecionou "... porque deixa as reuniões sociais mais agradáveis "
O quanto isso é importante para você?

- Extremamente importante
- Muito importante
- Moderadamente importante
- Levemente importante
- Nada importante

Você selecionou "... porque eu devo comer "
O quanto isso é importante para você?

- Extremamente importante
- Muito importante
- Moderadamente importante
- Levemente importante
- Nada importante

Você selecionou "... porque os outros gostam "
O quanto isso é importante para você?

- Extremamente importante
- Muito importante
- Moderadamente importante
- Levemente importante
- Nada importante

Você selecionou "... porque eu me sinto sozinho "
O quanto isso é importante para você?

- Extremamente importante
- Muito importante
- Moderadamente importante
- Levemente importante
- Nada importante

Você selecionou "... porque eu controlo meu peso "
O quanto isso é importante para você?

- Extremamente importante
- Muito importante
- Moderadamente importante
- Levemente importante
- Nada importante

Você selecionou "... porque não contém substâncias perigosas como agrotóxicos, poluentes, antibióticos "
O quanto isso é importante para você?

- Extremamente importante

- Muito importante
- Moderadamente importante
- Levemente importante
- Nada importante

Você selecionou "... porque é mais conveniente "
O quanto isso é importante para você?

- Extremamente importante
- Muito importante
- Moderadamente importante
- Levemente importante
- Nada importante

Você selecionou "... porque eu preciso de energia "
O quanto isso é importante para você?

- Extremamente importante
- Muito importante
- Moderadamente importante
- Levemente importante
- Nada importante

Você selecionou "... porque me é familiar "
O quanto isso é importante para você?

- Extremamente importante
- Muito importante
- Moderadamente importante
- Levemente importante
- Nada importante

Você selecionou "... porque eu gosto "
O quanto isso é importante para você?

- Extremamente importante
- Muito importante
- Moderadamente importante
- Levemente importante
- Nada importante

Você selecionou "... porque me sacia de forma agradável "
O quanto isso é importante para você?

- Extremamente importante
- Muito importante
- Moderadamente importante
- Levemente importante
- Nada importante

Você selecionou "... para compensar "
O quanto isso é importante para você?

- Extremamente importante
- Muito importante
- Moderadamente importante
- Levemente importante
- Nada importante

Você selecionou "... porque não faz parte das tradições como Natal, Ano Novo e ocasiões especiais "
O quanto isso é importante para você?

- Extremamente importante

- Muito importante
- Moderadamente importante
- Levemente importante
- Nada importante

Você selecionou "... porque faz parte de certas situações "
O quanto isso é importante para você?

- Extremamente importante
- Muito importante
- Moderadamente importante
- Levemente importante
- Nada importante

Você selecionou "... para manter uma dieta balanceada "
O quanto isso é importante para você?

- Extremamente importante
- Muito importante
- Moderadamente importante
- Levemente importante
- Nada importante

Você selecionou "... porque é sociável "
O quanto isso é importante para você?

- Extremamente importante
- Muito importante
- Moderadamente importante
- Levemente importante
- Nada importante

Você selecionou "... porque não é caro "
O quanto isso é importante para você?

- Extremamente importante
- Muito importante
- Moderadamente importante
- Levemente importante
- Nada importante

Você selecionou "... porque me atrai aparentemente, por exemplo cores atraentes "
O quanto isso é importante para você?

- Extremamente importante
- Muito importante
- Moderadamente importante
- Levemente importante
- Nada importante

Você selecionou "... para não desapontar alguém que está tentando me agradar "
O quanto isso é importante para você?

- Extremamente importante
- Muito importante
- Moderadamente importante
- Levemente importante
- Nada importante

Você selecionou "... porque é moda "
O quanto isso é importante para você?

- Extremamente importante

- Muito importante
- Moderadamente importante
- Levemente importante
- Nada importante

Você selecionou "... porque está em promoção "
O quanto isso é importante para você?

- Extremamente importante
- Muito importante
- Moderadamente importante
- Levemente importante
- Nada importante

Você selecionou "... porque me mantém em forma exemplo, vigoroso, dinâmico "
O quanto isso é importante para você?

- Extremamente importante
- Muito importante
- Moderadamente importante
- Levemente importante
- Nada importante

Você selecionou "... porque é a única escolha "
O quanto isso é importante para você?

- Extremamente importante
- Muito importante
- Moderadamente importante
- Levemente importante
- Nada importante

Você selecionou "... porque eu quero comer todos os dias "
O quanto isso é importante para você?

- Extremamente importante
- Muito importante
- Moderadamente importante
- Levemente importante
- Nada importante

Quais as razões pelas quais você não come feijão? (Marque todas as respostas que correspondem)

- ... Eu não quero me dar ao luxo
- ... Não é orgânico
- ... Não é um alimento que eu como perto de outras pessoas
- ... Essa comida me faz sentir triste, solitário e frustrado
- ... Não é apropriado para a situação
- ... Não é saudável
- ... Eu normalmente não como isso
- ... Eu nunca escolheria isso e eu gosto de comer a mesma comida todos os dias
- .. Eu comi recentemente e não quero comer a mesma comida com tanta frequência
- ... Não sustenta o suficiente
- ... Eu não devo comer isso
- ... Eu não gosto do jeito que parece
- ... Eu não acho que seja um lanche
- ... O tamanho da porção não é adequado
- ... Comer isso me faz parecer antiquado
- ... Muito rico em calorias
- .. Não é nada que eu queira comer agora
- ... O preço está muito alto
- ... Eu não gosto disso

- [ ] ... Não é conveniente

Você selecionou "... Eu não acho que seja um lanche"
O quanto isso é importante para você?

- Extremamente importante
- Muito importante
- Moderadamente importante
- Levemente importante
- Nada importante

Você selecionou "... Eu não gosto disso"
O quanto isso é importante para você?

- Extremamente importante
- Muito importante
- Moderadamente importante
- Levemente importante
- Nada importante

Você selecionou "... Eu normalmente não como isso"
O quanto isso é importante para você?

- Extremamente importante
- Muito importante
- Moderadamente importante
- Levemente importante
- Nada importante

Você selecionou "... O tamanho da porção não é adequado"
O quanto isso é importante para você?

- Extremamente importante
- Muito importante
- Moderadamente importante
- Levemente importante
- Nada importante

Você selecionou "... Não sustenta o suficiente"
O quanto isso é importante para você?

- Extremamente importante
- Muito importante
- Moderadamente importante
- Levemente importante
- Nada importante

Você selecionou "... Não é saudável"
O quanto isso é importante para você?

- Extremamente importante
- Muito importante
- Moderadamente importante
- Levemente importante
- Nada importante

Você selecionou "... Não é conveniente"
O quanto isso é importante para você?

- Extremamente importante
- Muito importante
- Moderadamente importante
- Levemente importante

- Nada importante

Você selecionou "... Eu não quero me dar ao luxo"
O quanto isso é importante para você?

- Extremamente importante
- Muito importante
- Moderadamente importante
- Levemente importante
- Nada importante

Você selecionou "... Não é apropriado para a situação"
O quanto isso é importante para você?

- Extremamente importante
- Muito importante
- Moderadamente importante
- Levemente importante
- Nada importante

Você selecionou "... Não é um alimento que eu como perto de outras pessoas"
O quanto isso é importante para você?

- Extremamente importante
- Muito importante
- Moderadamente importante
- Levemente importante
- Nada importante

Você selecionou "... O preço está muito alto"
O quanto isso é importante para você?

- Extremamente importante
- Muito importante
- Moderadamente importante
- Levemente importante
- Nada importante

Você selecionou "... Eu não gosto do jeito que parece"
O quanto isso é importante para você?

- Extremamente importante
- Muito importante
- Moderadamente importante
- Levemente importante
- Nada importante

Você selecionou "... Muito rico em calorias"
O quanto isso é importante para você?

- Extremamente importante
- Muito importante
- Moderadamente importante
- Levemente importante
- Nada importante

Você selecionou "... Eu não devo comer isso"
O quanto isso é importante para você?

- Extremamente importante
- Muito importante
- Moderadamente importante
- Levemente importante

- Nada importante

Você selecionou "... Comer isso me faz parecer antiquado"
O quanto isso é importante para você?

- Extremamente importante
- Muito importante
- Moderadamente importante
- Levemente importante
- Nada importante

Você selecionou ".. Não é nada que eu queira comer agora"
O quanto isso é importante para você?

- Extremamente importante
- Muito importante
- Moderadamente importante
- Levemente importante
- Nada importante

Você selecionou ".. Eu comi recentemente e não quero comer a mesma comida com tanta frequência"
O quanto isso é importante para você?

- Extremamente importante
- Muito importante
- Moderadamente importante
- Levemente importante
- Nada importante

Você selecionou "... Eu nunca escolheria isso e eu gosto de comer a mesma comida todos os dias"
O quanto isso é importante para você?

- Extremamente importante
- Muito importante
- Moderadamente importante
- Levemente importante
- Nada importante

Você selecionou "... Essa comida me faz sentir triste, solitário e frustrado"
O quanto isso é importante para você?

- Extremamente importante
- Muito importante
- Moderadamente importante
- Levemente importante
- Nada importante

Você selecionou "... Não é orgânico"
O quanto isso é importante para você?

- Extremamente importante
- Muito importante
- Moderadamente importante
- Levemente importante
- Nada importante

Reasons for Dairy Food Choices(RATA)

Você consome leite?

- Sim
- Não

Quais as razões pelas quais você come leite? (Marque todas as respostas que correspondem)

- ... porque normalmente eu como isso
- ... porque deixa as reuniões sociais mais agradáveis
- ... porque é saboroso
- ... porque tem pouca gordura
- ... porque a apresentação é atraente, por exemplo a embalagem
- ... para não desapontar alguém que está tentando me agradar
- ... porque me dou ao luxo
- ... para compensar
- ... porque estou habituado
- ... porque faz parte de certas situações
- ... porque está em promoção
- ... porque eu tenho vontade de comer isso
- ... porque não é caro
- ... porque eu controlo meu peso
- ... porque os outros gostam
- ... porque é sociável
- ... porque eu curto
- ... porque me mantém em forma exemplo, vigoroso, dinâmico
- ... porque eu me sinto sozinho
- ... porque é saudável
- ... para manter uma dieta balanceada
- ... porque eu posso desfrutar o momento com outras pessoas
- ... porque é mais conveniente
- ... porque eu quero comer todos os dias
- ... porque me atrai aparentemente, por exemplo cores atraentes

- ☐ ... porque eu preciso de energia
- ☐ ... porque eu cresci comendo isso
- ☐ ... porque não faz parte das tradições como Natal, Ano Novo e ocasiões especiais
- ☐ ... porque eu gosto
- ☐ ... porque eu estou triste
- ☐ ... porque é rápido de preparar
- ☐ ... porque eu vejo propaganda em algum lugar ou vejo na TV
- ☐ ... porque me é familiar
- ☐ ... porque é fácil de preparar
- ☐ ... porque é natural (exemplo, não geneticamente modificado)
- ☐ ... porque é orgânico
- ☐ ... porque não contém substâncias perigosas como agrotóxicos, poluentes, antibióticos
- ☐ ... porque eu devo comer
- ☐ ... porque eu não quero gastar muito dinheiro
- ☐ ... porque me sacia de forma agradável
- ☐ ... porque estou frustrado
- ☐ ... porque seria falta de educação não comer isso
- ☐ ... porque tem baixas calorias
- ☐ ... porque é a única escolha
- ☐ ... porque me faz parecer melhor na frente dos outros
- ☐ ... porque é moda
- ☐ ... porque estou faminto

Você selecionou "... porque estou habituado "
O quanto isso é importante para você?

- ◯ Extremamente importante
- ◯ Muito importante
- ◯ Moderadamente importante
- ◯ Levemente importante
- ◯ Nada importante

Você selecionou "... porque é saudável "
O quanto isso é importante para você?

- Extremamente importante
- Muito importante
- Moderadamente importante
- Levemente importante
- Nada importante

Você selecionou "... porque é rápido de preparar "
O quanto isso é importante para você?

- Extremamente importante
- Muito importante
- Moderadamente importante
- Levemente importante
- Nada importante

Você selecionou "... porque é saboroso "
O quanto isso é importante para você?

- Extremamente importante
- Muito importante
- Moderadamente importante
- Levemente importante
- Nada importante

Você selecionou "... porque me dou ao luxo "
O quanto isso é importante para você?

- Extremamente importante

- Muito importante
- Moderadamente importante
- Levemente importante
- Nada importante

Você selecionou "... porque é natural (exemplo, não geneticamente modificado) "
O quanto isso é importante para você?

- Extremamente importante
- Muito importante
- Moderadamente importante
- Levemente importante
- Nada importante

Você selecionou "... porque eu não quero gastar muito dinheiro "
O quanto isso é importante para você?

- Extremamente importante
- Muito importante
- Moderadamente importante
- Levemente importante
- Nada importante

Você selecionou "... porque tem baixas calorias "
O quanto isso é importante para você?

- Extremamente importante
- Muito importante
- Moderadamente importante
- Levemente importante
- Nada importante

Você selecionou "... porque estou frustrado "
O quanto isso é importante para você?

- Extremamente importante
- Muito importante
- Moderadamente importante
- Levemente importante
- Nada importante

Você selecionou "... porque me faz parecer melhor na frente dos outros "
O quanto isso é importante para você?

- Extremamente importante
- Muito importante
- Moderadamente importante
- Levemente importante
- Nada importante

Você selecionou "... porque seria falta de educação não comer isso "
O quanto isso é importante para você?

- Extremamente importante
- Muito importante
- Moderadamente importante
- Levemente importante
- Nada importante

Você selecionou "... porque a apresentação é atraente, por exemplo a embalagem "
O quanto isso é importante para você?

- Extremamente importante

- Muito importante
- Moderadamente importante
- Levemente importante
- Nada importante

Você selecionou "... porque eu posso desfrutar o momento com outras pessoas "
O quanto isso é importante para você?

- Extremamente importante
- Muito importante
- Moderadamente importante
- Levemente importante
- Nada importante

Você selecionou "... porque é orgânico "
O quanto isso é importante para você?

- Extremamente importante
- Muito importante
- Moderadamente importante
- Levemente importante
- Nada importante

Você selecionou "... porque eu cresci comendo isso "
O quanto isso é importante para você?

- Extremamente importante
- Muito importante
- Moderadamente importante
- Levemente importante
- Nada importante

Você selecionou "... porque eu curto "
O quanto isso é importante para você?

- Extremamente importante
- Muito importante
- Moderadamente importante
- Levemente importante
- Nada importante

Você selecionou "... porque estou faminto "
O quanto isso é importante para você?

- Extremamente importante
- Muito importante
- Moderadamente importante
- Levemente importante
- Nada importante

Você selecionou "... porque normalmente eu como isso "
O quanto isso é importante para você?

- Extremamente importante
- Muito importante
- Moderadamente importante
- Levemente importante
- Nada importante

Você selecionou "... porque eu tenho vontade de comer isso "
O quanto isso é importante para você?

- Extremamente importante

- Muito importante
- Moderadamente importante
- Levemente importante
- Nada importante

Você selecionou "... porque é fácil de preparar "
O quanto isso é importante para você?

- Extremamente importante
- Muito importante
- Moderadamente importante
- Levemente importante
- Nada importante

Você selecionou "... porque eu estou triste "
O quanto isso é importante para você?

- Extremamente importante
- Muito importante
- Moderadamente importante
- Levemente importante
- Nada importante

Você selecionou "... porque tem pouca gordura "
O quanto isso é importante para você?

- Extremamente importante
- Muito importante
- Moderadamente importante
- Levemente importante
- Nada importante

Você selecionou "... porque eu vejo propaganda em algum lugar ou vejo na TV "
O quanto isso é importante para você?

- Extremamente importante
- Muito importante
- Moderadamente importante
- Levemente importante
- Nada importante

Você selecionou "... porque deixa as reuniões sociais mais agradáveis "
O quanto isso é importante para você?

- Extremamente importante
- Muito importante
- Moderadamente importante
- Levemente importante
- Nada importante

Você selecionou "... porque eu devo comer "
O quanto isso é importante para você?

- Extremamente importante
- Muito importante
- Moderadamente importante
- Levemente importante
- Nada importante

Você selecionou "... porque os outros gostam "
O quanto isso é importante para você?

- Extremamente importante

- Muito importante
- Moderadamente importante
- Levemente importante
- Nada importante

Você selecionou "... porque eu me sinto sozinho "
O quanto isso é importante para você?

- Extremamente importante
- Muito importante
- Moderadamente importante
- Levemente importante
- Nada importante

Você selecionou "... porque eu controlo meu peso "
O quanto isso é importante para você?

- Extremamente importante
- Muito importante
- Moderadamente importante
- Levemente importante
- Nada importante

Você selecionou "... porque não contém substâncias perigosas como agrotóxicos, poluentes, antibióticos "
O quanto isso é importante para você?

- Extremamente importante
- Muito importante
- Moderadamente importante
- Levemente importante
- Nada importante

Você selecionou "... porque é mais conveniente "
O quanto isso é importante para você?

- Extremamente importante
- Muito importante
- Moderadamente importante
- Levemente importante
- Nada importante

Você selecionou "... porque eu preciso de energia "
O quanto isso é importante para você?

- Extremamente importante
- Muito importante
- Moderadamente importante
- Levemente importante
- Nada importante

Você selecionou "... porque me é familiar "
O quanto isso é importante para você?

- Extremamente importante
- Muito importante
- Moderadamente importante
- Levemente importante
- Nada importante

Você selecionou "... porque eu gosto "
O quanto isso é importante para você?

- Extremamente importante
- Muito importante
- Moderadamente importante
- Levemente importante
- Nada importante

Você selecionou "... porque me sacia de forma agradável "
O quanto isso é importante para você?

- Extremamente importante
- Muito importante
- Moderadamente importante
- Levemente importante
- Nada importante

Você selecionou "... para compensar "
O quanto isso é importante para você?

- Extremamente importante
- Muito importante
- Moderadamente importante
- Levemente importante
- Nada importante

Você selecionou "... porque não faz parte das tradições como Natal, Ano Novo e ocasiões especiais "
O quanto isso é importante para você?

- Extremamente importante
- Muito importante
- Moderadamente importante
- Levemente importante

- Nada importante

Você selecionou "... porque faz parte de certas situações "
O quanto isso é importante para você?

- Extremamente importante
- Muito importante
- Moderadamente importante
- Levemente importante
- Nada importante

Você selecionou "... para manter uma dieta balanceada "
O quanto isso é importante para você?

- Extremamente importante
- Muito importante
- Moderadamente importante
- Levemente importante
- Nada importante

Você selecionou "... porque é sociável "
O quanto isso é importante para você?

- Extremamente importante
- Muito importante
- Moderadamente importante
- Levemente importante
- Nada importante

Você selecionou "... porque não é caro "
O quanto isso é importante para você?

- Extremamente importante
- Muito importante
- Moderadamente importante
- Levemente importante
- Nada importante

Você selecionou "... porque me atrai aparentemente, por exemplo cores atraentes "

O quanto isso é importante para você?

- Extremamente importante
- Muito importante
- Moderadamente importante
- Levemente importante
- Nada importante

Você selecionou "... para não desapontar alguém que está tentando me agradar "
O quanto isso é importante para você?

- Extremamente importante
- Muito importante
- Moderadamente importante
- Levemente importante
- Nada importante

Você selecionou "... porque é moda "
O quanto isso é importante para você?

- Extremamente importante
- Muito importante
- Moderadamente importante

- Levemente importante
- Nada importante

Você selecionou "... porque está em promoção "
O quanto isso é importante para você?

- Extremamente importante
- Muito importante
- Moderadamente importante
- Levemente importante
- Nada importante

Você selecionou "... porque me mantém em forma exemplo, vigoroso, dinâmico "
O quanto isso é importante para você?

- Extremamente importante
- Muito importante
- Moderadamente importante
- Levemente importante
- Nada importante

Você selecionou "... porque é a única escolha "
O quanto isso é importante para você?

- Extremamente importante
- Muito importante
- Moderadamente importante
- Levemente importante
- Nada importante

Você selecionou "... porque eu quero comer todos os dias "

O quanto isso é importante para você?

- Extremamente importante
- Muito importante
- Moderadamente importante
- Levemente importante
- Nada importante

Quais as razões pelas quais você não come leite? (Marque todas as respostas que correspondem)

- ... Não é conveniente
- ... Eu normalmente não como isso
- ... Eu nunca escolheria isso e eu gosto de comer a mesma comida todos os dias
- ... Essa comida me faz sentir triste, solitário e frustrado
- ... Não é saudável
- ... Eu não quero me dar ao luxo
- ... Eu não gosto disso
- ... O preço está muito alto
- ... Eu não gosto do jeito que parece
- .. Eu comi recentemente e não quero comer a mesma comida com tanta frequência
- .. Não é nada que eu queira comer agora
- ... Comer isso me faz parecer antiquado
- ... Não é orgânico
- ... Eu não acho que seja um lanche
- ... Eu não devo comer isso
- ... Não é apropriado para a situação
- ... O tamanho da porção não é adequado
- ... Muito rico em calorias
- ... Não sustenta o suficiente
- ... Não é um alimento que eu como perto de outras pessoas

Você selecionou "... Eu não acho que seja um lanche"
O quanto isso é importante para você?

- Extremamente importante
- Muito importante
- Moderadamente importante
- Levemente importante
- Nada importante

Você selecionou "... Eu não gosto disso"
O quanto isso é importante para você?

- Extremamente importante
- Muito importante
- Moderadamente importante
- Levemente importante
- Nada importante

Você selecionou "... Eu normalmente não como isso"
O quanto isso é importante para você?

- Extremamente importante
- Muito importante
- Moderadamente importante
- Levemente importante
- Nada importante

Você selecionou "... O tamanho da porção não é adequado"
O quanto isso é importante para você?

- Extremamente importante
- Muito importante

- Moderadamente importante
- Levemente importante
- Nada importante

Você selecionou "... Não sustenta o suficiente"
O quanto isso é importante para você?

- Extremamente importante
- Muito importante
- Moderadamente importante
- Levemente importante
- Nada importante

Você selecionou "... Não é saudável"
O quanto isso é importante para você?

- Extremamente importante
- Muito importante
- Moderadamente importante
- Levemente importante
- Nada importante

Você selecionou "... Não é conveniente"
O quanto isso é importante para você?

- Extremamente importante
- Muito importante
- Moderadamente importante
- Levemente importante
- Nada importante

Você selecionou "... Eu não quero me dar ao luxo"
O quanto isso é importante para você?

- Extremamente importante
- Muito importante
- Moderadamente importante
- Levemente importante
- Nada importante

Você selecionou "... Não é apropriado para a situação"
O quanto isso é importante para você?

- Extremamente importante
- Muito importante
- Moderadamente importante
- Levemente importante
- Nada importante

Você selecionou "... Não é um alimento que eu como perto de outras pessoas"
O quanto isso é importante para você?

- Extremamente importante
- Muito importante
- Moderadamente importante
- Levemente importante
- Nada importante

Você selecionou "... O preço está muito alto"
O quanto isso é importante para você?

- Extremamente importante
- Muito importante

- Moderadamente importante
- Levemente importante
- Nada importante

Você selecionou "... Eu não gosto do jeito que parece"
O quanto isso é importante para você?

- Extremamente importante
- Muito importante
- Moderadamente importante
- Levemente importante
- Nada importante

Você selecionou "... Muito rico em calorias"
O quanto isso é importante para você?

- Extremamente importante
- Muito importante
- Moderadamente importante
- Levemente importante
- Nada importante

Você selecionou "... Eu não devo comer isso"
O quanto isso é importante para você?

- Extremamente importante
- Muito importante
- Moderadamente importante
- Levemente importante
- Nada importante

Você selecionou "... Comer isso me faz parecer antiquado"
O quanto isso é importante para você?

- Extremamente importante
- Muito importante
- Moderadamente importante
- Levemente importante
- Nada importante

Você selecionou ".. Não é nada que eu queira comer agora"
O quanto isso é importante para você?

- Extremamente importante
- Muito importante
- Moderadamente importante
- Levemente importante
- Nada importante

Você selecionou ".. Eu comi recentemente e não quero comer a mesma comida com tanta frequência"
O quanto isso é importante para você?

- Extremamente importante
- Muito importante
- Moderadamente importante
- Levemente importante
- Nada importante

Você selecionou "... Eu nunca escolheria isso e eu gosto de comer a mesma comida todos os dias"
O quanto isso é importante para você?

- Extremamente importante
- Muito importante
- Moderadamente importante
- Levemente importante
- Nada importante

Você selecionou "... Essa comida me faz sentir triste, solitário e frustrado"
O quanto isso é importante para você?

- Extremamente importante
- Muito importante
- Moderadamente importante
- Levemente importante
- Nada importante

Você selecionou "... Não é orgânico"
O quanto isso é importante para você?

- Extremamente importante
- Muito importante
- Moderadamente importante
- Levemente importante
- Nada importante

Reasons for Fruit Choices(RATA)

Você consome banana?

- Sim
- Não

Quais as razões pelas quais você come banana? (Marque todas as respostas que correspondem)

- ☐ ... porque é saboroso
- ☐ ... porque me sacia de forma agradável
- ☐ ... porque normalmente eu como isso
- ☐ ... porque é orgânico
- ☐ ... porque me faz parecer melhor na frente dos outros
- ☐ ... porque eu curto
- ☐ ... para manter uma dieta balanceada
- ☐ ... porque é natural (exemplo, não geneticamente modificado)
- ☐ ... porque tem baixas calorias
- ☐ ... porque estou habituado
- ☐ ... porque eu não quero gastar muito dinheiro
- ☐ ... porque me mantém em forma exemplo, vigoroso, dinâmico
- ☐ ... porque é saudável
- ☐ ... porque eu devo comer
- ☐ ... porque é a única escolha
- ☐ ... porque eu preciso de energia
- ☐ ... porque estou frustrado
- ☐ ... porque é mais conveniente
- ☐ ... porque eu me sinto sozinho
- ☐ ... porque eu vejo propaganda em algum lugar ou vejo na TV
- ☐ ... para não desapontar alguém que está tentando me agradar
- ☐ ... porque eu cresci comendo isso
- ☐ ... porque me é familiar
- ☐ ... porque eu controlo meu peso
- ☐ ... porque não faz parte das tradições como Natal, Ano Novo e ocasiões especiais
- ☐ ... porque não é caro
- ☐ ... porque deixa as reuniões sociais mais agradáveis
- ☐ ... porque é sociável
- ☐ ... porque eu posso desfrutar o momento com outras pessoas

- ☐ ... porque tem pouca gordura
- ☐ ... para compensar
- ☐ ... porque me dou ao luxo
- ☐ ... porque estou faminto
- ☐ ... porque seria falta de educação não comer isso
- ☐ ... porque os outros gostam
- ☐ ... porque é fácil de preparar
- ☐ ... porque me atrai aparentemente, por exemplo cores atraentes
- ☐ ... porque não contém substâncias perigosas como agrotóxicos, poluentes, antibióticos
- ☐ ... porque eu estou triste
- ☐ ... porque faz parte de certas situações
- ☐ ... porque eu gosto
- ☐ ... porque é moda
- ☐ ... porque é rápido de preparar
- ☐ ... porque está em promoção
- ☐ ... porque eu tenho vontade de comer isso
- ☐ ... porque a apresentação é atraente, por exemplo a embalagem
- ☐ ... porque eu quero comer todos os dias

Você selecionou "... porque estou habituado "
O quanto isso é importante para você?

- ○ Extremamente importante
- ○ Muito importante
- ○ Moderadamente importante
- ○ Levemente importante
- ○ Nada importante

Você selecionou "... porque é saudável "
O quanto isso é importante para você?

- Extremamente importante
- Muito importante
- Moderadamente importante
- Levemente importante
- Nada importante

Você selecionou "... porque é rápido de preparar "
O quanto isso é importante para você?

- Extremamente importante
- Muito importante
- Moderadamente importante
- Levemente importante
- Nada importante

Você selecionou "... porque é saboroso "
O quanto isso é importante para você?

- Extremamente importante
- Muito importante
- Moderadamente importante
- Levemente importante
- Nada importante

Você selecionou "... porque me dou ao luxo "
O quanto isso é importante para você?

- Extremamente importante
- Muito importante
- Moderadamente importante
- Levemente importante
- Nada importante

Você selecionou "... porque é natural (exemplo, não geneticamente modificado) "
O quanto isso é importante para você?

- Extremamente importante
- Muito importante
- Moderadamente importante
- Levemente importante
- Nada importante

Você selecionou "... porque eu não quero gastar muito dinheiro "
O quanto isso é importante para você?

- Extremamente importante
- Muito importante
- Moderadamente importante
- Levemente importante
- Nada importante

Você selecionou "... porque tem baixas calorias "
O quanto isso é importante para você?

- Extremamente importante
- Muito importante
- Moderadamente importante
- Levemente importante
- Nada importante

Você selecionou "... porque estou frustrado "
O quanto isso é importante para você?

- Extremamente importante
- Muito importante
- Moderadamente importante
- Levemente importante
- Nada importante

Você selecionou "... porque me faz parecer melhor na frente dos outros "
O quanto isso é importante para você?

- Extremamente importante
- Muito importante
- Moderadamente importante
- Levemente importante
- Nada importante

Você selecionou "... porque seria falta de educação não comer isso "
O quanto isso é importante para você?

- Extremamente importante
- Muito importante
- Moderadamente importante
- Levemente importante
- Nada importante

Você selecionou "... porque a apresentação é atraente, por exemplo a embalagem "

O quanto isso é importante para você?

- Extremamente importante
- Muito importante
- Moderadamente importante
- Levemente importante

- Nada importante

Você selecionou "... porque eu posso desfrutar o momento com outras pessoas "
O quanto isso é importante para você?

- Extremamente importante
- Muito importante
- Moderadamente importante
- Levemente importante
- Nada importante

Você selecionou "... porque é orgânico "
O quanto isso é importante para você?

- Extremamente importante
- Muito importante
- Moderadamente importante
- Levemente importante
- Nada importante

Você selecionou "... porque eu cresci comendo isso "
O quanto isso é importante para você?

- Extremamente importante
- Muito importante
- Moderadamente importante
- Levemente importante
- Nada importante

Você selecionou "... porque eu curto "
O quanto isso é importante para você?

- Extremamente importante
- Muito importante
- Moderadamente importante
- Levemente importante
- Nada importante

Você selecionou "... porque estou faminto "
O quanto isso é importante para você?

- Extremamente importante
- Muito importante
- Moderadamente importante
- Levemente importante
- Nada importante

Você selecionou "... porque normalmente eu como isso "
O quanto isso é importante para você?

- Extremamente importante
- Muito importante
- Moderadamente importante
- Levemente importante
- Nada importante

Você selecionou "... porque eu tenho vontade de comer isso "
O quanto isso é importante para você?

- Extremamente importante
- Muito importante
- Moderadamente importante
- Levemente importante

- Nada importante

Você selecionou "... porque é fácil de preparar "
O quanto isso é importante para você?

- Extremamente importante
- Muito importante
- Moderadamente importante
- Levemente importante
- Nada importante

Você selecionou "... porque eu estou triste "
O quanto isso é importante para você?

- Extremamente importante
- Muito importante
- Moderadamente importante
- Levemente importante
- Nada importante

Você selecionou "... porque tem pouca gordura "
O quanto isso é importante para você?

- Extremamente importante
- Muito importante
- Moderadamente importante
- Levemente importante
- Nada importante

Você selecionou "... porque eu vejo propaganda em algum lugar ou vejo na TV "
O quanto isso é importante para você?

- Extremamente importante
- Muito importante
- Moderadamente importante
- Levemente importante
- Nada importante

Você selecionou "... porque deixa as reuniões sociais mais agradáveis "
O quanto isso é importante para você?

- Extremamente importante
- Muito importante
- Moderadamente importante
- Levemente importante
- Nada importante

Você selecionou "... porque eu devo comer "
O quanto isso é importante para você?

- Extremamente importante
- Muito importante
- Moderadamente importante
- Levemente importante
- Nada importante

Você selecionou "... porque os outros gostam "
O quanto isso é importante para você?

- Extremamente importante
- Muito importante
- Moderadamente importante
- Levemente importante

- Nada importante

Você selecionou "... porque eu me sinto sozinho "
O quanto isso é importante para você?

- Extremamente importante
- Muito importante
- Moderadamente importante
- Levemente importante
- Nada importante

Você selecionou "... porque eu controlo meu peso "
O quanto isso é importante para você?

- Extremamente importante
- Muito importante
- Moderadamente importante
- Levemente importante
- Nada importante

Você selecionou "... porque não contém substâncias perigosas como agrotóxicos, poluentes, antibióticos "
O quanto isso é importante para você?

- Extremamente importante
- Muito importante
- Moderadamente importante
- Levemente importante
- Nada importante

Você selecionou "... porque é mais conveniente "

O quanto isso é importante para você?

- Extremamente importante
- Muito importante
- Moderadamente importante
- Levemente importante
- Nada importante

Você selecionou "... porque eu preciso de energia "
O quanto isso é importante para você?

- Extremamente importante
- Muito importante
- Moderadamente importante
- Levemente importante
- Nada importante

Você selecionou "... porque me é familiar "
O quanto isso é importante para você?

- Extremamente importante
- Muito importante
- Moderadamente importante
- Levemente importante
- Nada importante

Você selecionou "... porque eu gosto "
O quanto isso é importante para você?

- Extremamente importante
- Muito importante
- Moderadamente importante

- Levemente importante
- Nada importante

Você selecionou "... porque me sacia de forma agradável "
O quanto isso é importante para você?

- Extremamente importante
- Muito importante
- Moderadamente importante
- Levemente importante
- Nada importante

Você selecionou "... para compensar "
O quanto isso é importante para você?

- Extremamente importante
- Muito importante
- Moderadamente importante
- Levemente importante
- Nada importante

Você selecionou "... porque não faz parte das tradições como Natal, Ano Novo e ocasiões especiais "
O quanto isso é importante para você?

- Extremamente importante
- Muito importante
- Moderadamente importante
- Levemente importante
- Nada importante

Você selecionou "... porque faz parte de certas situações "
O quanto isso é importante para você?

- Extremamente importante
- Muito importante
- Moderadamente importante
- Levemente importante
- Nada importante

Você selecionou "... para manter uma dieta balanceada "
O quanto isso é importante para você?

- Extremamente importante
- Muito importante
- Moderadamente importante
- Levemente importante
- Nada importante

Você selecionou "... porque é sociável "
O quanto isso é importante para você?

- Extremamente importante
- Muito importante
- Moderadamente importante
- Levemente importante
- Nada importante

Você selecionou "... porque não é caro "
O quanto isso é importante para você?

- Extremamente importante
- Muito importante

- Moderadamente importante
- Levemente importante
- Nada importante

Você selecionou "... porque me atrai aparentemente, por exemplo cores atraentes "

O quanto isso é importante para você?

- Extremamente importante
- Muito importante
- Moderadamente importante
- Levemente importante
- Nada importante

Você selecionou "... para não desapontar alguém que está tentando me agradar "
O quanto isso é importante para você?

- Extremamente importante
- Muito importante
- Moderadamente importante
- Levemente importante
- Nada importante

Você selecionou "... porque é moda "
O quanto isso é importante para você?

- Extremamente importante
- Muito importante
- Moderadamente importante
- Levemente importante
- Nada importante

Você selecionou "... porque está em promoção "
O quanto isso é importante para você?

- Extremamente importante
- Muito importante
- Moderadamente importante
- Levemente importante
- Nada importante

Você selecionou "... porque me mantém em forma exemplo, vigoroso, dinâmico "
O quanto isso é importante para você?

- Extremamente importante
- Muito importante
- Moderadamente importante
- Levemente importante
- Nada importante

Você selecionou "... porque é a única escolha "
O quanto isso é importante para você?

- Extremamente importante
- Muito importante
- Moderadamente importante
- Levemente importante
- Nada importante

Você selecionou "... porque eu quero comer todos os dias "
O quanto isso é importante para você?

- Extremamente importante

- Muito importante
- Moderadamente importante
- Levemente importante
- Nada importante

Quais as razões pelas quais você não come banana? (Marque todas as respostas que correspondem)

- ... O preço está muito alto
- ... Não é orgânico
- ... Não é saudável
- ... Não sustenta o suficiente
- ... Eu não quero me dar ao luxo
- ... Muito rico em calorias
- ... Eu não gosto do jeito que parece
- ... Não é conveniente
- ... Eu normalmente não como isso
- ... Eu não acho que seja um lanche
- ... Não é apropriado para a situação
- ... Essa comida me faz sentir triste, solitário e frustrado
- ... Eu não gosto disso
- ... Eu não devo comer isso
- .. Eu comi recentemente e não quero comer a mesma comida com tanta frequência
- ... O tamanho da porção não é adequado
- .. Não é nada que eu queira comer agora
- ... Eu nunca escolheria isso e eu gosto de comer a mesma comida todos os dias
- ... Não é um alimento que eu como perto de outras pessoas
- ... Comer isso me faz parecer antiquado

Você selecionou "... Eu não acho que seja um lanche"
O quanto isso é importante para você?

- Extremamente importante
- Muito importante
- Moderadamente importante
- Levemente importante
- Nada importante

Você selecionou "... Eu não gosto disso"
O quanto isso é importante para você?

- Extremamente importante
- Muito importante
- Moderadamente importante
- Levemente importante
- Nada importante

Você selecionou "... Eu normalmente não como isso"
O quanto isso é importante para você?

- Extremamente importante
- Muito importante
- Moderadamente importante
- Levemente importante
- Nada importante

Você selecionou "... O tamanho da porção não é adequado"
O quanto isso é importante para você?

- Extremamente importante
- Muito importante
- Moderadamente importante
- Levemente importante

- Nada importante

Você selecionou "... Não sustenta o suficiente"
O quanto isso é importante para você?

- Extremamente importante
- Muito importante
- Moderadamente importante
- Levemente importante
- Nada importante

Você selecionou "... Não é saudável"
O quanto isso é importante para você?

- Extremamente importante
- Muito importante
- Moderadamente importante
- Levemente importante
- Nada importante

Você selecionou "... Não é conveniente"
O quanto isso é importante para você?

- Extremamente importante
- Muito importante
- Moderadamente importante
- Levemente importante
- Nada importante

Você selecionou "... Eu não quero me dar ao luxo"
O quanto isso é importante para você?

- Extremamente importante
- Muito importante
- Moderadamente importante
- Levemente importante
- Nada importante

Você selecionou "... Não é apropriado para a situação"
O quanto isso é importante para você?

- Extremamente importante
- Muito importante
- Moderadamente importante
- Levemente importante
- Nada importante

Você selecionou "... Não é um alimento que eu como perto de outras pessoas"
O quanto isso é importante para você?

- Extremamente importante
- Muito importante
- Moderadamente importante
- Levemente importante
- Nada importante

Você selecionou "... O preço está muito alto"
O quanto isso é importante para você?

- Extremamente importante
- Muito importante
- Moderadamente importante
- Levemente importante

- Nada importante

Você selecionou "... Eu não gosto do jeito que parece"
O quanto isso é importante para você?

- Extremamente importante
- Muito importante
- Moderadamente importante
- Levemente importante
- Nada importante

Você selecionou "... Muito rico em calorias"
O quanto isso é importante para você?

- Extremamente importante
- Muito importante
- Moderadamente importante
- Levemente importante
- Nada importante

Você selecionou "... Eu não devo comer isso"
O quanto isso é importante para você?

- Extremamente importante
- Muito importante
- Moderadamente importante
- Levemente importante
- Nada importante

Você selecionou "... Comer isso me faz parecer antiquado"
O quanto isso é importante para você?

- Extremamente importante
- Muito importante
- Moderadamente importante
- Levemente importante
- Nada importante

Você selecionou ".. Não é nada que eu queira comer agora"
O quanto isso é importante para você?

- Extremamente importante
- Muito importante
- Moderadamente importante
- Levemente importante
- Nada importante

Você selecionou ".. Eu comi recentemente e não quero comer a mesma comida com tanta frequência"
O quanto isso é importante para você?

- Extremamente importante
- Muito importante
- Moderadamente importante
- Levemente importante
- Nada importante

Você selecionou "... Eu nunca escolheria isso e eu gosto de comer a mesma comida todos os dias"
O quanto isso é importante para você?

- Extremamente importante
- Muito importante
- Moderadamente importante

- Levemente importante
- Nada importante

Você selecionou "... Essa comida me faz sentir triste, solitário e frustrado"
O quanto isso é importante para você?

- Extremamente importante
- Muito importante
- Moderadamente importante
- Levemente importante
- Nada importante

Você selecionou "... Não é orgânico"
O quanto isso é importante para você?

- Extremamente importante
- Muito importante
- Moderadamente importante
- Levemente importante
- Nada importante

Reasons for Sweet food Choices(RATA)

Você consome brigadeiro?

- Sim
- Não

Quais as razões pelas quais você come brigadeiro? (Marque todas as respostas que correspondem)

- ☐ ... porque está em promoção
- ☐ ... porque tem pouca gordura
- ☐ ... porque estou faminto
- ☐ ... porque tem baixas calorias
- ☐ ... porque estou habituado
- ☐ ... porque me mantém em forma exemplo, vigoroso, dinâmico
- ☐ ... porque os outros gostam
- ☐ ... porque é orgânico
- ☐ ... porque é saudável
- ☐ ... porque estou frustrado
- ☐ ... porque seria falta de educação não comer isso
- ☐ ... porque eu preciso de energia
- ☐ ... para manter uma dieta balanceada
- ☐ ... porque eu não quero gastar muito dinheiro
- ☐ ... porque é saboroso
- ☐ ... porque eu cresci comendo isso
- ☐ ... porque normalmente eu como isso
- ☐ ... porque eu posso desfrutar o momento com outras pessoas
- ☐ ... porque me atrai aparentemente, por exemplo cores atraentes
- ☐ ... porque eu controlo meu peso
- ☐ ... porque é rápido de preparar
- ☐ ... para compensar
- ☐ ... porque não é caro
- ☐ ... porque eu devo comer
- ☐ ... para não desapontar alguém que está tentando me agradar
- ☐ ... porque é a única escolha
- ☐ ... porque não faz parte das tradições como Natal, Ano Novo e ocasiões especiais
- ☐ ... porque me sacia de forma agradável
- ☐ ... porque é natural (exemplo, não geneticamente modificado)
- ☐ ... porque me faz parecer melhor na frente dos outros
- ☐ ... porque deixa as reuniões sociais mais agradáveis
- ☐ ... porque a apresentação é atraente, por exemplo a embalagem

- ☐ ... porque é mais conveniente
- ☐ ... porque eu quero comer todos os dias
- ☐ ... porque eu gosto
- ☐ ... porque eu curto
- ☐ ... porque eu tenho vontade de comer isso
- ☐ ... porque é fácil de preparar
- ☐ ... porque faz parte de certas situações
- ☐ ... porque é sociável
- ☐ ... porque eu me sinto sozinho
- ☐ ... porque eu estou triste
- ☐ ... porque me dou ao luxo
- ☐ ... porque eu vejo propaganda em algum lugar ou vejo na TV
- ☐ ... porque não contém substâncias perigosas como agrotóxicos, poluentes, antibióticos
- ☐ ... porque é moda
- ☐ ... porque me é familiar

Você selecionou "... porque estou habituado "
O quanto isso é importante para você?

- ○ Extremamente importante
- ○ Muito importante
- ○ Moderadamente importante
- ○ Levemente importante
- ○ Nada importante

Você selecionou "... porque é saudável "
O quanto isso é importante para você?

- ○ Extremamente importante
- ○ Muito importante
- ○ Moderadamente importante

- Levemente importante
- Nada importante

Você selecionou "... porque é rápido de preparar "
O quanto isso é importante para você?

- Extremamente importante
- Muito importante
- Moderadamente importante
- Levemente importante
- Nada importante

Você selecionou "... porque é saboroso "
O quanto isso é importante para você?

- Extremamente importante
- Muito importante
- Moderadamente importante
- Levemente importante
- Nada importante

Você selecionou "... porque me dou ao luxo "
O quanto isso é importante para você?

- Extremamente importante
- Muito importante
- Moderadamente importante
- Levemente importante
- Nada importante

Você selecionou "... porque é natural (exemplo, não geneticamente modificado) "

O quanto isso é importante para você?

- Extremamente importante
- Muito importante
- Moderadamente importante
- Levemente importante
- Nada importante

Você selecionou "... porque eu não quero gastar muito dinheiro "
O quanto isso é importante para você?

- Extremamente importante
- Muito importante
- Moderadamente importante
- Levemente importante
- Nada importante

Você selecionou "... porque tem baixas calorias "
O quanto isso é importante para você?

- Extremamente importante
- Muito importante
- Moderadamente importante
- Levemente importante
- Nada importante

Você selecionou "... porque estou frustrado "
O quanto isso é importante para você?

- Extremamente importante
- Muito importante
- Moderadamente importante

- Levemente importante
- Nada importante

Você selecionou "... porque me faz parecer melhor na frente dos outros "
O quanto isso é importante para você?

- Extremamente importante
- Muito importante
- Moderadamente importante
- Levemente importante
- Nada importante

Você selecionou "... porque seria falta de educação não comer isso "
O quanto isso é importante para você?

- Extremamente importante
- Muito importante
- Moderadamente importante
- Levemente importante
- Nada importante

Você selecionou "... porque a apresentação é atraente, por exemplo a embalagem "
O quanto isso é importante para você?

- Extremamente importante
- Muito importante
- Moderadamente importante
- Levemente importante
- Nada importante

Você selecionou "... porque eu posso desfrutar o momento com outras pessoas "
O quanto isso é importante para você?

- Extremamente importante
- Muito importante
- Moderadamente importante
- Levemente importante
- Nada importante

Você selecionou "... porque é orgânico "
O quanto isso é importante para você?

- Extremamente importante
- Muito importante
- Moderadamente importante
- Levemente importante
- Nada importante

Você selecionou "... porque eu cresci comendo isso "
O quanto isso é importante para você?

- Extremamente importante
- Muito importante
- Moderadamente importante
- Levemente importante
- Nada importante

Você selecionou "... porque eu curto "
O quanto isso é importante para você?

- Extremamente importante
- Muito importante

- Moderadamente importante
- Levemente importante
- Nada importante

Você selecionou "... porque estou faminto "
O quanto isso é importante para você?

- Extremamente importante
- Muito importante
- Moderadamente importante
- Levemente importante
- Nada importante

Você selecionou "... porque normalmente eu como isso "
O quanto isso é importante para você?

- Extremamente importante
- Muito importante
- Moderadamente importante
- Levemente importante
- Nada importante

Você selecionou "... porque eu tenho vontade de comer isso "
O quanto isso é importante para você?

- Extremamente importante
- Muito importante
- Moderadamente importante
- Levemente importante
- Nada importante

Você selecionou "... porque é fácil de preparar "
O quanto isso é importante para você?

- Extremamente importante
- Muito importante
- Moderadamente importante
- Levemente importante
- Nada importante

Você selecionou "... porque eu estou triste "
O quanto isso é importante para você?

- Extremamente importante
- Muito importante
- Moderadamente importante
- Levemente importante
- Nada importante

Você selecionou "... porque tem pouca gordura "
O quanto isso é importante para você?

- Extremamente importante
- Muito importante
- Moderadamente importante
- Levemente importante
- Nada importante

Você selecionou "... porque eu vejo propaganda em algum lugar ou vejo na TV "
O quanto isso é importante para você?

- Extremamente importante
- Muito importante

- Moderadamente importante
- Levemente importante
- Nada importante

Você selecionou "... porque deixa as reuniões sociais mais agradáveis "
O quanto isso é importante para você?

- Extremamente importante
- Muito importante
- Moderadamente importante
- Levemente importante
- Nada importante

Você selecionou "... porque eu devo comer "
O quanto isso é importante para você?

- Extremamente importante
- Muito importante
- Moderadamente importante
- Levemente importante
- Nada importante

Você selecionou "... porque os outros gostam "
O quanto isso é importante para você?

- Extremamente importante
- Muito importante
- Moderadamente importante
- Levemente importante
- Nada importante

Você selecionou "... porque eu me sinto sozinho "
O quanto isso é importante para você?

- Extremamente importante
- Muito importante
- Moderadamente importante
- Levemente importante
- Nada importante

Você selecionou "... porque eu controlo meu peso "
O quanto isso é importante para você?

- Extremamente importante
- Muito importante
- Moderadamente importante
- Levemente importante
- Nada importante

Você selecionou "... porque não contém substâncias perigosas como agrotóxicos, poluentes, antibióticos "
O quanto isso é importante para você?

- Extremamente importante
- Muito importante
- Moderadamente importante
- Levemente importante
- Nada importante

Você selecionou "... porque é mais conveniente "
O quanto isso é importante para você?

- Extremamente importante

- Muito importante
- Moderadamente importante
- Levemente importante
- Nada importante

Você selecionou "... porque eu preciso de energia "
O quanto isso é importante para você?

- Extremamente importante
- Muito importante
- Moderadamente importante
- Levemente importante
- Nada importante

Você selecionou "... porque me é familiar "
O quanto isso é importante para você?

- Extremamente importante
- Muito importante
- Moderadamente importante
- Levemente importante
- Nada importante

Você selecionou "... porque eu gosto "
O quanto isso é importante para você?

- Extremamente importante
- Muito importante
- Moderadamente importante
- Levemente importante
- Nada importante

Você selecionou "... porque me sacia de forma agradável "
O quanto isso é importante para você?

- Extremamente importante
- Muito importante
- Moderadamente importante
- Levemente importante
- Nada importante

Você selecionou "... para compensar "
O quanto isso é importante para você?

- Extremamente importante
- Muito importante
- Moderadamente importante
- Levemente importante
- Nada importante

Você selecionou "... porque não faz parte das tradições como Natal, Ano Novo e ocasiões especiais "
O quanto isso é importante para você?

- Extremamente importante
- Muito importante
- Moderadamente importante
- Levemente importante
- Nada importante

Você selecionou "... porque faz parte de certas situações "
O quanto isso é importante para você?

- Extremamente importante

- Muito importante
- Moderadamente importante
- Levemente importante
- Nada importante

Você selecionou "... para manter uma dieta balanceada "
O quanto isso é importante para você?

- Extremamente importante
- Muito importante
- Moderadamente importante
- Levemente importante
- Nada importante

Você selecionou "... porque é sociável "
O quanto isso é importante para você?

- Extremamente importante
- Muito importante
- Moderadamente importante
- Levemente importante
- Nada importante

Você selecionou "... porque não é caro "
O quanto isso é importante para você?

- Extremamente importante
- Muito importante
- Moderadamente importante
- Levemente importante
- Nada importante

Você selecionou "... porque me atrai aparentemente, por exemplo cores atraentes "

O quanto isso é importante para você?

- Extremamente importante
- Muito importante
- Moderadamente importante
- Levemente importante
- Nada importante

Você selecionou "... para não desapontar alguém que está tentando me agradar "
O quanto isso é importante para você?

- Extremamente importante
- Muito importante
- Moderadamente importante
- Levemente importante
- Nada importante

Você selecionou "... porque é moda "
O quanto isso é importante para você?

- Extremamente importante
- Muito importante
- Moderadamente importante
- Levemente importante
- Nada importante

Você selecionou "... porque está em promoção "
O quanto isso é importante para você?

- Extremamente importante

- Muito importante
- Moderadamente importante
- Levemente importante
- Nada importante

Você selecionou "... porque me mantém em forma exemplo, vigoroso, dinâmico "
O quanto isso é importante para você?

- Extremamente importante
- Muito importante
- Moderadamente importante
- Levemente importante
- Nada importante

Você selecionou "... porque é a única escolha "
O quanto isso é importante para você?

- Extremamente importante
- Muito importante
- Moderadamente importante
- Levemente importante
- Nada importante

Você selecionou "... porque eu quero comer todos os dias "
O quanto isso é importante para você?

- Extremamente importante
- Muito importante
- Moderadamente importante
- Levemente importante
- Nada importante

Quais as razões pelas quais você não come brigadeiro? (Marque todas as respostas que correspondem)

- ☐ ... Comer isso me faz parecer antiquado
- ☐ ... Eu não gosto do jeito que parece
- ☐ ... Eu nunca escolheria isso e eu gosto de comer a mesma comida todos os dias
- ☐ .. Não é nada que eu queira comer agora
- ☐ ... Eu não quero me dar ao luxo
- ☐ .. Eu comi recentemente e não quero comer a mesma comida com tanta frequência
- ☐ ... Essa comida me faz sentir triste, solitário e frustrado
- ☐ ... Muito rico em calorias
- ☐ ... Eu normalmente não como isso
- ☐ ... Não é saudável
- ☐ ... O preço está muito alto
- ☐ ... Não sustenta o suficiente
- ☐ ... Eu não devo comer isso
- ☐ ... Não é apropriado para a situação
- ☐ ... Não é um alimento que eu como perto de outras pessoas
- ☐ ... Não é conveniente
- ☐ ... Eu não acho que seja um lanche
- ☐ ... O tamanho da porção não é adequado
- ☐ ... Não é orgânico
- ☐ ... Eu não gosto disso

Você selecionou "... Eu não acho que seja um lanche"
O quanto isso é importante para você?

- ○ Extremamente importante
- ○ Muito importante
- ○ Moderadamente importante
- ○ Levemente importante

- Nada importante

Você selecionou "... Eu não gosto disso"
O quanto isso é importante para você?

- Extremamente importante
- Muito importante
- Moderadamente importante
- Levemente importante
- Nada importante

Você selecionou "... Eu normalmente não como isso"
O quanto isso é importante para você?

- Extremamente importante
- Muito importante
- Moderadamente importante
- Levemente importante
- Nada importante

Você selecionou "... O tamanho da porção não é adequado"
O quanto isso é importante para você?

- Extremamente importante
- Muito importante
- Moderadamente importante
- Levemente importante
- Nada importante

Você selecionou "... Não sustenta o suficiente"
O quanto isso é importante para você?

- Extremamente importante
- Muito importante
- Moderadamente importante
- Levemente importante
- Nada importante

Você selecionou "... Não é saudável"
O quanto isso é importante para você?

- Extremamente importante
- Muito importante
- Moderadamente importante
- Levemente importante
- Nada importante

Você selecionou "... Não é conveniente"
O quanto isso é importante para você?

- Extremamente importante
- Muito importante
- Moderadamente importante
- Levemente importante
- Nada importante

Você selecionou "... Eu não quero me dar ao luxo"
O quanto isso é importante para você?

- Extremamente importante
- Muito importante
- Moderadamente importante
- Levemente importante

- Nada importante

Você selecionou "... Não é apropriado para a situação"
O quanto isso é importante para você?

- Extremamente importante
- Muito importante
- Moderadamente importante
- Levemente importante
- Nada importante

Você selecionou "... Não é um alimento que eu como perto de outras pessoas"
O quanto isso é importante para você?

- Extremamente importante
- Muito importante
- Moderadamente importante
- Levemente importante
- Nada importante

Você selecionou "... O preço está muito alto"
O quanto isso é importante para você?

- Extremamente importante
- Muito importante
- Moderadamente importante
- Levemente importante
- Nada importante

Você selecionou "... Eu não gosto do jeito que parece"
O quanto isso é importante para você?

- Extremamente importante
- Muito importante
- Moderadamente importante
- Levemente importante
- Nada importante

Você selecionou "... Muito rico em calorias"
O quanto isso é importante para você?

- Extremamente importante
- Muito importante
- Moderadamente importante
- Levemente importante
- Nada importante

Você selecionou "... Eu não devo comer isso"
O quanto isso é importante para você?

- Extremamente importante
- Muito importante
- Moderadamente importante
- Levemente importante
- Nada importante

Você selecionou "... Comer isso me faz parecer antiquado"
O quanto isso é importante para você?

- Extremamente importante
- Muito importante
- Moderadamente importante
- Levemente importante

- Nada importante

Você selecionou ".. Não é nada que eu queira comer agora"
O quanto isso é importante para você?

- Extremamente importante
- Muito importante
- Moderadamente importante
- Levemente importante
- Nada importante

Você selecionou ".. Eu comi recentemente e não quero comer a mesma comida com tanta frequência"
O quanto isso é importante para você?

- Extremamente importante
- Muito importante
- Moderadamente importante
- Levemente importante
- Nada importante

Você selecionou "... Eu nunca escolheria isso e eu gosto de comer a mesma comida todos os dias"
O quanto isso é importante para você?

- Extremamente importante
- Muito importante
- Moderadamente importante
- Levemente importante
- Nada importante

Você selecionou "... Essa comida me faz sentir triste, solitário e frustrado"
O quanto isso é importante para você?

- Extremamente importante
- Muito importante
- Moderadamente importante
- Levemente importante
- Nada importante

Você selecionou "... Não é orgânico"
O quanto isso é importante para você?

- Extremamente importante
- Muito importante
- Moderadamente importante
- Levemente importante
- Nada importante

Reasons for Starchy food choices(Full RATA)

Você consome arroz?

- Sim
- Não

Pense sobre as razões pelas quais você consome arroz.
O quanto essa afirmação é importante para você "...porque estou a acostumado a comer isso"

- Extremamente importante

- Muito importante
- Moderadamente importante
- Levemente importante
- Nada importante

Pense sobre as razões pelas quais você consome arroz.
O quanto essa afirmação é importante para você "...porque é saudável"

- Extremamente importante
- Muito importante
- Moderadamente importante
- Levemente importante
- Nada importante

Pense sobre as razões pelas quais você consome arroz.
O quanto essa afirmação é importante para você "...porque é rápido de preparar"

- Extremamente importante
- Muito importante
- Moderadamente importante
- Levemente importante
- Nada importante

Pense sobre as razões pelas quais você consome arroz.
O quanto essa afirmação é importante para você "...porque é saboroso"

- Extremamente importante
- Muito importante
- Moderadamente importante
- Levemente importante
- Nada importante

Pense sobre as razões pelas quais você consome arroz.
O quanto essa afirmação é importante para você "...porque eu me dou ao luxo"

- Extremamente importante
- Muito importante
- Moderadamente importante
- Levemente importante
- Nada importante

Pense sobre as razões pelas quais você consome arroz.
O quanto essa afirmação é importante para você "...porque é natural (exemplo, não geneticamente modificado)"

- Extremamente importante
- Muito importante
- Moderadamente importante
- Levemente importante
- Nada importante

Pense sobre as razões pelas quais você consome arroz.
O quanto essa afirmação é importante para você "...porque eu não quero gastar muito"

- Extremamente importante
- Muito importante
- Moderadamente importante
- Levemente importante
- Nada importante

Pense sobre as razões pelas quais você consome arroz.
O quanto essa afirmação é importante para você "...porque tem baixas calorias"

- Extremamente importante
- Muito importante
- Moderadamente importante
- Levemente importante
- Nada importante

Pense sobre as razões pelas quais você consome arroz.
O quanto essa afirmação é importante para você "...porque estou frustrado"

- Extremamente importante
- Muito importante
- Moderadamente importante
- Levemente importante
- Nada importante

Pense sobre as razões pelas quais você consome arroz.
O quanto essa afirmação é importante para você "...porque me faz parecer melhor na frente dos outros "

- Extremamente importante
- Muito importante
- Moderadamente importante
- Levemente importante
- Nada importante

Pense sobre as razões pelas quais você consome arroz.
O quanto essa afirmação é importante para você "...porque seria falta de educação não comer "

- Extremamente importante
- Muito importante
- Moderadamente importante

- Levemente importante
- Nada importante

Pense sobre as razões pelas quais você consome arroz.
O quanto essa afirmação é importante para você "...porque a apresentação é atraente (por exemplo a embalagem)"

- Extremamente importante
- Muito importante
- Moderadamente importante
- Levemente importante
- Nada importante

Pense sobre as razões pelas quais você consome arroz.
O quanto essa afirmação é importante para você "...porque eu posso desfrutar o momento com outras pessoas"

- Extremamente importante
- Muito importante
- Moderadamente importante
- Levemente importante
- Nada importante

Pense sobre as razões pelas quais você consome arroz.
O quanto essa afirmação é importante para você "...porque é orgânico"

- Extremamente importante
- Muito importante
- Moderadamente importante
- Levemente importante
- Nada importante

Pense sobre as razões pelas quais você consome arroz.
O quanto essa afirmação é importante para você "...porque eu cresci comendo isso"

- Extremamente importante
- Muito importante
- Moderadamente importante
- Levemente importante
- Nada importante

Pense sobre as razões pelas quais você consome arroz.
O quanto essa afirmação é importante para você "...porque eu curto"

- Extremamente importante
- Muito importante
- Moderadamente importante
- Levemente importante
- Nada importante

Pense sobre as razões pelas quais você consome arroz.
O quanto essa afirmação é importante para você "...porque eu estou faminto"

- Extremamente importante
- Muito importante
- Moderadamente importante
- Levemente importante
- Nada importante

Pense sobre as razões pelas quais você consome arroz.
O quanto essa afirmação é importante para você "...porque eu tenho o hábito de comer"

- Extremamente importante
- Muito importante
- Moderadamente importante
- Levemente importante
- Nada importante

Pense sobre as razões pelas quais você consome arroz.
O quanto essa afirmação é importante para você "...porque eu tenho vontade de comer isso"

- Extremamente importante
- Muito importante
- Moderadamente importante
- Levemente importante
- Nada importante

Pense sobre as razões pelas quais você consome arroz.
O quanto essa afirmação é importante para você "...porque é fácil preparar"

- Extremamente importante
- Muito importante
- Moderadamente importante
- Levemente importante
- Nada importante

Pense sobre as razões pelas quais você consome arroz.
O quanto essa afirmação é importante para você "...porque eu estou triste"

- Extremamente importante
- Muito importante
- Moderadamente importante
- Levemente importante

- Nada importante

Pense sobre as razões pelas quais você consome arroz.
O quanto essa afirmação é importante para você "...porque tem pouca gordura"

- Extremamente importante
- Muito importante
- Moderadamente importante
- Levemente importante
- Nada importante

Pense sobre as razões pelas quais você consome arroz.
O quanto essa afirmação é importante para você "...porque eu vi propaganda em algum lugar ou vi na TV"

- Extremamente importante
- Muito importante
- Moderadamente importante
- Levemente importante
- Nada importante

Pense sobre as razões pelas quais você consome arroz.
O quanto essa afirmação é importante para você "...porque deixa as reuniões sociais mais agradáveis"

- Extremamente importante
- Muito importante
- Moderadamente importante
- Levemente importante
- Nada importante

Pense sobre as razões pelas quais você consome arroz.
O quanto essa afirmação é importante para você "...porque eu devo comer"

- Extremamente importante
- Muito importante
- Moderadamente importante
- Levemente importante
- Nada importante

Pense sobre as razões pelas quais você consome arroz.
O quanto essa afirmação é importante para você "...porque os outros gostam"

- Extremamente importante
- Muito importante
- Moderadamente importante
- Levemente importante
- Nada importante

Pense sobre as razões pelas quais você consome arroz.
O quanto essa afirmação é importante para você "...porque eu me sinto sozinho"

- Extremamente importante
- Muito importante
- Moderadamente importante
- Levemente importante
- Nada importante

Pense sobre as razões pelas quais você consome arroz.
O quanto essa afirmação é importante para você "...porque eu controlo meu peso"

- Extremamente importante
- Muito importante

- Moderadamente importante
- Levemente importante
- Nada importante

Pense sobre as razões pelas quais você consome arroz.
O quanto essa afirmação é importante para você "...porque não contém substâncias perigosas (por exemplo, agrotóxicos, poluentes, antibióticos)"

- Extremamente importante
- Muito importante
- Moderadamente importante
- Levemente importante
- Nada importante

Pense sobre as razões pelas quais você consome arroz.
O quanto essa afirmação é importante para você "...porque é mais conveniente"

- Extremamente importante
- Muito importante
- Moderadamente importante
- Levemente importante
- Nada importante

Pense sobre as razões pelas quais você consome arroz.
O quanto essa afirmação é importante para você "...porque eu preciso de energia"

- Extremamente importante
- Muito importante
- Moderadamente importante
- Levemente importante
- Nada importante

Pense sobre as razões pelas quais você consome arroz.
O quanto essa afirmação é importante para você "...porque me é familiar"

- Extremamente importante
- Muito importante
- Moderadamente importante
- Levemente importante
- Nada importante

Pense sobre as razões pelas quais você consome arroz.
O quanto essa afirmação é importante para você "...porque eu gosto"

- Extremamente importante
- Muito importante
- Moderadamente importante
- Levemente importante
- Nada importante

Pense sobre as razões pelas quais você consome arroz.
O quanto essa afirmação é importante para você "...porque me sacia de forma agradável"

- Extremamente importante
- Muito importante
- Moderadamente importante
- Levemente importante
- Nada importante

Pense sobre as razões pelas quais você consome arroz.
O quanto essa afirmação é importante para você "...para compensar"

- Extremamente importante

- Muito importante
- Moderadamente importante
- Levemente importante
- Nada importante

Pense sobre as razões pelas quais você consome arroz.
O quanto essa afirmação é importante para você "...porque não faz parte das tradições como Natal, Ano Novo e ocasiões especiais"

- Extremamente importante
- Muito importante
- Moderadamente importante
- Levemente importante
- Nada importante

Pense sobre as razões pelas quais você consome arroz.
O quanto essa afirmação é importante para você "...porque faz parte de certas situações"

- Extremamente importante
- Muito importante
- Moderadamente importante
- Levemente importante
- Nada importante

Pense sobre as razões pelas quais você consome arroz.
O quanto essa afirmação é importante para você "...para manter uma dieta balanceada"

- Extremamente importante
- Muito importante
- Moderadamente importante

- Levemente importante
- Nada importante

Pense sobre as razões pelas quais você consome arroz.
O quanto essa afirmação é importante para você "...porque é sociável"

- Extremamente importante
- Muito importante
- Moderadamente importante
- Levemente importante
- Nada importante

Pense sobre as razões pelas quais você consome arroz.
O quanto essa afirmação é importante para você "...porque não é caro"

- Extremamente importante
- Muito importante
- Moderadamente importante
- Levemente importante
- Nada importante

Pense sobre as razões pelas quais você consome arroz.
O quanto essa afirmação é importante para você "...porque me atraiu aparentemente (por exemplo, cores atraentes)"

- Extremamente importante
- Muito importante
- Moderadamente importante
- Levemente importante
- Nada importante

Pense sobre as razões pelas quais você consome arroz.
O quanto essa afirmação é importante para você "...para não desapontar alguém que está tentando me agradar"

- Extremamente importante
- Muito importante
- Moderadamente importante
- Levemente importante
- Nada importante

Pense sobre as razões pelas quais você consome arroz.
O quanto essa afirmação é importante para você "...porque é moda"

- Extremamente importante
- Muito importante
- Moderadamente importante
- Levemente importante
- Nada importante

Pense sobre as razões pelas quais você consome arroz.
O quanto essa afirmação é importante para você "...porque está em promoção"

- Extremamente importante
- Muito importante
- Moderadamente importante
- Levemente importante
- Nada importante

Pense sobre as razões pelas quais você consome arroz.
O quanto essa afirmação é importante para você "...porque me mantém em forma (por exemplo, vigoroso, dinâmico)"

- Extremamente importante
- Muito importante
- Moderadamente importante
- Levemente importante
- Nada importante

Pense sobre as razões pelas quais você consome arroz.
O quanto essa afirmação é importante para você "...porque é a única escolha"

- Extremamente importante
- Muito importante
- Moderadamente importante
- Levemente importante
- Nada importante

Pense sobre as razões pelas quais você consome arroz.
O quanto essa afirmação é importante para você "...porque eu quero comer todos os dias"

- Extremamente importante
- Muito importante
- Moderadamente importante
- Levemente importante
- Nada importante

Pense sobre as razões pelas quais você não consome arroz.
O quanto essa afirmação é importante para você "...eu não acho que seja um lanche"

- Extremamente importante
- Muito importante
- Moderadamente importante

- Levemente importante
- Nada importante

Pense sobre as razões pelas quais você não consome arroz.
O quanto essa afirmação é importante para você "...eu não gosto disso"

- Extremamente importante
- Muito importante
- Moderadamente importante
- Levemente importante
- Nada importante

Pense sobre as razões pelas quais você não consome arroz.
O quanto essa afirmação é importante para você "...normalmente eu não como isso"

- Extremamente importante
- Muito importante
- Moderadamente importante
- Levemente importante
- Nada importante

Pense sobre as razões pelas quais você não consome arroz.
O quanto essa afirmação é importante para você "...o tamanho da porção não é adequado"

- Extremamente importante
- Muito importante
- Moderadamente importante
- Levemente importante
- Nada importante

Pense sobre as razões pelas quais você não consome arroz.
O quanto essa afirmação é importante para você "...não sustenta o suficiente"

- Extremamente importante
- Muito importante
- Moderadamente importante
- Levemente importante
- Nada importante

Pense sobre as razões pelas quais você não consome arroz.
O quanto essa afirmação é importante para você "...não é saudável"

- Extremamente importante
- Muito importante
- Moderadamente importante
- Levemente importante
- Nada importante

Pense sobre as razões pelas quais você não consome arroz.
O quanto essa afirmação é importante para você "...não é conveniente"

- Extremamente importante
- Muito importante
- Moderadamente importante
- Levemente importante
- Nada importante

Pense sobre as razões pelas quais você não consome arroz.
O quanto essa afirmação é importante para você "...eu não quero me dar ao luxo"

- Extremamente importante
- Muito importante

- Moderadamente importante
- Levemente importante
- Nada importante

Pense sobre as razões pelas quais você não consome arroz.
O quanto essa afirmação é importante para você "...não é apropriado para a situação"

- Extremamente importante
- Muito importante
- Moderadamente importante
- Levemente importante
- Nada importante

Pense sobre as razões pelas quais você não consome arroz.
O quanto essa afirmação é importante para você "...não é um alimento que eu como perto de outras pessoas"

- Extremamente importante
- Muito importante
- Moderadamente importante
- Levemente importante
- Nada importante

Pense sobre as razões pelas quais você não consome arroz.
O quanto essa afirmação é importante para você "...o preço está muito alto"

- Extremamente importante
- Muito importante
- Moderadamente importante
- Levemente importante
- Nada importante

Pense sobre as razões pelas quais você não consome arroz.
O quanto essa afirmação é importante para você "...eu não gosto do jeito que parece"

- Extremamente importante
- Muito importante
- Moderadamente importante
- Levemente importante
- Nada importante

Pense sobre as razões pelas quais você não consome arroz.
O quanto essa afirmação é importante para você "...muito rico em calorias"

- Extremamente importante
- Muito importante
- Moderadamente importante
- Levemente importante
- Nada importante

Pense sobre as razões pelas quais você não consome arroz.
O quanto essa afirmação é importante para você "...eu não devo comer isso"

- Extremamente importante
- Muito importante
- Moderadamente importante
- Levemente importante
- Nada importante

Pense sobre as razões pelas quais você não consome arroz.

O quanto essa afirmação é importante para você "...comer isso me faz parecer antiquado"

- Extremamente importante
- Muito importante
- Moderadamente importante
- Levemente importante
- Nada importante

Pense sobre as razões pelas quais você não consome arroz.
O quanto essa afirmação é importante para você "...não é nada que eu queira comer agora"

- Extremamente importante
- Muito importante
- Moderadamente importante
- Levemente importante
- Nada importante

Pense sobre as razões pelas quais você não consome arroz.
O quanto essa afirmação é importante para você "...eu comi recentemente e não quero comer a mesma comida com tanta frequência"

- Extremamente importante
- Muito importante
- Moderadamente importante
- Levemente importante
- Nada importante

Pense sobre as razões pelas quais você não consome arroz.
O quanto essa afirmação é importante para você "...eu nunca escolheria isso porque eu gosto de comer a mesma comida todos os dias"

- Extremamente importante
- Muito importante
- Moderadamente importante
- Levemente importante
- Nada importante

Pense sobre as razões pelas quais você não consome arroz.
O quanto essa afirmação é importante para você "...essa comida me faz sentir triste, solitário e frustrado"

- Extremamente importante
- Muito importante
- Moderadamente importante
- Levemente importante
- Nada importante

Pense sobre as razões pelas quais você não consome arroz.
O quanto essa afirmação é importante para você "...não é orgânico"

- Extremamente importante
- Muito importante
- Moderadamente importante
- Levemente importante
- Nada importante

Reasons for Protein foods Choices(Full RATA)

Você consome feijão?

- Sim

- Não

Pense sobre as razões pelas quais você consome feijão.
O quanto essa afirmação é importante para você "...porque estou a acostumado a comer isso"

- Extremamente importante
- Muito importante
- Moderadamente importante
- Levemente importante
- Nada importante

Pense sobre as razões pelas quais você consome feijão.
O quanto essa afirmação é importante para você "...porque é saudável"

- Extremamente importante
- Muito importante
- Moderadamente importante
- Levemente importante
- Nada importante

Pense sobre as razões pelas quais você consome feijão.
O quanto essa afirmação é importante para você "...porque é rápido de preparar"

- Extremamente importante
- Muito importante
- Moderadamente importante
- Levemente importante
- Nada importante

Pense sobre as razões pelas quais você consome feijão.

O quanto essa afirmação é importante para você "...porque é saboroso"

- Extremamente importante
- Muito importante
- Moderadamente importante
- Levemente importante
- Nada importante

Pense sobre as razões pelas quais você consome feijão.
O quanto essa afirmação é importante para você "...porque eu me dou ao luxo"

- Extremamente importante
- Muito importante
- Moderadamente importante
- Levemente importante
- Nada importante

Pense sobre as razões pelas quais você consome feijão.
O quanto essa afirmação é importante para você "...porque é natural (exemplo, não geneticamente modificado)"

- Extremamente importante
- Muito importante
- Moderadamente importante
- Levemente importante
- Nada importante

Pense sobre as razões pelas quais você consome feijão.
O quanto essa afirmação é importante para você "...porque eu não quero gastar muito"

- Extremamente importante

- Muito importante
- Moderadamente importante
- Levemente importante
- Nada importante

Pense sobre as razões pelas quais você consome feijão.
O quanto essa afirmação é importante para você "...porque tem baixas calorias"

- Extremamente importante
- Muito importante
- Moderadamente importante
- Levemente importante
- Nada importante

Pense sobre as razões pelas quais você consome feijão.
O quanto essa afirmação é importante para você "...porque estou frustrado"

- Extremamente importante
- Muito importante
- Moderadamente importante
- Levemente importante
- Nada importante

Pense sobre as razões pelas quais você consome feijão.
O quanto essa afirmação é importante para você "...porque me faz parecer melhor na frente dos outros "

- Extremamente importante
- Muito importante
- Moderadamente importante
- Levemente importante
- Nada importante

Pense sobre as razões pelas quais você consome feijão.
O quanto essa afirmação é importante para você "...porque seria falta de educação não comer "

- Extremamente importante
- Muito importante
- Moderadamente importante
- Levemente importante
- Nada importante

Pense sobre as razões pelas quais você consome feijão.
O quanto essa afirmação é importante para você "...porque a apresentação é atraente (por exemplo a embalagem)"

- Extremamente importante
- Muito importante
- Moderadamente importante
- Levemente importante
- Nada importante

Pense sobre as razões pelas quais você consome feijão.
O quanto essa afirmação é importante para você "...porque eu posso desfrutar o momento com outras pessoas"

- Extremamente importante
- Muito importante
- Moderadamente importante
- Levemente importante
- Nada importante

Pense sobre as razões pelas quais você consome feijão.
O quanto essa afirmação é importante para você "...porque é orgânico"

- Extremamente importante
- Muito importante
- Moderadamente importante
- Levemente importante
- Nada importante

Pense sobre as razões pelas quais você consome feijão.
O quanto essa afirmação é importante para você "...porque eu cresci comendo isso"

- Extremamente importante
- Muito importante
- Moderadamente importante
- Levemente importante
- Nada importante

Pense sobre as razões pelas quais você consome feijão.
O quanto essa afirmação é importante para você "...porque eu curto"

- Extremamente importante
- Muito importante
- Moderadamente importante
- Levemente importante
- Nada importante

Pense sobre as razões pelas quais você consome feijão.
O quanto essa afirmação é importante para você "...porque eu estou faminto"

- Extremamente importante

- Muito importante
- Moderadamente importante
- Levemente importante
- Nada importante

Pense sobre as razões pelas quais você consome feijão.
O quanto essa afirmação é importante para você "...porque eu tenho o hábito de comer"

- Extremamente importante
- Muito importante
- Moderadamente importante
- Levemente importante
- Nada importante

Pense sobre as razões pelas quais você consome feijão.
O quanto essa afirmação é importante para você "...porque eu tenho vontade de comer isso"

- Extremamente importante
- Muito importante
- Moderadamente importante
- Levemente importante
- Nada importante

Pense sobre as razões pelas quais você consome feijão.
O quanto essa afirmação é importante para você "...porque é fácil preparar"

- Extremamente importante
- Muito importante
- Moderadamente importante
- Levemente importante

- Nada importante

Pense sobre as razões pelas quais você consome feijão.
O quanto essa afirmação é importante para você "...porque eu estou triste"

- Extremamente importante
- Muito importante
- Moderadamente importante
- Levemente importante
- Nada importante

Pense sobre as razões pelas quais você consome feijão.
O quanto essa afirmação é importante para você "...porque tem pouca gordura"

- Extremamente importante
- Muito importante
- Moderadamente importante
- Levemente importante
- Nada importante

Pense sobre as razões pelas quais você consome feijão.
O quanto essa afirmação é importante para você "...porque eu vi propaganda em algum lugar ou vi na TV"

- Extremamente importante
- Muito importante
- Moderadamente importante
- Levemente importante
- Nada importante

Pense sobre as razões pelas quais você consome feijão.

O quanto essa afirmação é importante para você "...porque deixa as reuniões sociais mais agradáveis"

- Extremamente importante
- Muito importante
- Moderadamente importante
- Levemente importante
- Nada importante

Pense sobre as razões pelas quais você consome feijão.
O quanto essa afirmação é importante para você "...porque eu devo comer"

- Extremamente importante
- Muito importante
- Moderadamente importante
- Levemente importante
- Nada importante

Pense sobre as razões pelas quais você consome feijão.
O quanto essa afirmação é importante para você "...porque os outros gostam"

- Extremamente importante
- Muito importante
- Moderadamente importante
- Levemente importante
- Nada importante

Pense sobre as razões pelas quais você consome feijão.
O quanto essa afirmação é importante para você "...porque eu me sinto sozinho"

- Extremamente importante
- Muito importante

- Moderadamente importante
- Levemente importante
- Nada importante

Pense sobre as razões pelas quais você consome feijão.
O quanto essa afirmação é importante para você "...porque eu controlo meu peso"

- Extremamente importante
- Muito importante
- Moderadamente importante
- Levemente importante
- Nada importante

Pense sobre as razões pelas quais você consome feijão.
O quanto essa afirmação é importante para você "...porque não contém substâncias perigosas (por exemplo, agrotóxicos, poluentes, antibióticos)"

- Extremamente importante
- Muito importante
- Moderadamente importante
- Levemente importante
- Nada importante

Pense sobre as razões pelas quais você consome feijão.
O quanto essa afirmação é importante para você "...porque é mais conveniente"

- Extremamente importante
- Muito importante
- Moderadamente importante
- Levemente importante
- Nada importante

Pense sobre as razões pelas quais você consome feijão.
O quanto essa afirmação é importante para você "...porque eu preciso de energia"

- Extremamente importante
- Muito importante
- Moderadamente importante
- Levemente importante
- Nada importante

Pense sobre as razões pelas quais você consome feijão.
O quanto essa afirmação é importante para você "...porque me é familiar"

- Extremamente importante
- Muito importante
- Moderadamente importante
- Levemente importante
- Nada importante

Pense sobre as razões pelas quais você consome feijão.
O quanto essa afirmação é importante para você "...porque eu gosto"

- Extremamente importante
- Muito importante
- Moderadamente importante
- Levemente importante
- Nada importante

Pense sobre as razões pelas quais você consome feijão.
O quanto essa afirmação é importante para você "...porque me sacia de forma agradável"

- Extremamente importante

- Muito importante
- Moderadamente importante
- Levemente importante
- Nada importante

Pense sobre as razões pelas quais você consome feijão.
O quanto essa afirmação é importante para você "...para compensar"

- Extremamente importante
- Muito importante
- Moderadamente importante
- Levemente importante
- Nada importante

Pense sobre as razões pelas quais você consome feijão.
O quanto essa afirmação é importante para você "...porque não faz parte das tradições como Natal, Ano Novo e ocasiões especiais"

- Extremamente importante
- Muito importante
- Moderadamente importante
- Levemente importante
- Nada importante

Pense sobre as razões pelas quais você consome feijão.
O quanto essa afirmação é importante para você "...porque faz parte de certas situações"

- Extremamente importante
- Muito importante
- Moderadamente importante
- Levemente importante

- Nada importante

Pense sobre as razões pelas quais você consome feijão.
O quanto essa afirmação é importante para você "...para manter uma dieta balanceada"

- Extremamente importante
- Muito importante
- Moderadamente importante
- Levemente importante
- Nada importante

Pense sobre as razões pelas quais você consome feijão.
O quanto essa afirmação é importante para você "...porque é sociável"

- Extremamente importante
- Muito importante
- Moderadamente importante
- Levemente importante
- Nada importante

Pense sobre as razões pelas quais você consome feijão.
O quanto essa afirmação é importante para você "...porque não é caro"

- Extremamente importante
- Muito importante
- Moderadamente importante
- Levemente importante
- Nada importante

Pense sobre as razões pelas quais você consome feijão.

O quanto essa afirmação é importante para você "...porque me atraiu aparentemente (por exemplo, cores atraentes)"

- Extremamente importante
- Muito importante
- Moderadamente importante
- Levemente importante
- Nada importante

Pense sobre as razões pelas quais você consome feijão.
O quanto essa afirmação é importante para você "...para não desapontar alguém que está tentando me agradar"

- Extremamente importante
- Muito importante
- Moderadamente importante
- Levemente importante
- Nada importante

Pense sobre as razões pelas quais você consome feijão.
O quanto essa afirmação é importante para você "...porque é moda"

- Extremamente importante
- Muito importante
- Moderadamente importante
- Levemente importante
- Nada importante

Pense sobre as razões pelas quais você consome feijão.
O quanto essa afirmação é importante para você "...porque está em promoção"

- Extremamente importante

- Muito importante
- Moderadamente importante
- Levemente importante
- Nada importante

Pense sobre as razões pelas quais você consome feijão.
O quanto essa afirmação é importante para você "...porque me mantém em forma (por exemplo, vigoroso, dinâmico)"

- Extremamente importante
- Muito importante
- Moderadamente importante
- Levemente importante
- Nada importante

Pense sobre as razões pelas quais você consome feijão.
O quanto essa afirmação é importante para você "...porque é a única escolha"

- Extremamente importante
- Muito importante
- Moderadamente importante
- Levemente importante
- Nada importante

Pense sobre as razões pelas quais você consome feijão.
O quanto essa afirmação é importante para você "...porque eu quero comer todos os dias"

- Extremamente importante
- Muito importante
- Moderadamente importante
- Levemente importante

- Nada importante

Pense sobre as razões pelas quais você não consome feijão.
O quanto essa afirmação é importante para você "...eu não acho que seja um lanche"

- Extremamente importante
- Muito importante
- Moderadamente importante
- Levemente importante
- Nada importante

Pense sobre as razões pelas quais você não consome feijão.
O quanto essa afirmação é importante para você "...eu não gosto disso"

- Extremamente importante
- Muito importante
- Moderadamente importante
- Levemente importante
- Nada importante

Pense sobre as razões pelas quais você não consome feijão.
O quanto essa afirmação é importante para você "...normalmente eu não como isso"

- Extremamente importante
- Muito importante
- Moderadamente importante
- Levemente importante
- Nada importante

Pense sobre as razões pelas quais você não consome feijão.
O quanto essa afirmação é importante para você "...o tamanho da porção não é adequado"

- Extremamente importante
- Muito importante
- Moderadamente importante
- Levemente importante
- Nada importante

Pense sobre as razões pelas quais você não consome feijão.
O quanto essa afirmação é importante para você "...não sustenta o suficiente"

- Extremamente importante
- Muito importante
- Moderadamente importante
- Levemente importante
- Nada importante

Pense sobre as razões pelas quais você não consome feijão.
O quanto essa afirmação é importante para você "...não é saudável"

- Extremamente importante
- Muito importante
- Moderadamente importante
- Levemente importante
- Nada importante

Pense sobre as razões pelas quais você não consome feijão.
O quanto essa afirmação é importante para você "...não é conveniente"

- Extremamente importante

- Muito importante
- Moderadamente importante
- Levemente importante
- Nada importante

Pense sobre as razões pelas quais você não consome feijão.
O quanto essa afirmação é importante para você "...eu não quero me dar ao luxo"

- Extremamente importante
- Muito importante
- Moderadamente importante
- Levemente importante
- Nada importante

Pense sobre as razões pelas quais você não consome feijão.
O quanto essa afirmação é importante para você "...não é apropriado para a situação"

- Extremamente importante
- Muito importante
- Moderadamente importante
- Levemente importante
- Nada importante

Pense sobre as razões pelas quais você não consome feijão.
O quanto essa afirmação é importante para você "...não é um alimento que eu como perto de outras pessoas"

- Extremamente importante
- Muito importante
- Moderadamente importante
- Levemente importante

- Nada importante

Pense sobre as razões pelas quais você não consome feijão.
O quanto essa afirmação é importante para você "...o preço está muito alto"

- Extremamente importante
- Muito importante
- Moderadamente importante
- Levemente importante
- Nada importante

Pense sobre as razões pelas quais você não consome feijão.
O quanto essa afirmação é importante para você "...eu não gosto do jeito que parece"

- Extremamente importante
- Muito importante
- Moderadamente importante
- Levemente importante
- Nada importante

Pense sobre as razões pelas quais você não consome feijão.
O quanto essa afirmação é importante para você "...muito rico em calorias"

- Extremamente importante
- Muito importante
- Moderadamente importante
- Levemente importante
- Nada importante

Pense sobre as razões pelas quais você não consome feijão.

O quanto essa afirmação é importante para você "...eu não devo comer isso"

- Extremamente importante
- Muito importante
- Moderadamente importante
- Levemente importante
- Nada importante

Pense sobre as razões pelas quais você não consome feijão.
O quanto essa afirmação é importante para você "...comer isso me faz parecer antiquado"

- Extremamente importante
- Muito importante
- Moderadamente importante
- Levemente importante
- Nada importante

Pense sobre as razões pelas quais você não consome feijão.
O quanto essa afirmação é importante para você "...não é nada que eu queira comer agora"

- Extremamente importante
- Muito importante
- Moderadamente importante
- Levemente importante
- Nada importante

Pense sobre as razões pelas quais você não consome feijão.
O quanto essa afirmação é importante para você "...eu comi recentemente e não quero comer a mesma comida com tanta frequência"

- Extremamente importante
- Muito importante
- Moderadamente importante
- Levemente importante
- Nada importante

Pense sobre as razões pelas quais você não consome feijão.
O quanto essa afirmação é importante para você "...eu nunca escolheria isso porque eu gosto de comer a mesma comida todos os dias"

- Extremamente importante
- Muito importante
- Moderadamente importante
- Levemente importante
- Nada importante

Pense sobre as razões pelas quais você não consome feijão.
O quanto essa afirmação é importante para você "...essa comida me faz sentir triste, solitário e frustrado"

- Extremamente importante
- Muito importante
- Moderadamente importante
- Levemente importante
- Nada importante

Pense sobre as razões pelas quais você não consome feijão.
O quanto essa afirmação é importante para você "...não é orgânico"

- Extremamente importante
- Muito importante
- Moderadamente importante

- Levemente importante
- Nada importante

Reasons for Dairy Food Choices(Full RATA)

Você consome leite?

- Sim
- Não

Pense sobre as razões pelas quais você consome leite.
O quanto essa afirmação é importante para você "...porque estou a acostumado a comer isso"

- Extremamente importante
- Muito importante
- Moderadamente importante
- Levemente importante
- Nada importante

Pense sobre as razões pelas quais você consome leite.
O quanto essa afirmação é importante para você "...porque é saudável"

- Extremamente importante
- Muito importante
- Moderadamente importante
- Levemente importante
- Nada importante

Pense sobre as razões pelas quais você consome leite.

O quanto essa afirmação é importante para você "...porque é rápido de preparar"

- Extremamente importante
- Muito importante
- Moderadamente importante
- Levemente importante
- Nada importante

Pense sobre as razões pelas quais você consome leite.
O quanto essa afirmação é importante para você "...porque é saboroso"

- Extremamente importante
- Muito importante
- Moderadamente importante
- Levemente importante
- Nada importante

Pense sobre as razões pelas quais você consome leite.
O quanto essa afirmação é importante para você "...porque eu me dou ao luxo"

- Extremamente importante
- Muito importante
- Moderadamente importante
- Levemente importante
- Nada importante

Pense sobre as razões pelas quais você consome leite.
O quanto essa afirmação é importante para você "...porque é natural (exemplo, não geneticamente modificado)"

- Extremamente importante
- Muito importante

- Moderadamente importante
- Levemente importante
- Nada importante

Pense sobre as razões pelas quais você consome leite.
O quanto essa afirmação é importante para você "...porque eu não quero gastar muito"

- Extremamente importante
- Muito importante
- Moderadamente importante
- Levemente importante
- Nada importante

Pense sobre as razões pelas quais você consome leite.
O quanto essa afirmação é importante para você "...porque tem baixas calorias"

- Extremamente importante
- Muito importante
- Moderadamente importante
- Levemente importante
- Nada importante

Pense sobre as razões pelas quais você consome leite.
O quanto essa afirmação é importante para você "...porque estou frustrado"

- Extremamente importante
- Muito importante
- Moderadamente importante
- Levemente importante
- Nada importante

Pense sobre as razões pelas quais você consome leite.
O quanto essa afirmação é importante para você "...porque me faz parecer melhor na frente dos outros "

- Extremamente importante
- Muito importante
- Moderadamente importante
- Levemente importante
- Nada importante

Pense sobre as razões pelas quais você consome leite.
O quanto essa afirmação é importante para você "...porque seria falta de educação não comer "

- Extremamente importante
- Muito importante
- Moderadamente importante
- Levemente importante
- Nada importante

Pense sobre as razões pelas quais você consome leite.
O quanto essa afirmação é importante para você "...porque a apresentação é atraente (por exemplo a embalagem)"

- Extremamente importante
- Muito importante
- Moderadamente importante
- Levemente importante
- Nada importante

Pense sobre as razões pelas quais você consome leite.

O quanto essa afirmação é importante para você "...porque eu posso desfrutar o momento com outras pessoas"

- Extremamente importante
- Muito importante
- Moderadamente importante
- Levemente importante
- Nada importante

Pense sobre as razões pelas quais você consome leite.
O quanto essa afirmação é importante para você "...porque é orgânico"

- Extremamente importante
- Muito importante
- Moderadamente importante
- Levemente importante
- Nada importante

Pense sobre as razões pelas quais você consome leite.
O quanto essa afirmação é importante para você "...porque eu cresci comendo isso"

- Extremamente importante
- Muito importante
- Moderadamente importante
- Levemente importante
- Nada importante

Pense sobre as razões pelas quais você consome leite.
O quanto essa afirmação é importante para você "...porque eu curto"

- Extremamente importante

- Muito importante
- Moderadamente importante
- Levemente importante
- Nada importante

Pense sobre as razões pelas quais você consome leite.
O quanto essa afirmação é importante para você "...porque eu estou faminto"

- Extremamente importante
- Muito importante
- Moderadamente importante
- Levemente importante
- Nada importante

Pense sobre as razões pelas quais você consome leite.
O quanto essa afirmação é importante para você "...porque eu tenho o hábito de comer"

- Extremamente importante
- Muito importante
- Moderadamente importante
- Levemente importante
- Nada importante

Pense sobre as razões pelas quais você consome leite.
O quanto essa afirmação é importante para você "...porque eu tenho vontade de comer isso"

- Extremamente importante
- Muito importante
- Moderadamente importante
- Levemente importante

- Nada importante

Pense sobre as razões pelas quais você consome leite.
O quanto essa afirmação é importante para você "...porque é fácil preparar"

- Extremamente importante
- Muito importante
- Moderadamente importante
- Levemente importante
- Nada importante

Pense sobre as razões pelas quais você consome leite.
O quanto essa afirmação é importante para você "...porque eu estou triste"

- Extremamente importante
- Muito importante
- Moderadamente importante
- Levemente importante
- Nada importante

Pense sobre as razões pelas quais você consome leite.
O quanto essa afirmação é importante para você "...porque tem pouca gordura"

- Extremamente importante
- Muito importante
- Moderadamente importante
- Levemente importante
- Nada importante

Pense sobre as razões pelas quais você consome leite.

O quanto essa afirmação é importante para você "...porque eu vi propaganda em algum lugar ou vi na TV"

- Extremamente importante
- Muito importante
- Moderadamente importante
- Levemente importante
- Nada importante

Pense sobre as razões pelas quais você consome leite.
O quanto essa afirmação é importante para você "...porque deixa as reuniões sociais mais agradáveis"

- Extremamente importante
- Muito importante
- Moderadamente importante
- Levemente importante
- Nada importante

Pense sobre as razões pelas quais você consome leite.
O quanto essa afirmação é importante para você "...porque eu devo comer"

- Extremamente importante
- Muito importante
- Moderadamente importante
- Levemente importante
- Nada importante

Pense sobre as razões pelas quais você consome leite.
O quanto essa afirmação é importante para você "...porque os outros gostam"

- Extremamente importante

- Muito importante
- Moderadamente importante
- Levemente importante
- Nada importante

Pense sobre as razões pelas quais você consome leite.
O quanto essa afirmação é importante para você "...porque eu me sinto sozinho"

- Extremamente importante
- Muito importante
- Moderadamente importante
- Levemente importante
- Nada importante

Pense sobre as razões pelas quais você consome leite.
O quanto essa afirmação é importante para você "...porque eu controlo meu peso"

- Extremamente importante
- Muito importante
- Moderadamente importante
- Levemente importante
- Nada importante

Pense sobre as razões pelas quais você consome leite.
O quanto essa afirmação é importante para você "...porque não contém substâncias perigosas (por exemplo, agrotóxicos, poluentes, antibióticos)"

- Extremamente importante
- Muito importante
- Moderadamente importante
- Levemente importante
- Nada importante

Pense sobre as razões pelas quais você consome leite.
O quanto essa afirmação é importante para você "...porque é mais conveniente"

- Extremamente importante
- Muito importante
- Moderadamente importante
- Levemente importante
- Nada importante

Pense sobre as razões pelas quais você consome leite.
O quanto essa afirmação é importante para você "...porque eu preciso de energia"

- Extremamente importante
- Muito importante
- Moderadamente importante
- Levemente importante
- Nada importante

Pense sobre as razões pelas quais você consome leite.
O quanto essa afirmação é importante para você "...porque me é familiar"

- Extremamente importante
- Muito importante
- Moderadamente importante
- Levemente importante
- Nada importante

Pense sobre as razões pelas quais você consome leite.
O quanto essa afirmação é importante para você "...porque eu gosto"

- Extremamente importante
- Muito importante
- Moderadamente importante
- Levemente importante
- Nada importante

Pense sobre as razões pelas quais você consome leite.
O quanto essa afirmação é importante para você "...porque me sacia de forma agradável"

- Extremamente importante
- Muito importante
- Moderadamente importante
- Levemente importante
- Nada importante

Pense sobre as razões pelas quais você consome leite.
O quanto essa afirmação é importante para você "...para compensar"

- Extremamente importante
- Muito importante
- Moderadamente importante
- Levemente importante
- Nada importante

Pense sobre as razões pelas quais você consome leite.
O quanto essa afirmação é importante para você "...porque não faz parte das tradições como Natal, Ano Novo e ocasiões especiais"

- Extremamente importante
- Muito importante
- Moderadamente importante

- Levemente importante
- Nada importante

Pense sobre as razões pelas quais você consome leite.
O quanto essa afirmação é importante para você "...porque faz parte de certas situações"

- Extremamente importante
- Muito importante
- Moderadamente importante
- Levemente importante
- Nada importante

Pense sobre as razões pelas quais você consome leite.
O quanto essa afirmação é importante para você "...para manter uma dieta balanceada"

- Extremamente importante
- Muito importante
- Moderadamente importante
- Levemente importante
- Nada importante

Pense sobre as razões pelas quais você consome leite.
O quanto essa afirmação é importante para você "...porque é sociável"

- Extremamente importante
- Muito importante
- Moderadamente importante
- Levemente importante
- Nada importante

Pense sobre as razões pelas quais você consome leite.
O quanto essa afirmação é importante para você "...porque não é caro"

- Extremamente importante
- Muito importante
- Moderadamente importante
- Levemente importante
- Nada importante

Pense sobre as razões pelas quais você consome leite.
O quanto essa afirmação é importante para você "...porque me atraiu aparentemente (por exemplo, cores atraentes)"

- Extremamente importante
- Muito importante
- Moderadamente importante
- Levemente importante
- Nada importante

Pense sobre as razões pelas quais você consome leite.
O quanto essa afirmação é importante para você "...para não desapontar alguém que está tentando me agradar"

- Extremamente importante
- Muito importante
- Moderadamente importante
- Levemente importante
- Nada importante

Pense sobre as razões pelas quais você consome leite.
O quanto essa afirmação é importante para você "...porque é moda"

- Extremamente importante
- Muito importante
- Moderadamente importante
- Levemente importante
- Nada importante

Pense sobre as razões pelas quais você consome leite.
O quanto essa afirmação é importante para você "...porque está em promoção"

- Extremamente importante
- Muito importante
- Moderadamente importante
- Levemente importante
- Nada importante

Pense sobre as razões pelas quais você consome leite.
O quanto essa afirmação é importante para você "...porque me mantém em forma (por exemplo, vigoroso, dinâmico)"

- Extremamente importante
- Muito importante
- Moderadamente importante
- Levemente importante
- Nada importante

Pense sobre as razões pelas quais você consome leite.
O quanto essa afirmação é importante para você "...porque é a única escolha"

- Extremamente importante
- Muito importante
- Moderadamente importante
- Levemente importante

- Nada importante

Pense sobre as razões pelas quais você consome leite.
O quanto essa afirmação é importante para você "...porque eu quero comer todos os dias"

- Extremamente importante
- Muito importante
- Moderadamente importante
- Levemente importante
- Nada importante

Pense sobre as razões pelas quais você não consome leite.
O quanto essa afirmação é importante para você "...eu não acho que seja um lanche"

- Extremamente importante
- Muito importante
- Moderadamente importante
- Levemente importante
- Nada importante

Pense sobre as razões pelas quais você não consome leite.
O quanto essa afirmação é importante para você "...eu não gosto disso"

- Extremamente importante
- Muito importante
- Moderadamente importante
- Levemente importante
- Nada importante

Pense sobre as razões pelas quais você não consome leite.
O quanto essa afirmação é importante para você "...normalmente eu não como isso"

- Extremamente importante
- Muito importante
- Moderadamente importante
- Levemente importante
- Nada importante

Pense sobre as razões pelas quais você não consome leite.
O quanto essa afirmação é importante para você "...o tamanho da porção não é adequado"

- Extremamente importante
- Muito importante
- Moderadamente importante
- Levemente importante
- Nada importante

Pense sobre as razões pelas quais você não consome leite.
O quanto essa afirmação é importante para você "...não sustenta o suficiente"

- Extremamente importante
- Muito importante
- Moderadamente importante
- Levemente importante
- Nada importante

Pense sobre as razões pelas quais você não consome leite.
O quanto essa afirmação é importante para você "...não é saudável"

- Extremamente importante
- Muito importante
- Moderadamente importante
- Levemente importante
- Nada importante

Pense sobre as razões pelas quais você não consome leite.
O quanto essa afirmação é importante para você "...não é conveniente"

- Extremamente importante
- Muito importante
- Moderadamente importante
- Levemente importante
- Nada importante

Pense sobre as razões pelas quais você não consome leite.
O quanto essa afirmação é importante para você "...eu não quero me dar ao luxo"

- Extremamente importante
- Muito importante
- Moderadamente importante
- Levemente importante
- Nada importante

Pense sobre as razões pelas quais você não consome leite.
O quanto essa afirmação é importante para você "...não é apropriado para a situação"

- Extremamente importante
- Muito importante
- Moderadamente importante
- Levemente importante

- Nada importante

Pense sobre as razões pelas quais você não consome leite.
O quanto essa afirmação é importante para você "...não é um alimento que eu como perto de outras pessoas"

- Extremamente importante
- Muito importante
- Moderadamente importante
- Levemente importante
- Nada importante

Pense sobre as razões pelas quais você não consome leite.
O quanto essa afirmação é importante para você "...o preço está muito alto"

- Extremamente importante
- Muito importante
- Moderadamente importante
- Levemente importante
- Nada importante

Pense sobre as razões pelas quais você não consome leite.
O quanto essa afirmação é importante para você "...eu não gosto do jeito que parece"

- Extremamente importante
- Muito importante
- Moderadamente importante
- Levemente importante
- Nada importante

Pense sobre as razões pelas quais você não consome leite.
O quanto essa afirmação é importante para você "...muito rico em calorias"

- Extremamente importante
- Muito importante
- Moderadamente importante
- Levemente importante
- Nada importante

Pense sobre as razões pelas quais você não consome leite.
O quanto essa afirmação é importante para você "...eu não devo comer isso"

- Extremamente importante
- Muito importante
- Moderadamente importante
- Levemente importante
- Nada importante

Pense sobre as razões pelas quais você não consome leite.
O quanto essa afirmação é importante para você "...comer isso me faz parecer antiquado"

- Extremamente importante
- Muito importante
- Moderadamente importante
- Levemente importante
- Nada importante

Pense sobre as razões pelas quais você não consome leite.
O quanto essa afirmação é importante para você "...não é nada que eu queira comer agora"

- Extremamente importante
- Muito importante
- Moderadamente importante
- Levemente importante
- Nada importante

Pense sobre as razões pelas quais você não consome leite.
O quanto essa afirmação é importante para você "...eu comi recentemente e não quero comer a mesma comida com tanta frequência"

- Extremamente importante
- Muito importante
- Moderadamente importante
- Levemente importante
- Nada importante

Pense sobre as razões pelas quais você não consome leite.
O quanto essa afirmação é importante para você "...eu nunca escolheria isso porque eu gosto de comer a mesma comida todos os dias"

- Extremamente importante
- Muito importante
- Moderadamente importante
- Levemente importante
- Nada importante

Pense sobre as razões pelas quais você não consome leite.
O quanto essa afirmação é importante para você "...essa comida me faz sentir triste, solitário e frustrado"

- Extremamente importante
- Muito importante

- Moderadamente importante
- Levemente importante
- Nada importante

Pense sobre as razões pelas quais você não consome leite.
O quanto essa afirmação é importante para você "...não é orgânico"

- Extremamente importante
- Muito importante
- Moderadamente importante
- Levemente importante
- Nada importante

Reasons for Fruit Choices(Full RATA)

Você consome banana?

- Sim
- Não

Pense sobre as razões pelas quais você consome banana.
O quanto essa afirmação é importante para você "...porque estou a acostumado a comer isso"

- Extremamente importante
- Muito importante
- Moderadamente importante
- Levemente importante
- Nada importante

Pense sobre as razões pelas quais você consome banana.
O quanto essa afirmação é importante para você "...porque é saudável"

- Extremamente importante
- Muito importante
- Moderadamente importante
- Levemente importante
- Nada importante

Pense sobre as razões pelas quais você consome banana.
O quanto essa afirmação é importante para você "...porque é rápido de preparar"

- Extremamente importante
- Muito importante
- Moderadamente importante
- Levemente importante
- Nada importante

Pense sobre as razões pelas quais você consome banana.
O quanto essa afirmação é importante para você "...porque é saboroso"

- Extremamente importante
- Muito importante
- Moderadamente importante
- Levemente importante
- Nada importante

Pense sobre as razões pelas quais você consome banana.
O quanto essa afirmação é importante para você "...porque eu me dou ao luxo"

- Extremamente importante
- Muito importante

- Moderadamente importante
- Levemente importante
- Nada importante

Pense sobre as razões pelas quais você consome banana.
O quanto essa afirmação é importante para você "...porque é natural (exemplo, não geneticamente modificado)"

- Extremamente importante
- Muito importante
- Moderadamente importante
- Levemente importante
- Nada importante

Pense sobre as razões pelas quais você consome banana.
O quanto essa afirmação é importante para você "...porque eu não quero gastar muito"

- Extremamente importante
- Muito importante
- Moderadamente importante
- Levemente importante
- Nada importante

Pense sobre as razões pelas quais você consome banana.
O quanto essa afirmação é importante para você "...porque tem baixas calorias"

- Extremamente importante
- Muito importante
- Moderadamente importante
- Levemente importante
- Nada importante

Pense sobre as razões pelas quais você consome banana.
O quanto essa afirmação é importante para você "...porque estou frustrado"

- Extremamente importante
- Muito importante
- Moderadamente importante
- Levemente importante
- Nada importante

Pense sobre as razões pelas quais você consome banana.
O quanto essa afirmação é importante para você "...porque me faz parecer melhor na frente dos outros "

- Extremamente importante
- Muito importante
- Moderadamente importante
- Levemente importante
- Nada importante

Pense sobre as razões pelas quais você consome banana.
O quanto essa afirmação é importante para você "...porque seria falta de educação não comer "

- Extremamente importante
- Muito importante
- Moderadamente importante
- Levemente importante
- Nada importante

Pense sobre as razões pelas quais você consome banana.

O quanto essa afirmação é importante para você "...porque a apresentação é atraente (por exemplo a embalagem)"

- Extremamente importante
- Muito importante
- Moderadamente importante
- Levemente importante
- Nada importante

Pense sobre as razões pelas quais você consome banana.
O quanto essa afirmação é importante para você "...porque eu posso desfrutar o momento com outras pessoas"

- Extremamente importante
- Muito importante
- Moderadamente importante
- Levemente importante
- Nada importante

Pense sobre as razões pelas quais você consome banana.
O quanto essa afirmação é importante para você "...porque é orgânico"

- Extremamente importante
- Muito importante
- Moderadamente importante
- Levemente importante
- Nada importante

Pense sobre as razões pelas quais você consome banana.
O quanto essa afirmação é importante para você "...porque eu cresci comendo isso"

- Extremamente importante
- Muito importante
- Moderadamente importante
- Levemente importante
- Nada importante

Pense sobre as razões pelas quais você consome banana.
O quanto essa afirmação é importante para você "...porque eu curto"

- Extremamente importante
- Muito importante
- Moderadamente importante
- Levemente importante
- Nada importante

Pense sobre as razões pelas quais você consome banana.
O quanto essa afirmação é importante para você "...porque eu estou faminto"

- Extremamente importante
- Muito importante
- Moderadamente importante
- Levemente importante
- Nada importante

Pense sobre as razões pelas quais você consome banana.
O quanto essa afirmação é importante para você "...porque eu tenho o hábito de comer"

- Extremamente importante
- Muito importante
- Moderadamente importante
- Levemente importante

- Nada importante

Pense sobre as razões pelas quais você consome banana.
O quanto essa afirmação é importante para você "...porque eu tenho vontade de comer isso"

- Extremamente importante
- Muito importante
- Moderadamente importante
- Levemente importante
- Nada importante

Pense sobre as razões pelas quais você consome banana.
O quanto essa afirmação é importante para você "...porque é fácil preparar"

- Extremamente importante
- Muito importante
- Moderadamente importante
- Levemente importante
- Nada importante

Pense sobre as razões pelas quais você consome banana.
O quanto essa afirmação é importante para você "...porque eu estou triste"

- Extremamente importante
- Muito importante
- Moderadamente importante
- Levemente importante
- Nada importante

Pense sobre as razões pelas quais você consome banana.

O quanto essa afirmação é importante para você "...porque tem pouca gordura"

- Extremamente importante
- Muito importante
- Moderadamente importante
- Levemente importante
- Nada importante

Pense sobre as razões pelas quais você consome banana.
O quanto essa afirmação é importante para você "...porque eu vi propaganda em algum lugar ou vi na TV"

- Extremamente importante
- Muito importante
- Moderadamente importante
- Levemente importante
- Nada importante

Pense sobre as razões pelas quais você consome banana.
O quanto essa afirmação é importante para você "...porque deixa as reuniões sociais mais agradáveis"

- Extremamente importante
- Muito importante
- Moderadamente importante
- Levemente importante
- Nada importante

Pense sobre as razões pelas quais você consome banana.
O quanto essa afirmação é importante para você "...porque eu devo comer"

- Extremamente importante

- Muito importante
- Moderadamente importante
- Levemente importante
- Nada importante

Pense sobre as razões pelas quais você consome banana.
O quanto essa afirmação é importante para você "...porque os outros gostam"

- Extremamente importante
- Muito importante
- Moderadamente importante
- Levemente importante
- Nada importante

Pense sobre as razões pelas quais você consome banana.
O quanto essa afirmação é importante para você "...porque eu me sinto sozinho"

- Extremamente importante
- Muito importante
- Moderadamente importante
- Levemente importante
- Nada importante

Pense sobre as razões pelas quais você consome banana.
O quanto essa afirmação é importante para você "...porque eu controlo meu peso"

- Extremamente importante
- Muito importante
- Moderadamente importante
- Levemente importante
- Nada importante

Pense sobre as razões pelas quais você consome banana.
O quanto essa afirmação é importante para você "...porque não contém substâncias perigosas (por exemplo, agrotóxicos, poluentes, antibióticos)"

- Extremamente importante
- Muito importante
- Moderadamente importante
- Levemente importante
- Nada importante

Pense sobre as razões pelas quais você consome banana.
O quanto essa afirmação é importante para você "...porque é mais conveniente"

- Extremamente importante
- Muito importante
- Moderadamente importante
- Levemente importante
- Nada importante

Pense sobre as razões pelas quais você consome banana.
O quanto essa afirmação é importante para você "...porque eu preciso de energia"

- Extremamente importante
- Muito importante
- Moderadamente importante
- Levemente importante
- Nada importante

Pense sobre as razões pelas quais você consome banana.
O quanto essa afirmação é importante para você "...porque me é familiar"

- Extremamente importante

- Muito importante
- Moderadamente importante
- Levemente importante
- Nada importante

Pense sobre as razões pelas quais você consome banana.
O quanto essa afirmação é importante para você "...porque eu gosto"

- Extremamente importante
- Muito importante
- Moderadamente importante
- Levemente importante
- Nada importante

Pense sobre as razões pelas quais você consome banana.
O quanto essa afirmação é importante para você "...porque me sacia de forma agradável"

- Extremamente importante
- Muito importante
- Moderadamente importante
- Levemente importante
- Nada importante

Pense sobre as razões pelas quais você consome banana.
O quanto essa afirmação é importante para você "...para compensar"

- Extremamente importante
- Muito importante
- Moderadamente importante
- Levemente importante
- Nada importante

Pense sobre as razões pelas quais você consome banana.
O quanto essa afirmação é importante para você "...porque não faz parte das tradições como Natal, Ano Novo e ocasiões especiais"

- Extremamente importante
- Muito importante
- Moderadamente importante
- Levemente importante
- Nada importante

Pense sobre as razões pelas quais você consome banana.
O quanto essa afirmação é importante para você "...porque faz parte de certas situações"

- Extremamente importante
- Muito importante
- Moderadamente importante
- Levemente importante
- Nada importante

Pense sobre as razões pelas quais você consome banana.
O quanto essa afirmação é importante para você "...para manter uma dieta balanceada"

- Extremamente importante
- Muito importante
- Moderadamente importante
- Levemente importante
- Nada importante

Pense sobre as razões pelas quais você consome banana.
O quanto essa afirmação é importante para você "...porque é sociável"

- Extremamente importante
- Muito importante
- Moderadamente importante
- Levemente importante
- Nada importante

Pense sobre as razões pelas quais você consome banana.
O quanto essa afirmação é importante para você "...porque não é caro"

- Extremamente importante
- Muito importante
- Moderadamente importante
- Levemente importante
- Nada importante

Pense sobre as razões pelas quais você consome banana.
O quanto essa afirmação é importante para você "...porque me atraiu aparentemente (por exemplo, cores atraentes)"

- Extremamente importante
- Muito importante
- Moderadamente importante
- Levemente importante
- Nada importante

Pense sobre as razões pelas quais você consome banana.
O quanto essa afirmação é importante para você "...para não desapontar alguém que está tentando me agradar"

- Extremamente importante
- Muito importante
- Moderadamente importante
- Levemente importante
- Nada importante

Pense sobre as razões pelas quais você consome banana.
O quanto essa afirmação é importante para você "...porque é moda"

- Extremamente importante
- Muito importante
- Moderadamente importante
- Levemente importante
- Nada importante

Pense sobre as razões pelas quais você consome banana.
O quanto essa afirmação é importante para você "...porque está em promoção"

- Extremamente importante
- Muito importante
- Moderadamente importante
- Levemente importante
- Nada importante

Pense sobre as razões pelas quais você consome banana.
O quanto essa afirmação é importante para você "...porque me mantém em forma (por exemplo, vigoroso, dinâmico)"

- Extremamente importante
- Muito importante
- Moderadamente importante
- Levemente importante

- Nada importante

Pense sobre as razões pelas quais você consome banana.
O quanto essa afirmação é importante para você "...porque é a única escolha"

- Extremamente importante
- Muito importante
- Moderadamente importante
- Levemente importante
- Nada importante

Pense sobre as razões pelas quais você consome banana.
O quanto essa afirmação é importante para você "...porque eu quero comer todos os dias"

- Extremamente importante
- Muito importante
- Moderadamente importante
- Levemente importante
- Nada importante

Pense sobre as razões pelas quais você não consome banana.
O quanto essa afirmação é importante para você "...eu não acho que seja um lanche"

- Extremamente importante
- Muito importante
- Moderadamente importante
- Levemente importante
- Nada importante

Pense sobre as razões pelas quais você não consome banana.
O quanto essa afirmação é importante para você "...eu não gosto disso"

- Extremamente importante
- Muito importante
- Moderadamente importante
- Levemente importante
- Nada importante

Pense sobre as razões pelas quais você não consome banana.
O quanto essa afirmação é importante para você "...normalmente eu não como isso"

- Extremamente importante
- Muito importante
- Moderadamente importante
- Levemente importante
- Nada importante

Pense sobre as razões pelas quais você não consome banana.
O quanto essa afirmação é importante para você "...o tamanho da porção não é adequado"

- Extremamente importante
- Muito importante
- Moderadamente importante
- Levemente importante
- Nada importante

Pense sobre as razões pelas quais você não consome banana.
O quanto essa afirmação é importante para você "...não sustenta o suficiente"

- Extremamente importante
- Muito importante
- Moderadamente importante
- Levemente importante
- Nada importante

Pense sobre as razões pelas quais você não consome banana.
O quanto essa afirmação é importante para você "...não é saudável"

- Extremamente importante
- Muito importante
- Moderadamente importante
- Levemente importante
- Nada importante

Pense sobre as razões pelas quais você não consome banana.
O quanto essa afirmação é importante para você "...não é conveniente"

- Extremamente importante
- Muito importante
- Moderadamente importante
- Levemente importante
- Nada importante

Pense sobre as razões pelas quais você não consome banana.
O quanto essa afirmação é importante para você "...eu não quero me dar ao luxo"

- Extremamente importante
- Muito importante
- Moderadamente importante
- Levemente importante
- Nada importante

Pense sobre as razões pelas quais você não consome banana.
O quanto essa afirmação é importante para você "...não é apropriado para a situação"

- Extremamente importante
- Muito importante
- Moderadamente importante
- Levemente importante
- Nada importante

Pense sobre as razões pelas quais você não consome banana.
O quanto essa afirmação é importante para você "...não é um alimento que eu como perto de outras pessoas"

- Extremamente importante
- Muito importante
- Moderadamente importante
- Levemente importante
- Nada importante

Pense sobre as razões pelas quais você não consome banana.
O quanto essa afirmação é importante para você "...o preço está muito alto"

- Extremamente importante
- Muito importante
- Moderadamente importante
- Levemente importante
- Nada importante

Pense sobre as razões pelas quais você não consome banana.

O quanto essa afirmação é importante para você "...eu não gosto do jeito que parece"

- Extremamente importante
- Muito importante
- Moderadamente importante
- Levemente importante
- Nada importante

Pense sobre as razões pelas quais você não consome banana.
O quanto essa afirmação é importante para você "...muito rico em calorias"

- Extremamente importante
- Muito importante
- Moderadamente importante
- Levemente importante
- Nada importante

Pense sobre as razões pelas quais você não consome banana.
O quanto essa afirmação é importante para você "...eu não devo comer isso"

- Extremamente importante
- Muito importante
- Moderadamente importante
- Levemente importante
- Nada importante

Pense sobre as razões pelas quais você não consome banana.
O quanto essa afirmação é importante para você "...comer isso me faz parecer antiquado"

- Extremamente importante

- Muito importante
- Moderadamente importante
- Levemente importante
- Nada importante

Pense sobre as razões pelas quais você não consome banana.
O quanto essa afirmação é importante para você "...não é nada que eu queira comer agora"

- Extremamente importante
- Muito importante
- Moderadamente importante
- Levemente importante
- Nada importante

Pense sobre as razões pelas quais você não consome banana.
O quanto essa afirmação é importante para você "...eu comi recentemente e não quero comer a mesma comida com tanta frequência"

- Extremamente importante
- Muito importante
- Moderadamente importante
- Levemente importante
- Nada importante

Pense sobre as razões pelas quais você não consome banana.
O quanto essa afirmação é importante para você "...eu nunca escolheria isso porque eu gosto de comer a mesma comida todos os dias"

- Extremamente importante
- Muito importante
- Moderadamente importante

- Levemente importante
- Nada importante

Pense sobre as razões pelas quais você não consome banana.
O quanto essa afirmação é importante para você "...essa comida me faz sentir triste, solitário e frustrado"

- Extremamente importante
- Muito importante
- Moderadamente importante
- Levemente importante
- Nada importante

Pense sobre as razões pelas quais você não consome banana.
O quanto essa afirmação é importante para você "...não é orgânico"

- Extremamente importante
- Muito importante
- Moderadamente importante
- Levemente importante
- Nada importante

Reasons for Sweet food Choices(Full RATA)

Você consome brigadeiro?

- Sim
- Não

Pense sobre as razões pelas quais você consome brigadeiro.

O quanto essa afirmação é importante para você "...porque estou a acostumado a comer isso"

- Extremamente importante
- Muito importante
- Moderadamente importante
- Levemente importante
- Nada importante

Pense sobre as razões pelas quais você consome brigadeiro.
O quanto essa afirmação é importante para você "...porque é saudável"

- Extremamente importante
- Muito importante
- Moderadamente importante
- Levemente importante
- Nada importante

Pense sobre as razões pelas quais você consome brigadeiro.
O quanto essa afirmação é importante para você "...porque é rápido de preparar"

- Extremamente importante
- Muito importante
- Moderadamente importante
- Levemente importante
- Nada importante

Pense sobre as razões pelas quais você consome brigadeiro.
O quanto essa afirmação é importante para você "...porque é saboroso"

- Extremamente importante
- Muito importante

- Moderadamente importante
- Levemente importante
- Nada importante

Pense sobre as razões pelas quais você consome brigadeiro.
O quanto essa afirmação é importante para você "...porque eu me dou ao luxo"

- Extremamente importante
- Muito importante
- Moderadamente importante
- Levemente importante
- Nada importante

Pense sobre as razões pelas quais você consome brigadeiro.
O quanto essa afirmação é importante para você "...porque é natural (exemplo, não geneticamente modificado)"

- Extremamente importante
- Muito importante
- Moderadamente importante
- Levemente importante
- Nada importante

Pense sobre as razões pelas quais você consome brigadeiro.
O quanto essa afirmação é importante para você "...porque eu não quero gastar muito"

- Extremamente importante
- Muito importante
- Moderadamente importante
- Levemente importante
- Nada importante

Pense sobre as razões pelas quais você consome brigadeiro.
O quanto essa afirmação é importante para você "...porque tem baixas calorias"

- Extremamente importante
- Muito importante
- Moderadamente importante
- Levemente importante
- Nada importante

Pense sobre as razões pelas quais você consome brigadeiro.
O quanto essa afirmação é importante para você "...porque estou frustrado"

- Extremamente importante
- Muito importante
- Moderadamente importante
- Levemente importante
- Nada importante

Pense sobre as razões pelas quais você consome brigadeiro.
O quanto essa afirmação é importante para você "...porque me faz parecer melhor na frente dos outros "

- Extremamente importante
- Muito importante
- Moderadamente importante
- Levemente importante
- Nada importante

Pense sobre as razões pelas quais você consome brigadeiro.

O quanto essa afirmação é importante para você "...porque seria falta de educação não comer "

- Extremamente importante
- Muito importante
- Moderadamente importante
- Levemente importante
- Nada importante

Pense sobre as razões pelas quais você consome brigadeiro.
O quanto essa afirmação é importante para você "...porque a apresentação é atraente (por exemplo a embalagem)"

- Extremamente importante
- Muito importante
- Moderadamente importante
- Levemente importante
- Nada importante

Pense sobre as razões pelas quais você consome brigadeiro.
O quanto essa afirmação é importante para você "...porque eu posso desfrutar o momento com outras pessoas"

- Extremamente importante
- Muito importante
- Moderadamente importante
- Levemente importante
- Nada importante

Pense sobre as razões pelas quais você consome brigadeiro.
O quanto essa afirmação é importante para você "...porque é orgânico"

- Extremamente importante
- Muito importante
- Moderadamente importante
- Levemente importante
- Nada importante

Pense sobre as razões pelas quais você consome brigadeiro.
O quanto essa afirmação é importante para você "...porque eu cresci comendo isso"

- Extremamente importante
- Muito importante
- Moderadamente importante
- Levemente importante
- Nada importante

Pense sobre as razões pelas quais você consome brigadeiro.
O quanto essa afirmação é importante para você "...porque eu curto"

- Extremamente importante
- Muito importante
- Moderadamente importante
- Levemente importante
- Nada importante

Pense sobre as razões pelas quais você consome brigadeiro.
O quanto essa afirmação é importante para você "...porque eu estou faminto"

- Extremamente importante
- Muito importante
- Moderadamente importante
- Levemente importante

- Nada importante

Pense sobre as razões pelas quais você consome brigadeiro.
O quanto essa afirmação é importante para você "...porque eu tenho o hábito de comer"

- Extremamente importante
- Muito importante
- Moderadamente importante
- Levemente importante
- Nada importante

Pense sobre as razões pelas quais você consome brigadeiro.
O quanto essa afirmação é importante para você "...porque eu tenho vontade de comer isso"

- Extremamente importante
- Muito importante
- Moderadamente importante
- Levemente importante
- Nada importante

Pense sobre as razões pelas quais você consome brigadeiro.
O quanto essa afirmação é importante para você "...porque é fácil preparar"

- Extremamente importante
- Muito importante
- Moderadamente importante
- Levemente importante
- Nada importante

Pense sobre as razões pelas quais você consome brigadeiro.
O quanto essa afirmação é importante para você "...porque eu estou triste"

- Extremamente importante
- Muito importante
- Moderadamente importante
- Levemente importante
- Nada importante

Pense sobre as razões pelas quais você consome brigadeiro.
O quanto essa afirmação é importante para você "...porque tem pouca gordura"

- Extremamente importante
- Muito importante
- Moderadamente importante
- Levemente importante
- Nada importante

Pense sobre as razões pelas quais você consome brigadeiro.
O quanto essa afirmação é importante para você "...porque eu vi propaganda em algum lugar ou vi na TV"

- Extremamente importante
- Muito importante
- Moderadamente importante
- Levemente importante
- Nada importante

Pense sobre as razões pelas quais você consome brigadeiro.
O quanto essa afirmação é importante para você "...porque deixa as reuniões sociais mais agradáveis"

- Extremamente importante
- Muito importante
- Moderadamente importante
- Levemente importante
- Nada importante

Pense sobre as razões pelas quais você consome brigadeiro.
O quanto essa afirmação é importante para você "...porque eu devo comer"

- Extremamente importante
- Muito importante
- Moderadamente importante
- Levemente importante
- Nada importante

Pense sobre as razões pelas quais você consome brigadeiro.
O quanto essa afirmação é importante para você "...porque os outros gostam"

- Extremamente importante
- Muito importante
- Moderadamente importante
- Levemente importante
- Nada importante

Pense sobre as razões pelas quais você consome brigadeiro.
O quanto essa afirmação é importante para você "...porque eu me sinto sozinho"

- Extremamente importante
- Muito importante
- Moderadamente importante
- Levemente importante
- Nada importante

Pense sobre as razões pelas quais você consome brigadeiro.
O quanto essa afirmação é importante para você "...porque eu controlo meu peso"

- Extremamente importante
- Muito importante
- Moderadamente importante
- Levemente importante
- Nada importante

Pense sobre as razões pelas quais você consome brigadeiro.
O quanto essa afirmação é importante para você "...porque não contém substâncias perigosas (por exemplo, agrotóxicos, poluentes, antibióticos)"

- Extremamente importante
- Muito importante
- Moderadamente importante
- Levemente importante
- Nada importante

Pense sobre as razões pelas quais você consome brigadeiro.
O quanto essa afirmação é importante para você "...porque é mais conveniente"

- Extremamente importante
- Muito importante
- Moderadamente importante
- Levemente importante
- Nada importante

Pense sobre as razões pelas quais você consome brigadeiro.
O quanto essa afirmação é importante para você "...porque eu preciso de energia"

- Extremamente importante
- Muito importante
- Moderadamente importante
- Levemente importante
- Nada importante

Pense sobre as razões pelas quais você consome brigadeiro.
O quanto essa afirmação é importante para você "...porque me é familiar"

- Extremamente importante
- Muito importante
- Moderadamente importante
- Levemente importante
- Nada importante

Pense sobre as razões pelas quais você consome brigadeiro.
O quanto essa afirmação é importante para você "...porque eu gosto"

- Extremamente importante
- Muito importante
- Moderadamente importante
- Levemente importante
- Nada importante

Pense sobre as razões pelas quais você consome brigadeiro.
O quanto essa afirmação é importante para você "...porque me sacia de forma agradável"

- Extremamente importante
- Muito importante
- Moderadamente importante
- Levemente importante

- Nada importante

Pense sobre as razões pelas quais você consome brigadeiro.
O quanto essa afirmação é importante para você "...para compensar"

- Extremamente importante
- Muito importante
- Moderadamente importante
- Levemente importante
- Nada importante

Pense sobre as razões pelas quais você consome brigadeiro.
O quanto essa afirmação é importante para você "...porque não faz parte das tradições como Natal, Ano Novo e ocasiões especiais"

- Extremamente importante
- Muito importante
- Moderadamente importante
- Levemente importante
- Nada importante

Pense sobre as razões pelas quais você consome brigadeiro.
O quanto essa afirmação é importante para você "...porque faz parte de certas situações"

- Extremamente importante
- Muito importante
- Moderadamente importante
- Levemente importante
- Nada importante

Pense sobre as razões pelas quais você consome brigadeiro.
O quanto essa afirmação é importante para você "...para manter uma dieta balanceada"

- Extremamente importante
- Muito importante
- Moderadamente importante
- Levemente importante
- Nada importante

Pense sobre as razões pelas quais você consome brigadeiro.
O quanto essa afirmação é importante para você "...porque é sociável"

- Extremamente importante
- Muito importante
- Moderadamente importante
- Levemente importante
- Nada importante

Pense sobre as razões pelas quais você consome brigadeiro.
O quanto essa afirmação é importante para você "...porque não é caro"

- Extremamente importante
- Muito importante
- Moderadamente importante
- Levemente importante
- Nada importante

Pense sobre as razões pelas quais você consome brigadeiro.
O quanto essa afirmação é importante para você "...porque me atraiu aparentemente (por exemplo, cores atraentes)"

- Extremamente importante
- Muito importante
- Moderadamente importante
- Levemente importante
- Nada importante

Pense sobre as razões pelas quais você consome brigadeiro.
O quanto essa afirmação é importante para você "...para não desapontar alguém que está tentando me agradar"

- Extremamente importante
- Muito importante
- Moderadamente importante
- Levemente importante
- Nada importante

Pense sobre as razões pelas quais você consome brigadeiro.
O quanto essa afirmação é importante para você "...porque é moda"

- Extremamente importante
- Muito importante
- Moderadamente importante
- Levemente importante
- Nada importante

Pense sobre as razões pelas quais você consome brigadeiro.
O quanto essa afirmação é importante para você "...porque está em promoção"

- Extremamente importante
- Muito importante
- Moderadamente importante
- Levemente importante

- Nada importante

Pense sobre as razões pelas quais você consome brigadeiro.
O quanto essa afirmação é importante para você "...porque me mantém em forma (por exemplo, vigoroso, dinâmico)"

- Extremamente importante
- Muito importante
- Moderadamente importante
- Levemente importante
- Nada importante

Pense sobre as razões pelas quais você consome brigadeiro.
O quanto essa afirmação é importante para você "...porque é a única escolha"

- Extremamente importante
- Muito importante
- Moderadamente importante
- Levemente importante
- Nada importante

Pense sobre as razões pelas quais você consome brigadeiro.
O quanto essa afirmação é importante para você "...porque eu quero comer todos os dias"

- Extremamente importante
- Muito importante
- Moderadamente importante
- Levemente importante
- Nada importante

Pense sobre as razões pelas quais você não consome brigadeiro.
O quanto essa afirmação é importante para você "...eu não acho que seja um lanche"

- Extremamente importante
- Muito importante
- Moderadamente importante
- Levemente importante
- Nada importante

Pense sobre as razões pelas quais você não consome brigadeiro.
O quanto essa afirmação é importante para você "...eu não gosto disso"

- Extremamente importante
- Muito importante
- Moderadamente importante
- Levemente importante
- Nada importante

Pense sobre as razões pelas quais você não consome brigadeiro.
O quanto essa afirmação é importante para você "...normalmente eu não como isso"

- Extremamente importante
- Muito importante
- Moderadamente importante
- Levemente importante
- Nada importante

Pense sobre as razões pelas quais você não consome brigadeiro.

O quanto essa afirmação é importante para você "...o tamanho da porção não é adequado"

- Extremamente importante
- Muito importante
- Moderadamente importante
- Levemente importante
- Nada importante

Pense sobre as razões pelas quais você não consome brigadeiro.
O quanto essa afirmação é importante para você "...não sustenta o suficiente"

- Extremamente importante
- Muito importante
- Moderadamente importante
- Levemente importante
- Nada importante

Pense sobre as razões pelas quais você não consome brigadeiro.
O quanto essa afirmação é importante para você "...não é saudável"

- Extremamente importante
- Muito importante
- Moderadamente importante
- Levemente importante
- Nada importante

Pense sobre as razões pelas quais você não consome brigadeiro.
O quanto essa afirmação é importante para você "...não é conveniente"

- Extremamente importante
- Muito importante

- Moderadamente importante
- Levemente importante
- Nada importante

Pense sobre as razões pelas quais você não consome brigadeiro.
O quanto essa afirmação é importante para você "...eu não quero me dar ao luxo"

- Extremamente importante
- Muito importante
- Moderadamente importante
- Levemente importante
- Nada importante

Pense sobre as razões pelas quais você não consome brigadeiro.
O quanto essa afirmação é importante para você "...não é apropriado para a situação"

- Extremamente importante
- Muito importante
- Moderadamente importante
- Levemente importante
- Nada importante

Pense sobre as razões pelas quais você não consome brigadeiro.
O quanto essa afirmação é importante para você "...não é um alimento que eu como perto de outras pessoas"

- Extremamente importante
- Muito importante
- Moderadamente importante
- Levemente importante
- Nada importante

Pense sobre as razões pelas quais você não consome brigadeiro.
O quanto essa afirmação é importante para você "...o preço está muito alto"

- Extremamente importante
- Muito importante
- Moderadamente importante
- Levemente importante
- Nada importante

Pense sobre as razões pelas quais você não consome brigadeiro.
O quanto essa afirmação é importante para você "...eu não gosto do jeito que parece"

- Extremamente importante
- Muito importante
- Moderadamente importante
- Levemente importante
- Nada importante

Pense sobre as razões pelas quais você não consome brigadeiro.
O quanto essa afirmação é importante para você "...muito rico em calorias"

- Extremamente importante
- Muito importante
- Moderadamente importante
- Levemente importante
- Nada importante

Pense sobre as razões pelas quais você não consome brigadeiro.
O quanto essa afirmação é importante para você "...eu não devo comer isso"

- Extremamente importante
- Muito importante
- Moderadamente importante
- Levemente importante
- Nada importante

Pense sobre as razões pelas quais você não consome brigadeiro.
O quanto essa afirmação é importante para você "...comer isso me faz parecer antiquado"

- Extremamente importante
- Muito importante
- Moderadamente importante
- Levemente importante
- Nada importante

Pense sobre as razões pelas quais você não consome brigadeiro.
O quanto essa afirmação é importante para você "...não é nada que eu queira comer agora"

- Extremamente importante
- Muito importante
- Moderadamente importante
- Levemente importante
- Nada importante

Pense sobre as razões pelas quais você não consome brigadeiro.
O quanto essa afirmação é importante para você "...eu comi recentemente e não quero comer a mesma comida com tanta frequência"

- Extremamente importante
- Muito importante

- Moderadamente importante
- Levemente importante
- Nada importante

Pense sobre as razões pelas quais você não consome brigadeiro.
O quanto essa afirmação é importante para você "...eu nunca escolheria isso porque eu gosto de comer a mesma comida todos os dias"

- Extremamente importante
- Muito importante
- Moderadamente importante
- Levemente importante
- Nada importante

Pense sobre as razões pelas quais você não consome brigadeiro.
O quanto essa afirmação é importante para você "...essa comida me faz sentir triste, solitário e frustrado"

- Extremamente importante
- Muito importante
- Moderadamente importante
- Levemente importante
- Nada importante

Pense sobre as razões pelas quais você não consome brigadeiro.
O quanto essa afirmação é importante para você "...não é orgânico"

- Extremamente importante
- Muito importante
- Moderadamente importante
- Levemente importante
- Nada importante

Powered by Qualtrics
