# Supplementary material for: Comparing Four Question Formats in Five Languages for On-Line Consumer Surveys
Source: Methods Protoc. 2020 Jul 14;3(3):49. doi: 10.3390/mps3030049 (PMC7563457; doi:10.3390/mps3030049)
Supplement: Supplementary file 1 [file mps-03-00049-s001.zip › supplementary/Figure S3 (China- Mandarin).pdf]

NSAS STATE
IVERSITY

Demographics

D1. 您的性别是？

- 男性
- 女性

D2. 您是在哪一年出生的？

- 1943 及以前
- 1944 - 1964
- 1965 -1979
- 1980 - 1994
- 1995 - 2001
- 2002 及以后

D3. 您的学历是

- 小学及以下
- 高中
- 大学、研究生及以上

D4. 包括您自己，您家里一共有几位？

- 1
- 2

- 3
- 4
- 5个或者超过5个

D5. 你家里有几位小于18岁的家庭成员？

- 0
- 1
- 2
- 3
- 4个或者超过4个

QC.
为了保证调查数据的质量， 我们希望可以获得您最准确的意见，所以您对每一个问题的谨慎回答进而提供最好的答案对我们很重要 。

您可以承诺为本次调查中的问题提供具体和诚实的答案吗？

- 我会提供我最好的答案
- 我不会提供最好的答案
- 我不能保证能否提供最好的答案

Food Involvement

INTRO. 接下来，您将完成一个有关食品行为的调查问卷。

请根据您的对如下表述的 认同程度回答下面的问题：

Q1. 我每天不太去想关于食物的事

- 极度不同意
- 不同意
- 有一点不同意
- 不同意也不反对
- 有一点同意
- 同意
- 极度同意

Q2. 做饭并不是很有趣

- 极度不同意
- 不同意
- 有一点不同意
- 不同意也不反对
- 有一点同意
- 同意
- 极度同意

Q3. 我喜欢谈论我吃了什么或者要吃什么

- 极度不同意
- 不同意
- 有一点不同意
- 不同意也不反对
- 有一点同意
- 同意
- 极度同意

Q4. 相对于其他每天要做的决定来说，选择吃什么这件事对我来说并不是那么重要

- 极度不同意
- 不同意
- 有一点不同意
- 不同意也不反对
- 有一点同意
- 同意
- 极度同意

Q5. 我旅游时，最期待的事情之一就是吃当地的食物

- 极度不同意
- 不同意
- 有一点不同意
- 不同意也不反对
- 有一点同意
- 同意
- 极度同意

Q6. 吃饭后我做大部分或者全部的清洁工作

- 极度不同意
- 不同意
- 有一点不同意
- 不同意也不反对
- 有一点同意
- 同意
- 极度同意

Q7. 我喜欢为别人或自己做饭

- 极度不同意
- 不同意
- 有一点不同意
- 不同意也不反对
- 有一点同意
- 同意
- 极度同意

Q8. 出去吃饭时，我不思考或者谈论我的食物味道怎么样

- 极度不同意
- 不同意
- 有一点不同意
- 不同意也不反对
- 有一点同意
- 同意
- 极度同意

Q9. 我不喜欢混合或切碎了的食物

- 极度不同意
- 不同意
- 有一点不同意
- 不同意也不反对
- 有一点同意
- 同意
- 极度同意

Q10. 我为自己购买大部分或全部的食物

- 极度不同意
- 不同意
- 有一点不同意
- 不同意也不反对
- 有一点同意
- 同意
- 极度同意

Q11. 我不洗碗或者清理餐桌

- 极度不同意
- 不同意
- 有一点不同意
- 不同意也不反对
- 有一点同意
- 同意
- 极度同意

Q12. 我在乎饭桌有没有整齐地整理好

- 极度不同意
- 不同意
- 有一点不同意
- 不同意也不反对
- 有一点同意
- 同意
- 极度同意

Health and Taste Attitude

Q13. 我很在乎食物是否健康

- 极度不同意
- 不同意
- 有一点不同意
- 不同意也不反对
- 有一点同意
- 同意
- 极度同意

Q14. 我总是追随一个健康平衡的饮食

- 极度不同意
- 不同意
- 有一点不同意
- 不同意也不反对
- 有一点同意
- 同意
- 极度同意

Q15. 低脂对我很重要

- 极度不同意
- 不同意
- 有一点不同意
- 不同意也不反对
- 有一点同意
- 同意
- 极度同意

Q16. 高维他命和高矿物质含量对我每日饮食很重要

- 极度不同意
- 不同意
- 有一点不同意
- 不同意也不反对
- 有一点同意
- 同意
- 极度同意

Q17. 我吃我喜欢的，我不担心食物健不健康

- 极度不同意
- 不同意
- 有一点不同意
- 不同意也不反对
- 有一点同意
- 同意
- 极度同意

Q18. 我不回避任何食物，即使他会使我的胆固醇升高

- 极度不同意
- 不同意
- 有一点不同意
- 不同意也不反对
- 有一点同意
- 同意
- 极度同意

Q19. 食物的健康与否对我选择食物有很小的影响

- 极度不同意
- 不同意
- 有一点不同意
- 不同意也不反对
- 有一点同意
- 同意
- 极度同意

Q20. 零食的健康与否对我来说都一样

- 极度不同意
- 不同意
- 有一点不同意
- 不同意也不反对
- 有一点同意
- 同意
- 极度同意

Q21. 我不相信食物总是快乐的来源

- 极度不同意
- 不同意
- 有一点不同意
- 不同意也不反对
- 有一点同意
- 同意
- 极度同意

Q22. 食物的外观对我来说无所谓

- 极度不同意
- 不同意
- 有一点不同意
- 不同意也不反对
- 有一点同意
- 同意
- 极度同意

Q23. 对我来说，不管是周末还是非周末，吃好吃的食物都是一件很重要的事

- 极度不同意
- 不同意
- 有一点不同意
- 不同意也不反对
- 有一点同意
- 同意
- 极度同意

Q24. 我吃饭时专注于享受食物的味道

- 极度不同意
- 不同意
- 有一点不同意
- 不同意也不反对
- 有一点同意
- 同意
- 极度同意

Q25. 即便我不喜欢食物的味道我也会把我的食物吃完

- 极度不同意
- 不同意
- 有一点不同意
- 不同意也不反对
- 有一点同意
- 同意
- 极度同意

Q26. 周末的一个重要部分就是吃美味的食物

- 极度不同意
- 不同意
- 有一点不同意
- 不同意也不反对
- 有一点同意
- 同意
- 极度同意

Neophobia

Q27. 我不断去尝试新的和不同的食物

- 极度不同意
- 不同意
- 有一点不同意
- 不同意也不反对
- 有一点同意
- 同意

- 极度同意

Q28. 我不信任新的没有见过的事物

- 极度不同意
- 不同意
- 有一点不同意
- 不同意也不反对
- 有一点同意
- 同意
- 极度同意

Q29. 如果我不知道食物里有什么， 我不会尝试

- 极度不同意
- 不同意
- 有一点不同意
- 不同意也不反对
- 有一点同意
- 同意
- 极度同意

Q30. 我喜欢不同国家的食物

- 极度不同意
- 不同意
- 有一点不同意
- 不同意也不反对
- 有一点同意
- 同意

- 极度同意

Q31. 不同民族的食物看起来太奇怪了以至于无法尝试

- 极度不同意
- 不同意
- 有一点不同意
- 不同意也不反对
- 有一点同意
- 同意
- 极度同意

Q32. 在聚会时我会去尝试一个新食物

- 极度不同意
- 不同意
- 有一点不同意
- 不同意也不反对
- 有一点同意
- 同意
- 极度同意

Q33. 我害怕尝试没吃过的食物

- 极度不同意
- 不同意
- 有一点不同意
- 不同意也不反对
- 有一点同意
- 同意

- 极度同意

Q34. 我对我的食物很挑剔

- 极度不同意
- 不同意
- 有一点不同意
- 不同意也不反对
- 有一点同意
- 同意
- 极度同意

Q35. 我几乎什么都吃

- 极度不同意
- 不同意
- 有一点不同意
- 不同意也不反对
- 有一点同意
- 同意
- 极度同意

Q36. 我喜欢尝试新的不同民族风味的饭店

- 极度不同意
- 不同意
- 有一点不同意
- 不同意也不反对
- 有一点同意
- 同意

- 极度同意

Reasons for Starchy food choices(CATA)

Q84.
您吃白米饭吗?

- 是的， 我吃
- 不是， 我不吃

Q99.
请告诉我们您吃白米饭的原因（请选择所有适用的选项）

- ... 因为它让我在别人面前看起来很好
- ... 因为我喜欢它
- ... 因为吃它让我很享受
- ... 为了奖励自己
- ... 因为我关注我的体重
- ... 因为我很沮丧
- ... 因为它使社交聚会更加舒适
- ... 保持均衡饮食
- ... 因为我熟悉它
- ... 因为不吃它会不礼貌
- ... 因为我对它有食欲
- ... 因为它很健康
- ... 因为它最方便
- ... 因为我应该吃它
- ... 因为其他人喜欢它
- ... 因为它的味道很好
- ... 因为它很容易准备
- ... 因为脂肪含量低

- ☐ ... 因为我需要能量
- ☐ ... 因为它准备起来很快
- ☐ ... 因为它是天然的（例如没有经过基因改造）
- ☐ ... 因为我很伤心
- ☐ ...因为我饿了
- ☐ ... 为了纵容自己
- ☐ ... 因为它很新潮
- ☐ ... 因为它的卡路里很低
- ☐ ... 因为它不含有害物质（例如杀虫剂，污染物，抗生素）
- ☐ ...因为我习惯吃它
- ☐ ... 这样我就可以和其他人共度时光
- ☐ ... 因为我不想再花更多的钱
- ☐ ... 因为我从广告或在电视上看过它
- ☐ ... 因为我经常吃它
- ☐ ... 因为我想每天都吃它
- ☐ ... 避免让那些试图让我开心的人失望
- ☐ ... 因为它能令我感到愉悦的饱腹感
- ☐ ... 因为它帮助我社交
- ☐ ... 因为它适用于特定场合
- ☐ ... 因为这是唯一的选择
- ☐ ... 因为它的呈现方式很有吸引力（例如包装）
- ☐ ... 因为它自然而然地吸引了我（吸引人的颜色）
- ☐ ... 因为它打折
- ☐ ... 因为我从小吃到大
- ☐ ... 出于传统（例如家庭传统，特殊场合）
- ☐ ... 因为它是有机的
- ☐ ... 因为它让我保持身材
- ☐ ... 因为我感到孤独
- ☐ ... 因为它的价格不贵

Q88. 请告诉我们您不吃白米饭的原因（请选择所有适用的选项）

- ☐ 我不应该吃它
- ☐ 它的分量大小不合适
- ☐ 我不经常吃它
- ☐ 有其他人在的时候我不吃它
- ☐ 价格太高
- ☐ 它不适用于特定场合
- ☐ 我不喜欢它看起来的样子
- ☐ 它不方便
- ☐ 我不想纵容自己
- ☐ 吃它显得我很土
- ☐ 我不认为它是一种零食
- ☐ 我不喜欢它
- ☐ 我最近刚吃过， 我不想太经常吃同样的东西
- ☐ 它不是有机的
- ☐ 它不健康
- ☐ 我绝不选它。 我喜欢每天吃一样的食物
- ☐ 它不足以填饱肚子
- ☐ 这个食物让我感到伤心、孤单或沮丧
- ☐ 它卡路里太高
- ☐ 它不是我在那个时刻想吃的东西

Reasons for Protein foods Choices(CATA)

Q168.
您吃红烧肉吗?

- ○ 是的， 我吃
- ○ 不是， 我不吃

Q169.
请告诉我们您吃红烧肉的原因（请选择所有适用的选项）

- ☐ ... 因为它的卡路里很低
- ☐ ...因为我饿了
- ☐ ... 因为我关注我的体重
- ☐ ... 因为这是唯一的选择
- ☐ ... 因为它很新潮
- ☐ ... 因为它适用于特定场合
- ☐ ... 因为它很健康
- ☐ ... 因为我从广告或在电视上看过它
- ☐ ... 因为它是天然的（例如没有经过基因改造）
- ☐ ... 因为我应该吃它
- ☐ ... 因为它的呈现方式很有吸引力（例如包装）
- ☐ ... 因为它让我在别人面前看起来很好
- ☐ ... 因为我想每天都吃它
- ☐ ... 因为它能令我感到愉悦的饱腹感
- ☐ ... 因为它打折
- ☐ ... 因为我感到孤独
- ☐ ... 因为我不想再花更多的钱
- ☐ ... 因为它不含有害物质（例如杀虫剂，污染物，抗生素）
- ☐ ... 因为它使社交聚会更加舒适
- ☐ ... 因为它的价格不贵
- ☐ ... 因为我对它有食欲
- ☐ ... 因为它是有机的
- ☐ ... 避免让那些试图让我开心的人失望
- ☐ ... 因为它的味道很好
- ☐ ... 因为我熟悉它
- ☐ ... 这样我就可以和其他人共度时光
- ☐ ... 保持均衡饮食
- ☐ ... 为了纵容自己

- ☐ ... 因为我很伤心
- ☐ ... 因为其他人喜欢它
- ☐ ... 因为我从小吃到大
- ☐ ... 因为脂肪含量低
- ☐ ... 因为吃它让我很享受
- ☐ ... 为了奖励自己
- ☐ ... 出于传统（例如家庭传统，特殊场合）
- ☐ ... 因为我经常吃它
- ☐ ... 因为它很容易准备
- ☐ ... 因为它自然而然地吸引了我（吸引人的颜色）
- ☐ ... 因为我需要能量
- ☐ ... 因为它帮助我社交
- ☐ ... 因为它让我保持身材
- ☐ ... 因为不吃它会不礼貌
- ☐ ... 因为它准备起来很快
- ☐ ...因为我习惯吃它
- ☐ ... 因为我喜欢它
- ☐ ... 因为我很沮丧
- ☐ ... 因为它最方便

Q170. 请告诉我们您不吃红烧肉的原因（请选择所有适用的选项）

- ☐ 它不足以填饱肚子
- ☐ 我最近刚吃过， 我不想太经常吃同样的东西
- ☐ 它不适用于特定场合
- ☐ 我不应该吃它
- ☐ 它的分量大小不合适
- ☐ 它卡路里太高
- ☐ 我绝不选它。 我喜欢每天吃一样的食物
- ☐ 有其他人在的时候我不吃它

- ☐ 我不经常吃它
- ☐ 这个食物让我感到伤心、孤单或沮丧
- ☐ 我不认为它是一种零食
- ☐ 吃它显得我很土
- ☐ 我不喜欢它
- ☐ 它不是有机的
- ☐ 它不是我在那个时刻想吃的东西
- ☐ 价格太高
- ☐ 我不想纵容自己
- ☐ 它不健康
- ☐ 我不喜欢它看起来的样子
- ☐ 它不方便

Reasons for Dairy Food Choices(CATA)

Q171.

您吃豆浆吗?

- ○ 是的， 我吃
- ○ 不是， 我不吃

Q172.

请告诉我们您吃豆浆的原因（请选择所有适用的选项）

- ☐ ... 因为我对它有食欲
- ☐ ... 因为它打折
- ☐ ... 因为我熟悉它
- ☐ ... 因为它最方便
- ☐ ... 因为它的味道很好
- ☐ ... 因为脂肪含量低
- ☐ ... 因为它不含有害物质（例如杀虫剂，污染物，抗生素）

- ☐ ... 因为它很健康
- ☐ ... 因为我想每天都吃它
- ☐ ... 因为我很伤心
- ☐ ... 这样我就可以和其他人共度时光
- ☐ ... 因为其他人喜欢它
- ☐ ...因为我习惯吃它
- ☐ ... 因为这是唯一的选择
- ☐ ... 因为我关注我的体重
- ☐ ... 因为它的卡路里很低
- ☐ ... 因为我从小吃到大
- ☐ ... 因为我应该吃它
- ☐ ... 因为我不想再花更多的钱
- ☐ ... 因为它帮助我社交
- ☐ ... 因为吃它让我很享受
- ☐ ... 因为它使社交聚会更加舒适
- ☐ ... 因为不吃它会不礼貌
- ☐ ... 保持均衡饮食
- ☐ ... 出于传统（例如家庭传统，特殊场合）
- ☐ ... 因为我喜欢它
- ☐ ... 因为我经常吃它
- ☐ ... 因为它让我保持身材
- ☐ ... 因为它是天然的（例如没有经过基因改造）
- ☐ ... 因为我很沮丧
- ☐ ... 因为它很容易准备
- ☐ ... 因为它的价格不贵
- ☐ ... 因为它的呈现方式很有吸引力（例如包装）
- ☐ ... 避免让那些试图让我开心的人失望
- ☐ ... 因为我需要能量
- ☐ ... 因为它适用于特定场合
- ☐ ...因为我饿了

- ☐ ... 为了奖励自己
- ☐ ... 为了纵容自己
- ☐ ... 因为它自然而然地吸引了我（吸引人的颜色）
- ☐ ... 因为它让我在别人面前看起来很好
- ☐ ... 因为它是有机的
- ☐ ... 因为我感到孤独
- ☐ ... 因为它准备起来很快
- ☐ ... 因为我从广告或在电视上看过它
- ☐ ... 因为它很新潮
- ☐ ... 因为它能令我感到愉悦的饱腹感

Q173. 请告诉我们您不吃豆浆的原因（请选择所有适用的选项）

- ☐ 它不是我在那个时刻想吃的东西
- ☐ 它卡路里太高
- ☐ 它不健康
- ☐ 我不认为它是一种零食
- ☐ 它不方便
- ☐ 我不喜欢它看起来的样子
- ☐ 吃它显得我很土
- ☐ 它不是有机的
- ☐ 我最近刚吃过， 我不想太经常吃同样的东西
- ☐ 价格太高
- ☐ 它不足以填饱肚子
- ☐ 它不适用于特定场合
- ☐ 我不喜欢它
- ☐ 有其他人在的时候我不吃它
- ☐ 我不经常吃它
- ☐ 这个食物让我感到伤心、孤单或沮丧
- ☐ 我不应该吃它

- ☐ 它的分量大小不合适
- ☐ 我不想纵容自己
- ☐ 我绝不选它。 我喜欢每天吃一样的食物

Reasons for Fruit Choices(CATA)

Q174.
您吃香蕉吗?

- ○ 是的， 我吃
- ○ 不是， 我不吃

Q175.
请告诉我们您吃香蕉的原因（请选择所有适用的选项）

- ☐ ... 因为它的呈现方式很有吸引力（例如包装）
- ☐ ... 因为这是唯一的选择
- ☐ ... 因为我经常吃它
- ☐ ... 因为它准备起来很快
- ☐ ... 因为脂肪含量低
- ☐ ... 因为它的味道很好
- ☐ ... 这样我就可以和其他人共度时光
- ☐ ... 因为它不含有害物质（例如杀虫剂，污染物，抗生素）
- ☐ ... 因为它让我保持身材
- ☐ ... 因为它很容易准备
- ☐ ... 因为我很伤心
- ☐ ... 因为它让我在别人面前看起来很好
- ☐ ... 因为我从广告或在电视上看过它
- ☐ ... 为了奖励自己
- ☐ ...因为我饿了
- ☐ ... 因为我想每天都吃它

- ☐ ... 因为它使社交聚会更加舒适
- ☐ ... 避免让那些试图让我开心的人失望
- ☐ ... 因为它自然而然地吸引了我（吸引人的颜色）
- ☐ ... 为了纵容自己
- ☐ ... 因为不吃它会不礼貌
- ☐ ... 因为我喜欢它
- ☐ ... 因为它适用于特定场合
- ☐ ... 出于传统（例如家庭传统，特殊场合）
- ☐ ... 因为其他人喜欢它
- ☐ ... 因为我从小吃到大
- ☐ ... 因为我需要能量
- ☐ ... 因为它能令我感到愉悦的饱腹感
- ☐ ... 因为我很沮丧
- ☐ ...因为我习惯吃它
- ☐ ... 因为它的价格不贵
- ☐ ... 因为我感到孤独
- ☐ ... 因为它打折
- ☐ ... 因为它的卡路里很低
- ☐ ... 因为我对它有食欲
- ☐ ... 因为我熟悉它
- ☐ ... 因为我关注我的体重
- ☐ ... 因为它是有机的
- ☐ ... 因为它最方便
- ☐ ... 因为它是天然的（例如没有经过基因改造）
- ☐ ... 因为我不想再花更多的钱
- ☐ ... 因为吃它让我很享受
- ☐ ... 因为它帮助我社交
- ☐ ... 保持均衡饮食
- ☐ ... 因为它很健康
- ☐ ... 因为它很新潮

☐ ... 因为我应该吃它

Q176. 请告诉我们您不吃香蕉的原因（请选择所有适用的选项）

☐ 我最近刚吃过， 我不想太经常吃同样的东西
☐ 它的分量大小不合适
☐ 这个食物让我感到伤心、孤单或沮丧
☐ 它不是我在那个时刻想吃的东西
☐ 它不方便
☐ 它不适用于特定场合
☐ 价格太高
☐ 我不应该吃它
☐ 它不是有机的
☐ 我不喜欢它
☐ 有其他人在的时候我不吃它
☐ 我不想纵容自己
☐ 我绝不选它。 我喜欢每天吃一样的食物
☐ 它卡路里太高
☐ 它不健康
☐ 它不足以填饱肚子
☐ 我不喜欢它看起来的样子
☐ 我不认为它是一种零食
☐ 吃它显得我很土
☐ 我不经常吃它

Reasons for Sweet food Choices(CATA)

Q177.
您吃麻球吗?

- ○ 是的， 我吃
- ○ 不是， 我不吃

Q178.

请告诉我们您吃麻球的原因（请选择所有适用的选项）

- ☐ ... 因为我很沮丧
- ☐ ... 因为我对它有食欲
- ☐ ... 因为我关注我的体重
- ☐ ... 因为我想每天都吃它
- ☐ ... 因为它打折
- ☐ ... 因为我应该吃它
- ☐ ...因为我习惯吃它
- ☐ ... 保持均衡饮食
- ☐ ... 因为我感到孤独
- ☐ ... 因为它很新潮
- ☐ ... 因为我从小吃到大
- ☐ ... 因为它的卡路里很低
- ☐ ...因为我饿了
- ☐ ... 因为它不含有害物质（例如杀虫剂，污染物，抗生素）
- ☐ ... 因为它让我保持身材
- ☐ ... 出于传统（例如家庭传统，特殊场合）
- ☐ ... 因为它适用于特定场合
- ☐ ... 因为我经常吃它
- ☐ ... 因为它是有机的
- ☐ ... 因为我需要能量
- ☐ ... 因为我熟悉它
- ☐ ... 因为它能令我感到愉悦的饱腹感
- ☐ ... 因为它准备起来很快
- ☐ ... 为了纵容自己
- ☐ ... 因为它最方便

- ☐ ... 因为我从广告或在电视上看过它
- ☐ ... 避免让那些试图让我开心的人失望
- ☐ ... 因为它自然而然地吸引了我（吸引人的颜色）
- ☐ ... 因为它的呈现方式很有吸引力（例如包装）
- ☐ ... 因为脂肪含量低
- ☐ ... 这样我就可以和其他人共度时光
- ☐ ... 因为它使社交聚会更加舒适
- ☐ ... 为了奖励自己
- ☐ ... 因为它让我在别人面前看起来很好
- ☐ ... 因为我很伤心
- ☐ ... 因为我喜欢它
- ☐ ... 因为其他人喜欢它
- ☐ ... 因为我不想再花更多的钱
- ☐ ... 因为不吃它会不礼貌
- ☐ ... 因为它帮助我社交
- ☐ ... 因为这是唯一的选择
- ☐ ... 因为它的价格不贵
- ☐ ... 因为它是天然的（例如没有经过基因改造）
- ☐ ... 因为它的味道很好
- ☐ ... 因为它很健康
- ☐ ... 因为吃它让我很享受
- ☐ ... 因为它很容易准备

Q179. 请告诉我们您不吃麻球的原因（请选择所有适用的选项）

- ☐ 吃它显得我很土
- ☐ 它不健康
- ☐ 我不经常吃它
- ☐ 我不应该吃它
- ☐ 它的分量大小不合适

- ☐ 我最近刚吃过， 我不想太经常吃同样的东西
- ☐ 我不喜欢它
- ☐ 它不是有机的
- ☐ 价格太高
- ☐ 它不方便
- ☐ 这个食物让我感到伤心、孤单或沮丧
- ☐ 我绝不选它。 我喜欢每天吃一样的食物
- ☐ 我不想纵容自己
- ☐ 它不适用于特定场合
- ☐ 它不足以填饱肚子
- ☐ 我不认为它是一种零食
- ☐ 有其他人在的时候我不吃它
- ☐ 我不喜欢它看起来的样子
- ☐ 它不是我在那个时刻想吃的东西
- ☐ 它卡路里太高

JAR & Liking

Q60.

您认为本问卷的长度如何?

- ○ 过短
- ○ 太短
- ○ 有点短
- ○ 适中
- ○ 有点长
- ○ 太长
- ○ 过长

Q62.

您对于填写完这份问卷有什麽感觉?

- 我非常喜欢
- 我喜欢
- 我没有特别的感觉
- 我不喜欢
- 我非常不喜欢

Reasons for Starchy food choices(Full CATA)

Q225.
您吃白米饭吗?

- 是的， 我吃
- 不是， 我不吃

Q226. 您吃白米饭是因为您习惯吃它吗?

- 是
- 不是

Q227. 您吃白米饭是因为它很健康吗?

- 是
- 不是

Q228. 您吃白米饭是因为它准备起来很快吗?

- 是
- 不是

Q229. 您吃白米饭是因为它的味道很好吗?

- 是
- 不是

Q230. 您吃白米饭是为了纵容自己吗?

- 是
- 不是

Q231. 您吃白米饭是因为它是天然的吗（例如没有经过基因改造的）?

- 是
- 不是

Q232. 您吃白米饭是因为您不想再花更多的钱吗?

- 是
- 不是

Q233. 您吃白米饭是因为它的卡路里很低吗?

- 是
- 不是

Q234. 您吃白米饭是因为您很沮丧吗?

- 是
- 不是

Q235. 您吃白米饭是因为它让您在别人面前看起来很好吗?

- 是
- 不是

Q236. 您吃白米饭是因为不吃它会不礼貌吗?

- 是
- 不是

Q237. 您吃白米饭是因为它的呈现方式很有吸引力吗（例如包装）?

- 是
- 不是

Q238. 您吃白米饭是因为它能让您和其他人共度时光吗?

- 是
- 不是

Q239. 您吃白米饭是因为它是有机的吗?

- 是
- 不是

Q240. 您吃白米饭是因为您从小一直吃它长大吗?

- 是
- 不是

Q241. 您吃白米饭是因为吃它让您很享受吗?

- 是
- 不是

Q242. 您吃白米饭是因为您饿了吗?

- 是
- 不是

Q243. 您吃白米饭是因为您经常吃它吗?

- 是
- 不是

Q244. 您吃白米饭是因为您对它有食欲吗?

- 是
- 不是

Q245. 您吃白米饭是因为它很容易准备吗?

- 是
- 不是

Q246. 您吃白米饭是因为您很伤心吗?

- 是
- 不是

Q247. 您吃白米饭是因为它的脂肪含量低吗?

- 是
- 不是

Q248. 您吃白米饭是因为您从广告或电视上看到过它吗?

- 是
- 不是

Q249. 您吃白米饭是因为它能使社交聚会更加舒适吗?

- 是
- 不是

Q250. 您吃白米饭是因为您应该吃它吗?

- 是
- 不是

Q251. 您吃白米饭是因为其他人喜欢它吗?

- 是
- 不是

Q252. 您吃白米饭是因为您感到孤独吗?

- 是
- 不是

Q253. 您吃白米饭是因为您关注您的体重吗?

- 是
- 不是

Q254. 您吃白米饭是因为它不含有害物质吗（例如杀虫剂、污染物、抗生素）?

- 是
- 不是

Q255. 您吃白米饭是因为它最方便吗?

- 是
- 不是

Q256. 您吃白米饭是因为您需要能量吗?

- 是
- 不是

Q257. 您吃白米饭是因为您对它熟悉吗?

- 是
- 不是

Q258. 您吃白米饭是因为您喜欢它吗?

- 是
- 不是

Q259. 您吃白米饭是因为它能让您感到愉悦的饱腹感吗?

- 是
- 不是

Q260. 您吃白米饭是为了奖励自己吗?

- 是
- 不是

Q261. 您吃白米饭是出于传统吗（例如家庭传统、特殊场合）?

- 是
- 不是

Q262. 您吃白米饭是因为它适用于特定场合吗?

- 是
- 不是

Q263. 您吃白米饭是为了保持均衡饮食吗?

- 是
- 不是

Q264. 您吃白米饭是因为它帮助您社交吗?

- 是
- 不是

Q265. 您吃白米饭是因为它的价格不贵吗?

- 是
- 不是

Q266. 您吃白米饭是因为它自然而然地吸引了您吗（例如它的颜色吸引了您）?

- 是
- 不是

Q267. 您吃白米饭是因为您不想使试图让你开心的人失望吗?

- 是
- 不是

Q268. 您吃白米饭是因为它很新潮吗?

- 是
- 不是

Q269. 您吃白米饭是因为它打折吗?

- 是
- 不是

Q270. 您吃白米饭是因为它能让你保持身材吗?

- 是
- 不是

Q271. 您吃白米饭是因为它是您的唯一选择吗?

- 是
- 不是

Q272. 您吃白米饭是因为您每天都想吃它吗?

- 是
- 不是

Q273. 您不吃白米饭是因为您不认为它是一种零食吗?

- 是
- 不是

Q274. 您不吃白米饭是因为您不喜欢它吗?

- 是
- 不是

Q275. 您不吃白米饭是因为您不经常吃它吗?

- 是
- 不是

Q276. 您不吃白米饭是因为它的分量大小不合适吗?

- 是
- 不是

Q277. 您不吃白米饭是因为它不足以填饱肚子吗?

- 是
- 不是

Q278. 您不吃白米饭是因为它不健康吗?

- 是
- 不是

Q279. 您不吃白米饭是因为它不方便吗?

- 是
- 不是

Q280. 您不吃白米饭是因为您不想纵容自己吗?

- 是
- 不是

Q281. 您不吃白米饭是因为它不适用于特定场合吗?

- 是
- 不是

Q282. 您不吃白米饭是因为有其他人在的时候您不吃它吗?

- 是
- 不是

Q283. 您不吃白米饭是因为它的价格太高吗?

- 是
- 不是

Q284. 您不吃白米饭是因为您不喜欢它看起来的样子吗?

- 是
- 不是

Q285. 您不吃白米饭是因为它的卡路里太高吗?

- 是
- 不是

Q286. 您不吃白米饭是因为您不应该吃吗（例如出于文化、宗教习俗）?

- 是
- 不是

Q287. 您不吃白米饭是因为您觉得吃它显得您很土吗?

- 是
- 不是

Q288. 您不吃白米饭是因为它不是您在那个时候想吃的东西吗?

- 是
- 不是

Q289. 您不吃白米饭是因为最近您刚吃过它，您不想太经常吃同样的东西吗?

- 是
- 不是

Q290. 您不吃白米饭是因为您每天喜欢吃一样的食物吗 （不包括白米饭的其他食物）?

- 是
- 不是

Q291. 您不吃白米饭是因为这个食物让您感到伤心、孤单或沮丧吗?

- 是
- 不是

Q292. 您不吃白米饭是因为它不是有机的吗?

- 是
- 不是

Reasons for Protein foods Choices(Full CATA)

Q293.
您吃红烧肉吗?

- 是的， 我吃
- 不是， 我不吃

Q294. 您吃红烧肉是因为您习惯吃它吗?

- 是
- 不是

Q295. 您吃红烧肉是因为它很健康吗?

- 是
- 不是

Q296. 您吃红烧肉是因为它准备起来很快吗?

- 是
- 不是

Q297. 您吃红烧肉是因为它的味道很好吗?

- 是
- 不是

Q298. 您吃红烧肉是为了纵容自己吗?

- 是
- 不是

Q299. 您吃红烧肉是因为它是天然的吗（例如没有经过基因改造的）?

- 是
- 不是

Q300. 您吃红烧肉是因为您不想再花更多的钱吗?

- 是
- 不是

Q301. 您吃红烧肉是因为它的卡路里很低吗?

- 是
- 不是

Q302. 您吃红烧肉是因为您很沮丧吗?

- 是
- 不是

Q303. 您吃红烧肉是因为它让您在别人面前看起来很好吗?

- 是
- 不是

Q304. 您吃红烧肉是因为不吃它会不礼貌吗?

- 是
- 不是

Q305. 您吃红烧肉是因为它的呈现方式很有吸引力吗（例如包装）?

- 是
- 不是

Q306. 您吃红烧肉是因为它能让您和其他人共度时光吗?

- 是
- 不是

Q307. 您吃红烧肉是因为它是有机的吗?

- 是
- 不是

Q308. 您吃红烧肉是因为您从小一直吃它长大吗?

- 是
- 不是

Q309. 您吃红烧肉是因为吃它让您很享受吗?

- 是
- 不是

Q310. 您吃红烧肉是因为您饿了吗?

- 是
- 不是

Q311. 您吃红烧肉是因为您经常吃它吗?

- 是
- 不是

Q312. 您吃红烧肉是因为您对它有食欲吗?

○ 是
○ 不是

Q313. 您吃红烧肉是因为它很容易准备吗?

○ 是
○ 不是

Q314. 您吃红烧肉是因为您很伤心吗?

○ 是
○ 不是

Q315. 您吃红烧肉是因为它的脂肪含量低吗?

○ 是
○ 不是

Q316. 您吃红烧肉是因为您从广告或电视上看到过它吗?

○ 是
○ 不是

Q317. 您吃红烧肉是因为它能使社交聚会更加舒适吗?

○ 是
○ 不是

Q318. 您吃红烧肉是因为您应该吃它吗?

- 是
- 不是

Q319. 您吃红烧肉是因为其他人喜欢它吗?

- 是
- 不是

Q320. 您吃红烧肉是因为您感到孤独吗?

- 是
- 不是

Q321. 您吃红烧肉是因为您关注您的体重吗?

- 是
- 不是

Q322. 您吃红烧肉是因为它不含有害物质吗（例如杀虫剂、污染物、抗生素）?

- 是
- 不是

Q323. 您吃红烧肉是因为它最方便吗?

- 是
- 不是

Q324. 您吃红烧肉是因为您需要能量吗?

是

不是

Q325. 您吃红烧肉是因为您对它熟悉吗?

是

不是

Q326. 您吃红烧肉是因为您喜欢它吗?

是

不是

Q327. 您吃红烧肉是因为它能让您感到愉悦的饱腹感吗?

是

不是

Q328. 您吃红烧肉是为了奖励自己吗?

是

不是

Q329. 您吃红烧肉是出于传统吗（例如家庭传统、特殊场合）?

是

不是

Q330. 您吃红烧肉是因为它适用于特定场合吗?

- 是
- 不是

Q331. 您吃红烧肉是为了保持均衡饮食吗?

- 是
- 不是

Q332. 您吃红烧肉是因为它帮助您社交吗?

- 是
- 不是

Q333. 您吃红烧肉是因为它的价格不贵吗?

- 是
- 不是

Q334. 您吃红烧肉是因为它自然而然地吸引了您吗（例如它的颜色吸引了您）?

- 是
- 不是

Q335. 您吃红烧肉是因为您不想使试图让你开心的人失望吗?

- 是
- 不是

Q336. 您吃红烧肉是因为它很新潮吗?

- 是
- 不是

Q337. 您吃红烧肉是因为它打折吗?

- 是
- 不是

Q338. 您吃红烧肉是因为它能让你保持身材吗?

- 是
- 不是

Q339. 您吃红烧肉是因为它是您的唯一选择吗?

- 是
- 不是

Q340. 您吃红烧肉是因为您每天都想吃它吗?

- 是
- 不是

Q341. 您不吃红烧肉是因为您不认为它是一种零食吗?

- 是
- 不是

Q342. 您不吃红烧肉是因为您不喜欢它吗?

- 是
- 不是

Q343. 您不吃红烧肉是因为您不经常吃它吗?

- 是
- 不是

Q344. 您不吃红烧肉是因为它的分量大小不合适吗?

- 是
- 不是

Q345. 您不吃红烧肉是因为它不足以填饱肚子吗?

- 是
- 不是

Q346. 您不吃红烧肉是因为它不健康吗?

- 是
- 不是

Q347. 您不吃红烧肉是因为它不方便吗?

- 是
- 不是

Q348. 您不吃红烧肉是因为您不想纵容自己吗?

是

不是

Q349. 您不吃红烧肉是因为它不适用于特定场合吗?

是

不是

Q350. 您不吃红烧肉是因为有其他人在的时候您不吃它吗?

是

不是

Q351. 您不吃红烧肉是因为它的价格太高吗?

是

不是

Q352. 您不吃红烧肉是因为您不喜欢它看起来的样子吗?

是

不是

Q353. 您不吃红烧肉是因为它的卡路里太高吗?

是

不是

Q354. 您不吃红烧肉是因为您不应该吃吗（例如出于文化、宗教习俗）?

- 是
- 不是

Q355. 您不吃红烧肉是因为您觉得吃它显得您很土吗?

- 是
- 不是

Q356. 您不吃红烧肉是因为它不是您在那个时候想吃的东西吗?

- 是
- 不是

Q357. 您不吃红烧肉是因为最近您刚吃过它，您不想太经常吃同样的东西吗?

- 是
- 不是

Q358. 您不吃红烧肉是因为您每天喜欢吃一样的食物吗（不包括红烧肉的其他食物）?

- 是
- 不是

Q359. 您不吃红烧肉是因为这个食物让您感到伤心、孤单或沮丧吗?

- 是
- 不是

Q360. 您不吃红烧肉是因为它不是有机的吗?

- 是
- 不是

Reasons for Dairy Food Choices(Full CATA)

Q361.
您吃豆浆吗?

- 是的， 我吃
- 不是， 我不吃

Q362. 您吃豆浆是因为您习惯吃它吗?

- 是
- 不是

Q363. 您吃豆浆是因为它很健康吗?

- 是
- 不是

Q364. 您吃豆浆是因为它准备起来很快吗?

- 是
- 不是

Q365. 您吃豆浆是因为它的味道很好吗?

- 是
- 不是

Q366. 您吃豆浆是为了纵容自己吗?

- 是
- 不是

Q367. 您吃豆浆是因为它是天然的吗（例如没有经过基因改造的）?

- 是
- 不是

Q368. 您吃豆浆是因为您不想再花更多的钱吗?

- 是
- 不是

Q369. 您吃豆浆是因为它的卡路里很低吗?

- 是
- 不是

Q370. 您吃豆浆是因为您很沮丧吗?

- 是
- 不是

Q371. 您吃豆浆是因为它让您在别人面前看起来很好吗?

- 是
- 不是

Q372. 您吃豆浆是因为不吃它会不礼貌吗?

- 是
- 不是

Q373. 您吃豆浆是因为它的呈现方式很有吸引力吗（例如包装）?

- 是
- 不是

Q374. 您吃豆浆是因为它能让您和其他人共度时光吗?

- 是
- 不是

Q375. 您吃豆浆是因为它是有机的吗?

- 是
- 不是

Q376. 您吃豆浆是因为您从小一直吃它长大吗?

- 是
- 不是

Q377. 您吃豆浆是因为吃它让您很享受吗?

- 是
- 不是

Q378. 您吃豆浆是因为您饿了吗?

- 是
- 不是

Q379. 您吃豆浆是因为您经常吃它吗?

- 是
- 不是

Q380. 您吃豆浆是因为您对它有食欲吗?

- 是
- 不是

Q381. 您吃豆浆是因为它很容易准备吗?

- 是
- 不是

Q382. 您吃豆浆是因为您很伤心吗?

- 是
- 不是

Q383. 您吃豆浆是因为它的脂肪含量低吗?

- 是
- 不是

Q384. 您吃豆浆是因为您从广告或电视上看到过它吗?

- 是
- 不是

Q385. 您吃豆浆是因为它能使社交聚会更加舒适吗?

- 是
- 不是

Q386. 您吃豆浆是因为您应该吃它吗?

- 是
- 不是

Q387. 您吃豆浆是因为其他人喜欢它吗?

- 是
- 不是

Q388. 您吃豆浆是因为您感到孤独吗?

- 是
- 不是

Q389. 您吃豆浆是因为您关注您的体重吗?

- 是
- 不是

Q390. 您吃豆浆是因为它不含有害物质吗（例如杀虫剂、污染物、抗生素）?

- 是
- 不是

Q391. 您吃豆浆是因为它最方便吗?

- 是
- 不是

Q392. 您吃豆浆是因为您需要能量吗?

- 是
- 不是

Q393. 您吃豆浆是因为您对它熟悉吗?

- 是
- 不是

Q394. 您吃豆浆是因为您喜欢它吗?

- 是
- 不是

Q395. 您吃豆浆是因为它能让您感到愉悦的饱腹感吗?

- 是
- 不是

Q396. 您吃豆浆是为了奖励自己吗?

- 是
- 不是

Q397. 您吃豆浆是出于传统吗（例如家庭传统、特殊场合）?

- 是
- 不是

Q398. 您吃豆浆是因为它适用于特定场合吗?

- 是
- 不是

Q399. 您吃豆浆是为了保持均衡饮食吗?

- 是
- 不是

Q400. 您吃豆浆是因为它帮助您社交吗?

- 是
- 不是

Q401. 您吃豆浆是因为它的价格不贵吗?

- 是
- 不是

Q402. 您吃豆浆是因为它自然而然地吸引了您吗（例如它的颜色吸引了您）？

- 是
- 不是

Q403. 您吃豆浆是因为您不想使试图让你开心的人失望吗?

- 是
- 不是

Q404. 您吃豆浆是因为它很新潮吗?

- 是
- 不是

Q405. 您吃豆浆是因为它打折吗?

- 是
- 不是

Q406. 您吃豆浆是因为它能让你保持身材吗?

- 是
- 不是

Q407. 您吃豆浆是因为它是您的唯一选择吗?

- 是
- 不是

Q408. 您吃豆浆是因为您每天都想吃它吗?

- 是
- 不是

Q409. 您不吃豆浆是因为您不认为它是一种零食吗?

- 是
- 不是

Q410. 您不吃豆浆是因为您不喜欢它吗?

- 是
- 不是

Q411. 您不吃豆浆是因为您不经常吃它吗?

- 是
- 不是

Q412. 您不吃豆浆是因为它的分量大小不合适吗?

- 是
- 不是

Q413. 您不吃豆浆是因为它不足以填饱肚子吗?

- 是
- 不是

Q414. 您不吃豆浆是因为它不健康吗?

- 是
- 不是

Q415. 您不吃豆浆是因为它不方便吗?

- 是
- 不是

Q416. 您不吃豆浆是因为您不想纵容自己吗?

- 是
- 不是

Q417. 您不吃豆浆是因为它不适用于特定场合吗?

- 是
- 不是

Q418. 您不吃豆浆是因为有其他人在的时候您不吃它吗?

- 是
- 不是

Q419. 您不吃豆浆是因为它的价格太高吗?

- 是
- 不是

Q420. 您不吃豆浆是因为您不喜欢它看起来的样子吗?

- 是
- 不是

Q421. 您不吃豆浆是因为它的卡路里太高吗?

- 是
- 不是

Q422. 您不吃豆浆是因为您不应该吃吗（例如出于文化、宗教习俗）?

- 是
- 不是

Q423. 您不吃豆浆是因为您觉得吃它显得您很土吗?

- 是
- 不是

Q424. 您不吃豆浆是因为它不是您在那个时候想吃的东西吗?

- 是
- 不是

Q425. 您不吃豆浆是因为最近您刚吃过它，您不想太经常吃同样的东西吗?

- 是
- 不是

Q426. 您不吃豆浆是因为您每天喜欢吃一样的食物吗（不包括豆浆的其他食物）?

- 是
- 不是

Q427. 您不吃豆浆是因为这个食物让您感到伤心、孤单或沮丧吗?

- 是
- 不是

Q428. 您不吃豆浆是因为它不是有机的吗?

- 是
- 不是

Reasons for Fruit Choices(Full CATA)

Q429.
您吃香蕉吗?

- 是的， 我吃
- 不是， 我不吃

Q430. 您吃香蕉是因为您习惯吃它吗?

- 是
- 不是

Q431. 您吃香蕉是因为它很健康吗?

- 是
- 不是

Q432. 您吃香蕉是因为它准备起来很快吗?

- 是
- 不是

Q433. 您吃香蕉是因为它的味道很好吗?

- 是
- 不是

Q434. 您吃香蕉是为了纵容自己吗?

- 是
- 不是

Q435. 您吃香蕉是因为它是天然的吗（例如没有经过基因改造的）?

- 是
- 不是

Q436. 您吃香蕉是因为您不想再花更多的钱吗?

- 是
- 不是

Q437. 您吃香蕉是因为它的卡路里很低吗?

- 是
- 不是

Q438. 您吃香蕉是因为您很沮丧吗?

- 是
- 不是

Q439. 您吃香蕉是因为它让您在别人面前看起来很好吗?

- 是
- 不是

Q440. 您吃香蕉是因为不吃它会不礼貌吗?

- 是
- 不是

Q441. 您吃香蕉是因为它的呈现方式很有吸引力吗（例如包装）?

- 是
- 不是

Q442. 您吃香蕉是因为它能让您和其他人共度时光吗?

- 是
- 不是

Q443. 您吃香蕉是因为它是有机的吗?

- 是
- 不是

Q444. 您吃香蕉是因为您从小一直吃它长大吗?

- 是
- 不是

Q445. 您吃香蕉是因为吃它让您很享受吗?

- 是
- 不是

Q446. 您吃香蕉是因为您饿了吗?

- 是
- 不是

Q447. 您吃香蕉是因为您经常吃它吗?

- 是
- 不是

Q448. 您吃香蕉是因为您对它有食欲吗?

- 是
- 不是

Q449. 您吃香蕉是因为它很容易准备吗?

- 是
- 不是

Q450. 您吃香蕉是因为您很伤心吗?

- 是
- 不是

Q451. 您吃香蕉是因为它的脂肪含量低吗?

- 是
- 不是

Q452. 您吃香蕉是因为您从广告或电视上看到过它吗?

- 是
- 不是

Q453. 您吃香蕉是因为它能使社交聚会更加舒适吗?

- 是
- 不是

Q454. 您吃香蕉是因为您应该吃它吗?

- 是
- 不是

Q455. 您吃香蕉是因为其他人喜欢它吗?

- 是
- 不是

Q456. 您吃香蕉是因为您感到孤独吗?

- 是
- 不是

Q457. 您吃香蕉是因为您关注您的体重吗?

- 是
- 不是

Q458. 您吃香蕉是因为它不含有害物质吗（例如杀虫剂、污染物、抗生素）?

- 是
- 不是

Q459. 您吃香蕉是因为它最方便吗?

- 是
- 不是

Q460. 您吃香蕉是因为您需要能量吗?

- 是
- 不是

Q461. 您吃香蕉是因为您对它熟悉吗?

- 是
- 不是

Q462. 您吃香蕉是因为您喜欢它吗?

- 是
- 不是

Q463. 您吃香蕉是因为它能让您感到愉悦的饱腹感吗?

- 是
- 不是

Q464. 您吃香蕉是为了奖励自己吗?

- 是
- 不是

Q465. 您吃香蕉是出于传统吗（例如家庭传统、特殊场合）?

- 是
- 不是

Q466. 您吃香蕉是因为它适用于特定场合吗?

- 是
- 不是

Q467. 您吃香蕉是为了保持均衡饮食吗?

○ 是
○ 不是

Q468. 您吃香蕉是因为它帮助您社交吗?

○ 是
○ 不是

Q469. 您吃香蕉是因为它的价格不贵吗?

○ 是
○ 不是

Q470. 您吃香蕉是因为它自然而然地吸引了您吗（例如它的颜色吸引了您）?

○ 是
○ 不是

Q471. 您吃香蕉是因为您不想使试图让你开心的人失望吗?

○ 是
○ 不是

Q472. 您吃香蕉是因为它很新潮吗?

○ 是
○ 不是

Q473. 您吃香蕉是因为它打折吗?

- 是
- 不是

Q474. 您吃香蕉是因为它能让你保持身材吗?

- 是
- 不是

Q475. 您吃香蕉是因为它是您的唯一选择吗?

- 是
- 不是

Q476. 您吃香蕉是因为您每天都想吃它吗?

- 是
- 不是

Q477. 您不吃香蕉是因为您不认为它是一种零食吗?

- 是
- 不是

Q478. 您不吃香蕉是因为您不喜欢它吗?

- 是
- 不是

Q479. 您不吃香蕉是因为您不经常吃它吗?

- 是
- 不是

Q480. 您不吃香蕉是因为它的分量大小不合适吗?

- 是
- 不是

Q481. 您不吃香蕉是因为它不足以填饱肚子吗?

- 是
- 不是

Q482. 您不吃香蕉是因为它不健康吗?

- 是
- 不是

Q483. 您不吃香蕉是因为它不方便吗?

- 是
- 不是

Q484. 您不吃香蕉是因为您不想纵容自己吗?

- 是
- 不是

Q485. 您不吃香蕉是因为它不适用于特定场合吗?

- 是
- 不是

Q486. 您不吃香蕉是因为有其他人在的时候您不吃它吗?

- 是
- 不是

Q487. 您不吃香蕉是因为它的价格太高吗?

- 是
- 不是

Q488. 您不吃香蕉是因为您不喜欢它看起来的样子吗?

- 是
- 不是

Q489. 您不吃香蕉是因为它的卡路里太高吗?

- 是
- 不是

Q490. 您不吃香蕉是因为您不应该吃吗(例如出于文化、宗教习俗)?

- 是
- 不是

Q491. 您不吃香蕉是因为您觉得吃它显得您很土吗?

- 是
- 不是

Q492. 您不吃香蕉是因为它不是您在那个时候想吃的东西吗?

- 是
- 不是

Q493. 您不吃香蕉是因为最近您刚吃过它，您不想太经常吃同样的东西吗?

- 是
- 不是

Q494. 您不吃香蕉是因为您每天喜欢吃一样的食物吗 （不包括香蕉的其他食物）?

- 是
- 不是

Q495. 您不吃香蕉是因为这个食物让您感到伤心、孤单或沮丧吗?

- 是
- 不是

Q496. 您不吃香蕉是因为它不是有机的吗?

- 是
- 不是

Reasons for Sweet food Choices(Full CATA)

Q497.
您吃麻球吗?

- 是的， 我吃
- 不是， 我不吃

Q498. 您吃麻球是因为您习惯吃它吗?

- 是
- 不是

Q499. 您吃麻球是因为它很健康吗?

- 是
- 不是

Q500. 您吃麻球是因为它准备起来很快吗?

- 是
- 不是

Q501. 您吃麻球是因为它的味道很好吗?

- 是
- 不是

Q502. 您吃麻球是为了纵容自己吗?

- 是
- 不是

Q503. 您吃麻球是因为它是天然的吗（例如没有经过基因改造的）？

- 是
- 不是

Q504. 您吃麻球是因为您不想再花更多的钱吗?

- 是
- 不是

Q505. 您吃麻球是因为它的卡路里很低吗?

- 是
- 不是

Q506. 您吃麻球是因为您很沮丧吗?

- 是
- 不是

Q507. 您吃麻球是因为它让您在别人面前看起来很好吗?

- 是
- 不是

Q508. 您吃麻球是因为不吃它会不礼貌吗?

- 是
- 不是

Q509. 您吃麻球是因为它的呈现方式很有吸引力吗（例如包装）？

- 是
- 不是

Q510. 您吃麻球是因为它能让您和其他人共度时光吗?

- 是
- 不是

Q511. 您吃麻球是因为它是有机的吗?

- 是
- 不是

Q512. 您吃麻球是因为您从小一直吃它长大吗?

- 是
- 不是

Q513. 您吃麻球是因为吃它让您很享受吗?

- 是
- 不是

Q514. 您吃麻球是因为您饿了吗?

- 是
- 不是

Q515. 您吃麻球是因为您经常吃它吗?

- 是
- 不是

Q516. 您吃麻球是因为您对它有食欲吗?

- 是
- 不是

Q517. 您吃麻球是因为它很容易准备吗?

- 是
- 不是

Q518. 您吃麻球是因为您很伤心吗?

- 是
- 不是

Q519. 您吃麻球是因为它的脂肪含量低吗?

- 是
- 不是

Q520. 您吃麻球是因为您从广告或电视上看到过它吗?

- 是
- 不是

Q521. 您吃麻球是因为它能使社交聚会更加舒适吗?

- 是
- 不是

Q522. 您吃麻球是因为您应该吃它吗?

- 是
- 不是

Q523. 您吃麻球是因为其他人喜欢它吗?

- 是
- 不是

Q524. 您吃麻球是因为您感到孤独吗?

- 是
- 不是

Q525. 您吃麻球是因为您关注您的体重吗?

- 是
- 不是

Q526. 您吃麻球是因为它不含有害物质吗（例如杀虫剂、污染物、抗生素）?

- 是
- 不是

Q527. 您吃麻球是因为它最方便吗?

- 是
- 不是

Q528. 您吃麻球是因为您需要能量吗?

- 是
- 不是

Q529. 您吃麻球是因为您对它熟悉吗?

- 是
- 不是

Q530. 您吃麻球是因为您喜欢它吗?

- 是
- 不是

Q531. 您吃麻球是因为它能让您感到愉悦的饱腹感吗?

- 是
- 不是

Q532. 您吃麻球是为了奖励自己吗?

- 是
- 不是

Q533. 您吃麻球是出于传统吗（例如家庭传统、特殊场合）?

- 是
- 不是

Q534. 您吃麻球是因为它适用于特定场合吗?

- 是
- 不是

Q535. 您吃麻球是为了保持均衡饮食吗?

- 是
- 不是

Q536. 您吃麻球是因为它帮助您社交吗?

- 是
- 不是

Q537. 您吃麻球是因为它的价格不贵吗?

- 是
- 不是

Q538. 您吃麻球是因为它自然而然地吸引了您吗（例如它的颜色吸引了您）?

- 是
- 不是

Q539. 您吃麻球是因为您不想使试图让你开心的人失望吗?

- 是
- 不是

Q540. 您吃麻球是因为它很新潮吗?

- 是
- 不是

Q541. 您吃麻球是因为它打折吗?

- 是
- 不是

Q542. 您吃麻球是因为它能让你保持身材吗?

- 是
- 不是

Q543. 您吃麻球是因为它是您的唯一选择吗?

- 是
- 不是

Q544. 您吃麻球是因为您每天都想吃它吗?

- 是
- 不是

Q545. 您不吃麻球是因为您不认为它是一种零食吗?

- 是
- 不是

Q546. 您不吃麻球是因为您不喜欢它吗?

- 是
- 不是

Q547. 您不吃麻球是因为您不经常吃它吗?

- 是
- 不是

Q548. 您不吃麻球是因为它的分量大小不合适吗?

- 是
- 不是

Q549. 您不吃麻球是因为它不足以填饱肚子吗?

- 是
- 不是

Q550. 您不吃麻球是因为它不健康吗?

- 是
- 不是

Q551. 您不吃麻球是因为它不方便吗?

- 是
- 不是

Q552. 您不吃麻球是因为您不想纵容自己吗?

- 是
- 不是

Q553. 您不吃麻球是因为它不适用于特定场合吗?

- 是
- 不是

Q554. 您不吃麻球是因为有其他人在的时候您不吃它吗?

- 是
- 不是

Q555. 您不吃麻球是因为它的价格太高吗?

- 是
- 不是

Q556. 您不吃麻球是因为您不喜欢它看起来的样子吗?

- 是
- 不是

Q557. 您不吃麻球是因为它的卡路里太高吗?

- 是
- 不是

Q558. 您不吃麻球是因为您不应该吃吗（例如出于文化、宗教习俗）?

- 是
- 不是

Q559. 您不吃麻球是因为您觉得吃它显得您很土吗?

- 是
- 不是

Q560. 您不吃麻球是因为它不是您在那个时候想吃的东西吗?

- 是
- 不是

Q561. 您不吃麻球是因为最近您刚吃过它，您不想太经常吃同样的东西吗?

- 是
- 不是

Q562. 您不吃麻球是因为您每天喜欢吃一样的食物吗 （不包括麻球的其他食物）?

- 是
- 不是

Q563. 您不吃麻球是因为这个食物让您感到伤心、孤单或沮丧吗?

- 是
- 不是

Q564. 您不吃麻球是因为它不是有机的吗?

- 是
- 不是

Reasons for Starchy food choices(RATA)

Q610.
您吃白米饭吗?

- 是的， 我吃
- 不是， 我不吃

Q611.
试回想您上一次吃白米饭，并请回答以下问题。
您吃白米饭的原因有哪些（请选择所有适用的选项）

- ... 因为它让我在别人面前看起来很好
- ... 因为我想每天都吃它
- ... 因为吃它让我很享受
- ... 因为我很沮丧
- ... 因为它准备起来很快
- ...因为我习惯吃它
- ... 因为我需要能量
- ... 这样我就可以和其他人共度时光

- ☐ ... 因为它很容易准备
- ☐ ... 为了纵容自己
- ☐ ...因为我饿了
- ☐ ... 因为它让我保持身材
- ☐ ... 因为我关注我的体重
- ☐ ... 因为它是天然的（例如没有经过基因改造）
- ☐ ... 保持均衡饮食
- ☐ ... 避免让那些试图让我开心的人失望
- ☐ ... 因为它很健康
- ☐ ... 因为它能令我感到愉悦的饱腹感
- ☐ ... 因为它的卡路里很低
- ☐ ... 因为它适用于特定场合
- ☐ ... 因为它自然而然地吸引了我（吸引人的颜色）
- ☐ ... 因为我感到孤独
- ☐ ... 因为我从小吃到大
- ☐ ... 因为我应该吃它
- ☐ ... 因为它的价格不贵
- ☐ ... 因为我不想再花更多的钱
- ☐ ... 因为不吃它会不礼貌
- ☐ ... 为了奖励自己
- ☐ ... 因为这是唯一的选择
- ☐ ... 因为我经常吃它
- ☐ ... 因为我对它有食欲
- ☐ ... 因为它的呈现方式很有吸引力（例如包装）
- ☐ ... 因为它是有机的
- ☐ ... 因为它帮助我社交
- ☐ ... 因为我很伤心
- ☐ ... 因为它的味道很好
- ☐ ... 因为其他人喜欢它
- ☐ ... 因为它打折

- ☐ ... 因为它很新潮
- ☐ ... 因为它最方便
- ☐ ... 因为我喜欢它
- ☐ ... 出于传统（例如家庭传统，特殊场合）
- ☐ ... 因为脂肪含量低
- ☐ ... 因为我熟悉它
- ☐ ... 因为我从广告或在电视上看过它
- ☐ ... 因为它使社交聚会更加舒适
- ☐ ... 因为它不含有害物质（例如杀虫剂，污染物，抗生素）

Q612. 您选择"...因为我习惯吃它"
您认为该陈述有多重要？

- ○ 极其重要
- ○ 非常重要
- ○ 一般重要
- ○ 有点重要
- ○ 完全不重要

Q613. 您选择"... 因为它很健康"
您认为该陈述有多重要？

- ○ 极其重要
- ○ 非常重要
- ○ 一般重要
- ○ 有点重要
- ○ 完全不重要

Q614. 您选择"... 因为它准备起来很快"
您认为该陈述有多重要？

- 极其重要
- 非常重要
- 一般重要
- 有点重要
- 完全不重要

Q615. 您选择"... 因为它的味道很好"
您认为该陈述有多重要？

- 极其重要
- 非常重要
- 一般重要
- 有点重要
- 完全不重要

Q616. 您选择"... 为了纵容自己"
您认为该陈述有多重要？

- 极其重要
- 非常重要
- 一般重要
- 有点重要
- 完全不重要

Q617. 您选择"... 因为它是天然的（例如没有经过基因改造）"
您认为该陈述有多重要？

- 极其重要
- 非常重要
- 一般重要

- 有点重要
- 完全不重要

Q618. 您选择"... 因为我不想再花更多的钱"
您认为该陈述有多重要？

- 极其重要
- 非常重要
- 一般重要
- 有点重要
- 完全不重要

Q619. 您选择"... 因为它的卡路里很低"
您认为该陈述有多重要？

- 极其重要
- 非常重要
- 一般重要
- 有点重要
- 完全不重要

Q620. 您选择"... 因为我很沮丧"
您认为该陈述有多重要？

- 极其重要
- 非常重要
- 一般重要
- 有点重要
- 完全不重要

Q621. 您选择"... 因为它让我在别人面前看起来很好"
您认为该陈述有多重要？

- 极其重要
- 非常重要
- 一般重要
- 有点重要
- 完全不重要

Q622. 您选择"... 因为不吃它会不礼貌"
您认为该陈述有多重要？

- 极其重要
- 非常重要
- 一般重要
- 有点重要
- 完全不重要

Q623. 您选择"... 因为它的呈现方式很有吸引力（例如包装）"
您认为该陈述有多重要？

- 极其重要
- 非常重要
- 一般重要
- 有点重要
- 完全不重要

Q624. 您选择"... 这样我就可以和其他人共度时光"
您认为该陈述有多重要？

- 极其重要

- 非常重要
- 一般重要
- 有点重要
- 完全不重要

Q625. 您选择"... 因为它是有机的"
您认为该陈述有多重要？

- 极其重要
- 非常重要
- 一般重要
- 有点重要
- 完全不重要

Q626. 您选择"... 因为我从小吃到大"
您认为该陈述有多重要？

- 极其重要
- 非常重要
- 一般重要
- 有点重要
- 完全不重要

Q627. 您选择"... 因为吃它让我很享受"
您认为该陈述有多重要？

- 极其重要
- 非常重要
- 一般重要
- 有点重要

- 完全不重要

Q628. 您选择"...因为我饿了"
您认为该陈述有多重要？

- 极其重要
- 非常重要
- 一般重要
- 有点重要
- 完全不重要

Q629. 您选择"... 因为我经常吃它"
您认为该陈述有多重要？

- 极其重要
- 非常重要
- 一般重要
- 有点重要
- 完全不重要

Q630. 您选择"... 因为我对它有食欲"
您认为该陈述有多重要？

- 极其重要
- 非常重要
- 一般重要
- 有点重要
- 完全不重要

Q631. 您选择"... 因为它很容易准备"

您认为该陈述有多重要？

- 极其重要
- 非常重要
- 一般重要
- 有点重要
- 完全不重要

Q632. 您选择"... 因为我很伤心"
您认为该陈述有多重要？

- 极其重要
- 非常重要
- 一般重要
- 有点重要
- 完全不重要

Q633. 您选择"... 因为脂肪含量低"
您认为该陈述有多重要？

- 极其重要
- 非常重要
- 一般重要
- 有点重要
- 完全不重要

Q634. 您选择"... 因为我从广告或在电视上看过它"
您认为该陈述有多重要？

- 极其重要
- 非常重要

- 一般重要
- 有点重要
- 完全不重要

Q635. 您选择"... 因为它使社交聚会更加舒适"
您认为该陈述有多重要？

- 极其重要
- 非常重要
- 一般重要
- 有点重要
- 完全不重要

Q636. 您选择"... 因为我应该吃它"
您认为该陈述有多重要？

- 极其重要
- 非常重要
- 一般重要
- 有点重要
- 完全不重要

Q637. 您选择"... 因为其他人喜欢它"
您认为该陈述有多重要？

- 极其重要
- 非常重要
- 一般重要
- 有点重要
- 完全不重要

Q638. 您选择"... 因为我感到孤独"
您认为该陈述有多重要？

- 极其重要
- 非常重要
- 一般重要
- 有点重要
- 完全不重要

Q639. 您选择"... 因为我关注我的体重"
您认为该陈述有多重要？

- 极其重要
- 非常重要
- 一般重要
- 有点重要
- 完全不重要

Q640. 您选择"... 因为它不含有害物质（例如杀虫剂，污染物，抗生素）"
您认为该陈述有多重要？

- 极其重要
- 非常重要
- 一般重要
- 有点重要
- 完全不重要

Q641. 您选择"... 因为它最方便"
您认为该陈述有多重要？

- 极其重要
- 非常重要
- 一般重要
- 有点重要
- 完全不重要

Q642. 您选择"... 因为我需要能量"
您认为该陈述有多重要？

- 极其重要
- 非常重要
- 一般重要
- 有点重要
- 完全不重要

Q643. 您选择"... 因为我熟悉它"
您认为该陈述有多重要？

- 极其重要
- 非常重要
- 一般重要
- 有点重要
- 完全不重要

Q644. 您选择"... 因为我喜欢它"
您认为该陈述有多重要？

- 极其重要
- 非常重要
- 一般重要

- 有点重要
- 完全不重要

Q645. 您选择"... 因为它能令我感到愉悦的饱腹感"
您认为该陈述有多重要？

- 极其重要
- 非常重要
- 一般重要
- 有点重要
- 完全不重要

Q646. 您选择"... 为了奖励自己"
您认为该陈述有多重要？

- 极其重要
- 非常重要
- 一般重要
- 有点重要
- 完全不重要

Q647. 您选择"... 出于传统（例如家庭传统，特殊场合）"
您认为该陈述有多重要？

- 极其重要
- 非常重要
- 一般重要
- 有点重要
- 完全不重要

Q648. 您选择"... 因为它适用于特定场合"
您认为该陈述有多重要？

- 极其重要
- 非常重要
- 一般重要
- 有点重要
- 完全不重要

Q649. 您选择"... 保持均衡饮食"
您认为该陈述有多重要？

- 极其重要
- 非常重要
- 一般重要
- 有点重要
- 完全不重要

Q650. 您选择"... 因为它帮助我社交"
您认为该陈述有多重要？

- 极其重要
- 非常重要
- 一般重要
- 有点重要
- 完全不重要

Q651. 您选择"... 因为它的价格不贵"
您认为该陈述有多重要？

- 极其重要

- 非常重要
- 一般重要
- 有点重要
- 完全不重要

Q652. 您选择"... 因为它自然而然地吸引了我（吸引人的颜色）"
您认为该陈述有多重要？

- 极其重要
- 非常重要
- 一般重要
- 有点重要
- 完全不重要

Q653. 您选择"... 避免让那些试图让我开心的人失望"
您认为该陈述有多重要？

- 极其重要
- 非常重要
- 一般重要
- 有点重要
- 完全不重要

Q654. 您选择"... 因为它很新潮"
您认为该陈述有多重要？

- 极其重要
- 非常重要
- 一般重要
- 有点重要

完全不重要

Q655. 您选择"... 因为它打折"
您认为该陈述有多重要？

- 极其重要
- 非常重要
- 一般重要
- 有点重要
- 完全不重要

Q656. 您选择"... 因为它让我保持身材"
您认为该陈述有多重要？

- 极其重要
- 非常重要
- 一般重要
- 有点重要
- 完全不重要

Q657. 您选择"... 因为这是唯一的选择"
您认为该陈述有多重要？

- 极其重要
- 非常重要
- 一般重要
- 有点重要
- 完全不重要

Q658. 您选择"... 因为我想每天都吃它"

您认为该陈述有多重要？

- 极其重要
- 非常重要
- 一般重要
- 有点重要
- 完全不重要

Q659. 请告诉我们您不吃白米饭的原因（请选择所有适用的选项）

- 它不是我在那个时刻想吃的东西
- 有其他人在的时候我不吃它
- 我不经常吃它
- 我绝不选它。 我喜欢每天吃一样的食物
- 它不足以填饱肚子
- 它卡路里太高
- 我不想纵容自己
- 它不是有机的
- 这个食物让我感到伤心、孤单或沮丧
- 价格太高
- 它不健康
- 我不认为它是一种零食
- 我不应该吃它
- 我不喜欢它看起来的样子
- 吃它显得我很土
- 它不适用于特定场合
- 我最近刚吃过， 我不想太经常吃同样的东西
- 它的分量大小不合适
- 它不方便
- 我不喜欢它

Q660. 您选择”我不认为它是一种零食“
您认为该陈述有多重要？

- 极其重要
- 非常重要
- 一般重要
- 有点重要
- 完全不重要

Q661. 您选择”我不喜欢它“
您认为该陈述有多重要？

- 极其重要
- 非常重要
- 一般重要
- 有点重要
- 完全不重要

Q662. 您选择”我不经常吃它“
您认为该陈述有多重要？

- 极其重要
- 非常重要
- 一般重要
- 有点重要
- 完全不重要

Q663. 您选择”它的分量大小不合适“
您认为该陈述有多重要？

- 极其重要

- 非常重要
- 一般重要
- 有点重要
- 完全不重要

Q664. 您选择”它不足以填饱肚子“
您认为该陈述有多重要？

- 极其重要
- 非常重要
- 一般重要
- 有点重要
- 完全不重要

Q665. 您选择”它不健康“
您认为该陈述有多重要？

- 极其重要
- 非常重要
- 一般重要
- 有点重要
- 完全不重要

Q666. 您选择”它不方便“
您认为该陈述有多重要？

- 极其重要
- 非常重要
- 一般重要
- 有点重要

- 完全不重要

Q667. 您选择”我不想纵容自己“
您认为该陈述有多重要？

- 极其重要
- 非常重要
- 一般重要
- 有点重要
- 完全不重要

Q668. 您选择”它不适用于特定场合“
您认为该陈述有多重要？

- 极其重要
- 非常重要
- 一般重要
- 有点重要
- 完全不重要

Q669. 您选择”有其他人在的时候我不吃它“
您认为该陈述有多重要？

- 极其重要
- 非常重要
- 一般重要
- 有点重要
- 完全不重要

Q670. 您选择”价格太高“

您认为该陈述有多重要？

- 极其重要
- 非常重要
- 一般重要
- 有点重要
- 完全不重要

Q671. 您选择”我不喜欢它看起来的样子“
您认为该陈述有多重要？

- 极其重要
- 非常重要
- 一般重要
- 有点重要
- 完全不重要

Q672. 您选择”它卡路里太高“
您认为该陈述有多重要？

- 极其重要
- 非常重要
- 一般重要
- 有点重要
- 完全不重要

Q673. 您选择”我不应该吃它“
您认为该陈述有多重要？

- 极其重要
- 非常重要

- 一般重要
- 有点重要
- 完全不重要

Q674. 您选择”吃它显得我很土“
您认为该陈述有多重要？

- 极其重要
- 非常重要
- 一般重要
- 有点重要
- 完全不重要

Q675. 您选择”它不是我在那个时刻想吃的东西“
您认为该陈述有多重要？

- 极其重要
- 非常重要
- 一般重要
- 有点重要
- 完全不重要

Q676. 您选择”我最近刚吃过， 我不想太经常吃同样的东西“
您认为该陈述有多重要？

- 极其重要
- 非常重要
- 一般重要
- 有点重要
- 完全不重要

Q677. 您选择”我绝不选它。 我喜欢每天吃一样的食物“
您认为该陈述有多重要？

- 极其重要
- 非常重要
- 一般重要
- 有点重要
- 完全不重要

Q678. 您选择”这个食物让我感到伤心、孤单或沮丧“
您认为该陈述有多重要？

- 极其重要
- 非常重要
- 一般重要
- 有点重要
- 完全不重要

Q679. 您选择”它不是有机的“
您认为该陈述有多重要？

- 极其重要
- 非常重要
- 一般重要
- 有点重要
- 完全不重要

Reasons for Protein foods Choices(RATA)

Q680.

您吃红烧肉吗?

- 是的， 我吃
- 不是， 我不吃

Q681.

试回想您上一次吃红烧肉， 并请回答以下问题。

您吃红烧肉的原因有哪些（请选择所有适用的选项）

- ... 因为我不想再花更多的钱
- ... 出于传统（例如家庭传统，特殊场合）
- ... 因为我需要能量
- ... 因为它是天然的（例如没有经过基因改造）
- ... 因为吃它让我很享受
- ... 因为它的呈现方式很有吸引力（例如包装）
- ...因为我习惯吃它
- ... 因为它准备起来很快
- ... 为了奖励自己
- ... 因为它很新潮
- ... 因为我经常吃它
- ... 因为我关注我的体重
- ... 因为我感到孤独
- ... 因为它能令我感到愉悦的饱腹感
- ... 为了纵容自己
- ... 因为它使社交聚会更加舒适
- ... 因为它不含有害物质（例如杀虫剂，污染物，抗生素）
- ... 因为我喜欢它
- ... 因为其他人喜欢它
- ... 因为它自然而然地吸引了我（吸引人的颜色）
- ... 因为它打折

- ☐ ... 因为我很伤心
- ☐ ... 因为我应该吃它
- ☐ ... 避免让那些试图让我开心的人失望
- ☐ ... 因为它的价格不贵
- ☐ ... 因为它很健康
- ☐ ... 因为脂肪含量低
- ☐ ... 这样我就可以和其他人共度时光
- ☐ ... 因为我对它有食欲
- ☐ ... 因为它是有机的
- ☐ ... 因为我从广告或在电视上看过它
- ☐ ... 因为它的味道很好
- ☐ ...因为我饿了
- ☐ ... 因为它帮助我社交
- ☐ ... 因为它的卡路里很低
- ☐ ... 因为它让我保持身材
- ☐ ... 因为我从小吃到大
- ☐ ... 因为它很容易准备
- ☐ ... 因为我想每天都吃它
- ☐ ... 因为我很沮丧
- ☐ ... 因为它最方便
- ☐ ... 因为我熟悉它
- ☐ ... 保持均衡饮食
- ☐ ... 因为它让我在别人面前看起来很好
- ☐ ... 因为不吃它会不礼貌
- ☐ ... 因为这是唯一的选择
- ☐ ... 因为它适用于特定场合

Q682. 您选择"...因为我习惯吃它"
您认为该陈述有多重要？

- 极其重要
- 非常重要
- 一般重要
- 有点重要
- 完全不重要

Q683. 您选择"... 因为它很健康"
您认为该陈述有多重要？

- 极其重要
- 非常重要
- 一般重要
- 有点重要
- 完全不重要

Q684. 您选择"... 因为它准备起来很快"
您认为该陈述有多重要？

- 极其重要
- 非常重要
- 一般重要
- 有点重要
- 完全不重要

Q685. 您选择"... 因为它的味道很好"
您认为该陈述有多重要？

- 极其重要
- 非常重要
- 一般重要

- 有点重要
- 完全不重要

Q686. 您选择"... 为了纵容自己"
您认为该陈述有多重要？

- 极其重要
- 非常重要
- 一般重要
- 有点重要
- 完全不重要

Q687. 您选择"... 因为它是天然的（例如没有经过基因改造）"
您认为该陈述有多重要？

- 极其重要
- 非常重要
- 一般重要
- 有点重要
- 完全不重要

Q688. 您选择"... 因为我不想再花更多的钱"
您认为该陈述有多重要？

- 极其重要
- 非常重要
- 一般重要
- 有点重要
- 完全不重要

Q689. 您选择"... 因为它的卡路里很低"
您认为该陈述有多重要？

- 极其重要
- 非常重要
- 一般重要
- 有点重要
- 完全不重要

Q690. 您选择"... 因为我很沮丧"
您认为该陈述有多重要？

- 极其重要
- 非常重要
- 一般重要
- 有点重要
- 完全不重要

Q691. 您选择"... 因为它让我在别人面前看起来很好"
您认为该陈述有多重要？

- 极其重要
- 非常重要
- 一般重要
- 有点重要
- 完全不重要

Q692. 您选择"... 因为不吃它会不礼貌"
您认为该陈述有多重要？

- 极其重要

- 非常重要
- 一般重要
- 有点重要
- 完全不重要

Q693. 您选择"... 因为它的呈现方式很有吸引力（例如包装）"
您认为该陈述有多重要？

- 极其重要
- 非常重要
- 一般重要
- 有点重要
- 完全不重要

Q694. 您选择"... 这样我就可以和其他人共度时光"
您认为该陈述有多重要？

- 极其重要
- 非常重要
- 一般重要
- 有点重要
- 完全不重要

Q695. 您选择"... 因为它是有机的"
您认为该陈述有多重要？

- 极其重要
- 非常重要
- 一般重要
- 有点重要

- 完全不重要

Q696. 您选择"... 因为我从小吃到大"
您认为该陈述有多重要？

- 极其重要
- 非常重要
- 一般重要
- 有点重要
- 完全不重要

Q697. 您选择"... 因为吃它让我很享受"
您认为该陈述有多重要？

- 极其重要
- 非常重要
- 一般重要
- 有点重要
- 完全不重要

Q698. 您选择"...因为我饿了"
您认为该陈述有多重要？

- 极其重要
- 非常重要
- 一般重要
- 有点重要
- 完全不重要

Q699. 您选择"... 因为我经常吃它"

您认为该陈述有多重要？

- 极其重要
- 非常重要
- 一般重要
- 有点重要
- 完全不重要

Q700. 您选择"... 因为我对它有食欲"
您认为该陈述有多重要？

- 极其重要
- 非常重要
- 一般重要
- 有点重要
- 完全不重要

Q701. 您选择"... 因为它很容易准备"
您认为该陈述有多重要？

- 极其重要
- 非常重要
- 一般重要
- 有点重要
- 完全不重要

Q702. 您选择"... 因为我很伤心"
您认为该陈述有多重要？

- 极其重要
- 非常重要

- 一般重要
- 有点重要
- 完全不重要

Q703. 您选择"... 因为脂肪含量低"
您认为该陈述有多重要？

- 极其重要
- 非常重要
- 一般重要
- 有点重要
- 完全不重要

Q704. 您选择"... 因为我从广告或在电视上看过它"
您认为该陈述有多重要？

- 极其重要
- 非常重要
- 一般重要
- 有点重要
- 完全不重要

Q705. 您选择"... 因为它使社交聚会更加舒适"
您认为该陈述有多重要？

- 极其重要
- 非常重要
- 一般重要
- 有点重要
- 完全不重要

Q706. 您选择"... 因为我应该吃它"
您认为该陈述有多重要？

- ○ 极其重要
- ○ 非常重要
- ○ 一般重要
- ○ 有点重要
- ○ 完全不重要

Q707. 您选择"... 因为其他人喜欢它"
您认为该陈述有多重要？

- ○ 极其重要
- ○ 非常重要
- ○ 一般重要
- ○ 有点重要
- ○ 完全不重要

Q708. 您选择"... 因为我感到孤独"
您认为该陈述有多重要？

- ○ 极其重要
- ○ 非常重要
- ○ 一般重要
- ○ 有点重要
- ○ 完全不重要

Q709. 您选择"... 因为我关注我的体重"
您认为该陈述有多重要？

- 极其重要
- 非常重要
- 一般重要
- 有点重要
- 完全不重要

Q710. 您选择"... 因为它不含有害物质（例如杀虫剂，污染物，抗生素）"
您认为该陈述有多重要？

- 极其重要
- 非常重要
- 一般重要
- 有点重要
- 完全不重要

Q711. 您选择"... 因为它最方便"
您认为该陈述有多重要？

- 极其重要
- 非常重要
- 一般重要
- 有点重要
- 完全不重要

Q712. 您选择"... 因为我需要能量"
您认为该陈述有多重要？

- 极其重要
- 非常重要
- 一般重要

- 有点重要
- 完全不重要

Q713. 您选择"... 因为我熟悉它"
您认为该陈述有多重要？

- 极其重要
- 非常重要
- 一般重要
- 有点重要
- 完全不重要

Q714. 您选择"... 因为我喜欢它"
您认为该陈述有多重要？

- 极其重要
- 非常重要
- 一般重要
- 有点重要
- 完全不重要

Q715. 您选择"... 因为它能令我感到愉悦的饱腹感"
您认为该陈述有多重要？

- 极其重要
- 非常重要
- 一般重要
- 有点重要
- 完全不重要

Q716. 您选择"... 为了奖励自己"
您认为该陈述有多重要？

- 极其重要
- 非常重要
- 一般重要
- 有点重要
- 完全不重要

Q717. 您选择"... 出于传统（例如家庭传统，特殊场合）"
您认为该陈述有多重要？

- 极其重要
- 非常重要
- 一般重要
- 有点重要
- 完全不重要

Q718. 您选择"... 因为它适用于特定场合"
您认为该陈述有多重要？

- 极其重要
- 非常重要
- 一般重要
- 有点重要
- 完全不重要

Q719. 您选择"... 保持均衡饮食"
您认为该陈述有多重要？

- 极其重要

- 非常重要
- 一般重要
- 有点重要
- 完全不重要

Q720. 您选择"... 因为它帮助我社交"
您认为该陈述有多重要？

- 极其重要
- 非常重要
- 一般重要
- 有点重要
- 完全不重要

Q721. 您选择"... 因为它的价格不贵"
您认为该陈述有多重要？

- 极其重要
- 非常重要
- 一般重要
- 有点重要
- 完全不重要

Q722. 您选择"... 因为它自然而然地吸引了我（吸引人的颜色）"
您认为该陈述有多重要？

- 极其重要
- 非常重要
- 一般重要
- 有点重要

- 完全不重要

Q723. 您选择"... 避免让那些试图让我开心的人失望"
您认为该陈述有多重要？

- 极其重要
- 非常重要
- 一般重要
- 有点重要
- 完全不重要

Q724. 您选择"... 因为它很新潮"
您认为该陈述有多重要？

- 极其重要
- 非常重要
- 一般重要
- 有点重要
- 完全不重要

Q725. 您选择"... 因为它打折"
您认为该陈述有多重要？

- 极其重要
- 非常重要
- 一般重要
- 有点重要
- 完全不重要

Q726. 您选择"... 因为它让我保持身材"

您认为该陈述有多重要？

- 极其重要
- 非常重要
- 一般重要
- 有点重要
- 完全不重要

Q727. 您选择"... 因为这是唯一的选择"
您认为该陈述有多重要？

- 极其重要
- 非常重要
- 一般重要
- 有点重要
- 完全不重要

Q728. 您选择"... 因为我想每天都吃它"
您认为该陈述有多重要？

- 极其重要
- 非常重要
- 一般重要
- 有点重要
- 完全不重要

Q729. 请告诉我们您不吃红烧肉的原因（请选择所有适用的选项）

- ☐ 它不适用于特定场合
- ☐ 我绝不选它。 我喜欢每天吃一样的食物
- ☐ 我不认为它是一种零食

☐ 有其他人在的时候我不吃它
☐ 这个食物让我感到伤心、孤单或沮丧
☐ 它不是我在那个时刻想吃的东西
☐ 价格太高
☐ 我不经常吃它
☐ 我不想纵容自己
☐ 它的分量大小不合适
☐ 它不足以填饱肚子
☐ 它不方便
☐ 我不应该吃它
☐ 吃它显得我很土
☐ 它不是有机的
☐ 我不喜欢它
☐ 我不喜欢它看起来的样子
☐ 它卡路里太高
☐ 它不健康
☐ 我最近刚吃过， 我不想太经常吃同样的东西

Q730. 您选择”我不认为它是一种零食“
您认为该陈述有多重要？

○ 极其重要
○ 非常重要
○ 一般重要
○ 有点重要
○ 完全不重要

Q731. 您选择”我不喜欢它“
您认为该陈述有多重要？

- 极其重要
- 非常重要
- 一般重要
- 有点重要
- 完全不重要

Q732. 您选择"我不经常吃它"
您认为该陈述有多重要？

- 极其重要
- 非常重要
- 一般重要
- 有点重要
- 完全不重要

Q733. 您选择"它的分量大小不合适"
您认为该陈述有多重要？

- 极其重要
- 非常重要
- 一般重要
- 有点重要
- 完全不重要

Q734. 您选择"它不足以填饱肚子"
您认为该陈述有多重要？

- 极其重要
- 非常重要
- 一般重要

- 有点重要
- 完全不重要

Q735. 您选择"它不健康“
您认为该陈述有多重要？

- 极其重要
- 非常重要
- 一般重要
- 有点重要
- 完全不重要

Q736. 您选择"它不方便“
您认为该陈述有多重要？

- 极其重要
- 非常重要
- 一般重要
- 有点重要
- 完全不重要

Q737. 您选择"我不想纵容自己“
您认为该陈述有多重要？

- 极其重要
- 非常重要
- 一般重要
- 有点重要
- 完全不重要

Q738. 您选择”它不适用于特定场合“
您认为该陈述有多重要？

- 极其重要
- 非常重要
- 一般重要
- 有点重要
- 完全不重要

Q739. 您选择”有其他人在的时候我不吃它“
您认为该陈述有多重要？

- 极其重要
- 非常重要
- 一般重要
- 有点重要
- 完全不重要

Q740. 您选择”价格太高“
您认为该陈述有多重要？

- 极其重要
- 非常重要
- 一般重要
- 有点重要
- 完全不重要

Q741. 您选择”我不喜欢它看起来的样子“
您认为该陈述有多重要？

- 极其重要

- 非常重要
- 一般重要
- 有点重要
- 完全不重要

Q742. 您选择”它卡路里太高“
您认为该陈述有多重要？

- 极其重要
- 非常重要
- 一般重要
- 有点重要
- 完全不重要

Q743. 您选择”我不应该吃它“
您认为该陈述有多重要？

- 极其重要
- 非常重要
- 一般重要
- 有点重要
- 完全不重要

Q744. 您选择”吃它显得我很土“
您认为该陈述有多重要？

- 极其重要
- 非常重要
- 一般重要
- 有点重要

- 完全不重要

Q745. 您选择”它不是我在那个时刻想吃的东西“
您认为该陈述有多重要？

- 极其重要
- 非常重要
- 一般重要
- 有点重要
- 完全不重要

Q746. 您选择”我最近刚吃过， 我不想太经常吃同样的东西“
您认为该陈述有多重要？

- 极其重要
- 非常重要
- 一般重要
- 有点重要
- 完全不重要

Q747. 您选择”我绝不选它。 我喜欢每天吃一样的食物“
您认为该陈述有多重要？

- 极其重要
- 非常重要
- 一般重要
- 有点重要
- 完全不重要

Q748. 您选择”这个食物让我感到伤心、孤单或沮丧“

您认为该陈述有多重要？

- 极其重要
- 非常重要
- 一般重要
- 有点重要
- 完全不重要

Q749. 您选择"它不是有机的"
您认为该陈述有多重要？

- 极其重要
- 非常重要
- 一般重要
- 有点重要
- 完全不重要

Reasons for Dairy Food Choices(RATA)

Q750.
您吃豆浆吗?

- 是的， 我吃
- 不是， 我不吃

Q751.
试回想您上一次吃豆浆，并请回答以下问题。
您吃豆浆的原因有哪些（请选择所有适用的选项）

- ☐ ... 因为我很沮丧
- ☐ ... 这样我就可以和其他人共度时光

- ☐ ... 因为它不含有害物质（例如杀虫剂，污染物，抗生素）
- ☐ ... 因为它的味道很好
- ☐ ... 出于传统（例如家庭传统，特殊场合）
- ☐ ... 因为它很健康
- ☐ ... 因为它的价格不贵
- ☐ ... 因为我应该吃它
- ☐ ... 避免让那些试图让我开心的人失望
- ☐ ... 因为不吃它会不礼貌
- ☐ ... 因为它帮助我社交
- ☐ ... 因为它打折
- ☐ ... 因为它是天然的（例如没有经过基因改造）
- ☐ ... 因为我喜欢它
- ☐ ... 因为我从广告或在电视上看过它
- ☐ ... 因为它自然而然地吸引了我（吸引人的颜色）
- ☐ ... 因为我想每天都吃它
- ☐ ... 为了奖励自己
- ☐ ... 因为它适用于特定场合
- ☐ ... 因为我不想再花更多的钱
- ☐ ... 因为它让我保持身材
- ☐ ...因为我饿了
- ☐ ... 因为我经常吃它
- ☐ ... 因为我感到孤独
- ☐ ... 因为它很新潮
- ☐ ... 因为我关注我的体重
- ☐ ... 因为它准备起来很快
- ☐ ... 为了纵容自己
- ☐ ... 因为它很容易准备
- ☐ ... 因为我从小吃到大
- ☐ ...因为我习惯吃它
- ☐ ... 因为它的卡路里很低

- ☐ ... 保持均衡饮食
- ☐ ... 因为我需要能量
- ☐ ... 因为我熟悉它
- ☐ ... 因为其他人喜欢它
- ☐ ... 因为我对它有食欲
- ☐ ... 因为脂肪含量低
- ☐ ... 因为它最方便
- ☐ ... 因为它能令我感到愉悦的饱腹感
- ☐ ... 因为吃它让我很享受
- ☐ ... 因为我很伤心
- ☐ ... 因为它是有机的
- ☐ ... 因为这是唯一的选择
- ☐ ... 因为它使社交聚会更加舒适
- ☐ ... 因为它让我在别人面前看起来很好
- ☐ ... 因为它的呈现方式很有吸引力（例如包装）

Q752. 您选择"...因为我习惯吃它"
您认为该陈述有多重要？

- ○ 极其重要
- ○ 非常重要
- ○ 一般重要
- ○ 有点重要
- ○ 完全不重要

Q753. 您选择"... 因为它很健康"
您认为该陈述有多重要？

- ○ 极其重要
- ○ 非常重要

- 一般重要
- 有点重要
- 完全不重要

Q754. 您选择"... 因为它准备起来很快"
您认为该陈述有多重要？

- 极其重要
- 非常重要
- 一般重要
- 有点重要
- 完全不重要

Q755. 您选择"... 因为它的味道很好"
您认为该陈述有多重要？

- 极其重要
- 非常重要
- 一般重要
- 有点重要
- 完全不重要

Q756. 您选择"... 为了纵容自己"
您认为该陈述有多重要？

- 极其重要
- 非常重要
- 一般重要
- 有点重要
- 完全不重要

Q757. 您选择"... 因为它是天然的（例如没有经过基因改造）"
您认为该陈述有多重要？

- 极其重要
- 非常重要
- 一般重要
- 有点重要
- 完全不重要

Q758. 您选择"... 因为我不想再花更多的钱"
您认为该陈述有多重要？

- 极其重要
- 非常重要
- 一般重要
- 有点重要
- 完全不重要

Q759. 您选择"... 因为它的卡路里很低"
您认为该陈述有多重要？

- 极其重要
- 非常重要
- 一般重要
- 有点重要
- 完全不重要

Q760. 您选择"... 因为我很沮丧"
您认为该陈述有多重要？

- 极其重要
- 非常重要
- 一般重要
- 有点重要
- 完全不重要

Q761. 您选择"... 因为它让我在别人面前看起来很好"
您认为该陈述有多重要？

- 极其重要
- 非常重要
- 一般重要
- 有点重要
- 完全不重要

Q762. 您选择"... 因为不吃它会不礼貌"
您认为该陈述有多重要？

- 极其重要
- 非常重要
- 一般重要
- 有点重要
- 完全不重要

Q763. 您选择"... 因为它的呈现方式很有吸引力（例如包装）"
您认为该陈述有多重要？

- 极其重要
- 非常重要
- 一般重要

- 有点重要
- 完全不重要

Q764. 您选择"... 这样我就可以和其他人共度时光"
您认为该陈述有多重要？

- 极其重要
- 非常重要
- 一般重要
- 有点重要
- 完全不重要

Q765. 您选择"... 因为它是有机的"
您认为该陈述有多重要？

- 极其重要
- 非常重要
- 一般重要
- 有点重要
- 完全不重要

Q766. 您选择"... 因为我从小吃到大"
您认为该陈述有多重要？

- 极其重要
- 非常重要
- 一般重要
- 有点重要
- 完全不重要

Q767. 您选择"... 因为吃它让我很享受"
您认为该陈述有多重要？

- 极其重要
- 非常重要
- 一般重要
- 有点重要
- 完全不重要

Q768. 您选择"...因为我饿了"
您认为该陈述有多重要？

- 极其重要
- 非常重要
- 一般重要
- 有点重要
- 完全不重要

Q769. 您选择"... 因为我经常吃它"
您认为该陈述有多重要？

- 极其重要
- 非常重要
- 一般重要
- 有点重要
- 完全不重要

Q770. 您选择"... 因为我对它有食欲"
您认为该陈述有多重要？

- 极其重要

- 非常重要
- 一般重要
- 有点重要
- 完全不重要

Q771. 您选择"... 因为它很容易准备"
您认为该陈述有多重要？

- 极其重要
- 非常重要
- 一般重要
- 有点重要
- 完全不重要

Q772. 您选择"... 因为我很伤心"
您认为该陈述有多重要？

- 极其重要
- 非常重要
- 一般重要
- 有点重要
- 完全不重要

Q773. 您选择"... 因为脂肪含量低"
您认为该陈述有多重要？

- 极其重要
- 非常重要
- 一般重要
- 有点重要

- 完全不重要

Q774. 您选择"... 因为我从广告或在电视上看过它"
您认为该陈述有多重要？

- 极其重要
- 非常重要
- 一般重要
- 有点重要
- 完全不重要

Q775. 您选择"... 因为它使社交聚会更加舒适"
您认为该陈述有多重要？

- 极其重要
- 非常重要
- 一般重要
- 有点重要
- 完全不重要

Q776. 您选择"... 因为我应该吃它"
您认为该陈述有多重要？

- 极其重要
- 非常重要
- 一般重要
- 有点重要
- 完全不重要

Q777. 您选择"... 因为其他人喜欢它"

您认为该陈述有多重要？

- 极其重要
- 非常重要
- 一般重要
- 有点重要
- 完全不重要

Q778. 您选择"... 因为我感到孤独"
您认为该陈述有多重要？

- 极其重要
- 非常重要
- 一般重要
- 有点重要
- 完全不重要

Q779. 您选择"... 因为我关注我的体重"
您认为该陈述有多重要？

- 极其重要
- 非常重要
- 一般重要
- 有点重要
- 完全不重要

Q780. 您选择"... 因为它不含有害物质（例如杀虫剂，污染物，抗生素）"
您认为该陈述有多重要？

- 极其重要
- 非常重要

- 一般重要
- 有点重要
- 完全不重要

Q781. 您选择"... 因为它最方便"
您认为该陈述有多重要？

- 极其重要
- 非常重要
- 一般重要
- 有点重要
- 完全不重要

Q782. 您选择"... 因为我需要能量"
您认为该陈述有多重要？

- 极其重要
- 非常重要
- 一般重要
- 有点重要
- 完全不重要

Q783. 您选择"... 因为我熟悉它"
您认为该陈述有多重要？

- 极其重要
- 非常重要
- 一般重要
- 有点重要
- 完全不重要

Q784. 您选择"... 因为我喜欢它"
您认为该陈述有多重要？

- 极其重要
- 非常重要
- 一般重要
- 有点重要
- 完全不重要

Q785. 您选择"... 因为它能令我感到愉悦的饱腹感"
您认为该陈述有多重要？

- 极其重要
- 非常重要
- 一般重要
- 有点重要
- 完全不重要

Q786. 您选择"... 为了奖励自己"
您认为该陈述有多重要？

- 极其重要
- 非常重要
- 一般重要
- 有点重要
- 完全不重要

Q787. 您选择"... 出于传统（例如家庭传统，特殊场合）"
您认为该陈述有多重要？

- 极其重要
- 非常重要
- 一般重要
- 有点重要
- 完全不重要

Q788. 您选择"... 因为它适用于特定场合"
您认为该陈述有多重要？

- 极其重要
- 非常重要
- 一般重要
- 有点重要
- 完全不重要

Q789. 您选择"... 保持均衡饮食"
您认为该陈述有多重要？

- 极其重要
- 非常重要
- 一般重要
- 有点重要
- 完全不重要

Q790. 您选择"... 因为它帮助我社交"
您认为该陈述有多重要？

- 极其重要
- 非常重要
- 一般重要

- 有点重要
- 完全不重要

Q791. 您选择"... 因为它的价格不贵"
您认为该陈述有多重要？

- 极其重要
- 非常重要
- 一般重要
- 有点重要
- 完全不重要

Q792. 您选择"... 因为它自然而然地吸引了我（吸引人的颜色）"
您认为该陈述有多重要？

- 极其重要
- 非常重要
- 一般重要
- 有点重要
- 完全不重要

Q793. 您选择"... 避免让那些试图让我开心的人失望"
您认为该陈述有多重要？

- 极其重要
- 非常重要
- 一般重要
- 有点重要
- 完全不重要

Q794. 您选择"... 因为它很新潮"
您认为该陈述有多重要？

- 极其重要
- 非常重要
- 一般重要
- 有点重要
- 完全不重要

Q795. 您选择"... 因为它打折"
您认为该陈述有多重要？

- 极其重要
- 非常重要
- 一般重要
- 有点重要
- 完全不重要

Q796. 您选择"... 因为它让我保持身材"
您认为该陈述有多重要？

- 极其重要
- 非常重要
- 一般重要
- 有点重要
- 完全不重要

Q797. 您选择"... 因为这是唯一的选择"
您认为该陈述有多重要？

- 极其重要

- 非常重要
- 一般重要
- 有点重要
- 完全不重要

Q798. 您选择"... 因为我想每天都吃它"
您认为该陈述有多重要？

- 极其重要
- 非常重要
- 一般重要
- 有点重要
- 完全不重要

Q799. 请告诉我们您不吃豆浆的原因（请选择所有适用的选项）

- 吃它显得我很土
- 我不喜欢它看起来的样子
- 它不是有机的
- 它卡路里太高
- 我不经常吃它
- 它不适用于特定场合
- 有其他人在的时候我不吃它
- 我绝不选它。 我喜欢每天吃一样的食物
- 这个食物让我感到伤心、孤单或沮丧
- 价格太高
- 我不认为它是一种零食
- 它不方便
- 它不健康
- 它的分量大小不合适

- ☐ 我不喜欢它
- ☐ 我最近刚吃过， 我不想太经常吃同样的东西
- ☐ 它不是我在那个时刻想吃的东西
- ☐ 我不想纵容自己
- ☐ 它不足以填饱肚子
- ☐ 我不应该吃它

Q800. 您选择”我不认为它是一种零食“
您认为该陈述有多重要？

- ○ 极其重要
- ○ 非常重要
- ○ 一般重要
- ○ 有点重要
- ○ 完全不重要

Q801. 您选择”我不喜欢它“
您认为该陈述有多重要？

- ○ 极其重要
- ○ 非常重要
- ○ 一般重要
- ○ 有点重要
- ○ 完全不重要

Q802. 您选择”我不经常吃它“
您认为该陈述有多重要？

- ○ 极其重要
- ○ 非常重要

○ 一般重要
○ 有点重要
○ 完全不重要

Q803. 您选择”它的分量大小不合适“
您认为该陈述有多重要？

○ 极其重要
○ 非常重要
○ 一般重要
○ 有点重要
○ 完全不重要

Q804. 您选择”它不足以填饱肚子“
您认为该陈述有多重要？

○ 极其重要
○ 非常重要
○ 一般重要
○ 有点重要
○ 完全不重要

Q805. 您选择”它不健康“
您认为该陈述有多重要？

○ 极其重要
○ 非常重要
○ 一般重要
○ 有点重要
○ 完全不重要

Q806. 您选择”它不方便“
您认为该陈述有多重要？

- 极其重要
- 非常重要
- 一般重要
- 有点重要
- 完全不重要

Q807. 您选择”我不想纵容自己“
您认为该陈述有多重要？

- 极其重要
- 非常重要
- 一般重要
- 有点重要
- 完全不重要

Q808. 您选择”它不适用于特定场合“
您认为该陈述有多重要？

- 极其重要
- 非常重要
- 一般重要
- 有点重要
- 完全不重要

Q809. 您选择”有其他人在的时候我不吃它“
您认为该陈述有多重要？

- 极其重要
- 非常重要
- 一般重要
- 有点重要
- 完全不重要

Q810. 您选择"价格太高"
您认为该陈述有多重要？

- 极其重要
- 非常重要
- 一般重要
- 有点重要
- 完全不重要

Q811. 您选择"我不喜欢它看起来的样子"
您认为该陈述有多重要？

- 极其重要
- 非常重要
- 一般重要
- 有点重要
- 完全不重要

Q812. 您选择"它卡路里太高"
您认为该陈述有多重要？

- 极其重要
- 非常重要
- 一般重要

- 有点重要
- 完全不重要

Q813. 您选择"我不应该吃它"
您认为该陈述有多重要？

- 极其重要
- 非常重要
- 一般重要
- 有点重要
- 完全不重要

Q814. 您选择"吃它显得我很土"
您认为该陈述有多重要？

- 极其重要
- 非常重要
- 一般重要
- 有点重要
- 完全不重要

Q815. 您选择"它不是我在那个时刻想吃的东西"
您认为该陈述有多重要？

- 极其重要
- 非常重要
- 一般重要
- 有点重要
- 完全不重要

Q816. 您选择”我最近刚吃过， 我不想太经常吃同样的东西“
您认为该陈述有多重要？

- 极其重要
- 非常重要
- 一般重要
- 有点重要
- 完全不重要

Q817. 您选择”我绝不选它。 我喜欢每天吃一样的食物“
您认为该陈述有多重要？

- 极其重要
- 非常重要
- 一般重要
- 有点重要
- 完全不重要

Q818. 您选择”这个食物让我感到伤心、孤单或沮丧“
您认为该陈述有多重要？

- 极其重要
- 非常重要
- 一般重要
- 有点重要
- 完全不重要

Q819. 您选择”它不是有机的“
您认为该陈述有多重要？

- 极其重要

- 非常重要
- 一般重要
- 有点重要
- 完全不重要

Reasons for Fruit Choices(RATA)

Q820.
您吃香蕉吗?

- 是的， 我吃
- 不是， 我不吃

Q821.
试回想您上一次吃香蕉，并请回答以下问题。
您吃香蕉的原因有哪些（请选择所有适用的选项）

- ... 因为我想每天都吃它
- ... 因为我经常吃它
- ... 保持均衡饮食
- ... 为了奖励自己
- ... 因为我应该吃它
- ... 因为我需要能量
- ... 因为它准备起来很快
- ... 因为不吃它会不礼貌
- ... 因为它的价格不贵
- ... 因为它让我在别人面前看起来很好
- ... 因为脂肪含量低
- ...因为我饿了
- ... 因为我很沮丧

- ☐ ... 因为吃它让我很享受
- ☐ ... 因为我很伤心
- ☐ ... 避免让那些试图让我开心的人失望
- ☐ ... 因为它能令我感到愉悦的饱腹感
- ☐ ... 因为我从广告或在电视上看过它
- ☐ ... 因为它自然而然地吸引了我（吸引人的颜色）
- ☐ ... 出于传统（例如家庭传统，特殊场合）
- ☐ ...因为我习惯吃它
- ☐ ... 这样我就可以和其他人共度时光
- ☐ ... 因为它的卡路里很低
- ☐ ... 因为我喜欢它
- ☐ ... 为了纵容自己
- ☐ ... 因为我熟悉它
- ☐ ... 因为我感到孤独
- ☐ ... 因为它很健康
- ☐ ... 因为我对它有食欲
- ☐ ... 因为它让我保持身材
- ☐ ... 因为它很新潮
- ☐ ... 因为它帮助我社交
- ☐ ... 因为这是唯一的选择
- ☐ ... 因为我不想再花更多的钱
- ☐ ... 因为它很容易准备
- ☐ ... 因为它适用于特定场合
- ☐ ... 因为它使社交聚会更加舒适
- ☐ ... 因为它的味道很好
- ☐ ... 因为其他人喜欢它
- ☐ ... 因为它最方便
- ☐ ... 因为它是天然的（例如没有经过基因改造）
- ☐ ... 因为它打折
- ☐ ... 因为它的呈现方式很有吸引力（例如包装）

- ☐ ... 因为我从小吃到大
- ☐ ... 因为它不含有害物质（例如杀虫剂，污染物，抗生素）
- ☐ ... 因为我关注我的体重
- ☐ ... 因为它是有机的

Q822. 您选择"...因为我习惯吃它"
您认为该陈述有多重要？

- ○ 极其重要
- ○ 非常重要
- ○ 一般重要
- ○ 有点重要
- ○ 完全不重要

Q823. 您选择"... 因为它很健康"
您认为该陈述有多重要？

- ○ 极其重要
- ○ 非常重要
- ○ 一般重要
- ○ 有点重要
- ○ 完全不重要

Q824. 您选择"... 因为它准备起来很快"
您认为该陈述有多重要？

- ○ 极其重要
- ○ 非常重要
- ○ 一般重要
- ○ 有点重要

完全不重要

Q825. 您选择"... 因为它的味道很好"
您认为该陈述有多重要？

极其重要
非常重要
一般重要
有点重要
完全不重要

Q826. 您选择"... 为了纵容自己"
您认为该陈述有多重要？

极其重要
非常重要
一般重要
有点重要
完全不重要

Q827. 您选择"... 因为它是天然的（例如没有经过基因改造）"
您认为该陈述有多重要？

极其重要
非常重要
一般重要
有点重要
完全不重要

Q828. 您选择"... 因为我不想再花更多的钱"

您认为该陈述有多重要？

- 极其重要
- 非常重要
- 一般重要
- 有点重要
- 完全不重要

Q829. 您选择"... 因为它的卡路里很低"
您认为该陈述有多重要？

- 极其重要
- 非常重要
- 一般重要
- 有点重要
- 完全不重要

Q830. 您选择"... 因为我很沮丧"
您认为该陈述有多重要？

- 极其重要
- 非常重要
- 一般重要
- 有点重要
- 完全不重要

Q831. 您选择"... 因为它让我在别人面前看起来很好"
您认为该陈述有多重要？

- 极其重要
- 非常重要

- 一般重要
- 有点重要
- 完全不重要

Q832. 您选择"... 因为不吃它会不礼貌"
您认为该陈述有多重要？

- 极其重要
- 非常重要
- 一般重要
- 有点重要
- 完全不重要

Q833. 您选择"... 因为它的呈现方式很有吸引力（例如包装）"
您认为该陈述有多重要？

- 极其重要
- 非常重要
- 一般重要
- 有点重要
- 完全不重要

Q834. 您选择"... 这样我就可以和其他人共度时光"
您认为该陈述有多重要？

- 极其重要
- 非常重要
- 一般重要
- 有点重要
- 完全不重要

Q835. 您选择"... 因为它是有机的"
您认为该陈述有多重要？

- 极其重要
- 非常重要
- 一般重要
- 有点重要
- 完全不重要

Q836. 您选择"... 因为我从小吃到大"
您认为该陈述有多重要？

- 极其重要
- 非常重要
- 一般重要
- 有点重要
- 完全不重要

Q837. 您选择"... 因为吃它让我很享受"
您认为该陈述有多重要？

- 极其重要
- 非常重要
- 一般重要
- 有点重要
- 完全不重要

Q838. 您选择"...因为我饿了"
您认为该陈述有多重要？

- 极其重要
- 非常重要
- 一般重要
- 有点重要
- 完全不重要

Q839. 您选择"... 因为我经常吃它"
您认为该陈述有多重要？

- 极其重要
- 非常重要
- 一般重要
- 有点重要
- 完全不重要

Q840. 您选择"... 因为我对它有食欲"
您认为该陈述有多重要？

- 极其重要
- 非常重要
- 一般重要
- 有点重要
- 完全不重要

Q841. 您选择"... 因为它很容易准备"
您认为该陈述有多重要？

- 极其重要
- 非常重要
- 一般重要

- 有点重要
- 完全不重要

Q842. 您选择"... 因为我很伤心"
您认为该陈述有多重要？

- 极其重要
- 非常重要
- 一般重要
- 有点重要
- 完全不重要

Q843. 您选择"... 因为脂肪含量低"
您认为该陈述有多重要？

- 极其重要
- 非常重要
- 一般重要
- 有点重要
- 完全不重要

Q844. 您选择"... 因为我从广告或在电视上看过它"
您认为该陈述有多重要？

- 极其重要
- 非常重要
- 一般重要
- 有点重要
- 完全不重要

Q845. 您选择"... 因为它使社交聚会更加舒适"
您认为该陈述有多重要？

- 极其重要
- 非常重要
- 一般重要
- 有点重要
- 完全不重要

Q846. 您选择"... 因为我应该吃它"
您认为该陈述有多重要？

- 极其重要
- 非常重要
- 一般重要
- 有点重要
- 完全不重要

Q847. 您选择"... 因为其他人喜欢它"
您认为该陈述有多重要？

- 极其重要
- 非常重要
- 一般重要
- 有点重要
- 完全不重要

Q848. 您选择"... 因为我感到孤独"
您认为该陈述有多重要？

- 极其重要

- 非常重要
- 一般重要
- 有点重要
- 完全不重要

Q849. 您选择"... 因为我关注我的体重"
您认为该陈述有多重要？

- 极其重要
- 非常重要
- 一般重要
- 有点重要
- 完全不重要

Q850. 您选择"... 因为它不含有害物质（例如杀虫剂，污染物，抗生素）"
您认为该陈述有多重要？

- 极其重要
- 非常重要
- 一般重要
- 有点重要
- 完全不重要

Q851. 您选择"... 因为它最方便"
您认为该陈述有多重要？

- 极其重要
- 非常重要
- 一般重要
- 有点重要

○ 完全不重要

Q852. 您选择"... 因为我需要能量"
您认为该陈述有多重要？

○ 极其重要
○ 非常重要
○ 一般重要
○ 有点重要
○ 完全不重要

Q853. 您选择"... 因为我熟悉它"
您认为该陈述有多重要？

○ 极其重要
○ 非常重要
○ 一般重要
○ 有点重要
○ 完全不重要

Q854. 您选择"... 因为我喜欢它"
您认为该陈述有多重要？

○ 极其重要
○ 非常重要
○ 一般重要
○ 有点重要
○ 完全不重要

Q855. 您选择"... 因为它能令我感到愉悦的饱腹感"

您认为该陈述有多重要？

- 极其重要
- 非常重要
- 一般重要
- 有点重要
- 完全不重要

Q856. 您选择"... 为了奖励自己"
您认为该陈述有多重要？

- 极其重要
- 非常重要
- 一般重要
- 有点重要
- 完全不重要

Q857. 您选择"... 出于传统（例如家庭传统，特殊场合）"
您认为该陈述有多重要？

- 极其重要
- 非常重要
- 一般重要
- 有点重要
- 完全不重要

Q858. 您选择"... 因为它适用于特定场合"
您认为该陈述有多重要？

- 极其重要
- 非常重要

- 一般重要
- 有点重要
- 完全不重要

Q859. 您选择"... 保持均衡饮食"
您认为该陈述有多重要？

- 极其重要
- 非常重要
- 一般重要
- 有点重要
- 完全不重要

Q860. 您选择"... 因为它帮助我社交"
您认为该陈述有多重要？

- 极其重要
- 非常重要
- 一般重要
- 有点重要
- 完全不重要

Q861. 您选择"... 因为它的价格不贵"
您认为该陈述有多重要？

- 极其重要
- 非常重要
- 一般重要
- 有点重要
- 完全不重要

Q862. 您选择"... 因为它自然而然地吸引了我（吸引人的颜色）"
您认为该陈述有多重要？

- 极其重要
- 非常重要
- 一般重要
- 有点重要
- 完全不重要

Q863. 您选择"... 避免让那些试图让我开心的人失望"
您认为该陈述有多重要？

- 极其重要
- 非常重要
- 一般重要
- 有点重要
- 完全不重要

Q864. 您选择"... 因为它很新潮"
您认为该陈述有多重要？

- 极其重要
- 非常重要
- 一般重要
- 有点重要
- 完全不重要

Q865. 您选择"... 因为它打折"
您认为该陈述有多重要？

- 极其重要
- 非常重要
- 一般重要
- 有点重要
- 完全不重要

Q866. 您选择"... 因为它让我保持身材"
您认为该陈述有多重要？

- 极其重要
- 非常重要
- 一般重要
- 有点重要
- 完全不重要

Q867. 您选择"... 因为这是唯一的选择"
您认为该陈述有多重要？

- 极其重要
- 非常重要
- 一般重要
- 有点重要
- 完全不重要

Q868. 您选择"... 因为我想每天都吃它"
您认为该陈述有多重要？

- 极其重要
- 非常重要
- 一般重要

- 有点重要
- 完全不重要

Q869. 请告诉我们您不吃香蕉的原因（请选择所有适用的选项）

- 我不经常吃它
- 我不喜欢它看起来的样子
- 我不认为它是一种零食
- 我绝不选它。 我喜欢每天吃一样的食物
- 它不方便
- 它不足以填饱肚子
- 吃它显得我很土
- 它不健康
- 我不想纵容自己
- 我不应该吃它
- 它卡路里太高
- 它不是我在那个时刻想吃的东西
- 有其他人在的时候我不吃它
- 它不适用于特定场合
- 这个食物让我感到伤心、孤单或沮丧
- 价格太高
- 它不是有机的
- 我不喜欢它
- 我最近刚吃过， 我不想太经常吃同样的东西
- 它的分量大小不合适

Q870. 您选择”我不认为它是一种零食“
您认为该陈述有多重要？

- 极其重要

- 非常重要
- 一般重要
- 有点重要
- 完全不重要

Q871. 您选择”我不喜欢它“
您认为该陈述有多重要？

- 极其重要
- 非常重要
- 一般重要
- 有点重要
- 完全不重要

Q872. 您选择”我不经常吃它“
您认为该陈述有多重要？

- 极其重要
- 非常重要
- 一般重要
- 有点重要
- 完全不重要

Q873. 您选择”它的分量大小不合适“
您认为该陈述有多重要？

- 极其重要
- 非常重要
- 一般重要
- 有点重要

○ 完全不重要

Q874. 您选择"它不足以填饱肚子"
您认为该陈述有多重要？

○ 极其重要
○ 非常重要
○ 一般重要
○ 有点重要
○ 完全不重要

Q875. 您选择"它不健康"
您认为该陈述有多重要？

○ 极其重要
○ 非常重要
○ 一般重要
○ 有点重要
○ 完全不重要

Q876. 您选择"它不方便"
您认为该陈述有多重要？

○ 极其重要
○ 非常重要
○ 一般重要
○ 有点重要
○ 完全不重要

Q877. 您选择"我不想纵容自己"

您认为该陈述有多重要？

- 极其重要
- 非常重要
- 一般重要
- 有点重要
- 完全不重要

Q878. 您选择”它不适用于特定场合“
您认为该陈述有多重要？

- 极其重要
- 非常重要
- 一般重要
- 有点重要
- 完全不重要

Q879. 您选择”有其他人在的时候我不吃它“
您认为该陈述有多重要？

- 极其重要
- 非常重要
- 一般重要
- 有点重要
- 完全不重要

Q880. 您选择”价格太高“
您认为该陈述有多重要？

- 极其重要
- 非常重要

- 一般重要
- 有点重要
- 完全不重要

Q881. 您选择"我不喜欢它看起来的样子"
您认为该陈述有多重要？

- 极其重要
- 非常重要
- 一般重要
- 有点重要
- 完全不重要

Q882. 您选择"它卡路里太高"
您认为该陈述有多重要？

- 极其重要
- 非常重要
- 一般重要
- 有点重要
- 完全不重要

Q883. 您选择"我不应该吃它"
您认为该陈述有多重要？

- 极其重要
- 非常重要
- 一般重要
- 有点重要
- 完全不重要

Q884. 您选择”吃它显得我很土“
您认为该陈述有多重要？

- 极其重要
- 非常重要
- 一般重要
- 有点重要
- 完全不重要

Q885. 您选择”它不是我在那个时刻想吃的东西“
您认为该陈述有多重要？

- 极其重要
- 非常重要
- 一般重要
- 有点重要
- 完全不重要

Q886. 您选择”我最近刚吃过， 我不想太经常吃同样的东西“
您认为该陈述有多重要？

- 极其重要
- 非常重要
- 一般重要
- 有点重要
- 完全不重要

Q887. 您选择”我绝不选它。 我喜欢每天吃一样的食物“
您认为该陈述有多重要？

- 极其重要
- 非常重要
- 一般重要
- 有点重要
- 完全不重要

Q888. 您选择”这个食物让我感到伤心、孤单或沮丧“
您认为该陈述有多重要？

- 极其重要
- 非常重要
- 一般重要
- 有点重要
- 完全不重要

Q889. 您选择”它不是有机的“
您认为该陈述有多重要？

- 极其重要
- 非常重要
- 一般重要
- 有点重要
- 完全不重要

Reasons for Sweet food Choices(RATA)

Q890.
您吃麻球吗?

- 是的，我吃

- 不是， 我不吃

Q891.

试回想您上一次吃麻球，并请回答以下问题。

您吃麻球的原因有哪些（请选择所有适用的选项）

- ☐ ... 因为它很容易准备
- ☐ ... 因为它很新潮
- ☐ ... 因为吃它让我很享受
- ☐ ... 因为我想每天都吃它
- ☐ ... 因为它是天然的（例如没有经过基因改造）
- ☐ ... 因为我从小吃到大
- ☐ ... 因为它的价格不贵
- ☐ ... 因为它使社交聚会更加舒适
- ☐ ... 因为我关注我的体重
- ☐ ... 因为它让我保持身材
- ☐ ... 因为我对它有食欲
- ☐ ... 因为它适用于特定场合
- ☐ ... 因为我从广告或在电视上看过它
- ☐ ... 因为它让我在别人面前看起来很好
- ☐ ... 因为它是有机的
- ☐ ...因为我习惯吃它
- ☐ ... 因为脂肪含量低
- ☐ ... 出于传统（例如家庭传统，特殊场合）
- ☐ ... 为了纵容自己
- ☐ ... 因为它的呈现方式很有吸引力（例如包装）
- ☐ ... 因为其他人喜欢它
- ☐ ... 这样我就可以和其他人共度时光
- ☐ ... 因为它打折
- ☐ ... 因为我熟悉它

- ☐ ... 因为它的卡路里很低
- ☐ ... 因为我很伤心
- ☐ ... 因为我不想再花更多的钱
- ☐ ... 因为我应该吃它
- ☐ ... 因为我喜欢它
- ☐ ... 因为它能令我感到愉悦的饱腹感
- ☐ ... 因为我感到孤独
- ☐ ... 因为它准备起来很快
- ☐ ... 因为它帮助我社交
- ☐ ... 避免让那些试图让我开心的人失望
- ☐ ... 因为我很沮丧
- ☐ ... 因为我经常吃它
- ☐ ... 因为不吃它会不礼貌
- ☐ ... 因为它很健康
- ☐ ... 因为它不含有害物质（例如杀虫剂，污染物，抗生素）
- ☐ ... 因为它最方便
- ☐ ... 保持均衡饮食
- ☐ ... 因为它自然而然地吸引了我（吸引人的颜色）
- ☐ ... 因为它的味道很好
- ☐ ... 因为我需要能量
- ☐ ... 为了奖励自己
- ☐ ... 因为这是唯一的选择
- ☐ ...因为我饿了

Q892. 您选择"...因为我习惯吃它"
您认为该陈述有多重要？

- ○ 极其重要
- ○ 非常重要
- ○ 一般重要

- 有点重要
- 完全不重要

Q893. 您选择"... 因为它很健康"
您认为该陈述有多重要？

- 极其重要
- 非常重要
- 一般重要
- 有点重要
- 完全不重要

Q894. 您选择"... 因为它准备起来很快"
您认为该陈述有多重要？

- 极其重要
- 非常重要
- 一般重要
- 有点重要
- 完全不重要

Q895. 您选择"... 因为它的味道很好"
您认为该陈述有多重要？

- 极其重要
- 非常重要
- 一般重要
- 有点重要
- 完全不重要

Q896. 您选择"... 为了纵容自己"
您认为该陈述有多重要？

- 极其重要
- 非常重要
- 一般重要
- 有点重要
- 完全不重要

Q897. 您选择"... 因为它是天然的（例如没有经过基因改造）"
您认为该陈述有多重要？

- 极其重要
- 非常重要
- 一般重要
- 有点重要
- 完全不重要

Q898. 您选择"... 因为我不想再花更多的钱"
您认为该陈述有多重要？

- 极其重要
- 非常重要
- 一般重要
- 有点重要
- 完全不重要

Q899. 您选择"... 因为它的卡路里很低"
您认为该陈述有多重要？

- 极其重要

- 非常重要
- 一般重要
- 有点重要
- 完全不重要

Q900. 您选择"... 因为我很沮丧"
您认为该陈述有多重要？

- 极其重要
- 非常重要
- 一般重要
- 有点重要
- 完全不重要

Q901. 您选择"... 因为它让我在别人面前看起来很好"
您认为该陈述有多重要？

- 极其重要
- 非常重要
- 一般重要
- 有点重要
- 完全不重要

Q902. 您选择"... 因为不吃它会不礼貌"
您认为该陈述有多重要？

- 极其重要
- 非常重要
- 一般重要
- 有点重要

- 完全不重要

Q903. 您选择"... 因为它的呈现方式很有吸引力（例如包装）"
您认为该陈述有多重要？

- 极其重要
- 非常重要
- 一般重要
- 有点重要
- 完全不重要

Q904. 您选择"... 这样我就可以和其他人共度时光"
您认为该陈述有多重要？

- 极其重要
- 非常重要
- 一般重要
- 有点重要
- 完全不重要

Q905. 您选择"... 因为它是有机的"
您认为该陈述有多重要？

- 极其重要
- 非常重要
- 一般重要
- 有点重要
- 完全不重要

Q906. 您选择"... 因为我从小吃到大"

您认为该陈述有多重要？

- 极其重要
- 非常重要
- 一般重要
- 有点重要
- 完全不重要

Q907. 您选择"... 因为吃它让我很享受"
您认为该陈述有多重要？

- 极其重要
- 非常重要
- 一般重要
- 有点重要
- 完全不重要

Q908. 您选择"...因为我饿了"
您认为该陈述有多重要？

- 极其重要
- 非常重要
- 一般重要
- 有点重要
- 完全不重要

Q909. 您选择"... 因为我经常吃它"
您认为该陈述有多重要？

- 极其重要
- 非常重要

- 一般重要
- 有点重要
- 完全不重要

Q910. 您选择"... 因为我对它有食欲"
您认为该陈述有多重要？

- 极其重要
- 非常重要
- 一般重要
- 有点重要
- 完全不重要

Q911. 您选择"... 因为它很容易准备"
您认为该陈述有多重要？

- 极其重要
- 非常重要
- 一般重要
- 有点重要
- 完全不重要

Q912. 您选择"... 因为我很伤心"
您认为该陈述有多重要？

- 极其重要
- 非常重要
- 一般重要
- 有点重要
- 完全不重要

Q913. 您选择"... 因为脂肪含量低"
您认为该陈述有多重要？

- 极其重要
- 非常重要
- 一般重要
- 有点重要
- 完全不重要

Q914. 您选择"... 因为我从广告或在电视上看过它"
您认为该陈述有多重要？

- 极其重要
- 非常重要
- 一般重要
- 有点重要
- 完全不重要

Q915. 您选择"... 因为它使社交聚会更加舒适"
您认为该陈述有多重要？

- 极其重要
- 非常重要
- 一般重要
- 有点重要
- 完全不重要

Q916. 您选择"... 因为我应该吃它"
您认为该陈述有多重要？

- 极其重要
- 非常重要
- 一般重要
- 有点重要
- 完全不重要

Q917. 您选择"... 因为其他人喜欢它"
您认为该陈述有多重要？

- 极其重要
- 非常重要
- 一般重要
- 有点重要
- 完全不重要

Q918. 您选择"... 因为我感到孤独"
您认为该陈述有多重要？

- 极其重要
- 非常重要
- 一般重要
- 有点重要
- 完全不重要

Q919. 您选择"... 因为我关注我的体重"
您认为该陈述有多重要？

- 极其重要
- 非常重要
- 一般重要

- 有点重要
- 完全不重要

Q920. 您选择"... 因为它不含有害物质（例如杀虫剂，污染物，抗生素）"
您认为该陈述有多重要？

- 极其重要
- 非常重要
- 一般重要
- 有点重要
- 完全不重要

Q921. 您选择"... 因为它最方便"
您认为该陈述有多重要？

- 极其重要
- 非常重要
- 一般重要
- 有点重要
- 完全不重要

Q922. 您选择"... 因为我需要能量"
您认为该陈述有多重要？

- 极其重要
- 非常重要
- 一般重要
- 有点重要
- 完全不重要

Q923. 您选择"... 因为我熟悉它"
您认为该陈述有多重要？

- 极其重要
- 非常重要
- 一般重要
- 有点重要
- 完全不重要

Q924. 您选择"... 因为我喜欢它"
您认为该陈述有多重要？

- 极其重要
- 非常重要
- 一般重要
- 有点重要
- 完全不重要

Q925. 您选择"... 因为它能令我感到愉悦的饱腹感"
您认为该陈述有多重要？

- 极其重要
- 非常重要
- 一般重要
- 有点重要
- 完全不重要

Q926. 您选择"... 为了奖励自己"
您认为该陈述有多重要？

- 极其重要

- 非常重要
- 一般重要
- 有点重要
- 完全不重要

Q927. 您选择"... 出于传统（例如家庭传统，特殊场合）"
您认为该陈述有多重要？

- 极其重要
- 非常重要
- 一般重要
- 有点重要
- 完全不重要

Q928. 您选择"... 因为它适用于特定场合"
您认为该陈述有多重要？

- 极其重要
- 非常重要
- 一般重要
- 有点重要
- 完全不重要

Q929. 您选择"... 保持均衡饮食"
您认为该陈述有多重要？

- 极其重要
- 非常重要
- 一般重要
- 有点重要

◯ 完全不重要

Q930. 您选择"... 因为它帮助我社交"
您认为该陈述有多重要？

◯ 极其重要
◯ 非常重要
◯ 一般重要
◯ 有点重要
◯ 完全不重要

Q931. 您选择"... 因为它的价格不贵"
您认为该陈述有多重要？

◯ 极其重要
◯ 非常重要
◯ 一般重要
◯ 有点重要
◯ 完全不重要

Q932. 您选择"... 因为它自然而然地吸引了我（吸引人的颜色）"
您认为该陈述有多重要？

◯ 极其重要
◯ 非常重要
◯ 一般重要
◯ 有点重要
◯ 完全不重要

Q933. 您选择"... 避免让那些试图让我开心的人失望"

您认为该陈述有多重要？

- 极其重要
- 非常重要
- 一般重要
- 有点重要
- 完全不重要

Q934. 您选择"... 因为它很新潮"
您认为该陈述有多重要？

- 极其重要
- 非常重要
- 一般重要
- 有点重要
- 完全不重要

Q935. 您选择"... 因为它打折"
您认为该陈述有多重要？

- 极其重要
- 非常重要
- 一般重要
- 有点重要
- 完全不重要

Q936. 您选择"... 因为它让我保持身材"
您认为该陈述有多重要？

- 极其重要
- 非常重要

- 一般重要
- 有点重要
- 完全不重要

Q937. 您选择"... 因为这是唯一的选择"
您认为该陈述有多重要？

- 极其重要
- 非常重要
- 一般重要
- 有点重要
- 完全不重要

Q938. 您选择"... 因为我想每天都吃它"
您认为该陈述有多重要？

- 极其重要
- 非常重要
- 一般重要
- 有点重要
- 完全不重要

Q939. 请告诉我们您不吃麻球的原因（请选择所有适用的选项）

- 它不是我在那个时刻想吃的东西
- 它不适用于特定场合
- 它不足以填饱肚子
- 我绝不选它。 我喜欢每天吃一样的食物
- 它不健康
- 这个食物让我感到伤心、孤单或沮丧

- ☐ 我不喜欢它看起来的样子
- ☐ 吃它显得我很土
- ☐ 我不应该吃它
- ☐ 我不想纵容自己
- ☐ 它不是有机的
- ☐ 价格太高
- ☐ 我不经常吃它
- ☐ 有其他人在的时候我不吃它
- ☐ 它卡路里太高
- ☐ 我不认为它是一种零食
- ☐ 它不方便
- ☐ 它的分量大小不合适
- ☐ 我最近刚吃过， 我不想太经常吃同样的东西
- ☐ 我不喜欢它

Q940. 您选择”我不认为它是一种零食“
您认为该陈述有多重要？

- ○ 极其重要
- ○ 非常重要
- ○ 一般重要
- ○ 有点重要
- ○ 完全不重要

Q941. 您选择”我不喜欢它“
您认为该陈述有多重要？

- ○ 极其重要
- ○ 非常重要
- ○ 一般重要

- 有点重要
- 完全不重要

Q942. 您选择"我不经常吃它"
您认为该陈述有多重要？

- 极其重要
- 非常重要
- 一般重要
- 有点重要
- 完全不重要

Q943. 您选择"它的分量大小不合适"
您认为该陈述有多重要？

- 极其重要
- 非常重要
- 一般重要
- 有点重要
- 完全不重要

Q944. 您选择"它不足以填饱肚子"
您认为该陈述有多重要？

- 极其重要
- 非常重要
- 一般重要
- 有点重要
- 完全不重要

Q945. 您选择”它不健康“
您认为该陈述有多重要？

- 极其重要
- 非常重要
- 一般重要
- 有点重要
- 完全不重要

Q946. 您选择”它不方便“
您认为该陈述有多重要？

- 极其重要
- 非常重要
- 一般重要
- 有点重要
- 完全不重要

Q947. 您选择”我不想纵容自己“
您认为该陈述有多重要？

- 极其重要
- 非常重要
- 一般重要
- 有点重要
- 完全不重要

Q948. 您选择”它不适用于特定场合“
您认为该陈述有多重要？

- 极其重要

- 非常重要
- 一般重要
- 有点重要
- 完全不重要

Q949. 您选择"有其他人在的时候我不吃它"
您认为该陈述有多重要？

- 极其重要
- 非常重要
- 一般重要
- 有点重要
- 完全不重要

Q950. 您选择"价格太高"
您认为该陈述有多重要？

- 极其重要
- 非常重要
- 一般重要
- 有点重要
- 完全不重要

Q951. 您选择"我不喜欢它看起来的样子"
您认为该陈述有多重要？

- 极其重要
- 非常重要
- 一般重要
- 有点重要

完全不重要

Q952. 您选择”它卡路里太高“
您认为该陈述有多重要？

- 极其重要
- 非常重要
- 一般重要
- 有点重要
- 完全不重要

Q953. 您选择”我不应该吃它“
您认为该陈述有多重要？

- 极其重要
- 非常重要
- 一般重要
- 有点重要
- 完全不重要

Q954. 您选择”吃它显得我很土“
您认为该陈述有多重要？

- 极其重要
- 非常重要
- 一般重要
- 有点重要
- 完全不重要

Q955. 您选择”它不是我在那个时刻想吃的东西“

您认为该陈述有多重要？

- 极其重要
- 非常重要
- 一般重要
- 有点重要
- 完全不重要

Q956. 您选择”我最近刚吃过， 我不想太经常吃同样的东西“
您认为该陈述有多重要？

- 极其重要
- 非常重要
- 一般重要
- 有点重要
- 完全不重要

Q957. 您选择”我绝不选它。 我喜欢每天吃一样的食物“
您认为该陈述有多重要？

- 极其重要
- 非常重要
- 一般重要
- 有点重要
- 完全不重要

Q958. 您选择”这个食物让我感到伤心、孤单或沮丧“
您认为该陈述有多重要？

- 极其重要
- 非常重要

- 一般重要
- 有点重要
- 完全不重要

Q959. 您选择”它不是有机的“
您认为该陈述有多重要？

- 极其重要
- 非常重要
- 一般重要
- 有点重要
- 完全不重要

Reasons for Starchy food choices(Full RATA)

Q1073.
您吃白米饭吗?

- 是的， 我吃
- 不是， 我不吃

Q1074.
试回想您吃白米饭的原因。
您认为以下陈述有多重要？
”...因为我习惯吃它“

- 极其重要
- 非常重要
- 一般重要
- 有点重要
- 完全不重要

Q1075.
试回想您吃白米饭的原因。
您认为以下陈述有多重要？
”...因为它很健康“

- 极其重要
- 非常重要
- 一般重要
- 有点重要
- 完全不重要

Q1076.
试回想您吃白米饭的原因。
您认为以下陈述有多重要？
”...因为它准备起来很快“

- 极其重要
- 非常重要
- 一般重要
- 有点重要
- 完全不重要

Q1077.
试回想您吃白米饭的原因。
您认为以下陈述有多重要？
”...因为它的味道很好“

- 极其重要
- 非常重要
- 一般重要
- 有点重要

- 完全不重要

Q1078.

试回想您吃白米饭的原因。

您认为以下陈述有多重要？

”...为了纵容自己"

- 极其重要
- 非常重要
- 一般重要
- 有点重要
- 完全不重要

Q1079.

试回想您吃白米饭的原因。

您认为以下陈述有多重要？

”...因为它是天然的（例如没有经过基因改造)“

- 极其重要
- 非常重要
- 一般重要
- 有点重要
- 完全不重要

Q1080.

试回想您吃白米饭的原因。

您认为以下陈述有多重要？

”...因为我不想再花更多的钱“

- 极其重要
- 非常重要

- 一般重要
- 有点重要
- 完全不重要

Q1081.

试回想您吃白米饭的原因。
您认为以下陈述有多重要？

”...因为它的卡路里很低“

- 极其重要
- 非常重要
- 一般重要
- 有点重要
- 完全不重要

Q1082.

试回想您吃白米饭的原因。
您认为以下陈述有多重要？

”...因为我很沮丧“

- 极其重要
- 非常重要
- 一般重要
- 有点重要
- 完全不重要

Q1083.

试回想您吃白米饭的原因。
您认为以下陈述有多重要？

”...因为它让我在别人面前看起来很好“

- 极其重要
- 非常重要
- 一般重要
- 有点重要
- 完全不重要

Q1084.
试回想您吃白米饭的原因。
您认为以下陈述有多重要？

”....因为不吃它会不礼貌“

- 极其重要
- 非常重要
- 一般重要
- 有点重要
- 完全不重要

Q1085.
试回想您吃白米饭的原因。
您认为以下陈述有多重要？

”....因为它的呈现方式很有吸引力（例如包装)“

- 极其重要
- 非常重要
- 一般重要
- 有点重要
- 完全不重要

Q1086.
试回想您吃白米饭的原因。

您认为以下陈述有多重要？

”....这样我就可以和其他人共度时光“

- 极其重要
- 非常重要
- 一般重要
- 有点重要
- 完全不重要

Q1087.

试回想您吃白米饭的原因。

您认为以下陈述有多重要？

”....因为它是有机的“

- 极其重要
- 非常重要
- 一般重要
- 有点重要
- 完全不重要

Q1088.

试回想您吃白米饭的原因。

您认为以下陈述有多重要？

”....因为我从小吃到大“

- 极其重要
- 非常重要
- 一般重要
- 有点重要
- 完全不重要

Q1089.
试回想您吃白米饭的原因。
您认为以下陈述有多重要？

"...因为吃它让我很享受“

- 极其重要
- 非常重要
- 一般重要
- 有点重要
- 完全不重要

Q1090.
试回想您吃白米饭的原因。
您认为以下陈述有多重要？

"...因为我饿了“

- 极其重要
- 非常重要
- 一般重要
- 有点重要
- 完全不重要

Q1091.
试回想您吃白米饭的原因。
您认为以下陈述有多重要？

"...因为我经常吃它“

- 极其重要
- 非常重要
- 一般重要
- 有点重要

完全不重要

Q1092.
试回想您吃白米饭的原因。
您认为以下陈述有多重要？
"...因为我对它有食欲"

- 极其重要
- 非常重要
- 一般重要
- 有点重要
- 完全不重要

Q1093.
试回想您吃白米饭的原因。
您认为以下陈述有多重要？
"...因为它很容易准备“

- 极其重要
- 非常重要
- 一般重要
- 有点重要
- 完全不重要

Q1094.
试回想您吃白米饭的原因。
您认为以下陈述有多重要？
"...因为我很伤心"

- 极其重要
- 非常重要

- 一般重要
- 有点重要
- 完全不重要

Q1095.
试回想您吃白米饭的原因。
您认为以下陈述有多重要？

"...因为脂肪含量低"

- 极其重要
- 非常重要
- 一般重要
- 有点重要
- 完全不重要

Q1096.
试回想您吃白米饭的原因。
您认为以下陈述有多重要？

"...因为我从广告或在电视上看过它"

- 极其重要
- 非常重要
- 一般重要
- 有点重要
- 完全不重要

Q1097.
试回想您吃白米饭的原因。
您认为以下陈述有多重要？

"...因为它使社交聚会更加舒适"

- 极其重要
- 非常重要
- 一般重要
- 有点重要
- 完全不重要

Q1098.
试回想您吃白米饭的原因。
您认为以下陈述有多重要？

"...因为我应该吃它"

- 极其重要
- 非常重要
- 一般重要
- 有点重要
- 完全不重要

Q1099.
试回想您吃白米饭的原因。
您认为以下陈述有多重要？

"...因为其他人喜欢它"

- 极其重要
- 非常重要
- 一般重要
- 有点重要
- 完全不重要

Q1100.
试回想您吃白米饭的原因。

您认为以下陈述有多重要？

"...因为我感到孤独"

- 极其重要
- 非常重要
- 一般重要
- 有点重要
- 完全不重要

Q1101.

试回想您吃白米饭的原因。

您认为以下陈述有多重要？

"...因为我关注我的体重"

- 极其重要
- 非常重要
- 一般重要
- 有点重要
- 完全不重要

Q1102.

试回想您吃白米饭的原因。

您认为以下陈述有多重要？

"...因为它不含有害物质（例如杀虫剂，污染物，抗生素)"

- 极其重要
- 非常重要
- 一般重要
- 有点重要
- 完全不重要

Q1103.
试回想您吃白米饭的原因。
您认为以下陈述有多重要？

"...因为它最方便"

- 极其重要
- 非常重要
- 一般重要
- 有点重要
- 完全不重要

Q1104.
试回想您吃白米饭的原因。
您认为以下陈述有多重要？

"...因为我需要能量"

- 极其重要
- 非常重要
- 一般重要
- 有点重要
- 完全不重要

Q1105.
试回想您吃白米饭的原因。
您认为以下陈述有多重要？

"...因为我熟悉它"

- 极其重要
- 非常重要
- 一般重要
- 有点重要

完全不重要

Q1106.

试回想您吃白米饭的原因。
您认为以下陈述有多重要？

"...因为我喜欢它"

- 极其重要
- 非常重要
- 一般重要
- 有点重要
- 完全不重要

Q1107.

试回想您吃白米饭的原因。
您认为以下陈述有多重要？

"...因为它能令我感到愉悦的饱腹感"

- 极其重要
- 非常重要
- 一般重要
- 有点重要
- 完全不重要

Q1108.

试回想您吃白米饭的原因。
您认为以下陈述有多重要？

"...为了奖励自己"

- 极其重要
- 非常重要

- 一般重要
- 有点重要
- 完全不重要

Q1109.
试回想您吃白米饭的原因。
您认为以下陈述有多重要？

"...出于传统（例如家庭传统，特殊场合)"

- 极其重要
- 非常重要
- 一般重要
- 有点重要
- 完全不重要

Q1110.
试回想您吃白米饭的原因。
您认为以下陈述有多重要？

"...因为它适用于特定场合"

- 极其重要
- 非常重要
- 一般重要
- 有点重要
- 完全不重要

Q1111.
试回想您吃白米饭的原因。
您认为以下陈述有多重要？

"...保持均衡饮食"

- 极其重要
- 非常重要
- 一般重要
- 有点重要
- 完全不重要

Q1112.
试回想您吃白米饭的原因。
您认为以下陈述有多重要？

"...因为它的社交性"

- 极其重要
- 非常重要
- 一般重要
- 有点重要
- 完全不重要

Q1113.
试回想您吃白米饭的原因。
您认为以下陈述有多重要？

"...因为它的价格不贵"

- 极其重要
- 非常重要
- 一般重要
- 有点重要
- 完全不重要

Q1114.
试回想您吃白米饭的原因。

您认为以下陈述有多重要？

"...因为它自然而然地吸引了我（吸引人的颜色)"

- 极其重要
- 非常重要
- 一般重要
- 有点重要
- 完全不重要

Q1115.

试回想您吃白米饭的原因。

您认为以下陈述有多重要？

"...避免让那些试图让我开心的人失望"

- 极其重要
- 非常重要
- 一般重要
- 有点重要
- 完全不重要

Q1116.

试回想您吃白米饭的原因。

您认为以下陈述有多重要？

"...因为它很新潮"

- 极其重要
- 非常重要
- 一般重要
- 有点重要
- 完全不重要

Q1117.
试回想您吃白米饭的原因。
您认为以下陈述有多重要？

"...因为它打折"

- 极其重要
- 非常重要
- 一般重要
- 有点重要
- 完全不重要

Q1118.
试回想您吃白米饭的原因。
您认为以下陈述有多重要？

"...因为它让我保持身材"

- 极其重要
- 非常重要
- 一般重要
- 有点重要
- 完全不重要

Q1119.
试回想您吃白米饭的原因。
您认为以下陈述有多重要？

"...因为这是唯一的选择"

- 极其重要
- 非常重要
- 一般重要
- 有点重要

◯ 完全不重要

Q1120.

试回想您吃白米饭的原因。

您认为以下陈述有多重要？

"...因为我想每天都吃它"

◯ 极其重要

◯ 非常重要

◯ 一般重要

◯ 有点重要

◯ 完全不重要

Q1121.

试回想您不吃白米饭的原因。

您认为以下陈述有多重要？

”我不认为它是一种零食“

◯ 极其重要

◯ 非常重要

◯ 一般重要

◯ 有点重要

◯ 完全不重要

Q1122.

试回想您不吃白米饭的原因。

您认为以下陈述有多重要？

”我不喜欢它“

◯ 极其重要

◯ 非常重要

◯ 一般重要

- 有点重要
- 完全不重要

Q1123.
试回想您不吃白米饭的原因。
您认为以下陈述有多重要？
”我不经常吃它“

- 极其重要
- 非常重要
- 一般重要
- 有点重要
- 完全不重要

Q1124.
试回想您不吃白米饭的原因。
您认为以下陈述有多重要？
”它的分量大小不合适“

- 极其重要
- 非常重要
- 一般重要
- 有点重要
- 完全不重要

Q1125.
试回想您不吃白米饭的原因。
您认为以下陈述有多重要？
”它不足以填饱肚子“

- 极其重要
- 非常重要

- 一般重要
- 有点重要
- 完全不重要

Q1126.
试回想您不吃白米饭的原因。
您认为以下陈述有多重要？
”它不健康“

- 极其重要
- 非常重要
- 一般重要
- 有点重要
- 完全不重要

Q1127.
试回想您不吃白米饭的原因。
您认为以下陈述有多重要？
”它不方便“

- 极其重要
- 非常重要
- 一般重要
- 有点重要
- 完全不重要

Q1128.
试回想您不吃白米饭的原因。
您认为以下陈述有多重要？
”我不想纵容自己“

- 极其重要

- 非常重要
- 一般重要
- 有点重要
- 完全不重要

Q1129.

试回想您不吃白米饭的原因。
您认为以下陈述有多重要？

”它不适用于特定场合“

- 极其重要
- 非常重要
- 一般重要
- 有点重要
- 完全不重要

Q1130.

试回想您不吃白米饭的原因。
您认为以下陈述有多重要？

”有其他人在的时候我不吃它“

- 极其重要
- 非常重要
- 一般重要
- 有点重要
- 完全不重要

Q1131.

试回想您不吃白米饭的原因。
您认为以下陈述有多重要？

”价格太高“

- 极其重要
- 非常重要
- 一般重要
- 有点重要
- 完全不重要

Q1132.
试回想您不吃白米饭的原因。
您认为以下陈述有多重要？
”我不喜欢它看起来的样子“

- 极其重要
- 非常重要
- 一般重要
- 有点重要
- 完全不重要

Q1133.
试回想您不吃白米饭的原因。
您认为以下陈述有多重要？
”它卡路里太高“

- 极其重要
- 非常重要
- 一般重要
- 有点重要
- 完全不重要

Q1134.
试回想您不吃白米饭的原因。
您认为以下陈述有多重要？

”我不应该吃它（例如出于文化、宗教习俗）“

- 极其重要
- 非常重要
- 一般重要
- 有点重要
- 完全不重要

Q1135.

试回想您不吃白米饭的原因。
您认为以下陈述有多重要？
”吃它显得我很土“

- 极其重要
- 非常重要
- 一般重要
- 有点重要
- 完全不重要

Q1136.

试回想您不吃白米饭的原因。
您认为以下陈述有多重要？
”它不是我在那个时刻想吃的东西“

- 极其重要
- 非常重要
- 一般重要
- 有点重要
- 完全不重要

Q1137.

试回想您不吃白米饭的原因。

您认为以下陈述有多重要？

”我最近刚吃过， 我不想太经常吃同样的东西 ”

- 极其重要
- 非常重要
- 一般重要
- 有点重要
- 完全不重要

Q1138.

试回想您不吃白米饭的原因。
您认为以下陈述有多重要？

”我绝不选它。 我喜欢每天吃一样的食物 ”

- 极其重要
- 非常重要
- 一般重要
- 有点重要
- 完全不重要

Q1139.

试回想您不吃白米饭的原因。
您认为以下陈述有多重要？

”这个食物让我感到伤心、孤单或沮丧 ”

- 极其重要
- 非常重要
- 一般重要
- 有点重要
- 完全不重要

Q1140.
试回想您不吃白米饭的原因。
您认为以下陈述有多重要？
"它不是有机的"

- 极其重要
- 非常重要
- 一般重要
- 有点重要
- 完全不重要

Reasons for Protein foods Choices(Full RATA)

Q1005.
您吃红烧肉吗?

- 是的， 我吃
- 不是， 我不吃

Q1006.
试回想您吃红烧肉的原因。
您认为以下陈述有多重要？
"...因为我习惯吃它"

- 极其重要
- 非常重要
- 一般重要
- 有点重要
- 完全不重要

Q1007.

试回想您吃红烧肉的原因。
您认为以下陈述有多重要？
”...因为它很健康“

- 极其重要
- 非常重要
- 一般重要
- 有点重要
- 完全不重要

Q1008.
试回想您吃红烧肉的原因。
您认为以下陈述有多重要？
”...因为它准备起来很快“

- 极其重要
- 非常重要
- 一般重要
- 有点重要
- 完全不重要

Q1009.
试回想您吃红烧肉的原因。
您认为以下陈述有多重要？
”...因为它的味道很好“

- 极其重要
- 非常重要
- 一般重要
- 有点重要
- 完全不重要

Q1010.
试回想您吃红烧肉的原因。
您认为以下陈述有多重要？
”...为了纵容自己"

- 极其重要
- 非常重要
- 一般重要
- 有点重要
- 完全不重要

Q1011.
试回想您吃红烧肉的原因。
您认为以下陈述有多重要？
”...因为它是天然的（例如没有经过基因改造)“

- 极其重要
- 非常重要
- 一般重要
- 有点重要
- 完全不重要

Q1012.
试回想您吃红烧肉的原因。
您认为以下陈述有多重要？
”...因为我不想再花更多的钱“

- 极其重要
- 非常重要
- 一般重要
- 有点重要
- 完全不重要

Q1013.
试回想您吃红烧肉的原因。
您认为以下陈述有多重要？
”...因为它的卡路里很低“

- 极其重要
- 非常重要
- 一般重要
- 有点重要
- 完全不重要

Q1014.
试回想您吃红烧肉的原因。
您认为以下陈述有多重要？
”...因为我很沮丧“

- 极其重要
- 非常重要
- 一般重要
- 有点重要
- 完全不重要

Q1015.
试回想您吃红烧肉的原因。
您认为以下陈述有多重要？
”...因为它让我在别人面前看起来很好“

- 极其重要
- 非常重要
- 一般重要

- 有点重要
- 完全不重要

Q1016.
试回想您吃红烧肉的原因。
您认为以下陈述有多重要？

”....因为不吃它会不礼貌“

- 极其重要
- 非常重要
- 一般重要
- 有点重要
- 完全不重要

Q1017.
试回想您吃红烧肉的原因。
您认为以下陈述有多重要？

”....因为它的呈现方式很有吸引力（例如包装)“

- 极其重要
- 非常重要
- 一般重要
- 有点重要
- 完全不重要

Q1018.
试回想您吃红烧肉的原因。
您认为以下陈述有多重要？

”....这样我就可以和其他人共度时光“

- 极其重要

- 非常重要
- 一般重要
- 有点重要
- 完全不重要

Q1019.
试回想您吃红烧肉的原因。
您认为以下陈述有多重要？
”....因为它是有机的“

- 极其重要
- 非常重要
- 一般重要
- 有点重要
- 完全不重要

Q1020.
试回想您吃红烧肉的原因。
您认为以下陈述有多重要？
”....因为我从小吃到大“

- 极其重要
- 非常重要
- 一般重要
- 有点重要
- 完全不重要

Q1021.
试回想您吃红烧肉的原因。
您认为以下陈述有多重要？

"...因为吃它让我很享受“

- 极其重要
- 非常重要
- 一般重要
- 有点重要
- 完全不重要

Q1022.

试回想您吃红烧肉的原因。
您认为以下陈述有多重要？

"...因为我饿了“

- 极其重要
- 非常重要
- 一般重要
- 有点重要
- 完全不重要

Q1023.

试回想您吃红烧肉的原因。
您认为以下陈述有多重要？

"...因为我经常吃它“

- 极其重要
- 非常重要
- 一般重要
- 有点重要
- 完全不重要

Q1024.

试回想您吃红烧肉的原因。
您认为以下陈述有多重要？
"...因为我对它有食欲"

- 极其重要
- 非常重要
- 一般重要
- 有点重要
- 完全不重要

Q1025.
试回想您吃红烧肉的原因。
您认为以下陈述有多重要？
"...因为它很容易准备“

- 极其重要
- 非常重要
- 一般重要
- 有点重要
- 完全不重要

Q1026.
试回想您吃红烧肉的原因。
您认为以下陈述有多重要？
"...因为我很伤心"

- 极其重要
- 非常重要
- 一般重要
- 有点重要
- 完全不重要

Q1027.
试回想您吃红烧肉的原因。
您认为以下陈述有多重要？

"...因为脂肪含量低"

- 极其重要
- 非常重要
- 一般重要
- 有点重要
- 完全不重要

Q1028.
试回想您吃红烧肉的原因。
您认为以下陈述有多重要？

"...因为我从广告或在电视上看过它"

- 极其重要
- 非常重要
- 一般重要
- 有点重要
- 完全不重要

Q1029.
试回想您吃红烧肉的原因。
您认为以下陈述有多重要？

"...因为它使社交聚会更加舒适"

- 极其重要
- 非常重要
- 一般重要
- 有点重要

完全不重要

Q1030.
试回想您吃红烧肉的原因。
您认为以下陈述有多重要？

"...因为我应该吃它"

- 极其重要
- 非常重要
- 一般重要
- 有点重要
- 完全不重要

Q1031.
试回想您吃红烧肉的原因。
您认为以下陈述有多重要？

"...因为其他人喜欢它"

- 极其重要
- 非常重要
- 一般重要
- 有点重要
- 完全不重要

Q1032.
试回想您吃红烧肉的原因。
您认为以下陈述有多重要？

"...因为我感到孤独"

- 极其重要
- 非常重要

- 一般重要
- 有点重要
- 完全不重要

Q1033.
试回想您吃红烧肉的原因。
您认为以下陈述有多重要？

"...因为我关注我的体重"

- 极其重要
- 非常重要
- 一般重要
- 有点重要
- 完全不重要

Q1034.
试回想您吃红烧肉的原因。
您认为以下陈述有多重要？

"...因为它不含有害物质（例如杀虫剂，污染物，抗生素)"

- 极其重要
- 非常重要
- 一般重要
- 有点重要
- 完全不重要

Q1035.
试回想您吃红烧肉的原因。
您认为以下陈述有多重要？

"...因为它最方便"

- 极其重要
- 非常重要
- 一般重要
- 有点重要
- 完全不重要

Q1036.
试回想您吃红烧肉的原因。
您认为以下陈述有多重要？

"...因为我需要能量"

- 极其重要
- 非常重要
- 一般重要
- 有点重要
- 完全不重要

Q1037.
试回想您吃红烧肉的原因。
您认为以下陈述有多重要？

"...因为我熟悉它"

- 极其重要
- 非常重要
- 一般重要
- 有点重要
- 完全不重要

Q1038.
试回想您吃红烧肉的原因。

您认为以下陈述有多重要？

"...因为我喜欢它"

- 极其重要
- 非常重要
- 一般重要
- 有点重要
- 完全不重要

Q1039.

试回想您吃红烧肉的原因。

您认为以下陈述有多重要？

"...因为它能令我感到愉悦的饱腹感"

- 极其重要
- 非常重要
- 一般重要
- 有点重要
- 完全不重要

Q1040.

试回想您吃红烧肉的原因。

您认为以下陈述有多重要？

"...为了奖励自己"

- 极其重要
- 非常重要
- 一般重要
- 有点重要
- 完全不重要

Q1041.
试回想您吃红烧肉的原因。
您认为以下陈述有多重要？
"...出于传统（例如家庭传统，特殊场合)"

- 极其重要
- 非常重要
- 一般重要
- 有点重要
- 完全不重要

Q1042.
试回想您吃红烧肉的原因。
您认为以下陈述有多重要？
"...因为它适用于特定场合"

- 极其重要
- 非常重要
- 一般重要
- 有点重要
- 完全不重要

Q1043.
试回想您吃红烧肉的原因。
您认为以下陈述有多重要？
"...保持均衡饮食"

- 极其重要
- 非常重要
- 一般重要
- 有点重要

○ 完全不重要

Q1044.
试回想您吃红烧肉的原因。
您认为以下陈述有多重要？

"...因为它的社交性"

○ 极其重要
○ 非常重要
○ 一般重要
○ 有点重要
○ 完全不重要

Q1045.
试回想您吃红烧肉的原因。
您认为以下陈述有多重要？

"...因为它的价格不贵"

○ 极其重要
○ 非常重要
○ 一般重要
○ 有点重要
○ 完全不重要

Q1046.
试回想您吃红烧肉的原因。
您认为以下陈述有多重要？

"...因为它自然而然地吸引了我（吸引人的颜色)"

○ 极其重要
○ 非常重要

- 一般重要
- 有点重要
- 完全不重要

Q1047.

试回想您吃红烧肉的原因。

您认为以下陈述有多重要？

"...避免让那些试图让我开心的人失望"

- 极其重要
- 非常重要
- 一般重要
- 有点重要
- 完全不重要

Q1048.

试回想您吃红烧肉的原因。

您认为以下陈述有多重要？

"...因为它很新潮"

- 极其重要
- 非常重要
- 一般重要
- 有点重要
- 完全不重要

Q1049.

试回想您吃红烧肉的原因。

您认为以下陈述有多重要？

"...因为它打折"

- 极其重要
- 非常重要
- 一般重要
- 有点重要
- 完全不重要

Q1050.
试回想您吃红烧肉的原因。
您认为以下陈述有多重要？

"...因为它让我保持身材"

- 极其重要
- 非常重要
- 一般重要
- 有点重要
- 完全不重要

Q1051.
试回想您吃红烧肉的原因。
您认为以下陈述有多重要？

"...因为这是唯一的选择"

- 极其重要
- 非常重要
- 一般重要
- 有点重要
- 完全不重要

Q1052.
试回想您吃红烧肉的原因。

您认为以下陈述有多重要？
"...因为我想每天都吃它"

- 极其重要
- 非常重要
- 一般重要
- 有点重要
- 完全不重要

Q1053.
试回想您不吃红烧肉的原因。
您认为以下陈述有多重要？
”我不认为它是一种零食“

- 极其重要
- 非常重要
- 一般重要
- 有点重要
- 完全不重要

Q1054.
试回想您不吃红烧肉的原因。
您认为以下陈述有多重要？
”我不喜欢它“

- 极其重要
- 非常重要
- 一般重要
- 有点重要
- 完全不重要

Q1055.

试回想您不吃红烧肉的原因。
您认为以下陈述有多重要？
”我不经常吃它“

- 极其重要
- 非常重要
- 一般重要
- 有点重要
- 完全不重要

Q1056.
试回想您不吃红烧肉的原因。
您认为以下陈述有多重要？
”它的分量大小不合适“

- 极其重要
- 非常重要
- 一般重要
- 有点重要
- 完全不重要

Q1057.
试回想您不吃红烧肉的原因。
您认为以下陈述有多重要？
”它不足以填饱肚子“

- 极其重要
- 非常重要
- 一般重要
- 有点重要
- 完全不重要

Q1058.

试回想您不吃红烧肉的原因。

您认为以下陈述有多重要？

"它不健康"

- 极其重要
- 非常重要
- 一般重要
- 有点重要
- 完全不重要

Q1059.

试回想您不吃红烧肉的原因。

您认为以下陈述有多重要？

"它不方便"

- 极其重要
- 非常重要
- 一般重要
- 有点重要
- 完全不重要

Q1060.

试回想您不吃红烧肉的原因。

您认为以下陈述有多重要？

"我不想纵容自己"

- 极其重要
- 非常重要
- 一般重要
- 有点重要
- 完全不重要

Q1061.

试回想您不吃红烧肉的原因。

您认为以下陈述有多重要？

”它不适用于特定场合“

- 极其重要
- 非常重要
- 一般重要
- 有点重要
- 完全不重要

Q1062.

试回想您不吃红烧肉的原因。

您认为以下陈述有多重要？

”有其他人在的时候我不吃它“

- 极其重要
- 非常重要
- 一般重要
- 有点重要
- 完全不重要

Q1063.

试回想您不吃红烧肉的原因。

您认为以下陈述有多重要？

”价格太高“

- 极其重要
- 非常重要
- 一般重要
- 有点重要
- 完全不重要

Q1064.
试回想您不吃红烧肉的原因。
您认为以下陈述有多重要？
”我不喜欢它看起来的样子“

- 极其重要
- 非常重要
- 一般重要
- 有点重要
- 完全不重要

Q1065.
试回想您不吃红烧肉的原因。
您认为以下陈述有多重要？
”它卡路里太高“

- 极其重要
- 非常重要
- 一般重要
- 有点重要
- 完全不重要

Q1066.
试回想您不吃红烧肉的原因。
您认为以下陈述有多重要？
”我不应该吃它（例如出于文化、宗教习俗）“

- 极其重要
- 非常重要
- 一般重要
- 有点重要

○ 完全不重要

Q1067.

试回想您不吃红烧肉的原因。
您认为以下陈述有多重要？

”吃它显得我很土“

○ 极其重要
○ 非常重要
○ 一般重要
○ 有点重要
○ 完全不重要

Q1068.

试回想您不吃红烧肉的原因。
您认为以下陈述有多重要？

”它不是我在那个时刻想吃的东西“

○ 极其重要
○ 非常重要
○ 一般重要
○ 有点重要
○ 完全不重要

Q1069.

试回想您不吃红烧肉的原因。
您认为以下陈述有多重要？

”我最近刚吃过， 我不想太经常吃同样的东西 ”

○ 极其重要
○ 非常重要
○ 一般重要

- 有点重要
- 完全不重要

Q1070.
试回想您不吃红烧肉的原因。
您认为以下陈述有多重要？

”我绝不选它。 我喜欢每天吃一样的食物 ”

- 极其重要
- 非常重要
- 一般重要
- 有点重要
- 完全不重要

Q1071.
试回想您不吃红烧肉的原因。
您认为以下陈述有多重要？

”这个食物让我感到伤心、孤单或沮丧 ”

- 极其重要
- 非常重要
- 一般重要
- 有点重要
- 完全不重要

Q1072.
试回想您不吃红烧肉的原因。
您认为以下陈述有多重要？

”它不是有机的”

- 极其重要

- 非常重要
- 一般重要
- 有点重要
- 完全不重要

Reasons for Dairy Food Choices(Full RATA)

Q1141.
您吃豆浆吗?

- 是的， 我吃
- 不是， 我不吃

Q1142.
试回想您吃豆浆的原因。
您认为以下陈述有多重要？
”...因为我习惯吃它“

- 极其重要
- 非常重要
- 一般重要
- 有点重要
- 完全不重要

Q1143.
试回想您吃豆浆的原因。
您认为以下陈述有多重要？
”...因为它很健康“

- 极其重要
- 非常重要

- 一般重要
- 有点重要
- 完全不重要

Q1144.
试回想您吃豆浆的原因。
您认为以下陈述有多重要？
”...因为它准备起来很快“

- 极其重要
- 非常重要
- 一般重要
- 有点重要
- 完全不重要

Q1145.
试回想您吃豆浆的原因。
您认为以下陈述有多重要？
”...因为它的味道很好“

- 极其重要
- 非常重要
- 一般重要
- 有点重要
- 完全不重要

Q1146.
试回想您吃豆浆的原因。
您认为以下陈述有多重要？
”...为了纵容自己"

- 极其重要

- 非常重要
- 一般重要
- 有点重要
- 完全不重要

Q1147.

试回想您吃豆浆的原因。
您认为以下陈述有多重要？

”...因为它是天然的（例如没有经过基因改造)“

- 极其重要
- 非常重要
- 一般重要
- 有点重要
- 完全不重要

Q1148.

试回想您吃豆浆的原因。
您认为以下陈述有多重要？

”...因为我不想再花更多的钱“

- 极其重要
- 非常重要
- 一般重要
- 有点重要
- 完全不重要

Q1149.

试回想您吃豆浆的原因。
您认为以下陈述有多重要？

”...因为它的卡路里很低“

- 极其重要
- 非常重要
- 一般重要
- 有点重要
- 完全不重要

Q1150.
试回想您吃豆浆的原因。
您认为以下陈述有多重要？

”...因为我很沮丧“

- 极其重要
- 非常重要
- 一般重要
- 有点重要
- 完全不重要

Q1151.
试回想您吃豆浆的原因。
您认为以下陈述有多重要？

”...因为它让我在别人面前看起来很好“

- 极其重要
- 非常重要
- 一般重要
- 有点重要
- 完全不重要

Q1152.

试回想您吃豆浆的原因。
您认为以下陈述有多重要？

”....因为不吃它会不礼貌“

- 极其重要
- 非常重要
- 一般重要
- 有点重要
- 完全不重要

Q1153.

试回想您吃豆浆的原因。
您认为以下陈述有多重要？

”....因为它的呈现方式很有吸引力（例如包装)“

- 极其重要
- 非常重要
- 一般重要
- 有点重要
- 完全不重要

Q1154.

试回想您吃豆浆的原因。
您认为以下陈述有多重要？

”....这样我就可以和其他人共度时光“

- 极其重要
- 非常重要
- 一般重要
- 有点重要
- 完全不重要

Q1155.
试回想您吃豆浆的原因。
您认为以下陈述有多重要？
”....因为它是有机的“

- 极其重要
- 非常重要
- 一般重要
- 有点重要
- 完全不重要

Q1156.
试回想您吃豆浆的原因。
您认为以下陈述有多重要？
”....因为我从小吃到大“

- 极其重要
- 非常重要
- 一般重要
- 有点重要
- 完全不重要

Q1157.
试回想您吃豆浆的原因。
您认为以下陈述有多重要？
"...因为吃它让我很享受“

- 极其重要
- 非常重要
- 一般重要
- 有点重要

- 完全不重要

Q1158.
试回想您吃豆浆的原因。
您认为以下陈述有多重要？

"...因为我饿了“

- 极其重要
- 非常重要
- 一般重要
- 有点重要
- 完全不重要

Q1159.
试回想您吃豆浆的原因。
您认为以下陈述有多重要？

"...因为我经常吃它“

- 极其重要
- 非常重要
- 一般重要
- 有点重要
- 完全不重要

Q1160.
试回想您吃豆浆的原因。
您认为以下陈述有多重要？
"...因为我对它有食欲"

- 极其重要
- 非常重要

- 一般重要
- 有点重要
- 完全不重要

Q1161.
试回想您吃豆浆的原因。
您认为以下陈述有多重要？
"...因为它很容易准备“

- 极其重要
- 非常重要
- 一般重要
- 有点重要
- 完全不重要

Q1162.
试回想您吃豆浆的原因。
您认为以下陈述有多重要？
"...因为我很伤心"

- 极其重要
- 非常重要
- 一般重要
- 有点重要
- 完全不重要

Q1163.
试回想您吃豆浆的原因。
您认为以下陈述有多重要？
"...因为脂肪含量低"

- 极其重要
- 非常重要
- 一般重要
- 有点重要
- 完全不重要

Q1164.
试回想您吃豆浆的原因。
您认为以下陈述有多重要？

"...因为我从广告或在电视上看过它"

- 极其重要
- 非常重要
- 一般重要
- 有点重要
- 完全不重要

Q1165.
试回想您吃豆浆的原因。
您认为以下陈述有多重要？

"...因为它使社交聚会更加舒适"

- 极其重要
- 非常重要
- 一般重要
- 有点重要
- 完全不重要

Q1166.
试回想您吃豆浆的原因。

您认为以下陈述有多重要？

"...因为我应该吃它"

- 极其重要
- 非常重要
- 一般重要
- 有点重要
- 完全不重要

Q1167.

试回想您吃豆浆的原因。

您认为以下陈述有多重要？

"...因为其他人喜欢它"

- 极其重要
- 非常重要
- 一般重要
- 有点重要
- 完全不重要

Q1168.

试回想您吃豆浆的原因。

您认为以下陈述有多重要？

"...因为我感到孤独"

- 极其重要
- 非常重要
- 一般重要
- 有点重要
- 完全不重要

Q1169.
试回想您吃豆浆的原因。
您认为以下陈述有多重要？

"...因为我关注我的体重"

- 极其重要
- 非常重要
- 一般重要
- 有点重要
- 完全不重要

Q1170.
试回想您吃豆浆的原因。
您认为以下陈述有多重要？

"...因为它不含有害物质（例如杀虫剂，污染物，抗生素)"

- 极其重要
- 非常重要
- 一般重要
- 有点重要
- 完全不重要

Q1171.
试回想您吃豆浆的原因。
您认为以下陈述有多重要？

"...因为它最方便"

- 极其重要
- 非常重要
- 一般重要
- 有点重要

○ 完全不重要

Q1172.
试回想您吃豆浆的原因。
您认为以下陈述有多重要？

"...因为我需要能量"

○ 极其重要
○ 非常重要
○ 一般重要
○ 有点重要
○ 完全不重要

Q1173.
试回想您吃豆浆的原因。
您认为以下陈述有多重要？

"...因为我熟悉它"

○ 极其重要
○ 非常重要
○ 一般重要
○ 有点重要
○ 完全不重要

Q1174.
试回想您吃豆浆的原因。
您认为以下陈述有多重要？

"...因为我喜欢它"

○ 极其重要
○ 非常重要

- 一般重要
- 有点重要
- 完全不重要

Q1175.
试回想您吃豆浆的原因。
您认为以下陈述有多重要？

"...因为它能令我感到愉悦的饱腹感"

- 极其重要
- 非常重要
- 一般重要
- 有点重要
- 完全不重要

Q1176.
试回想您吃豆浆的原因。
您认为以下陈述有多重要？

"...为了奖励自己"

- 极其重要
- 非常重要
- 一般重要
- 有点重要
- 完全不重要

Q1177.
试回想您吃豆浆的原因。
您认为以下陈述有多重要？

"...出于传统（例如家庭传统，特殊场合)"

- 极其重要
- 非常重要
- 一般重要
- 有点重要
- 完全不重要

Q1178.
试回想您吃豆浆的原因。
您认为以下陈述有多重要？
"...因为它适用于特定场合"

- 极其重要
- 非常重要
- 一般重要
- 有点重要
- 完全不重要

Q1179.
试回想您吃豆浆的原因。
您认为以下陈述有多重要？
"...保持均衡饮食"

- 极其重要
- 非常重要
- 一般重要
- 有点重要
- 完全不重要

Q1180.
试回想您吃豆浆的原因。

您认为以下陈述有多重要？

"...因为它的社交性"

- 极其重要
- 非常重要
- 一般重要
- 有点重要
- 完全不重要

Q1181.

试回想您吃豆浆的原因。

您认为以下陈述有多重要？

"...因为它的价格不贵"

- 极其重要
- 非常重要
- 一般重要
- 有点重要
- 完全不重要

Q1182.

试回想您吃豆浆的原因。

您认为以下陈述有多重要？

"...因为它自然而然地吸引了我（吸引人的颜色)"

- 极其重要
- 非常重要
- 一般重要
- 有点重要
- 完全不重要

Q1183.
试回想您吃豆浆的原因。
您认为以下陈述有多重要？
"...避免让那些试图让我开心的人失望"

- 极其重要
- 非常重要
- 一般重要
- 有点重要
- 完全不重要

Q1184.
试回想您吃豆浆的原因。
您认为以下陈述有多重要？
"...因为它很新潮"

- 极其重要
- 非常重要
- 一般重要
- 有点重要
- 完全不重要

Q1185.
试回想您吃豆浆的原因。
您认为以下陈述有多重要？
"...因为它打折"

- 极其重要
- 非常重要
- 一般重要
- 有点重要

- 完全不重要

Q1186.
试回想您吃豆浆的原因。
您认为以下陈述有多重要？

"...因为它让我保持身材"

- 极其重要
- 非常重要
- 一般重要
- 有点重要
- 完全不重要

Q1187.
试回想您吃豆浆的原因。
您认为以下陈述有多重要？

"...因为这是唯一的选择"

- 极其重要
- 非常重要
- 一般重要
- 有点重要
- 完全不重要

Q1188.
试回想您吃豆浆的原因。
您认为以下陈述有多重要？

"...因为我想每天都吃它"

- 极其重要
- 非常重要

- 一般重要
- 有点重要
- 完全不重要

Q1189.

试回想您不吃豆浆的原因。
您认为以下陈述有多重要？
”我不认为它是一种零食“

- 极其重要
- 非常重要
- 一般重要
- 有点重要
- 完全不重要

Q1190.

试回想您不吃豆浆的原因。
您认为以下陈述有多重要？
”我不喜欢它“

- 极其重要
- 非常重要
- 一般重要
- 有点重要
- 完全不重要

Q1191.

试回想您不吃豆浆的原因。
您认为以下陈述有多重要？
”我不经常吃它“

- 极其重要

- 非常重要
- 一般重要
- 有点重要
- 完全不重要

Q1192.

试回想您不吃豆浆的原因。

您认为以下陈述有多重要？

”它的分量大小不合适“

- 极其重要
- 非常重要
- 一般重要
- 有点重要
- 完全不重要

Q1193.

试回想您不吃豆浆的原因。

您认为以下陈述有多重要？

”它不足以填饱肚子“

- 极其重要
- 非常重要
- 一般重要
- 有点重要
- 完全不重要

Q1194.

试回想您不吃豆浆的原因。

您认为以下陈述有多重要？

”它不健康“

- 极其重要
- 非常重要
- 一般重要
- 有点重要
- 完全不重要

Q1195.
试回想您不吃豆浆的原因。
您认为以下陈述有多重要？
”它不方便“

- 极其重要
- 非常重要
- 一般重要
- 有点重要
- 完全不重要

Q1196.
试回想您不吃豆浆的原因。
您认为以下陈述有多重要？
”我不想纵容自己“

- 极其重要
- 非常重要
- 一般重要
- 有点重要
- 完全不重要

Q1197.
试回想您不吃豆浆的原因。
您认为以下陈述有多重要？
”它不适用于特定场合“

- 极其重要
- 非常重要
- 一般重要
- 有点重要
- 完全不重要

Q1198.
试回想您不吃豆浆的原因。
您认为以下陈述有多重要？
”有其他人在的时候我不吃它“

- 极其重要
- 非常重要
- 一般重要
- 有点重要
- 完全不重要

Q1199.
试回想您不吃豆浆的原因。
您认为以下陈述有多重要？
”价格太高“

- 极其重要
- 非常重要
- 一般重要
- 有点重要
- 完全不重要

Q1200.
试回想您不吃豆浆的原因。
您认为以下陈述有多重要？

”我不喜欢它看起来的样子“

- 极其重要
- 非常重要
- 一般重要
- 有点重要
- 完全不重要

Q1201.
试回想您不吃豆浆的原因。
您认为以下陈述有多重要？
”它卡路里太高“

- 极其重要
- 非常重要
- 一般重要
- 有点重要
- 完全不重要

Q1202.
试回想您不吃豆浆的原因。
您认为以下陈述有多重要？
”我不应该吃它（例如出于文化、宗教习俗）“

- 极其重要
- 非常重要
- 一般重要
- 有点重要
- 完全不重要

Q1203.
试回想您不吃豆浆的原因。

您认为以下陈述有多重要？

”吃它显得我很土“

- 极其重要
- 非常重要
- 一般重要
- 有点重要
- 完全不重要

Q1204.

试回想您不吃豆浆的原因。

您认为以下陈述有多重要？

”它不是我在那个时刻想吃的东西“

- 极其重要
- 非常重要
- 一般重要
- 有点重要
- 完全不重要

Q1205.

试回想您不吃豆浆的原因。

您认为以下陈述有多重要？

”我最近刚吃过， 我不想太经常吃同样的东西 ”

- 极其重要
- 非常重要
- 一般重要
- 有点重要
- 完全不重要

Q1206.

试回想您不吃豆浆的原因。
您认为以下陈述有多重要？

”我绝不选它。 我喜欢每天吃一样的食物 ”

- 极其重要
- 非常重要
- 一般重要
- 有点重要
- 完全不重要

Q1207.
试回想您不吃豆浆的原因。
您认为以下陈述有多重要？

”这个食物让我感到伤心、孤单或沮丧 ”

- 极其重要
- 非常重要
- 一般重要
- 有点重要
- 完全不重要

Q1208.
试回想您不吃豆浆的原因。
您认为以下陈述有多重要？

”它不是有机的”

- 极其重要
- 非常重要
- 一般重要
- 有点重要
- 完全不重要

Reasons for Fruit Choices(Full RATA)

Q1209.
您吃香蕉吗?

- 是的， 我吃
- 不是， 我不吃

Q1210.
试回想您吃香蕉的原因。
您认为以下陈述有多重要？
”...因为我习惯吃它“

- 极其重要
- 非常重要
- 一般重要
- 有点重要
- 完全不重要

Q1211.
试回想您吃香蕉的原因。
您认为以下陈述有多重要？
”...因为它很健康“

- 极其重要
- 非常重要
- 一般重要
- 有点重要
- 完全不重要

Q1212.

试回想您吃香蕉的原因。
您认为以下陈述有多重要？
”...因为它准备起来很快“

- 极其重要
- 非常重要
- 一般重要
- 有点重要
- 完全不重要

Q1213.
试回想您吃香蕉的原因。
您认为以下陈述有多重要？
”...因为它的味道很好“

- 极其重要
- 非常重要
- 一般重要
- 有点重要
- 完全不重要

Q1214.
试回想您吃香蕉的原因。
您认为以下陈述有多重要？
”...为了纵容自己"

- 极其重要
- 非常重要
- 一般重要
- 有点重要
- 完全不重要

Q1215.

试回想您吃香蕉的原因。
您认为以下陈述有多重要？

”...因为它是天然的（例如没有经过基因改造)“

- 极其重要
- 非常重要
- 一般重要
- 有点重要
- 完全不重要

Q1216.

试回想您吃香蕉的原因。
您认为以下陈述有多重要？

”...因为我不想再花更多的钱“

- 极其重要
- 非常重要
- 一般重要
- 有点重要
- 完全不重要

Q1217.

试回想您吃香蕉的原因。
您认为以下陈述有多重要？

”...因为它的卡路里很低“

- 极其重要
- 非常重要
- 一般重要
- 有点重要

完全不重要

Q1218.
试回想您吃香蕉的原因。
您认为以下陈述有多重要？
”...因为我很沮丧“

- 极其重要
- 非常重要
- 一般重要
- 有点重要
- 完全不重要

Q1219.
试回想您吃香蕉的原因。
您认为以下陈述有多重要？
”...因为它让我在别人面前看起来很好“

- 极其重要
- 非常重要
- 一般重要
- 有点重要
- 完全不重要

Q1220.
试回想您吃香蕉的原因。
您认为以下陈述有多重要？
”....因为不吃它会不礼貌“

- 极其重要
- 非常重要

- 一般重要
- 有点重要
- 完全不重要

Q1221.
试回想您吃香蕉的原因。
您认为以下陈述有多重要？

”....因为它的呈现方式很有吸引力（例如包装)“

- 极其重要
- 非常重要
- 一般重要
- 有点重要
- 完全不重要

Q1222.
试回想您吃香蕉的原因。
您认为以下陈述有多重要？

”....这样我就可以和其他人共度时光“

- 极其重要
- 非常重要
- 一般重要
- 有点重要
- 完全不重要

Q1223.
试回想您吃香蕉的原因。
您认为以下陈述有多重要？

”....因为它是有机的“

- 极其重要
- 非常重要
- 一般重要
- 有点重要
- 完全不重要

Q1224.
试回想您吃香蕉的原因。
您认为以下陈述有多重要？
”....因为我从小吃到大“

- 极其重要
- 非常重要
- 一般重要
- 有点重要
- 完全不重要

Q1225.
试回想您吃香蕉的原因。
您认为以下陈述有多重要？
"...因为吃它让我很享受“

- 极其重要
- 非常重要
- 一般重要
- 有点重要
- 完全不重要

Q1226.
试回想您吃香蕉的原因。

您认为以下陈述有多重要？

"...因为我饿了“

- 极其重要
- 非常重要
- 一般重要
- 有点重要
- 完全不重要

Q1227.

试回想您吃香蕉的原因。

您认为以下陈述有多重要？

"...因为我经常吃它“

- 极其重要
- 非常重要
- 一般重要
- 有点重要
- 完全不重要

Q1228.

试回想您吃香蕉的原因。

您认为以下陈述有多重要？

"...因为我对它有食欲"

- 极其重要
- 非常重要
- 一般重要
- 有点重要
- 完全不重要

Q1229.
试回想您吃香蕉的原因。
您认为以下陈述有多重要？
"...因为它很容易准备“

- 极其重要
- 非常重要
- 一般重要
- 有点重要
- 完全不重要

Q1230.
试回想您吃香蕉的原因。
您认为以下陈述有多重要？
"...因为我很伤心"

- 极其重要
- 非常重要
- 一般重要
- 有点重要
- 完全不重要

Q1231.
试回想您吃香蕉的原因。
您认为以下陈述有多重要？
"...因为脂肪含量低"

- 极其重要
- 非常重要
- 一般重要
- 有点重要

- 完全不重要

Q1232.
试回想您吃香蕉的原因。
您认为以下陈述有多重要？
"...因为我从广告或在电视上看过它"

- 极其重要
- 非常重要
- 一般重要
- 有点重要
- 完全不重要

Q1233.
试回想您吃香蕉的原因。
您认为以下陈述有多重要？
"...因为它使社交聚会更加舒适"

- 极其重要
- 非常重要
- 一般重要
- 有点重要
- 完全不重要

Q1234.
试回想您吃香蕉的原因。
您认为以下陈述有多重要？
"...因为我应该吃它"

- 极其重要
- 非常重要

- 一般重要
- 有点重要
- 完全不重要

Q1235.
试回想您吃香蕉的原因。
您认为以下陈述有多重要？

"...因为其他人喜欢它"

- 极其重要
- 非常重要
- 一般重要
- 有点重要
- 完全不重要

Q1236.
试回想您吃香蕉的原因。
您认为以下陈述有多重要？

"...因为我感到孤独"

- 极其重要
- 非常重要
- 一般重要
- 有点重要
- 完全不重要

Q1237.
试回想您吃香蕉的原因。
您认为以下陈述有多重要？

"...因为我关注我的体重"

- 极其重要
- 非常重要
- 一般重要
- 有点重要
- 完全不重要

Q1238.

试回想您吃香蕉的原因。
您认为以下陈述有多重要？

"...因为它不含有害物质（例如杀虫剂，污染物，抗生素)"

- 极其重要
- 非常重要
- 一般重要
- 有点重要
- 完全不重要

Q1239.

试回想您吃香蕉的原因。
您认为以下陈述有多重要？

"...因为它最方便"

- 极其重要
- 非常重要
- 一般重要
- 有点重要
- 完全不重要

Q1240.

试回想您吃香蕉的原因。

您认为以下陈述有多重要？

"...因为我需要能量"

- 极其重要
- 非常重要
- 一般重要
- 有点重要
- 完全不重要

Q1241.

试回想您吃香蕉的原因。

您认为以下陈述有多重要？

"...因为我熟悉它"

- 极其重要
- 非常重要
- 一般重要
- 有点重要
- 完全不重要

Q1242.

试回想您吃香蕉的原因。

您认为以下陈述有多重要？

"...因为我喜欢它"

- 极其重要
- 非常重要
- 一般重要
- 有点重要
- 完全不重要

Q1243.

试回想您吃香蕉的原因。
您认为以下陈述有多重要？

"...因为它能令我感到愉悦的饱腹感"

- 极其重要
- 非常重要
- 一般重要
- 有点重要
- 完全不重要

Q1244.

试回想您吃香蕉的原因。
您认为以下陈述有多重要？

"...为了奖励自己"

- 极其重要
- 非常重要
- 一般重要
- 有点重要
- 完全不重要

Q1245.

试回想您吃香蕉的原因。
您认为以下陈述有多重要？

"...出于传统（例如家庭传统，特殊场合)"

- 极其重要
- 非常重要
- 一般重要
- 有点重要

○ 完全不重要

Q1246.
试回想您吃香蕉的原因。
您认为以下陈述有多重要？

"...因为它适用于特定场合"

○ 极其重要
○ 非常重要
○ 一般重要
○ 有点重要
○ 完全不重要

Q1247.
试回想您吃香蕉的原因。
您认为以下陈述有多重要？

"...保持均衡饮食"

○ 极其重要
○ 非常重要
○ 一般重要
○ 有点重要
○ 完全不重要

Q1248.
试回想您吃香蕉的原因。
您认为以下陈述有多重要？

"...因为它的社交性"

○ 极其重要
○ 非常重要

- 一般重要
- 有点重要
- 完全不重要

Q1249.
试回想您吃香蕉的原因。
您认为以下陈述有多重要？
"...因为它的价格不贵"

- 极其重要
- 非常重要
- 一般重要
- 有点重要
- 完全不重要

Q1250.
试回想您吃香蕉的原因。
您认为以下陈述有多重要？
"...因为它自然而然地吸引了我（吸引人的颜色)"

- 极其重要
- 非常重要
- 一般重要
- 有点重要
- 完全不重要

Q1251.
试回想您吃香蕉的原因。
您认为以下陈述有多重要？
"...避免让那些试图让我开心的人失望"

- 极其重要
- 非常重要
- 一般重要
- 有点重要
- 完全不重要

Q1252.
试回想您吃香蕉的原因。
您认为以下陈述有多重要？
"...因为它很新潮"

- 极其重要
- 非常重要
- 一般重要
- 有点重要
- 完全不重要

Q1253.
试回想您吃香蕉的原因。
您认为以下陈述有多重要？
"...因为它打折"

- 极其重要
- 非常重要
- 一般重要
- 有点重要
- 完全不重要

Q1254.
试回想您吃香蕉的原因。

您认为以下陈述有多重要？

"...因为它让我保持身材"

- 极其重要
- 非常重要
- 一般重要
- 有点重要
- 完全不重要

Q1255.

试回想您吃香蕉的原因。

您认为以下陈述有多重要？

"...因为这是唯一的选择"

- 极其重要
- 非常重要
- 一般重要
- 有点重要
- 完全不重要

Q1256.

试回想您吃香蕉的原因。

您认为以下陈述有多重要？

"...因为我想每天都吃它"

- 极其重要
- 非常重要
- 一般重要
- 有点重要
- 完全不重要

Q1257.
试回想您不吃香蕉的原因。
您认为以下陈述有多重要？
”我不认为它是一种零食“

- 极其重要
- 非常重要
- 一般重要
- 有点重要
- 完全不重要

Q1258.
试回想您不吃香蕉的原因。
您认为以下陈述有多重要？
”我不喜欢它“

- 极其重要
- 非常重要
- 一般重要
- 有点重要
- 完全不重要

Q1259.
试回想您不吃香蕉的原因。
您认为以下陈述有多重要？
”我不经常吃它“

- 极其重要
- 非常重要
- 一般重要
- 有点重要
- 完全不重要

Q1260.
试回想您不吃香蕉的原因。
您认为以下陈述有多重要？
”它的分量大小不合适“

- 极其重要
- 非常重要
- 一般重要
- 有点重要
- 完全不重要

Q1261.
试回想您不吃香蕉的原因。
您认为以下陈述有多重要？
”它不足以填饱肚子“

- 极其重要
- 非常重要
- 一般重要
- 有点重要
- 完全不重要

Q1262.
试回想您不吃香蕉的原因。
您认为以下陈述有多重要？
”它不健康“

- 极其重要
- 非常重要
- 一般重要
- 有点重要
- 完全不重要

Q1263.

试回想您不吃香蕉的原因。

您认为以下陈述有多重要？

”它不方便“

- 极其重要
- 非常重要
- 一般重要
- 有点重要
- 完全不重要

Q1264.

试回想您不吃香蕉的原因。

您认为以下陈述有多重要？

”我不想纵容自己“

- 极其重要
- 非常重要
- 一般重要
- 有点重要
- 完全不重要

Q1265.

试回想您不吃香蕉的原因。

您认为以下陈述有多重要？

”它不适用于特定场合“

- 极其重要
- 非常重要
- 一般重要
- 有点重要
- 完全不重要

Q1266.
试回想您不吃香蕉的原因。
您认为以下陈述有多重要？
”有其他人在的时候我不吃它“

- 极其重要
- 非常重要
- 一般重要
- 有点重要
- 完全不重要

Q1267.
试回想您不吃香蕉的原因。
您认为以下陈述有多重要？
”价格太高“

- 极其重要
- 非常重要
- 一般重要
- 有点重要
- 完全不重要

Q1268.
试回想您不吃香蕉的原因。
您认为以下陈述有多重要？
”我不喜欢它看起来的样子“

- 极其重要
- 非常重要
- 一般重要
- 有点重要

完全不重要

Q1269.

试回想您不吃香蕉的原因。
您认为以下陈述有多重要？

”它卡路里太高“

- 极其重要
- 非常重要
- 一般重要
- 有点重要
- 完全不重要

Q1270.

试回想您不吃香蕉的原因。
您认为以下陈述有多重要？

”我不应该吃它（例如出于文化、宗教习俗）“

- 极其重要
- 非常重要
- 一般重要
- 有点重要
- 完全不重要

Q1271.

试回想您不吃香蕉的原因。
您认为以下陈述有多重要？

”吃它显得我很土“

- 极其重要
- 非常重要
- 一般重要

- 有点重要
- 完全不重要

Q1272.
试回想您不吃香蕉的原因。
您认为以下陈述有多重要？
”它不是我在那个时刻想吃的东西“

- 极其重要
- 非常重要
- 一般重要
- 有点重要
- 完全不重要

Q1273.
试回想您不吃香蕉的原因。
您认为以下陈述有多重要？
”我最近刚吃过， 我不想太经常吃同样的东西 ”

- 极其重要
- 非常重要
- 一般重要
- 有点重要
- 完全不重要

Q1274.
试回想您不吃香蕉的原因。
您认为以下陈述有多重要？
”我绝不选它。 我喜欢每天吃一样的食物 ”

- 极其重要

- 非常重要
- 一般重要
- 有点重要
- 完全不重要

Q1275.

试回想您不吃香蕉的原因。
您认为以下陈述有多重要？

”这个食物让我感到伤心、孤单或沮丧 ”

- 极其重要
- 非常重要
- 一般重要
- 有点重要
- 完全不重要

Q1276.

试回想您不吃红烧肉的原因。
您认为以下陈述有多重要？

”它不是有机的”

- 极其重要
- 非常重要
- 一般重要
- 有点重要
- 完全不重要

Reasons for Sweet food Choices(Full RATA)

Q1277.

您吃麻球吗?

- 是的， 我吃
- 不是， 我不吃

Q1278.
试回想您吃麻球的原因。
您认为以下陈述有多重要？
”...因为我习惯吃它“

- 极其重要
- 非常重要
- 一般重要
- 有点重要
- 完全不重要

Q1279.
试回想您吃麻球的原因。
您认为以下陈述有多重要？
”...因为它很健康“

- 极其重要
- 非常重要
- 一般重要
- 有点重要
- 完全不重要

Q1280.
试回想您吃麻球的原因。
您认为以下陈述有多重要？
”...因为它准备起来很快“

- 极其重要

- 非常重要
- 一般重要
- 有点重要
- 完全不重要

Q1281.

试回想您吃麻球的原因。

您认为以下陈述有多重要？

”...因为它的味道很好“

- 极其重要
- 非常重要
- 一般重要
- 有点重要
- 完全不重要

Q1282.

试回想您吃麻球的原因。

您认为以下陈述有多重要？

”...为了纵容自己"

- 极其重要
- 非常重要
- 一般重要
- 有点重要
- 完全不重要

Q1283.

试回想您吃麻球的原因。

您认为以下陈述有多重要？

”...因为它是天然的（例如没有经过基因改造)“

- 极其重要
- 非常重要
- 一般重要
- 有点重要
- 完全不重要

Q1284.
试回想您吃麻球的原因。
您认为以下陈述有多重要？
”...因为我不想再花更多的钱“

- 极其重要
- 非常重要
- 一般重要
- 有点重要
- 完全不重要

Q1285.
试回想您吃麻球的原因。
您认为以下陈述有多重要？
”...因为它的卡路里很低“

- 极其重要
- 非常重要
- 一般重要
- 有点重要
- 完全不重要

Q1286.
试回想您吃麻球的原因。

您认为以下陈述有多重要？

”...因为我很沮丧“

- 极其重要
- 非常重要
- 一般重要
- 有点重要
- 完全不重要

Q1287.

试回想您吃麻球的原因。

您认为以下陈述有多重要？

”...因为它让我在别人面前看起来很好“

- 极其重要
- 非常重要
- 一般重要
- 有点重要
- 完全不重要

Q1288.

试回想您吃麻球的原因。

您认为以下陈述有多重要？

”....因为不吃它会不礼貌“

- 极其重要
- 非常重要
- 一般重要
- 有点重要
- 完全不重要

Q1289.
试回想您吃麻球的原因。
您认为以下陈述有多重要？
”....因为它的呈现方式很有吸引力（例如包装)“

- 极其重要
- 非常重要
- 一般重要
- 有点重要
- 完全不重要

Q1290.
试回想您吃麻球的原因。
您认为以下陈述有多重要？
”....这样我就可以和其他人共度时光“

- 极其重要
- 非常重要
- 一般重要
- 有点重要
- 完全不重要

Q1291.
试回想您吃麻球的原因。
您认为以下陈述有多重要？
”....因为它是有机的“

- 极其重要
- 非常重要
- 一般重要
- 有点重要

○ 完全不重要

Q1292.
试回想您吃麻球的原因。
您认为以下陈述有多重要？
”....因为我从小吃到大“

○ 极其重要
○ 非常重要
○ 一般重要
○ 有点重要
○ 完全不重要

Q1293.
试回想您吃麻球的原因。
您认为以下陈述有多重要？
"...因为吃它让我很享受“

○ 极其重要
○ 非常重要
○ 一般重要
○ 有点重要
○ 完全不重要

Q1294.
试回想您吃麻球的原因。
您认为以下陈述有多重要？
"...因为我饿了“

○ 极其重要
○ 非常重要

- 一般重要
- 有点重要
- 完全不重要

Q1295.
试回想您吃麻球的原因。
您认为以下陈述有多重要？
"...因为我经常吃它“

- 极其重要
- 非常重要
- 一般重要
- 有点重要
- 完全不重要

Q1296.
试回想您吃麻球的原因。
您认为以下陈述有多重要？
"...因为我对它有食欲"

- 极其重要
- 非常重要
- 一般重要
- 有点重要
- 完全不重要

Q1297.
试回想您吃麻球的原因。
您认为以下陈述有多重要？
"...因为它很容易准备“

- 极其重要
- 非常重要
- 一般重要
- 有点重要
- 完全不重要

Q1298.

试回想您吃麻球的原因。
您认为以下陈述有多重要？

"...因为我很伤心"

- 极其重要
- 非常重要
- 一般重要
- 有点重要
- 完全不重要

Q1299.

试回想您吃麻球的原因。
您认为以下陈述有多重要？

"...因为脂肪含量低"

- 极其重要
- 非常重要
- 一般重要
- 有点重要
- 完全不重要

Q1300.

试回想您吃麻球的原因。

您认为以下陈述有多重要？

"...因为我从广告或在电视上看过它"

- 极其重要
- 非常重要
- 一般重要
- 有点重要
- 完全不重要

Q1301.

试回想您吃麻球的原因。

您认为以下陈述有多重要？

"...因为它使社交聚会更加舒适"

- 极其重要
- 非常重要
- 一般重要
- 有点重要
- 完全不重要

Q1302.

试回想您吃麻球的原因。

您认为以下陈述有多重要？

"...因为我应该吃它"

- 极其重要
- 非常重要
- 一般重要
- 有点重要
- 完全不重要

Q1303.
试回想您吃麻球的原因。
您认为以下陈述有多重要？

"...因为其他人喜欢它"

- 极其重要
- 非常重要
- 一般重要
- 有点重要
- 完全不重要

Q1304.
试回想您吃麻球的原因。
您认为以下陈述有多重要？

"...因为我感到孤独"

- 极其重要
- 非常重要
- 一般重要
- 有点重要
- 完全不重要

Q1305.
试回想您吃麻球的原因。
您认为以下陈述有多重要？

"...因为我关注我的体重"

- 极其重要
- 非常重要
- 一般重要
- 有点重要

完全不重要

Q1306.

试回想您吃麻球的原因。

您认为以下陈述有多重要？

"...因为它不含有害物质（例如杀虫剂，污染物，抗生素)"

- 极其重要
- 非常重要
- 一般重要
- 有点重要
- 完全不重要

Q1307.

试回想您吃麻球的原因。

您认为以下陈述有多重要？

"...因为它最方便"

- 极其重要
- 非常重要
- 一般重要
- 有点重要
- 完全不重要

Q1308.

试回想您吃麻球的原因。

您认为以下陈述有多重要？

"...因为我需要能量"

- 极其重要
- 非常重要

- 一般重要
- 有点重要
- 完全不重要

Q1309.
试回想您吃麻球的原因。
您认为以下陈述有多重要？
"...因为我熟悉它"

- 极其重要
- 非常重要
- 一般重要
- 有点重要
- 完全不重要

Q1310.
试回想您吃麻球的原因。
您认为以下陈述有多重要？
"...因为我喜欢它"

- 极其重要
- 非常重要
- 一般重要
- 有点重要
- 完全不重要

Q1311.
试回想您吃麻球的原因。
您认为以下陈述有多重要？
"...因为它能令我感到愉悦的饱腹感"

- 极其重要
- 非常重要
- 一般重要
- 有点重要
- 完全不重要

Q1312.

试回想您吃麻球的原因。
您认为以下陈述有多重要？

"...为了奖励自己"

- 极其重要
- 非常重要
- 一般重要
- 有点重要
- 完全不重要

Q1313.

试回想您吃麻球的原因。
您认为以下陈述有多重要？

"...出于传统（例如家庭传统，特殊场合)"

- 极其重要
- 非常重要
- 一般重要
- 有点重要
- 完全不重要

Q1314.

试回想您吃麻球的原因。

您认为以下陈述有多重要？

"...因为它适用于特定场合"

- 极其重要
- 非常重要
- 一般重要
- 有点重要
- 完全不重要

Q1315.

试回想您吃麻球的原因。

您认为以下陈述有多重要？

"...保持均衡饮食"

- 极其重要
- 非常重要
- 一般重要
- 有点重要
- 完全不重要

Q1316.

试回想您吃麻球的原因。

您认为以下陈述有多重要？

"...因为它的社交性"

- 极其重要
- 非常重要
- 一般重要
- 有点重要
- 完全不重要

Q1317.
试回想您吃麻球的原因。
您认为以下陈述有多重要？

"...因为它的价格不贵"

- 极其重要
- 非常重要
- 一般重要
- 有点重要
- 完全不重要

Q1318.
试回想您吃麻球的原因。
您认为以下陈述有多重要？

"...因为它自然而然地吸引了我（吸引人的颜色)"

- 极其重要
- 非常重要
- 一般重要
- 有点重要
- 完全不重要

Q1319.
试回想您吃麻球的原因。
您认为以下陈述有多重要？

"...避免让那些试图让我开心的人失望"

- 极其重要
- 非常重要
- 一般重要
- 有点重要

- 完全不重要

Q1320.
试回想您吃麻球的原因。
您认为以下陈述有多重要？

"...因为它很新潮"

- 极其重要
- 非常重要
- 一般重要
- 有点重要
- 完全不重要

Q1321.
试回想您吃麻球的原因。
您认为以下陈述有多重要？

"...因为它打折"

- 极其重要
- 非常重要
- 一般重要
- 有点重要
- 完全不重要

Q1322.
试回想您吃麻球的原因。
您认为以下陈述有多重要？

"...因为它让我保持身材"

- 极其重要
- 非常重要

- 一般重要
- 有点重要
- 完全不重要

Q1323.
试回想您吃麻球的原因。
您认为以下陈述有多重要？
"...因为这是唯一的选择"

- 极其重要
- 非常重要
- 一般重要
- 有点重要
- 完全不重要

Q1324.
试回想您吃麻球的原因。
您认为以下陈述有多重要？
"...因为我想每天都吃它"

- 极其重要
- 非常重要
- 一般重要
- 有点重要
- 完全不重要

Q1325.
试回想您不吃麻球的原因。
您认为以下陈述有多重要？
”我不认为它是一种零食“

- 极其重要
- 非常重要
- 一般重要
- 有点重要
- 完全不重要

Q1326.
试回想您不吃麻球的原因。
您认为以下陈述有多重要？
”我不喜欢它“

- 极其重要
- 非常重要
- 一般重要
- 有点重要
- 完全不重要

Q1327.
试回想您不吃麻球的原因。
您认为以下陈述有多重要？
”我不经常吃它“

- 极其重要
- 非常重要
- 一般重要
- 有点重要
- 完全不重要

Q1328.
试回想您不吃麻球的原因。
您认为以下陈述有多重要？
”它的分量大小不合适“

- 极其重要
- 非常重要
- 一般重要
- 有点重要
- 完全不重要

Q1329.
试回想您不吃麻球的原因。
您认为以下陈述有多重要？
”它不足以填饱肚子“

- 极其重要
- 非常重要
- 一般重要
- 有点重要
- 完全不重要

Q1330.
试回想您不吃麻球的原因。
您认为以下陈述有多重要？
”它不健康“

- 极其重要
- 非常重要
- 一般重要
- 有点重要
- 完全不重要

Q1331.
试回想您不吃麻球的原因。
您认为以下陈述有多重要？

”它不方便“

- 极其重要
- 非常重要
- 一般重要
- 有点重要
- 完全不重要

Q1332.

试回想您不吃麻球的原因。
您认为以下陈述有多重要？
”我不想纵容自己“

- 极其重要
- 非常重要
- 一般重要
- 有点重要
- 完全不重要

Q1333.

试回想您不吃麻球的原因。
您认为以下陈述有多重要？
”它不适用于特定场合“

- 极其重要
- 非常重要
- 一般重要
- 有点重要
- 完全不重要

Q1334.

试回想您不吃麻球的原因。

您认为以下陈述有多重要？
”有其他人在的时候我不吃它“

- 极其重要
- 非常重要
- 一般重要
- 有点重要
- 完全不重要

Q1335.
试回想您不吃麻球的原因。
您认为以下陈述有多重要？
”价格太高“

- 极其重要
- 非常重要
- 一般重要
- 有点重要
- 完全不重要

Q1336.
试回想您不吃麻球的原因。
您认为以下陈述有多重要？
”我不喜欢它看起来的样子“

- 极其重要
- 非常重要
- 一般重要
- 有点重要
- 完全不重要

Q1337.

试回想您不吃麻球的原因。
您认为以下陈述有多重要？
”它卡路里太高“

- 极其重要
- 非常重要
- 一般重要
- 有点重要
- 完全不重要

Q1338.
试回想您不吃麻球的原因。
您认为以下陈述有多重要？
”我不应该吃它（例如出于文化、宗教习俗）“

- 极其重要
- 非常重要
- 一般重要
- 有点重要
- 完全不重要

Q1339.
试回想您不吃麻球的原因。
您认为以下陈述有多重要？
”吃它显得我很土“

- 极其重要
- 非常重要
- 一般重要
- 有点重要
- 完全不重要

Q1340.
试回想您不吃麻球的原因。
您认为以下陈述有多重要？
"它不是我在那个时刻想吃的东西"

- 极其重要
- 非常重要
- 一般重要
- 有点重要
- 完全不重要

Q1341.
试回想您不吃麻球的原因。
您认为以下陈述有多重要？
"我最近刚吃过， 我不想太经常吃同样的东西 "

- 极其重要
- 非常重要
- 一般重要
- 有点重要
- 完全不重要

Q1342.
试回想您不吃麻球的原因。
您认为以下陈述有多重要？
"我绝不选它。 我喜欢每天吃一样的食物 "

- 极其重要
- 非常重要
- 一般重要
- 有点重要
- 完全不重要

Q1343.
试回想您不吃豆浆的原因。
您认为以下陈述有多重要？

”这个食物让我感到伤心、孤单或沮丧 ”

- 极其重要
- 非常重要
- 一般重要
- 有点重要
- 完全不重要

Q1344.
试回想您不吃红烧肉的原因。
您认为以下陈述有多重要？

”它不是有机的”

- 极其重要
- 非常重要
- 一般重要
- 有点重要
- 完全不重要

Powered by Qualtrics
