# Supplementary material for: Comparing Four Question Formats in Five Languages for On-Line Consumer Surveys
Source: Methods Protoc. 2020 Jul 14;3(3):49. doi: 10.3390/mps3030049 (PMC7563457; doi:10.3390/mps3030049)
Supplement: Supplementary file 1 [file mps-03-00049-s001.zip › supplementary/Figure S4 (Spain-Spanish).pdf]

Demographics

¿Cuál es su género?

- Hombre
- Mujer

¿En qué año nació?

- 1943 o antes
- 1944 - 1964
- 1965 - 1979
- 1980 -1994
- 1995 - 2001
- 2002 o después

¿Cuál es su nivel de estudios (tipo de estudio más avanzado que ha completado)?

- Primarios (educación primaria)
- Secundarios (ESO y/o bachillerato)
- Universitarios

¿Cuántos adultos viven en su hogar, incluyéndose usted?

- 0
- 1

- 2
- 3
- 4
- 5 +

¿Cuántos niños menores de edad (<18) viven en su hogar?

- 0
- 1
- 2
- 3
- 4 +

Nos preocupamos por la calidad de nuestras encuestas y esperamos recibir respuestas muy precisas con respecto a sus opiniones, es por eso que es muy importante para nosotros que responda de la mejor manera a cada una de las preguntas en esta encuesta.

¿Se comprometería a brindar respuestas honestas y serias a cada pregunta de la encuesta?

- Proporcionare mi mejor respuesta
- No proporcionare mi mejor respuesta
- No me puedo comprometer

Food Involvement

El cuestionario que está a punto de realizar esta relacionado con sus hábitos alimentarios

Por favor, indique si está de acuerdo con las siguientes preguntas/afirmaciones:

No pienso mucho en comida cada día.

- Totalmente en desacuerdo
- En desacuerdo
- Ligeramente en desacuerdo
- Ni de acuerdo ni en desacuerdo
- Ligeramente de acuerdo
- De acuerdo
- Totalmente de acuerdo

Cocinar no es muy divertido.

- Totalmente en desacuerdo
- En desacuerdo
- Ligeramente en desacuerdo
- Ni de acuerdo ni en desacuerdo
- Ligeramente de acuerdo
- De acuerdo
- Totalmente de acuerdo

Me gusta hablar sobre lo que comí o voy a comer.

- Totalmente en desacuerdo
- En desacuerdo
- Ligeramente en desacuerdo
- Ni de acuerdo ni en desacuerdo
- Ligeramente de acuerdo
- De acuerdo
- Totalmente de acuerdo

Comparado con otras decisiones diarias, mis preferencias alimentarias no son muy importantes.

- Totalmente en desacuerdo
- En desacuerdo
- Ligeramente en desacuerdo
- Ni de acuerdo ni en desacuerdo
- Ligeramente de acuerdo
- De acuerdo
- Totalmente de acuerdo

Cuando viajo, una de las cosas que preparo con más antelación es lo que voy a comer allí.

- Totalmente en desacuerdo
- En desacuerdo
- Ligeramente en desacuerdo
- Ni de acuerdo ni en desacuerdo
- Ligeramente de acuerdo
- De acuerdo
- Totalmente de acuerdo

Hago la mayor parte de la limpieza después de comer.

- Totalmente en desacuerdo
- En desacuerdo
- Ligeramente en desacuerdo
- Ni de acuerdo ni en desacuerdo
- Ligeramente de acuerdo
- De acuerdo
- Totalmente de acuerdo

Disfruto cocinando para otros y para mí.

- Totalmente en desacuerdo
- En desacuerdo

- Ligeramente en desacuerdo
- Ni de acuerdo ni en desacuerdo
- Ligeramente de acuerdo
- De acuerdo
- Totalmente de acuerdo

Cuando como fuera de casa, no pienso ni hablo mucho acerca de cómo sabe la comida.

- Totalmente en desacuerdo
- En desacuerdo
- Ligeramente en desacuerdo
- Ni de acuerdo ni en desacuerdo
- Ligeramente de acuerdo
- De acuerdo
- Totalmente de acuerdo

No me gusta mezclar o trocear los alimentos.

- Totalmente en desacuerdo
- En desacuerdo
- Ligeramente en desacuerdo
- Ni de acuerdo ni en desacuerdo
- Ligeramente de acuerdo
- De acuerdo
- Totalmente de acuerdo

Hago la mayor parte o toda mi compra de alimentos.

- Totalmente en desacuerdo
- En desacuerdo
- Ligeramente en desacuerdo

- Ni de acuerdo ni en desacuerdo
- Ligeramente de acuerdo
- De acuerdo
- Totalmente de acuerdo

No lavo los platos ni limpio la mesa.

- Totalmente en desacuerdo
- En desacuerdo
- Ligeramente en desacuerdo
- Ni de acuerdo ni en desacuerdo
- Ligeramente de acuerdo
- De acuerdo
- Totalmente de acuerdo

Me preocupa si la mesa está bien montada o no.

- Totalmente en desacuerdo
- En desacuerdo
- Ligeramente en desacuerdo
- Ni de acuerdo ni en desacuerdo
- Ligeramente de acuerdo
- De acuerdo
- Totalmente de acuerdo

Health and Taste Attitudes

Soy muy particular acerca de qué tan saludable es mi comida.

- Totalmente en desacuerdo
- En desacuerdo

- Ligeramente en desacuerdo
- Ni de acuerdo ni en desacuerdo
- Ligeramente de acuerdo
- De acuerdo
- Totalmente de acuerdo

Siempre sigo una dieta saludable y equilibrada.

- Totalmente en desacuerdo
- En desacuerdo
- Ligeramente en desacuerdo
- Ni de acuerdo ni en desacuerdo
- Ligeramente de acuerdo
- De acuerdo
- Totalmente de acuerdo

Es importante para mí que mi dieta sea baja en grasas.

- Totalmente en desacuerdo
- En desacuerdo
- Ligeramente en desacuerdo
- Ni de acuerdo ni en desacuerdo
- Ligeramente de acuerdo
- De acuerdo
- Totalmente de acuerdo

Es importante para mí que mi dieta contenga muchas vitaminas y minerales.

- Totalmente en desacuerdo
- En desacuerdo
- Ligeramente en desacuerdo
- Ni de acuerdo ni en desacuerdo

- Ligeramente de acuerdo
- De acuerdo
- Totalmente de acuerdo

Como lo que me gusta y no me preocupa si la comida es saludable.

- Totalmente en desacuerdo
- En desacuerdo
- Ligeramente en desacuerdo
- Ni de acuerdo ni en desacuerdo
- Ligeramente de acuerdo
- De acuerdo
- Totalmente de acuerdo

No evito ningún tipo de alimentos, incluso aunque puedan elevar mi colesterol.

- Totalmente en desacuerdo
- En desacuerdo
- Ligeramente en desacuerdo
- Ni de acuerdo ni en desacuerdo
- Ligeramente de acuerdo
- De acuerdo
- Totalmente de acuerdo

Los alimentos saludables no afectan mi elección de alimentos.

- Totalmente en desacuerdo
- En desacuerdo
- Ligeramente en desacuerdo
- Ni de acuerdo ni en desacuerdo
- Ligeramente de acuerdo
- De acuerdo

- Totalmente de acuerdo

No me influye en absoluto si los aperitivos/picoteos/snacks son saludables.

- Totalmente en desacuerdo
- En desacuerdo
- Ligeramente en desacuerdo
- Ni de acuerdo ni en desacuerdo
- Ligeramente de acuerdo
- De acuerdo
- Totalmente de acuerdo

No creo que la comida deba ser siempre placentera.

- Totalmente en desacuerdo
- En desacuerdo
- Ligeramente en desacuerdo
- Ni de acuerdo ni en desacuerdo
- Ligeramente de acuerdo
- De acuerdo
- Totalmente de acuerdo

No me afecta en absoluto la apariencia de los alimentos

- Totalmente en desacuerdo
- En desacuerdo
- Ligeramente en desacuerdo
- Ni de acuerdo ni en desacuerdo
- Ligeramente de acuerdo
- De acuerdo
- Totalmente de acuerdo

Para mí es importante comer alimentos deliciosos tanto entre semana como los fines de semana.

- Totalmente en desacuerdo
- En desacuerdo
- Ligeramente en desacuerdo
- Ni de acuerdo ni en desacuerdo
- Ligeramente de acuerdo
- De acuerdo
- Totalmente de acuerdo

Cuando como, me concentro en disfrutar el sabor de los alimentos.

- Totalmente en desacuerdo
- En desacuerdo
- Ligeramente en desacuerdo
- Ni de acuerdo ni en desacuerdo
- Ligeramente de acuerdo
- De acuerdo
- Totalmente de acuerdo

Termino mi comida, aunque no me guste su sabor.

- Totalmente en desacuerdo
- En desacuerdo
- Ligeramente en desacuerdo
- Ni de acuerdo ni en desacuerdo
- Ligeramente de acuerdo
- De acuerdo
- Totalmente de acuerdo

Una parte esencial de mi fin de semana es comer alimentos deliciosos.

- Totalmente en desacuerdo
- En desacuerdo
- Ligeramente en desacuerdo
- Ni de acuerdo ni en desacuerdo
- Ligeramente de acuerdo
- De acuerdo
- Totalmente de acuerdo

Neophobia

Estoy constantemente probando comida nueva y diferente.

- Totalmente en desacuerdo
- En desacuerdo
- Ligeramente en desacuerdo
- Ni de acuerdo ni en desacuerdo
- Ligeramente de acuerdo
- De acuerdo
- Totalmente de acuerdo

No confío en los alimentos nuevos.

- Totalmente en desacuerdo
- En desacuerdo
- Ligeramente en desacuerdo
- Ni de acuerdo ni en desacuerdo
- Ligeramente de acuerdo
- De acuerdo
- Totalmente de acuerdo

Si no sé lo que contiene un alimento, no lo probaré.

- Totalmente en desacuerdo
- En desacuerdo
- Ligeramente en desacuerdo
- Ni de acuerdo ni en desacuerdo
- Ligeramente de acuerdo
- De acuerdo
- Totalmente de acuerdo

Me gustan los alimentos de otros países.

- Totalmente en desacuerdo
- En desacuerdo
- Ligeramente en desacuerdo
- Ni de acuerdo ni en desacuerdo
- Ligeramente de acuerdo
- De acuerdo
- Totalmente de acuerdo

La comida étnica me parece demasiado extraña para comerla.

- Totalmente en desacuerdo
- En desacuerdo
- Ligeramente en desacuerdo
- Ni de acuerdo ni en desacuerdo
- Ligeramente de acuerdo
- De acuerdo
- Totalmente de acuerdo

En las fiestas, pruebo alimentos nuevos.

- Totalmente en desacuerdo
- En desacuerdo
- Ligeramente en desacuerdo
- Ni de acuerdo ni en desacuerdo
- Ligeramente de acuerdo
- De acuerdo
- Totalmente de acuerdo

Me preocupa comer cosas que no he probado antes.

- Totalmente en desacuerdo
- En desacuerdo
- Ligeramente en desacuerdo
- Ni de acuerdo ni en desacuerdo
- Ligeramente de acuerdo
- De acuerdo
- Totalmente de acuerdo

Soy muy particular con los alimentos que como.

- Totalmente en desacuerdo
- En desacuerdo
- Ligeramente en desacuerdo
- Ni de acuerdo ni en desacuerdo
- Ligeramente de acuerdo
- De acuerdo
- Totalmente de acuerdo

Comería casi cualquier cosa.

- Totalmente en desacuerdo
- En desacuerdo
- Ligeramente en desacuerdo
- Ni de acuerdo ni en desacuerdo
- Ligeramente de acuerdo
- De acuerdo
- Totalmente de acuerdo

Me gusta probar nuevos restaurantes étnicos.

- Totalmente en desacuerdo
- En desacuerdo
- Ligeramente en desacuerdo
- Ni de acuerdo ni en desacuerdo
- Ligeramente de acuerdo
- De acuerdo
- Totalmente de acuerdo

Reasons for Starchy food choices(CATA)

¿Come usted paella?

- Sí
- No

¿Cuáles fueron las razones por las que consume paella? (Marque todas las opciones que apliquen)

- ... para poder pasar tiempo con otras personas y socializar
- ... porque sabe bien
- ... porque crecí con ello

- ☐ ... porque sería descortés no comerlo
- ☐ ... porque es lo más conveniente
- ☐ ... porque es orgánico
- ☐ ... porque me hace quedar bien delante de los demás
- ☐ ... porque hace más agradables las reuniones sociales
- ☐ ... porque me mantiene en forma (por ejemplo, energético, motivado)
- ☐ ... porque está en rebaja
- ☐ ... porque me siento frustrado
- ☐ ... porque tengo hambre
- ☐ ... porque es barato
- ☐ ... porque estoy familiarizado con ello
- ☐ ... para mantener una dieta equilibrada
- ☐ ... es parte de mis tradiciones (por ejemplo, tradiciones familiares, ocasiones especiales)
- ☐ ... porque me gusta
- ☐ ... porque esa era la única opción
- ☐ ... porque estoy acostumbrado a comerlo
- ☐ ... para complacerme
- ☐ ... porque lo disfruto
- ☐ ... porque es rápido de preparar
- ☐ ... porque vigilo mi peso
- ☐ ... porque no contiene sustancias peligrosas (por ejemplo, pesticidas, contaminantes, o antibióticos
- ☐ ... porque se consume en ciertas ocasiones
- ☐ ...porque está de moda
- ☐ ... porque no quiero gastar más dinero
- ☐ ... porque me apetece
- ☐ ... porque me apetece de modo espontáneo (por ejemplo, colores llamativos a la vista)
- ☐ ... porque me siento triste
- ☐ ... para evitar decepcionar a alguien que está tratando de hacerme feliz
- ☐ ... porque es bajo en calorías
- ☐ ... porque es fácil de preparar

- ☐ ... porque la presentación es atractiva (por ejemplo, el empaque)
- ☐ ... porque usualmente lo como
- ☐ ... porque lo he visto en anuncios o en la televisión
- ☐ ... porque se supone que debo comerlo
- ☐ ... porque es natural (por ejemplo, no modificado genéticamente)
- ☐ ... porque quiero comerlo todos los días
- ☐ ... porque me siento solo
- ☐ ... porque es social
- ☐ ... porque me llena de un modo agradable
- ☐ ... porque necesito energía
- ☐ ... porque es saludable
- ☐ ...porque es bajo en grasa
- ☐ ... porque le gusta a otros
- ☐ ... para recompensarme

Por favor indíquenos las razones por las que usted no consume paella (Marque todas las opciones que apliquen)

- ☐ No me gusta
- ☐ Se supone que no debo consumirlo
- ☐ Nunca lo escogería. Me gusta consumir el mismo alimento todos los días
- ☐ No es saludable
- ☐ Es muy alto en calorías
- ☐ No lo considero un alimento que llene lo suficiente
- ☐ Me hace sentir triste, solitario, o frustrado
- ☐ No quise consentirme
- ☐ No lo consumo usualmente
- ☐ El tamaño de la porción no era adecuado
- ☐ Lo consumí recientemente y no quiero consumir el mismo alimento muy seguido
- ☐ No era apropiado para la ocasión
- ☐ No lo considero como un aperitivo/snack
- ☐ No es un tipo de comida que consumo en presencia de otras personas
- ☐ El precio era muy elevado

- ☐ No era algo que se me apetecía en ese momento
- ☐ No es orgánico
- ☐ Mi generación no consume este tipo de alimento
- ☐ No es práctico de preparar
- ☐ No me gustó la apariencia

Reasons for Protein foods Choices(CATA)

¿Come usted jamón serrano?

- ○ Sí
- ○ No

¿Cuáles fueron las razones por las que consume jamón serrano? (Marque todas las opciones que apliquen)

- ☐ ... porque quiero comerlo todos los días
- ☐ ... porque lo he visto en anuncios o en la televisión
- ☐ ... porque me apetece de modo espontáneo (por ejemplo, colores llamativos a la vista)
- ☐ ... porque se supone que debo comerlo
- ☐ ... porque se consume en ciertas ocasiones
- ☐ ... porque me siento triste
- ☐ ... porque es lo más conveniente
- ☐ ... porque me mantiene en forma (por ejemplo, energético, motivado)
- ☐ ... porque está en rebaja
- ☐ ... porque sabe bien
- ☐ ... porque es bajo en calorías
- ☐ ... porque es social
- ☐ ... porque le gusta a otros
- ☐ ... porque me siento solo

- ☐ ... para recompensarme
- ☐ ... porque lo disfruto
- ☐ ... porque me siento frustrado
- ☐ ... para evitar decepcionar a alguien que está tratando de hacerme feliz
- ☐ ... porque me hace quedar bien delante de los demás
- ☐ ... porque estoy familiarizado con ello
- ☐ ...porque es bajo en grasa
- ☐ ... porque estoy acostumbrado a comerlo
- ☐ ... para mantener una dieta equilibrada
- ☐ ... porque no contiene sustancias peligrosas (por ejemplo, pesticidas, contaminantes, o antibióticos
- ☐ ... para complacerme
- ☐ ... porque crecí con ello
- ☐ ... porque es saludable
- ☐ ... porque es barato
- ☐ ... porque no quiero gastar más dinero
- ☐ ... porque sería descortés no comerlo
- ☐ ... es parte de mis tradiciones (por ejemplo, tradiciones familiares, ocasiones especiales)
- ☐ ... porque tengo hambre
- ☐ ...porque está de moda
- ☐ ... porque usualmente lo como
- ☐ ... porque hace más agradables las reuniones sociales
- ☐ ... porque vigilo mi peso
- ☐ ... porque es orgánico
- ☐ ... porque me gusta
- ☐ ... porque necesito energía
- ☐ ... porque me llena de un modo agradable
- ☐ ... para poder pasar tiempo con otras personas y socializar
- ☐ ... porque la presentación es atractiva (por ejemplo, el empaque)
- ☐ ... porque me apetece
- ☐ ... porque es rápido de preparar
- ☐ ... porque es fácil de preparar

- [ ] ... porque es natural (por ejemplo, no modificado genéticamente)
- [ ] ... porque esa era la única opción

Por favor indíquenos las razones por las que usted no consume jamón serrano (Marque todas las opciones que apliquen)

- [ ] No era algo que se me apetecía en ese momento
- [ ] No era apropiado para la ocasión
- [ ] Es muy alto en calorías
- [ ] No es saludable
- [ ] El tamaño de la porción no era adecuado
- [ ] No lo consumo usualmente
- [ ] No lo considero como un aperitivo/snack
- [ ] No es orgánico
- [ ] Mi generación no consume este tipo de alimento
- [ ] No quise consentirme
- [ ] No me gustó la apariencia
- [ ] No es un tipo de comida que consumo en presencia de otras personas
- [ ] No me gusta
- [ ] Lo consumí recientemente y no quiero consumir el mismo alimento muy seguido
- [ ] No lo considero un alimento que llene lo suficiente
- [ ] Nunca lo escogería. Me gusta consumir el mismo alimento todos los días
- [ ] El precio era muy elevado
- [ ] Me hace sentir triste, solitario, o frustrado
- [ ] Se supone que no debo consumirlo
- [ ] No es práctico de preparar

Reasons for Dairy Food Choices(CATA)

¿Come usted leche?

- ○ Sí

- [ ] No

¿Cuáles fueron las razones por las que consume leche? (Marque todas las opciones que apliquen)

- [ ] ... porque me siento solo
- [ ] ...porque es bajo en grasa
- [ ] ... porque esa era la única opción
- [ ] ... porque no contiene sustancias peligrosas (por ejemplo, pesticidas, contaminantes, o antibióticos
- [ ] ... porque quiero comerlo todos los días
- [ ] ... porque está en rebaja
- [ ] ... porque es bajo en calorías
- [ ] ... porque es barato
- [ ] ... porque sabe bien
- [ ] ... para mantener una dieta equilibrada
- [ ] ... porque hace más agradables las reuniones sociales
- [ ] ... porque le gusta a otros
- [ ] ... porque me siento frustrado
- [ ] ... porque necesito energía
- [ ] ... porque usualmente lo como
- [ ] ... porque lo he visto en anuncios o en la televisión
- [ ] ... porque la presentación es atractiva (por ejemplo, el empaque)
- [ ] ... porque no quiero gastar más dinero
- [ ] ... es parte de mis tradiciones (por ejemplo, tradiciones familiares, ocasiones especiales)
- [ ] ... porque lo disfruto
- [ ] ... porque es rápido de preparar
- [ ] ... porque crecí con ello
- [ ] ... porque me apetece de modo espontáneo (por ejemplo, colores llamativos a la vista)
- [ ] ... porque tengo hambre

- ☐ ... porque me llena de un modo agradable
- ☐ ... porque vigilo mi peso
- ☐ ... porque es fácil de preparar
- ☐ ... porque estoy familiarizado con ello
- ☐ ... porque me mantiene en forma (por ejemplo, energético, motivado)
- ☐ ... porque me siento triste
- ☐ ... porque me apetece
- ☐ ... porque es orgánico
- ☐ ... porque me hace quedar bien delante de los demás
- ☐ ... porque me gusta
- ☐ ... porque es lo más conveniente
- ☐ ...porque está de moda
- ☐ ... para poder pasar tiempo con otras personas y socializar
- ☐ ... porque se supone que debo comerlo
- ☐ ... porque es saludable
- ☐ ... porque es natural (por ejemplo, no modificado genéticamente)
- ☐ ... porque estoy acostumbrado a comerlo
- ☐ ... porque es social
- ☐ ... para recompensarme
- ☐ ... para complacerme
- ☐ ... porque se consume en ciertas ocasiones
- ☐ ... para evitar decepcionar a alguien que está tratando de hacerme feliz
- ☐ ... porque sería descortés no comerlo

Por favor indíquenos las razones por las que usted no consume leche (Marque todas las opciones que apliquen)

- ☐ Lo consumí recientemente y no quiero consumir el mismo alimento muy seguido
- ☐ No lo considero como un aperitivo/snack
- ☐ No quise consentirme
- ☐ Mi generación no consume este tipo de alimento
- ☐ No lo considero un alimento que llene lo suficiente
- ☐ El tamaño de la porción no era adecuado

- ☐ No me gustó la apariencia
- ☐ Es muy alto en calorías
- ☐ El precio era muy elevado
- ☐ No es práctico de preparar
- ☐ No lo consumo usualmente
- ☐ Nunca lo escogería. Me gusta consumir el mismo alimento todos los días
- ☐ No es saludable
- ☐ Se supone que no debo consumirlo
- ☐ No es orgánico
- ☐ No me gusta
- ☐ No era algo que se me apetecía en ese momento
- ☐ No era apropiado para la ocasión
- ☐ No es un tipo de comida que consumo en presencia de otras personas
- ☐ Me hace sentir triste, solitario, o frustrado

Reasons for Fruit Choices(CATA)

¿Come usted plátano?

- ○ Sí
- ○ No

¿Cuáles fueron las razones por las que consume plátano? (Marque todas las opciones que apliquen)

- ☐ ... porque lo he visto en anuncios o en la televisión
- ☐ ... porque necesito energía
- ☐ ... porque me apetece
- ☐ ... es parte de mis tradiciones (por ejemplo, tradiciones familiares, ocasiones especiales)
- ☐ ... para complacerme

- [ ] ... porque tengo hambre
- [ ] ... porque sabe bien
- [ ] ... porque es rápido de preparar
- [ ] ... porque hace más agradables las reuniones sociales
- [ ] ... porque crecí con ello
- [ ] ... porque me mantiene en forma (por ejemplo, energético, motivado)
- [ ] ... porque me llena de un modo agradable
- [ ] ... porque es orgánico
- [ ] ...porque está de moda
- [ ] ... porque es bajo en calorías
- [ ] ... para recompensarme
- [ ] ... para poder pasar tiempo con otras personas y socializar
- [ ] ... porque estoy acostumbrado a comerlo
- [ ] ... porque es barato
- [ ] ... porque esa era la única opción
- [ ] ... porque no contiene sustancias peligrosas (por ejemplo, pesticidas, contaminantes, o antibióticos
- [ ] ...porque es bajo en grasa
- [ ] ... para mantener una dieta equilibrada
- [ ] ... porque es fácil de preparar
- [ ] ... porque es saludable
- [ ] ... porque me siento triste
- [ ] ... porque sería descortés no comerlo
- [ ] ... porque le gusta a otros
- [ ] ... porque está en rebaja
- [ ] ... porque se supone que debo comerlo
- [ ] ... porque es social
- [ ] ... porque es natural (por ejemplo, no modificado genéticamente)
- [ ] ... porque usualmente lo como
- [ ] ... porque vigilo mi peso
- [ ] ... porque lo disfruto
- [ ] ... porque me siento solo
- [ ] ... porque estoy familiarizado con ello

- ☐ ... porque quiero comerlo todos los días
- ☐ ... porque no quiero gastar más dinero
- ☐ ... porque es lo más conveniente
- ☐ ... porque me gusta
- ☐ ... porque me hace quedar bien delante de los demás
- ☐ ... porque me siento frustrado
- ☐ ... porque me apetece de modo espontáneo (por ejemplo, colores llamativos a la vista)
- ☐ ... para evitar decepcionar a alguien que está tratando de hacerme feliz
- ☐ ... porque la presentación es atractiva (por ejemplo, el empaque)
- ☐ ... porque se consume en ciertas ocasiones

Por favor indíquenos las razones por las que usted no consume plátano (Marque todas las opciones que apliquen)

- ☐ El tamaño de la porción no era adecuado
- ☐ Me hace sentir triste, solitario, o frustrado
- ☐ No lo considero un alimento que llene lo suficiente
- ☐ No era algo que se me apetecía en ese momento
- ☐ No me gustó la apariencia
- ☐ El precio era muy elevado
- ☐ No lo consumo usualmente
- ☐ Lo consumí recientemente y no quiero consumir el mismo alimento muy seguido
- ☐ Nunca lo escogería. Me gusta consumir el mismo alimento todos los días
- ☐ No quise consentirme
- ☐ No es práctico de preparar
- ☐ Es muy alto en calorías
- ☐ No es orgánico
- ☐ Se supone que no debo consumirlo
- ☐ No lo considero como un aperitivo/snack
- ☐ Mi generación no consume este tipo de alimento
- ☐ No es saludable

- ☐ No me gusta
- ☐ No era apropiado para la ocasión
- ☐ No es un tipo de comida que consumo en presencia de otras personas

Reasons for Sweet food Choices(CATA)

¿Come usted turrón?

- ○ Sí
- ○ No

¿Cuáles fueron las razones por las que consume turrón? (Marque todas las opciones que apliquen)

- ☐ ... porque estoy familiarizado con ello
- ☐ ... para recompensarme
- ☐ ... porque sería descortés no comerlo
- ☐ ... porque se supone que debo comerlo
- ☐ ... porque me gusta
- ☐ ... porque la presentación es atractiva (por ejemplo, el empaque)
- ☐ ... porque le gusta a otros
- ☐ ... porque me siento triste
- ☐ ... porque tengo hambre
- ☐ ... porque está en rebaja
- ☐ ... porque no quiero gastar más dinero
- ☐ ... porque usualmente lo como
- ☐ ... para poder pasar tiempo con otras personas y socializar
- ☐ ... porque me hace quedar bien delante de los demás
- ☐ ... porque lo he visto en anuncios o en la televisión
- ☐ ... porque es bajo en calorías
- ☐ ...porque es bajo en grasa

- ☐ ... porque es natural (por ejemplo, no modificado genéticamente)
- ☐ ... porque me mantiene en forma (por ejemplo, energético, motivado)
- ☐ ... porque crecí con ello
- ☐ ... porque me siento solo
- ☐ ... porque es orgánico
- ☐ ... es parte de mis tradiciones (por ejemplo, tradiciones familiares, ocasiones especiales)
- ☐ ... porque necesito energía
- ☐ ... porque es lo más conveniente
- ☐ ... porque es barato
- ☐ ... porque esa era la única opción
- ☐ ... porque me siento frustrado
- ☐ ... para evitar decepcionar a alguien que está tratando de hacerme feliz
- ☐ ... porque es fácil de preparar
- ☐ ... porque sabe bien
- ☐ ... porque me apetece de modo espontáneo (por ejemplo, colores llamativos a la vista)
- ☐ ... porque quiero comerlo todos los días
- ☐ ... porque estoy acostumbrado a comerlo
- ☐ ... porque no contiene sustancias peligrosas (por ejemplo, pesticidas, contaminantes, o antibióticos
- ☐ ... para complacerme
- ☐ ... porque lo disfruto
- ☐ ... porque es rápido de preparar
- ☐ ... para mantener una dieta equilibrada
- ☐ ... porque vigilo mi peso
- ☐ ... porque me apetece
- ☐ ... porque es social
- ☐ ...porque está de moda
- ☐ ... porque hace más agradables las reuniones sociales
- ☐ ... porque se consume en ciertas ocasiones
- ☐ ... porque me llena de un modo agradable
- ☐ ... porque es saludable

Por favor indíquenos las razones por las que usted no consume turrón (Marque todas las opciones que apliquen)

- ☐ No quise consentirme
- ☐ No es un tipo de comida que consumo en presencia de otras personas
- ☐ No era apropiado para la ocasión
- ☐ No es práctico de preparar
- ☐ Se supone que no debo consumirlo
- ☐ Es muy alto en calorías
- ☐ Mi generación no consume este tipo de alimento
- ☐ Nunca lo escogería. Me gusta consumir el mismo alimento todos los días
- ☐ No era algo que se me apetecía en ese momento
- ☐ El tamaño de la porción no era adecuado
- ☐ No me gusta
- ☐ El precio era muy elevado
- ☐ No me gustó la apariencia
- ☐ No lo considero un alimento que llene lo suficiente
- ☐ No es saludable
- ☐ Me hace sentir triste, solitario, o frustrado
- ☐ No lo consumo usualmente
- ☐ No es orgánico
- ☐ Lo consumí recientemente y no quiero consumir el mismo alimento muy seguido
- ☐ No lo considero como un aperitivo/snack

JAR & Liking

¿Qué tan corto/largo piensa que fué esta encuesta?

- ○ Demasiado corta
- ○ Muy corta
- ○ Un poco muy corta
- ○ Justo lo correcto

- Un poco muy larga
- Muy larga
- Demasiado larga

¿Cómo se sintió al tomar esta encuesta después de que terminó?

- Me gustó mucho la encuesta
- Me gustó tomar esta encuesta
- Me es indiferente, no siento nada al tomar esta encuesta
- No me gustó tomar esta encuesta
- Odié esta encuesta

Reasons for Starchy food choices(Full CATA)

¿Come usted paella?

- Sí
- No

¿Usted come paella porque está acostumbrado a comerlo?

- Sí
- No

¿Usted come paella porque es saludable?

- Sí
- No

¿Usted come paella porque es rápido de preparar?

- Sí
- No

¿Usted come paella porque sabe bien?

- Sí
- No

¿Usted come paella para complacerse?

- Sí
- No

¿Usted come paella porque es natural (por ejemplo, no modificado genéticamente)?

- Sí
- No

¿Usted come paella porque no quiere gastar más dinero?

- Sí
- No

¿Usted come paella porque es bajo en calorías?

- Sí
- No

¿Usted come paella porque se siente frustrado?

- Sí
- No

¿Usted come paella porque le hace quedar bien delante de los demás?

- Sí
- No

¿Usted come paella porque sería descortés no comerlo?

- Sí
- No

¿Usted come paella porque la presentación es atractiva (por ejemplo, el empaque)?

- Sí
- No

¿Usted come paella para poder pasar tiempo con otras personas y socializar?

- Sí
- No

¿Usted come paella porque es orgánico?

- Sí
- No

¿Usted come paella porque creció con ello?

- Sí
- No

¿Usted come paella porque lo disfruta?

- Sí
- No

¿Usted come paella porque tiene hambre?

- Sí
- No

¿Usted come paella porque usualmente lo come?

- Sí

- No

¿Usted come paella porque le apetece?

- Sí
- No

¿Usted come paella porque es fácil de preparar?

- Sí
- No

¿Usted come paella porque se siente triste?

- Sí
- No

¿Usted come paella porque es bajo en grasa?

- Sí
- No

¿Usted come paella porque lo ha visto en anuncios o en la televisión?

- Sí
- No

¿Usted come paella porque hace más agradables las reuniones sociales?

- Sí
- No

¿Usted come paella porque se supone que debe comerlo?

- Sí
- No

¿Usted come paella porque le gusta a otros?

- Sí
- No

¿Usted come paella porque se siente solo?

- Sí
- No

¿Usted come paella porque vigila su peso?

- Sí
- No

¿Usted come paella porque no contiene sustancias peligrosas (por ejemplo, pesticidas, contaminantes, o antibióticos?

- Sí
- No

¿Usted come paella porque es lo más conveniente?

- Sí
- No

¿Usted come paella porque necesita energía?

- Sí
- No

¿Usted come paella porque está familiarizado con ello?

- Sí
- No

¿Usted come paella porque le gusta?

- Sí
- No

¿Usted come paella porque le llena de un modo agradable?

- Sí
- No

¿Usted come paella para recompensarse?

- Sí
- No

¿Usted come paella porque es parte de sus tradiciones (por ejemplo, tradiciones familiares, ocasiones especiales)?

- Sí
- No

¿Usted come paella porque se consume en ciertas ocasiones?

- Sí
- No

¿Usted come paella para mantener una dieta equilibrada?

- Sí
- No

¿Usted come paella porque es social?

- Sí
- No

¿Usted come paella porque es barato?

- Sí
- No

¿Usted come paella porque le apetece de modo espontáneo (por ejemplo, colores llamativos a la vista)?

- Sí
- No

¿Usted come paella para evitar decepcionar a alguien que está tratando de hacerle feliz?

- Sí
- No

¿Usted come paella porque está de moda?

- Sí
- No

¿Usted come paella porque está en rebaja?

- Sí
- No

¿Usted come paella porque le mantiene en forma (por ejemplo, energético, motivado)?

- Sí
- No

¿Usted come paella porque esa era la única opción?

- Sí
- No

¿Usted come paella porque quiero comerlo todos los días?

- Sí
- No

¿Usted no come paella porque no lo considera como un aperitivo/snack?

- Sí
- No

¿Usted no come paella porque no le gusta?

Sí

No

¿Usted no come paella porque no lo consume usualmente?

Sí

No

¿Usted no come paella porque el tamaño de la porción no era adecuado?

Sí

No

¿Usted no come paella porque no lo considera un alimento que llene lo suficiente?

Sí

No

¿Usted no come paella porque no es saludable?

Sí

No

¿Usted no come paella porque no es práctico de preparar?

Sí

No

¿Usted no come paella porque no quiso consentirse?

Sí

No

¿Usted no come paella porque no era apropiado para la ocasión?

Sí

No

¿Usted no come paella porque no es un tipo de comida que consumo en presencia de otras personas?

Sí

No

¿Usted no come paella porque el precio era muy elevado?

Sí

No

¿Usted no come paella porque no le gustó la apariencia?

Sí

No

¿Usted no come paella porque es muy alto en calorías?

- Sí
- No

¿Usted no come paella porque se supone que no debo consumirlo?

- Sí
- No

¿Usted no come paella porque mi generación no consume este tipo de alimento?

- Sí
- No

¿Usted no come paella porque no era algo que se me apetecía en ese momento?

- Sí
- No

¿Usted no come paella porque lo consumí recientemente y no quiero consumir el mismo alimento muy seguido?

- Sí
- No

¿Usted no come paella porque no le gusta consumir el mismo alimento todos los días?

- Sí
- No

¿Usted no come paella porque lo hace sentir triste, solitario, o frustrado?

- Sí
- No

¿Usted no come paella porque no es orgánico?

- Sí
- No

Reasons for Protein foods Choices(Full CATA)

¿Come usted jamón serrano?

- Sí
- No

¿Usted come jamón serrano porque está acostumbrado a comerlo?

- Sí
- No

¿Usted come jamón serrano porque es saludable?

- Sí
- No

¿Usted come jamón serrano porque es rápido de preparar?

- Sí
- No

¿Usted come jamón serrano porque sabe bien?

- Sí
- No

¿Usted come jamón serrano para complacerse?

- Sí
- No

¿Usted come jamón serrano porque es natural (por ejemplo, no modificado genéticamente)?

- Sí
- No

¿Usted come jamón serrano porque no quiere gastar más dinero?

- Sí
- No

¿Usted come jamón serrano porque es bajo en calorías?

- Sí
- No

¿Usted come jamón serrano porque se siente frustrado?

- Sí
- No

¿Usted come jamón serrano porque le hace quedar bien delante de los demás?

- Sí
- No

¿Usted come jamón serrano porque sería descortés no comerlo?

- Sí
- No

¿Usted come jamón serrano porque la presentación es atractiva (por ejemplo, el empaque)?

- Sí
- No

¿Usted come jamón serrano para poder pasar tiempo con otras personas y socializar?

- Sí
- No

¿Usted come jamón serrano porque es orgánico?

- Sí
- No

¿Usted come jamón serrano porque creció con ello?

- Sí
- No

¿Usted come jamón serrano porque lo disfruta?

- Sí
- No

¿Usted come jamón serrano porque tiene hambre?

- Sí

No

¿Usted come jamón serrano porque usualmente lo come?

Sí

No

¿Usted come jamón serrano porque le apetece?

Sí

No

¿Usted come jamón serrano porque es fácil de preparar?

Sí

No

¿Usted come jamón serrano porque se siente triste?

Sí

No

¿Usted come jamón serrano porque es bajo en grasa?

Sí

No

¿Usted come jamón serrano porque lo ha visto en anuncios o en la televisión?

- Sí
- No

¿Usted come jamón serrano porque hace más agradables las reuniones sociales?

- Sí
- No

¿Usted come jamón serrano porque se supone que debe comerlo?

- Sí
- No

¿Usted come jamón serrano porque le gusta a otros?

- Sí
- No

¿Usted come jamón serrano porque se siente solo?

- Sí
- No

¿Usted come jamón serrano porque vigila su peso?

- Sí
- No

¿Usted come jamón serrano porque no contiene sustancias peligrosas (por ejemplo, pesticidas, contaminantes, o antibióticos?

- Sí
- No

¿Usted come jamón serrano porque es lo más conveniente?

- Sí
- No

¿Usted come jamón serrano porque necesita energía?

- Sí
- No

¿Usted come jamón serrano porque está familiarizado con ello?

- Sí
- No

¿Usted come jamón serrano porque le gusta?

- Sí
- No

¿Usted come jamón serrano porque le llena de un modo agradable?

- Sí
- No

¿Usted come jamón serrano para recompensarse?

- Sí
- No

¿Usted come jamón serrano porque es parte de sus tradiciones (por ejemplo, tradiciones familiares, ocasiones especiales)?

- Sí
- No

¿Usted come jamón serrano porque se consume en ciertas ocasiones?

- Sí
- No

¿Usted come jamón serrano para mantener una dieta equilibrada?

- Sí
- No

¿Usted come jamón serrano porque es social?

- Sí
- No

¿Usted come jamón serrano porque es barato?

- Sí
- No

¿Usted come jamón serrano porque le apetece de modo espontáneo (por ejemplo, colores llamativos a la vista)?

- Sí
- No

¿Usted come jamón serrano para evitar decepcionar a alguien que está tratando de hacerle feliz?

- Sí
- No

¿Usted come jamón serrano porque está de moda?

- Sí
- No

¿Usted come jamón serrano porque está en rebaja?

- Sí
- No

¿Usted come jamón serrano porque le mantiene en forma (por ejemplo, energético, motivado)?

- Sí
- No

¿Usted come jamón serrano porque esa era la única opción?

- Sí
- No

¿Usted come jamón serrano porque quiero comerlo todos los días?

- Sí
- No

¿Usted no come jamón serrano porque no lo considera como un aperitivo/snack?

- Sí
- No

¿Usted no come jamón serrano porque no le gusta?

- Sí
- No

¿Usted no come jamón serrano porque no lo consume usualmente?

- Sí
- No

¿Usted no come jamón serrano porque el tamaño de la porción no era adecuado?

- Sí
- No

¿Usted no come jamón serrano porque no lo considera un alimento que llene lo suficiente?

- Sí
- No

¿Usted no come jamón serrano porque no es saludable?

- Sí
- No

¿Usted no come jamón serrano porque no es práctico de preparar?

- Sí
- No

¿Usted no come jamón serrano porque no quiso consentirse?

- Sí
- No

¿Usted no come jamón serrano porque no era apropiado para la ocasión?

- Sí
- No

¿Usted no come jamón serrano porque no es un tipo de comida que consumo en presencia de otras personas?

- Sí
- No

¿Usted no come jamón serrano porque el precio era muy elevado?

- Sí

- No

¿Usted no come jamón serrano porque no le gustó la apariencia?

- Sí
- No

¿Usted no come jamón serrano porque es muy alto en calorías?

- Sí
- No

¿Usted no come jamón serrano porque se supone que no debo consumirlo?

- Sí
- No

¿Usted no come jamón serrano porque mi generación no consume este tipo de alimento?

- Sí
- No

¿Usted no come jamón serrano porque no era algo que se me apetecía en ese momento?

- Sí
- No

¿Usted no come jamón serrano porque lo consumí recientemente y no quiero consumir el mismo alimento muy seguido?

- Sí
- No

¿Usted no come jamón serrano porque no le gusta consumir el mismo alimento todos los días?

- Sí
- No

¿Usted no come jamón serrano porque lo hace sentir triste, solitario, o frustrado?

- Sí
- No

¿Usted no come jamón serrano porque no es orgánico?

- Sí
- No

Reasons for Dairy Food Choices(Full CATA)

¿Come usted leche?

- Sí
- No

¿Usted come leche porque está acostumbrado a comerlo?

- Sí
- No

¿Usted come leche porque es saludable?

- Sí
- No

¿Usted come leche porque es rápido de preparar?

- Sí
- No

¿Usted come leche porque sabe bien?

- Sí
- No

¿Usted come leche para complacerse?

- Sí
- No

¿Usted come leche porque es natural (por ejemplo, no modificado genéticamente)?

- Sí
- No

¿Usted come leche porque no quiere gastar más dinero?

- Sí
- No

¿Usted come leche porque es bajo en calorías?

- Sí
- No

¿Usted come leche porque se siente frustrado?

- Sí
- No

¿Usted come leche porque le hace quedar bien delante de los demás?

- Sí
- No

¿Usted come leche porque sería descortés no comerlo?

- Sí
- No

¿Usted come leche porque la presentación es atractiva (por ejemplo, el empaque)?

- Sí
- No

¿Usted come leche para poder pasar tiempo con otras personas y socializar?

- Sí
- No

¿Usted come leche porque es orgánico?

- Sí
- No

¿Usted come leche porque creció con ello?

- Sí
- No

¿Usted come leche porque lo disfruta?

- Sí
- No

¿Usted come leche porque tiene hambre?

- Sí
- No

¿Usted come leche porque usualmente lo come?

- Sí
- No

¿Usted come leche porque le apetece?

- Sí
- No

¿Usted come leche porque es fácil de preparar?

- Sí
- No

¿Usted come leche porque se siente triste?

- Sí

No

¿Usted come leche porque es bajo en grasa?

Sí

No

¿Usted come leche porque lo ha visto en anuncios o en la televisión?

Sí

No

¿Usted come leche porque hace más agradables las reuniones sociales?

Sí

No

¿Usted come leche porque se supone que debe comerlo?

Sí

No

¿Usted come leche porque le gusta a otros?

Sí

No

¿Usted come leche porque se siente solo?

- Sí
- No

¿Usted come leche porque vigila su peso?

- Sí
- No

¿Usted come leche porque no contiene sustancias peligrosas (por ejemplo, pesticidas, contaminantes, o antibióticos?

- Sí
- No

¿Usted come leche porque es lo más conveniente?

- Sí
- No

¿Usted come leche porque necesita energía?

- Sí
- No

¿Usted come leche porque está familiarizado con ello?

- Sí
- No

¿Usted come leche porque le gusta?

- Sí
- No

¿Usted come leche porque le llena de un modo agradable?

- Sí
- No

¿Usted come leche para recompensarse?

- Sí
- No

¿Usted come leche porque es parte de sus tradiciones (por ejemplo, tradiciones familiares, ocasiones especiales)?

- Sí
- No

¿Usted come leche porque se consume en ciertas ocasiones?

- Sí
- No

¿Usted come leche para mantener una dieta equilibrada?

- Sí
- No

¿Usted come leche porque es social?

- Sí
- No

¿Usted come leche porque es barato?

- Sí
- No

¿Usted come leche porque le apetece de modo espontáneo (por ejemplo, colores llamativos a la vista)?

- Sí
- No

¿Usted come leche para evitar decepcionar a alguien que está tratando de hacerle feliz?

- Sí
- No

¿Usted come leche porque está de moda?

- Sí
- No

¿Usted come leche porque está en rebaja?

- Sí
- No

¿Usted come leche porque le mantiene en forma (por ejemplo, energético, motivado)?

- Sí
- No

¿Usted come leche porque esa era la única opción?

- Sí
- No

¿Usted come leche porque quiero comerlo todos los días?

Sí

No

¿Usted no come leche porque no lo considera como un aperitivo/snack?

Sí

No

¿Usted no come leche porque no le gusta?

Sí

No

¿Usted no come leche porque no lo consume usualmente?

Sí

No

¿Usted no come leche porque el tamaño de la porción no era adecuado?

Sí

No

¿Usted no come leche porque no lo considera un alimento que llene lo suficiente?

Sí

No

¿Usted no come leche porque no es saludable?

Sí

No

¿Usted no come leche porque no es práctico de preparar?

Sí

No

¿Usted no come leche porque no quiso consentirse?

Sí

No

¿Usted no come leche porque no era apropiado para la ocasión?

Sí

No

¿Usted no come leche porque no es un tipo de comida que consumo en presencia de otras personas?

Sí

No

¿Usted no come leche porque el precio era muy elevado?

- Sí
- No

¿Usted no come leche porque no le gustó la apariencia?

- Sí
- No

¿Usted no come leche porque es muy alto en calorías?

- Sí
- No

¿Usted no come leche porque se supone que no debo consumirlo?

- Sí
- No

¿Usted no come leche porque mi generación no consume este tipo de alimento?

- Sí
- No

¿Usted no come leche porque no era algo que se me apetecía en ese momento?

- Sí
- No

¿Usted no come leche porque lo consumí recientemente y no quiero consumir el mismo alimento muy seguido?

- Sí
- No

¿Usted no come leche porque no le gusta consumir el mismo alimento todos los días?

- Sí
- No

¿Usted no come leche porque lo hace sentir triste, solitario, o frustrado?

- Sí
- No

¿Usted no come leche porque no es orgánico?

- Sí
- No

Reasons for Fruit Choices(Full CATA)

¿Come usted plátano ?

- Sí
- No

¿Usted come plátano porque está acostumbrado a comerlo?

- Sí
- No

¿Usted come plátano porque es saludable?

- Sí
- No

¿Usted come plátano porque es rápido de preparar?

- Sí
- No

¿Usted come plátano porque sabe bien?

- Sí
- No

¿Usted come plátano para complacerse?

- Sí
- No

¿Usted come plátano porque es natural (por ejemplo, no modificado genéticamente)?

- Sí
- No

¿Usted come plátano porque no quiere gastar más dinero?

- Sí
- No

¿Usted come plátano porque es bajo en calorías?

- Sí
- No

¿Usted come plátano porque se siente frustrado?

- Sí
- No

¿Usted come plátano porque le hace quedar bien delante de los demás?

- Sí
- No

¿Usted come plátano porque sería descortés no comerlo?

- Sí
- No

¿Usted come plátano porque la presentación es atractiva (por ejemplo, el empaque)?

- Sí
- No

¿Usted come plátano para poder pasar tiempo con otras personas y socializar?

- Sí
- No

¿Usted come plátano porque es orgánico?

- Sí
- No

¿Usted come plátano porque creció con ello?

- Sí
- No

¿Usted come plátano porque lo disfruta?

- Sí
- No

¿Usted come plátano porque tiene hambre?

- Sí
- No

¿Usted come plátano porque usualmente lo come?

- Sí
- No

¿Usted come plátano porque le apetece?

- Sí
- No

¿Usted come plátano porque es fácil de preparar?

- Sí

- No

¿Usted come plátano porque se siente triste?

- Sí
- No

¿Usted come plátano porque es bajo en grasa?

- Sí
- No

¿Usted come plátano porque lo ha visto en anuncios o en la televisión?

- Sí
- No

¿Usted come plátano porque hace más agradables las reuniones sociales?

- Sí
- No

¿Usted come plátano porque se supone que debe comerlo?

- Sí
- No

¿Usted come plátano porque le gusta a otros?

- Sí
- No

¿Usted come plátano porque se siente solo?

- Sí
- No

¿Usted come plátano porque vigila su peso?

- Sí
- No

¿Usted come plátano porque no contiene sustancias peligrosas (por ejemplo, pesticidas, contaminantes, o antibióticos?

- Sí
- No

¿Usted come plátano porque es lo más conveniente?

- Sí
- No

¿Usted come plátano porque necesita energía?

- Sí
- No

¿Usted come plátano porque está familiarizado con ello?

- Sí
- No

¿Usted come plátano porque le gusta?

- Sí
- No

¿Usted come plátano porque le llena de un modo agradable?

- Sí
- No

¿Usted come plátano para recompensarse?

- Sí
- No

¿Usted come plátano porque es parte de sus tradiciones (por ejemplo, tradiciones familiares, ocasiones especiales)?

- Sí
- No

¿Usted come plátano porque se consume en ciertas ocasiones?

- Sí
- No

¿Usted come plátano para mantener una dieta equilibrada?

- Sí
- No

¿Usted come plátano porque es social?

- Sí
- No

¿Usted come plátano porque es barato?

- Sí
- No

¿Usted come plátano porque le apetece de modo espontáneo (por ejemplo, colores llamativos a la vista)?

- Sí
- No

¿Usted come plátano para evitar decepcionar a alguien que está tratando de hacerle feliz?

- Sí
- No

¿Usted come plátano porque está de moda?

- Sí
- No

¿Usted come plátano porque está en rebaja?

- Sí
- No

¿Usted come plátano porque le mantiene en forma (por ejemplo, energético, motivado)?

- Sí
- No

¿Usted come plátano porque esa era la única opción?

- Sí
- No

¿Usted come plátano porque quiero comerlo todos los días?

- Sí
- No

¿Usted no come plátano porque no lo considera como un aperitivo/snack?

- Sí
- No

¿Usted no come plátano porque no le gusta?

- Sí
- No

¿Usted no come plátano porque no lo consume usualmente?

- Sí
- No

¿Usted no come plátano porque el tamaño de la porción no era adecuado?

- Sí

No

¿Usted no come plátano porque no lo considera un alimento que llene lo suficiente?

Sí

No

¿Usted no come plátano porque no es saludable?

Sí

No

¿Usted no come plátano porque no es práctico de preparar?

Sí

No

¿Usted no come plátano porque no quiso consentirse?

Sí

No

¿Usted no come plátano porque no era apropiado para la ocasión?

Sí

No

¿Usted no come plátano porque no es un tipo de comida que consumo en presencia de otras personas?

- Sí
- No

¿Usted no come plátano porque el precio era muy elevado?

- Sí
- No

¿Usted no come plátano porque no le gustó la apariencia?

- Sí
- No

¿Usted no come plátano porque es muy alto en calorías?

- Sí
- No

¿Usted no come plátano porque se supone que no debo consumirlo?

- Sí
- No

¿Usted no come plátano porque mi generación no consume este tipo de alimento?

- Sí
- No

¿Usted no come plátano porque no era algo que se me apetecía en ese momento?

- Sí
- No

¿Usted no come plátano porque lo consumí recientemente y no quiero consumir el mismo alimento muy seguido?

- Sí
- No

¿Usted no come plátano porque no le gusta consumir el mismo alimento todos los días?

- Sí
- No

¿Usted no come plátano porque lo hace sentir triste, solitario, o frustrado?

- Sí
- No

¿Usted no come plátano porque no es orgánico?

- Sí
- No

Reasons for Sweet food Choices(Full CATA)

¿Come usted turrón ?

- Sí
- No

¿Usted come turrón porque está acostumbrado a comerlo?

- Sí
- No

¿Usted come turrón porque es saludable?

- Sí
- No

¿Usted come turrón porque es rápido de preparar?

- Sí
- No

¿Usted come turrón porque sabe bien?

- Sí
- No

¿Usted come turrón para complacerse?

- Sí
- No

¿Usted come turrón porque es natural (por ejemplo, no modificado genéticamente)?

- Sí
- No

¿Usted come turrón porque no quiere gastar más dinero?

- Sí
- No

¿Usted come turrón porque es bajo en calorías?

- Sí
- No

¿Usted come turrón porque se siente frustrado?

- Sí
- No

¿Usted come turrón porque le hace quedar bien delante de los demás?

- Sí
- No

¿Usted come turrón porque sería descortés no comerlo?

- Sí
- No

¿Usted come turrón porque la presentación es atractiva (por ejemplo, el empaque)?

- Sí
- No

¿Usted come turrón para poder pasar tiempo con otras personas y socializar?

- Sí
- No

¿Usted come turrón porque es orgánico?

- Sí
- No

¿Usted come turrón porque creció con ello?

- Sí
- No

¿Usted come turrón porque lo disfruta?

- Sí
- No

¿Usted come turrón porque tiene hambre?

- Sí
- No

¿Usted come turrón porque usualmente lo come?

- Sí
- No

¿Usted come turrón porque le apetece?

- Sí

No

¿Usted come turrón porque es fácil de preparar?

Sí

No

¿Usted come turrón porque se siente triste?

Sí

No

¿Usted come turrón porque es bajo en grasa?

Sí

No

¿Usted come turrón porque lo ha visto en anuncios o en la televisión?

Sí

No

¿Usted come turrón porque hace más agradables las reuniones sociales?

Sí

No

¿Usted come turrón porque se supone que debe comerlo?

- Sí
- No

¿Usted come turrón porque le gusta a otros?

- Sí
- No

¿Usted come turrón porque se siente solo?

- Sí
- No

¿Usted come turrón porque vigila su peso?

- Sí
- No

¿Usted come turrón porque no contiene sustancias peligrosas (por ejemplo, pesticidas, contaminantes, o antibióticos?

- Sí
- No

¿Usted come turrón porque es lo más conveniente?

- Sí
- No

¿Usted come turrón porque necesita energía?

- Sí
- No

¿Usted come turrón porque está familiarizado con ello?

- Sí
- No

¿Usted come turrón porque le gusta?

- Sí
- No

¿Usted come turrón porque le llena de un modo agradable?

- Sí
- No

¿Usted come turrón para recompensarse?

- Sí
- No

¿Usted come turrón porque es parte de sus tradiciones (por ejemplo, tradiciones familiares, ocasiones especiales)?

- Sí
- No

¿Usted come turrón porque se consume en ciertas ocasiones?

- Sí
- No

¿Usted come turrón para mantener una dieta equilibrada?

- Sí
- No

¿Usted come turrón porque es social?

- Sí
- No

¿Usted come turrón porque es barato?

- Sí
- No

¿Usted come turrón porque le apetece de modo espontáneo (por ejemplo, colores llamativos a la vista)?

- Sí
- No

¿Usted come turrón para evitar decepcionar a alguien que está tratando de hacerle feliz?

- Sí
- No

¿Usted come turrón porque está de moda?

- Sí
- No

¿Usted come turrón porque está en rebaja?

- Sí
- No

¿Usted come turrón porque le mantiene en forma (por ejemplo, energético, motivado)?

- Sí
- No

¿Usted come turrón porque esa era la única opción?

- Sí
- No

¿Usted come turrón porque quiero comerlo todos los días?

- Sí
- No

¿Usted no come turrón porque no lo considera como un aperitivo/snack?

- Sí
- No

¿Usted no come turrón porque no le gusta?

- Sí
- No

¿Usted no come turrón porque no lo consume usualmente?

- Sí

No

¿Usted no come turrón porque el tamaño de la porción no era adecuado?

- Sí
- No

¿Usted no come turrón porque no lo considera un alimento que llene lo suficiente?

- Sí
- No

¿Usted no come turrón porque no es saludable?

- Sí
- No

¿Usted no come turrón porque no es práctico de preparar?

- Sí
- No

¿Usted no come turrón porque no quiso consentirse?

- Sí
- No

¿Usted no come turrón porque no era apropiado para la ocasión?

- Sí
- No

¿Usted no come turrón porque no es un tipo de comida que consumo en presencia de otras personas?

- Sí
- No

¿Usted no come turrón porque el precio era muy elevado?

- Sí
- No

¿Usted no come turrón porque no le gustó la apariencia?

- Sí
- No

¿Usted no come turrón porque es muy alto en calorías?

- Sí
- No

¿Usted no come turrón porque se supone que no debo consumirlo?

- Sí
- No

¿Usted no come turrón porque mi generación no consume este tipo de alimento?

- Sí
- No

¿Usted no come turrón porque no era algo que se me apetecía en ese momento?

- Sí
- No

¿Usted no come turrón porque lo consumí recientemente y no quiero consumir el mismo alimento muy seguido?

- Sí
- No

¿Usted no come turrón porque no le gusta consumir el mismo alimento todos los días?

- Sí
- No

¿Usted no come turrón porque lo hace sentir triste, solitario, o frustrado?

- Sí
- No

¿Usted no come turrón porque no es orgánico?

- Sí
- No

Reasons for Starchy food choices(RATA)

¿Come usted paella?

- Sí
- No

¿Cuáles fueron las razones por las que consume paella? (Marque todas las opciones que apliquen)

- ... porque vigilo mi peso
- ... porque quiero comerlo todos los días
- ... porque sería descortés no comerlo
- ... porque me siento frustrado
- ... para mantener una dieta equilibrada
- ... para evitar decepcionar a alguien que está tratando de hacerme feliz
- ... porque usualmente lo como
- ... porque es fácil de preparar
- ... porque hace más agradables las reuniones sociales

- ☐ ... porque me mantiene en forma (por ejemplo, energético, motivado)
- ☐ ... porque me gusta
- ☐ ... porque me apetece
- ☐ ... porque la presentación es atractiva (por ejemplo, el empaque)
- ☐ ... es parte de mis tradiciones (por ejemplo, tradiciones familiares, ocasiones especiales)
- ☐ ... porque es barato
- ☐ ... porque es rápido de preparar
- ☐ ... porque me hace quedar bien delante de los demás
- ☐ ... porque no quiero gastar más dinero
- ☐ ... porque es bajo en calorías
- ☐ ... porque tengo hambre
- ☐ ... porque me siento triste
- ☐ ... porque es natural (por ejemplo, no modificado genéticamente)
- ☐ ... porque esa era la única opción
- ☐ ... porque crecí con ello
- ☐ ... porque lo he visto en anuncios o en la televisión
- ☐ ... porque es orgánico
- ☐ ... porque le gusta a otros
- ☐ ... porque estoy acostumbrado a comerlo
- ☐ ... porque me apetece de modo espontáneo (por ejemplo, colores llamativos a la vista)
- ☐ ... para poder pasar tiempo con otras personas y socializar
- ☐ ... porque estoy familiarizado con ello
- ☐ ... porque está en rebaja
- ☐ ... porque se consume en ciertas ocasiones
- ☐ ... porque me siento solo
- ☐ ... porque necesito energía
- ☐ ...porque está de moda
- ☐ ... porque sabe bien
- ☐ ... porque es social
- ☐ ... porque es lo más conveniente
- ☐ ... para complacerme

- ☐ ...porque es bajo en grasa
- ☐ ... porque no contiene sustancias peligrosas (por ejemplo, pesticidas, contaminantes, o antibióticos
- ☐ ... porque me llena de un modo agradable
- ☐ ... porque es saludable
- ☐ ... para recompensarme
- ☐ ... porque lo disfruto
- ☐ ... porque se supone que debo comerlo

Usted seleccionó"... porque estoy acostumbrado a comerlo"
¿Cuál es la importancia de esta declaración para usted?

- ○ Extremedamente importante
- ○ Muy importante
- ○ Moderadamente importante
- ○ Ligeramente importante
- ○ No es nada importante

Usted seleccionó"... porque es saludable"
¿Cuál es la importancia de esta declaración para usted?

- ○ Extremedamente importante
- ○ Muy importante
- ○ Moderadamente importante
- ○ Ligeramente importante
- ○ No es nada importante

Usted seleccionó"... porque es rápido de preparar"
¿Cuál es la importancia de esta declaración para usted?

- ○ Extremedamente importante
- ○ Muy importante
- ○ Moderadamente importante

- Ligeramente importante
- No es nada importante

Usted seleccionó"... porque sabe bien"
¿Cuál es la importancia de esta declaración para usted?

- Extremedamente importante
- Muy importante
- Moderadamente importante
- Ligeramente importante
- No es nada importante

Usted seleccionó"... para complacerme"
¿Cuál es la importancia de esta declaración para usted?

- Extremedamente importante
- Muy importante
- Moderadamente importante
- Ligeramente importante
- No es nada importante

Usted seleccionó"... porque es natural (por ejemplo, no modificado genéticamente)"
¿Cuál es la importancia de esta declaración para usted?

- Extremedamente importante
- Muy importante
- Moderadamente importante
- Ligeramente importante
- No es nada importante

Usted seleccionó"... porque no quiero gastar más dinero"
¿Cuál es la importancia de esta declaración para usted?

- Extremedamente importante
- Muy importante
- Moderadamente importante
- Ligeramente importante
- No es nada importante

Usted seleccionó"... porque es bajo en calorías"
¿Cuál es la importancia de esta declaración para usted?

- Extremedamente importante
- Muy importante
- Moderadamente importante
- Ligeramente importante
- No es nada importante

Usted seleccionó"... porque me siento frustrado"
¿Cuál es la importancia de esta declaración para usted?

- Extremedamente importante
- Muy importante
- Moderadamente importante
- Ligeramente importante
- No es nada importante

Usted seleccionó"... porque me hace quedar bien delante de los demás"
¿Cuál es la importancia de esta declaración para usted?

- Extremedamente importante
- Muy importante
- Moderadamente importante
- Ligeramente importante
- No es nada importante

Usted seleccionó"... porque sería descortés no comerlo"
¿Cuál es la importancia de esta declaración para usted?

- Extremedamente importante
- Muy importante
- Moderadamente importante
- Ligeramente importante
- No es nada importante

Usted seleccionó"... porque la presentación es atractiva (por ejemplo, el empaque)"
¿Cuál es la importancia de esta declaración para usted?

- Extremedamente importante
- Muy importante
- Moderadamente importante
- Ligeramente importante
- No es nada importante

Usted seleccionó"... para poder pasar tiempo con otras personas y socializar"
¿Cuál es la importancia de esta declaración para usted?

- Extremedamente importante
- Muy importante
- Moderadamente importante
- Ligeramente importante
- No es nada importante

Usted seleccionó"... porque es orgánico"
¿Cuál es la importancia de esta declaración para usted?

- Extremedamente importante
- Muy importante
- Moderadamente importante
- Ligeramente importante

- No es nada importante

Usted seleccionó"... porque crecí con ello"
¿Cuál es la importancia de esta declaración para usted?

- Extremedamente importante
- Muy importante
- Moderadamente importante
- Ligeramente importante
- No es nada importante

Usted seleccionó"... porque lo disfruto"
¿Cuál es la importancia de esta declaración para usted?

- Extremedamente importante
- Muy importante
- Moderadamente importante
- Ligeramente importante
- No es nada importante

Usted seleccionó"... porque tengo hambre"
¿Cuál es la importancia de esta declaración para usted?

- Extremedamente importante
- Muy importante
- Moderadamente importante
- Ligeramente importante
- No es nada importante

Usted seleccionó"... porque usualmente lo como"
¿Cuál es la importancia de esta declaración para usted?

- Extremedamente importante

- Muy importante
- Moderadamente importante
- Ligeramente importante
- No es nada importante

Usted seleccionó"... porque me apetece"
¿Cuál es la importancia de esta declaración para usted?

- Extremedamente importante
- Muy importante
- Moderadamente importante
- Ligeramente importante
- No es nada importante

Usted seleccionó"... porque es fácil de preparar"
¿Cuál es la importancia de esta declaración para usted?

- Extremedamente importante
- Muy importante
- Moderadamente importante
- Ligeramente importante
- No es nada importante

Usted seleccionó"... porque me siento triste"
¿Cuál es la importancia de esta declaración para usted?

- Extremedamente importante
- Muy importante
- Moderadamente importante
- Ligeramente importante
- No es nada importante

Usted seleccionó"...porque es bajo en grasa"
¿Cuál es la importancia de esta declaración para usted?

- Extremedamente importante
- Muy importante
- Moderadamente importante
- Ligeramente importante
- No es nada importante

Usted seleccionó"... porque lo he visto en anuncios o en la televisión"
¿Cuál es la importancia de esta declaración para usted?

- Extremedamente importante
- Muy importante
- Moderadamente importante
- Ligeramente importante
- No es nada importante

Usted seleccionó"... porque hace más agradables las reuniones sociales"
¿Cuál es la importancia de esta declaración para usted?

- Extremedamente importante
- Muy importante
- Moderadamente importante
- Ligeramente importante
- No es nada importante

Usted seleccionó"... porque se supone que debo comerlo"
¿Cuál es la importancia de esta declaración para usted?

- Extremedamente importante
- Muy importante
- Moderadamente importante
- Ligeramente importante

- No es nada importante

Usted seleccionó"... porque le gusta a otros"
¿Cuál es la importancia de esta declaración para usted?

- Extremedamente importante
- Muy importante
- Moderadamente importante
- Ligeramente importante
- No es nada importante

Usted seleccionó"... porque me siento solo"
¿Cuál es la importancia de esta declaración para usted?

- Extremedamente importante
- Muy importante
- Moderadamente importante
- Ligeramente importante
- No es nada importante

Usted seleccionó"... porque vigilo mi peso"
¿Cuál es la importancia de esta declaración para usted?

- Extremedamente importante
- Muy importante
- Moderadamente importante
- Ligeramente importante
- No es nada importante

Usted seleccionó"... porque no contiene sustancias peligrosas (por ejemplo, pesticidas, contaminantes, o antibióticos"
¿Cuál es la importancia de esta declaración para usted?

- Extremedamente importante
- Muy importante
- Moderadamente importante
- Ligeramente importante
- No es nada importante

Usted seleccionó"... porque es lo más conveniente"
¿Cuál es la importancia de esta declaración para usted?

- Extremedamente importante
- Muy importante
- Moderadamente importante
- Ligeramente importante
- No es nada importante

Usted seleccionó"... porque necesito energía"
¿Cuál es la importancia de esta declaración para usted?

- Extremedamente importante
- Muy importante
- Moderadamente importante
- Ligeramente importante
- No es nada importante

Usted seleccionó"... porque estoy familiarizado con ello"
¿Cuál es la importancia de esta declaración para usted?

- Extremedamente importante
- Muy importante
- Moderadamente importante
- Ligeramente importante
- No es nada importante

Usted seleccionó"... porque me gusta"
¿Cuál es la importancia de esta declaración para usted?

- Extremedamente importante
- Muy importante
- Moderadamente importante
- Ligeramente importante
- No es nada importante

Usted seleccionó"... porque me llena de un modo agradable"
¿Cuál es la importancia de esta declaración para usted?

- Extremedamente importante
- Muy importante
- Moderadamente importante
- Ligeramente importante
- No es nada importante

Usted seleccionó"... para recompensarme"
¿Cuál es la importancia de esta declaración para usted?

- Extremedamente importante
- Muy importante
- Moderadamente importante
- Ligeramente importante
- No es nada importante

Usted seleccionó"... es parte de mis tradiciones (por ejemplo, tradiciones familiares, ocasiones especiales)"
¿Cuál es la importancia de esta declaración para usted?

- Extremedamente importante
- Muy importante
- Moderadamente importante
- Ligeramente importante

- No es nada importante

Usted seleccionó"... porque se consume en ciertas ocasiones"
¿Cuál es la importancia de esta declaración para usted?

- Extremedamente importante
- Muy importante
- Moderadamente importante
- Ligeramente importante
- No es nada importante

Usted seleccionó"... para mantener una dieta equilibrada"
¿Cuál es la importancia de esta declaración para usted?

- Extremedamente importante
- Muy importante
- Moderadamente importante
- Ligeramente importante
- No es nada importante

Usted seleccionó"... porque es social"
¿Cuál es la importancia de esta declaración para usted?

- Extremedamente importante
- Muy importante
- Moderadamente importante
- Ligeramente importante
- No es nada importante

Usted seleccionó"... porque es barato"
¿Cuál es la importancia de esta declaración para usted?

- Extremedamente importante

- Muy importante
- Moderadamente importante
- Ligeramente importante
- No es nada importante

Usted seleccionó"... porque me apetece de modo espontáneo (por ejemplo, colores llamativos a la vista)"
¿Cuál es la importancia de esta declaración para usted?

- Extremedamente importante
- Muy importante
- Moderadamente importante
- Ligeramente importante
- No es nada importante

Usted seleccionó"... para evitar decepcionar a alguien que está tratando de hacerme feliz"
¿Cuál es la importancia de esta declaración para usted?

- Extremedamente importante
- Muy importante
- Moderadamente importante
- Ligeramente importante
- No es nada importante

Usted seleccionó"...porque está de moda"
¿Cuál es la importancia de esta declaración para usted?

- Extremedamente importante
- Muy importante
- Moderadamente importante
- Ligeramente importante
- No es nada importante

Usted seleccionó"... porque está en rebaja"
¿Cuál es la importancia de esta declaración para usted?

- Extremedamente importante
- Muy importante
- Moderadamente importante
- Ligeramente importante
- No es nada importante

Usted seleccionó"... porque me mantiene en forma (por ejemplo, energético, motivado)"
¿Cuál es la importancia de esta declaración para usted?

- Extremedamente importante
- Muy importante
- Moderadamente importante
- Ligeramente importante
- No es nada importante

Usted seleccionó"... porque esa era la única opción"
¿Cuál es la importancia de esta declaración para usted?

- Extremedamente importante
- Muy importante
- Moderadamente importante
- Ligeramente importante
- No es nada importante

Usted seleccionó"... porque quiero comerlo todos los días"
¿Cuál es la importancia de esta declaración para usted?

- Extremedamente importante
- Muy importante
- Moderadamente importante
- Ligeramente importante

- No es nada importante

Por favor indíquenos las razones por las que usted no consume paella (Marque todas las opciones que apliquen)

- No lo consumo usualmente
- No es práctico de preparar
- El tamaño de la porción no era adecuado
- Se supone que no debo consumirlo
- No era apropiado para la ocasión
- No lo considero un alimento que llene lo suficiente
- No es orgánico
- Me hace sentir triste, solitario, o frustrado
- No lo considero como un aperitivo/snack
- No es un tipo de comida que consumo en presencia de otras personas
- Nunca lo escogería. Me gusta consumir el mismo alimento todos los días
- No quise consentirme
- Lo consumí recientemente y no quiero consumir el mismo alimento muy seguido
- Es muy alto en calorías
- Mi generación no consume este tipo de alimento
- El precio era muy elevado
- No es saludable
- No era algo que se me apetecía en ese momento
- No me gusta
- No me gustó la apariencia

Usted seleccionó"No lo considero como un aperitivo/snack"
¿Cuál es la importancia de esta declaración para usted?

- Extremedamente importante
- Muy importante
- Moderadamente importante
- Ligeramente importante

- No es nada importante

Usted seleccionó"No me gusta"
¿Cuál es la importancia de esta declaración para usted?

- Extremedamente importante
- Muy importante
- Moderadamente importante
- Ligeramente importante
- No es nada importante

Usted seleccionó"No lo consumo usualmente"
¿Cuál es la importancia de esta declaración para usted?

- Extremedamente importante
- Muy importante
- Moderadamente importante
- Ligeramente importante
- No es nada importante

Usted seleccionó"El tamaño de la porción no era adecuado"
¿Cuál es la importancia de esta declaración para usted?

- Extremedamente importante
- Muy importante
- Moderadamente importante
- Ligeramente importante
- No es nada importante

Usted seleccionó"No lo considero un alimento que llene lo suficiente"
¿Cuál es la importancia de esta declaración para usted?

- Extremedamente importante

- Muy importante
- Moderadamente importante
- Ligeramente importante
- No es nada importante

Usted seleccionó"No es saludable"
¿Cuál es la importancia de esta declaración para usted?

- Extremedamente importante
- Muy importante
- Moderadamente importante
- Ligeramente importante
- No es nada importante

Usted seleccionó"No es práctico de preparar"
¿Cuál es la importancia de esta declaración para usted?

- Extremedamente importante
- Muy importante
- Moderadamente importante
- Ligeramente importante
- No es nada importante

Usted seleccionó"No quise consentirme"
¿Cuál es la importancia de esta declaración para usted?

- Extremedamente importante
- Muy importante
- Moderadamente importante
- Ligeramente importante
- No es nada importante

Usted seleccionó"No era apropiado para la ocasión"
¿Cuál es la importancia de esta declaración para usted?

- Extremedamente importante
- Muy importante
- Moderadamente importante
- Ligeramente importante
- No es nada importante

Usted seleccionó"No es un tipo de comida que consumo en presencia de otras personas"
¿Cuál es la importancia de esta declaración para usted?

- Extremedamente importante
- Muy importante
- Moderadamente importante
- Ligeramente importante
- No es nada importante

Usted seleccionó"El precio era muy elevado"
¿Cuál es la importancia de esta declaración para usted?

- Extremedamente importante
- Muy importante
- Moderadamente importante
- Ligeramente importante
- No es nada importante

Usted seleccionó"No me gustó la apariencia"
¿Cuál es la importancia de esta declaración para usted?

- Extremedamente importante
- Muy importante
- Moderadamente importante
- Ligeramente importante

- No es nada importante

Usted seleccionó"Es muy alto en calorías"
¿Cuál es la importancia de esta declaración para usted?

- Extremedamente importante
- Muy importante
- Moderadamente importante
- Ligeramente importante
- No es nada importante

Usted seleccionó"Se supone que no debo consumirlo"
¿Cuál es la importancia de esta declaración para usted?

- Extremedamente importante
- Muy importante
- Moderadamente importante
- Ligeramente importante
- No es nada importante

Usted seleccionó"Mi generación no consume este tipo de alimento"
¿Cuál es la importancia de esta declaración para usted?

- Extremedamente importante
- Muy importante
- Moderadamente importante
- Ligeramente importante
- No es nada importante

Usted seleccionó"No era algo que se me apetecía en ese momento"
¿Cuál es la importancia de esta declaración para usted?

- Extremedamente importante

- Muy importante
- Moderadamente importante
- Ligeramente importante
- No es nada importante

Usted seleccionó"Lo consumí recientemente y no quiero consumir el mismo alimento muy seguido"
¿Cuál es la importancia de esta declaración para usted?

- Extremedamente importante
- Muy importante
- Moderadamente importante
- Ligeramente importante
- No es nada importante

Usted seleccionó"Nunca lo escogería. Me gusta consumir el mismo alimento todos los días"
¿Cuál es la importancia de esta declaración para usted?

- Extremedamente importante
- Muy importante
- Moderadamente importante
- Ligeramente importante
- No es nada importante

Usted seleccionó"Me hace sentir triste, solitario, o frustrado"
¿Cuál es la importancia de esta declaración para usted?

- Extremedamente importante
- Muy importante
- Moderadamente importante
- Ligeramente importante
- No es nada importante

Usted seleccionó"No es orgánico"
¿Cuál es la importancia de esta declaración para usted?

- Extremedamente importante
- Muy importante
- Moderadamente importante
- Ligeramente importante
- No es nada importante

Reasons for Protein foods Choices(RATA)

¿Come usted jamón serrano?

- Sí
- No

¿Cuáles fueron las razones por las que consume jamón serrano? (Marque todas las opciones que apliquen)

- ... porque sabe bien
- ... porque me apetece
- ... porque es rápido de preparar
- ... porque es social
- ...porque está de moda
- ... porque me siento solo
- ... porque quiero comerlo todos los días
- ... porque hace más agradables las reuniones sociales
- ... porque esa era la única opción
- ... porque estoy familiarizado con ello
- ...porque es bajo en grasa
- ... es parte de mis tradiciones (por ejemplo, tradiciones familiares, ocasiones especiales)

- [ ] ... para mantener una dieta equilibrada
- [ ] ... porque sería descortés no comerlo
- [ ] ... porque es lo más conveniente
- [ ] ... porque es fácil de preparar
- [ ] ... porque necesito energía
- [ ] ... porque la presentación es atractiva (por ejemplo, el empaque)
- [ ] ... porque me siento frustrado
- [ ] ... porque crecí con ello
- [ ] ... porque me siento triste
- [ ] ... porque me llena de un modo agradable
- [ ] ... porque es saludable
- [ ] ... porque se consume en ciertas ocasiones
- [ ] ... porque no contiene sustancias peligrosas (por ejemplo, pesticidas, contaminantes, o antibióticos
- [ ] ... porque me apetece de modo espontáneo (por ejemplo, colores llamativos a la vista)
- [ ] ... para complacerme
- [ ] ... porque es bajo en calorías
- [ ] ... porque lo disfruto
- [ ] ... porque me mantiene en forma (por ejemplo, energético, motivado)
- [ ] ... porque no quiero gastar más dinero
- [ ] ... porque me gusta
- [ ] ... porque está en rebaja
- [ ] ... para evitar decepcionar a alguien que está tratando de hacerme feliz
- [ ] ... para recompensarme
- [ ] ... porque lo he visto en anuncios o en la televisión
- [ ] ... porque es orgánico
- [ ] ... para poder pasar tiempo con otras personas y socializar
- [ ] ... porque me hace quedar bien delante de los demás
- [ ] ... porque le gusta a otros
- [ ] ... porque estoy acostumbrado a comerlo
- [ ] ... porque es natural (por ejemplo, no modificado genéticamente)
- [ ] ... porque es barato

- ☐ ... porque usualmente lo como
- ☐ ... porque se supone que debo comerlo
- ☐ ... porque tengo hambre
- ☐ ... porque vigilo mi peso

Usted seleccionó"... porque estoy acostumbrado a comerlo"
¿Cuál es la importancia de esta declaración para usted?

- ○ Extremedamente importante
- ○ Muy importante
- ○ Moderadamente importante
- ○ Ligeramente importante
- ○ No es nada importante

Usted seleccionó"... porque es saludable"
¿Cuál es la importancia de esta declaración para usted?

- ○ Extremedamente importante
- ○ Muy importante
- ○ Moderadamente importante
- ○ Ligeramente importante
- ○ No es nada importante

Usted seleccionó"... porque es rápido de preparar"
¿Cuál es la importancia de esta declaración para usted?

- ○ Extremedamente importante
- ○ Muy importante
- ○ Moderadamente importante
- ○ Ligeramente importante
- ○ No es nada importante

Usted seleccionó"... porque sabe bien"
¿Cuál es la importancia de esta declaración para usted?

- Extremedamente importante
- Muy importante
- Moderadamente importante
- Ligeramente importante
- No es nada importante

Usted seleccionó"... para complacerme"
¿Cuál es la importancia de esta declaración para usted?

- Extremedamente importante
- Muy importante
- Moderadamente importante
- Ligeramente importante
- No es nada importante

Usted seleccionó"... porque es natural (por ejemplo, no modificado genéticamente)"
¿Cuál es la importancia de esta declaración para usted?

- Extremedamente importante
- Muy importante
- Moderadamente importante
- Ligeramente importante
- No es nada importante

Usted seleccionó"... porque no quiero gastar más dinero"
¿Cuál es la importancia de esta declaración para usted?

- Extremedamente importante
- Muy importante
- Moderadamente importante
- Ligeramente importante

- No es nada importante

Usted seleccionó"... porque es bajo en calorías"
¿Cuál es la importancia de esta declaración para usted?

- Extremedamente importante
- Muy importante
- Moderadamente importante
- Ligeramente importante
- No es nada importante

Usted seleccionó"... porque me siento frustrado"
¿Cuál es la importancia de esta declaración para usted?

- Extremedamente importante
- Muy importante
- Moderadamente importante
- Ligeramente importante
- No es nada importante

Usted seleccionó"... porque me hace quedar bien delante de los demás"
¿Cuál es la importancia de esta declaración para usted?

- Extremedamente importante
- Muy importante
- Moderadamente importante
- Ligeramente importante
- No es nada importante

Usted seleccionó"... porque sería descortés no comerlo"
¿Cuál es la importancia de esta declaración para usted?

- Extremedamente importante

- Muy importante
- Moderadamente importante
- Ligeramente importante
- No es nada importante

Usted seleccionó"... porque la presentación es atractiva (por ejemplo, el empaque)"
¿Cuál es la importancia de esta declaración para usted?

- Extremedamente importante
- Muy importante
- Moderadamente importante
- Ligeramente importante
- No es nada importante

Usted seleccionó"... para poder pasar tiempo con otras personas y socializar"
¿Cuál es la importancia de esta declaración para usted?

- Extremedamente importante
- Muy importante
- Moderadamente importante
- Ligeramente importante
- No es nada importante

Usted seleccionó"... porque es orgánico"
¿Cuál es la importancia de esta declaración para usted?

- Extremedamente importante
- Muy importante
- Moderadamente importante
- Ligeramente importante
- No es nada importante

Usted seleccionó"... porque crecí con ello"
¿Cuál es la importancia de esta declaración para usted?

- Extremedamente importante
- Muy importante
- Moderadamente importante
- Ligeramente importante
- No es nada importante

Usted seleccionó"... porque lo disfruto"
¿Cuál es la importancia de esta declaración para usted?

- Extremedamente importante
- Muy importante
- Moderadamente importante
- Ligeramente importante
- No es nada importante

Usted seleccionó"... porque tengo hambre"
¿Cuál es la importancia de esta declaración para usted?

- Extremedamente importante
- Muy importante
- Moderadamente importante
- Ligeramente importante
- No es nada importante

Usted seleccionó"... porque usualmente lo como"
¿Cuál es la importancia de esta declaración para usted?

- Extremedamente importante
- Muy importante
- Moderadamente importante
- Ligeramente importante

- No es nada importante

Usted seleccionó"... porque me apetece"
¿Cuál es la importancia de esta declaración para usted?

- Extremedamente importante
- Muy importante
- Moderadamente importante
- Ligeramente importante
- No es nada importante

Usted seleccionó"... porque es fácil de preparar"
¿Cuál es la importancia de esta declaración para usted?

- Extremedamente importante
- Muy importante
- Moderadamente importante
- Ligeramente importante
- No es nada importante

Usted seleccionó"... porque me siento triste"
¿Cuál es la importancia de esta declaración para usted?

- Extremedamente importante
- Muy importante
- Moderadamente importante
- Ligeramente importante
- No es nada importante

Usted seleccionó"...porque es bajo en grasa"
¿Cuál es la importancia de esta declaración para usted?

- Extremedamente importante

- Muy importante
- Moderadamente importante
- Ligeramente importante
- No es nada importante

Usted seleccionó"... porque lo he visto en anuncios o en la televisión"
¿Cuál es la importancia de esta declaración para usted?

- Extremedamente importante
- Muy importante
- Moderadamente importante
- Ligeramente importante
- No es nada importante

Usted seleccionó"... porque hace más agradables las reuniones sociales"
¿Cuál es la importancia de esta declaración para usted?

- Extremedamente importante
- Muy importante
- Moderadamente importante
- Ligeramente importante
- No es nada importante

Usted seleccionó"... porque se supone que debo comerlo"
¿Cuál es la importancia de esta declaración para usted?

- Extremedamente importante
- Muy importante
- Moderadamente importante
- Ligeramente importante
- No es nada importante

Usted seleccionó"... porque le gusta a otros"
¿Cuál es la importancia de esta declaración para usted?

- Extremedamente importante
- Muy importante
- Moderadamente importante
- Ligeramente importante
- No es nada importante

Usted seleccionó"... porque me siento solo"
¿Cuál es la importancia de esta declaración para usted?

- Extremedamente importante
- Muy importante
- Moderadamente importante
- Ligeramente importante
- No es nada importante

Usted seleccionó"... porque vigilo mi peso"
¿Cuál es la importancia de esta declaración para usted?

- Extremedamente importante
- Muy importante
- Moderadamente importante
- Ligeramente importante
- No es nada importante

Usted seleccionó"... porque no contiene sustancias peligrosas (por ejemplo, pesticidas, contaminantes, o antibióticos"
¿Cuál es la importancia de esta declaración para usted?

- Extremedamente importante
- Muy importante
- Moderadamente importante

- Ligeramente importante
- No es nada importante

Usted seleccionó"... porque es lo más conveniente"
¿Cuál es la importancia de esta declaración para usted?

- Extremedamente importante
- Muy importante
- Moderadamente importante
- Ligeramente importante
- No es nada importante

Usted seleccionó"... porque necesito energía"
¿Cuál es la importancia de esta declaración para usted?

- Extremedamente importante
- Muy importante
- Moderadamente importante
- Ligeramente importante
- No es nada importante

Usted seleccionó"... porque estoy familiarizado con ello"
¿Cuál es la importancia de esta declaración para usted?

- Extremedamente importante
- Muy importante
- Moderadamente importante
- Ligeramente importante
- No es nada importante

Usted seleccionó"... porque me gusta"
¿Cuál es la importancia de esta declaración para usted?

- Extremedamente importante
- Muy importante
- Moderadamente importante
- Ligeramente importante
- No es nada importante

Usted seleccionó"... porque me llena de un modo agradable"
¿Cuál es la importancia de esta declaración para usted?

- Extremedamente importante
- Muy importante
- Moderadamente importante
- Ligeramente importante
- No es nada importante

Usted seleccionó"... para recompensarme"
¿Cuál es la importancia de esta declaración para usted?

- Extremedamente importante
- Muy importante
- Moderadamente importante
- Ligeramente importante
- No es nada importante

Usted seleccionó"... es parte de mis tradiciones (por ejemplo, tradiciones familiares, ocasiones especiales)"
¿Cuál es la importancia de esta declaración para usted?

- Extremedamente importante
- Muy importante
- Moderadamente importante
- Ligeramente importante
- No es nada importante

Usted seleccionó"... porque se consume en ciertas ocasiones"
¿Cuál es la importancia de esta declaración para usted?

- Extremedamente importante
- Muy importante
- Moderadamente importante
- Ligeramente importante
- No es nada importante

Usted seleccionó"... para mantener una dieta equilibrada"
¿Cuál es la importancia de esta declaración para usted?

- Extremedamente importante
- Muy importante
- Moderadamente importante
- Ligeramente importante
- No es nada importante

Usted seleccionó"... porque es social"
¿Cuál es la importancia de esta declaración para usted?

- Extremedamente importante
- Muy importante
- Moderadamente importante
- Ligeramente importante
- No es nada importante

Usted seleccionó"... porque es barato"
¿Cuál es la importancia de esta declaración para usted?

- Extremedamente importante
- Muy importante
- Moderadamente importante
- Ligeramente importante

No es nada importante

Usted seleccionó"... porque me apetece de modo espontáneo (por ejemplo, colores llamativos a la vista)"
¿Cuál es la importancia de esta declaración para usted?

- Extremedamente importante
- Muy importante
- Moderadamente importante
- Ligeramente importante
- No es nada importante

Usted seleccionó"... para evitar decepcionar a alguien que está tratando de hacerme feliz"
¿Cuál es la importancia de esta declaración para usted?

- Extremedamente importante
- Muy importante
- Moderadamente importante
- Ligeramente importante
- No es nada importante

Usted seleccionó"...porque está de moda"
¿Cuál es la importancia de esta declaración para usted?

- Extremedamente importante
- Muy importante
- Moderadamente importante
- Ligeramente importante
- No es nada importante

Usted seleccionó"... porque está en rebaja"
¿Cuál es la importancia de esta declaración para usted?

- Extremedamente importante

- Muy importante
- Moderadamente importante
- Ligeramente importante
- No es nada importante

Usted seleccionó"... porque me mantiene en forma (por ejemplo, energético, motivado)"
¿Cuál es la importancia de esta declaración para usted?

- Extremedamente importante
- Muy importante
- Moderadamente importante
- Ligeramente importante
- No es nada importante

Usted seleccionó"... porque esa era la única opción"
¿Cuál es la importancia de esta declaración para usted?

- Extremedamente importante
- Muy importante
- Moderadamente importante
- Ligeramente importante
- No es nada importante

Usted seleccionó"... porque quiero comerlo todos los días"
¿Cuál es la importancia de esta declaración para usted?

- Extremedamente importante
- Muy importante
- Moderadamente importante
- Ligeramente importante
- No es nada importante

Por favor indíquenos las razones por las que usted no consume jamón serrano (Marque todas las opciones que apliquen)

- ☐ No es práctico de preparar
- ☐ No lo consumo usualmente
- ☐ Nunca lo escogería. Me gusta consumir el mismo alimento todos los días
- ☐ Me hace sentir triste, solitario, o frustrado
- ☐ No era algo que se me apetecía en ese momento
- ☐ El tamaño de la porción no era adecuado
- ☐ No me gustó la apariencia
- ☐ El precio era muy elevado
- ☐ No me gusta
- ☐ Mi generación no consume este tipo de alimento
- ☐ No lo considero como un aperitivo/snack
- ☐ Lo consumí recientemente y no quiero consumir el mismo alimento muy seguido
- ☐ Se supone que no debo consumirlo
- ☐ Es muy alto en calorías
- ☐ No es orgánico
- ☐ No era apropiado para la ocasión
- ☐ No quise consentirme
- ☐ No es un tipo de comida que consumo en presencia de otras personas
- ☐ No lo considero un alimento que llene lo suficiente
- ☐ No es saludable

Usted seleccionó"No lo considero como un aperitivo/snack"
¿Cuál es la importancia de esta declaración para usted?

- ○ Extremedamente importante
- ○ Muy importante
- ○ Moderadamente importante
- ○ Ligeramente importante
- ○ No es nada importante

Usted seleccionó"No me gusta"
¿Cuál es la importancia de esta declaración para usted?

- Extremedamente importante
- Muy importante
- Moderadamente importante
- Ligeramente importante
- No es nada importante

Usted seleccionó"No lo consumo usualmente"
¿Cuál es la importancia de esta declaración para usted?

- Extremedamente importante
- Muy importante
- Moderadamente importante
- Ligeramente importante
- No es nada importante

Usted seleccionó"El tamaño de la porción no era adecuado"
¿Cuál es la importancia de esta declaración para usted?

- Extremedamente importante
- Muy importante
- Moderadamente importante
- Ligeramente importante
- No es nada importante

Usted seleccionó"No lo considero un alimento que llene lo suficiente"
¿Cuál es la importancia de esta declaración para usted?

- Extremedamente importante
- Muy importante
- Moderadamente importante

- Ligeramente importante
- No es nada importante

Usted seleccionó"No es saludable"
¿Cuál es la importancia de esta declaración para usted?

- Extremedamente importante
- Muy importante
- Moderadamente importante
- Ligeramente importante
- No es nada importante

Usted seleccionó"No es práctico de preparar"
¿Cuál es la importancia de esta declaración para usted?

- Extremedamente importante
- Muy importante
- Moderadamente importante
- Ligeramente importante
- No es nada importante

Usted seleccionó"No quise consentirme"
¿Cuál es la importancia de esta declaración para usted?

- Extremedamente importante
- Muy importante
- Moderadamente importante
- Ligeramente importante
- No es nada importante

Usted seleccionó"No era apropiado para la ocasión"
¿Cuál es la importancia de esta declaración para usted?

- Extremedamente importante
- Muy importante
- Moderadamente importante
- Ligeramente importante
- No es nada importante

Usted seleccionó"No es un tipo de comida que consumo en presencia de otras personas"
¿Cuál es la importancia de esta declaración para usted?

- Extremedamente importante
- Muy importante
- Moderadamente importante
- Ligeramente importante
- No es nada importante

Usted seleccionó"El precio era muy elevado"
¿Cuál es la importancia de esta declaración para usted?

- Extremedamente importante
- Muy importante
- Moderadamente importante
- Ligeramente importante
- No es nada importante

Usted seleccionó"No me gustó la apariencia"
¿Cuál es la importancia de esta declaración para usted?

- Extremedamente importante
- Muy importante
- Moderadamente importante
- Ligeramente importante
- No es nada importante

Usted seleccionó"Es muy alto en calorías"
¿Cuál es la importancia de esta declaración para usted?

- Extremedamente importante
- Muy importante
- Moderadamente importante
- Ligeramente importante
- No es nada importante

Usted seleccionó"Se supone que no debo consumirlo"
¿Cuál es la importancia de esta declaración para usted?

- Extremedamente importante
- Muy importante
- Moderadamente importante
- Ligeramente importante
- No es nada importante

Usted seleccionó"Mi generación no consume este tipo de alimento"
¿Cuál es la importancia de esta declaración para usted?

- Extremedamente importante
- Muy importante
- Moderadamente importante
- Ligeramente importante
- No es nada importante

Usted seleccionó"No era algo que se me apetecía en ese momento"
¿Cuál es la importancia de esta declaración para usted?

- Extremedamente importante
- Muy importante
- Moderadamente importante
- Ligeramente importante

- No es nada importante

Usted seleccionó"Lo consumí recientemente y no quiero consumir el mismo alimento muy seguido"
¿Cuál es la importancia de esta declaración para usted?

- Extremedamente importante
- Muy importante
- Moderadamente importante
- Ligeramente importante
- No es nada importante

Usted seleccionó"Nunca lo escogería. Me gusta consumir el mismo alimento todos los días"
¿Cuál es la importancia de esta declaración para usted?

- Extremedamente importante
- Muy importante
- Moderadamente importante
- Ligeramente importante
- No es nada importante

Usted seleccionó"Me hace sentir triste, solitario, o frustrado"
¿Cuál es la importancia de esta declaración para usted?

- Extremedamente importante
- Muy importante
- Moderadamente importante
- Ligeramente importante
- No es nada importante

Usted seleccionó"No es orgánico"
¿Cuál es la importancia de esta declaración para usted?

- Extremedamente importante

- Muy importante
- Moderadamente importante
- Ligeramente importante
- No es nada importante

Reasons for Dairy Food Choices(RATA)

¿Come usted leche?

- Sí
- No

¿Cuáles fueron las razones por las que consume leche? (Marque todas las opciones que apliquen)

- ☐ ... porque me apetece
- ☐ ... porque lo he visto en anuncios o en la televisión
- ☐ ... porque sabe bien
- ☐ ... porque me siento frustrado
- ☐ ... porque no quiero gastar más dinero
- ☐ ... porque sería descortés no comerlo
- ☐ ... para evitar decepcionar a alguien que está tratando de hacerme feliz
- ☐ ... porque es rápido de preparar
- ☐ ... para poder pasar tiempo con otras personas y socializar
- ☐ ... para complacerme
- ☐ ... porque me mantiene en forma (por ejemplo, energético, motivado)
- ☐ ... porque estoy familiarizado con ello
- ☐ ... porque se consume en ciertas ocasiones
- ☐ ... es parte de mis tradiciones (por ejemplo, tradiciones familiares, ocasiones especiales)
- ☐ ... porque es natural (por ejemplo, no modificado genéticamente)

- ☐ ... porque me hace quedar bien delante de los demás
- ☐ ... porque me siento solo
- ☐ ... porque tengo hambre
- ☐ ... porque me siento triste
- ☐ ...porque está de moda
- ☐ ... porque es bajo en calorías
- ☐ ... porque me gusta
- ☐ ... porque quiero comerlo todos los días
- ☐ ... para mantener una dieta equilibrada
- ☐ ... porque lo disfruto
- ☐ ... porque la presentación es atractiva (por ejemplo, el empaque)
- ☐ ... porque crecí con ello
- ☐ ... porque es barato
- ☐ ... porque está en rebaja
- ☐ ... porque usualmente lo como
- ☐ ... porque le gusta a otros
- ☐ ... porque esa era la única opción
- ☐ ... porque hace más agradables las reuniones sociales
- ☐ ...porque es bajo en grasa
- ☐ ... porque es fácil de preparar
- ☐ ... porque me llena de un modo agradable
- ☐ ... porque es social
- ☐ ... porque me apetece de modo espontáneo (por ejemplo, colores llamativos a la vista)
- ☐ ... porque es orgánico
- ☐ ... porque estoy acostumbrado a comerlo
- ☐ ... porque vigilo mi peso
- ☐ ... porque se supone que debo comerlo
- ☐ ... porque es lo más conveniente
- ☐ ... porque no contiene sustancias peligrosas (por ejemplo, pesticidas, contaminantes, o antibióticos
- ☐ ... para recompensarme
- ☐ ... porque necesito energía

- [ ] ... porque es saludable

Usted seleccionó"... porque estoy acostumbrado a comerlo"
¿Cuál es la importancia de esta declaración para usted?

- Extremedamente importante
- Muy importante
- Moderadamente importante
- Ligeramente importante
- No es nada importante

Usted seleccionó"... porque es saludable"
¿Cuál es la importancia de esta declaración para usted?

- Extremedamente importante
- Muy importante
- Moderadamente importante
- Ligeramente importante
- No es nada importante

Usted seleccionó"... porque es rápido de preparar"
¿Cuál es la importancia de esta declaración para usted?

- Extremedamente importante
- Muy importante
- Moderadamente importante
- Ligeramente importante
- No es nada importante

Usted seleccionó"... porque sabe bien"
¿Cuál es la importancia de esta declaración para usted?

- Extremedamente importante

- Muy importante
- Moderadamente importante
- Ligeramente importante
- No es nada importante

Usted seleccionó"... para complacerme"
¿Cuál es la importancia de esta declaración para usted?

- Extremedamente importante
- Muy importante
- Moderadamente importante
- Ligeramente importante
- No es nada importante

Usted seleccionó"... porque es natural (por ejemplo, no modificado genéticamente)"
¿Cuál es la importancia de esta declaración para usted?

- Extremedamente importante
- Muy importante
- Moderadamente importante
- Ligeramente importante
- No es nada importante

Usted seleccionó"... porque no quiero gastar más dinero"
¿Cuál es la importancia de esta declaración para usted?

- Extremedamente importante
- Muy importante
- Moderadamente importante
- Ligeramente importante
- No es nada importante

Usted seleccionó"... porque es bajo en calorías"
¿Cuál es la importancia de esta declaración para usted?

- Extremedamente importante
- Muy importante
- Moderadamente importante
- Ligeramente importante
- No es nada importante

Usted seleccionó"... porque me siento frustrado"
¿Cuál es la importancia de esta declaración para usted?

- Extremedamente importante
- Muy importante
- Moderadamente importante
- Ligeramente importante
- No es nada importante

Usted seleccionó"... porque me hace quedar bien delante de los demás"
¿Cuál es la importancia de esta declaración para usted?

- Extremedamente importante
- Muy importante
- Moderadamente importante
- Ligeramente importante
- No es nada importante

Usted seleccionó"... porque sería descortés no comerlo"
¿Cuál es la importancia de esta declaración para usted?

- Extremedamente importante
- Muy importante
- Moderadamente importante
- Ligeramente importante

- No es nada importante

Usted seleccionó"... porque la presentación es atractiva (por ejemplo, el empaque)"
¿Cuál es la importancia de esta declaración para usted?

- Extremedamente importante
- Muy importante
- Moderadamente importante
- Ligeramente importante
- No es nada importante

Usted seleccionó"... para poder pasar tiempo con otras personas y socializar"
¿Cuál es la importancia de esta declaración para usted?

- Extremedamente importante
- Muy importante
- Moderadamente importante
- Ligeramente importante
- No es nada importante

Usted seleccionó"... porque es orgánico"
¿Cuál es la importancia de esta declaración para usted?

- Extremedamente importante
- Muy importante
- Moderadamente importante
- Ligeramente importante
- No es nada importante

Usted seleccionó"... porque crecí con ello"
¿Cuál es la importancia de esta declaración para usted?

- Extremedamente importante

- Muy importante
- Moderadamente importante
- Ligeramente importante
- No es nada importante

Usted seleccionó"... porque lo disfruto"
¿Cuál es la importancia de esta declaración para usted?

- Extremedamente importante
- Muy importante
- Moderadamente importante
- Ligeramente importante
- No es nada importante

Usted seleccionó"... porque tengo hambre"
¿Cuál es la importancia de esta declaración para usted?

- Extremedamente importante
- Muy importante
- Moderadamente importante
- Ligeramente importante
- No es nada importante

Usted seleccionó"... porque usualmente lo como"
¿Cuál es la importancia de esta declaración para usted?

- Extremedamente importante
- Muy importante
- Moderadamente importante
- Ligeramente importante
- No es nada importante

Usted seleccionó"... porque me apetece"
¿Cuál es la importancia de esta declaración para usted?

- Extremedamente importante
- Muy importante
- Moderadamente importante
- Ligeramente importante
- No es nada importante

Usted seleccionó"... porque es fácil de preparar"
¿Cuál es la importancia de esta declaración para usted?

- Extremedamente importante
- Muy importante
- Moderadamente importante
- Ligeramente importante
- No es nada importante

Usted seleccionó"... porque me siento triste"
¿Cuál es la importancia de esta declaración para usted?

- Extremedamente importante
- Muy importante
- Moderadamente importante
- Ligeramente importante
- No es nada importante

Usted seleccionó"...porque es bajo en grasa"
¿Cuál es la importancia de esta declaración para usted?

- Extremedamente importante
- Muy importante
- Moderadamente importante
- Ligeramente importante

- No es nada importante

Usted seleccionó"... porque lo he visto en anuncios o en la televisión"
¿Cuál es la importancia de esta declaración para usted?

- Extremedamente importante
- Muy importante
- Moderadamente importante
- Ligeramente importante
- No es nada importante

Usted seleccionó"... porque hace más agradables las reuniones sociales"
¿Cuál es la importancia de esta declaración para usted?

- Extremedamente importante
- Muy importante
- Moderadamente importante
- Ligeramente importante
- No es nada importante

Usted seleccionó"... porque se supone que debo comerlo"
¿Cuál es la importancia de esta declaración para usted?

- Extremedamente importante
- Muy importante
- Moderadamente importante
- Ligeramente importante
- No es nada importante

Usted seleccionó"... porque le gusta a otros"
¿Cuál es la importancia de esta declaración para usted?

- Extremedamente importante

- Muy importante
- Moderadamente importante
- Ligeramente importante
- No es nada importante

Usted seleccionó"... porque me siento solo"
¿Cuál es la importancia de esta declaración para usted?

- Extremedamente importante
- Muy importante
- Moderadamente importante
- Ligeramente importante
- No es nada importante

Usted seleccionó"... porque vigilo mi peso"
¿Cuál es la importancia de esta declaración para usted?

- Extremedamente importante
- Muy importante
- Moderadamente importante
- Ligeramente importante
- No es nada importante

Usted seleccionó"... porque no contiene sustancias peligrosas (por ejemplo, pesticidas, contaminantes, o antibióticos"
¿Cuál es la importancia de esta declaración para usted?

- Extremedamente importante
- Muy importante
- Moderadamente importante
- Ligeramente importante
- No es nada importante

Usted seleccionó"... porque es lo más conveniente"
¿Cuál es la importancia de esta declaración para usted?

- Extremedamente importante
- Muy importante
- Moderadamente importante
- Ligeramente importante
- No es nada importante

Usted seleccionó"... porque necesito energía"
¿Cuál es la importancia de esta declaración para usted?

- Extremedamente importante
- Muy importante
- Moderadamente importante
- Ligeramente importante
- No es nada importante

Usted seleccionó"... porque estoy familiarizado con ello"
¿Cuál es la importancia de esta declaración para usted?

- Extremedamente importante
- Muy importante
- Moderadamente importante
- Ligeramente importante
- No es nada importante

Usted seleccionó"... porque me gusta"
¿Cuál es la importancia de esta declaración para usted?

- Extremedamente importante
- Muy importante
- Moderadamente importante
- Ligeramente importante

- No es nada importante

Usted seleccionó"... porque me llena de un modo agradable"
¿Cuál es la importancia de esta declaración para usted?

- Extremedamente importante
- Muy importante
- Moderadamente importante
- Ligeramente importante
- No es nada importante

Usted seleccionó"... para recompensarme"
¿Cuál es la importancia de esta declaración para usted?

- Extremedamente importante
- Muy importante
- Moderadamente importante
- Ligeramente importante
- No es nada importante

Usted seleccionó"... es parte de mis tradiciones (por ejemplo, tradiciones familiares, ocasiones especiales)"
¿Cuál es la importancia de esta declaración para usted?

- Extremedamente importante
- Muy importante
- Moderadamente importante
- Ligeramente importante
- No es nada importante

Usted seleccionó"... porque se consume en ciertas ocasiones"
¿Cuál es la importancia de esta declaración para usted?

- Extremedamente importante

- Muy importante
- Moderadamente importante
- Ligeramente importante
- No es nada importante

Usted seleccionó"... para mantener una dieta equilibrada"
¿Cuál es la importancia de esta declaración para usted?

- Extremedamente importante
- Muy importante
- Moderadamente importante
- Ligeramente importante
- No es nada importante

Usted seleccionó"... porque es social"
¿Cuál es la importancia de esta declaración para usted?

- Extremedamente importante
- Muy importante
- Moderadamente importante
- Ligeramente importante
- No es nada importante

Usted seleccionó"... porque es barato"
¿Cuál es la importancia de esta declaración para usted?

- Extremedamente importante
- Muy importante
- Moderadamente importante
- Ligeramente importante
- No es nada importante

Usted seleccionó"... porque me apetece de modo espontáneo (por ejemplo, colores llamativos a la vista)"
¿Cuál es la importancia de esta declaración para usted?

- Extremedamente importante
- Muy importante
- Moderadamente importante
- Ligeramente importante
- No es nada importante

Usted seleccionó"... para evitar decepcionar a alguien que está tratando de hacerme feliz"
¿Cuál es la importancia de esta declaración para usted?

- Extremedamente importante
- Muy importante
- Moderadamente importante
- Ligeramente importante
- No es nada importante

Usted seleccionó"...porque está de moda"
¿Cuál es la importancia de esta declaración para usted?

- Extremedamente importante
- Muy importante
- Moderadamente importante
- Ligeramente importante
- No es nada importante

Usted seleccionó"... porque está en rebaja"
¿Cuál es la importancia de esta declaración para usted?

- Extremedamente importante
- Muy importante
- Moderadamente importante
- Ligeramente importante

○ No es nada importante

Usted seleccionó"... porque me mantiene en forma (por ejemplo, energético, motivado)"
¿Cuál es la importancia de esta declaración para usted?

○ Extremedamente importante
○ Muy importante
○ Moderadamente importante
○ Ligeramente importante
○ No es nada importante

Usted seleccionó"... porque esa era la única opción"
¿Cuál es la importancia de esta declaración para usted?

○ Extremedamente importante
○ Muy importante
○ Moderadamente importante
○ Ligeramente importante
○ No es nada importante

Usted seleccionó"... porque quiero comerlo todos los días"
¿Cuál es la importancia de esta declaración para usted?

○ Extremedamente importante
○ Muy importante
○ Moderadamente importante
○ Ligeramente importante
○ No es nada importante

Por favor indíquenos las razones por las que usted no consume leche (Marque todas las opciones que apliquen)

☐ No es práctico de preparar

- [ ] No me gustó la apariencia
- [ ] No lo considero como un aperitivo/snack
- [ ] No es saludable
- [ ] Se supone que no debo consumirlo
- [ ] No es un tipo de comida que consumo en presencia de otras personas
- [ ] El tamaño de la porción no era adecuado
- [ ] No me gusta
- [ ] No era apropiado para la ocasión
- [ ] Es muy alto en calorías
- [ ] No era algo que se me apetecía en ese momento
- [ ] No lo consumo usualmente
- [ ] Me hace sentir triste, solitario, o frustrado
- [ ] No es orgánico
- [ ] Nunca lo escogería. Me gusta consumir el mismo alimento todos los días
- [ ] Mi generación no consume este tipo de alimento
- [ ] Lo consumí recientemente y no quiero consumir el mismo alimento muy seguido
- [ ] El precio era muy elevado
- [ ] No quise consentirme
- [ ] No lo considero un alimento que llene lo suficiente

Usted seleccionó"No lo considero como un aperitivo/snack"
¿Cuál es la importancia de esta declaración para usted?

- ○ Extremedamente importante
- ○ Muy importante
- ○ Moderadamente importante
- ○ Ligeramente importante
- ○ No es nada importante

Usted seleccionó"No me gusta"
¿Cuál es la importancia de esta declaración para usted?

- ○ Extremedamente importante

- Muy importante
- Moderadamente importante
- Ligeramente importante
- No es nada importante

Usted seleccionó"No lo consumo usualmente"
¿Cuál es la importancia de esta declaración para usted?

- Extremedamente importante
- Muy importante
- Moderadamente importante
- Ligeramente importante
- No es nada importante

Usted seleccionó"El tamaño de la porción no era adecuado"
¿Cuál es la importancia de esta declaración para usted?

- Extremedamente importante
- Muy importante
- Moderadamente importante
- Ligeramente importante
- No es nada importante

Usted seleccionó"No lo considero un alimento que llene lo suficiente"
¿Cuál es la importancia de esta declaración para usted?

- Extremedamente importante
- Muy importante
- Moderadamente importante
- Ligeramente importante
- No es nada importante

Usted seleccionó"No es saludable"
¿Cuál es la importancia de esta declaración para usted?

- Extremedamente importante
- Muy importante
- Moderadamente importante
- Ligeramente importante
- No es nada importante

Usted seleccionó"No es práctico de preparar"
¿Cuál es la importancia de esta declaración para usted?

- Extremedamente importante
- Muy importante
- Moderadamente importante
- Ligeramente importante
- No es nada importante

Usted seleccionó"No quise consentirme"
¿Cuál es la importancia de esta declaración para usted?

- Extremedamente importante
- Muy importante
- Moderadamente importante
- Ligeramente importante
- No es nada importante

Usted seleccionó"No era apropiado para la ocasión"
¿Cuál es la importancia de esta declaración para usted?

- Extremedamente importante
- Muy importante
- Moderadamente importante
- Ligeramente importante

- No es nada importante

Usted seleccionó"No es un tipo de comida que consumo en presencia de otras personas"
¿Cuál es la importancia de esta declaración para usted?

- Extremedamente importante
- Muy importante
- Moderadamente importante
- Ligeramente importante
- No es nada importante

Usted seleccionó"El precio era muy elevado"
¿Cuál es la importancia de esta declaración para usted?

- Extremedamente importante
- Muy importante
- Moderadamente importante
- Ligeramente importante
- No es nada importante

Usted seleccionó"No me gustó la apariencia"
¿Cuál es la importancia de esta declaración para usted?

- Extremedamente importante
- Muy importante
- Moderadamente importante
- Ligeramente importante
- No es nada importante

Usted seleccionó"Es muy alto en calorías"
¿Cuál es la importancia de esta declaración para usted?

- Extremedamente importante

- Muy importante
- Moderadamente importante
- Ligeramente importante
- No es nada importante

Usted seleccionó"Se supone que no debo consumirlo"
¿Cuál es la importancia de esta declaración para usted?

- Extremedamente importante
- Muy importante
- Moderadamente importante
- Ligeramente importante
- No es nada importante

Usted seleccionó"Mi generación no consume este tipo de alimento"
¿Cuál es la importancia de esta declaración para usted?

- Extremedamente importante
- Muy importante
- Moderadamente importante
- Ligeramente importante
- No es nada importante

Usted seleccionó"No era algo que se me apetecía en ese momento"
¿Cuál es la importancia de esta declaración para usted?

- Extremedamente importante
- Muy importante
- Moderadamente importante
- Ligeramente importante
- No es nada importante

Usted seleccionó"Lo consumí recientemente y no quiero consumir el mismo alimento muy seguido"
¿Cuál es la importancia de esta declaración para usted?

- Extremedamente importante
- Muy importante
- Moderadamente importante
- Ligeramente importante
- No es nada importante

Usted seleccionó"Nunca lo escogería. Me gusta consumir el mismo alimento todos los días"
¿Cuál es la importancia de esta declaración para usted?

- Extremedamente importante
- Muy importante
- Moderadamente importante
- Ligeramente importante
- No es nada importante

Usted seleccionó"Me hace sentir triste, solitario, o frustrado"
¿Cuál es la importancia de esta declaración para usted?

- Extremedamente importante
- Muy importante
- Moderadamente importante
- Ligeramente importante
- No es nada importante

Usted seleccionó"No es orgánico"
¿Cuál es la importancia de esta declaración para usted?

- Extremedamente importante
- Muy importante
- Moderadamente importante
- Ligeramente importante

- No es nada importante

Reasons for Fruit Choices(RATA)

¿Come usted plátano ?

- Sí
- No

¿Cuáles fueron las razones por las que consume plátano ? (Marque todas las opciones que apliquen)

- ... porque es orgánico
- ... porque vigilo mi peso
- ... porque estoy familiarizado con ello
- ... porque se consume en ciertas ocasiones
- ... porque sería descortés no comerlo
- ... porque se supone que debo comerlo
- ... para evitar decepcionar a alguien que está tratando de hacerme feliz
- ... porque me siento triste
- ... porque me apetece de modo espontáneo (por ejemplo, colores llamativos a la vista)
- ... porque la presentación es atractiva (por ejemplo, el empaque)
- ... porque es natural (por ejemplo, no modificado genéticamente)
- ... porque hace más agradables las reuniones sociales
- ... para poder pasar tiempo con otras personas y socializar
- ... porque esa era la única opción
- ... porque es rápido de preparar
- ... porque me llena de un modo agradable
- ... para mantener una dieta equilibrada
- ... porque me mantiene en forma (por ejemplo, energético, motivado)

- ☐ ... porque no quiero gastar más dinero
- ☐ ... porque me siento frustrado
- ☐ ...porque es bajo en grasa
- ☐ ... porque me siento solo
- ☐ ... porque lo disfruto
- ☐ ... para recompensarme
- ☐ ... porque es barato
- ☐ ... porque es lo más conveniente
- ☐ ... porque le gusta a otros
- ☐ ... porque es bajo en calorías
- ☐ ... porque está en rebaja
- ☐ ... porque sabe bien
- ☐ ... porque es saludable
- ☐ ...porque está de moda
- ☐ ... porque crecí con ello
- ☐ ... porque me hace quedar bien delante de los demás
- ☐ ... porque lo he visto en anuncios o en la televisión
- ☐ ... porque tengo hambre
- ☐ ... porque estoy acostumbrado a comerlo
- ☐ ... porque me apetece
- ☐ ... para complacerme
- ☐ ... porque usualmente lo como
- ☐ ... porque es social
- ☐ ... porque me gusta
- ☐ ... porque quiero comerlo todos los días
- ☐ ... es parte de mis tradiciones (por ejemplo, tradiciones familiares, ocasiones especiales)
- ☐ ... porque no contiene sustancias peligrosas (por ejemplo, pesticidas, contaminantes, o antibióticos
- ☐ ... porque necesito energía
- ☐ ... porque es fácil de preparar

Usted seleccionó"... porque estoy acostumbrado a comerlo"
¿Cuál es la importancia de esta declaración para usted?

- Extremedamente importante
- Muy importante
- Moderadamente importante
- Ligeramente importante
- No es nada importante

Usted seleccionó"... porque es saludable"
¿Cuál es la importancia de esta declaración para usted?

- Extremedamente importante
- Muy importante
- Moderadamente importante
- Ligeramente importante
- No es nada importante

Usted seleccionó"... porque es rápido de preparar"
¿Cuál es la importancia de esta declaración para usted?

- Extremedamente importante
- Muy importante
- Moderadamente importante
- Ligeramente importante
- No es nada importante

Usted seleccionó"... porque sabe bien"
¿Cuál es la importancia de esta declaración para usted?

- Extremedamente importante
- Muy importante
- Moderadamente importante
- Ligeramente importante

- No es nada importante

Usted seleccionó"... para complacerme"
¿Cuál es la importancia de esta declaración para usted?

- Extremedamente importante
- Muy importante
- Moderadamente importante
- Ligeramente importante
- No es nada importante

Usted seleccionó"... porque es natural (por ejemplo, no modificado genéticamente)"
¿Cuál es la importancia de esta declaración para usted?

- Extremedamente importante
- Muy importante
- Moderadamente importante
- Ligeramente importante
- No es nada importante

Usted seleccionó"... porque no quiero gastar más dinero"
¿Cuál es la importancia de esta declaración para usted?

- Extremedamente importante
- Muy importante
- Moderadamente importante
- Ligeramente importante
- No es nada importante

Usted seleccionó"... porque es bajo en calorías"
¿Cuál es la importancia de esta declaración para usted?

- Extremedamente importante

- Muy importante
- Moderadamente importante
- Ligeramente importante
- No es nada importante

Usted seleccionó"... porque me siento frustrado"
¿Cuál es la importancia de esta declaración para usted?

- Extremedamente importante
- Muy importante
- Moderadamente importante
- Ligeramente importante
- No es nada importante

Usted seleccionó"... porque me hace quedar bien delante de los demás"
¿Cuál es la importancia de esta declaración para usted?

- Extremedamente importante
- Muy importante
- Moderadamente importante
- Ligeramente importante
- No es nada importante

Usted seleccionó"... porque sería descortés no comerlo"
¿Cuál es la importancia de esta declaración para usted?

- Extremedamente importante
- Muy importante
- Moderadamente importante
- Ligeramente importante
- No es nada importante

Usted seleccionó"... porque la presentación es atractiva (por ejemplo, el empaque)"
¿Cuál es la importancia de esta declaración para usted?

- Extremedamente importante
- Muy importante
- Moderadamente importante
- Ligeramente importante
- No es nada importante

Usted seleccionó"... para poder pasar tiempo con otras personas y socializar"
¿Cuál es la importancia de esta declaración para usted?

- Extremedamente importante
- Muy importante
- Moderadamente importante
- Ligeramente importante
- No es nada importante

Usted seleccionó"... porque es orgánico"
¿Cuál es la importancia de esta declaración para usted?

- Extremedamente importante
- Muy importante
- Moderadamente importante
- Ligeramente importante
- No es nada importante

Usted seleccionó"... porque crecí con ello"
¿Cuál es la importancia de esta declaración para usted?

- Extremedamente importante
- Muy importante
- Moderadamente importante
- Ligeramente importante

◯ No es nada importante

Usted seleccionó"... porque lo disfruto"
¿Cuál es la importancia de esta declaración para usted?

◯ Extremedamente importante
◯ Muy importante
◯ Moderadamente importante
◯ Ligeramente importante
◯ No es nada importante

Usted seleccionó"... porque tengo hambre"
¿Cuál es la importancia de esta declaración para usted?

◯ Extremedamente importante
◯ Muy importante
◯ Moderadamente importante
◯ Ligeramente importante
◯ No es nada importante

Usted seleccionó"... porque usualmente lo como"
¿Cuál es la importancia de esta declaración para usted?

◯ Extremedamente importante
◯ Muy importante
◯ Moderadamente importante
◯ Ligeramente importante
◯ No es nada importante

Usted seleccionó"... porque me apetece"
¿Cuál es la importancia de esta declaración para usted?

◯ Extremedamente importante

- Muy importante
- Moderadamente importante
- Ligeramente importante
- No es nada importante

Usted seleccionó"... porque es fácil de preparar"
¿Cuál es la importancia de esta declaración para usted?

- Extremedamente importante
- Muy importante
- Moderadamente importante
- Ligeramente importante
- No es nada importante

Usted seleccionó"... porque me siento triste"
¿Cuál es la importancia de esta declaración para usted?

- Extremedamente importante
- Muy importante
- Moderadamente importante
- Ligeramente importante
- No es nada importante

Usted seleccionó"...porque es bajo en grasa"
¿Cuál es la importancia de esta declaración para usted?

- Extremedamente importante
- Muy importante
- Moderadamente importante
- Ligeramente importante
- No es nada importante

Usted seleccionó"... porque lo he visto en anuncios o en la televisión"
¿Cuál es la importancia de esta declaración para usted?

- Extremedamente importante
- Muy importante
- Moderadamente importante
- Ligeramente importante
- No es nada importante

Usted seleccionó"... porque hace más agradables las reuniones sociales"
¿Cuál es la importancia de esta declaración para usted?

- Extremedamente importante
- Muy importante
- Moderadamente importante
- Ligeramente importante
- No es nada importante

Usted seleccionó"... porque se supone que debo comerlo"
¿Cuál es la importancia de esta declaración para usted?

- Extremedamente importante
- Muy importante
- Moderadamente importante
- Ligeramente importante
- No es nada importante

Usted seleccionó"... porque le gusta a otros"
¿Cuál es la importancia de esta declaración para usted?

- Extremedamente importante
- Muy importante
- Moderadamente importante
- Ligeramente importante

- No es nada importante

Usted seleccionó"... porque me siento solo"
¿Cuál es la importancia de esta declaración para usted?

- Extremedamente importante
- Muy importante
- Moderadamente importante
- Ligeramente importante
- No es nada importante

Usted seleccionó"... porque vigilo mi peso"
¿Cuál es la importancia de esta declaración para usted?

- Extremedamente importante
- Muy importante
- Moderadamente importante
- Ligeramente importante
- No es nada importante

Usted seleccionó"... porque no contiene sustancias peligrosas (por ejemplo, pesticidas, contaminantes, o antibióticos"
¿Cuál es la importancia de esta declaración para usted?

- Extremedamente importante
- Muy importante
- Moderadamente importante
- Ligeramente importante
- No es nada importante

Usted seleccionó"... porque es lo más conveniente"
¿Cuál es la importancia de esta declaración para usted?

- Extremedamente importante
- Muy importante
- Moderadamente importante
- Ligeramente importante
- No es nada importante

Usted seleccionó"... porque necesito energía"
¿Cuál es la importancia de esta declaración para usted?

- Extremedamente importante
- Muy importante
- Moderadamente importante
- Ligeramente importante
- No es nada importante

Usted seleccionó"... porque estoy familiarizado con ello"
¿Cuál es la importancia de esta declaración para usted?

- Extremedamente importante
- Muy importante
- Moderadamente importante
- Ligeramente importante
- No es nada importante

Usted seleccionó"... porque me gusta"
¿Cuál es la importancia de esta declaración para usted?

- Extremedamente importante
- Muy importante
- Moderadamente importante
- Ligeramente importante
- No es nada importante

Usted seleccionó"... porque me llena de un modo agradable"
¿Cuál es la importancia de esta declaración para usted?

- Extremedamente importante
- Muy importante
- Moderadamente importante
- Ligeramente importante
- No es nada importante

Usted seleccionó"... para recompensarme"
¿Cuál es la importancia de esta declaración para usted?

- Extremedamente importante
- Muy importante
- Moderadamente importante
- Ligeramente importante
- No es nada importante

Usted seleccionó"... es parte de mis tradiciones (por ejemplo, tradiciones familiares, ocasiones especiales)"
¿Cuál es la importancia de esta declaración para usted?

- Extremedamente importante
- Muy importante
- Moderadamente importante
- Ligeramente importante
- No es nada importante

Usted seleccionó"... porque se consume en ciertas ocasiones"
¿Cuál es la importancia de esta declaración para usted?

- Extremedamente importante
- Muy importante
- Moderadamente importante
- Ligeramente importante

No es nada importante

Usted seleccionó"... para mantener una dieta equilibrada"
¿Cuál es la importancia de esta declaración para usted?

- Extremedamente importante
- Muy importante
- Moderadamente importante
- Ligeramente importante
- No es nada importante

Usted seleccionó"... porque es social"
¿Cuál es la importancia de esta declaración para usted?

- Extremedamente importante
- Muy importante
- Moderadamente importante
- Ligeramente importante
- No es nada importante

Usted seleccionó"... porque es barato"
¿Cuál es la importancia de esta declaración para usted?

- Extremedamente importante
- Muy importante
- Moderadamente importante
- Ligeramente importante
- No es nada importante

Usted seleccionó"... porque me apetece de modo espontáneo (por ejemplo, colores llamativos a la vista)"
¿Cuál es la importancia de esta declaración para usted?

- Extremedamente importante

- Muy importante
- Moderadamente importante
- Ligeramente importante
- No es nada importante

Usted seleccionó"... para evitar decepcionar a alguien que está tratando de hacerme feliz"
¿Cuál es la importancia de esta declaración para usted?

- Extremedamente importante
- Muy importante
- Moderadamente importante
- Ligeramente importante
- No es nada importante

Usted seleccionó"...porque está de moda"
¿Cuál es la importancia de esta declaración para usted?

- Extremedamente importante
- Muy importante
- Moderadamente importante
- Ligeramente importante
- No es nada importante

Usted seleccionó"... porque está en rebaja"
¿Cuál es la importancia de esta declaración para usted?

- Extremedamente importante
- Muy importante
- Moderadamente importante
- Ligeramente importante
- No es nada importante

Usted seleccionó"... porque me mantiene en forma (por ejemplo, energético, motivado)"
¿Cuál es la importancia de esta declaración para usted?

- Extremedamente importante
- Muy importante
- Moderadamente importante
- Ligeramente importante
- No es nada importante

Usted seleccionó"... porque esa era la única opción"
¿Cuál es la importancia de esta declaración para usted?

- Extremedamente importante
- Muy importante
- Moderadamente importante
- Ligeramente importante
- No es nada importante

Usted seleccionó"... porque quiero comerlo todos los días"
¿Cuál es la importancia de esta declaración para usted?

- Extremedamente importante
- Muy importante
- Moderadamente importante
- Ligeramente importante
- No es nada importante

Por favor indíquenos las razones por las que usted no consume plátano (Marque todas las opciones que apliquen)

- [ ] No es un tipo de comida que consumo en presencia de otras personas
- [ ] No lo consumo usualmente
- [ ] No me gustó la apariencia

- ☐ No es orgánico
- ☐ El precio era muy elevado
- ☐ No es práctico de preparar
- ☐ El tamaño de la porción no era adecuado
- ☐ No era apropiado para la ocasión
- ☐ No me gusta
- ☐ Se supone que no debo consumirlo
- ☐ Es muy alto en calorías
- ☐ Mi generación no consume este tipo de alimento
- ☐ No lo considero como un aperitivo/snack
- ☐ No es saludable
- ☐ No quise consentirme
- ☐ No era algo que se me apetecía en ese momento
- ☐ Nunca lo escogería. Me gusta consumir el mismo alimento todos los días
- ☐ No lo considero un alimento que llene lo suficiente
- ☐ Lo consumí recientemente y no quiero consumir el mismo alimento muy seguido
- ☐ Me hace sentir triste, solitario, o frustrado

Usted seleccionó"No lo considero como un aperitivo/snack"
¿Cuál es la importancia de esta declaración para usted?

- ○ Extremedamente importante
- ○ Muy importante
- ○ Moderadamente importante
- ○ Ligeramente importante
- ○ No es nada importante

Usted seleccionó"No me gusta"
¿Cuál es la importancia de esta declaración para usted?

- ○ Extremedamente importante
- ○ Muy importante
- ○ Moderadamente importante

- Ligeramente importante
- No es nada importante

Usted seleccionó"No lo consumo usualmente"
¿Cuál es la importancia de esta declaración para usted?

- Extremedamente importante
- Muy importante
- Moderadamente importante
- Ligeramente importante
- No es nada importante

Usted seleccionó"El tamaño de la porción no era adecuado"
¿Cuál es la importancia de esta declaración para usted?

- Extremedamente importante
- Muy importante
- Moderadamente importante
- Ligeramente importante
- No es nada importante

Usted seleccionó"No lo considero un alimento que llene lo suficiente"
¿Cuál es la importancia de esta declaración para usted?

- Extremedamente importante
- Muy importante
- Moderadamente importante
- Ligeramente importante
- No es nada importante

Usted seleccionó"No es saludable"
¿Cuál es la importancia de esta declaración para usted?

- Extremedamente importante
- Muy importante
- Moderadamente importante
- Ligeramente importante
- No es nada importante

Usted seleccionó"No es práctico de preparar"
¿Cuál es la importancia de esta declaración para usted?

- Extremedamente importante
- Muy importante
- Moderadamente importante
- Ligeramente importante
- No es nada importante

Usted seleccionó"No quise consentirme"
¿Cuál es la importancia de esta declaración para usted?

- Extremedamente importante
- Muy importante
- Moderadamente importante
- Ligeramente importante
- No es nada importante

Usted seleccionó"No era apropiado para la ocasión"
¿Cuál es la importancia de esta declaración para usted?

- Extremedamente importante
- Muy importante
- Moderadamente importante
- Ligeramente importante
- No es nada importante

Usted seleccionó"No es un tipo de comida que consumo en presencia de otras personas"
¿Cuál es la importancia de esta declaración para usted?

- Extremedamente importante
- Muy importante
- Moderadamente importante
- Ligeramente importante
- No es nada importante

Usted seleccionó"El precio era muy elevado"
¿Cuál es la importancia de esta declaración para usted?

- Extremedamente importante
- Muy importante
- Moderadamente importante
- Ligeramente importante
- No es nada importante

Usted seleccionó"No me gustó la apariencia"
¿Cuál es la importancia de esta declaración para usted?

- Extremedamente importante
- Muy importante
- Moderadamente importante
- Ligeramente importante
- No es nada importante

Usted seleccionó"Es muy alto en calorías"
¿Cuál es la importancia de esta declaración para usted?

- Extremedamente importante
- Muy importante
- Moderadamente importante
- Ligeramente importante

No es nada importante

Usted seleccionó"Se supone que no debo consumirlo"
¿Cuál es la importancia de esta declaración para usted?

- Extremedamente importante
- Muy importante
- Moderadamente importante
- Ligeramente importante
- No es nada importante

Usted seleccionó"Mi generación no consume este tipo de alimento"
¿Cuál es la importancia de esta declaración para usted?

- Extremedamente importante
- Muy importante
- Moderadamente importante
- Ligeramente importante
- No es nada importante

Usted seleccionó"No era algo que se me apetecía en ese momento"
¿Cuál es la importancia de esta declaración para usted?

- Extremedamente importante
- Muy importante
- Moderadamente importante
- Ligeramente importante
- No es nada importante

Usted seleccionó"Lo consumí recientemente y no quiero consumir el mismo alimento muy seguido"
¿Cuál es la importancia de esta declaración para usted?

- Extremedamente importante

- Muy importante
- Moderadamente importante
- Ligeramente importante
- No es nada importante

Usted seleccionó"Nunca lo escogería. Me gusta consumir el mismo alimento todos los días"
¿Cuál es la importancia de esta declaración para usted?

- Extremedamente importante
- Muy importante
- Moderadamente importante
- Ligeramente importante
- No es nada importante

Usted seleccionó"Me hace sentir triste, solitario, o frustrado"
¿Cuál es la importancia de esta declaración para usted?

- Extremedamente importante
- Muy importante
- Moderadamente importante
- Ligeramente importante
- No es nada importante

Usted seleccionó"No es orgánico"
¿Cuál es la importancia de esta declaración para usted?

- Extremedamente importante
- Muy importante
- Moderadamente importante
- Ligeramente importante
- No es nada importante

Reasons for Sweet food Choices(RATA)

¿Come usted turrón ?

- Sí
- No

¿Cuáles fueron las razones por las que consume turrón ? (Marque todas las opciones que apliquen)

- ☐ ... porque es social
- ☐ ... porque crecí con ello
- ☐ ... para evitar decepcionar a alguien que está tratando de hacerme feliz
- ☐ ... porque sería descortés no comerlo
- ☐ ... porque es fácil de preparar
- ☐ ... porque es barato
- ☐ ... porque me siento frustrado
- ☐ ... porque quiero comerlo todos los días
- ☐ ... porque la presentación es atractiva (por ejemplo, el empaque)
- ☐ ... porque me siento triste
- ☐ ... porque es lo más conveniente
- ☐ ... porque me hace quedar bien delante de los demás
- ☐ ... porque se supone que debo comerlo
- ☐ ... porque me llena de un modo agradable
- ☐ ...porque está de moda
- ☐ ... porque es orgánico
- ☐ ... para mantener una dieta equilibrada
- ☐ ... porque me apetece de modo espontáneo (por ejemplo, colores llamativos a la vista)
- ☐ ... porque no contiene sustancias peligrosas (por ejemplo, pesticidas, contaminantes, o antibióticos
- ☐ ... porque estoy familiarizado con ello

- ☐ ... porque es saludable
- ☐ ... porque hace más agradables las reuniones sociales
- ☐ ... porque me mantiene en forma (por ejemplo, energético, motivado)
- ☐ ... porque le gusta a otros
- ☐ ... porque tengo hambre
- ☐ ... es parte de mis tradiciones (por ejemplo, tradiciones familiares, ocasiones especiales)
- ☐ ... porque es bajo en calorías
- ☐ ...porque es bajo en grasa
- ☐ ... porque vigilo mi peso
- ☐ ... porque necesito energía
- ☐ ... para recompensarme
- ☐ ... porque usualmente lo como
- ☐ ... porque está en rebaja
- ☐ ... porque me siento solo
- ☐ ... porque me apetece
- ☐ ... porque estoy acostumbrado a comerlo
- ☐ ... porque esa era la única opción
- ☐ ... para complacerme
- ☐ ... para poder pasar tiempo con otras personas y socializar
- ☐ ... porque se consume en ciertas ocasiones
- ☐ ... porque no quiero gastar más dinero
- ☐ ... porque sabe bien
- ☐ ... porque me gusta
- ☐ ... porque lo disfruto
- ☐ ... porque es natural (por ejemplo, no modificado genéticamente)
- ☐ ... porque es rápido de preparar
- ☐ ... porque lo he visto en anuncios o en la televisión

Usted seleccionó"... porque estoy acostumbrado a comerlo"
¿Cuál es la importancia de esta declaración para usted?

- ○ Extremedamente importante

- Muy importante
- Moderadamente importante
- Ligeramente importante
- No es nada importante

Usted seleccionó"... porque es saludable"
¿Cuál es la importancia de esta declaración para usted?

- Extremedamente importante
- Muy importante
- Moderadamente importante
- Ligeramente importante
- No es nada importante

Usted seleccionó"... porque es rápido de preparar"
¿Cuál es la importancia de esta declaración para usted?

- Extremedamente importante
- Muy importante
- Moderadamente importante
- Ligeramente importante
- No es nada importante

Usted seleccionó"... porque sabe bien"
¿Cuál es la importancia de esta declaración para usted?

- Extremedamente importante
- Muy importante
- Moderadamente importante
- Ligeramente importante
- No es nada importante

Usted seleccionó"... para complacerme"
¿Cuál es la importancia de esta declaración para usted?

- Extremedamente importante
- Muy importante
- Moderadamente importante
- Ligeramente importante
- No es nada importante

Usted seleccionó"... porque es natural (por ejemplo, no modificado genéticamente)"
¿Cuál es la importancia de esta declaración para usted?

- Extremedamente importante
- Muy importante
- Moderadamente importante
- Ligeramente importante
- No es nada importante

Usted seleccionó"... porque no quiero gastar más dinero"
¿Cuál es la importancia de esta declaración para usted?

- Extremedamente importante
- Muy importante
- Moderadamente importante
- Ligeramente importante
- No es nada importante

Usted seleccionó"... porque es bajo en calorías"
¿Cuál es la importancia de esta declaración para usted?

- Extremedamente importante
- Muy importante
- Moderadamente importante
- Ligeramente importante

- No es nada importante

Usted seleccionó"... porque me siento frustrado"
¿Cuál es la importancia de esta declaración para usted?

- Extremedamente importante
- Muy importante
- Moderadamente importante
- Ligeramente importante
- No es nada importante

Usted seleccionó"... porque me hace quedar bien delante de los demás"
¿Cuál es la importancia de esta declaración para usted?

- Extremedamente importante
- Muy importante
- Moderadamente importante
- Ligeramente importante
- No es nada importante

Usted seleccionó"... porque sería descortés no comerlo"
¿Cuál es la importancia de esta declaración para usted?

- Extremedamente importante
- Muy importante
- Moderadamente importante
- Ligeramente importante
- No es nada importante

Usted seleccionó"... porque la presentación es atractiva (por ejemplo, el empaque)"
¿Cuál es la importancia de esta declaración para usted?

- Extremedamente importante

- Muy importante
- Moderadamente importante
- Ligeramente importante
- No es nada importante

Usted seleccionó"... para poder pasar tiempo con otras personas y socializar"
¿Cuál es la importancia de esta declaración para usted?

- Extremedamente importante
- Muy importante
- Moderadamente importante
- Ligeramente importante
- No es nada importante

Usted seleccionó"... porque es orgánico"
¿Cuál es la importancia de esta declaración para usted?

- Extremedamente importante
- Muy importante
- Moderadamente importante
- Ligeramente importante
- No es nada importante

Usted seleccionó"... porque crecí con ello"
¿Cuál es la importancia de esta declaración para usted?

- Extremedamente importante
- Muy importante
- Moderadamente importante
- Ligeramente importante
- No es nada importante

Usted seleccionó"... porque lo disfruto"
¿Cuál es la importancia de esta declaración para usted?

- Extremedamente importante
- Muy importante
- Moderadamente importante
- Ligeramente importante
- No es nada importante

Usted seleccionó"... porque tengo hambre"
¿Cuál es la importancia de esta declaración para usted?

- Extremedamente importante
- Muy importante
- Moderadamente importante
- Ligeramente importante
- No es nada importante

Usted seleccionó"... porque usualmente lo como"
¿Cuál es la importancia de esta declaración para usted?

- Extremedamente importante
- Muy importante
- Moderadamente importante
- Ligeramente importante
- No es nada importante

Usted seleccionó"... porque me apetece"
¿Cuál es la importancia de esta declaración para usted?

- Extremedamente importante
- Muy importante
- Moderadamente importante
- Ligeramente importante

- No es nada importante

Usted seleccionó"... porque es fácil de preparar"
¿Cuál es la importancia de esta declaración para usted?

- Extremedamente importante
- Muy importante
- Moderadamente importante
- Ligeramente importante
- No es nada importante

Usted seleccionó"... porque me siento triste"
¿Cuál es la importancia de esta declaración para usted?

- Extremedamente importante
- Muy importante
- Moderadamente importante
- Ligeramente importante
- No es nada importante

Usted seleccionó"...porque es bajo en grasa"
¿Cuál es la importancia de esta declaración para usted?

- Extremedamente importante
- Muy importante
- Moderadamente importante
- Ligeramente importante
- No es nada importante

Usted seleccionó"... porque lo he visto en anuncios o en la televisión"
¿Cuál es la importancia de esta declaración para usted?

- Extremedamente importante

- Muy importante
- Moderadamente importante
- Ligeramente importante
- No es nada importante

Usted seleccionó"... porque hace más agradables las reuniones sociales"
¿Cuál es la importancia de esta declaración para usted?

- Extremedamente importante
- Muy importante
- Moderadamente importante
- Ligeramente importante
- No es nada importante

Usted seleccionó"... porque se supone que debo comerlo"
¿Cuál es la importancia de esta declaración para usted?

- Extremedamente importante
- Muy importante
- Moderadamente importante
- Ligeramente importante
- No es nada importante

Usted seleccionó"... porque le gusta a otros"
¿Cuál es la importancia de esta declaración para usted?

- Extremedamente importante
- Muy importante
- Moderadamente importante
- Ligeramente importante
- No es nada importante

Usted seleccionó"... porque me siento solo"
¿Cuál es la importancia de esta declaración para usted?

- Extremedamente importante
- Muy importante
- Moderadamente importante
- Ligeramente importante
- No es nada importante

Usted seleccionó"... porque vigilo mi peso"
¿Cuál es la importancia de esta declaración para usted?

- Extremedamente importante
- Muy importante
- Moderadamente importante
- Ligeramente importante
- No es nada importante

Usted seleccionó"... porque no contiene sustancias peligrosas (por ejemplo, pesticidas, contaminantes, o antibióticos"
¿Cuál es la importancia de esta declaración para usted?

- Extremedamente importante
- Muy importante
- Moderadamente importante
- Ligeramente importante
- No es nada importante

Usted seleccionó"... porque es lo más conveniente"
¿Cuál es la importancia de esta declaración para usted?

- Extremedamente importante
- Muy importante
- Moderadamente importante

- Ligeramente importante
- No es nada importante

Usted seleccionó"... porque necesito energía"
¿Cuál es la importancia de esta declaración para usted?

- Extremedamente importante
- Muy importante
- Moderadamente importante
- Ligeramente importante
- No es nada importante

Usted seleccionó"... porque estoy familiarizado con ello"
¿Cuál es la importancia de esta declaración para usted?

- Extremedamente importante
- Muy importante
- Moderadamente importante
- Ligeramente importante
- No es nada importante

Usted seleccionó"... porque me gusta"
¿Cuál es la importancia de esta declaración para usted?

- Extremedamente importante
- Muy importante
- Moderadamente importante
- Ligeramente importante
- No es nada importante

Usted seleccionó"... porque me llena de un modo agradable"
¿Cuál es la importancia de esta declaración para usted?

- Extremedamente importante
- Muy importante
- Moderadamente importante
- Ligeramente importante
- No es nada importante

Usted seleccionó"... para recompensarme"
¿Cuál es la importancia de esta declaración para usted?

- Extremedamente importante
- Muy importante
- Moderadamente importante
- Ligeramente importante
- No es nada importante

Usted seleccionó"... es parte de mis tradiciones (por ejemplo, tradiciones familiares, ocasiones especiales)"
¿Cuál es la importancia de esta declaración para usted?

- Extremedamente importante
- Muy importante
- Moderadamente importante
- Ligeramente importante
- No es nada importante

Usted seleccionó"... porque se consume en ciertas ocasiones"
¿Cuál es la importancia de esta declaración para usted?

- Extremedamente importante
- Muy importante
- Moderadamente importante
- Ligeramente importante
- No es nada importante

Usted seleccionó"... para mantener una dieta equilibrada"
¿Cuál es la importancia de esta declaración para usted?

- Extremedamente importante
- Muy importante
- Moderadamente importante
- Ligeramente importante
- No es nada importante

Usted seleccionó"... porque es social"
¿Cuál es la importancia de esta declaración para usted?

- Extremedamente importante
- Muy importante
- Moderadamente importante
- Ligeramente importante
- No es nada importante

Usted seleccionó"... porque es barato"
¿Cuál es la importancia de esta declaración para usted?

- Extremedamente importante
- Muy importante
- Moderadamente importante
- Ligeramente importante
- No es nada importante

Usted seleccionó"... porque me apetece de modo espontáneo (por ejemplo, colores llamativos a la vista)"
¿Cuál es la importancia de esta declaración para usted?

- Extremedamente importante
- Muy importante
- Moderadamente importante
- Ligeramente importante

- No es nada importante

Usted seleccionó"... para evitar decepcionar a alguien que está tratando de hacerme feliz"
¿Cuál es la importancia de esta declaración para usted?

- Extremedamente importante
- Muy importante
- Moderadamente importante
- Ligeramente importante
- No es nada importante

Usted seleccionó"...porque está de moda"
¿Cuál es la importancia de esta declaración para usted?

- Extremedamente importante
- Muy importante
- Moderadamente importante
- Ligeramente importante
- No es nada importante

Usted seleccionó"... porque está en rebaja"
¿Cuál es la importancia de esta declaración para usted?

- Extremedamente importante
- Muy importante
- Moderadamente importante
- Ligeramente importante
- No es nada importante

Usted seleccionó"... porque me mantiene en forma (por ejemplo, energético, motivado)"
¿Cuál es la importancia de esta declaración para usted?

- Extremedamente importante

- Muy importante
- Moderadamente importante
- Ligeramente importante
- No es nada importante

Usted seleccionó"... porque esa era la única opción"
¿Cuál es la importancia de esta declaración para usted?

- Extremedamente importante
- Muy importante
- Moderadamente importante
- Ligeramente importante
- No es nada importante

Usted seleccionó"... porque quiero comerlo todos los días"
¿Cuál es la importancia de esta declaración para usted?

- Extremedamente importante
- Muy importante
- Moderadamente importante
- Ligeramente importante
- No es nada importante

Por favor indíquenos las razones por las que usted no consume turrón (Marque todas las opciones que apliquen)

- ☐ No me gusta
- ☐ No es práctico de preparar
- ☐ Se supone que no debo consumirlo
- ☐ No lo considero como un aperitivo/snack
- ☐ No lo consumo usualmente
- ☐ Mi generación no consume este tipo de alimento
- ☐ No lo considero un alimento que llene lo suficiente

- ☐ El tamaño de la porción no era adecuado
- ☐ No era algo que se me apetecía en ese momento
- ☐ El precio era muy elevado
- ☐ Me hace sentir triste, solitario, o frustrado
- ☐ Lo consumí recientemente y no quiero consumir el mismo alimento muy seguido
- ☐ No quise consentirme
- ☐ No es un tipo de comida que consumo en presencia de otras personas
- ☐ Es muy alto en calorías
- ☐ No es orgánico
- ☐ Nunca lo escogería. Me gusta consumir el mismo alimento todos los días
- ☐ No es saludable
- ☐ No me gustó la apariencia
- ☐ No era apropiado para la ocasión

Usted seleccionó"No lo considero como un aperitivo/snack"
¿Cuál es la importancia de esta declaración para usted?

- ○ Extremedamente importante
- ○ Muy importante
- ○ Moderadamente importante
- ○ Ligeramente importante
- ○ No es nada importante

Usted seleccionó"No me gusta"
¿Cuál es la importancia de esta declaración para usted?

- ○ Extremedamente importante
- ○ Muy importante
- ○ Moderadamente importante
- ○ Ligeramente importante
- ○ No es nada importante

Usted seleccionó"No lo consumo usualmente"
¿Cuál es la importancia de esta declaración para usted?

- Extremedamente importante
- Muy importante
- Moderadamente importante
- Ligeramente importante
- No es nada importante

Usted seleccionó"El tamaño de la porción no era adecuado"
¿Cuál es la importancia de esta declaración para usted?

- Extremedamente importante
- Muy importante
- Moderadamente importante
- Ligeramente importante
- No es nada importante

Usted seleccionó"No lo considero un alimento que llene lo suficiente"
¿Cuál es la importancia de esta declaración para usted?

- Extremedamente importante
- Muy importante
- Moderadamente importante
- Ligeramente importante
- No es nada importante

Usted seleccionó"No es saludable"
¿Cuál es la importancia de esta declaración para usted?

- Extremedamente importante
- Muy importante
- Moderadamente importante
- Ligeramente importante

- No es nada importante

Usted seleccionó"No es práctico de preparar"
¿Cuál es la importancia de esta declaración para usted?

- Extremedamente importante
- Muy importante
- Moderadamente importante
- Ligeramente importante
- No es nada importante

Usted seleccionó"No quise consentirme"
¿Cuál es la importancia de esta declaración para usted?

- Extremedamente importante
- Muy importante
- Moderadamente importante
- Ligeramente importante
- No es nada importante

Usted seleccionó"No era apropiado para la ocasión"
¿Cuál es la importancia de esta declaración para usted?

- Extremedamente importante
- Muy importante
- Moderadamente importante
- Ligeramente importante
- No es nada importante

Usted seleccionó"No es un tipo de comida que consumo en presencia de otras personas"
¿Cuál es la importancia de esta declaración para usted?

- Extremedamente importante

- Muy importante
- Moderadamente importante
- Ligeramente importante
- No es nada importante

Usted seleccionó"El precio era muy elevado"
¿Cuál es la importancia de esta declaración para usted?

- Extremedamente importante
- Muy importante
- Moderadamente importante
- Ligeramente importante
- No es nada importante

Usted seleccionó"No me gustó la apariencia"
¿Cuál es la importancia de esta declaración para usted?

- Extremedamente importante
- Muy importante
- Moderadamente importante
- Ligeramente importante
- No es nada importante

Usted seleccionó"Es muy alto en calorías"
¿Cuál es la importancia de esta declaración para usted?

- Extremedamente importante
- Muy importante
- Moderadamente importante
- Ligeramente importante
- No es nada importante

Usted seleccionó"Se supone que no debo consumirlo"
¿Cuál es la importancia de esta declaración para usted?

- Extremedamente importante
- Muy importante
- Moderadamente importante
- Ligeramente importante
- No es nada importante

Usted seleccionó"Mi generación no consume este tipo de alimento"
¿Cuál es la importancia de esta declaración para usted?

- Extremedamente importante
- Muy importante
- Moderadamente importante
- Ligeramente importante
- No es nada importante

Usted seleccionó"No era algo que se me apetecía en ese momento"
¿Cuál es la importancia de esta declaración para usted?

- Extremedamente importante
- Muy importante
- Moderadamente importante
- Ligeramente importante
- No es nada importante

Usted seleccionó"Lo consumí recientemente y no quiero consumir el mismo alimento muy seguido"
¿Cuál es la importancia de esta declaración para usted?

- Extremedamente importante
- Muy importante
- Moderadamente importante
- Ligeramente importante

○ No es nada importante

Usted seleccionó"Nunca lo escogería. Me gusta consumir el mismo alimento todos los días"
¿Cuál es la importancia de esta declaración para usted?

○ Extremedamente importante
○ Muy importante
○ Moderadamente importante
○ Ligeramente importante
○ No es nada importante

Usted seleccionó"Me hace sentir triste, solitario, o frustrado"
¿Cuál es la importancia de esta declaración para usted?

○ Extremedamente importante
○ Muy importante
○ Moderadamente importante
○ Ligeramente importante
○ No es nada importante

Usted seleccionó"No es orgánico"
¿Cuál es la importancia de esta declaración para usted?

○ Extremedamente importante
○ Muy importante
○ Moderadamente importante
○ Ligeramente importante
○ No es nada importante

Reasons for Starchy food choices(Full RATA)

¿Come usted paella?

- Sí
- No

Pensando en las razones por las que usted consume paella.

¿Cuál es la importancia de esta declaración para usted? "...porque estoy acostumbrado a comerlo "

- Extremedamente importante
- Muy importante
- Moderadamente importante
- Ligeramente importante
- No es nada importante

Pensando en las razones por las que usted consume paella.

¿Cuál es la importancia de esta declaración para usted? "...porque es saludable"

- Extremedamente importante
- Muy importante
- Moderadamente importante
- Ligeramente importante
- No es nada importante

Pensando en las razones por las que usted consume paella.

¿Cuál es la importancia de esta declaración para usted? "...porque es rápido de preparar"

- Extremedamente importante
- Muy importante
- Moderadamente importante
- Ligeramente importante
- No es nada importante

Pensando en las razones por las que usted consume paella.

¿Cuál es la importancia de esta declaración para usted? "...porque sabe bien"

- Extremedamente importante
- Muy importante
- Moderadamente importante
- Ligeramente importante
- No es nada importante

Pensando en las razones por las que usted consume paella.
¿Cuál es la importancia de esta declaración para usted? "...para complacerme"

- Extremedamente importante
- Muy importante
- Moderadamente importante
- Ligeramente importante
- No es nada importante

Pensando en las razones por las que usted consume paella.
¿Cuál es la importancia de esta declaración para usted? "...porque es natural (por ejemplo, no modificado genéticamente)"

- Extremedamente importante
- Muy importante
- Moderadamente importante
- Ligeramente importante
- No es nada importante

Pensando en las razones por las que usted consume paella.
¿Cuál es la importancia de esta declaración para usted? "...porque no quiero gastar más dinero"

- Extremedamente importante
- Muy importante
- Moderadamente importante

- Ligeramente importante
- No es nada importante

Pensando en las razones por las que usted consume paella.

¿Cuál es la importancia de esta declaración para usted? "...porque es bajo en calorías"

- Extremedamente importante
- Muy importante
- Moderadamente importante
- Ligeramente importante
- No es nada importante

Pensando en las razones por las que usted consume paella.

¿Cuál es la importancia de esta declaración para usted? "...porque me siento frustrado"

- Extremedamente importante
- Muy importante
- Moderadamente importante
- Ligeramente importante
- No es nada importante

Pensando en las razones por las que usted consume paella.

¿Cuál es la importancia de esta declaración para usted? "...porque me hace quedar bien delante de los demás"

- Extremedamente importante
- Muy importante
- Moderadamente importante
- Ligeramente importante
- No es nada importante

Pensando en las razones por las que usted consume paella.
¿Cuál es la importancia de esta declaración para usted? "...porque sería descortés no comerlo"

- Extremedamente importante
- Muy importante
- Moderadamente importante
- Ligeramente importante
- No es nada importante

Pensando en las razones por las que usted consume paella.
¿Cuál es la importancia de esta declaración para usted? "...porque la presentación es atractiva (por ejemplo, el empaque)"

- Extremedamente importante
- Muy importante
- Moderadamente importante
- Ligeramente importante
- No es nada importante

Pensando en las razones por las que usted consume paella.
¿Cuál es la importancia de esta declaración para usted? "...para poder pasar tiempo con otras personas y socializar"

- Extremedamente importante
- Muy importante
- Moderadamente importante
- Ligeramente importante
- No es nada importante

Pensando en las razones por las que usted consume paella.

¿Cuál es la importancia de esta declaración para usted? "...porque es orgánico"

- Extremedamente importante
- Muy importante
- Moderadamente importante
- Ligeramente importante
- No es nada importante

Pensando en las razones por las que usted consume paella.

¿Cuál es la importancia de esta declaración para usted? "...porque crecí con ello"

- Extremedamente importante
- Muy importante
- Moderadamente importante
- Ligeramente importante
- No es nada importante

Pensando en las razones por las que usted consume paella.

¿Cuál es la importancia de esta declaración para usted? "...porque lo disfruto"

- Extremedamente importante
- Muy importante
- Moderadamente importante
- Ligeramente importante
- No es nada importante

Pensando en las razones por las que usted consume paella.

¿Cuál es la importancia de esta declaración para usted? "...porque tengo hambre"

- Extremedamente importante
- Muy importante
- Moderadamente importante

- Ligeramente importante
- No es nada importante

Pensando en las razones por las que usted consume paella.

¿Cuál es la importancia de esta declaración para usted? "...porque usualmente lo como"

- Extremedamente importante
- Muy importante
- Moderadamente importante
- Ligeramente importante
- No es nada importante

Pensando en las razones por las que usted consume paella.

¿Cuál es la importancia de esta declaración para usted? "...porque me apetece"

- Extremedamente importante
- Muy importante
- Moderadamente importante
- Ligeramente importante
- No es nada importante

Pensando en las razones por las que usted consume paella.

¿Cuál es la importancia de esta declaración para usted? "...porque es fácil de preparar"

- Extremedamente importante
- Muy importante
- Moderadamente importante
- Ligeramente importante
- No es nada importante

Pensando en las razones por las que usted consume paella.

¿Cuál es la importancia de esta declaración para usted? "...porque me siento triste"

- Extremedamente importante
- Muy importante
- Moderadamente importante
- Ligeramente importante
- No es nada importante

Pensando en las razones por las que usted consume paella.

¿Cuál es la importancia de esta declaración para usted? "...porque es bajo en grasa"

- Extremedamente importante
- Muy importante
- Moderadamente importante
- Ligeramente importante
- No es nada importante

Pensando en las razones por las que usted consume paella.

¿Cuál es la importancia de esta declaración para usted? "...porque lo he visto en anuncios o en la televisión"

- Extremedamente importante
- Muy importante
- Moderadamente importante
- Ligeramente importante
- No es nada importante

Pensando en las razones por las que usted consume paella.

¿Cuál es la importancia de esta declaración para usted? "...porque hace más agradables las reuniones sociales"

- Extremedamente importante
- Muy importante
- Moderadamente importante
- Ligeramente importante
- No es nada importante

Pensando en las razones por las que usted consume paella.

¿Cuál es la importancia de esta declaración para usted? "...porque se supone que debo comerlo"

- Extremedamente importante
- Muy importante
- Moderadamente importante
- Ligeramente importante
- No es nada importante

Pensando en las razones por las que usted consume paella.

¿Cuál es la importancia de esta declaración para usted? "...porque le gusta a otros"

- Extremedamente importante
- Muy importante
- Moderadamente importante
- Ligeramente importante
- No es nada importante

Pensando en las razones por las que usted consume paella.

¿Cuál es la importancia de esta declaración para usted? "...porque me siento solo"

- Extremedamente importante
- Muy importante
- Moderadamente importante
- Ligeramente importante
- No es nada importante

Pensando en las razones por las que usted consume paella.

¿Cuál es la importancia de esta declaración para usted? "...porque vigilo mi peso"

- Extremedamente importante
- Muy importante

- Moderadamente importante
- Ligeramente importante
- No es nada importante

Pensando en las razones por las que usted consume paella.
¿Cuál es la importancia de esta declaración para usted? "...porque no contiene sustancias peligrosas (por ejemplo, pesticidas, contaminantes, o antibióticos"

- Extremedamente importante
- Muy importante
- Moderadamente importante
- Ligeramente importante
- No es nada importante

Pensando en las razones por las que usted consume paella.
¿Cuál es la importancia de esta declaración para usted? "...porque es lo más conveniente"

- Extremedamente importante
- Muy importante
- Moderadamente importante
- Ligeramente importante
- No es nada importante

Pensando en las razones por las que usted consume paella.
¿Cuál es la importancia de esta declaración para usted? "...porque necesito energía"

- Extremedamente importante
- Muy importante
- Moderadamente importante
- Ligeramente importante
- No es nada importante

Pensando en las razones por las que usted consume paella.

¿Cuál es la importancia de esta declaración para usted? "...porque estoy familiarizado con ello"

- Extremedamente importante
- Muy importante
- Moderadamente importante
- Ligeramente importante
- No es nada importante

Pensando en las razones por las que usted consume paella.

¿Cuál es la importancia de esta declaración para usted? "..porque me gusta"

- Extremedamente importante
- Muy importante
- Moderadamente importante
- Ligeramente importante
- No es nada importante

Pensando en las razones por las que usted consume paella.

¿Cuál es la importancia de esta declaración para usted? "..porque me llena de un modo agradable"

- Extremedamente importante
- Muy importante
- Moderadamente importante
- Ligeramente importante
- No es nada importante

Pensando en las razones por las que usted consume paella.

¿Cuál es la importancia de esta declaración para usted? "..para recompensarme"

- Extremedamente importante
- Muy importante
- Moderadamente importante
- Ligeramente importante

- No es nada importante

Pensando en las razones por las que usted consume paella.

¿Cuál es la importancia de esta declaración para usted? "...es parte de mis tradiciones (por ejemplo, tradiciones familiares, ocasiones especiales)"

- Extremedamente importante
- Muy importante
- Moderadamente importante
- Ligeramente importante
- No es nada importante

Pensando en las razones por las que usted consume paella.

¿Cuál es la importancia de esta declaración para usted? "...porque se consume en ciertas ocasiones"

- Extremedamente importante
- Muy importante
- Moderadamente importante
- Ligeramente importante
- No es nada importante

Pensando en las razones por las que usted consume paella.

¿Cuál es la importancia de esta declaración para usted? "...para mantener una dieta equilibrada"

- Extremedamente importante
- Muy importante
- Moderadamente importante
- Ligeramente importante
- No es nada importante

Pensando en las razones por las que usted consume paella.

¿Cuál es la importancia de esta declaración para usted? "...porque es social"

- Extremedamente importante
- Muy importante
- Moderadamente importante
- Ligeramente importante
- No es nada importante

Pensando en las razones por las que usted consume paella.
¿Cuál es la importancia de esta declaración para usted? "...porque es barato"

- Extremedamente importante
- Muy importante
- Moderadamente importante
- Ligeramente importante
- No es nada importante

Pensando en las razones por las que usted consume paella.
¿Cuál es la importancia de esta declaración para usted? "...porque me apetece de modo espontáneo (por ejemplo, colores llamativos a la vista)"

- Extremedamente importante
- Muy importante
- Moderadamente importante
- Ligeramente importante
- No es nada importante

Pensando en las razones por las que usted consume paella.
¿Cuál es la importancia de esta declaración para usted? "...para evitar decepcionar a alguien que está tratando de hacerme feliz"

- Extremedamente importante
- Muy importante
- Moderadamente importante
- Ligeramente importante

- No es nada importante

Pensando en las razones por las que usted consume paella.

¿Cuál es la importancia de esta declaración para usted? "...porque está de moda"

- Extremedamente importante
- Muy importante
- Moderadamente importante
- Ligeramente importante
- No es nada importante

Pensando en las razones por las que usted consume paella.

¿Cuál es la importancia de esta declaración para usted? "...porque está en rebaja"

- Extremedamente importante
- Muy importante
- Moderadamente importante
- Ligeramente importante
- No es nada importante

Pensando en las razones por las que usted consume paella.

¿Cuál es la importancia de esta declaración para usted? "...porque me mantiene en forma (por ejemplo, energético, motivado)"

- Extremedamente importante
- Muy importante
- Moderadamente importante
- Ligeramente importante
- No es nada importante

Pensando en las razones por las que usted consume paella.

¿Cuál es la importancia de esta declaración para usted? "...porque esa era la única opción"

- Extremedamente importante
- Muy importante
- Moderadamente importante
- Ligeramente importante
- No es nada importante

Pensando en las razones por las que usted consume paella.

¿Cuál es la importancia de esta declaración para usted? "…porque quiero comerlo todos los días"

- Extremedamente importante
- Muy importante
- Moderadamente importante
- Ligeramente importante
- No es nada importante

Pensando en las razones por las que usted no consume paella.

¿Cuál es la importancia de esta declaración para usted? "…No lo considero como un aperitivo/snack "

- Extremedamente importante
- Muy importante
- Moderadamente importante
- Ligeramente importante
- No es nada importante

Pensando en las razones por las que usted no consume paella.

¿Cuál es la importancia de esta declaración para usted? "…No me gusta"

- Extremedamente importante
- Muy importante
- Moderadamente importante
- Ligeramente importante
- No es nada importante

Pensando en las razones por las que usted no consume paella.

¿Cuál es la importancia de esta declaración para usted? "...No lo consumo usualmente"

- Extremedamente importante
- Muy importante
- Moderadamente importante
- Ligeramente importante
- No es nada importante

Pensando en las razones por las que usted no consume paella.

¿Cuál es la importancia de esta declaración para usted? "...El tamaño de la porción no era adecuado"

- Extremedamente importante
- Muy importante
- Moderadamente importante
- Ligeramente importante
- No es nada importante

Pensando en las razones por las que usted no consume paella.

¿Cuál es la importancia de esta declaración para usted? "... No lo considero un alimento que llene lo suficiente"

- Extremedamente importante
- Muy importante
- Moderadamente importante
- Ligeramente importante
- No es nada importante

Pensando en las razones por las que usted no consume paella.

¿Cuál es la importancia de esta declaración para usted? "... No es saludable"

- Extremedamente importante
- Muy importante

- Moderadamente importante
- Ligeramente importante
- No es nada importante

Pensando en las razones por las que usted no consume paella.

¿Cuál es la importancia de esta declaración para usted? "**...** No es práctico de preparar"

- Extremedamente importante
- Muy importante
- Moderadamente importante
- Ligeramente importante
- No es nada importante

Pensando en las razones por las que usted no consume paella.

¿Cuál es la importancia de esta declaración para usted? "**...** No quise consentirme"

- Extremedamente importante
- Muy importante
- Moderadamente importante
- Ligeramente importante
- No es nada importante

Pensando en las razones por las que usted no consume paella.

¿Cuál es la importancia de esta declaración para usted? "**...** No era apropiado para la ocasión"

- Extremedamente importante
- Muy importante
- Moderadamente importante
- Ligeramente importante
- No es nada importante

Pensando en las razones por las que usted no consume paella.

¿Cuál es la importancia de esta declaración para usted? "... No es un tipo de comida que consumo en presencia de otras personas"

- Extremedamente importante
- Muy importante
- Moderadamente importante
- Ligeramente importante
- No es nada importante

Pensando en las razones por las que usted no consume paella.

¿Cuál es la importancia de esta declaración para usted? "...El precio era muy elevado"

- Extremedamente importante
- Muy importante
- Moderadamente importante
- Ligeramente importante
- No es nada importante

Pensando en las razones por las que usted no consume paella.

¿Cuál es la importancia de esta declaración para usted? "...No me gustó la apariencia"

- Extremedamente importante
- Muy importante
- Moderadamente importante
- Ligeramente importante
- No es nada importante

Pensando en las razones por las que usted no consume paella.

¿Cuál es la importancia de esta declaración para usted? "...Es muy alto en calorías"

- Extremedamente importante
- Muy importante
- Moderadamente importante

- Ligeramente importante
- No es nada importante

Pensando en las razones por las que usted no consume paella.

¿Cuál es la importancia de esta declaración para usted? "...Se supone que no debo consumirlo"

- Extremedamente importante
- Muy importante
- Moderadamente importante
- Ligeramente importante
- No es nada importante

Pensando en las razones por las que usted no consume paella.

¿Cuál es la importancia de esta declaración para usted? "...Mi generación no consume este tipo de alimento"

- Extremedamente importante
- Muy importante
- Moderadamente importante
- Ligeramente importante
- No es nada importante

Pensando en las razones por las que usted no consume paella.

¿Cuál es la importancia de esta declaración para usted? "...No era algo que se me apetecía en ese momento"

- Extremedamente importante
- Muy importante
- Moderadamente importante
- Ligeramente importante
- No es nada importante

Pensando en las razones por las que usted no consume paella.

¿Cuál es la importancia de esta declaración para usted? "…Lo consumí recientemente y no quiero consumir el mismo alimento muy seguido"

- Extremedamente importante
- Muy importante
- Moderadamente importante
- Ligeramente importante
- No es nada importante

Pensando en las razones por las que usted no consume paella.

¿Cuál es la importancia de esta declaración para usted? "…Nunca lo escogería. Me gusta consumir el mismo alimento todos los días"

- Extremedamente importante
- Muy importante
- Moderadamente importante
- Ligeramente importante
- No es nada importante

Pensando en las razones por las que usted no consume paella.

¿Cuál es la importancia de esta declaración para usted? "…Me hace sentir triste, solitario, o frustrado"

- Extremedamente importante
- Muy importante
- Moderadamente importante
- Ligeramente importante
- No es nada importante

Pensando en las razones por las que usted no consume paella.

¿Cuál es la importancia de esta declaración para usted? "…No es orgánico"

- Extremedamente importante
- Muy importante

- Moderadamente importante
- Ligeramente importante
- No es nada importante

Reasons for Protein foods Choices(Full RATA)

¿Come usted jamón serrano?

- Sí
- No

Pensando en las razones por las que usted consume jamón serrano.
¿Cuál es la importancia de esta declaración para usted? "...porque estoy acostumbrado a comerlo "

- Extremedamente importante
- Muy importante
- Moderadamente importante
- Ligeramente importante
- No es nada importante

Pensando en las razones por las que usted consume jamón serrano.
¿Cuál es la importancia de esta declaración para usted? "...porque es saludable"

- Extremedamente importante
- Muy importante
- Moderadamente importante
- Ligeramente importante
- No es nada importante

Pensando en las razones por las que usted consume jamón serrano.
¿Cuál es la importancia de esta declaración para usted? "...porque es rápido de preparar"

- Extremedamente importante

- Muy importante
- Moderadamente importante
- Ligeramente importante
- No es nada importante

Pensando en las razones por las que usted consume jamón serrano.

¿Cuál es la importancia de esta declaración para usted? "...porque sabe bien"

- Extremedamente importante
- Muy importante
- Moderadamente importante
- Ligeramente importante
- No es nada importante

Pensando en las razones por las que usted consume jamón serrano.

¿Cuál es la importancia de esta declaración para usted? "...para complacerme"

- Extremedamente importante
- Muy importante
- Moderadamente importante
- Ligeramente importante
- No es nada importante

Pensando en las razones por las que usted consume jamón serrano.

¿Cuál es la importancia de esta declaración para usted? "...porque es natural (por ejemplo, no modificado genéticamente)"

- Extremedamente importante
- Muy importante
- Moderadamente importante
- Ligeramente importante
- No es nada importante

Pensando en las razones por las que usted consume jamón serrano.

¿Cuál es la importancia de esta declaración para usted? "...porque no quiero gastar más dinero"

- Extremedamente importante
- Muy importante
- Moderadamente importante
- Ligeramente importante
- No es nada importante

Pensando en las razones por las que usted consume jamón serrano.

¿Cuál es la importancia de esta declaración para usted? "...porque es bajo en calorías"

- Extremedamente importante
- Muy importante
- Moderadamente importante
- Ligeramente importante
- No es nada importante

Pensando en las razones por las que usted consume jamón serrano.

¿Cuál es la importancia de esta declaración para usted? "...porque me siento frustrado"

- Extremedamente importante
- Muy importante
- Moderadamente importante
- Ligeramente importante
- No es nada importante

Pensando en las razones por las que usted consume jamón serrano.

¿Cuál es la importancia de esta declaración para usted? "...porque me hace quedar bien delante de los demás"

- Extremedamente importante
- Muy importante
- Moderadamente importante
- Ligeramente importante
- No es nada importante

Pensando en las razones por las que usted consume jamón serrano.
¿Cuál es la importancia de esta declaración para usted? "...porque sería descortés no comerlo"

- Extremedamente importante
- Muy importante
- Moderadamente importante
- Ligeramente importante
- No es nada importante

Pensando en las razones por las que usted consume jamón serrano.
¿Cuál es la importancia de esta declaración para usted? "...porque la presentación es atractiva (por ejemplo, el empaque)"

- Extremedamente importante
- Muy importante
- Moderadamente importante
- Ligeramente importante
- No es nada importante

Pensando en las razones por las que usted consume jamón serrano.

¿Cuál es la importancia de esta declaración para usted? "...para poder pasar tiempo con otras personas y socializar"

- Extremedamente importante
- Muy importante
- Moderadamente importante
- Ligeramente importante
- No es nada importante

Pensando en las razones por las que usted consume jamón serrano.
¿Cuál es la importancia de esta declaración para usted? "...porque es orgánico"

- Extremedamente importante
- Muy importante
- Moderadamente importante
- Ligeramente importante
- No es nada importante

Pensando en las razones por las que usted consume jamón serrano.
¿Cuál es la importancia de esta declaración para usted? "...porque crecí con ello"

- Extremedamente importante
- Muy importante
- Moderadamente importante
- Ligeramente importante
- No es nada importante

Pensando en las razones por las que usted consume jamón serrano.
¿Cuál es la importancia de esta declaración para usted? "...porque lo disfruto"

- Extremedamente importante
- Muy importante

- Moderadamente importante
- Ligeramente importante
- No es nada importante

Pensando en las razones por las que usted consume jamón serrano.

¿Cuál es la importancia de esta declaración para usted? "...porque tengo hambre"

- Extremedamente importante
- Muy importante
- Moderadamente importante
- Ligeramente importante
- No es nada importante

Pensando en las razones por las que usted consume jamón serrano.

¿Cuál es la importancia de esta declaración para usted? "...porque usualmente lo como"

- Extremedamente importante
- Muy importante
- Moderadamente importante
- Ligeramente importante
- No es nada importante

Pensando en las razones por las que usted consume jamón serrano.

¿Cuál es la importancia de esta declaración para usted? "...porque me apetece"

- Extremedamente importante
- Muy importante
- Moderadamente importante
- Ligeramente importante
- No es nada importante

Pensando en las razones por las que usted consume jamón serrano.

¿Cuál es la importancia de esta declaración para usted? "...porque es fácil de preparar"

- Extremedamente importante
- Muy importante
- Moderadamente importante
- Ligeramente importante
- No es nada importante

Pensando en las razones por las que usted consume jamón serrano.

¿Cuál es la importancia de esta declaración para usted? "...porque me siento triste"

- Extremedamente importante
- Muy importante
- Moderadamente importante
- Ligeramente importante
- No es nada importante

Pensando en las razones por las que usted consume jamón serrano.

¿Cuál es la importancia de esta declaración para usted? "...porque es bajo en grasa"

- Extremedamente importante
- Muy importante
- Moderadamente importante
- Ligeramente importante
- No es nada importante

Pensando en las razones por las que usted consume jamón serrano.

¿Cuál es la importancia de esta declaración para usted? "...porque lo he visto en anuncios o en la televisión"

- Extremedamente importante
- Muy importante
- Moderadamente importante
- Ligeramente importante

- No es nada importante

Pensando en las razones por las que usted consume jamón serrano.
¿Cuál es la importancia de esta declaración para usted? "...porque hace más agradables las reuniones sociales"

- Extremedamente importante
- Muy importante
- Moderadamente importante
- Ligeramente importante
- No es nada importante

Pensando en las razones por las que usted consume jamón serrano.
¿Cuál es la importancia de esta declaración para usted? "...porque se supone que debo comerlo"

- Extremedamente importante
- Muy importante
- Moderadamente importante
- Ligeramente importante
- No es nada importante

Pensando en las razones por las que usted consume jamón serrano.
¿Cuál es la importancia de esta declaración para usted? "...porque le gusta a otros"

- Extremedamente importante
- Muy importante
- Moderadamente importante
- Ligeramente importante
- No es nada importante

Pensando en las razones por las que usted consume jamón serrano.
¿Cuál es la importancia de esta declaración para usted? "...porque me siento solo"

- Extremedamente importante
- Muy importante
- Moderadamente importante
- Ligeramente importante
- No es nada importante

Pensando en las razones por las que usted consume jamón serrano.
¿Cuál es la importancia de esta declaración para usted? "...porque vigilo mi peso"

- Extremedamente importante
- Muy importante
- Moderadamente importante
- Ligeramente importante
- No es nada importante

Pensando en las razones por las que usted consume jamón serrano.
¿Cuál es la importancia de esta declaración para usted? "...porque no contiene sustancias peligrosas (por ejemplo, pesticidas, contaminantes, o antibióticos"

- Extremedamente importante
- Muy importante
- Moderadamente importante
- Ligeramente importante
- No es nada importante

Pensando en las razones por las que usted consume jamón serrano.
¿Cuál es la importancia de esta declaración para usted? "...porque es lo más conveniente"

- Extremedamente importante
- Muy importante
- Moderadamente importante
- Ligeramente importante
- No es nada importante

Pensando en las razones por las que usted consume jamón serrano.
¿Cuál es la importancia de esta declaración para usted? "...porque necesito energía"

- Extremedamente importante
- Muy importante
- Moderadamente importante
- Ligeramente importante
- No es nada importante

Pensando en las razones por las que usted consume jamón serrano.
¿Cuál es la importancia de esta declaración para usted? "...porque estoy familiarizado con ello"

- Extremedamente importante
- Muy importante
- Moderadamente importante
- Ligeramente importante
- No es nada importante

Pensando en las razones por las que usted consume jamón serrano.
¿Cuál es la importancia de esta declaración para usted? "..porque me gusta"

- Extremedamente importante
- Muy importante
- Moderadamente importante
- Ligeramente importante
- No es nada importante

Pensando en las razones por las que usted consume jamón serrano.
¿Cuál es la importancia de esta declaración para usted? "..porque me llena de un modo agradable"

- Extremedamente importante
- Muy importante

- Moderadamente importante
- Ligeramente importante
- No es nada importante

Pensando en las razones por las que usted consume jamón serrano.

¿Cuál es la importancia de esta declaración para usted? "..para recompensarme"

- Extremedamente importante
- Muy importante
- Moderadamente importante
- Ligeramente importante
- No es nada importante

Pensando en las razones por las que usted consume jamón serrano.

¿Cuál es la importancia de esta declaración para usted? "...es parte de mis tradiciones (por ejemplo, tradiciones familiares, ocasiones especiales)"

- Extremedamente importante
- Muy importante
- Moderadamente importante
- Ligeramente importante
- No es nada importante

Pensando en las razones por las que usted consume jamón serrano.

¿Cuál es la importancia de esta declaración para usted? "...porque se consume en ciertas ocasiones"

- Extremedamente importante
- Muy importante
- Moderadamente importante
- Ligeramente importante
- No es nada importante

Pensando en las razones por las que usted consume jamón serrano.
¿Cuál es la importancia de esta declaración para usted? "...para mantener una dieta equilibrada"

- Extremedamente importante
- Muy importante
- Moderadamente importante
- Ligeramente importante
- No es nada importante

Pensando en las razones por las que usted consume jamón serrano.
¿Cuál es la importancia de esta declaración para usted? "...porque es social"

- Extremedamente importante
- Muy importante
- Moderadamente importante
- Ligeramente importante
- No es nada importante

Pensando en las razones por las que usted consume jamón serrano.
¿Cuál es la importancia de esta declaración para usted? "...porque es barato"

- Extremedamente importante
- Muy importante
- Moderadamente importante
- Ligeramente importante
- No es nada importante

Pensando en las razones por las que usted consume jamón serrano.
¿Cuál es la importancia de esta declaración para usted? "...porque me apetece de modo espontáneo (por ejemplo, colores llamativos a la vista)"

- Extremedamente importante
- Muy importante

- Moderadamente importante
- Ligeramente importante
- No es nada importante

Pensando en las razones por las que usted consume jamón serrano.

¿Cuál es la importancia de esta declaración para usted? "...para evitar decepcionar a alguien que está tratando de hacerme feliz"

- Extremedamente importante
- Muy importante
- Moderadamente importante
- Ligeramente importante
- No es nada importante

Pensando en las razones por las que usted consume jamón serrano.

¿Cuál es la importancia de esta declaración para usted? "...porque está de moda"

- Extremedamente importante
- Muy importante
- Moderadamente importante
- Ligeramente importante
- No es nada importante

Pensando en las razones por las que usted consume jamón serrano.

¿Cuál es la importancia de esta declaración para usted? "...porque está en rebaja"

- Extremedamente importante
- Muy importante
- Moderadamente importante
- Ligeramente importante
- No es nada importante

Pensando en las razones por las que usted consume jamón serrano.
¿Cuál es la importancia de esta declaración para usted? "...porque me mantiene en forma (por ejemplo, energético, motivado)"

- Extremedamente importante
- Muy importante
- Moderadamente importante
- Ligeramente importante
- No es nada importante

Pensando en las razones por las que usted consume jamón serrano.
¿Cuál es la importancia de esta declaración para usted? "...porque esa era la única opción"

- Extremedamente importante
- Muy importante
- Moderadamente importante
- Ligeramente importante
- No es nada importante

Pensando en las razones por las que usted consume jamón serrano.
¿Cuál es la importancia de esta declaración para usted? "...porque quiero comerlo todos los días"

- Extremedamente importante
- Muy importante
- Moderadamente importante
- Ligeramente importante
- No es nada importante

Pensando en las razones por las que usted no consume jamón serrano.
¿Cuál es la importancia de esta declaración para usted? "...No lo considero como un aperitivo/snack "

- Extremedamente importante
- Muy importante

- Moderadamente importante
- Ligeramente importante
- No es nada importante

Pensando en las razones por las que usted no consume jamón serrano.

¿Cuál es la importancia de esta declaración para usted? "...No me gusta"

- Extremedamente importante
- Muy importante
- Moderadamente importante
- Ligeramente importante
- No es nada importante

Pensando en las razones por las que usted no consume jamón serrano.

¿Cuál es la importancia de esta declaración para usted? "...No lo consumo usualmente"

- Extremedamente importante
- Muy importante
- Moderadamente importante
- Ligeramente importante
- No es nada importante

Pensando en las razones por las que usted no consume jamón serrano.

¿Cuál es la importancia de esta declaración para usted? "...El tamaño de la porción no era adecuado"

- Extremedamente importante
- Muy importante
- Moderadamente importante
- Ligeramente importante
- No es nada importante

Pensando en las razones por las que usted no consume jamón serrano.

¿Cuál es la importancia de esta declaración para usted? "... No lo considero un alimento que llene lo suficiente"

- Extremedamente importante
- Muy importante
- Moderadamente importante
- Ligeramente importante
- No es nada importante

Pensando en las razones por las que usted no consume jamón serrano.
¿Cuál es la importancia de esta declaración para usted? "... No es saludable"

- Extremedamente importante
- Muy importante
- Moderadamente importante
- Ligeramente importante
- No es nada importante

Pensando en las razones por las que usted no consume jamón serrano.
¿Cuál es la importancia de esta declaración para usted? "... No es práctico de preparar"

- Extremedamente importante
- Muy importante
- Moderadamente importante
- Ligeramente importante
- No es nada importante

Pensando en las razones por las que usted no consume jamón serrano.
¿Cuál es la importancia de esta declaración para usted? "... No quise consentirme"

- Extremedamente importante
- Muy importante
- Moderadamente importante

- Ligeramente importante
- No es nada importante

Pensando en las razones por las que usted no consume jamón serrano.

¿Cuál es la importancia de esta declaración para usted? "... No era apropiado para la ocasión"

- Extremedamente importante
- Muy importante
- Moderadamente importante
- Ligeramente importante
- No es nada importante

Pensando en las razones por las que usted no consume jamón serrano.

¿Cuál es la importancia de esta declaración para usted? "... No es un tipo de comida que consumo en presencia de otras personas"

- Extremedamente importante
- Muy importante
- Moderadamente importante
- Ligeramente importante
- No es nada importante

Pensando en las razones por las que usted no consume jamón serrano.

¿Cuál es la importancia de esta declaración para usted? "...El precio era muy elevado"

- Extremedamente importante
- Muy importante
- Moderadamente importante
- Ligeramente importante
- No es nada importante

Pensando en las razones por las que usted no consume jamón serrano.

¿Cuál es la importancia de esta declaración para usted? "...No me gustó la apariencia"

- Extremedamente importante
- Muy importante
- Moderadamente importante
- Ligeramente importante
- No es nada importante

Pensando en las razones por las que usted no consume jamón serrano.

¿Cuál es la importancia de esta declaración para usted? "...Es muy alto en calorías"

- Extremedamente importante
- Muy importante
- Moderadamente importante
- Ligeramente importante
- No es nada importante

Pensando en las razones por las que usted no consume jamón serrano.

¿Cuál es la importancia de esta declaración para usted? "...Se supone que no debo consumirlo"

- Extremedamente importante
- Muy importante
- Moderadamente importante
- Ligeramente importante
- No es nada importante

Pensando en las razones por las que usted no consume jamón serrano.

¿Cuál es la importancia de esta declaración para usted? "...Mi generación no consume este tipo de alimento"

- Extremedamente importante
- Muy importante
- Moderadamente importante
- Ligeramente importante

- No es nada importante

Pensando en las razones por las que usted no consume jamón serrano.

¿Cuál es la importancia de esta declaración para usted? "...No era algo que se me apetecía en ese momento"

- Extremedamente importante
- Muy importante
- Moderadamente importante
- Ligeramente importante
- No es nada importante

Pensando en las razones por las que usted no consume jamón serrano.

¿Cuál es la importancia de esta declaración para usted? "...Lo consumí recientemente y no quiero consumir el mismo alimento muy seguido"

- Extremedamente importante
- Muy importante
- Moderadamente importante
- Ligeramente importante
- No es nada importante

Pensando en las razones por las que usted no consume jamón serrano.

¿Cuál es la importancia de esta declaración para usted? "...Nunca lo escogería. Me gusta consumir el mismo alimento todos los días"

- Extremedamente importante
- Muy importante
- Moderadamente importante
- Ligeramente importante
- No es nada importante

Pensando en las razones por las que usted no consume jamón serrano.

¿Cuál es la importancia de esta declaración para usted? "...Me hace sentir triste, solitario, o frustrado"

- Extremedamente importante
- Muy importante
- Moderadamente importante
- Ligeramente importante
- No es nada importante

Pensando en las razones por las que usted no consume jamón serrano.

¿Cuál es la importancia de esta declaración para usted? "...No es orgánico"

- Extremedamente importante
- Muy importante
- Moderadamente importante
- Ligeramente importante
- No es nada importante

Reasons for Dairy Food Choices(Full RATA)

¿Come usted leche?

- Sí
- No

Pensando en las razones por las que usted consume leche.

¿Cuál es la importancia de esta declaración para usted? "...porque estoy acostumbrado a comerlo "

- Extremedamente importante
- Muy importante
- Moderadamente importante
- Ligeramente importante
- No es nada importante

Pensando en las razones por las que usted consume leche.

¿Cuál es la importancia de esta declaración para usted? "...porque es saludable"

- Extremedamente importante
- Muy importante
- Moderadamente importante
- Ligeramente importante
- No es nada importante

Pensando en las razones por las que usted consume leche.

¿Cuál es la importancia de esta declaración para usted? "...porque es rápido de preparar"

- Extremedamente importante
- Muy importante
- Moderadamente importante
- Ligeramente importante
- No es nada importante

Pensando en las razones por las que usted consume leche.

¿Cuál es la importancia de esta declaración para usted? "...porque sabe bien"

- Extremedamente importante
- Muy importante
- Moderadamente importante
- Ligeramente importante
- No es nada importante

Pensando en las razones por las que usted consume leche.

¿Cuál es la importancia de esta declaración para usted? "...para complacerme"

- Extremedamente importante
- Muy importante
- Moderadamente importante

- Ligeramente importante
- No es nada importante

Pensando en las razones por las que usted consume leche.

¿Cuál es la importancia de esta declaración para usted? "...porque es natural (por ejemplo, no modificado genéticamente)"

- Extremedamente importante
- Muy importante
- Moderadamente importante
- Ligeramente importante
- No es nada importante

Pensando en las razones por las que usted consume leche.

¿Cuál es la importancia de esta declaración para usted? "...porque no quiero gastar más dinero"

- Extremedamente importante
- Muy importante
- Moderadamente importante
- Ligeramente importante
- No es nada importante

Pensando en las razones por las que usted consume leche.

¿Cuál es la importancia de esta declaración para usted? "...porque es bajo en calorías"

- Extremedamente importante
- Muy importante
- Moderadamente importante
- Ligeramente importante
- No es nada importante

Pensando en las razones por las que usted consume leche.

¿Cuál es la importancia de esta declaración para usted? "...porque me siento frustrado"

- Extremedamente importante
- Muy importante
- Moderadamente importante
- Ligeramente importante
- No es nada importante

Pensando en las razones por las que usted consume leche.

¿Cuál es la importancia de esta declaración para usted? "...porque me hace quedar bien delante de los demás"

- Extremedamente importante
- Muy importante
- Moderadamente importante
- Ligeramente importante
- No es nada importante

Pensando en las razones por las que usted consume leche.

¿Cuál es la importancia de esta declaración para usted? "...porque sería descortés no comerlo"

- Extremedamente importante
- Muy importante
- Moderadamente importante
- Ligeramente importante
- No es nada importante

Pensando en las razones por las que usted consume leche.

¿Cuál es la importancia de esta declaración para usted? "...porque la presentación es atractiva (por ejemplo, el empaque)"

- Extremedamente importante
- Muy importante
- Moderadamente importante
- Ligeramente importante
- No es nada importante

Pensando en las razones por las que usted consume leche.
¿Cuál es la importancia de esta declaración para usted? "...para poder pasar tiempo con otras personas y socializar"

- Extremedamente importante
- Muy importante
- Moderadamente importante
- Ligeramente importante
- No es nada importante

Pensando en las razones por las que usted consume leche.
¿Cuál es la importancia de esta declaración para usted? "...porque es orgánico"

- Extremedamente importante
- Muy importante
- Moderadamente importante
- Ligeramente importante
- No es nada importante

Pensando en las razones por las que usted consume leche.
¿Cuál es la importancia de esta declaración para usted? "...porque crecí con ello"

- Extremedamente importante
- Muy importante
- Moderadamente importante
- Ligeramente importante
- No es nada importante

Pensando en las razones por las que usted consume leche.
¿Cuál es la importancia de esta declaración para usted? "...porque lo disfruto"

- Extremedamente importante
- Muy importante
- Moderadamente importante
- Ligeramente importante
- No es nada importante

Pensando en las razones por las que usted consume leche.
¿Cuál es la importancia de esta declaración para usted? "...porque tengo hambre"

- Extremedamente importante
- Muy importante
- Moderadamente importante
- Ligeramente importante
- No es nada importante

Pensando en las razones por las que usted consume leche.
¿Cuál es la importancia de esta declaración para usted? "...porque usualmente lo como"

- Extremedamente importante
- Muy importante
- Moderadamente importante
- Ligeramente importante
- No es nada importante

Pensando en las razones por las que usted consume leche.

¿Cuál es la importancia de esta declaración para usted? "...porque me apetece"

- Extremedamente importante
- Muy importante
- Moderadamente importante
- Ligeramente importante
- No es nada importante

Pensando en las razones por las que usted consume leche.

¿Cuál es la importancia de esta declaración para usted? "...porque es fácil de preparar"

- Extremedamente importante
- Muy importante
- Moderadamente importante
- Ligeramente importante
- No es nada importante

Pensando en las razones por las que usted consume leche.

¿Cuál es la importancia de esta declaración para usted? "...porque me siento triste"

- Extremedamente importante
- Muy importante
- Moderadamente importante
- Ligeramente importante
- No es nada importante

Pensando en las razones por las que usted consume leche.

¿Cuál es la importancia de esta declaración para usted? "...porque es bajo en grasa"

- Extremedamente importante
- Muy importante
- Moderadamente importante

- Ligeramente importante
- No es nada importante

Pensando en las razones por las que usted consume leche.

¿Cuál es la importancia de esta declaración para usted? "...porque lo he visto en anuncios o en la televisión"

- Extremedamente importante
- Muy importante
- Moderadamente importante
- Ligeramente importante
- No es nada importante

Pensando en las razones por las que usted consume leche.

¿Cuál es la importancia de esta declaración para usted? "...porque hace más agradables las reuniones sociales"

- Extremedamente importante
- Muy importante
- Moderadamente importante
- Ligeramente importante
- No es nada importante

Pensando en las razones por las que usted consume leche.

¿Cuál es la importancia de esta declaración para usted? "...porque se supone que debo comerlo"

- Extremedamente importante
- Muy importante
- Moderadamente importante
- Ligeramente importante
- No es nada importante

Pensando en las razones por las que usted consume leche.

¿Cuál es la importancia de esta declaración para usted? "...porque le gusta a otros"

- Extremedamente importante
- Muy importante
- Moderadamente importante
- Ligeramente importante
- No es nada importante

Pensando en las razones por las que usted consume leche.

¿Cuál es la importancia de esta declaración para usted? "...porque me siento solo"

- Extremedamente importante
- Muy importante
- Moderadamente importante
- Ligeramente importante
- No es nada importante

Pensando en las razones por las que usted consume leche.

¿Cuál es la importancia de esta declaración para usted? "...porque vigilo mi peso"

- Extremedamente importante
- Muy importante
- Moderadamente importante
- Ligeramente importante
- No es nada importante

Pensando en las razones por las que usted consume leche.

¿Cuál es la importancia de esta declaración para usted? "...porque no contiene sustancias peligrosas (por ejemplo, pesticidas, contaminantes, o antibióticos"

- Extremedamente importante
- Muy importante
- Moderadamente importante

- Ligeramente importante
- No es nada importante

Pensando en las razones por las que usted consume leche.

¿Cuál es la importancia de esta declaración para usted? "...porque es lo más conveniente"

- Extremedamente importante
- Muy importante
- Moderadamente importante
- Ligeramente importante
- No es nada importante

Pensando en las razones por las que usted consume leche.

¿Cuál es la importancia de esta declaración para usted? "...porque necesito energía"

- Extremedamente importante
- Muy importante
- Moderadamente importante
- Ligeramente importante
- No es nada importante

Pensando en las razones por las que usted consume leche.

¿Cuál es la importancia de esta declaración para usted? "...porque estoy familiarizado con ello"

- Extremedamente importante
- Muy importante
- Moderadamente importante
- Ligeramente importante
- No es nada importante

Pensando en las razones por las que usted consume leche.

¿Cuál es la importancia de esta declaración para usted? "..porque me gusta"

- Extremedamente importante
- Muy importante
- Moderadamente importante
- Ligeramente importante
- No es nada importante

Pensando en las razones por las que usted consume leche.
¿Cuál es la importancia de esta declaración para usted? "..porque me llena de un modo agradable"

- Extremedamente importante
- Muy importante
- Moderadamente importante
- Ligeramente importante
- No es nada importante

Pensando en las razones por las que usted consume leche.
¿Cuál es la importancia de esta declaración para usted? "..para recompensarme"

- Extremedamente importante
- Muy importante
- Moderadamente importante
- Ligeramente importante
- No es nada importante

Pensando en las razones por las que usted consume leche.
¿Cuál es la importancia de esta declaración para usted? "...es parte de mis tradiciones (por ejemplo, tradiciones familiares, ocasiones especiales)"

- Extremedamente importante
- Muy importante
- Moderadamente importante
- Ligeramente importante
- No es nada importante

Pensando en las razones por las que usted consume leche.

¿Cuál es la importancia de esta declaración para usted? "...porque se consume en ciertas ocasiones"

- Extremedamente importante
- Muy importante
- Moderadamente importante
- Ligeramente importante
- No es nada importante

Pensando en las razones por las que usted consume leche.

¿Cuál es la importancia de esta declaración para usted? "...para mantener una dieta equilibrada"

- Extremedamente importante
- Muy importante
- Moderadamente importante
- Ligeramente importante
- No es nada importante

Pensando en las razones por las que usted consume leche.

¿Cuál es la importancia de esta declaración para usted? "...porque es social"

- Extremedamente importante
- Muy importante
- Moderadamente importante
- Ligeramente importante
- No es nada importante

Pensando en las razones por las que usted consume leche.

¿Cuál es la importancia de esta declaración para usted? "...porque es barato"

- Extremedamente importante
- Muy importante

- Moderadamente importante
- Ligeramente importante
- No es nada importante

Pensando en las razones por las que usted consume leche.
¿Cuál es la importancia de esta declaración para usted? "**...**porque me apetece de modo espontáneo (por ejemplo, colores llamativos a la vista)"

- Extremedamente importante
- Muy importante
- Moderadamente importante
- Ligeramente importante
- No es nada importante

Pensando en las razones por las que usted consume leche.
¿Cuál es la importancia de esta declaración para usted? "**...**para evitar decepcionar a alguien que está tratando de hacerme feliz"

- Extremedamente importante
- Muy importante
- Moderadamente importante
- Ligeramente importante
- No es nada importante

Pensando en las razones por las que usted consume leche.
¿Cuál es la importancia de esta declaración para usted? "**...**porque está de moda"

- Extremedamente importante
- Muy importante
- Moderadamente importante
- Ligeramente importante
- No es nada importante

Pensando en las razones por las que usted consume leche.
¿Cuál es la importancia de esta declaración para usted? "...porque está en rebaja"

- Extremedamente importante
- Muy importante
- Moderadamente importante
- Ligeramente importante
- No es nada importante

Pensando en las razones por las que usted consume leche.
¿Cuál es la importancia de esta declaración para usted? "...porque me mantiene en forma (por ejemplo, energético, motivado)"

- Extremedamente importante
- Muy importante
- Moderadamente importante
- Ligeramente importante
- No es nada importante

Pensando en las razones por las que usted consume leche.
¿Cuál es la importancia de esta declaración para usted? "...porque esa era la única opción"

- Extremedamente importante
- Muy importante
- Moderadamente importante
- Ligeramente importante
- No es nada importante

Pensando en las razones por las que usted consume leche.
¿Cuál es la importancia de esta declaración para usted? "...porque quiero comerlo todos los días"

- Extremedamente importante
- Muy importante

- Moderadamente importante
- Ligeramente importante
- No es nada importante

Pensando en las razones por las que usted no consume leche.

¿Cuál es la importancia de esta declaración para usted? "...No lo considero como un aperitivo/snack "

- Extremedamente importante
- Muy importante
- Moderadamente importante
- Ligeramente importante
- No es nada importante

Pensando en las razones por las que usted no consume leche.

¿Cuál es la importancia de esta declaración para usted? "...No me gusta"

- Extremedamente importante
- Muy importante
- Moderadamente importante
- Ligeramente importante
- No es nada importante

Pensando en las razones por las que usted no consume leche.

¿Cuál es la importancia de esta declaración para usted? "...No lo consumo usualmente"

- Extremedamente importante
- Muy importante
- Moderadamente importante
- Ligeramente importante
- No es nada importante

Pensando en las razones por las que usted no consume leche.

¿Cuál es la importancia de esta declaración para usted? "...El tamaño de la porción no era adecuado"

- Extremedamente importante
- Muy importante
- Moderadamente importante
- Ligeramente importante
- No es nada importante

Pensando en las razones por las que usted no consume leche.

¿Cuál es la importancia de esta declaración para usted? "... No lo considero un alimento que llene lo suficiente"

- Extremedamente importante
- Muy importante
- Moderadamente importante
- Ligeramente importante
- No es nada importante

Pensando en las razones por las que usted no consume leche.

¿Cuál es la importancia de esta declaración para usted? "... No es saludable"

- Extremedamente importante
- Muy importante
- Moderadamente importante
- Ligeramente importante
- No es nada importante

Pensando en las razones por las que usted no consume leche.

¿Cuál es la importancia de esta declaración para usted? "... No es práctico de preparar"

- Extremedamente importante
- Muy importante
- Moderadamente importante

- Ligeramente importante
- No es nada importante

Pensando en las razones por las que usted no consume leche.

¿Cuál es la importancia de esta declaración para usted? "... No quise consentirme"

- Extremedamente importante
- Muy importante
- Moderadamente importante
- Ligeramente importante
- No es nada importante

Pensando en las razones por las que usted no consume leche.

¿Cuál es la importancia de esta declaración para usted? "... No era apropiado para la ocasión"

- Extremedamente importante
- Muy importante
- Moderadamente importante
- Ligeramente importante
- No es nada importante

Pensando en las razones por las que usted no consume leche.

¿Cuál es la importancia de esta declaración para usted? "... No es un tipo de comida que consumo en presencia de otras personas"

- Extremedamente importante
- Muy importante
- Moderadamente importante
- Ligeramente importante
- No es nada importante

Pensando en las razones por las que usted no consume leche.

¿Cuál es la importancia de esta declaración para usted? "...El precio era muy elevado"

- Extremedamente importante
- Muy importante
- Moderadamente importante
- Ligeramente importante
- No es nada importante

Pensando en las razones por las que usted no consume leche.

¿Cuál es la importancia de esta declaración para usted? "...No me gustó la apariencia"

- Extremedamente importante
- Muy importante
- Moderadamente importante
- Ligeramente importante
- No es nada importante

Pensando en las razones por las que usted no consume leche.

¿Cuál es la importancia de esta declaración para usted? "...Es muy alto en calorías"

- Extremedamente importante
- Muy importante
- Moderadamente importante
- Ligeramente importante
- No es nada importante

Pensando en las razones por las que usted no consume leche.

¿Cuál es la importancia de esta declaración para usted? "...Se supone que no debo consumirlo"

- Extremedamente importante
- Muy importante
- Moderadamente importante
- Ligeramente importante

- No es nada importante

Pensando en las razones por las que usted no consume leche.

¿Cuál es la importancia de esta declaración para usted? "...Mi generación no consume este tipo de alimento"

- Extremedamente importante
- Muy importante
- Moderadamente importante
- Ligeramente importante
- No es nada importante

Pensando en las razones por las que usted no consume leche.

¿Cuál es la importancia de esta declaración para usted? "...No era algo que se me apetecía en ese momento"

- Extremedamente importante
- Muy importante
- Moderadamente importante
- Ligeramente importante
- No es nada importante

Pensando en las razones por las que usted no consume leche.

¿Cuál es la importancia de esta declaración para usted? "...Lo consumí recientemente y no quiero consumir el mismo alimento muy seguido"

- Extremedamente importante
- Muy importante
- Moderadamente importante
- Ligeramente importante
- No es nada importante

Pensando en las razones por las que usted no consume leche.

¿Cuál es la importancia de esta declaración para usted? "...Nunca lo escogería. Me gusta consumir el mismo alimento todos los días"

- Extremedamente importante
- Muy importante
- Moderadamente importante
- Ligeramente importante
- No es nada importante

Pensando en las razones por las que usted no consume leche.
¿Cuál es la importancia de esta declaración para usted? "...Me hace sentir triste, solitario, o frustrado"

- Extremedamente importante
- Muy importante
- Moderadamente importante
- Ligeramente importante
- No es nada importante

Pensando en las razones por las que usted no consume leche.
¿Cuál es la importancia de esta declaración para usted? "...No es orgánico"

- Extremedamente importante
- Muy importante
- Moderadamente importante
- Ligeramente importante
- No es nada importante

Reasons for Fruit Choices(Full RATA)

¿Come usted plátano ?

- Sí
- No

Pensando en las razones por las que usted consume plátano .

¿Cuál es la importancia de esta declaración para usted? "...porque estoy acostumbrado a comerlo "

- Extremedamente importante
- Muy importante
- Moderadamente importante
- Ligeramente importante
- No es nada importante

Pensando en las razones por las que usted consume plátano .

¿Cuál es la importancia de esta declaración para usted? "...porque es saludable"

- Extremedamente importante
- Muy importante
- Moderadamente importante
- Ligeramente importante
- No es nada importante

Pensando en las razones por las que usted consume plátano .

¿Cuál es la importancia de esta declaración para usted? "...porque es rápido de preparar"

- Extremedamente importante
- Muy importante
- Moderadamente importante
- Ligeramente importante
- No es nada importante

Pensando en las razones por las que usted consume plátano .

¿Cuál es la importancia de esta declaración para usted? "...porque sabe bien"

- Extremedamente importante
- Muy importante

- Moderadamente importante
- Ligeramente importante
- No es nada importante

Pensando en las razones por las que usted consume plátano .

¿Cuál es la importancia de esta declaración para usted? "...para complacerme"

- Extremedamente importante
- Muy importante
- Moderadamente importante
- Ligeramente importante
- No es nada importante

Pensando en las razones por las que usted consume plátano .

¿Cuál es la importancia de esta declaración para usted? "...porque es natural (por ejemplo, no modificado genéticamente)"

- Extremedamente importante
- Muy importante
- Moderadamente importante
- Ligeramente importante
- No es nada importante

Pensando en las razones por las que usted consume plátano .

¿Cuál es la importancia de esta declaración para usted? "...porque no quiero gastar más dinero"

- Extremedamente importante
- Muy importante
- Moderadamente importante
- Ligeramente importante
- No es nada importante

Pensando en las razones por las que usted consume plátano .

¿Cuál es la importancia de esta declaración para usted? "...porque es bajo en calorías"

- Extremedamente importante
- Muy importante
- Moderadamente importante
- Ligeramente importante
- No es nada importante

Pensando en las razones por las que usted consume plátano .

¿Cuál es la importancia de esta declaración para usted? "...porque me siento frustrado"

- Extremedamente importante
- Muy importante
- Moderadamente importante
- Ligeramente importante
- No es nada importante

Pensando en las razones por las que usted consume plátano .

¿Cuál es la importancia de esta declaración para usted? "...porque me hace quedar bien delante de los demás"

- Extremedamente importante
- Muy importante
- Moderadamente importante
- Ligeramente importante
- No es nada importante

Pensando en las razones por las que usted consume plátano .

¿Cuál es la importancia de esta declaración para usted? "...porque sería descortés no comerlo"

- Extremedamente importante
- Muy importante
- Moderadamente importante
- Ligeramente importante
- No es nada importante

Pensando en las razones por las que usted consume plátano .
¿Cuál es la importancia de esta declaración para usted? "...porque la presentación es atractiva (por ejemplo, el empaque)"

- Extremedamente importante
- Muy importante
- Moderadamente importante
- Ligeramente importante
- No es nada importante

Pensando en las razones por las que usted consume plátano .
¿Cuál es la importancia de esta declaración para usted? "...para poder pasar tiempo con otras personas y socializar"

- Extremedamente importante
- Muy importante
- Moderadamente importante
- Ligeramente importante
- No es nada importante

Pensando en las razones por las que usted consume plátano .

¿Cuál es la importancia de esta declaración para usted? "...porque es orgánico"

- Extremedamente importante
- Muy importante
- Moderadamente importante
- Ligeramente importante
- No es nada importante

Pensando en las razones por las que usted consume plátano .

¿Cuál es la importancia de esta declaración para usted? "...porque crecí con ello"

- Extremedamente importante
- Muy importante
- Moderadamente importante
- Ligeramente importante
- No es nada importante

Pensando en las razones por las que usted consume plátano .

¿Cuál es la importancia de esta declaración para usted? "...porque lo disfruto"

- Extremedamente importante
- Muy importante
- Moderadamente importante
- Ligeramente importante
- No es nada importante

Pensando en las razones por las que usted consume plátano .

¿Cuál es la importancia de esta declaración para usted? "...porque tengo hambre"

- Extremedamente importante
- Muy importante
- Moderadamente importante
- Ligeramente importante

- No es nada importante

Pensando en las razones por las que usted consume plátano .
¿Cuál es la importancia de esta declaración para usted? "...porque usualmente lo como"

- Extremedamente importante
- Muy importante
- Moderadamente importante
- Ligeramente importante
- No es nada importante

Pensando en las razones por las que usted consume plátano .
¿Cuál es la importancia de esta declaración para usted? "...porque me apetece"

- Extremedamente importante
- Muy importante
- Moderadamente importante
- Ligeramente importante
- No es nada importante

Pensando en las razones por las que usted consume plátano .
¿Cuál es la importancia de esta declaración para usted? "...porque es fácil de preparar"

- Extremedamente importante
- Muy importante
- Moderadamente importante
- Ligeramente importante
- No es nada importante

Pensando en las razones por las que usted consume plátano .
¿Cuál es la importancia de esta declaración para usted? "...porque me siento triste"

- Extremedamente importante

- Muy importante
- Moderadamente importante
- Ligeramente importante
- No es nada importante

Pensando en las razones por las que usted consume plátano .

¿Cuál es la importancia de esta declaración para usted? "...porque es bajo en grasa"

- Extremedamente importante
- Muy importante
- Moderadamente importante
- Ligeramente importante
- No es nada importante

Pensando en las razones por las que usted consume plátano .

¿Cuál es la importancia de esta declaración para usted? "...porque lo he visto en anuncios o en la televisión"

- Extremedamente importante
- Muy importante
- Moderadamente importante
- Ligeramente importante
- No es nada importante

Pensando en las razones por las que usted consume plátano .

¿Cuál es la importancia de esta declaración para usted? "...porque hace más agradables las reuniones sociales"

- Extremedamente importante
- Muy importante
- Moderadamente importante
- Ligeramente importante
- No es nada importante

Pensando en las razones por las que usted consume plátano .

¿Cuál es la importancia de esta declaración para usted? "...porque se supone que debo comerlo"

- Extremedamente importante
- Muy importante
- Moderadamente importante
- Ligeramente importante
- No es nada importante

Pensando en las razones por las que usted consume plátano .

¿Cuál es la importancia de esta declaración para usted? "...porque le gusta a otros"

- Extremedamente importante
- Muy importante
- Moderadamente importante
- Ligeramente importante
- No es nada importante

Pensando en las razones por las que usted consume plátano .

¿Cuál es la importancia de esta declaración para usted? "...porque me siento solo"

- Extremedamente importante
- Muy importante
- Moderadamente importante
- Ligeramente importante
- No es nada importante

Pensando en las razones por las que usted consume plátano .

¿Cuál es la importancia de esta declaración para usted? "...porque vigilo mi peso"

- Extremedamente importante
- Muy importante
- Moderadamente importante

- Ligeramente importante
- No es nada importante

Pensando en las razones por las que usted consume plátano .
¿Cuál es la importancia de esta declaración para usted? "...porque no contiene sustancias peligrosas (por ejemplo, pesticidas, contaminantes, o antibióticos"

- Extremedamente importante
- Muy importante
- Moderadamente importante
- Ligeramente importante
- No es nada importante

Pensando en las razones por las que usted consume plátano .
¿Cuál es la importancia de esta declaración para usted? "...porque es lo más conveniente"

- Extremedamente importante
- Muy importante
- Moderadamente importante
- Ligeramente importante
- No es nada importante

Pensando en las razones por las que usted consume plátano .
¿Cuál es la importancia de esta declaración para usted? "...porque necesito energía"

- Extremedamente importante
- Muy importante
- Moderadamente importante
- Ligeramente importante
- No es nada importante

Pensando en las razones por las que usted consume plátano .

¿Cuál es la importancia de esta declaración para usted? "...porque estoy familiarizado con ello"

- Extremedamente importante
- Muy importante
- Moderadamente importante
- Ligeramente importante
- No es nada importante

Pensando en las razones por las que usted consume plátano .

¿Cuál es la importancia de esta declaración para usted? "..porque me gusta"

- Extremedamente importante
- Muy importante
- Moderadamente importante
- Ligeramente importante
- No es nada importante

Pensando en las razones por las que usted consume plátano .

¿Cuál es la importancia de esta declaración para usted? "..porque me llena de un modo agradable"

- Extremedamente importante
- Muy importante
- Moderadamente importante
- Ligeramente importante
- No es nada importante

Pensando en las razones por las que usted consume plátano .

¿Cuál es la importancia de esta declaración para usted? "..para recompensarme"

- Extremedamente importante
- Muy importante
- Moderadamente importante
- Ligeramente importante

○ No es nada importante

Pensando en las razones por las que usted consume plátano .

¿Cuál es la importancia de esta declaración para usted? "…es parte de mis tradiciones (por ejemplo, tradiciones familiares, ocasiones especiales)"

○ Extremedamente importante
○ Muy importante
○ Moderadamente importante
○ Ligeramente importante
○ No es nada importante

Pensando en las razones por las que usted consume plátano .

¿Cuál es la importancia de esta declaración para usted? "…porque se consume en ciertas ocasiones"

○ Extremedamente importante
○ Muy importante
○ Moderadamente importante
○ Ligeramente importante
○ No es nada importante

Pensando en las razones por las que usted consume plátano .

¿Cuál es la importancia de esta declaración para usted? "…para mantener una dieta equilibrada"

○ Extremedamente importante
○ Muy importante
○ Moderadamente importante
○ Ligeramente importante
○ No es nada importante

Pensando en las razones por las que usted consume plátano .

¿Cuál es la importancia de esta declaración para usted? "…porque es social"

- Extremedamente importante
- Muy importante
- Moderadamente importante
- Ligeramente importante
- No es nada importante

Pensando en las razones por las que usted consume plátano .
¿Cuál es la importancia de esta declaración para usted? "...porque es barato"

- Extremedamente importante
- Muy importante
- Moderadamente importante
- Ligeramente importante
- No es nada importante

Pensando en las razones por las que usted consume plátano .
¿Cuál es la importancia de esta declaración para usted? "...porque me apetece de modo espontáneo (por ejemplo, colores llamativos a la vista)"

- Extremedamente importante
- Muy importante
- Moderadamente importante
- Ligeramente importante
- No es nada importante

Pensando en las razones por las que usted consume plátano .
¿Cuál es la importancia de esta declaración para usted? "...para evitar decepcionar a alguien que está tratando de hacerme feliz"

- Extremedamente importante
- Muy importante
- Moderadamente importante
- Ligeramente importante

- No es nada importante

Pensando en las razones por las que usted consume plátano .

¿Cuál es la importancia de esta declaración para usted? "...porque está de moda"

- Extremedamente importante
- Muy importante
- Moderadamente importante
- Ligeramente importante
- No es nada importante

Pensando en las razones por las que usted consume plátano .

¿Cuál es la importancia de esta declaración para usted? "...porque está en rebaja"

- Extremedamente importante
- Muy importante
- Moderadamente importante
- Ligeramente importante
- No es nada importante

Pensando en las razones por las que usted consume plátano .

¿Cuál es la importancia de esta declaración para usted? "...porque me mantiene en forma (por ejemplo, energético, motivado)"

- Extremedamente importante
- Muy importante
- Moderadamente importante
- Ligeramente importante
- No es nada importante

Pensando en las razones por las que usted consume plátano .

¿Cuál es la importancia de esta declaración para usted? "...porque esa era la única opción"

- Extremedamente importante
- Muy importante
- Moderadamente importante
- Ligeramente importante
- No es nada importante

Pensando en las razones por las que usted consume plátano .

¿Cuál es la importancia de esta declaración para usted? "…porque quiero comerlo todos los días"

- Extremedamente importante
- Muy importante
- Moderadamente importante
- Ligeramente importante
- No es nada importante

Pensando en las razones por las que usted no consume plátano .

¿Cuál es la importancia de esta declaración para usted? "…No lo considero como un aperitivo/snack "

- Extremedamente importante
- Muy importante
- Moderadamente importante
- Ligeramente importante
- No es nada importante

Pensando en las razones por las que usted no consume plátano .

¿Cuál es la importancia de esta declaración para usted? "…No me gusta"

- Extremedamente importante
- Muy importante
- Moderadamente importante
- Ligeramente importante
- No es nada importante

Pensando en las razones por las que usted no consume plátano .

¿Cuál es la importancia de esta declaración para usted? "...No lo consumo usualmente"

- Extremedamente importante
- Muy importante
- Moderadamente importante
- Ligeramente importante
- No es nada importante

Pensando en las razones por las que usted no consume plátano .

¿Cuál es la importancia de esta declaración para usted? "...El tamaño de la porción no era adecuado"

- Extremedamente importante
- Muy importante
- Moderadamente importante
- Ligeramente importante
- No es nada importante

Pensando en las razones por las que usted no consume plátano .

¿Cuál es la importancia de esta declaración para usted? "... No lo considero un alimento que llene lo suficiente"

- Extremedamente importante
- Muy importante
- Moderadamente importante
- Ligeramente importante
- No es nada importante

Pensando en las razones por las que usted no consume plátano .

¿Cuál es la importancia de esta declaración para usted? "... No es saludable"

- Extremedamente importante
- Muy importante

- Moderadamente importante
- Ligeramente importante
- No es nada importante

Pensando en las razones por las que usted no consume plátano .

¿Cuál es la importancia de esta declaración para usted? "... No es práctico de preparar"

- Extremedamente importante
- Muy importante
- Moderadamente importante
- Ligeramente importante
- No es nada importante

Pensando en las razones por las que usted no consume plátano .

¿Cuál es la importancia de esta declaración para usted? "... No quise consentirme"

- Extremedamente importante
- Muy importante
- Moderadamente importante
- Ligeramente importante
- No es nada importante

Pensando en las razones por las que usted no consume plátano .

¿Cuál es la importancia de esta declaración para usted? "... No era apropiado para la ocasión"

- Extremedamente importante
- Muy importante
- Moderadamente importante
- Ligeramente importante
- No es nada importante

Pensando en las razones por las que usted no consume plátano .

¿Cuál es la importancia de esta declaración para usted? "... No es un tipo de comida que consumo en presencia de otras personas"

- Extremedamente importante
- Muy importante
- Moderadamente importante
- Ligeramente importante
- No es nada importante

Pensando en las razones por las que usted no consume plátano .
¿Cuál es la importancia de esta declaración para usted? "...El precio era muy elevado"

- Extremedamente importante
- Muy importante
- Moderadamente importante
- Ligeramente importante
- No es nada importante

Pensando en las razones por las que usted no consume plátano .
¿Cuál es la importancia de esta declaración para usted? "...No me gustó la apariencia"

- Extremedamente importante
- Muy importante
- Moderadamente importante
- Ligeramente importante
- No es nada importante

Pensando en las razones por las que usted no consume plátano .
¿Cuál es la importancia de esta declaración para usted? "...Es muy alto en calorías"

- Extremedamente importante
- Muy importante
- Moderadamente importante

- Ligeramente importante
- No es nada importante

Pensando en las razones por las que usted no consume plátano .

¿Cuál es la importancia de esta declaración para usted? "...Se supone que no debo consumirlo"

- Extremedamente importante
- Muy importante
- Moderadamente importante
- Ligeramente importante
- No es nada importante

Pensando en las razones por las que usted no consume plátano .

¿Cuál es la importancia de esta declaración para usted? "...Mi generación no consume este tipo de alimento"

- Extremedamente importante
- Muy importante
- Moderadamente importante
- Ligeramente importante
- No es nada importante

Pensando en las razones por las que usted no consume plátano .

¿Cuál es la importancia de esta declaración para usted? "...No era algo que se me apetecía en ese momento"

- Extremedamente importante
- Muy importante
- Moderadamente importante
- Ligeramente importante
- No es nada importante

Pensando en las razones por las que usted no consume plátano .

¿Cuál es la importancia de esta declaración para usted? "...Lo consumí recientemente y no quiero consumir el mismo alimento muy seguido"

- Extremedamente importante
- Muy importante
- Moderadamente importante
- Ligeramente importante
- No es nada importante

Pensando en las razones por las que usted no consume plátano .

¿Cuál es la importancia de esta declaración para usted? "...Nunca lo escogería. Me gusta consumir el mismo alimento todos los días"

- Extremedamente importante
- Muy importante
- Moderadamente importante
- Ligeramente importante
- No es nada importante

Pensando en las razones por las que usted no consume plátano .

¿Cuál es la importancia de esta declaración para usted? "...Me hace sentir triste, solitario, o frustrado"

- Extremedamente importante
- Muy importante
- Moderadamente importante
- Ligeramente importante
- No es nada importante

Pensando en las razones por las que usted no consume plátano.

¿Cuál es la importancia de esta declaración para usted? "...No es orgánico"

- Extremedamente importante
- Muy importante

- Moderadamente importante
- Ligeramente importante
- No es nada importante

Reasons for Sweet food Choices(Full RATA)

¿Come usted turrón ?

- Sí
- No

Pensando en las razones por las que usted consume turrón . ¿Cuál es la importancia de esta declaración para usted? "...porque estoy acostumbrado a comerlo "

- Extremedamente importante
- Muy importante
- Moderadamente importante
- Ligeramente importante
- No es nada importante

Pensando en las razones por las que usted consume turrón . ¿Cuál es la importancia de esta declaración para usted? "...porque es saludable"

- Extremedamente importante
- Muy importante
- Moderadamente importante
- Ligeramente importante
- No es nada importante

Pensando en las razones por las que usted consume turrón . ¿Cuál es la importancia de esta declaración para usted? "...porque es rápido de preparar"

- Extremedamente importante
- Muy importante
- Moderadamente importante
- Ligeramente importante
- No es nada importante

Pensando en las razones por las que usted consume turrón . ¿Cuál es la importancia de esta declaración para usted? "...porque sabe bien"

- Extremedamente importante
- Muy importante
- Moderadamente importante
- Ligeramente importante
- No es nada importante

Pensando en las razones por las que usted consume turrón . ¿Cuál es la importancia de esta declaración para usted? "...para complacerme"

- Extremedamente importante
- Muy importante
- Moderadamente importante
- Ligeramente importante
- No es nada importante

Pensando en las razones por las que usted consume turrón . ¿Cuál es la importancia de esta declaración para usted? "...porque es natural (por ejemplo, no modificado genéticamente)"

- Extremedamente importante

- Muy importante
- Moderadamente importante
- Ligeramente importante
- No es nada importante

Pensando en las razones por las que usted consume turrón . ¿Cuál es la importancia de esta declaración para usted? "...porque no quiero gastar más dinero"

- Extremedamente importante
- Muy importante
- Moderadamente importante
- Ligeramente importante
- No es nada importante

Pensando en las razones por las que usted consume turrón . ¿Cuál es la importancia de esta declaración para usted? "...porque es bajo en calorías"

- Extremedamente importante
- Muy importante
- Moderadamente importante
- Ligeramente importante
- No es nada importante

Pensando en las razones por las que usted consume turrón . ¿Cuál es la importancia de esta declaración para usted? "...porque me siento frustrado"

- Extremedamente importante
- Muy importante
- Moderadamente importante
- Ligeramente importante

- No es nada importante

Pensando en las razones por las que usted consume turrón . ¿Cuál es la importancia de esta declaración para usted? "...porque me hace quedar bien delante de los demás"

- Extremedamente importante
- Muy importante
- Moderadamente importante
- Ligeramente importante
- No es nada importante

Pensando en las razones por las que usted consume turrón . ¿Cuál es la importancia de esta declaración para usted? "...porque sería descortés no comerlo"

- Extremedamente importante
- Muy importante
- Moderadamente importante
- Ligeramente importante
- No es nada importante

Pensando en las razones por las que usted consume turrón . ¿Cuál es la importancia de esta declaración para usted? "...porque la presentación es atractiva (por ejemplo, el empaque)"

- Extremedamente importante
- Muy importante
- Moderadamente importante
- Ligeramente importante
- No es nada importante

Pensando en las razones por las que usted consume turrón . ¿Cuál es la importancia de esta declaración para usted? "...para poder pasar tiempo con otras personas y socializar"

- Extremedamente importante
- Muy importante
- Moderadamente importante
- Ligeramente importante
- No es nada importante

Pensando en las razones por las que usted consume turrón . ¿Cuál es la importancia de esta declaración para usted? "...porque es orgánico"

- Extremedamente importante
- Muy importante
- Moderadamente importante
- Ligeramente importante
- No es nada importante

Pensando en las razones por las que usted consume turrón . ¿Cuál es la importancia de esta declaración para usted? "...porque crecí con ello"

- Extremedamente importante
- Muy importante
- Moderadamente importante
- Ligeramente importante
- No es nada importante

Pensando en las razones por las que usted consume turrón . ¿Cuál es la importancia de esta declaración para usted? "...porque lo disfruto"

- Extremedamente importante
- Muy importante
- Moderadamente importante
- Ligeramente importante
- No es nada importante

Pensando en las razones por las que usted consume turrón . ¿Cuál es la importancia de esta declaración para usted? "...porque tengo hambre"

- Extremedamente importante
- Muy importante
- Moderadamente importante
- Ligeramente importante
- No es nada importante

Pensando en las razones por las que usted consume turrón . ¿Cuál es la importancia de esta declaración para usted? "...porque usualmente lo como"

- Extremedamente importante
- Muy importante
- Moderadamente importante
- Ligeramente importante
- No es nada importante

Pensando en las razones por las que usted consume turrón . ¿Cuál es la importancia de esta declaración para usted? "...porque me apetece"

- Extremedamente importante
- Muy importante
- Moderadamente importante
- Ligeramente importante
- No es nada importante

Pensando en las razones por las que usted consume turrón . ¿Cuál es la importancia de esta declaración para usted? "...porque es fácil de preparar"

- Extremedamente importante
- Muy importante
- Moderadamente importante
- Ligeramente importante
- No es nada importante

Pensando en las razones por las que usted consume turrón . ¿Cuál es la importancia de esta declaración para usted? "...porque me siento triste"

- Extremedamente importante
- Muy importante
- Moderadamente importante
- Ligeramente importante
- No es nada importante

Pensando en las razones por las que usted consume turrón . ¿Cuál es la importancia de esta declaración para usted? "...porque es bajo en grasa"

- Extremedamente importante
- Muy importante
- Moderadamente importante
- Ligeramente importante
- No es nada importante

Pensando en las razones por las que usted consume turrón . ¿Cuál es la importancia de esta declaración para usted? "...porque lo he visto en anuncios o en la televisión"

- Extremedamente importante
- Muy importante
- Moderadamente importante
- Ligeramente importante
- No es nada importante

Pensando en las razones por las que usted consume turrón . ¿Cuál es la importancia de esta declaración para usted? "...porque hace más agradables las reuniones sociales"

- Extremedamente importante
- Muy importante
- Moderadamente importante
- Ligeramente importante
- No es nada importante

Pensando en las razones por las que usted consume turrón . ¿Cuál es la importancia de esta declaración para usted? "...porque se supone que debo comerlo"

- Extremedamente importante
- Muy importante
- Moderadamente importante
- Ligeramente importante
- No es nada importante

Pensando en las razones por las que usted consume turrón . ¿Cuál es la importancia de esta declaración para usted? "...porque le gusta a otros"

- Extremedamente importante
- Muy importante
- Moderadamente importante

- Ligeramente importante
- No es nada importante

Pensando en las razones por las que usted consume turrón . ¿Cuál es la importancia de esta declaración para usted? "...porque me siento solo"

- Extremedamente importante
- Muy importante
- Moderadamente importante
- Ligeramente importante
- No es nada importante

Pensando en las razones por las que usted consume turrón . ¿Cuál es la importancia de esta declaración para usted? "...porque vigilo mi peso"

- Extremedamente importante
- Muy importante
- Moderadamente importante
- Ligeramente importante
- No es nada importante

Pensando en las razones por las que usted consume turrón . ¿Cuál es la importancia de esta declaración para usted? "...porque no contiene sustancias peligrosas (por ejemplo, pesticidas, contaminantes, o antibióticos"

- Extremedamente importante
- Muy importante
- Moderadamente importante
- Ligeramente importante
- No es nada importante

Pensando en las razones por las que usted consume turrón . ¿Cuál es la importancia de esta declaración para usted? "...porque es lo más conveniente"

- Extremedamente importante
- Muy importante
- Moderadamente importante
- Ligeramente importante
- No es nada importante

Pensando en las razones por las que usted consume turrón . ¿Cuál es la importancia de esta declaración para usted? "...porque necesito energía"

- Extremedamente importante
- Muy importante
- Moderadamente importante
- Ligeramente importante
- No es nada importante

Pensando en las razones por las que usted consume turrón . ¿Cuál es la importancia de esta declaración para usted? "...porque estoy familiarizado con ello"

- Extremedamente importante
- Muy importante
- Moderadamente importante
- Ligeramente importante
- No es nada importante

Pensando en las razones por las que usted consume turrón . ¿Cuál es la importancia de esta declaración para usted? "..porque me gusta"

- Extremedamente importante
- Muy importante

- Moderadamente importante
- Ligeramente importante
- No es nada importante

Pensando en las razones por las que usted consume turrón . ¿Cuál es la importancia de esta declaración para usted? "..porque me llena de un modo agradable"

- Extremedamente importante
- Muy importante
- Moderadamente importante
- Ligeramente importante
- No es nada importante

Pensando en las razones por las que usted consume turrón . ¿Cuál es la importancia de esta declaración para usted? "..para recompensarme"

- Extremedamente importante
- Muy importante
- Moderadamente importante
- Ligeramente importante
- No es nada importante

Pensando en las razones por las que usted consume turrón . ¿Cuál es la importancia de esta declaración para usted? "...es parte de mis tradiciones (por ejemplo, tradiciones familiares, ocasiones especiales)"

- Extremedamente importante
- Muy importante
- Moderadamente importante
- Ligeramente importante
- No es nada importante

Pensando en las razones por las que usted consume turrón . ¿Cuál es la importancia de esta declaración para usted? "...porque se consume en ciertas ocasiones"

- Extremedamente importante
- Muy importante
- Moderadamente importante
- Ligeramente importante
- No es nada importante

Pensando en las razones por las que usted consume turrón . ¿Cuál es la importancia de esta declaración para usted? "...para mantener una dieta equilibrada"

- Extremedamente importante
- Muy importante
- Moderadamente importante
- Ligeramente importante
- No es nada importante

Pensando en las razones por las que usted consume turrón . ¿Cuál es la importancia de esta declaración para usted? "...porque es social"

- Extremedamente importante
- Muy importante
- Moderadamente importante
- Ligeramente importante
- No es nada importante

Pensando en las razones por las que usted consume turrón . ¿Cuál es la importancia de esta declaración para usted? "...porque es barato"

- Extremedamente importante
- Muy importante
- Moderadamente importante
- Ligeramente importante
- No es nada importante

Pensando en las razones por las que usted consume turrón . ¿Cuál es la importancia de esta declaración para usted? "...porque me apetece de modo espontáneo (por ejemplo, colores llamativos a la vista)"

- Extremedamente importante
- Muy importante
- Moderadamente importante
- Ligeramente importante
- No es nada importante

Pensando en las razones por las que usted consume turrón . ¿Cuál es la importancia de esta declaración para usted? "...para evitar decepcionar a alguien que está tratando de hacerme feliz"

- Extremedamente importante
- Muy importante
- Moderadamente importante
- Ligeramente importante
- No es nada importante

Pensando en las razones por las que usted consume turrón . ¿Cuál es la importancia de esta declaración para usted? "...porque está de moda"

- Extremedamente importante
- Muy importante
- Moderadamente importante
- Ligeramente importante
- No es nada importante

Pensando en las razones por las que usted consume turrón . ¿Cuál es la importancia de esta declaración para usted? "...porque está en rebaja"

- Extremedamente importante
- Muy importante
- Moderadamente importante
- Ligeramente importante
- No es nada importante

Pensando en las razones por las que usted consume turrón . ¿Cuál es la importancia de esta declaración para usted? "...porque me mantiene en forma (por ejemplo, energético, motivado)"

- Extremedamente importante
- Muy importante
- Moderadamente importante
- Ligeramente importante
- No es nada importante

Pensando en las razones por las que usted consume turrón . ¿Cuál es la importancia de esta declaración para usted? "...porque esa era la única opción"

- Extremedamente importante
- Muy importante
- Moderadamente importante
- Ligeramente importante

- No es nada importante

Pensando en las razones por las que usted consume turrón . ¿Cuál es la importancia de esta declaración para usted? "...porque quiero comerlo todos los días"

- Extremedamente importante
- Muy importante
- Moderadamente importante
- Ligeramente importante
- No es nada importante

Pensando en las razones por las que usted no consume turrón . ¿Cuál es la importancia de esta declaración para usted? "...No lo considero como un aperitivo/snack "

- Extremedamente importante
- Muy importante
- Moderadamente importante
- Ligeramente importante
- No es nada importante

Pensando en las razones por las que usted no consume turrón . ¿Cuál es la importancia de esta declaración para usted? "...No me gusta"

- Extremedamente importante
- Muy importante
- Moderadamente importante
- Ligeramente importante
- No es nada importante

Pensando en las razones por las que usted no consume turrón . ¿Cuál es la importancia de esta declaración para usted? "...No lo consumo usualmente"

- Extremedamente importante
- Muy importante
- Moderadamente importante
- Ligeramente importante
- No es nada importante

Pensando en las razones por las que usted no consume turrón . ¿Cuál es la importancia de esta declaración para usted? "...El tamaño de la porción no era adecuado"

- Extremedamente importante
- Muy importante
- Moderadamente importante
- Ligeramente importante
- No es nada importante

Pensando en las razones por las que usted no consume turrón . ¿Cuál es la importancia de esta declaración para usted? "... No lo considero un alimento que llene lo suficiente"

- Extremedamente importante
- Muy importante
- Moderadamente importante
- Ligeramente importante
- No es nada importante

Pensando en las razones por las que usted no consume turrón . ¿Cuál es la importancia de esta declaración para usted? "... No es saludable"

- Extremedamente importante
- Muy importante
- Moderadamente importante
- Ligeramente importante
- No es nada importante

Pensando en las razones por las que usted no consume turrón . ¿Cuál es la importancia de esta declaración para usted? "... No es práctico de preparar"

- Extremedamente importante
- Muy importante
- Moderadamente importante
- Ligeramente importante
- No es nada importante

Pensando en las razones por las que usted no consume turrón . ¿Cuál es la importancia de esta declaración para usted? "... No quise consentirme"

- Extremedamente importante
- Muy importante
- Moderadamente importante
- Ligeramente importante
- No es nada importante

Pensando en las razones por las que usted no consume turrón . ¿Cuál es la importancia de esta declaración para usted? "... No era apropiado para la ocasión"

- Extremedamente importante
- Muy importante
- Moderadamente importante
- Ligeramente importante
- No es nada importante

Pensando en las razones por las que usted no consume turrón . ¿Cuál es la importancia de esta declaración para usted? "... No es un tipo de comida que consumo en presencia de otras personas"

- Extremedamente importante
- Muy importante
- Moderadamente importante
- Ligeramente importante
- No es nada importante

Pensando en las razones por las que usted no consume turrón . ¿Cuál es la importancia de esta declaración para usted? "...El precio era muy elevado"

- Extremedamente importante
- Muy importante
- Moderadamente importante
- Ligeramente importante
- No es nada importante

Pensando en las razones por las que usted no consume turrón . ¿Cuál es la importancia de esta declaración para usted? "...No me gustó la apariencia"

- Extremedamente importante
- Muy importante
- Moderadamente importante
- Ligeramente importante
- No es nada importante

Pensando en las razones por las que usted no consume turrón . ¿Cuál es la importancia de esta declaración para usted? "...Es muy alto en calorías"

- Extremedamente importante
- Muy importante
- Moderadamente importante
- Ligeramente importante
- No es nada importante

Pensando en las razones por las que usted no consume turrón . ¿Cuál es la importancia de esta declaración para usted? "...Se supone que no debo consumirlo"

- Extremedamente importante
- Muy importante
- Moderadamente importante
- Ligeramente importante
- No es nada importante

Pensando en las razones por las que usted no consume turrón . ¿Cuál es la importancia de esta declaración para usted? "...Mi generación no consume este tipo de alimento"

- Extremedamente importante
- Muy importante
- Moderadamente importante
- Ligeramente importante
- No es nada importante

Pensando en las razones por las que usted no consume turrón . ¿Cuál es la importancia de esta declaración para usted? "...No era algo que se me apetecía en ese momento"

- Extremedamente importante
- Muy importante

- Moderadamente importante
- Ligeramente importante
- No es nada importante

Pensando en las razones por las que usted no consume turrón . ¿Cuál es la importancia de esta declaración para usted? "...Lo consumí recientemente y no quiero consumir el mismo alimento muy seguido"

- Extremedamente importante
- Muy importante
- Moderadamente importante
- Ligeramente importante
- No es nada importante

Pensando en las razones por las que usted no consume turrón . ¿Cuál es la importancia de esta declaración para usted? "...Nunca lo escogería. Me gusta consumir el mismo alimento todos los días"

- Extremedamente importante
- Muy importante
- Moderadamente importante
- Ligeramente importante
- No es nada importante

Pensando en las razones por las que usted no consume turrón . ¿Cuál es la importancia de esta declaración para usted? "...Me hace sentir triste, solitario, o frustrado"

- Extremedamente importante
- Muy importante
- Moderadamente importante
- Ligeramente importante

- No es nada importante

Pensando en las razones por las que usted no consume turrón. ¿Cuál es la importancia de esta declaración para usted? "...No es orgánico"

- Extremedamente importante
- Muy importante
- Moderadamente importante
- Ligeramente importante
- No es nada importante

Powered by Qualtrics
