# Supplementary material for: Comparing Four Question Formats in Five Languages for On-Line Consumer Surveys
Source: Methods Protoc. 2020 Jul 14;3(3):49. doi: 10.3390/mps3030049 (PMC7563457; doi:10.3390/mps3030049)
Supplement: Supplementary file 1 [file mps-03-00049-s001.zip › supplementary/Figure S5 (India-Hindi).pdf]

NSAS STATE
IVERSITY

English ▾

Demographics

कृपया अपना लिंग चुने।

- पुरुष
- महिला

आप किस वर्ष में पैदा हुए थे।

- 1943 या इससे पहले
- 1944 -1964
- 1965 - 1979
- 1980 -1994
- 1995 - 2001
- 2002 या उसके बाद

आपके द्वारा पूरी की गई उच्चतम डिग्री क्या है।

- प्राथमिक विद्यालय या उससे कम
- उच्च विद्यालय
- कॉलेज या विश्वविद्यालय के स्नातक

आपके परिवार में आपके सहित कितने वयस्क घर में रहते हैं।

- 1
- 2
- 3
- 4
- 5 +

आपके परिवार में 18 वर्ष से कम उम्र के कितने बच्चे हैं।

- 0
- 1
- 2
- 3
- 4 +

हम सर्वेक्षण डेटा की गुणवत्ता के बारे में परवाह करते हैं और आपकी राय के सबसे सटीक उपायों को प्राप्त करने की आशा करते हैं इसलिए हमारे लिए यह महत्वपूर्ण है कि आप सर्वेक्षण में प्रत्येक प्रश्न के बारे में सोच समझकर सबसे अच्छा जवाब प्रदान कीजिए।

क्या आप सर्वेक्षण में सवालों के अपने विचारशील और ईमानदार जवाब प्रदान करने के लिए प्रतिबद्ध हैं।

- मैं सबसे अच्छा जवाब प्रदान करूँगा
- मैं अपने सबसे अच्छे उत्तर प्रदान नहीं करूँगा
- मैं किसी भी तरह से वादा नहीं कर सकता

Food Involvement

खाने के व्यवहार को जानने के लिए सर्वेक्षण

कृपया निम्नलिखित प्रश्नों के लिए अपना उत्तर बताएं।

मैं प्रत्येक दिन भोजन के बारे में ज्यादा नहीं सोचता।

- दृढ़तापूर्वक असहमत
- असहमत
- कुछ हद तक असहमत
- न तो इस बात से सहमत है और न ही असहमत
- कुछ हद तक सहमत
- सहमत
- दृढ़तापूर्वक सहमत

खाना बनाना मेरे लिए बहुत मज़ेदार नहीं है।

- दृढ़तापूर्वक असहमत
- असहमत
- कुछ हद तक असहमत
- न तो इस बात से सहमत है और न ही असहमत
- कुछ हद तक सहमत
- सहमत
- दृढ़तापूर्वक सहमत

मैं जो खाता हूँ या जो खाने जा रहा हूं उसके बारे में बात करना पसंद करता हूँ।

- दृढ़तापूर्वक असहमत
- असहमत
- कुछ हद तक असहमत
- न तो इस बात से सहमत है और न ही असहमत
- कुछ हद तक सहमत
- सहमत
- दृढ़तापूर्वक सहमत

अन्य दैनिक निर्णयों के मुकाबले, मेरे भोजन के विकल्प बहुत महत्वपूर्ण नहीं हैं।

- दृढ़तापूर्वक असहमत
- असहमत
- कुछ हद तक असहमत
- न तो इस बात से सहमत है और न ही असहमत
- कुछ हद तक सहमत
- सहमत
- दृढ़तापूर्वक सहमत

जब मैं यात्रा करता हूँ, जिन चीजों की मैं सबसे ज्यादा आशा करता हूँ उनमें से एक भोजन है।

- दृढ़तापूर्वक असहमत
- असहमत
- कुछ हद तक असहमत
- न तो इस बात से सहमत है और न ही असहमत
- कुछ हद तक सहमत
- सहमत
- दृढ़तापूर्वक सहमत

मैं भोजन के बाद अपने बर्तन खुद साफ़ करता हूँ।

- दृढ़तापूर्वक असहमत
- असहमत
- कुछ हद तक असहमत
- न तो इस बात से सहमत है और न ही असहमत
- कुछ हद तक सहमत
- सहमत
- दृढ़तापूर्वक सहमत

मैं अपने और दूसरों के लिए भोजन बनाने में ख़ुशी महसूस करता हूँ।

- दृढ़तापूर्वक असहमत
- असहमत
- कुछ हद तक असहमत
- न तो इस बात से सहमत है और न ही असहमत
- कुछ हद तक सहमत
- सहमत
- दृढ़तापूर्वक सहमत

जब मैं बाहर भोजन खाता हूँ , तो भोजन के स्वाद के बारे में सोचता या बात करता हूँ।

- दृढ़तापूर्वक असहमत
- असहमत
- कुछ हद तक असहमत
- न तो इस बात से सहमत है और न ही असहमत
- कुछ हद तक सहमत
- सहमत
- दृढ़तापूर्वक सहमत

मैं भोजन मिलाना या काटना पसंद नहीं करता।

- दृढ़तापूर्वक असहमत
- असहमत
- कुछ हद तक असहमत
- न तो इस बात से सहमत है और न ही असहमत
- कुछ हद तक सहमत
- सहमत
- दृढ़तापूर्वक सहमत

मैं अपने लिए भोजन या भोजन सामग्री बाजार से खुद खरीद कर लाता हूँ।

- दृढ़तापूर्वक असहमत
- असहमत
- कुछ हद तक असहमत
- न तो इस बात से सहमत है और न ही असहमत
- कुछ हद तक सहमत
- सहमत
- दृढ़तापूर्वक सहमत

मैं भोजन के बाद बर्तन और भोजन का स्थान या टेबल खुद साफ़ करता हूँ।

- दृढ़तापूर्वक असहमत
- असहमत
- कुछ हद तक असहमत
- न तो इस बात से सहमत है और न ही असहमत
- कुछ हद तक सहमत
- सहमत
- दृढ़तापूर्वक सहमत

मेरे लिए भोजन करने के स्थान की सफाई महत्पूर्ण है।

- दृढ़तापूर्वक असहमत
- असहमत
- कुछ हद तक असहमत
- न तो इस बात से सहमत है और न ही असहमत
- कुछ हद तक सहमत
- सहमत
- दृढ़तापूर्वक सहमत

Health and Taste Attitude

मेरे लिए भोजन की पोस्टिक्ता बहुत महत्पूर्ण है।

- दृढ़तापूर्वक असहमत
- असहमत
- कुछ हद तक असहमत
- न तो इस बात से सहमत है और न ही असहमत
- कुछ हद तक सहमत
- सहमत
- दृढ़तापूर्वक सहमत

हमेशा स्वस्थ और संतुलित आहार का पालन करता हूं

- दृढ़तापूर्वक असहमत
- असहमत
- कुछ हद तक असहमत
- न तो इस बात से सहमत है और न ही असहमत
- कुछ हद तक सहमत
- सहमत
- दृढ़तापूर्वक सहमत

मेरे लिए यह बहुत महत्वपूर्ण है कि मेरा आहार वसा (तेल) में कम हो।

- दृढ़तापूर्वक असहमत
- असहमत
- कुछ हद तक असहमत
- न तो इस बात से सहमत है और न ही असहमत
- कुछ हद तक सहमत
- सहमत
- दृढ़तापूर्वक सहमत

मेरे लिए यह बहुत महत्वपूर्ण है कि मेरे दैनिक आहार में बहुत सारे विटामिन और खनिज हो।

- दृढ़तापूर्वक असहमत
- असहमत
- कुछ हद तक असहमत
- न तो इस बात से सहमत है और न ही असहमत
- कुछ हद तक सहमत
- सहमत
- दृढ़तापूर्वक सहमत

मेरे लिए भोजन की पोस्टिक्ता महत्पूर्ण नहीं है, मैं जो भोजन पसंद करता हूं, वो खाता हूं,

- दृढ़तापूर्वक असहमत
- असहमत
- कुछ हद तक असहमत
- न तो इस बात से सहमत है और न ही असहमत
- कुछ हद तक सहमत
- सहमत
- दृढ़तापूर्वक सहमत

मैं किसी भी खाद्य पदार्थ को खाने से बचता नहीं हूँ, भले ही वो मेरे कोलेस्ट्रॉल को बढ़ा दे.

- दृढ़तापूर्वक असहमत
- असहमत
- कुछ हद तक असहमत
- न तो इस बात से सहमत है और न ही असहमत
- कुछ हद तक सहमत
- सहमत
- दृढ़तापूर्वक सहमत

भोजन की पोस्टिक्ता मेरे लिए बहुत महत्पूर्ण नहीं है। और यह मेरे भोजन विकल्पों पर बहुत कम प्रभाव डालती है।

- दृढ़तापूर्वक असहमत
- असहमत
- कुछ हद तक असहमत
- न तो इस बात से सहमत है और न ही असहमत
- कुछ हद तक सहमत
- सहमत
- दृढ़तापूर्वक सहमत

जलपान/ नाश्ता आदि की पोस्टिक्ता मेरे लिए बहुत महत्पूर्ण नहीं है।

- दृढ़तापूर्वक असहमत
- असहमत
- कुछ हद तक असहमत
- न तो इस बात से सहमत है और न ही असहमत
- कुछ हद तक सहमत
- सहमत
- दृढ़तापूर्वक सहमत

मैं यह नहीं मानता की भोजन हमेशा आनंद का स्रोत होना चाहिए।

- दृढ़तापूर्वक असहमत
- असहमत
- कुछ हद तक असहमत
- न तो इस बात से सहमत है और न ही असहमत
- कुछ हद तक सहमत
- सहमत

- दृढ़तापूर्वक सहमत

भोजन कैसा दीखता है। इस से मुझे कोई फर्क नहीं पड़ता है।

- दृढ़तापूर्वक असहमत
- असहमत
- कुछ हद तक असहमत
- न तो इस बात से सहमत है और न ही असहमत
- कुछ हद तक सहमत
- सहमत
- दृढ़तापूर्वक सहमत

मेरे लिए स्वादिष्ट भोजन सप्ताह के दिन और सप्ताहांत में खाना जरुरी है।

- दृढ़तापूर्वक असहमत
- असहमत
- कुछ हद तक असहमत
- न तो इस बात से सहमत है और न ही असहमत
- कुछ हद तक सहमत
- सहमत
- दृढ़तापूर्वक सहमत

जब मैं भोजन करता हूँ। तब मैं भोजन के स्वाद का आनंद लेने पर ध्यान केंद्रित करता हूँ।

- दृढ़तापूर्वक असहमत
- असहमत
- कुछ हद तक असहमत
- न तो इस बात से सहमत है और न ही असहमत
- कुछ हद तक सहमत

- सहमत
- दृढ़तापूर्वक सहमत

यदि मुझे भोजन का स्वाद पसंद नहीं आता है। तो भी मैं अपना भोजन पूरा समाप्त करता हूँ।

- दृढ़तापूर्वक असहमत
- असहमत
- कुछ हद तक असहमत
- न तो इस बात से सहमत है और न ही असहमत
- कुछ हद तक सहमत
- सहमत
- दृढ़तापूर्वक सहमत

मेरे सप्ताहांत का एक अनिवार्य हिस्सा स्वादिष्ट भोजन खाना है।

- दृढ़तापूर्वक असहमत
- असहमत
- कुछ हद तक असहमत
- न तो इस बात से सहमत है और न ही असहमत
- कुछ हद तक सहमत
- सहमत
- दृढ़तापूर्वक सहमत

Neophobia

मैं लगातार नए और अलग-अलग खाद्य पदार्थों का सेवन करता हूँ।

- दृढ़तापूर्वक असहमत
- असहमत

- कुछ हद तक असहमत
- न तो इस बात से सहमत है और न ही असहमत
- कुछ हद तक सहमत
- सहमत
- दृढ़तापूर्वक सहमत

मैं नए खाद्य पदार्थों पर भरोसा नहीं करता हूँ।

- दृढ़तापूर्वक असहमत
- असहमत
- कुछ हद तक असहमत
- न तो इस बात से सहमत है और न ही असहमत
- कुछ हद तक सहमत
- सहमत
- दृढ़तापूर्वक सहमत

अगर मुझे नहीं पता कि भोजन में क्या है। तो मैं वो भोजन नहीं खाऊंगा।

- दृढ़तापूर्वक असहमत
- असहमत
- कुछ हद तक असहमत
- न तो इस बात से सहमत है और न ही असहमत
- कुछ हद तक सहमत
- सहमत
- दृढ़तापूर्वक सहमत

मुझे विभिन्न देशों के खाद्य पदार्थ पसंद हैं।

- दृढ़तापूर्वक असहमत

- असहमत
- कुछ हद तक असहमत
- न तो इस बात से सहमत है और न ही असहमत
- कुछ हद तक सहमत
- सहमत
- दृढ़तापूर्वक सहमत

परंपरागत भोजन खाने में मैं बहुत असहज महसूस करता हूँ।

- दृढ़तापूर्वक असहमत
- असहमत
- कुछ हद तक असहमत
- न तो इस बात से सहमत है और न ही असहमत
- कुछ हद तक सहमत
- सहमत
- दृढ़तापूर्वक सहमत

मैं पार्टियों में नए भोजन खाने की कोशिश करता हूँ।

- दृढ़तापूर्वक असहमत
- असहमत
- कुछ हद तक असहमत
- न तो इस बात से सहमत है और न ही असहमत
- कुछ हद तक सहमत
- सहमत
- दृढ़तापूर्वक सहमत

मुझे उन चीजों को खाने से डर लग लगता है। जो मैंने पहले कभी नहीं खायें है।

- दृढ़तापूर्वक असहमत
- असहमत
- कुछ हद तक असहमत
- न तो इस बात से सहमत है और न ही असहमत
- कुछ हद तक सहमत
- सहमत
- दृढ़तापूर्वक सहमत

मैं उन खाद्य पदार्थों के बारे में बहुत विशिष्ट हूँ। जो मैं खाता हूँ ।

- दृढ़तापूर्वक असहमत
- असहमत
- कुछ हद तक असहमत
- न तो इस बात से सहमत है और न ही असहमत
- कुछ हद तक सहमत
- सहमत
- दृढ़तापूर्वक सहमत

मैं लगभग कुछ भी खा लेता हूँ ।

- दृढ़तापूर्वक असहमत
- असहमत
- कुछ हद तक असहमत
- न तो इस बात से सहमत है और न ही असहमत
- कुछ हद तक सहमत
- सहमत
- दृढ़तापूर्वक सहमत

मुझे नए परंपरागत रेस्तरां के वयंजन खाना पसंद हैं।

- दृढ़तापूर्वक असहमत
- असहमत
- कुछ हद तक असहमत
- न तो इस बात से सहमत है और न ही असहमत
- कुछ हद तक सहमत
- सहमत
- दृढ़तापूर्वक सहमत

Reasons for Starchy food choices(CATA)

क्या आप चावल खाते हैं।

- हाँ
- नहीं

किन कारणों के लिए आप चावल खातें हैं। (सभी विकल्प चुने)

- क्योंकि चावल सबसे सुविधाजनक है।
- क्योंकि मैं चावल को टीवी या विज्ञापन पर देखता हूँ।
- क्योंकि चावल खाना फैशनेबल है।
- क्योंकि मैं उदास रहता हूँ।
- ताकि मैं अन्य लोगों के साथ समय बिता सकूं।
- क्योंकि चावल या चावल से सम्बंदित भोज कम समय में बन जाता है।
- क्योंकि चावल खाना सामाजिक है।
- क्योंकि मूझे ऊर्जा की जरूरत होती है।
- क्योंकि मैं चावल हर दिन खाना चाहता हूँ।
- क्योंकि चावल खाना मूझे दूसरों के सामने अच्छा दिखाता है।
- क्योंकि मैं चावल से परिचित हूँ।
- क्योंकि चावल प्राकृतिक है।
- क्योंकि मैं अकेला महसूस करता हूँ।

- ☐ क्योंकि चावल ही एकमात्र विकल्प था।
- ☐ क्योंकि मैं अपने वजन पर निगरानी रखता हूँ।
- ☐ क्योंकि चावल खाना मुझे तृप्त करता है।
- ☐ संतुलित आहार बनाए रखने के लिए।
- ☐ क्योंकि चावल में कम कैलोरीज होती है।
- ☐ क्योंकि चावल तैयार करना आसान है।
- ☐ क्योंकि चावल खाना स्वास्थ्य के लिए लाभदायक है।
- ☐ दुसरो को निराश करने से बचने के लिए।
- ☐ क्योंकि चावल सस्ता है।
- ☐ क्योंकि चावलका स्वाद अच्छा है।
- ☐ क्योंकि मैं बचपन से चावल खाता रहा हूँ ।
- ☐ क्योंकि चावल खाना सुखदेय है।
- ☐ क्योंकि चावल कि प्रस्तुति आकर्षक होती है।
- ☐ क्योंकि मुझे चावल पसंद है।
- ☐ क्योंकि चावल कुछ स्थितियों से संबंधित है।
- ☐ मैं अपने आपको पुरस्कृत करने के लिए चावल खाता हूँ।
- ☐ क्योंकि चावल नहीं खाना अशिष्ट होता है।
- ☐ क्योंकि मैं चावल खाने का आदी हूँ।
- ☐ क्योंकि चावल सस्ता है।
- ☐ क्योंकि चावल सामाजिक समारोहों को अधिक आरामदायक बनाता है।
- ☐ चावल खाना मेरी परंपरा का हिस्सा है।
- ☐ क्योंकि मैं निराश रहता हूँ।
- ☐ क्योंकि मैं चावल खाने में आनंद महसूस करता हूँ ।
- ☐ क्योंकि चावल मेरे लिए आकर्षक भोजन है।
- ☐ क्योंकि चावल में कोई हानिकारक (कीटनाशक, प्रदूषक, एंटीबायोटिक) पदार्थ नहीं होता है।
- ☐ क्योंकि मैं भूखा रहता हूँ ।
- ☐ क्योंकि मेरे भीतर चावल खाने कि भूख रहती है।
- ☐ क्योंकि चावल में वसा कि मात्रा कम होती है।
- ☐ क्योंकि चावल मेरे शरीर को आकार में रखता है।

- ☐ क्योंकि मूझे चावल खाना पड़ता है।
- ☐ क्योंकि चावल विक्रय बिक्री पर होता है।
- ☐ क्योंकि चावल जैविक होता है।
- ☐ क्योंकि मैं आमतौर पर चावल खाता हूँ ।
- ☐ क्योंकि चावल दूसरों को पसंद है।

कृपया चावल नहीं खाने के सभी कारण बताएं। (सभी विकल्प चुने)

- ☐ चावल स्वास्थ्यय के लिए लाभदायक नहीं है।
- ☐ मैं चावल खाने से दूसरों के सामने पिछड़ा महसूस करता हूँ।
- ☐ क्योंकि चावल जैविक (आर्गेनिक) नहीं है।
- ☐ चावल कि खुराक का आकार मेरे लिए उपयुक्त नहीं होता है।
- ☐ मुझे नहीं लगता कि चावल एक नाश्ता है।
- ☐ चावल मेरी भूख मिटाने के लिए पर्याप्त नहीं होता है।
- ☐ चावल खाना मूझे दुखी, अकेला और कुंठित महसूस कराता है।
- ☐ मैं चावल नहीं खाना चाहता हूँ।
- ☐ चावल मे कैलोरी की मात्रा बहुत अधिक होती है।
- ☐ मैं आमतौर पर चावल नहीं खाता हूँ।
- ☐ चावल अन्य लोगों के आसपास खाने लायक भोजन नहीं है।
- ☐ मुझे चावल पसंद नहीं है।
- ☐ मेने हाल ही में चावल खाया था और अब मैं बार-बार चावल नहीं खाना चाहता हूँ।
- ☐ चावल बनाना मुश्किल होता है।
- ☐ मेरी चावल खाने कि इच्छा नहीं होती है।
- ☐ चावल कि कीमत बहुत अधिक होती है।
- ☐ चावल मेरी भूख को तृप्त नहीं कर पाता है।
- ☐ मैं कभी चावल नहीं खाऊंगा। क्योंकि मुझे हर दिन एक ही प्रकार का भोजन खाना पसंद है।
- ☐ चावल स्थिति के लिए उपयुक्त नहीं था।
- ☐ चावल मूझे दिखने में अच्छा नहीं लगता है।

Reasons for Protein foods Choices(CATA)

क्या आप दाल खाते हैं।

- ○ हाँ
- ○ नहीं

किन कारणों के लिए आप दाल खातें हैं। (सभी विकल्प चुने)

- ☐ क्योंकि दाल में कम कैलोरीज होती है।
- ☐ क्योंकि दाल नहीं खाना अशिष्ट होता है।
- ☐ क्योंकि दाल कि प्रस्तुति आकर्षक होती है।
- ☐ दुसरो को निराश करने से बचने के लिए।
- ☐ क्योंकि दाल विक्रय बिक्री पर होता है।
- ☐ क्योंकि दाल जैविक होता है।
- ☐ क्योंकि मैं दाल हर दिन खाना चाहता हूँ।
- ☐ क्योंकि दाल मेरे लिए आकर्षक भोजन है।
- ☐ क्योंकि मैं दाल से परिचित हूँ।
- ☐ क्योंकि दाल मेरे शरीर को आकार में रखता है।
- ☐ क्योंकि मैं अपने वजन पर निगरानी रखता हूँ।
- ☐ क्योंकि दाल खाना फैशनेबल है।
- ☐ क्योंकि दाल सामाजिक समारोहों को और अधिक आरामदायक बनाता है।
- ☐ क्योंकि मैं अकेला महसूस करता हूँ।
- ☐ क्योंकि दाल सस्ती है।
- ☐ क्योंकि मूझे दाल खानी पड़ती है।
- ☐ क्योंकि दाल कुछ स्थितियों से संबंधित है।
- ☐ क्योंकि मैं उदास रहता हूँ।
- ☐ क्योंकि मैं दाल को टीवी या विज्ञापन पर देखता हूँ।
- ☐ क्योंकि मेरे भीतर दाल खाने कि भूख रहती है।
- ☐ क्योंकि मैं आमतौर पर दाल खाता हूँ।

- ☐ क्योंकि दाल या दाल से सम्बंदित भोज कम समय में बन जाता है।
- ☐ क्योंकि मैं दाल खाने में आनंद महसूस करता हूँ।
- ☐ क्योंकि मैं निराश रहता हूँ।
- ☐ दाल खाना मेरी परंपरा का हिस्सा है।
- ☐ मैं अपने आपको पुरस्कृत करने के लिए दाल खाता हूँ।
- ☐ क्योंकि दाल प्राकृतिक है।
- ☐ क्योंकि दाल बनाना सुविधाजनक है।
- ☐ क्योंकि दाल खाना मुझे तृप्त करता है।
- ☐ क्योंकि दाल खाना स्वास्थ्य के लिए लाभदायक है।
- ☐ क्योंकि दाल सस्ती है।
- ☐ क्योंकि दाल खाना मूझे दूसरों के सामने अच्छा दिखाता है।
- ☐ क्योंकि मैं दाल खाने का आदी हूँ।
- ☐ क्योंकि मैं भूखा रहता था।
- ☐ ताकि मैं अन्य लोगों के साथ समय बिता सकूं।
- ☐ क्योंकि दाल में कोई हानिकारक (कीटनाशक, प्रदूषक, एंटीबायोटिक) पदार्थ नहीं होता है।
- ☐ क्योंकि दाल खाना सामाजिक है।
- ☐ क्योंकि दाल बनाना आसान है।
- ☐ क्योंकि दाल दूसरों को पसंद है।
- ☐ क्योंकि मूझे ऊर्जा की जरूरत होती है।
- ☐ क्योंकि दाल ही एकमात्र विकल्प है।
- ☐ क्योंकि आलू खाना सुखदेय है।
- ☐ क्योंकि दाल में वसा कि मात्रा कम होती है।
- ☐ क्योंकि दाल का स्वाद अच्छा है।
- ☐ क्योंकि मैं बचपन से दाल खाता रहा हूँ।
- ☐ संतुलित आहार बनाए रखने के लिए।
- ☐ क्योंकि मुझे दाल पसंद है।

कृपया दाल नहीं खाने के सभी कारण बताएं। (सभी विकल्प चुने)

- ☐ मैं दाल नहीं खाना चाहता हूँ।
- ☐ दाल कि खुराक का आकार मेरे लिए उपयुक्त नहीं है।
- ☐ मैं दाल खाने से दूसरों के सामने पिछड़ा महसूस करता हूँ।
- ☐ क्योंकि दाल जैविक (आर्गेनिक) नहीं है।
- ☐ मैं आमतौर पर दाल नहीं खाता हूँ।
- ☐ मुझे नहीं लगता कि दाल एक नाश्ता है।
- ☐ दाल मे कैलोरी की मात्रा बहुत अधिक होती है।
- ☐ दाल कि कीमत बहुत अधिक होती है।
- ☐ दाल मेरी भूख मिटाने के लिए पर्याप्त नहीं है।
- ☐ दाल खाना मूझे दुखी, अकेला और कुंठित महसूस कराता है।
- ☐ दाल अन्य लोगों के आसपास खाने लायक भोजन नहीं है।
- ☐ दाल मूझे दिखने में अच्छी नहीं लगती है।
- ☐ मैं कभी दाल नहीं खाऊंगा।क्योंकि मुझे हर दिन एक ही प्रकार का भोजन खाना पसंद है।
- ☐ दाल स्थिति के लिए उपयुक्त नहीं था।
- ☐ मुझे दाल पसंद नहीं है।
- ☐ दाल मेरी भूख को तृप्त नहीं कर पाती है।
- ☐ मेरी दाल खाने कि इच्छा नहीं होती है।
- ☐ दाल स्वास्थ्यय के लिए लाभदायक नहीं है।
- ☐ दाल बनाना मुश्किल होता है।
- ☐ मेने हाल ही में दाल खायी है। और अब मैं बार-बार दाल नहीं खाना चाहता हूँ।

Reasons for Dairy food choices(CATA)

क्या आप दूध खाते हैं।

- ○ हाँ
- ○ नहीं

किन कारणों के लिए आप दूध खातें हैं। (सभी विकल्प चुने)

- ☐ क्योंकि दूध सामाजिक समारोहों को और अधिक आरामदायक बनाता है।
- ☐ क्योंकि दूध में कोई हानिकारक (कीटनाशक, प्रदूषक, एंटीबायोटिक) पदार्थ नहीं होता है।
- ☐ क्योंकि दूध मेरे लिए आकर्षक भोजन है।
- ☐ क्योंकि दूध दूसरों को पसंद है।
- ☐ क्योंकि मेरे भीतर दूध खाने कि भूख रहती है।
- ☐ क्योंकि मैं दूध हर दिन खाना चाहता हूँ।
- ☐ क्योंकि दूध ही एकमात्र विकल्प है।
- ☐ क्योंकि मैं अपने वजन पर निगरानी रखता हूँ।
- ☐ क्योंकि मैं बचपन से दूध खाता रहा हूँ।
- ☐ क्योंकि दूध कुछ स्थितियों से संबंधित है।
- ☐ ताकि मैं अन्य लोगों के साथ समय बिता सकूं।
- ☐ क्योंकि दूध बनाना आसान है।
- ☐ दुसरो को निराश करने से बचने के लिए।
- ☐ क्योंकि दूध सस्ता है।
- ☐ क्योंकि दूध का स्वाद अच्छा है।
- ☐ क्योंकि मूझे ऊर्जा की जरूरत होती है।
- ☐ क्योंकि दूध खाना सुखदेय है।
- ☐ क्योंकि दूध विक्रय बिक्री पर होता है।
- ☐ क्योंकि दूध में वसा कि मात्रा कम होती है।
- ☐ क्योंकि मैं दूध से परिचित हूँ।
- ☐ क्योंकि दूध खाना फैशनेबल है।
- ☐ क्योंकि दूध खाना सामाजिक है।
- ☐ क्योंकि मुझे दूध पसंद है।
- ☐ क्योंकि दूध सस्ता है।
- ☐ संतुलित आहार बनाए रखने के लिए।
- ☐ क्योंकि मैं दूध खाने का आदी हूँ।
- ☐ क्योंकि दूध या दूध से सम्बंदित भोज कम समय में बन जाता है।
- ☐ क्योंकि दूध खाना मूझे दूसरों के सामने अच्छा दिखाता है।
- ☐ क्योंकि मैं आमतौर पर दूध खाता हूँ।

- ☐ क्योंकि दूध बनाना सुविधाजनक है।
- ☐ क्योंकि दूध नहीं खाना अशिष्ट होता है।
- ☐ क्योंकि मैं उदास रहता हूँ।
- ☐ क्योंकि दूध खाना मुझे तृप्त करता है।
- ☐ क्योंकि मैं दूध को टीवी या विज्ञापन पर देखता हूँ।
- ☐ दूध खाना मेरी परंपरा का हिस्सा है।
- ☐ क्योंकि मैं भूखा रहता था।
- ☐ क्योंकि मैं अकेला महसूस करता हूँ।
- ☐ क्योंकि दूध मेरे शरीर को आकार में रखता है।
- ☐ क्योंकि दूध प्राकृतिक है।
- ☐ क्योंकि मूझे दूध खाना पड़ता है।
- ☐ क्योंकि दूध में कम कैलोरीज होती है।
- ☐ मैं अपने आपको पुरस्कृत करने के लिए दूध खाता हूँ।
- ☐ क्योंकि दूध कि प्रस्तुति आकर्षक होती है।
- ☐ क्योंकि मैं निराश रहता हूँ।
- ☐ क्योंकि दूध खाना स्वास्थ्य के लिए लाभदायक है।
- ☐ क्योंकि दूध जैविक होता है।
- ☐ क्योंकि मैं दूध खाने में आनंद महसूस करता हूँ।

कृपया दूध नहीं खाने के सभी कारण बताएं। (सभी विकल्प चुने)

- ☐ दूध मेरी भूख मिटाने के लिए पर्याप्त नहीं है।
- ☐ दूध मे कैलोरी की मात्रा बहुत अधिक होती है।
- ☐ मुझे नहीं लगता कि दूध एक नाश्ता है।
- ☐ मेने हाल ही में दूध खाया है। और अब मैं बार-बार दूध नहीं खाना चाहता हूँ।
- ☐ दूध कि खुराक का आकार मेरे लिए उपयुक्त नहीं है।
- ☐ दूध मेरी भूख को तृप्त नहीं कर पाता है।
- ☐ दूध अन्य लोगों के आसपास खाने लायक भोजन नहीं है।
- ☐ मैं दूध खाने से दूसरों के सामने पिछड़ा महसूस करता हूँ।

- ☐ दूध स्वास्थ्यय के लिए लाभदायक नहीं है।
- ☐ मेरी दूध खाने कि इच्छा नहीं होती है।
- ☐ दूध खाना मूझे दुखी, अकेला और कुंठित महसूस कराता है।
- ☐ क्योंकि दूध जैविक (आर्गेनिक) नहीं है।
- ☐ दूध बनाना मुश्किल होता है।
- ☐ दूध कि कीमत बहुत अधिक होती है।
- ☐ दूध मूझे दिखने में अच्छा नहीं लगता है।
- ☐ दूध स्थिति के लिए उपयुक्त नहीं था।
- ☐ मैं दूध नहीं खाना चाहता हूँ।
- ☐ मैं आमतौर पर दूध नहीं खाता हूँ।
- ☐ मैं कभी दूध नहीं खाऊंगा।क्योंकि मुझे हर दिन एक ही प्रकार का भोजन खाना पसंद है।
- ☐ मुझे दूध पसंद नहीं है।

Reasons for Fruit choices(CATA)

क्या आप केले खाते हैं।

- ○ हाँ
- ○ नहीं

किन कारणों के लिए आप केले खातें हैं। (सभी विकल्प चुने)

- ☐ क्योंकि मेरे भीतर केले खाने कि भूख रहती है।
- ☐ क्योंकि केले प्राकृतिक है।
- ☐ क्योंकि मैं केले खाने का आदी हूँ।
- ☐ क्योंकि केले कि प्रस्तुति आकर्षक होती है।
- ☐ क्योंकि केले ही एकमात्र विकल्प है।
- ☐ क्योंकि केले खाना मुझे तृप्त करता है।
- ☐ क्योंकि केले खाना सामाजिक है।
- ☐ क्योंकि केले सस्ते हैं।

- ☐ क्योंकि केले मेरे लिए आकर्षक भोजन है।
- ☐ क्योंकि मुझे केले पसंद है।
- ☐ क्योंकि केले खाना स्वास्थ्य के लिए लाभदायक है।
- ☐ क्योंकि मैं केले खाने में आनंद महसूस करता हूँ।
- ☐ क्योंकि केले नहीं खाना अशिष्ट होता है।
- ☐ मैं अपने आपको पुरस्कृत करने के लिए केले खाता हूँ।
- ☐ क्योंकि केले या केले से सम्बंदित भोज कम समय में बन जाता है।
- ☐ क्योंकि केले का स्वाद अच्छा है।
- ☐ क्योंकि मैं अकेला महसूस करता हूँ।
- ☐ क्योंकि केले में वसा कि मात्रा कम होती है।
- ☐ क्योंकि केले बनाना आसान है।
- ☐ दुसरो को निराश करने से बचने के लिए।
- ☐ क्योंकि मैं बचपन से केले खाता रहा हूँ।
- ☐ केले खाना मेरी परंपरा का हिस्सा है।
- ☐ क्योंकि केले मेरे शरीर को आकार में रखता है।
- ☐ क्योंकि केले जैविक होते है।
- ☐ क्योंकि मैं निराश रहता हूँ।
- ☐ क्योंकि केले खाना सुखदेय है।
- ☐ क्योंकि मैं केले हर दिन खाना चाहता हूँ।
- ☐ क्योंकि मैं भूखा रहता था।
- ☐ क्योंकि मैं अपने वजन पर निगरानी रखता हूँ।
- ☐ ताकि मैं अन्य लोगों के साथ समय बिता सकूं।
- ☐ क्योंकि मैं उदास रहता हूँ।
- ☐ क्योंकि केले खाना फैशनेबल है।
- ☐ क्योंकि केले विक्रय बिक्री पर होता है।
- ☐ क्योंकि केले में कोई हानिकारक (कीटनाशक, प्रदूषक, एंटीबायोटिक) पदार्थ नहीं होता है।
- ☐ क्योंकि केले दूसरों को पसंद है।
- ☐ क्योंकि मूझे ऊर्जा की जरूरत होती है।
- ☐ क्योंकि केले खाना मूझे दूसरों के सामने अच्छा दिखाता है।

- ☐ क्योंकि मैं केले से परिचित हूँ।
- ☐ क्योंकि केले सामाजिक समारोहों को और अधिक आरामदायक बनाता है।
- ☐ क्योंकि केले बनाना सुविधाजनक है।
- ☐ क्योंकि केले सस्ते है।
- ☐ क्योंकि मैं केले को टीवी या विज्ञापन पर देखता हूँ।
- ☐ क्योंकि मूझे केले खाने पड़ते हैं।
- ☐ संतुलित आहार बनाए रखने के लिए।
- ☐ क्योंकि केले कुछ स्थितियों से संबंधित है।
- ☐ क्योंकि मैं आमतौर पर केले खाता हूँ।
- ☐ क्योंकि केले में कम कैलोरीज होती है।

कृपया केले नहीं खाने के सभी कारण बताएं। (सभी विकल्प चुने)

- ☐ केले मेरी भूख को तृप्त नहीं कर पाता है।
- ☐ केले स्थिति के लिए उपयुक्त नहीं था।
- ☐ क्योंकि केले जैविक (आर्गेनिक) नहीं है।
- ☐ केले मेरी भूख मिटाने के लिए पर्याप्त नहीं है।
- ☐ केले अन्य लोगों के आसपास खाने लायक भोजन नहीं है।
- ☐ मैं कभी केले नहीं खाऊंगा।क्योंकि मुझे हर दिन एक ही प्रकार का भोजन खाना पसंद है।
- ☐ मेने हाल ही में केले खाय है। और अब मैं बार-बार केले नहीं खाना चाहता हूँ।
- ☐ मैं आमतौर पर केले नहीं खाता हूँ।
- ☐ केले बनाना मुश्किल होता है।
- ☐ केले मे कैलोरी की मात्रा बहुत अधिक होती है।
- ☐ केले कि खुराक का आकार मेरे लिए उपयुक्त नहीं है।
- ☐ मेरी केले खाने कि इच्छा नहीं होती है।
- ☐ मुझे केले पसंद नहीं है।
- ☐ केले मूझे दिखने में अच्छा नहीं लगता है।
- ☐ मैं केले खाने से दूसरों के सामने पिछड़ा महसूस करता हूँ।
- ☐ केले कि कीमत बहुत अधिक होती है।

- ☐ मुझे नहीं लगता कि केले एक नाश्ता है।
- ☐ मैं केले नहीं खाना चाहता हूँ।
- ☐ केले खाना मूझे दुखी, अकेला और कुंठित महसूस कराता है।
- ☐ केले स्वास्थ्यय के लिए लाभदायक नहीं है।

Reasons for Sweet food choices(CATA)

क्या आप गुलाब जामुन खाते हैं।

- ○ हाँ
- ○ नहीं

किन कारणों के लिए आप गुलाब जामुन खातें हैं। (सभी विकल्प चुने)

- ☐ क्योंकि गुलाब जामुन में कम कैलोरीज होती है।
- ☐ क्योंकि गुलाब जामुन प्राकृतिक है।
- ☐ क्योंकि मैं बचपन से गुलाब जामुन खाता रहा हूँ।
- ☐ क्योंकि मूझे ऊर्जा की जरूरत होती है।
- ☐ क्योंकि गुलाब जामुन मेरे शरीर को आकार में रखता है।
- ☐ क्योंकि गुलाब जामुन खाना फैशनेबल है।
- ☐ क्योंकि गुलाब जामुन में वसा कि मात्रा कम होती है।
- ☐ क्योंकि मैं अकेला महसूस करता हूँ।
- ☐ क्योंकि मेरे भीतर गुलाब जामुन खाने कि भूख रहती है।
- ☐ क्योंकि गुलाब जामुन ही एकमात्र विकल्प है।
- ☐ क्योंकि गुलाब जामुन का स्वाद अच्छा है।
- ☐ क्योंकि गुलाब जामुन में कोई हानिकारक (कीटनाशक, प्रदूषक, एंटीबायोटिक) पदार्थ नहीं होता है।
- ☐ क्योंकि गुलाब जामुन बनाना सुविधाजनक है।
- ☐ क्योंकि मूझे गुलाब जामुन खाने पड़ते हैं।
- ☐ क्योंकि मैं गुलाब जामुन से परिचित हूँ।
- ☐ क्योंकि मैं गुलाब जामुन हर दिन खाना चाहता हूँ।

- ☐ क्योंकि गुलाब जामुन मेरे लिए आकर्षक भोजन है।
- ☐ क्योंकि मुझे गुलाब जामुन पसंद है।
- ☐ क्योंकि मैं अपने वजन पर निगरानी रखता हूँ।
- ☐ क्योंकि गुलाब जामुन विक्रय बिक्री पर होता है।
- ☐ क्योंकि गुलाब जामुन कुछ स्थितियों से संबंधित है।
- ☐ क्योंकि मैं आमतौर पर गुलाब जामुन खाता हूँ।
- ☐ क्योंकि मैं निराश रहता हूँ।
- ☐ क्योंकि गुलाब जामुन दूसरों को पसंद है।
- ☐ संतुलित आहार बनाए रखने के लिए।
- ☐ क्योंकि गुलाब जामुन सस्ते है।
- ☐ गुलाब जामुन खाना मेरी परंपरा का हिस्सा है।
- ☐ क्योंकि गुलाब जामुन खाना मूझे दूसरों के सामने अच्छा दिखाता है।
- ☐ क्योंकि मैं भूखा रहता था।
- ☐ क्योंकि गुलाब जामुन नहीं खाना अशिष्ट होता है।
- ☐ ताकि मैं अन्य लोगों के साथ समय बिता सकूं।
- ☐ क्योंकि गुलाब जामुन खाना स्वास्थ्य के लिए लाभदायक है।
- ☐ क्योंकि गुलाब जामुन खाना सुखदेय है।
- ☐ क्योंकि गुलाब जामुन सामाजिक समारोहों को और अधिक आरामदायक बनाता है।
- ☐ क्योंकि गुलाब जामुन कि प्रस्तुति आकर्षक होती है।
- ☐ क्योंकि मैं गुलाब जामुन को टीवी या विज्ञापन पर देखता हूँ।
- ☐ क्योंकि मैं गुलाब जामुन खाने का आदी हूँ।
- ☐ क्योंकि गुलाब जामुन जैविक होते है।
- ☐ क्योंकि गुलाब जामुन सस्ते हैं।
- ☐ क्योंकि मैं उदास रहता हूँ।
- ☐ दुसरो को निराश करने से बचने के लिए।
- ☐ क्योंकि मैं गुलाब जामुन खाने में आनंद महसूस करता हूँ।
- ☐ क्योंकि गुलाब जामुन खाना मुझे तृप्त करता है।
- ☐ क्योंकि गुलाब जामुन बनाना आसान है।
- ☐ क्योंकि गुलाब जामुन कम समय में बन जाता है।

- ☐ मैं अपने आपको पुरस्कृत करने के लिए गुलाब जामुन खाता हूँ।
- ☐ क्योंकि गुलाब जामुन खाना सामाजिक है।

कृपया गुलाब जामुन नहीं खाने के सभी कारण बताएं। (सभी विकल्प चुने)

- ☐ गुलाब जामुन मेरी भूख को तृप्त नहीं कर पाता है।
- ☐ क्योंकि गुलाब जामुन जैविक (आर्गेनिक) नहीं है।
- ☐ गुलाब जामुन खाना मूझे दुखी, अकेला और कुंठित महसूस कराता है।
- ☐ मैं गुलाब जामुन नहीं खाना चाहता हूँ।
- ☐ गुलाब जामुन अन्य लोगों के आसपास खाने लायक भोजन नहीं है।
- ☐ मेने हाल ही में गुलाब जामुन खाय है। और अब मैं बार-बार गुलाब जामुन नहीं खाना चाहता हूँ।
- ☐ मुझे नहीं लगता कि गुलाब जामुन एक नाश्ता है।
- ☐ गुलाब जामुन मूझे दिखने में अच्छा नहीं लगता है।
- ☐ गुलाब जामुन मे कैलोरी की मात्रा बहुत अधिक होती है।
- ☐ मैं कभी गुलाब जामुननहीं खाऊंगा।क्योंकि मुझे हर दिन एक ही प्रकार का भोजन खाना पसंद है।
- ☐ गुलाब जामुन स्थिति के लिए उपयुक्त नहीं था।
- ☐ गुलाब जामुन कि कीमत बहुत अधिक होती है।
- ☐ गुलाब जामुन स्वास्थ्यय के लिए लाभदायक नहीं है।
- ☐ मुझे गुलाब जामुन पसंद नहीं है।
- ☐ गुलाब जामुन कि खुराक का आकार मेरे लिए उपयुक्त नहीं है।
- ☐ मेरी गुलाब जामुन खाने कि इच्छा नहीं होती है।
- ☐ गुलाब जामुन मेरी भूख मिटाने के लिए पर्याप्त नहीं है।
- ☐ गुलाब जामुन बनाना मुश्किल होता है।
- ☐ मैं गुलाब जामुन खाने से दूसरों के सामने पिछड़ा महसूस करता हूँ।
- ☐ मैं आमतौर पर गुलाब जामुन नहीं खाता हूँ।

JAR & Liking

यह सर्वेक्षण आपको कितना संक्षिप्त या विस्तृत लगा।

- बहुत अधिक संक्षिप्त
- बहुत संक्षिप्त
- थोड़ा बहुत संक्षिप्त
- ठीक ठाक
- थोड़ा बहुत विस्तृत
- बहुत विस्तृत
- बहुत अधिक विस्तृत

यह सर्वेक्षण आपको कैसा लगा

- मुझे सर्वेक्षण बहुत अधिक पसंद आया
- मुझे सर्वेक्षण पसंद आया
- मुझे सर्वेक्षण ना अच्छा लगा और ना ही ख़राब लगा
- मुझे सर्वेक्षण पसंद नहीं आया
- मुझे सर्वेक्षण बहुत ख़राब लगा

Reasons for Starchy food choices(Full CATA)

क्या आप चावल खाते हैं।

- हाँ
- नहीं

आप चावल खाते हैं। क्योंकि आप चावल खाने के आदी हैं।

- हाँ
- नहीं

आप चावल खाते हैं। क्योंकि चावल खाना स्वास्थ्य के लिए लाभदायक हैं।

- हाँ
- नहीं

आप चावल खाते हैं। क्योंकि चावल कम समय में बन जाता है।

- हाँ
- नहीं

आप चावल खाते हैं। क्योंकि चावल का स्वाद अच्छा है।

- हाँ
- नहीं

आप चावल खाते हैं। क्योंकि चावल मुझे तृप्त करता है।

- हाँ
- नहीं

आप चावल खाते हैं। क्योंकि चावल प्राकृतिक है।

- हाँ
- नहीं

आप चावल खाते हैं। क्योंकि आप अधिक पैसे खर्च नहीं करना चाहते है।

- हाँ
- नहीं

आप चावल खाते हैं। क्योंकि चावल में कम कैलोरीज होती हैं।

- हाँ
- नहीं

आप चावल खाते हैं। क्योंकि आप निराश रहते हैं। ।

- हाँ
- नहीं

आप चावल खाते हैं। क्योंकि चावल खाना आपको दूसरों के सामने अच्छा दिखाता हैं।

- हाँ
- नहीं

आप चावल खाते हैं। क्योंकि चावल नहीं खाना अशिष्ट होगा।

- हाँ
- नहीं

आप चावल खाते हैं। क्योंकि चावल कि प्रस्तुति आकर्षक होती है।

- हाँ
- नहीं

आप चावल खाते हैं। ताकि आप अन्य लोगों के साथ समय बिता सके।

- हाँ
- नहीं

आप चावल खाते हैं। क्योंकि चावल जैविक होता है।

- हाँ
- नहीं

आप चावल खाते हैं। क्योंकि आप बचपन से चावल खाते रहे हैं।

- हाँ
- नहीं

आप चावल खाते हैं। क्योंकि आप चावल खाने में आनंद महसूस करते हैं।

- हाँ
- नहीं

आप चावल खाते हैं। क्योंकि आप भूखे रहते हैं।

- हाँ
- नहीं

आप चावल खाते हैं। क्योंकि आप आमतौर पर चावल खाते हैं।

- हाँ
- नहीं

आप चावल खाते हैं। क्योंकि आपके भीतर चावल खाने कि भूख रहती है।

- हाँ
- नहीं

आप चावल खाते हैं। क्योंकि चावल तैयार करना आसान है।

- हाँ
- नहीं

आप चावल खाते हैं। क्योंकि आप उदास रहते हैं।

- हाँ
- नहीं

आप चावल खाते हैं। क्योंकि चावल में वसा की मात्रा कम होती है।

- हाँ
- नहीं

आप चावल खाते हैं। क्योंकि आप चावल को टीवी या विज्ञापन पर देखते हैं।

- हाँ
- नहीं

आप चावल खाते हैं। क्योंकि चावल सामाजिक समारोहों को और अधिक आरामदायक बनाता है।

- हाँ
- नहीं

आप चावल खाते हैं। क्योंकि आपको चावल खाना पड़ता है।

- हाँ
- नहीं

आप चावल खाते हैं। क्योंकि चावल दूसरों को पसंद है।

- हाँ
- नहीं

आप चावल खाते हैं। क्योंकि आप अकेला महसूस करते हैं।

- हाँ
- नहीं

आप चावल खाते हैं। क्योंकि आप अपने वजन पर निगरानी रखते हैं।

- हाँ
- नहीं

आप चावल खाते हैं। क्योंकि चावल में कोई हानिकारक (कीटनाशक, प्रदूषक, एंटीबायोटिक) पदार्थ नहीं होता है।

- हाँ
- नहीं

आप चावल खाते हैं। क्योंकि चावल बनाना सुविधाजनक है।

- हाँ
- नहीं

आप चावल खाते हैं। क्योंकि मूझे ऊर्जा की जरूरत होती है।

- हाँ
- नहीं

आप चावल खाते हैं। क्योंकि आप चावल से परिचित है।

- हाँ
- नहीं

आप चावल खाते हैं। क्योंकि आपको चावल पसंद है।

- हाँ
- नहीं

आप चावल खाते हैं। क्योंकि चावल खाना सुखदेय है।

- हाँ
- नहीं

आप चावल अपने आपको पुरस्कृत करने के लिए चावल खाते हैं।

- हाँ
- नहीं

आप चावल खाते हैं। क्योंकि चावल खाना आपकी परंपरा का हिस्सा है।

- हाँ
- नहीं

आप चावल खाते हैं। क्योंकि यह कुछ स्थितियों से संबंधित है।

- हाँ
- नहीं

आप चावल संतुलित आहार पाने के लिए खाते हैं।

- हाँ
- नहीं

आप चावल खाते हैं। क्योंकि चावल खाना सामाजिक है।

- हाँ
- नहीं

आप चावल खाते हैं। क्योंकि चावल सस्ता है।

- हाँ
- नहीं

आप चावल खाते हैं। क्योंकि चावल आपके लिए आकर्षक भोजन है।

- हाँ
- नहीं

दुसरो को निराश करने से बचने के लिए आप चावल खाते हैं।

- हाँ
- नहीं

आप चावल खाते हैं। क्योंकि चावल खाना फैशनेबल है।

- हाँ
- नहीं

आप चावल खाते हैं। क्योंकि चावल सेल पर उपलब्ध होता है।

- हाँ
- नहीं

आप चावल खाते हैं। क्योंकि यह आपके शरीर को आकार में रखता है।

- हाँ

○ नहीं

आप चावल खाते हैं। क्योंकि चावल ही एकमात्र विकल्प था।

○ हाँ
○ नहीं

आप चावल खाते हैं। क्योंकि आप चावल हर दिन खाना चाहते हैं।

○ हाँ
○ नहीं

आप चावल नहीं खाते हैं। क्योंकि आपको नहीं लगता कि चावल एक नाश्ता है।

○ हाँ
○ नहीं

आप चावल नहीं खाते हैं। क्योंकि आपको चावल पसंद नहीं है।

○ हाँ
○ नहीं

आप चावल नहीं खाते हैं। क्योंकि आप आमतौर पर चावल नहीं खाते हैं।

○ हाँ
○ नहीं

आप चावल नहीं खाते हैं। क्योंकि चावल कि खुराक का आकार मेरे लिए उपयुक्त नहीं होता है।

- हाँ
- नहीं

आप चावल नहीं खाते हैं। क्योंकि चावल आपकी भूख मिटाने के लिए पर्याप्त नहीं होता है।

- हाँ
- नहीं

आप चावल नहीं खाते हैं। क्योंकि चावल स्वास्थ्यय के लिए लाभदायक नहीं है।

- हाँ
- नहीं

आप चावल नहीं खाते हैं। क्योंकि चावल बनाना मुश्किल होता है।

- हाँ
- नहीं

आप चावल नहीं खाते हैं। क्योंकि चावल आपकी भूख को तृप्त नहीं कर पाता है।

- हाँ
- नहीं

आप चावल नहीं खाते हैं। क्योंकि चावल स्थिति के लिए उपयुक्त नहीं होता है।

- हाँ
- नहीं

आप चावल नहीं खाते हैं। क्योंकि चावल अन्य लोगों के आसपास खाने लायक भोजन नहीं है।

- हाँ
- नहीं

आप चावल नहीं खाते हैं। क्योंकि चावल कि कीमत बहुत अधिक है।

- हाँ
- नहीं

आप चावल नहीं खाते हैं। क्योंकि चावल आपको दिखने में अच्छा नहीं लगता है।

- हाँ
- नहीं

आप चावल नहीं खाते हैं। क्योंकि चावल मे कैलोरी के मात्रा बहुत अधिक होती है।

- हाँ
- नहीं

आप चावल नहीं खाते हैं। क्योंकि आप चावल नहीं खाना चाहते हैं।

- हाँ
- नहीं

आप चावल नहीं खाते हैं। क्योंकि आप चावल खाने से दूसरों के सामने पिछड़ा महसूस करते हैं।

- हाँ
- नहीं

आप चावल नहीं खाते हैं। क्योंकि आपकी चावल खाने कि इच्छा नहीं होती है।

- हाँ
- नहीं

आप चावल नहीं खाते हैं। क्योंकि अपने हाल ही में चावल खाया था और अब आप बार-बार चावल नहीं खाना चाहते हैं।

- हाँ
- नहीं

आप चावल नहीं खाते हैं। क्योंकि आपको हर दिन एक ही प्रकार का भोजन खाना पसंद हैं।

- हाँ
- नहीं

आप चावल नहीं खाते हैं। क्योंकि चावल खाना आपको दुखी, अकेला और कुंठित महसूस कराता हैं।

- हाँ
- नहीं

आप चावल नहीं खाते हैं। क्योंकि चावल जैविक (आर्गेनिक) नहीं हैं।

- हाँ
- नहीं

Reasons for Protein food choices(Full CATA)

क्या आप दाल खाते हैं ।

- हाँ
- नहीं

आप दाल खाते हैं। क्योंकि आप दाल खाने के आदी हैं।

- हाँ
- नहीं

आप दाल खाते हैं। क्योंकि दाल खाना स्वास्थय के लिए लाभदायक हैं।

- हाँ
- नहीं

आप दाल खाते हैं। क्योंकि दाल कम समय में बन जाती है।

- हाँ
- नहीं

आप दाल खाते हैं। क्योंकि दाल का स्वाद अच्छा है।

- हाँ
- नहीं

आप दाल खाते हैं। क्योंकि दाल मुझे तृप्त करती है।

- हाँ
- नहीं

आप दाल खाते हैं। क्योंकि दाल प्राकृतिक है।

- हाँ
- नहीं

आप दाल खाते हैं। क्योंकि आप अधिक पैसे खर्च नहीं करना चाहते।

- हाँ
- नहीं

आप दाल खाते हैं। क्योंकि दाल में कम कैलोरीज होती हैं।

- हाँ
- नहीं

आप दाल खाते हैं। क्योंकि आप निराश रहते हैं।

- हाँ
- नहीं

आप दाल खाते हैं। क्योंकि दाल खाना आपको दूसरों के सामने अच्छा दिखाता हैं।

- हाँ
- नहीं

आप दाल खाते हैं। क्योंकि दाल नहीं खाना अशिष्ट होगा।

- हाँ
- नहीं

आप दाल खाते हैं। क्योंकि दाल कि प्रस्तुति आकर्षक होती है।

- हाँ
- नहीं

आप दाल खाते हैं। ताकि आप अन्य लोगों के साथ समय बिता सके।

- हाँ
- नहीं

आप दाल खाते हैं। क्योंकि दाल जैविक होती है।

- हाँ
- नहीं

आप दाल खाते हैं। क्योंकि आप बचपन से दाल खाते रहे है।

- हाँ
- नहीं

आप दाल खाते हैं। क्योंकि आप दाल खाने में आनंद महसूस करते है।

- हाँ
- नहीं

आप दाल खाते हैं। क्योंकि आप भूखे रहते हैं।

- हाँ
- नहीं

आप दाल खाते हैं। क्योंकि आप आमतौर पर दाल खाते हैं।

- हाँ
- नहीं

आप दाल खाते हैं। क्योंकि आपके भीतर दाल खाने कि भूख रहती है।

- हाँ
- नहीं

आप दाल खाते हैं। क्योंकि दाल बनाना आसान है।

- हाँ
- नहीं

आप दाल खाते हैं। क्योंकि आप उदास रहते हैं।

- हाँ
- नहीं

आप दाल खाते हैं। क्योंकि दाल में वसा कि मात्रा कम होती है।

- हाँ
- नहीं

आप दाल खाते हैं। क्योंकि आप दाल को टीवी या विज्ञापन पर देखते हैं।

- हाँ
- नहीं

आप दाल खाते हैं। क्योंकि दाल सामाजिक समारोहों को अधिक आरामदायक बनाता है।

- हाँ
- नहीं

आप दाल खाते हैं। क्योंकि आपको दाल खानी पड़ती है।

- हाँ
- नहीं

आप दाल खाते हैं। क्योंकि दाल दूसरों को पसंद है।

- हाँ
- नहीं

आप दाल खाते हैं। क्योंकि आप अकेला महसूस करते हैं।

- हाँ
- नहीं

आप दाल खाते हैं। क्योंकि आप अपने वजन पर निगरानी रखते हैं।

- हाँ
- नहीं

आप दाल खाते हैं। क्योंकि दाल में कोई हानिकारक (कीटनाशक, प्रदूषक, एंटीबायोटिक) पदार्थ नहीं होता है।

- हाँ
- नहीं

आप दाल खाते हैं। क्योंकि दाल बनाना सुविधाजनक है।

- हाँ

- नहीं

आप दाल खाते हैं। क्योंकि मूझे ऊर्जा की जरूरत होती है।

- हाँ
- नहीं

आप दाल खाते हैं। क्योंकि आप दाल से परिचित है।

- हाँ
- नहीं

आप दाल खाते हैं। क्योंकि आपको दाल पसंद है।

- हाँ
- नहीं

आप दाल खाते हैं। क्योंकि दाल खाना सुखदेय है।

- हाँ
- नहीं

आप दाल अपने आपको पुरस्कृत करने के लिए दाल खाते हैं।

- हाँ
- नहीं

आप दाल खाते हैं। क्योंकि दाल खाना आपकी परंपरा का हिस्सा है।

- हाँ
- नहीं

आप दाल खाते हैं। क्योंकि यह कुछ स्थितियों से संबंधित है।

- हाँ
- नहीं

आप दाल संतुलित आहार पाने के लिए खाते हैं।

- हाँ
- नहीं

आप दाल खाते हैं। क्योंकि दाल खाना सामाजिक है।

- हाँ
- नहीं

आप दाल खाते हैं। क्योंकि दाल सस्ती है।

- हाँ
- नहीं

आप दाल खाते हैं। क्योंकि दाल आपके लिए आकर्षक भोजन है।

- हाँ
- नहीं

दुसरो को निराश करने से बचने के लिए आप दाल खाते हैं।

- हाँ
- नहीं

आप दाल खाते हैं। क्योंकि दाल खाना फैशनेबल है।

- हाँ
- नहीं

आप दाल खाते हैं। क्योंकि दाल सेल पर होती है।

- हाँ
- नहीं

आप दाल खाते हैं। क्योंकि यह आपके शरीर को आकार में रखता है।

- हाँ
- नहीं

आप दाल खाते हैं। क्योंकि दाल ही एकमात्र विकल्प था।

- हाँ
- नहीं

आप दाल खाते हैं। क्योंकि आप दाल हर दिन खाना चाहते है।

- हाँ
- नहीं

आप दाल नहीं खाते हैं। क्योंकि आपको नहीं लगता कि दाल एक नाश्ता है।

- हाँ
- नहीं

आप दाल नहीं खाते हैं। क्योंकि आपको दाल पसंद नहीं है।

- हाँ
- नहीं

आप दाल नहीं खाते हैं। क्योंकि आप आमतौर पर दाल नहीं खाते है।

- हाँ
- नहीं

आप दाल नहीं खाते हैं। क्योंकि दाल कि खुराक का आकार मेरे लिए उपयुक्त नहीं होता है।

- हाँ
- नहीं

आप दाल नहीं खाते हैं। क्योंकि दाल आपकी भूख मिटाने के लिए पर्याप्त नहीं होता है।

- हाँ
- नहीं

आप दाल नहीं खाते हैं। क्योंकि दाल स्वास्थ्यय के लिए लाभदायक नहीं है।

- हाँ
- नहीं

आप दाल नहीं खाते हैं। क्योंकि दाल बनाना मुश्किल होता है।

- हाँ
- नहीं

आप दाल नहीं खाते हैं। क्योंकि दाल आपकी भूख को तृप्त नहीं कर पाता है।

- हाँ
- नहीं

आप दाल नहीं खाते हैं। क्योंकि दाल स्थिति के लिए उपयुक्त नहीं होती है।

- हाँ
- नहीं

आप दाल नहीं खाते हैं। क्योंकि दाल अन्य लोगों के आसपास खाने लायक भोजन नहीं है।

- हाँ
- नहीं

आप दाल नहीं खाते हैं। क्योंकि दाल कि कीमत बहुत अधिक है।

- हाँ
- नहीं

आप दाल नहीं खाते हैं। क्योंकि दाल आपको दिखने में अच्छी नहीं लगती है।

- हाँ
- नहीं

आप दाल नहीं खाते हैं। क्योंकि दाल मे कैलोरी के मात्रा बहुत अधिक होती है।

- हाँ
- नहीं

आप दाल नहीं खाते हैं। क्योंकि आप दाल नहीं खाना चाहते है।

- हाँ
- नहीं

आप दाल नहीं खाते हैं। क्योंकि आप दाल खाने से दूसरों के सामने पिछड़ा महसूस करते हैं।

- हाँ
- नहीं

आप दाल नहीं खाते हैं। क्योंकि आपकी दाल खाने कि इच्छा नहीं होती हैं।

- हाँ
- नहीं

आप दाल नहीं खाते हैं। क्योंकि अपने हाल ही में दाल खाया था और अब आप बार-बार दाल नहीं खाना चाहते हैं।

- हाँ
- नहीं

आप दाल नहीं खाते हैं। क्योंकि आपको हर दिन एक ही प्रकार का भोजन खाना पसंद है।

- हाँ
- नहीं

आप दाल नहीं खाते हैं। क्योंकि दाल खाना आपको दुखी, अकेला और कुंठित महसूस कराता है।

- हाँ
- नहीं

आप दाल नहीं खाते हैं। क्योंकि दाल जैविक (आर्गेनिक) नहीं है।

- हाँ
- नहीं

Reasons for Dairy food choices(Full CATA)

क्या आप दूध खाते हैं।

- हाँ
- नहीं

आप दूध खाते हैं। क्योंकि आप दूध खाने के आदी हैं।

- हाँ
- नहीं

आप दूध खाते हैं। क्योंकि दूध खाना स्वास्थय के लिए लाभदायक हैं।

- हाँ
- नहीं

आप दूध खाते हैं। क्योंकि दूध कम समय में बन जाता है।

- हाँ
- नहीं

आप दूध खाते हैं। क्योंकि दूध का स्वाद अच्छा है।

- हाँ
- नहीं

आप दूध खाते हैं। क्योंकि दूध मुझे तृप्त करता है।

- हाँ
- नहीं

आप दूध खाते हैं। क्योंकि दूध प्राकृतिक है।

- हाँ

○ नहीं

आप दूध खाते हैं। क्योंकि आप अधिक पैसे खर्च नहीं करना चाहते है।

○ हाँ
○ नहीं

आप दूध खाते हैं। क्योंकि दूध में कम कैलोरीज होती हैं।

○ हाँ
○ नहीं

आप दूध खाते हैं। क्योंकि आप निराश रहते हैं।

○ हाँ
○ नहीं

आप दूध खाते हैं। क्योंकि दूध खाना आपको दूसरों के सामने अच्छा दिखाता हैं।

○ हाँ
○ नहीं

आप दूध खाते हैं। क्योंकि दूध नहीं खाना अशिष्ट होता है।

○ हाँ
○ नहीं

आप दूध खाते हैं। क्योंकि दूध कि प्रस्तुति आकर्षक होती है।

○ हाँ
○ नहीं

आप दूध खाते हैं। ताकि आप अन्य लोगों के साथ समय बिता सके।

- हाँ
- नहीं

आप दूध खाते हैं। क्योंकि दूध जैविक होता है।

- हाँ
- नहीं

आप दूध खाते हैं। क्योंकि आप बचपन से दूध खाते रहे है।

- हाँ
- नहीं

आप दूध खाते हैं। क्योंकि आप दूध खाने में आनंद महसूस करते है।

- हाँ
- नहीं

आप दूध खाते हैं। क्योंकि आप भूखे रहते हैं।

- हाँ
- नहीं

आप दूध खाते हैं। क्योंकि आप आमतौर पर दूध खाते है।

- हाँ
- नहीं

आप दूध खाते हैं। क्योंकि आपके भीतर दूध खाने कि भूख रहती है।

- हाँ
- नहीं

आप दूध खाते हैं। क्योंकि दूध तैयार करना आसान है।

- हाँ
- नहीं

आप दूध खाते हैं। क्योंकि आप उदास रहते है।

- हाँ
- नहीं

आप दूध खाते हैं। क्योंकि दूध में वसा कि मात्रा कम होती है।

- हाँ
- नहीं

आप दूध खाते हैं। क्योंकि आप दूध को टीवी या विज्ञापन पर देखते है।

- हाँ
- नहीं

आप दूध खाते हैं। क्योंकि दूध सामाजिक समारोहों को अधिक आरामदायक बनाता है।

- हाँ
- नहीं

आप दूध खाते हैं। क्योंकि आपको दूध खाना पड़ता है।

- हाँ

- नहीं

आप दूध खाते हैं। क्योंकि दूध दूसरों को पसंद है।

- हाँ
- नहीं

आप दूध खाते हैं। क्योंकि आप अकेला महसूस करते हैं।

- हाँ
- नहीं

आप दूध खाते हैं। क्योंकि आप अपने वजन पर निगरानी रखते हैं।

- हाँ
- नहीं

आप दूध खाते हैं। क्योंकि दूध में कोई हानिकारक (कीटनाशक, प्रदूषक, एंटीबायोटिक) पदार्थ नहीं होता है।

- हाँ
- नहीं

आप दूध खाते हैं। क्योंकि दूध बनाना सुविधाजनक है।

- हाँ
- नहीं

आप दूध खाते हैं। क्योंकि मूझे ऊर्जा की जरूरत होती है।

- हाँ
- नहीं

आप दूध खाते हैं। क्योंकि आप दूध से परिचित है।

- हाँ
- नहीं

आप दूध खाते हैं। क्योंकि आपको दूध पसंद है।

- हाँ
- नहीं

आप दूध खाते हैं। क्योंकि दूध खाना सुखदेय है।

- हाँ
- नहीं

आप दूध अपने आपको पुरस्कृत करने के लिए दूध खाते हैं।

- हाँ
- नहीं

आप दूध खाते हैं। क्योंकि दूध खाना आपकी परंपरा का हिस्सा है।

- हाँ
- नहीं

आप दूध खाते हैं। क्योंकि यह कुछ स्थितियों से संबंधित है।

- हाँ
- नहीं

आप दूध संतुलित आहार पाने के लिए खाते हैं।

- हाँ
- नहीं

आप दूध खाते हैं। क्योंकि दूध खाना सामाजिक है।

- हाँ
- नहीं

आप दूध खाते हैं। क्योंकि दूध सस्ता है।

- हाँ
- नहीं

आप दूध खाते हैं। क्योंकि दूध आपके लिए आकर्षक भोजन है।

- हाँ
- नहीं

दुसरो को निराश करने से बचने के लिए आप दूध खाते हैं।

- हाँ
- नहीं

आप दूध खाते हैं। क्योंकि दूध खाना फैशनेबल है।

- हाँ
- नहीं

आप दूध खाते हैं। क्योंकि दूध सेल पर उपलब्ध होता है।

- हाँ

- नहीं

आप दूध खाते हैं। क्योंकि दूध आपके शरीर को आकार में रखता है।

- हाँ
- नहीं

आप दूध खाते हैं। क्योंकि दूध ही एकमात्र विकल्प था।

- हाँ
- नहीं

आप दूध खाते हैं। क्योंकि आप दूध हर दिन खाना चाहते है।

- हाँ
- नहीं

आप दूध नहीं खाते हैं। क्योंकि आपको नहीं लगता कि दूध एक नाश्ता है।

- हाँ
- नहीं

आप दूध नहीं खाते हैं। क्योंकि आपको दूध पसंद नहीं है।

- हाँ
- नहीं

आप दूध नहीं खाते हैं। क्योंकि आप आमतौर पर दूध नहीं खाते है।

- हाँ
- नहीं

आप दूध नहीं खाते हैं। क्योंकि दूध कि खुराक का आकार मेरे लिए उपयुक्त नहीं होता है।

- हाँ
- नहीं

आप दूध नहीं खाते हैं। क्योंकि दूध आपकी भूख मिटाने के लिए पर्याप्त नहीं होता है।

- हाँ
- नहीं

आप दूध नहीं खाते हैं। क्योंकि दूध स्वास्थ्यय के लिए लाभदायक नहीं है।

- हाँ
- नहीं

आप दूध नहीं खाते हैं। क्योंकि दूध बनाना मुश्किल होता है।

- हाँ
- नहीं

आप दूध नहीं खाते हैं। क्योंकि दूध आपकी भूख को तृप्त नहीं कर पाता है।

- हाँ
- नहीं

आप दूध नहीं खाते हैं। क्योंकि दूध स्थिति के लिए उपयुक्त नहीं होता है।

- हाँ
- नहीं

आप दूध नहीं खाते हैं। क्योंकि दूध अन्य लोगों के आसपास खाने लायक भोजन नहीं है।

- हाँ
- नहीं

आप दूध नहीं खाते हैं। क्योंकि दूध कि कीमत बहुत अधिक है।

- हाँ
- नहीं

आप दूध नहीं खाते हैं। क्योंकि दूध आपको दिखने में अच्छा नहीं लगता है।

- हाँ
- नहीं

आप दूध नहीं खाते हैं। क्योंकि दूध मे कैलोरी के मात्रा बहुत अधिक होती है।

- हाँ
- नहीं

आप दूध नहीं खाते हैं। क्योंकि आप दूध नहीं खाना चाहते है।

- हाँ
- नहीं

आप दूध नहीं खाते हैं। क्योंकि आप दूध खाने से दूसरों के सामने पिछड़ा महसूस करते है।

- हाँ
- नहीं

आप दूध नहीं खाते हैं। क्योंकि आपकी दूध खाने कि इच्छा नहीं होती है।

- हाँ
- नहीं

आप दूध नहीं खाते हैं। क्योंकि अपने हाल ही में दूध खाया था और अब आप बार-बार दूध नहीं खाना चाहते है।

- हाँ
- नहीं

आप दूध नहीं खाते हैं। क्योंकि आपको हर दिन एक ही प्रकार का भोजन खाना पसंद है।

- हाँ
- नहीं

आप दूध नहीं खाते हैं। क्योंकि दूध खाना आपको दुखी, अकेला और कुंठित महसूस कराता है।

- हाँ
- नहीं

आप दूध नहीं खाते हैं। क्योंकि दूध जैविक (आर्गेनिक) नहीं है।

- हाँ
- नहीं

Reasons for Fruit choices(Full CATA)

क्या आप केले खाते हैं।

- हाँ
- नहीं

आप केले खाते हैं। क्योंकि आप केले खाने के आदी हैं।

- हाँ
- नहीं

आप केले खाते हैं। क्योंकि केले खाना स्वास्थय के लिए लाभदायक हैं।

- हाँ
- नहीं

आप केले खाते हैं। क्योंकि केले कम समय में बन जाते है।

- हाँ
- नहीं

आप केले खाते हैं। क्योंकि केले का स्वाद अच्छा है।

- हाँ
- नहीं

आप केले खाते हैं। क्योंकि केले मुझे तृप्त करते है।

- हाँ
- नहीं

आप केले खाते हैं। क्योंकि केले प्राकृतिक है।

- हाँ
- नहीं

आप केले खाते हैं। क्योंकि आप अधिक पैसे खर्च नहीं करना चाहते।

- हाँ
- नहीं

आप केले खाते हैं। क्योंकि केले में कम कैलोरीज होती हैं।

- हाँ
- नहीं

आप केले खाते हैं। क्योंकि आप निराश रहते हैं।

- हाँ
- नहीं

आप केले खाते हैं। क्योंकि केले खाना आपको दूसरों के सामने अच्छा दिखाता हैं।

- हाँ
- नहीं

आप केले खाते हैं। क्योंकि केले नहीं खाना अशिष्ट होता है।

- हाँ
- नहीं

आप केले खाते हैं। क्योंकि केले कि प्रस्तुति आकर्षक होती है।

- हाँ
- नहीं

आप केले खाते हैं। ताकि आप अन्य लोगों के साथ समय बिता सके।

- हाँ
- नहीं

आप केले खाते हैं। क्योंकि केले जैविक होते है।

- हाँ
- नहीं

आप केले खाते हैं। क्योंकि आप बचपन से केले खाते रहे है।

- हाँ
- नहीं

आप केले खाते हैं। क्योंकि आप केले खाने में आनंद महसूस करते है।

- हाँ
- नहीं

आप केले खाते हैं। क्योंकि आप भूखे रहते है।

- हाँ
- नहीं

आप केले खाते हैं। क्योंकि आप आमतौर पर केले खाते है।

- हाँ
- नहीं

आप केले खाते हैं। क्योंकि आपके भीतर केले खाने कि भूख रहती है।

- हाँ
- नहीं

आप केले खाते हैं। क्योंकि केले तैयार करना आसान है।

- हाँ
- नहीं

आप केले खाते हैं। क्योंकि आप उदास रहते है।

- हाँ
- नहीं

आप केले खाते हैं। क्योंकि केले में वसा कि मात्रा कम होती है।

- हाँ
- नहीं

आप केले खाते हैं। क्योंकि आप केले को टीवी या विज्ञापन पर देखते है।

- हाँ
- नहीं

आप केले खाते हैं। क्योंकि केले सामाजिक समारोहों को अधिक आरामदायक बनाता है।

- हाँ
- नहीं

आप केले खाते हैं। क्योंकि आपको केले खाने पड़ते है।

- हाँ
- नहीं

आप केले खाते हैं। क्योंकि केले दूसरों को पसंद है।

- हाँ

- नहीं

आप केले खाते हैं। क्योंकि आप अकेला महसूस करते हैं।

- हाँ
- नहीं

आप केले खाते हैं। क्योंकि आप अपने वजन पर निगरानी रखते हैं।

- हाँ
- नहीं

आप केले खाते हैं। क्योंकि केले में कोई हानिकारक (कीटनाशक, प्रदूषक, एंटीबायोटिक) पदार्थ नहीं होता है।

- हाँ
- नहीं

आप केले खाते हैं। क्योंकि केले बनाना सुविधाजनक है।

- हाँ
- नहीं

आप केले खाते हैं। क्योंकि मूझे ऊर्जा की जरूरत होती है।

- हाँ
- नहीं

आप केले खाते हैं। क्योंकि आप केले से परिचित है।

- हाँ
- नहीं

आप केले खाते हैं। क्योंकि आपको केले पसंद है।

- हाँ
- नहीं

आप केले खाते हैं। क्योंकि केले खाना सुखदेय है।

- हाँ
- नहीं

आप केले अपने आपको पुरस्कृत करने के लिए केले खाते हैं।

- हाँ
- नहीं

आप केले खाते हैं। क्योंकि केले खाना आपकी परंपरा का हिस्सा है।

- हाँ
- नहीं

आप केले खाते हैं। क्योंकि यह कुछ स्थितियों से संबंधित है।

- हाँ
- नहीं

आप केले संतुलित आहार पाने के लिए खाते हैं।

- हाँ
- नहीं

आप केले खाते हैं। क्योंकि केले खाना सामाजिक है।

- हाँ
- नहीं

आप केले खाते हैं। क्योंकि केले सस्ते है।

- हाँ
- नहीं

आप केले खाते हैं। क्योंकि केले आपके लिए आकर्षक भोजन है।

- हाँ
- नहीं

दुसरो को निराश करने से बचने के लिए आप केले खाते हैं।

- हाँ
- नहीं

आप केले खाते हैं। क्योंकि केले खाना फैशनेबल है।

- हाँ
- नहीं

आप केले खाते हैं। क्योंकि केले सेल पर उपलब्ध होते हैं।

- हाँ
- नहीं

आप केले खाते हैं। क्योंकि केले आपके शरीर को आकार में रखता है।

- हाँ

○ नहीं

आप केले खाते हैं। क्योंकि केले ही एकमात्र विकल्प था।

○ हाँ
○ नहीं

आप केले खाते हैं। क्योंकि आप केले हर दिन खाना चाहते है।

○ हाँ
○ नहीं

आप केले नहीं खाते हैं। क्योंकि आपको नहीं लगता कि केले एक नाश्ता है।

○ हाँ
○ नहीं

आप केले नहीं खाते हैं। क्योंकि आपको केले पसंद नहीं है।

○ हाँ
○ नहीं

आप केले नहीं खाते हैं। क्योंकि आप आमतौर पर केले नहीं खाते है।

○ हाँ
○ नहीं

आप केले नहीं खाते हैं। क्योंकि केले कि खुराक का आकार मेरे लिए उपयुक्त नहीं होता है।

○ हाँ
○ नहीं

आप केले नहीं खाते हैं। क्योंकि केले आपकी भूख मिटाने के लिए पर्याप्त नहीं होता है।

- हाँ
- नहीं

आप केले नहीं खाते हैं। क्योंकि केले स्वास्थ्यय के लिए लाभदायक नहीं है।

- हाँ
- नहीं

आप केले नहीं खाते हैं। क्योंकि केले बनाना मुश्किल होता है।

- हाँ
- नहीं

आप केले नहीं खाते हैं। क्योंकि केले आपकी भूख को तृप्त नहीं कर पाता है।

- हाँ
- नहीं

आप केले नहीं खाते हैं। क्योंकि केले स्थिति के लिए उपयुक्त नहीं होता है।

- हाँ
- नहीं

आप केले नहीं खाते हैं। क्योंकि केले अन्य लोगों के आसपास खाने लायक भोजन नहीं है।

- हाँ
- नहीं

आप केले नहीं खाते हैं। क्योंकि केले कि कीमत बहुत अधिक है।

- हाँ
- नहीं

आप केले नहीं खाते हैं। क्योंकि केले आपको दिखने में अच्छा नहीं लगता है।

- हाँ
- नहीं

आप केले नहीं खाते हैं। क्योंकि केले मे कैलोरी के मात्रा बहुत अधिक होती है।

- हाँ
- नहीं

आप केले नहीं खाते हैं। क्योंकि आप केले नहीं खाना चाहते है।

- हाँ
- नहीं

आप केले नहीं खाते हैं। क्योंकि आप केले खाने से दूसरों के सामने पिछड़ा महसूस करते है।

- हाँ
- नहीं

आप केले नहीं खाते हैं। क्योंकि आपकी केले खाने कि इच्छा नहीं होती है।

- हाँ
- नहीं

आप केले नहीं खाते हैं। क्योंकि अपने हाल ही में केले खाया था और अब आप बार-बार केले नहीं खाना चाहते है।

- हाँ
- नहीं

आप केले नहीं खाते हैं। क्योंकि आपको हर दिन एक ही प्रकार का भोजन खाना पसंद है।

- हाँ
- नहीं

आप केले नहीं खाते हैं। क्योंकि केले खाना आपको दुखी, अकेला और कुंठित महसूस कराता है।

- हाँ
- नहीं

आप केले नहीं खाते हैं। क्योंकि केले जैविक (आर्गेनिक) नहीं है।

- हाँ
- नहीं

Reasons for Sweet food Choices(Full CATA)

क्या आप गुलाब जामुन खाते हैं ।

- हाँ
- नहीं

आप गुलाब जामुन खाते हैं। क्योंकि आप गुलाब जामुन खाने के आदी हैं।

- हाँ
- नहीं

आप गुलाब जामुन खाते हैं। क्योंकि गुलाब जामुन खाना स्वास्थ्य के लिए लाभदायक हैं।

- हाँ
- नहीं

आप गुलाब जामुन खाते हैं। क्योंकि गुलाब जामुन कम समय में बन जाता है।

- हाँ
- नहीं

आप गुलाब जामुन खाते हैं। क्योंकि गुलाब जामुन का स्वाद अच्छा है।

- हाँ
- नहीं

आप गुलाब जामुन खाते हैं। क्योंकि गुलाब जामुन मुझे तृप्त करता है।

- हाँ
- नहीं

आप गुलाब जामुन खाते हैं। क्योंकि गुलाब जामुन प्राकृतिक है।

- हाँ
- नहीं

आप गुलाब जामुन खाते हैं। क्योंकि आप अधिक पैसे खर्च नहीं करना चाहते है।

- हाँ
- नहीं

आप गुलाब जामुन खाते हैं। क्योंकि गुलाब जामुन में कम कैलोरीज होती हैं।

- हाँ
- नहीं

आप गुलाब जामुन खाते हैं। क्योंकि आप निराश रहते हैं।

○ हाँ
○ नहीं

आप गुलाब जामुन खाते हैं। क्योंकि गुलाब जामुन खाना आपको दूसरों के सामने अच्छा दिखाता हैं।

○ हाँ
○ नहीं

आप गुलाब जामुन खाते हैं। क्योंकि गुलाब जामुन नहीं खाना अशिष्ट होता है।

○ हाँ
○ नहीं

आप गुलाब जामुन खाते हैं। क्योंकि गुलाब जामुन कि प्रस्तुति आकर्षक होती है।

○ हाँ
○ नहीं

आप गुलाब जामुन खाते हैं। ताकि आप अन्य लोगों के साथ समय बिता सके।

○ हाँ
○ नहीं

आप गुलाब जामुन खाते हैं। क्योंकि गुलाब जामुन जैविक होता है।

○ हाँ
○ नहीं

आप गुलाब जामुन खाते हैं। क्योंकि आप बचपन से गुलाब जामुन खाते रहे हैं।

○ हाँ

○ नहीं

आप गुलाब जामुन खाते हैं। क्योंकि आप गुलाब जामुन खाने में आनंद महसूस करते हैं।

○ हाँ
○ नहीं

आप गुलाब जामुन खाते हैं। क्योंकि आप भूखे रहते हैं।

○ हाँ
○ नहीं

आप गुलाब जामुन खाते हैं। क्योंकि आप आमतौर पर गुलाब जामुन खाते हैं।

○ हाँ
○ नहीं

आप गुलाब जामुन खाते हैं। क्योंकि आपके भीतर गुलाब जामुन खाने कि भूख रहती है।

○ हाँ
○ नहीं

आप गुलाब जामुन खाते हैं। क्योंकि गुलाब जामुन तैयार करना आसान है।

○ हाँ
○ नहीं

आप गुलाब जामुन खाते हैं। क्योंकि आप उदास रहते है।

○ हाँ
○ नहीं

आप गुलाब जामुन खाते हैं। क्योंकि गुलाब जामुन में वसा कि मात्रा कम होती है।

- हाँ
- नहीं

आप गुलाब जामुन खाते हैं। क्योंकि आप गुलाब जामुन को टीवी या विज्ञापन पर देखते हैं।

- हाँ
- नहीं

आप गुलाब जामुन खाते हैं। क्योंकि गुलाब जामुन सामाजिक समारोहों को और अधिक आरामदायक बनाता है।

- हाँ
- नहीं

आप गुलाब जामुन खाते हैं। क्योंकि आपको गुलाब जामुन खाना पड़ता है।

- हाँ
- नहीं

आप गुलाब जामुन खाते हैं। क्योंकि गुलाब जामुन दूसरों को पसंद है।

- हाँ
- नहीं

आप गुलाब जामुन खाते हैं। क्योंकि आप अकेला महसूस करते है।

- हाँ
- नहीं

आप गुलाब जामुन खाते हैं। क्योंकि आप अपने वजन पर निगरानी रखते है।

- हाँ
- नहीं

आप गुलाब जामुन खाते हैं। क्योंकि गुलाब जामुन में कोई हानिकारक (कीटनाशक, प्रदूषक, एंटीबायोटिक) पदार्थ नहीं होता है।

- हाँ
- नहीं

आप गुलाब जामुन खाते हैं। क्योंकि गुलाब जामुन बनाना आसान है।

- हाँ
- नहीं

आप गुलाब जामुन खाते हैं। क्योंकि मूझे ऊर्जा की जरूरत होती है।

- हाँ
- नहीं

आप गुलाब जामुन खाते हैं। क्योंकि आप गुलाब जामुन से परिचित है।

- हाँ
- नहीं

आप गुलाब जामुन खाते हैं। क्योंकि आपको गुलाब जामुन पसंद है।

- हाँ
- नहीं

आप गुलाब जामुन खाते हैं। क्योंकि गुलाब जामुन खाना सुखदेय है।

- हाँ

○ नहीं

आप गुलाब जामुन अपने आपको पुरस्कृत करने के लिए गुलाब जामुन खाते हैं।

○ हाँ
○ नहीं

आप गुलाब जामुन खाते हैं। क्योंकि गुलाब जामुन खाना आपकी परंपरा का हिस्सा है।

○ हाँ
○ नहीं

आप गुलाब जामुन खाते हैं। क्योंकि यह कुछ स्थितियों से संबंधित है।

○ हाँ
○ नहीं

आप गुलाब जामुन संतुलित आहार पाने के लिए खाते हैं।

○ हाँ
○ नहीं

आप गुलाब जामुन खाते हैं। क्योंकि गुलाब जामुन खाना सामाजिक है।

○ हाँ
○ नहीं

आप गुलाब जामुन खाते हैं। क्योंकि गुलाब जामुन सस्ता है।

○ हाँ
○ नहीं

आप गुलाब जामुन खाते हैं। क्योंकि गुलाब जामुन आपके लिए आकर्षक भोजन है।

- हाँ
- नहीं

दुसरो को निराश करने से बचने के लिए आप गुलाब जामुन खाते हैं।

- हाँ
- नहीं

आप गुलाब जामुन खाते हैं। क्योंकि गुलाब जामुन खाना फैशनेबल है।

- हाँ
- नहीं

आप गुलाब जामुन खाते हैं। क्योंकि गुलाब जामुन सेल पर उपलब्ध हैं।

- हाँ
- नहीं

आप गुलाब जामुन खाते हैं। क्योंकि यह आपके शरीर को आकार में रखता है।

- हाँ
- नहीं

आप गुलाब जामुन खाते हैं। क्योंकि गुलाब जामुन ही एकमात्र विकल्प था।

- हाँ
- नहीं

आप गुलाब जामुन खाते हैं। क्योंकि आप गुलाब जामुन हर दिन खाना चाहते है।

- हाँ
- नहीं

आप गुलाब जामुन नहीं खाते हैं। क्योंकि आपको नहीं लगता कि गुलाब जामुन एक नाश्ता है।

- हाँ
- नहीं

आप गुलाब जामुन नहीं खाते हैं। क्योंकि आपको गुलाब जामुन पसंद नहीं है।

- हाँ
- नहीं

आप गुलाब जामुन नहीं खाते हैं। क्योंकि आप आमतौर पर गुलाब जामुन नहीं खाते है।

- हाँ
- नहीं

आप गुलाब जामुन नहीं खाते हैं। क्योंकि गुलाब जामुन कि खुराक का आकार मेरे लिए उपयुक्त नहीं होता है।

- हाँ
- नहीं

आप गुलाब जामुन नहीं खाते हैं। क्योंकि गुलाब जामुन आपकी भूख मिटाने के लिए पर्याप्त नहीं होता है।

- हाँ
- नहीं

आप गुलाब जामुन नहीं खाते हैं। क्योंकि गुलाब जामुन स्वास्थ्यय के लिए लाभदायक नहीं है।

- हाँ
- नहीं

आप गुलाब जामुन नहीं खाते हैं। क्योंकि गुलाब जामुन बनाना मुश्किल होता है।

- हाँ
- नहीं

आप गुलाब जामुन नहीं खाते हैं। क्योंकि गुलाब जामुन आपकी भूख को तृप्त नहीं कर पाता है।

- हाँ
- नहीं

आप गुलाब जामुन नहीं खाते हैं। क्योंकि गुलाब जामुन स्थिति के लिए उपयुक्त नहीं होता है।

- हाँ
- नहीं

आप गुलाब जामुन नहीं खाते हैं। क्योंकि गुलाब जामुन अन्य लोगों के आसपास खाने लायक भोजन नहीं है।

- हाँ
- नहीं

आप गुलाब जामुन नहीं खाते हैं। क्योंकि गुलाब जामुन कि कीमत बहुत अधिक है।

- हाँ
- नहीं

आप गुलाब जामुन नहीं खाते हैं। क्योंकि गुलाब जामुन आपको दिखने में अच्छा नहीं लगता है।

- हाँ
- नहीं

आप गुलाब जामुन नहीं खाते हैं। क्योंकि गुलाब जामुन मे कैलोरी के मात्रा बहुत अधिक होती है।

- हाँ
- नहीं

आप गुलाब जामुन नहीं खाते हैं। क्योंकि आप गुलाब जामुन नहीं खाना चाहते है।

- हाँ
- नहीं

आप गुलाब जामुन नहीं खाते हैं। क्योंकि आप गुलाब जामुन खाने से दूसरों के सामने पिछड़ा महसूस करते है।

- हाँ
- नहीं

आप गुलाब जामुन नहीं खाते हैं। क्योंकि आपकी गुलाब जामुन खाने कि इच्छा नहीं होती है।

- हाँ
- नहीं

आप गुलाब जामुन नहीं खाते हैं। क्योंकि अपने हाल ही में गुलाब जामुन खाया था और अब आप बार-बार गुलाब जामुन नहीं खाना चाहते हैं।

- हाँ
- नहीं

आप गुलाब जामुन नहीं खाते हैं। क्योंकि आपको हर दिन एक ही प्रकार का भोजन खाना पसंद है।

- हाँ
- नहीं

आप गुलाब जामुन नहीं खाते हैं। क्योंकि गुलाब जामुन खाना आपको दुखी, अकेला और कुंठित महसूस कराता है।

- हाँ
- नहीं

आप गुलाब जामुन नहीं खाते हैं। क्योंकि गुलाब जामुन जैविक (आर्गेनिक) नहीं है।

- हाँ
- नहीं

Reasons for Starchy food choices(RATA)

क्या आप चावल खाते हैं।

- हाँ
- नहीं

किन कारणों के लिए आप चावल खातें हैं। (सभी विकल्प चुने)

- क्योंकि चावल में वसा कि मात्रा कम होती है।
- क्योंकि मूझे चावल खाना पड़ता है।
- क्योंकि चावल सामाजिक समारोहों को अधिक आरामदायक बनाता है।
- क्योंकि मैं बचपन से चावल खाता रहा हूँ ।
- क्योंकि मैं अपने वजन पर निगरानी रखता हूँ।
- क्योंकि चावल खाना मुझे तृप्त करता है।
- क्योंकि चावल मेरे लिए आकर्षक भोजन है।
- क्योंकि चावल सबसे सुविधाजनक है।
- क्योंकि चावल में कम कैलोरीज होती है।
- क्योंकि चावल कुछ स्थितियों से संबंधित है।
- क्योंकि चावल खाना सुखदेय है।
- क्योंकि चावल सस्ता है।
- क्योंकि मेरे भीतर चावल खाने कि भूख रहती है।

- ☐ क्योंकि चावल दूसरों को पसंद है।
- ☐ क्योंकि मैं निराश रहता हूँ।
- ☐ क्योंकि चावल का स्वाद अच्छा है।
- ☐ क्योंकि मैं उदास रहता हूँ।
- ☐ क्योंकि चावल में कोई हानिकारक (कीटनाशक, प्रदूषक, एंटीबायोटिक) पदार्थ नहीं होता है।
- ☐ क्योंकि मैं आमतौर पर चावल खाता हूँ ।
- ☐ क्योंकि चावल सस्ता है।
- ☐ क्योंकि मुझे चावल पसंद है।
- ☐ क्योंकि चावल तैयार करना आसान है।
- ☐ क्योंकि चावल खाना मूझे दूसरों के सामने अच्छा दिखाता है।
- ☐ क्योंकि मैं चावल से परिचित हूँ।
- ☐ क्योंकि चावल खाना फैशनेबल है।
- ☐ ताकि मैं अन्य लोगों के साथ समय बिता सकूं।
- ☐ क्योंकि मैं चावल खाने में आनंद महसूस करता हूँ ।
- ☐ क्योंकि चावल प्राकृतिक है।
- ☐ दुसरो को निराश करने से बचने के लिए।
- ☐ क्योंकि चावल जैविक होता है।
- ☐ क्योंकि मैं चावल हर दिन खाना चाहता हूँ।
- ☐ क्योंकि मैं चावल खाने का आदी हूँ।
- ☐ क्योंकि मैं चावल को टीवी या विज्ञापन पर देखता हूँ।
- ☐ क्योंकि चावल खाना सामाजिक है।
- ☐ क्योंकि चावल कि प्रस्तुति आकर्षक होती है।
- ☐ क्योंकि मूझे ऊर्जा की जरूरत होती है।
- ☐ क्योंकि चावल ही एकमात्र विकल्प था।
- ☐ क्योंकि मैं भूखा रहता हूँ ।
- ☐ क्योंकि चावल खाना स्वास्थ्य के लिए लाभदायक है।
- ☐ चावल खाना मेरी परंपरा का हिस्सा है।
- ☐ क्योंकि चावल या चावल से सम्बंदित भोज कम समय में बन जाता है।
- ☐ मैं अपने आपको पुरस्कृत करने के लिए चावल खाता हूँ।

- ☐ क्योंकि चावल विक्रय बिक्री पर होता है।
- ☐ क्योंकि मैं अकेला महसूस करता हूँ।
- ☐ क्योंकि चावल नहीं खाना अशिष्ट होता है।
- ☐ संतुलित आहार बनाए रखने के लिए।
- ☐ क्योंकि चावल मेरे शरीर को आकार में रखता है।

आपके द्वारा चयनित "क्योंकि मैं चावल खाने का आदी हूँ।"

आपके लिए यह कथन कितना महत्वपूर्ण है।

- ○ अत्यंत महत्वपूर्ण
- ○ बहुत महत्वपूर्ण
- ○ मामूली रूप से महत्वपूर्ण
- ○ थोड़ा महत्वपूर्ण
- ○ कतई महत्वपूर्ण नहीं

आपके द्वारा चयनित "क्योंकि चावल खाना स्वास्थ्य के लिए लाभदायक है।"

आपके लिए यह कथन कितना महत्वपूर्ण है।

- ○ अत्यंत महत्वपूर्ण
- ○ बहुत महत्वपूर्ण
- ○ मामूली रूप से महत्वपूर्ण
- ○ थोड़ा महत्वपूर्ण
- ○ कतई महत्वपूर्ण नहीं

आपके द्वारा चयनित "क्योंकि चावल या चावल से सम्बंदित भोज कम समय में बन जाता है।"

आपके लिए यह कथन कितना महत्वपूर्ण है।

- अत्यंत महत्वपूर्ण
- बहुत महत्वपूर्ण
- मामूली रूप से महत्वपूर्ण
- थोड़ा महत्वपूर्ण
- कतई महत्वपूर्ण नहीं

आपके द्वारा चयनित "क्योंकि चावल का स्वाद अच्छा है।"

आपके लिए यह कथन कितना महत्वपूर्ण है।

- अत्यंत महत्वपूर्ण
- बहुत महत्वपूर्ण
- मामूली रूप से महत्वपूर्ण
- थोड़ा महत्वपूर्ण
- कतई महत्वपूर्ण नहीं

आपके द्वारा चयनित "क्योंकि चावल खाना मुझे तृप्त करता है।"

आपके लिए यह कथन कितना महत्वपूर्ण है।

- अत्यंत महत्वपूर्ण
- बहुत महत्वपूर्ण
- मामूली रूप से महत्वपूर्ण
- थोड़ा महत्वपूर्ण
- कतई महत्वपूर्ण नहीं

आपके द्वारा चयनित "क्योंकि चावल प्राकृतिक है।"

आपके लिए यह कथन कितना महत्वपूर्ण है।

- अत्यंत महत्वपूर्ण
- बहुत महत्वपूर्ण
- मामूली रूप से महत्वपूर्ण
- थोड़ा महत्वपूर्ण
- कतई महत्वपूर्ण नहीं

आपके द्वारा चयनित "क्योंकि चावल सस्ता है।"

आपके लिए यह कथन कितना महत्वपूर्ण है।

- अत्यंत महत्वपूर्ण
- बहुत महत्वपूर्ण
- मामूली रूप से महत्वपूर्ण
- थोड़ा महत्वपूर्ण
- कतई महत्वपूर्ण नहीं

आपके द्वारा चयनित "क्योंकि चावल में कम कैलोरीज होती है।"

आपके लिए यह कथन कितना महत्वपूर्ण है।

- अत्यंत महत्वपूर्ण
- बहुत महत्वपूर्ण
- मामूली रूप से महत्वपूर्ण
- थोड़ा महत्वपूर्ण

- कतई महत्वपूर्ण नहीं

आपके द्वारा चयनित "क्योंकि मैं निराश रहता हूँ।"

आपके लिए यह कथन कितना महत्वपूर्ण है।

- अत्यंत महत्वपूर्ण
- बहुत महत्वपूर्ण
- मामूली रूप से महत्वपूर्ण
- थोड़ा महत्वपूर्ण
- कतई महत्वपूर्ण नहीं

आपके द्वारा चयनित "क्योंकि चावल खाना मूझे दूसरों के सामने अच्छा दिखाता है।"

आपके लिए यह कथन कितना महत्वपूर्ण है।

- अत्यंत महत्वपूर्ण
- बहुत महत्वपूर्ण
- मामूली रूप से महत्वपूर्ण
- थोड़ा महत्वपूर्ण
- कतई महत्वपूर्ण नहीं

आपके द्वारा चयनित "क्योंकि चावल नहीं खाना अशिष्ट होता है।"

आपके लिए यह कथन कितना महत्वपूर्ण है।

- अत्यंत महत्वपूर्ण

- बहुत महत्वपूर्ण
- मामूली रूप से महत्वपूर्ण
- थोड़ा महत्वपूर्ण
- कतई महत्वपूर्ण नहीं

आपके द्वारा चयनित "क्योंकि चावल कि प्रस्तुति आकर्षक होती है।"

आपके लिए यह कथन कितना महत्वपूर्ण है।

- अत्यंत महत्वपूर्ण
- बहुत महत्वपूर्ण
- मामूली रूप से महत्वपूर्ण
- थोड़ा महत्वपूर्ण
- कतई महत्वपूर्ण नहीं

आपके द्वारा चयनित "ताकि मैं अन्य लोगों के साथ समय बिता सकूं।"

आपके लिए यह कथन कितना महत्वपूर्ण है।

- अत्यंत महत्वपूर्ण
- बहुत महत्वपूर्ण
- मामूली रूप से महत्वपूर्ण
- थोड़ा महत्वपूर्ण
- कतई महत्वपूर्ण नहीं

आपके द्वारा चयनित "क्योंकि चावल जैविक होता है।"

आपके लिए यह कथन कितना महत्वपूर्ण है।

- अत्यंत महत्वपूर्ण
- बहुत महत्वपूर्ण
- मामूली रूप से महत्वपूर्ण
- थोड़ा महत्वपूर्ण
- कतई महत्वपूर्ण नहीं

आपके द्वारा चयनित "क्योंकि मैं बचपन से चावल खाता रहा हूँ ।"

आपके लिए यह कथन कितना महत्वपूर्ण है।

- अत्यंत महत्वपूर्ण
- बहुत महत्वपूर्ण
- मामूली रूप से महत्वपूर्ण
- थोड़ा महत्वपूर्ण
- कतई महत्वपूर्ण नहीं

आपके द्वारा चयनित "क्योंकि मैं चावल खाने में आनंद महसूस करता हूँ ।"

आपके लिए यह कथन कितना महत्वपूर्ण है।

- अत्यंत महत्वपूर्ण
- बहुत महत्वपूर्ण
- मामूली रूप से महत्वपूर्ण
- थोड़ा महत्वपूर्ण
- कतई महत्वपूर्ण नहीं

आपके द्वारा चयनित "क्योंकि मैं भूखा रहता हूँ ।"

आपके लिए यह कथन कितना महत्वपूर्ण है।

- अत्यंत महत्वपूर्ण
- बहुत महत्वपूर्ण
- मामूली रूप से महत्वपूर्ण
- थोड़ा महत्वपूर्ण
- कतई महत्वपूर्ण नहीं

आपके द्वारा चयनित "क्योंकि मैं आमतौर पर चावल खाता हूँ ।"

आपके लिए यह कथन कितना महत्वपूर्ण है।

- अत्यंत महत्वपूर्ण
- बहुत महत्वपूर्ण
- मामूली रूप से महत्वपूर्ण
- थोड़ा महत्वपूर्ण
- कतई महत्वपूर्ण नहीं

आपके द्वारा चयनित "क्योंकि मेरे भीतर चावल खाने कि भूख रहती है।"

आपके लिए यह कथन कितना महत्वपूर्ण है।

- अत्यंत महत्वपूर्ण
- बहुत महत्वपूर्ण
- मामूली रूप से महत्वपूर्ण
- थोड़ा महत्वपूर्ण

- कतई महत्वपूर्ण नहीं

आपके द्वारा चयनित "क्योंकि चावल तैयार करना आसान है।"

आपके लिए यह कथन कितना महत्वपूर्ण है।

- अत्यंत महत्वपूर्ण
- बहुत महत्वपूर्ण
- मामूली रूप से महत्वपूर्ण
- थोड़ा महत्वपूर्ण
- कतई महत्वपूर्ण नहीं

आपके द्वारा चयनित "क्योंकि मैं उदास रहता हूँ।"

आपके लिए यह कथन कितना महत्वपूर्ण है।

- अत्यंत महत्वपूर्ण
- बहुत महत्वपूर्ण
- मामूली रूप से महत्वपूर्ण
- थोड़ा महत्वपूर्ण
- कतई महत्वपूर्ण नहीं

आपके द्वारा चयनित "क्योंकि चावल में वसा कि मात्रा कम होती है।"

आपके लिए यह कथन कितना महत्वपूर्ण है।

- अत्यंत महत्वपूर्ण

- बहुत महत्वपूर्ण
- मामूली रूप से महत्वपूर्ण
- थोड़ा महत्वपूर्ण
- कतई महत्वपूर्ण नहीं

आपके द्वारा चयनित "क्योंकि मैं चावल को टीवी या विज्ञापन पर देखता हूँ।"

आपके लिए यह कथन कितना महत्वपूर्ण है।

- अत्यंत महत्वपूर्ण
- बहुत महत्वपूर्ण
- मामूली रूप से महत्वपूर्ण
- थोड़ा महत्वपूर्ण
- कतई महत्वपूर्ण नहीं

आपके द्वारा चयनित "क्योंकि चावल सामाजिक समारोहों को अधिक आरामदायक बनाता है।"

आपके लिए यह कथन कितना महत्वपूर्ण है।

- अत्यंत महत्वपूर्ण
- बहुत महत्वपूर्ण
- मामूली रूप से महत्वपूर्ण
- थोड़ा महत्वपूर्ण
- कतई महत्वपूर्ण नहीं

आपके द्वारा चयनित "क्योंकि मूझे चावल खाना पड़ता है।"

आपके लिए यह कथन कितना महत्वपूर्ण है।

- अत्यंत महत्वपूर्ण
- बहुत महत्वपूर्ण
- मामूली रूप से महत्वपूर्ण
- थोड़ा महत्वपूर्ण
- कतई महत्वपूर्ण नहीं

आपके द्वारा चयनित "क्योंकि चावल दूसरों को पसंद है।"

आपके लिए यह कथन कितना महत्वपूर्ण है।

- अत्यंत महत्वपूर्ण
- बहुत महत्वपूर्ण
- मामूली रूप से महत्वपूर्ण
- थोड़ा महत्वपूर्ण
- कतई महत्वपूर्ण नहीं

आपके द्वारा चयनित "क्योंकि मैं अकेला महसूस करता हूँ।"

आपके लिए यह कथन कितना महत्वपूर्ण है।

- अत्यंत महत्वपूर्ण
- बहुत महत्वपूर्ण
- मामूली रूप से महत्वपूर्ण
- थोड़ा महत्वपूर्ण
- कतई महत्वपूर्ण नहीं

आपके द्वारा चयनित "क्योंकि मैं अपने वजन पर निगरानी रखता हूँ।"

आपके लिए यह कथन कितना महत्वपूर्ण है।

- अत्यंत महत्वपूर्ण
- बहुत महत्वपूर्ण
- मामूली रूप से महत्वपूर्ण
- थोड़ा महत्वपूर्ण
- कतई महत्वपूर्ण नहीं

आपके द्वारा चयनित "क्योंकि चावल में कोई हानिकारक (कीटनाशक, प्रदूषक, एंटीबायोटिक) पदार्थ नहीं होता है।"

आपके लिए यह कथन कितना महत्वपूर्ण है।

- अत्यंत महत्वपूर्ण
- बहुत महत्वपूर्ण
- मामूली रूप से महत्वपूर्ण
- थोड़ा महत्वपूर्ण
- कतई महत्वपूर्ण नहीं

आपके द्वारा चयनित "क्योंकि चावल सबसे सुविधाजनक है।"

आपके लिए यह कथन कितना महत्वपूर्ण है।

- अत्यंत महत्वपूर्ण
- बहुत महत्वपूर्ण
- मामूली रूप से महत्वपूर्ण

- थोड़ा महत्वपूर्ण
- कतई महत्वपूर्ण नहीं

आपके द्वारा चयनित "क्योंकि मूझे ऊर्जा की जरूरत होती है।"

आपके लिए यह कथन कितना महत्वपूर्ण है।

- अत्यंत महत्वपूर्ण
- बहुत महत्वपूर्ण
- मामूली रूप से महत्वपूर्ण
- थोड़ा महत्वपूर्ण
- कतई महत्वपूर्ण नहीं

आपके द्वारा चयनित "क्योंकि मैं चावल से परिचित हूँ।"

आपके लिए यह कथन कितना महत्वपूर्ण है।

- अत्यंत महत्वपूर्ण
- बहुत महत्वपूर्ण
- मामूली रूप से महत्वपूर्ण
- थोड़ा महत्वपूर्ण
- कतई महत्वपूर्ण नहीं

आपके द्वारा चयनित "क्योंकि मुझे चावल पसंद है।"

आपके लिए यह कथन कितना महत्वपूर्ण है।

- अत्यंत महत्वपूर्ण
- बहुत महत्वपूर्ण
- मामूली रूप से महत्वपूर्ण
- थोड़ा महत्वपूर्ण
- कतई महत्वपूर्ण नहीं

आपके द्वारा चयनित "क्योंकि चावल खाना सुखदेय है।"

आपके लिए यह कथन कितना महत्वपूर्ण है।

- अत्यंत महत्वपूर्ण
- बहुत महत्वपूर्ण
- मामूली रूप से महत्वपूर्ण
- थोड़ा महत्वपूर्ण
- कतई महत्वपूर्ण नहीं

आपके द्वारा चयनित "मैं अपने आपको पुरस्कृत करने के लिए चावल खाता हूँ।"

आपके लिए यह कथन कितना महत्वपूर्ण है।

- अत्यंत महत्वपूर्ण
- बहुत महत्वपूर्ण
- मामूली रूप से महत्वपूर्ण
- थोड़ा महत्वपूर्ण
- कतई महत्वपूर्ण नहीं

आपके द्वारा चयनित "चावल खाना मेरी परंपरा का हिस्सा है।"

आपके लिए यह कथन कितना महत्वपूर्ण है।

- अत्यंत महत्वपूर्ण
- बहुत महत्वपूर्ण
- मामूली रूप से महत्वपूर्ण
- थोड़ा महत्वपूर्ण
- कतई महत्वपूर्ण नहीं

आपके द्वारा चयनित "क्योंकि चावल कुछ स्थितियों से संबंधित है।"

आपके लिए यह कथन कितना महत्वपूर्ण है।

- अत्यंत महत्वपूर्ण
- बहुत महत्वपूर्ण
- मामूली रूप से महत्वपूर्ण
- थोड़ा महत्वपूर्ण
- कतई महत्वपूर्ण नहीं

आपके द्वारा चयनित "संतुलित आहार बनाए रखने के लिए।"

आपके लिए यह कथन कितना महत्वपूर्ण है।

- अत्यंत महत्वपूर्ण
- बहुत महत्वपूर्ण
- मामूली रूप से महत्वपूर्ण
- थोड़ा महत्वपूर्ण
- कतई महत्वपूर्ण नहीं

आपके द्वारा चयनित "क्योंकि चावल खाना सामाजिक है।"

आपके लिए यह कथन कितना महत्वपूर्ण है।

- अत्यंत महत्वपूर्ण
- बहुत महत्वपूर्ण
- मामूली रूप से महत्वपूर्ण
- थोड़ा महत्वपूर्ण
- कतई महत्वपूर्ण नहीं

आपके द्वारा चयनित "क्योंकि चावल सस्ता है।"

आपके लिए यह कथन कितना महत्वपूर्ण है।

- अत्यंत महत्वपूर्ण
- बहुत महत्वपूर्ण
- मामूली रूप से महत्वपूर्ण
- थोड़ा महत्वपूर्ण
- कतई महत्वपूर्ण नहीं

आपके द्वारा चयनित "क्योंकि चावल मेरे लिए आकर्षक भोजन है।"

आपके लिए यह कथन कितना महत्वपूर्ण है।

- अत्यंत महत्वपूर्ण
- बहुत महत्वपूर्ण
- मामूली रूप से महत्वपूर्ण
- थोड़ा महत्वपूर्ण

- कतई महत्वपूर्ण नहीं

आपके द्वारा चयनित "दुसरो को निराश करने से बचने के लिए।"

आपके लिए यह कथन कितना महत्वपूर्ण है।

- अत्यंत महत्वपूर्ण
- बहुत महत्वपूर्ण
- मामूली रूप से महत्वपूर्ण
- थोड़ा महत्वपूर्ण
- कतई महत्वपूर्ण नहीं

आपके द्वारा चयनित "क्योंकि चावल खाना फैशनेबल है।"

आपके लिए यह कथन कितना महत्वपूर्ण है।

- अत्यंत महत्वपूर्ण
- बहुत महत्वपूर्ण
- मामूली रूप से महत्वपूर्ण
- थोड़ा महत्वपूर्ण
- कतई महत्वपूर्ण नहीं

आपके द्वारा चयनित "क्योंकि चावल विक्रय बिक्री पर होता है।"

आपके लिए यह कथन कितना महत्वपूर्ण है।

- अत्यंत महत्वपूर्ण

- बहुत महत्वपूर्ण
- मामूली रूप से महत्वपूर्ण
- थोड़ा महत्वपूर्ण
- कतई महत्वपूर्ण नहीं

आपके द्वारा चयनित "क्योंकि चावल मेरे शरीर को आकार में रखता है।"

आपके लिए यह कथन कितना महत्वपूर्ण है।

- अत्यंत महत्वपूर्ण
- बहुत महत्वपूर्ण
- मामूली रूप से महत्वपूर्ण
- थोड़ा महत्वपूर्ण
- कतई महत्वपूर्ण नहीं

आपके द्वारा चयनित "क्योंकि चावल ही एकमात्र विकल्प था।"

आपके लिए यह कथन कितना महत्वपूर्ण है।

- अत्यंत महत्वपूर्ण
- बहुत महत्वपूर्ण
- मामूली रूप से महत्वपूर्ण
- थोड़ा महत्वपूर्ण
- कतई महत्वपूर्ण नहीं

आपके द्वारा चयनित "क्योंकि मैं चावल हर दिन खाना चाहता हूँ।"

आपके लिए यह कथन कितना महत्वपूर्ण है।

- अत्यंत महत्वपूर्ण
- बहुत महत्वपूर्ण
- मामूली रूप से महत्वपूर्ण
- थोड़ा महत्वपूर्ण
- कतई महत्वपूर्ण नहीं

कृपया चावल नहीं खाने के सभी कारण बताएं। (सभी विकल्प चुने)

- चावल खाना मूझे दुखी, अकेला और कुंठित महसूस कराता है।
- चावल मेरी भूख मिटाने के लिए पर्याप्त नहीं होता है।
- चावल मूझे दिखने में अच्छा नहीं लगता है।
- मुझे चावल पसंद नहीं है।
- मैं चावल नहीं खाना चाहता हूँ।
- क्योंकि चावल जैविक (आर्गेनिक) नहीं है।
- मेने हाल ही में चावल खाया था और अब मैं बार-बार चावल नहीं खाना चाहता हूँ।
- मुझे नहीं लगता कि चावल एक नाश्ता है।
- चावल बनाना मुश्किल होता है।
- चावल स्थिति के लिए उपयुक्त नहीं था।
- चावल अन्य लोगों के आसपास खाने लायक भोजन नहीं है।
- चावल स्वास्थ्यय के लिए लाभदायक नहीं है।
- चावल मेरी भूख को तृप्त नहीं कर पाता है।
- चावल कि कीमत बहुत अधिक होती है।
- मैं चावल खाने से दूसरों के सामने पिछड़ा महसूस करता हूँ।
- मैं कभी चावल नहीं खाऊंगा। क्योंकि मुझे हर दिन एक ही प्रकार का भोजन खाना पसंद है।
- मैं आमतौर पर चावल नहीं खाता हूँ।
- चावल मे कैलोरी की मात्रा बहुत अधिक होती है।
- मेरी चावल खाने कि इच्छा नहीं होती है।
- चावल कि खुराक का आकार मेरे लिए उपयुक्त नहीं होता है।

आपके द्वारा चयनित "मुझे नहीं लगता कि चावल एक नाश्ता है।"

आपके लिए यह कथन कितना महत्वपूर्ण है।

- अत्यंत महत्वपूर्ण
- बहुत महत्वपूर्ण
- मामूली रूप से महत्वपूर्ण
- थोड़ा महत्वपूर्ण
- कतई महत्वपूर्ण नहीं

आपके द्वारा चयनित "मुझे चावल पसंद नहीं है।"

आपके लिए यह कथन कितना महत्वपूर्ण है।

- अत्यंत महत्वपूर्ण
- बहुत महत्वपूर्ण
- मामूली रूप से महत्वपूर्ण
- थोड़ा महत्वपूर्ण
- कतई महत्वपूर्ण नहीं

आपके द्वारा चयनित "मैं आमतौर पर चावल नहीं खाता हूँ।"

आपके लिए यह कथन कितना महत्वपूर्ण है।

- अत्यंत महत्वपूर्ण
- बहुत महत्वपूर्ण
- मामूली रूप से महत्वपूर्ण

- थोड़ा महत्वपूर्ण
- कतई महत्वपूर्ण नहीं

आपके द्वारा चयनित "चावल कि खुराक का आकार मेरे लिए उपयुक्त नहीं होता है।"

आपके लिए यह कथन कितना महत्वपूर्ण है।

- अत्यंत महत्वपूर्ण
- बहुत महत्वपूर्ण
- मामूली रूप से महत्वपूर्ण
- थोड़ा महत्वपूर्ण
- कतई महत्वपूर्ण नहीं

आपके द्वारा चयनित "चावल मेरी भूख मिटाने के लिए पर्याप्त नहीं होता है।"

आपके लिए यह कथन कितना महत्वपूर्ण है।

- अत्यंत महत्वपूर्ण
- बहुत महत्वपूर्ण
- मामूली रूप से महत्वपूर्ण
- थोड़ा महत्वपूर्ण
- कतई महत्वपूर्ण नहीं

आपके द्वारा चयनित "चावल स्वास्थ्यय के लिए लाभदायक नहीं है।"

आपके लिए यह कथन कितना महत्वपूर्ण है।

- अत्यंत महत्वपूर्ण
- बहुत महत्वपूर्ण
- मामूली रूप से महत्वपूर्ण
- थोड़ा महत्वपूर्ण
- कतई महत्वपूर्ण नहीं

आपके द्वारा चयनित "चावल बनाना मुश्किल होता है।"

आपके लिए यह कथन कितना महत्वपूर्ण है।

- अत्यंत महत्वपूर्ण
- बहुत महत्वपूर्ण
- मामूली रूप से महत्वपूर्ण
- थोड़ा महत्वपूर्ण
- कतई महत्वपूर्ण नहीं

आपके द्वारा चयनित "चावल मेरी भूख को तृप्त नहीं कर पाता है।"

आपके लिए यह कथन कितना महत्वपूर्ण है।

- अत्यंत महत्वपूर्ण
- बहुत महत्वपूर्ण
- मामूली रूप से महत्वपूर्ण
- थोड़ा महत्वपूर्ण
- कतई महत्वपूर्ण नहीं

आपके द्वारा चयनित "चावल स्थिति के लिए उपयुक्त नहीं था।"

आपके लिए यह कथन कितना महत्वपूर्ण है।

- अत्यंत महत्वपूर्ण
- बहुत महत्वपूर्ण
- मामूली रूप से महत्वपूर्ण
- थोड़ा महत्वपूर्ण
- कतई महत्वपूर्ण नहीं

आपके द्वारा चयनित "चावल अन्य लोगों के आसपास खाने लायक भोजन नहीं है।"

आपके लिए यह कथन कितना महत्वपूर्ण है।

- अत्यंत महत्वपूर्ण
- बहुत महत्वपूर्ण
- मामूली रूप से महत्वपूर्ण
- थोड़ा महत्वपूर्ण
- कतई महत्वपूर्ण नहीं

आपके द्वारा चयनित "चावल कि कीमत बहुत अधिक होती है।"

आपके लिए यह कथन कितना महत्वपूर्ण है।

- अत्यंत महत्वपूर्ण
- बहुत महत्वपूर्ण
- मामूली रूप से महत्वपूर्ण
- थोड़ा महत्वपूर्ण
- कतई महत्वपूर्ण नहीं

आपके द्वारा चयनित "चावल मूझे दिखने में अच्छा नहीं लगता है।"

आपके लिए यह कथन कितना महत्वपूर्ण है।

- अत्यंत महत्वपूर्ण
- बहुत महत्वपूर्ण
- मामूली रूप से महत्वपूर्ण
- थोड़ा महत्वपूर्ण
- कतई महत्वपूर्ण नहीं

आपके द्वारा चयनित "चावल मे कैलोरी की मात्रा बहुत अधिक होती है।"

आपके लिए यह कथन कितना महत्वपूर्ण है।

- अत्यंत महत्वपूर्ण
- बहुत महत्वपूर्ण
- मामूली रूप से महत्वपूर्ण
- थोड़ा महत्वपूर्ण
- कतई महत्वपूर्ण नहीं

आपके द्वारा चयनित "मैं चावल नहीं खाना चाहता हूँ।"

आपके लिए यह कथन कितना महत्वपूर्ण है।

- अत्यंत महत्वपूर्ण
- बहुत महत्वपूर्ण
- मामूली रूप से महत्वपूर्ण
- थोड़ा महत्वपूर्ण

- कतई महत्वपूर्ण नहीं

आपके द्वारा चयनित "मैं चावल खाने से दूसरों के सामने पिछड़ा महसूस करता हूँ।"

आपके लिए यह कथन कितना महत्वपूर्ण है।

- अत्यंत महत्वपूर्ण
- बहुत महत्वपूर्ण
- मामूली रूप से महत्वपूर्ण
- थोड़ा महत्वपूर्ण
- कतई महत्वपूर्ण नहीं

आपके द्वारा चयनित "मेरी चावल खाने कि इच्छा नहीं होती है।"

आपके लिए यह कथन कितना महत्वपूर्ण है।

- अत्यंत महत्वपूर्ण
- बहुत महत्वपूर्ण
- मामूली रूप से महत्वपूर्ण
- थोड़ा महत्वपूर्ण
- कतई महत्वपूर्ण नहीं

आपके द्वारा चयनित "मेने हाल ही में चावल खाया था और अब मैं बार-बार चावल नहीं खाना चाहता हूँ।"

आपके लिए यह कथन कितना महत्वपूर्ण है।

- अत्यंत महत्वपूर्ण
- बहुत महत्वपूर्ण
- मामूली रूप से महत्वपूर्ण
- थोड़ा महत्वपूर्ण
- कतई महत्वपूर्ण नहीं

आपके द्वारा चयनित "मैं कभी चावल नहीं खाऊंगा। क्योंकि मुझे हर दिन एक ही प्रकार का भोजन खाना पसंद है।"

आपके लिए यह कथन कितना महत्वपूर्ण है।

- अत्यंत महत्वपूर्ण
- बहुत महत्वपूर्ण
- मामूली रूप से महत्वपूर्ण
- थोड़ा महत्वपूर्ण
- कतई महत्वपूर्ण नहीं

आपके द्वारा चयनित "चावल खाना मूझे दुखी, अकेला और कुंठित महसूस कराता है।"

आपके लिए यह कथन कितना महत्वपूर्ण है।

- अत्यंत महत्वपूर्ण
- बहुत महत्वपूर्ण
- मामूली रूप से महत्वपूर्ण
- थोड़ा महत्वपूर्ण
- कतई महत्वपूर्ण नहीं

आपके द्वारा चयनित "क्योंकि चावल जैविक (आर्गेनिक) नहीं है।"

आपके लिए यह कथन कितना महत्वपूर्ण है।

- अत्यंत महत्वपूर्ण
- बहुत महत्वपूर्ण
- मामूली रूप से महत्वपूर्ण
- थोड़ा महत्वपूर्ण
- कतई महत्वपूर्ण नहीं

Reasons for Protein foods Choices(RATA)

क्या आप दूध खाते हैं।

- हाँ
- नहीं

किन कारणों के लिए आप दूध खातें हैं। (सभी विकल्प चुने)

- मैं अपने आपको पुरस्कृत करने के लिए दूध खाता हूँ।
- दुसरो को निराश करने से बचने के लिए।
- क्योंकि मैं भूखा रहता था।
- क्योंकि दूध खाना मुझे तृप्त करता है।
- क्योंकि दूध बनाना सुविधाजनक है।
- क्योंकि दूध कि प्रस्तुति आकर्षक होती है।
- क्योंकि मैं उदास रहता हूँ।
- क्योंकि दूध खाना सुखदेय है।
- क्योंकि मैं बचपन से दूध खाता रहा हूँ।
- क्योंकि मुझे दूध पसंद है।
- ताकि मैं अन्य लोगों के साथ समय बिता सकूं।
- दूध खाना मेरी परंपरा का हिस्सा है।

- ☐ क्योंकि मैं दूध खाने का आदी हूँ।
- ☐ क्योंकि दूध नहीं खाना अशिष्ट होता है।
- ☐ क्योंकि दूध ही एकमात्र विकल्प है।
- ☐ क्योंकि दूध बनाना आसान है।
- ☐ क्योंकि मैं दूध को टीवी या विज्ञापन पर देखता हूँ।
- ☐ क्योंकि मैं दूध हर दिन खाना चाहता हूँ।
- ☐ संतुलित आहार बनाए रखने के लिए।
- ☐ क्योंकि दूध में कम कैलोरीज होती है।
- ☐ क्योंकि मूझे दूध खाना पड़ता है।
- ☐ क्योंकि दूध खाना मूझे दूसरों के सामने अच्छा दिखाता है।
- ☐ क्योंकि दूध सामाजिक समारोहों को और अधिक आरामदायक बनाता है।
- ☐ क्योंकि दूध में वसा कि मात्रा कम होती है।
- ☐ क्योंकि दूध का स्वाद अच्छा है।
- ☐ क्योंकि मैं दूध खाने में आनंद महसूस करता हूँ।
- ☐ क्योंकि दूध मेरे लिए आकर्षक भोजन है।
- ☐ क्योंकि दूध खाना फैशनेबल है।
- ☐ क्योंकि दूध प्राकृतिक है।
- ☐ क्योंकि दूध विक्रय बिक्री पर होता है।
- ☐ क्योंकि मैं अकेला महसूस करता हूँ।
- ☐ क्योंकि दूध या दूध से सम्बंदित भोज कम समय में बन जाता है।
- ☐ क्योंकि मैं आमतौर पर दूध खाता हूँ।
- ☐ क्योंकि मैं दूध से परिचित हूँ।
- ☐ क्योंकि दूध जैविक होता है।
- ☐ क्योंकि मैं निराश रहता हूँ।
- ☐ क्योंकि मूझे ऊर्जा की जरूरत होती है।
- ☐ क्योंकि मैं अपने वजन पर निगरानी रखता हूँ।
- ☐ क्योंकि दूध सस्ता है।
- ☐ क्योंकि दूध में कोई हानिकारक (कीटनाशक, प्रदूषक, एंटीबायोटिक) पदार्थ नहीं होता है।
- ☐ क्योंकि दूध खाना सामाजिक है।

- ☐ क्योंकि दूध दूसरों को पसंद है।
- ☐ क्योंकि दूध खाना स्वास्थ्य के लिए लाभदायक है।
- ☐ क्योंकि दूध मेरे शरीर को आकार में रखता है।
- ☐ क्योंकि दूध सस्ता है।
- ☐ क्योंकि दूध कुछ स्थितियों से संबंधित है।
- ☐ क्योंकि मेरे भीतर दूध खाने कि भूख रहती है।

आपके द्वारा चयनित "क्योंकि मैं दूध खाने का आदी हूँ।"

आपके लिए यह कथन कितना महत्वपूर्ण है।

- ○ अत्यंत महत्वपूर्ण
- ○ बहुत महत्वपूर्ण
- ○ मामूली रूप से महत्वपूर्ण
- ○ थोड़ा महत्वपूर्ण
- ○ कतई महत्वपूर्ण नहीं

आपके द्वारा चयनित "क्योंकि दूध खाना स्वास्थ्य के लिए लाभदायक है।"

आपके लिए यह कथन कितना महत्वपूर्ण है।

- ○ अत्यंत महत्वपूर्ण
- ○ बहुत महत्वपूर्ण
- ○ मामूली रूप से महत्वपूर्ण
- ○ थोड़ा महत्वपूर्ण
- ○ कतई महत्वपूर्ण नहीं

आपके द्वारा चयनित "क्योंकि दूध या दूध से सम्बंदित भोज कम समय में बन जाता है।"

आपके लिए यह कथन कितना महत्वपूर्ण है।

- अत्यंत महत्वपूर्ण
- बहुत महत्वपूर्ण
- मामूली रूप से महत्वपूर्ण
- थोड़ा महत्वपूर्ण
- कतई महत्वपूर्ण नहीं

आपके द्वारा चयनित "क्योंकि दूध का स्वाद अच्छा है।"

आपके लिए यह कथन कितना महत्वपूर्ण है।

- अत्यंत महत्वपूर्ण
- बहुत महत्वपूर्ण
- मामूली रूप से महत्वपूर्ण
- थोड़ा महत्वपूर्ण
- कतई महत्वपूर्ण नहीं

आपके द्वारा चयनित "क्योंकि दूध खाना मुझे तृप्त करता है।"

आपके लिए यह कथन कितना महत्वपूर्ण है।

- अत्यंत महत्वपूर्ण
- बहुत महत्वपूर्ण
- मामूली रूप से महत्वपूर्ण
- थोड़ा महत्वपूर्ण
- कतई महत्वपूर्ण नहीं

आपके द्वारा चयनित "क्योंकि दूध प्राकृतिक है।"

आपके लिए यह कथन कितना महत्वपूर्ण है।

- अत्यंत महत्वपूर्ण
- बहुत महत्वपूर्ण
- मामूली रूप से महत्वपूर्ण
- थोड़ा महत्वपूर्ण
- कतई महत्वपूर्ण नहीं

आपके द्वारा चयनित "क्योंकि दूध सस्ता है।"

आपके लिए यह कथन कितना महत्वपूर्ण है।

- अत्यंत महत्वपूर्ण
- बहुत महत्वपूर्ण
- मामूली रूप से महत्वपूर्ण
- थोड़ा महत्वपूर्ण
- कतई महत्वपूर्ण नहीं

आपके द्वारा चयनित "क्योंकि दूध में कम कैलोरीज होती है।"

आपके लिए यह कथन कितना महत्वपूर्ण है।

- अत्यंत महत्वपूर्ण
- बहुत महत्वपूर्ण
- मामूली रूप से महत्वपूर्ण

- थोड़ा महत्वपूर्ण
- कतई महत्वपूर्ण नहीं

आपके द्वारा चयनित "क्योंकि मैं निराश रहता हूँ।"

आपके लिए यह कथन कितना महत्वपूर्ण है।

- अत्यंत महत्वपूर्ण
- बहुत महत्वपूर्ण
- मामूली रूप से महत्वपूर्ण
- थोड़ा महत्वपूर्ण
- कतई महत्वपूर्ण नहीं

आपके द्वारा चयनित "क्योंकि दूध खाना मूझे दूसरों के सामने अच्छा दिखाता है।"

आपके लिए यह कथन कितना महत्वपूर्ण है।

- अत्यंत महत्वपूर्ण
- बहुत महत्वपूर्ण
- मामूली रूप से महत्वपूर्ण
- थोड़ा महत्वपूर्ण
- कतई महत्वपूर्ण नहीं

आपके द्वारा चयनित "क्योंकि दूध नहीं खाना अशिष्ट होता है।"

आपके लिए यह कथन कितना महत्वपूर्ण है।

- अत्यंत महत्वपूर्ण
- बहुत महत्वपूर्ण
- मामूली रूप से महत्वपूर्ण
- थोड़ा महत्वपूर्ण
- कतई महत्वपूर्ण नहीं

आपके द्वारा चयनित "क्योंकि दूध कि प्रस्तुति आकर्षक होती है।"

आपके लिए यह कथन कितना महत्वपूर्ण है।

- अत्यंत महत्वपूर्ण
- बहुत महत्वपूर्ण
- मामूली रूप से महत्वपूर्ण
- थोड़ा महत्वपूर्ण
- कतई महत्वपूर्ण नहीं

आपके द्वारा चयनित "ताकि मैं अन्य लोगों के साथ समय बिता सकूं।"

आपके लिए यह कथन कितना महत्वपूर्ण है।

- अत्यंत महत्वपूर्ण
- बहुत महत्वपूर्ण
- मामूली रूप से महत्वपूर्ण
- थोड़ा महत्वपूर्ण
- कतई महत्वपूर्ण नहीं

आपके द्वारा चयनित "क्योंकि दूध जैविक होता है।"

आपके लिए यह कथन कितना महत्वपूर्ण है।

- अत्यंत महत्वपूर्ण
- बहुत महत्वपूर्ण
- मामूली रूप से महत्वपूर्ण
- थोड़ा महत्वपूर्ण
- कतई महत्वपूर्ण नहीं

आपके द्वारा चयनित "क्योंकि मैं बचपन से दूध खाता रहा हूँ।"

आपके लिए यह कथन कितना महत्वपूर्ण है।

- अत्यंत महत्वपूर्ण
- बहुत महत्वपूर्ण
- मामूली रूप से महत्वपूर्ण
- थोड़ा महत्वपूर्ण
- कतई महत्वपूर्ण नहीं

आपके द्वारा चयनित "क्योंकि मैं दूध खाने में आनंद महसूस करता हूँ।"

आपके लिए यह कथन कितना महत्वपूर्ण है।

- अत्यंत महत्वपूर्ण
- बहुत महत्वपूर्ण
- मामूली रूप से महत्वपूर्ण
- थोड़ा महत्वपूर्ण
- कतई महत्वपूर्ण नहीं

आपके द्वारा चयनित "क्योंकि मैं भूखा रहता था।"

आपके लिए यह कथन कितना महत्वपूर्ण है।

- अत्यंत महत्वपूर्ण
- बहुत महत्वपूर्ण
- मामूली रूप से महत्वपूर्ण
- थोड़ा महत्वपूर्ण
- कतई महत्वपूर्ण नहीं

आपके द्वारा चयनित "क्योंकि मैं आमतौर पर दूध खाता हूँ।"

आपके लिए यह कथन कितना महत्वपूर्ण है।

- अत्यंत महत्वपूर्ण
- बहुत महत्वपूर्ण
- मामूली रूप से महत्वपूर्ण
- थोड़ा महत्वपूर्ण
- कतई महत्वपूर्ण नहीं

आपके द्वारा चयनित "क्योंकि मेरे भीतर दूध खाने कि भूख रहती है।"

आपके लिए यह कथन कितना महत्वपूर्ण है।

- अत्यंत महत्वपूर्ण
- बहुत महत्वपूर्ण
- मामूली रूप से महत्वपूर्ण
- थोड़ा महत्वपूर्ण

- कतई महत्वपूर्ण नहीं

आपके द्वारा चयनित "क्योंकि दूध बनाना आसान है।"

आपके लिए यह कथन कितना महत्वपूर्ण है।

- अत्यंत महत्वपूर्ण
- बहुत महत्वपूर्ण
- मामूली रूप से महत्वपूर्ण
- थोड़ा महत्वपूर्ण
- कतई महत्वपूर्ण नहीं

आपके द्वारा चयनित "क्योंकि मैं उदास रहता हूँ।"

आपके लिए यह कथन कितना महत्वपूर्ण है।

- अत्यंत महत्वपूर्ण
- बहुत महत्वपूर्ण
- मामूली रूप से महत्वपूर्ण
- थोड़ा महत्वपूर्ण
- कतई महत्वपूर्ण नहीं

आपके द्वारा चयनित "क्योंकि दूध में वसा कि मात्रा कम होती है।"

आपके लिए यह कथन कितना महत्वपूर्ण है।

- अत्यंत महत्वपूर्ण

- बहुत महत्वपूर्ण
- मामूली रूप से महत्वपूर्ण
- थोड़ा महत्वपूर्ण
- कतई महत्वपूर्ण नहीं

आपके द्वारा चयनित "क्योंकि मैं दूध को टीवी या विज्ञापन पर देखता हूँ।"

आपके लिए यह कथन कितना महत्वपूर्ण है।

- अत्यंत महत्वपूर्ण
- बहुत महत्वपूर्ण
- मामूली रूप से महत्वपूर्ण
- थोड़ा महत्वपूर्ण
- कतई महत्वपूर्ण नहीं

आपके द्वारा चयनित "क्योंकि दूध सामाजिक समारोहों को और अधिक आरामदायक बनाता है।"

आपके लिए यह कथन कितना महत्वपूर्ण है।

- अत्यंत महत्वपूर्ण
- बहुत महत्वपूर्ण
- मामूली रूप से महत्वपूर्ण
- थोड़ा महत्वपूर्ण
- कतई महत्वपूर्ण नहीं

आपके द्वारा चयनित "क्योंकि मूझे दूध खाना पड़ता है।"

आपके लिए यह कथन कितना महत्वपूर्ण है।

- अत्यंत महत्वपूर्ण
- बहुत महत्वपूर्ण
- मामूली रूप से महत्वपूर्ण
- थोड़ा महत्वपूर्ण
- कतई महत्वपूर्ण नहीं

आपके द्वारा चयनित "क्योंकि दूध दूसरों को पसंद है।"

आपके लिए यह कथन कितना महत्वपूर्ण है।

- अत्यंत महत्वपूर्ण
- बहुत महत्वपूर्ण
- मामूली रूप से महत्वपूर्ण
- थोड़ा महत्वपूर्ण
- कतई महत्वपूर्ण नहीं

आपके द्वारा चयनित "क्योंकि मैं अकेला महसूस करता हूँ।"

आपके लिए यह कथन कितना महत्वपूर्ण है।

- अत्यंत महत्वपूर्ण
- बहुत महत्वपूर्ण
- मामूली रूप से महत्वपूर्ण
- थोड़ा महत्वपूर्ण
- कतई महत्वपूर्ण नहीं

आपके द्वारा चयनित "क्योंकि मैं अपने वजन पर निगरानी रखता हूँ।"

आपके लिए यह कथन कितना महत्वपूर्ण है।

- अत्यंत महत्वपूर्ण
- बहुत महत्वपूर्ण
- मामूली रूप से महत्वपूर्ण
- थोड़ा महत्वपूर्ण
- कतई महत्वपूर्ण नहीं

आपके द्वारा चयनित "क्योंकि दूध में कोई हानिकारक (कीटनाशक, प्रदूषक, एंटीबायोटिक) पदार्थ नहीं होता है।"

आपके लिए यह कथन कितना महत्वपूर्ण है।

- अत्यंत महत्वपूर्ण
- बहुत महत्वपूर्ण
- मामूली रूप से महत्वपूर्ण
- थोड़ा महत्वपूर्ण
- कतई महत्वपूर्ण नहीं

आपके द्वारा चयनित "क्योंकि दूध बनाना सुविधाजनक है।"

आपके लिए यह कथन कितना महत्वपूर्ण है।

- अत्यंत महत्वपूर्ण
- बहुत महत्वपूर्ण
- मामूली रूप से महत्वपूर्ण

- थोड़ा महत्वपूर्ण
- कतई महत्वपूर्ण नहीं

आपके द्वारा चयनित "क्योंकि मूझे ऊर्जा की जरूरत होती है।"

आपके लिए यह कथन कितना महत्वपूर्ण है।

- अत्यंत महत्वपूर्ण
- बहुत महत्वपूर्ण
- मामूली रूप से महत्वपूर्ण
- थोड़ा महत्वपूर्ण
- कतई महत्वपूर्ण नहीं

आपके द्वारा चयनित "क्योंकि मैं दूध से परिचित हूँ।"

आपके लिए यह कथन कितना महत्वपूर्ण है।

- अत्यंत महत्वपूर्ण
- बहुत महत्वपूर्ण
- मामूली रूप से महत्वपूर्ण
- थोड़ा महत्वपूर्ण
- कतई महत्वपूर्ण नहीं

आपके द्वारा चयनित "क्योंकि मुझे दूध पसंद है।"

आपके लिए यह कथन कितना महत्वपूर्ण है।

- अत्यंत महत्वपूर्ण
- बहुत महत्वपूर्ण
- मामूली रूप से महत्वपूर्ण
- थोड़ा महत्वपूर्ण
- कतई महत्वपूर्ण नहीं

आपके द्वारा चयनित "क्योंकि दूध खाना सुखदेय है।"

आपके लिए यह कथन कितना महत्वपूर्ण है।

- अत्यंत महत्वपूर्ण
- बहुत महत्वपूर्ण
- मामूली रूप से महत्वपूर्ण
- थोड़ा महत्वपूर्ण
- कतई महत्वपूर्ण नहीं

आपके द्वारा चयनित "मैं अपने आपको पुरस्कृत करने के लिए दूध खाता हूँ।"

आपके लिए यह कथन कितना महत्वपूर्ण है।

- अत्यंत महत्वपूर्ण
- बहुत महत्वपूर्ण
- मामूली रूप से महत्वपूर्ण
- थोड़ा महत्वपूर्ण
- कतई महत्वपूर्ण नहीं

आपके द्वारा चयनित "दूध खाना मेरी परंपरा का हिस्सा है।"

आपके लिए यह कथन कितना महत्वपूर्ण है।

- अत्यंत महत्वपूर्ण
- बहुत महत्वपूर्ण
- मामूली रूप से महत्वपूर्ण
- थोड़ा महत्वपूर्ण
- कतई महत्वपूर्ण नहीं

आपके द्वारा चयनित "क्योंकि दूध कुछ स्थितियों से संबंधित है।"

आपके लिए यह कथन कितना महत्वपूर्ण है।

- अत्यंत महत्वपूर्ण
- बहुत महत्वपूर्ण
- मामूली रूप से महत्वपूर्ण
- थोड़ा महत्वपूर्ण
- कतई महत्वपूर्ण नहीं

आपके द्वारा चयनित "संतुलित आहार बनाए रखने के लिए।"

आपके लिए यह कथन कितना महत्वपूर्ण है।

- अत्यंत महत्वपूर्ण
- बहुत महत्वपूर्ण
- मामूली रूप से महत्वपूर्ण
- थोड़ा महत्वपूर्ण
- कतई महत्वपूर्ण नहीं

आपके द्वारा चयनित "क्योंकि दूध खाना सामाजिक है।"

आपके लिए यह कथन कितना महत्वपूर्ण है।

- अत्यंत महत्वपूर्ण
- बहुत महत्वपूर्ण
- मामूली रूप से महत्वपूर्ण
- थोड़ा महत्वपूर्ण
- कतई महत्वपूर्ण नहीं

आपके द्वारा चयनित "क्योंकि दूध सस्ता है।"

आपके लिए यह कथन कितना महत्वपूर्ण है।

- अत्यंत महत्वपूर्ण
- बहुत महत्वपूर्ण
- मामूली रूप से महत्वपूर्ण
- थोड़ा महत्वपूर्ण
- कतई महत्वपूर्ण नहीं

आपके द्वारा चयनित "क्योंकि दूध मेरे लिए आकर्षक भोजन है।"

आपके लिए यह कथन कितना महत्वपूर्ण है।

- अत्यंत महत्वपूर्ण
- बहुत महत्वपूर्ण
- मामूली रूप से महत्वपूर्ण
- थोड़ा महत्वपूर्ण

- कतई महत्वपूर्ण नहीं

आपके द्वारा चयनित "दुसरो को निराश करने से बचने के लिए।"

आपके लिए यह कथन कितना महत्वपूर्ण है।

- अत्यंत महत्वपूर्ण
- बहुत महत्वपूर्ण
- मामूली रूप से महत्वपूर्ण
- थोड़ा महत्वपूर्ण
- कतई महत्वपूर्ण नहीं

आपके द्वारा चयनित "क्योंकि दूध खाना फैशनेबल है।"

आपके लिए यह कथन कितना महत्वपूर्ण है।

- अत्यंत महत्वपूर्ण
- बहुत महत्वपूर्ण
- मामूली रूप से महत्वपूर्ण
- थोड़ा महत्वपूर्ण
- कतई महत्वपूर्ण नहीं

आपके द्वारा चयनित "क्योंकि दूध विक्रय बिक्री पर होता है।"

आपके लिए यह कथन कितना महत्वपूर्ण है।

- अत्यंत महत्वपूर्ण

- बहुत महत्वपूर्ण
- मामूली रूप से महत्वपूर्ण
- थोड़ा महत्वपूर्ण
- कतई महत्वपूर्ण नहीं

आपके द्वारा चयनित "क्योंकि दूध मेरे शरीर को आकार में रखता है।"

आपके लिए यह कथन कितना महत्वपूर्ण है।

- अत्यंत महत्वपूर्ण
- बहुत महत्वपूर्ण
- मामूली रूप से महत्वपूर्ण
- थोड़ा महत्वपूर्ण
- कतई महत्वपूर्ण नहीं

आपके द्वारा चयनित "क्योंकि दूध ही एकमात्र विकल्प है।"

आपके लिए यह कथन कितना महत्वपूर्ण है।

- अत्यंत महत्वपूर्ण
- बहुत महत्वपूर्ण
- मामूली रूप से महत्वपूर्ण
- थोड़ा महत्वपूर्ण
- कतई महत्वपूर्ण नहीं

आपके द्वारा चयनित "क्योंकि मैं दूध हर दिन खाना चाहता हूँ।"

आपके लिए यह कथन कितना महत्वपूर्ण है।

- ○ अत्यंत महत्वपूर्ण
- ○ बहुत महत्वपूर्ण
- ○ मामूली रूप से महत्वपूर्ण
- ○ थोड़ा महत्वपूर्ण
- ○ कतई महत्वपूर्ण नहीं

कृपया दाल नहीं खाने के सभी कारण बताएं। (सभी विकल्प चुने)

- ☐ दाल कि कीमत बहुत अधिक होती है।
- ☐ मैं दाल नहीं खाना चाहता हूँ।
- ☐ दाल अन्य लोगों के आसपास खाने लायक भोजन नहीं है।
- ☐ दाल स्थिति के लिए उपयुक्त नहीं था।
- ☐ दाल मेरी भूख मिटाने के लिए पर्याप्त नहीं है।
- ☐ दाल मेरी भूख को तृप्त नहीं कर पाती है।
- ☐ दाल खाना मूझे दुखी, अकेला और कुंठित महसूस कराता है।
- ☐ क्योंकि दाल जैविक (आर्गेनिक) नहीं है।
- ☐ मैं आमतौर पर दाल नहीं खाता हूँ।
- ☐ मुझे नहीं लगता कि दाल एक नाश्ता है।
- ☐ दाल मे कैलोरी की मात्रा बहुत अधिक होती है।
- ☐ मेने हाल ही में दाल खायी है। और अब मैं बार-बार दाल नहीं खाना चाहता हूँ।
- ☐ दाल मूझे दिखने में अच्छी नहीं लगती है।
- ☐ दाल स्वास्थ्यय के लिए लाभदायक नहीं है।
- ☐ दाल कि खुराक का आकार मेरे लिए उपयुक्त नहीं है।
- ☐ मेरी दाल खाने कि इच्छा नहीं होती है।
- ☐ मुझे दाल पसंद नहीं है।
- ☐ दाल बनाना मुश्किल होता है।
- ☐ मैं कभी दाल नहीं खाऊंगा।क्योंकि मुझे हर दिन एक ही प्रकार का भोजन खाना पसंद है।
- ☐ मैं दाल खाने से दूसरों के सामने पिछड़ा महसूस करता हूँ।

आपके द्वारा चयनित "मुझे नहीं लगता कि दाल एक नाश्ता है।"

आपके लिए यह कथन कितना महत्वपूर्ण है।

- अत्यंत महत्वपूर्ण
- बहुत महत्वपूर्ण
- मामूली रूप से महत्वपूर्ण
- थोड़ा महत्वपूर्ण
- कतई महत्वपूर्ण नहीं

आपके द्वारा चयनित "मुझे दाल पसंद नहीं है।"

आपके लिए यह कथन कितना महत्वपूर्ण है।

- अत्यंत महत्वपूर्ण
- बहुत महत्वपूर्ण
- मामूली रूप से महत्वपूर्ण
- थोड़ा महत्वपूर्ण
- कतई महत्वपूर्ण नहीं

आपके द्वारा चयनित "मैं आमतौर पर दाल नहीं खाता हूँ।"

आपके लिए यह कथन कितना महत्वपूर्ण है।

- अत्यंत महत्वपूर्ण
- बहुत महत्वपूर्ण
- मामूली रूप से महत्वपूर्ण

- थोड़ा महत्वपूर्ण
- कतई महत्वपूर्ण नहीं

आपके द्वारा चयनित "दाल कि खुराक का आकार मेरे लिए उपयुक्त नहीं है।"

आपके लिए यह कथन कितना महत्वपूर्ण है।

- अत्यंत महत्वपूर्ण
- बहुत महत्वपूर्ण
- मामूली रूप से महत्वपूर्ण
- थोड़ा महत्वपूर्ण
- कतई महत्वपूर्ण नहीं

आपके द्वारा चयनित "दाल मेरी भूख मिटाने के लिए पर्याप्त नहीं है।"

आपके लिए यह कथन कितना महत्वपूर्ण है।

- अत्यंत महत्वपूर्ण
- बहुत महत्वपूर्ण
- मामूली रूप से महत्वपूर्ण
- थोड़ा महत्वपूर्ण
- कतई महत्वपूर्ण नहीं

आपके द्वारा चयनित "दाल स्वास्थ्यय के लिए लाभदायक नहीं है।"

आपके लिए यह कथन कितना महत्वपूर्ण है।

- अत्यंत महत्वपूर्ण
- बहुत महत्वपूर्ण
- मामूली रूप से महत्वपूर्ण
- थोड़ा महत्वपूर्ण
- कतई महत्वपूर्ण नहीं

आपके द्वारा चयनित "दाल बनाना मुश्किल होता है।"

आपके लिए यह कथन कितना महत्वपूर्ण है।

- अत्यंत महत्वपूर्ण
- बहुत महत्वपूर्ण
- मामूली रूप से महत्वपूर्ण
- थोड़ा महत्वपूर्ण
- कतई महत्वपूर्ण नहीं

आपके द्वारा चयनित "दाल मेरी भूख को तृप्त नहीं कर पाती है।"

आपके लिए यह कथन कितना महत्वपूर्ण है।

- अत्यंत महत्वपूर्ण
- बहुत महत्वपूर्ण
- मामूली रूप से महत्वपूर्ण
- थोड़ा महत्वपूर्ण
- कतई महत्वपूर्ण नहीं

आपके द्वारा चयनित "दाल स्थिति के लिए उपयुक्त नहीं था।"

आपके लिए यह कथन कितना महत्वपूर्ण है।

- अत्यंत महत्वपूर्ण
- बहुत महत्वपूर्ण
- मामूली रूप से महत्वपूर्ण
- थोड़ा महत्वपूर्ण
- कतई महत्वपूर्ण नहीं

आपके द्वारा चयनित "दाल अन्य लोगों के आसपास खाने लायक भोजन नहीं है।"

आपके लिए यह कथन कितना महत्वपूर्ण है।

- अत्यंत महत्वपूर्ण
- बहुत महत्वपूर्ण
- मामूली रूप से महत्वपूर्ण
- थोड़ा महत्वपूर्ण
- कतई महत्वपूर्ण नहीं

आपके द्वारा चयनित "दाल कि कीमत बहुत अधिक होती है।"

आपके लिए यह कथन कितना महत्वपूर्ण है।

- अत्यंत महत्वपूर्ण
- बहुत महत्वपूर्ण
- मामूली रूप से महत्वपूर्ण
- थोड़ा महत्वपूर्ण
- कतई महत्वपूर्ण नहीं

आपके द्वारा चयनित "दाल मूझे दिखने में अच्छी नहीं लगती है।"

आपके लिए यह कथन कितना महत्वपूर्ण है।

- अत्यंत महत्वपूर्ण
- बहुत महत्वपूर्ण
- मामूली रूप से महत्वपूर्ण
- थोड़ा महत्वपूर्ण
- कतई महत्वपूर्ण नहीं

आपके द्वारा चयनित "दाल मे कैलोरी की मात्रा बहुत अधिक होती है।"

आपके लिए यह कथन कितना महत्वपूर्ण है।

- अत्यंत महत्वपूर्ण
- बहुत महत्वपूर्ण
- मामूली रूप से महत्वपूर्ण
- थोड़ा महत्वपूर्ण
- कतई महत्वपूर्ण नहीं

आपके द्वारा चयनित "मैं दाल नहीं खाना चाहता हूँ।"

आपके लिए यह कथन कितना महत्वपूर्ण है।

- अत्यंत महत्वपूर्ण
- बहुत महत्वपूर्ण
- मामूली रूप से महत्वपूर्ण
- थोड़ा महत्वपूर्ण

- कतई महत्वपूर्ण नहीं

आपके द्वारा चयनित "मैं दाल खाने से दूसरों के सामने पिछड़ा महसूस करता हूँ।"

आपके लिए यह कथन कितना महत्वपूर्ण है।

- अत्यंत महत्वपूर्ण
- बहुत महत्वपूर्ण
- मामूली रूप से महत्वपूर्ण
- थोड़ा महत्वपूर्ण
- कतई महत्वपूर्ण नहीं

आपके द्वारा चयनित "मेरी दाल खाने कि इच्छा नहीं होती है।"

आपके लिए यह कथन कितना महत्वपूर्ण है।

- अत्यंत महत्वपूर्ण
- बहुत महत्वपूर्ण
- मामूली रूप से महत्वपूर्ण
- थोड़ा महत्वपूर्ण
- कतई महत्वपूर्ण नहीं

आपके द्वारा चयनित "मेने हाल ही में दाल खायी है। और अब मैं बार-बार दाल नहीं खाना चाहता हूँ।"

आपके लिए यह कथन कितना महत्वपूर्ण है।

- अत्यंत महत्वपूर्ण

- बहुत महत्वपूर्ण
- मामूली रूप से महत्वपूर्ण
- थोड़ा महत्वपूर्ण
- कतई महत्वपूर्ण नहीं

आपके द्वारा चयनित "मैं कभी दाल नहीं खाऊंगा।क्योंकि मुझे हर दिन एक ही प्रकार का भोजन खाना पसंद है।"

आपके लिए यह कथन कितना महत्वपूर्ण है।

- अत्यंत महत्वपूर्ण
- बहुत महत्वपूर्ण
- मामूली रूप से महत्वपूर्ण
- थोड़ा महत्वपूर्ण
- कतई महत्वपूर्ण नहीं

आपके द्वारा चयनित "दाल खाना मूझे दुखी, अकेला और कुंठित महसूस कराता है।"

आपके लिए यह कथन कितना महत्वपूर्ण है।

- अत्यंत महत्वपूर्ण
- बहुत महत्वपूर्ण
- मामूली रूप से महत्वपूर्ण
- थोड़ा महत्वपूर्ण
- कतई महत्वपूर्ण नहीं

आपके द्वारा चयनित "क्योंकि दाल जैविक (आर्गेनिक) नहीं है।"

आपके लिए यह कथन कितना महत्वपूर्ण है।

- ○ अत्यंत महत्वपूर्ण
- ○ बहुत महत्वपूर्ण
- ○ मामूली रूप से महत्वपूर्ण
- ○ थोड़ा महत्वपूर्ण
- ○ कतई महत्वपूर्ण नहीं

Reasons for Dairy Food Choices(RATA)

क्या आप दूध खाते हैं।

- ○ हाँ
- ○ नहीं

किन कारणों के लिए आप दूध खातें हैं। (सभी विकल्प चुने)

- ☐ क्योंकि दूध का स्वाद अच्छा है।
- ☐ ताकि मैं अन्य लोगों के साथ समय बिता सकूं।
- ☐ क्योंकि दूध जैविक होता है।
- ☐ क्योंकि दूध खाना स्वास्थ्य के लिए लाभदायक है।
- ☐ क्योंकि दूध खाना फैशनेबल है।
- ☐ क्योंकि दूध कुछ स्थितियों से संबंधित है।
- ☐ संतुलित आहार बनाए रखने के लिए।
- ☐ क्योंकि दूध कि प्रस्तुति आकर्षक होती है।
- ☐ क्योंकि दूध सस्ता है।
- ☐ क्योंकि दूध ही एकमात्र विकल्प है।
- ☐ क्योंकि दूध या दूध से सम्बंदित भोज कम समय में बन जाता है।
- ☐ क्योंकि दूध बनाना आसान है।

- ☐ क्योंकि मैं दूध खाने का आदी हूँ।
- ☐ मैं अपने आपको पुरस्कृत करने के लिए दूध खाता हूँ।
- ☐ क्योंकि दूध मेरे लिए आकर्षक भोजन है।
- ☐ क्योंकि दूध में वसा कि मात्रा कम होती है।
- ☐ क्योंकि दूध प्राकृतिक है।
- ☐ क्योंकि दूध खाना सुखदेय है।
- ☐ क्योंकि मूझे ऊर्जा की जरूरत होती है।
- ☐ दुसरो को निराश करने से बचने के लिए।
- ☐ क्योंकि मेरे भीतर दूध खाने कि भूख रहती है।
- ☐ क्योंकि दूध खाना मूझे दूसरों के सामने अच्छा दिखाता है।
- ☐ क्योंकि दूध दूसरों को पसंद है।
- ☐ क्योंकि मैं दूध खाने में आनंद महसूस करता हूँ।
- ☐ क्योंकि मैं उदास रहता हूँ।
- ☐ क्योंकि दूध सामाजिक समारोहों को और अधिक आरामदायक बनाता है।
- ☐ क्योंकि दूध खाना सामाजिक है।
- ☐ क्योंकि मैं अपने वजन पर निगरानी रखता हूँ।
- ☐ क्योंकि मैं आमतौर पर दूध खाता हूँ।
- ☐ क्योंकि मैं भूखा रहता था।
- ☐ क्योंकि मैं निराश रहता हूँ।
- ☐ दूध खाना मेरी परंपरा का हिस्सा है।
- ☐ क्योंकि दूध खाना मुझे तृप्त करता है।
- ☐ क्योंकि दूध में कम कैलोरीज होती है।
- ☐ क्योंकि दूध में कोई हानिकारक (कीटनाशक, प्रदूषक, एंटीबायोटिक) पदार्थ नहीं होता है।
- ☐ क्योंकि दूध विक्रय बिक्री पर होता है।
- ☐ क्योंकि दूध नहीं खाना अशिष्ट होता है।
- ☐ क्योंकि दूध बनाना सुविधाजनक है।
- ☐ क्योंकि मूझे दूध खाना पड़ता है।
- ☐ क्योंकि मैं दूध हर दिन खाना चाहता हूँ।
- ☐ क्योंकि मैं बचपन से दूध खाता रहा हूँ।

- ☐ क्योंकि मैं दूध से परिचित हूँ।
- ☐ क्योंकि मैं दूध को टीवी या विज्ञापन पर देखता हूँ।
- ☐ क्योंकि दूध सस्ता है।
- ☐ क्योंकि दूध मेरे शरीर को आकार में रखता है।
- ☐ क्योंकि मैं अकेला महसूस करता हूँ।
- ☐ क्योंकि मुझे दूध पसंद है।

आपके द्वारा चयनित "क्योंकि मैं दूध खाने का आदी हूँ।"

आपके लिए यह कथन कितना महत्वपूर्ण है।

- ○ अत्यंत महत्वपूर्ण
- ○ बहुत महत्वपूर्ण
- ○ मामूली रूप से महत्वपूर्ण
- ○ थोड़ा महत्वपूर्ण
- ○ कतई महत्वपूर्ण नहीं

आपके द्वारा चयनित "क्योंकि दूध खाना स्वास्थ्य के लिए लाभदायक है।"

आपके लिए यह कथन कितना महत्वपूर्ण है।

- ○ अत्यंत महत्वपूर्ण
- ○ बहुत महत्वपूर्ण
- ○ मामूली रूप से महत्वपूर्ण
- ○ थोड़ा महत्वपूर्ण
- ○ कतई महत्वपूर्ण नहीं

आपके द्वारा चयनित "क्योंकि दूध या दूध से सम्बंदित भोज कम समय में बन जाता है।"

आपके लिए यह कथन कितना महत्वपूर्ण है।

- अत्यंत महत्वपूर्ण
- बहुत महत्वपूर्ण
- मामूली रूप से महत्वपूर्ण
- थोड़ा महत्वपूर्ण
- कतई महत्वपूर्ण नहीं

आपके द्वारा चयनित "क्योंकि दूध का स्वाद अच्छा है।"

आपके लिए यह कथन कितना महत्वपूर्ण है।

- अत्यंत महत्वपूर्ण
- बहुत महत्वपूर्ण
- मामूली रूप से महत्वपूर्ण
- थोड़ा महत्वपूर्ण
- कतई महत्वपूर्ण नहीं

आपके द्वारा चयनित "क्योंकि दूध खाना मुझे तृप्त करता है।"

आपके लिए यह कथन कितना महत्वपूर्ण है।

- अत्यंत महत्वपूर्ण
- बहुत महत्वपूर्ण
- मामूली रूप से महत्वपूर्ण
- थोड़ा महत्वपूर्ण
- कतई महत्वपूर्ण नहीं

आपके द्वारा चयनित "क्योंकि दूध प्राकृतिक है।"

आपके लिए यह कथन कितना महत्वपूर्ण है।

- अत्यंत महत्वपूर्ण
- बहुत महत्वपूर्ण
- मामूली रूप से महत्वपूर्ण
- थोड़ा महत्वपूर्ण
- कतई महत्वपूर्ण नहीं

आपके द्वारा चयनित "क्योंकि दूध सस्ता है।"

आपके लिए यह कथन कितना महत्वपूर्ण है।

- अत्यंत महत्वपूर्ण
- बहुत महत्वपूर्ण
- मामूली रूप से महत्वपूर्ण
- थोड़ा महत्वपूर्ण
- कतई महत्वपूर्ण नहीं

आपके द्वारा चयनित "क्योंकि दूध में कम कैलोरीज होती है।"

आपके लिए यह कथन कितना महत्वपूर्ण है।

- अत्यंत महत्वपूर्ण
- बहुत महत्वपूर्ण
- मामूली रूप से महत्वपूर्ण

- थोड़ा महत्वपूर्ण
- कतई महत्वपूर्ण नहीं

आपके द्वारा चयनित "क्योंकि मैं निराश रहता हूँ।"

आपके लिए यह कथन कितना महत्वपूर्ण है।

- अत्यंत महत्वपूर्ण
- बहुत महत्वपूर्ण
- मामूली रूप से महत्वपूर्ण
- थोड़ा महत्वपूर्ण
- कतई महत्वपूर्ण नहीं

आपके द्वारा चयनित "क्योंकि दूध खाना मूझे दूसरों के सामने अच्छा दिखाता है।"

आपके लिए यह कथन कितना महत्वपूर्ण है।

- अत्यंत महत्वपूर्ण
- बहुत महत्वपूर्ण
- मामूली रूप से महत्वपूर्ण
- थोड़ा महत्वपूर्ण
- कतई महत्वपूर्ण नहीं

आपके द्वारा चयनित "क्योंकि दूध नहीं खाना अशिष्ट होता है।"

आपके लिए यह कथन कितना महत्वपूर्ण है।

- अत्यंत महत्वपूर्ण
- बहुत महत्वपूर्ण
- मामूली रूप से महत्वपूर्ण
- थोड़ा महत्वपूर्ण
- कतई महत्वपूर्ण नहीं

आपके द्वारा चयनित "क्योंकि दूध कि प्रस्तुति आकर्षक होती है।"

आपके लिए यह कथन कितना महत्वपूर्ण है।

- अत्यंत महत्वपूर्ण
- बहुत महत्वपूर्ण
- मामूली रूप से महत्वपूर्ण
- थोड़ा महत्वपूर्ण
- कतई महत्वपूर्ण नहीं

आपके द्वारा चयनित "ताकि मैं अन्य लोगों के साथ समय बिता सकूं।"

आपके लिए यह कथन कितना महत्वपूर्ण है।

- अत्यंत महत्वपूर्ण
- बहुत महत्वपूर्ण
- मामूली रूप से महत्वपूर्ण
- थोड़ा महत्वपूर्ण
- कतई महत्वपूर्ण नहीं

आपके द्वारा चयनित "क्योंकि दूध जैविक होता है।"

आपके लिए यह कथन कितना महत्वपूर्ण है।

- अत्यंत महत्वपूर्ण
- बहुत महत्वपूर्ण
- मामूली रूप से महत्वपूर्ण
- थोड़ा महत्वपूर्ण
- कतई महत्वपूर्ण नहीं

आपके द्वारा चयनित "क्योंकि मैं बचपन से दूध खाता रहा हूँ।"

आपके लिए यह कथन कितना महत्वपूर्ण है।

- अत्यंत महत्वपूर्ण
- बहुत महत्वपूर्ण
- मामूली रूप से महत्वपूर्ण
- थोड़ा महत्वपूर्ण
- कतई महत्वपूर्ण नहीं

आपके द्वारा चयनित "क्योंकि मैं दूध खाने में आनंद महसूस करता हूँ।"

आपके लिए यह कथन कितना महत्वपूर्ण है।

- अत्यंत महत्वपूर्ण
- बहुत महत्वपूर्ण
- मामूली रूप से महत्वपूर्ण
- थोड़ा महत्वपूर्ण
- कतई महत्वपूर्ण नहीं

आपके द्वारा चयनित "क्योंकि मैं भूखा रहता था।"

आपके लिए यह कथन कितना महत्वपूर्ण है।

- अत्यंत महत्वपूर्ण
- बहुत महत्वपूर्ण
- मामूली रूप से महत्वपूर्ण
- थोड़ा महत्वपूर्ण
- कतई महत्वपूर्ण नहीं

आपके द्वारा चयनित "क्योंकि मैं आमतौर पर दूध खाता हूँ।"

आपके लिए यह कथन कितना महत्वपूर्ण है।

- अत्यंत महत्वपूर्ण
- बहुत महत्वपूर्ण
- मामूली रूप से महत्वपूर्ण
- थोड़ा महत्वपूर्ण
- कतई महत्वपूर्ण नहीं

आपके द्वारा चयनित "क्योंकि मेरे भीतर दूध खाने कि भूख रहती है।"

आपके लिए यह कथन कितना महत्वपूर्ण है।

- अत्यंत महत्वपूर्ण
- बहुत महत्वपूर्ण
- मामूली रूप से महत्वपूर्ण
- थोड़ा महत्वपूर्ण

- कतई महत्वपूर्ण नहीं

आपके द्वारा चयनित "क्योंकि दूध बनाना आसान है।"

आपके लिए यह कथन कितना महत्वपूर्ण है।

- अत्यंत महत्वपूर्ण
- बहुत महत्वपूर्ण
- मामूली रूप से महत्वपूर्ण
- थोड़ा महत्वपूर्ण
- कतई महत्वपूर्ण नहीं

आपके द्वारा चयनित "क्योंकि मैं उदास रहता हूँ।"

आपके लिए यह कथन कितना महत्वपूर्ण है।

- अत्यंत महत्वपूर्ण
- बहुत महत्वपूर्ण
- मामूली रूप से महत्वपूर्ण
- थोड़ा महत्वपूर्ण
- कतई महत्वपूर्ण नहीं

आपके द्वारा चयनित "क्योंकि दूध में वसा कि मात्रा कम होती है।"

आपके लिए यह कथन कितना महत्वपूर्ण है।

- अत्यंत महत्वपूर्ण

- बहुत महत्वपूर्ण
- मामूली रूप से महत्वपूर्ण
- थोड़ा महत्वपूर्ण
- कतई महत्वपूर्ण नहीं

आपके द्वारा चयनित "क्योंकि मैं दूध को टीवी या विज्ञापन पर देखता हूँ।"

आपके लिए यह कथन कितना महत्वपूर्ण है।

- अत्यंत महत्वपूर्ण
- बहुत महत्वपूर्ण
- मामूली रूप से महत्वपूर्ण
- थोड़ा महत्वपूर्ण
- कतई महत्वपूर्ण नहीं

आपके द्वारा चयनित "क्योंकि दूध सामाजिक समारोहों को और अधिक आरामदायक बनाता है।"

आपके लिए यह कथन कितना महत्वपूर्ण है।

- अत्यंत महत्वपूर्ण
- बहुत महत्वपूर्ण
- मामूली रूप से महत्वपूर्ण
- थोड़ा महत्वपूर्ण
- कतई महत्वपूर्ण नहीं

आपके द्वारा चयनित "क्योंकि मूझे दूध खाना पड़ता है।"

आपके लिए यह कथन कितना महत्वपूर्ण है।

- अत्यंत महत्वपूर्ण
- बहुत महत्वपूर्ण
- मामूली रूप से महत्वपूर्ण
- थोड़ा महत्वपूर्ण
- कतई महत्वपूर्ण नहीं

आपके द्वारा चयनित "क्योंकि दूध दूसरों को पसंद है।"

आपके लिए यह कथन कितना महत्वपूर्ण है।

- अत्यंत महत्वपूर्ण
- बहुत महत्वपूर्ण
- मामूली रूप से महत्वपूर्ण
- थोड़ा महत्वपूर्ण
- कतई महत्वपूर्ण नहीं

आपके द्वारा चयनित "क्योंकि मैं अकेला महसूस करता हूँ।"

आपके लिए यह कथन कितना महत्वपूर्ण है।

- अत्यंत महत्वपूर्ण
- बहुत महत्वपूर्ण
- मामूली रूप से महत्वपूर्ण
- थोड़ा महत्वपूर्ण
- कतई महत्वपूर्ण नहीं

आपके द्वारा चयनित "क्योंकि मैं अपने वजन पर निगरानी रखता हूँ।"

आपके लिए यह कथन कितना महत्वपूर्ण है।

- अत्यंत महत्वपूर्ण
- बहुत महत्वपूर्ण
- मामूली रूप से महत्वपूर्ण
- थोड़ा महत्वपूर्ण
- कतई महत्वपूर्ण नहीं

आपके द्वारा चयनित "क्योंकि दूध में कोई हानिकारक (कीटनाशक, प्रदूषक, एंटीबायोटिक) पदार्थ नहीं होता है।"

आपके लिए यह कथन कितना महत्वपूर्ण है।

- अत्यंत महत्वपूर्ण
- बहुत महत्वपूर्ण
- मामूली रूप से महत्वपूर्ण
- थोड़ा महत्वपूर्ण
- कतई महत्वपूर्ण नहीं

आपके द्वारा चयनित "क्योंकि दूध बनाना सुविधाजनक है।"

आपके लिए यह कथन कितना महत्वपूर्ण है।

- अत्यंत महत्वपूर्ण
- बहुत महत्वपूर्ण
- मामूली रूप से महत्वपूर्ण

- थोड़ा महत्वपूर्ण
- कतई महत्वपूर्ण नहीं

आपके द्वारा चयनित "क्योंकि मूझे ऊर्जा की जरूरत होती है।"

आपके लिए यह कथन कितना महत्वपूर्ण है।

- अत्यंत महत्वपूर्ण
- बहुत महत्वपूर्ण
- मामूली रूप से महत्वपूर्ण
- थोड़ा महत्वपूर्ण
- कतई महत्वपूर्ण नहीं

आपके द्वारा चयनित "क्योंकि मैं दूध से परिचित हूँ।"

आपके लिए यह कथन कितना महत्वपूर्ण है।

- अत्यंत महत्वपूर्ण
- बहुत महत्वपूर्ण
- मामूली रूप से महत्वपूर्ण
- थोड़ा महत्वपूर्ण
- कतई महत्वपूर्ण नहीं

आपके द्वारा चयनित "क्योंकि मुझे दूध पसंद है।"

आपके लिए यह कथन कितना महत्वपूर्ण है।

- अत्यंत महत्वपूर्ण
- बहुत महत्वपूर्ण
- मामूली रूप से महत्वपूर्ण
- थोड़ा महत्वपूर्ण
- कतई महत्वपूर्ण नहीं

आपके द्वारा चयनित "क्योंकि दूध खाना सुखदेय है।"

आपके लिए यह कथन कितना महत्वपूर्ण है।

- अत्यंत महत्वपूर्ण
- बहुत महत्वपूर्ण
- मामूली रूप से महत्वपूर्ण
- थोड़ा महत्वपूर्ण
- कतई महत्वपूर्ण नहीं

आपके द्वारा चयनित "मैं अपने आपको पुरस्कृत करने के लिए दूध खाता हूँ।"

आपके लिए यह कथन कितना महत्वपूर्ण है।

- अत्यंत महत्वपूर्ण
- बहुत महत्वपूर्ण
- मामूली रूप से महत्वपूर्ण
- थोड़ा महत्वपूर्ण
- कतई महत्वपूर्ण नहीं

आपके द्वारा चयनित "दूध खाना मेरी परंपरा का हिस्सा है।"

आपके लिए यह कथन कितना महत्वपूर्ण है।

- अत्यंत महत्वपूर्ण
- बहुत महत्वपूर्ण
- मामूली रूप से महत्वपूर्ण
- थोड़ा महत्वपूर्ण
- कतई महत्वपूर्ण नहीं

आपके द्वारा चयनित "क्योंकि दूध कुछ स्थितियों से संबंधित है।"

आपके लिए यह कथन कितना महत्वपूर्ण है।

- अत्यंत महत्वपूर्ण
- बहुत महत्वपूर्ण
- मामूली रूप से महत्वपूर्ण
- थोड़ा महत्वपूर्ण
- कतई महत्वपूर्ण नहीं

आपके द्वारा चयनित "संतुलित आहार बनाए रखने के लिए।"

आपके लिए यह कथन कितना महत्वपूर्ण है।

- अत्यंत महत्वपूर्ण
- बहुत महत्वपूर्ण
- मामूली रूप से महत्वपूर्ण
- थोड़ा महत्वपूर्ण
- कतई महत्वपूर्ण नहीं

आपके द्वारा चयनित "क्योंकि दूध खाना सामाजिक है।"

आपके लिए यह कथन कितना महत्वपूर्ण है।

- अत्यंत महत्वपूर्ण
- बहुत महत्वपूर्ण
- मामूली रूप से महत्वपूर्ण
- थोड़ा महत्वपूर्ण
- कतई महत्वपूर्ण नहीं

आपके द्वारा चयनित "क्योंकि दूध सस्ता है।"

आपके लिए यह कथन कितना महत्वपूर्ण है।

- अत्यंत महत्वपूर्ण
- बहुत महत्वपूर्ण
- मामूली रूप से महत्वपूर्ण
- थोड़ा महत्वपूर्ण
- कतई महत्वपूर्ण नहीं

आपके द्वारा चयनित "क्योंकि दूध मेरे लिए आकर्षक भोजन है।"

आपके लिए यह कथन कितना महत्वपूर्ण है।

- अत्यंत महत्वपूर्ण
- बहुत महत्वपूर्ण
- मामूली रूप से महत्वपूर्ण
- थोड़ा महत्वपूर्ण

- कतई महत्वपूर्ण नहीं

आपके द्वारा चयनित "दुसरो को निराश करने से बचने के लिए।"

आपके लिए यह कथन कितना महत्वपूर्ण है।

- अत्यंत महत्वपूर्ण
- बहुत महत्वपूर्ण
- मामूली रूप से महत्वपूर्ण
- थोड़ा महत्वपूर्ण
- कतई महत्वपूर्ण नहीं

आपके द्वारा चयनित "क्योंकि दूध खाना फैशनेबल है।"

आपके लिए यह कथन कितना महत्वपूर्ण है।

- अत्यंत महत्वपूर्ण
- बहुत महत्वपूर्ण
- मामूली रूप से महत्वपूर्ण
- थोड़ा महत्वपूर्ण
- कतई महत्वपूर्ण नहीं

आपके द्वारा चयनित "क्योंकि दूध विक्रय बिक्री पर होता है।"

आपके लिए यह कथन कितना महत्वपूर्ण है।

- अत्यंत महत्वपूर्ण

- बहुत महत्वपूर्ण
- मामूली रूप से महत्वपूर्ण
- थोड़ा महत्वपूर्ण
- कतई महत्वपूर्ण नहीं

आपके द्वारा चयनित "क्योंकि दूध मेरे शरीर को आकार में रखता है।"

आपके लिए यह कथन कितना महत्वपूर्ण है।

- अत्यंत महत्वपूर्ण
- बहुत महत्वपूर्ण
- मामूली रूप से महत्वपूर्ण
- थोड़ा महत्वपूर्ण
- कतई महत्वपूर्ण नहीं

आपके द्वारा चयनित "क्योंकि दूध ही एकमात्र विकल्प है।"

आपके लिए यह कथन कितना महत्वपूर्ण है।

- अत्यंत महत्वपूर्ण
- बहुत महत्वपूर्ण
- मामूली रूप से महत्वपूर्ण
- थोड़ा महत्वपूर्ण
- कतई महत्वपूर्ण नहीं

आपके द्वारा चयनित "क्योंकि मैं दूध हर दिन खाना चाहता हूँ।"

आपके लिए यह कथन कितना महत्वपूर्ण है।

- ○ अत्यंत महत्वपूर्ण
- ○ बहुत महत्वपूर्ण
- ○ मामूली रूप से महत्वपूर्ण
- ○ थोड़ा महत्वपूर्ण
- ○ कतई महत्वपूर्ण नहीं

कृपया दूध नहीं खाने के सभी कारण बताएं। (सभी विकल्प चुने)

- ☐ मैं आमतौर पर दूध नहीं खाता हूँ।
- ☐ दूध खाना मूझे दुखी, अकेला और कुंठित महसूस कराता है।
- ☐ दूध कि कीमत बहुत अधिक होती है।
- ☐ दूध स्थिति के लिए उपयुक्त नहीं था।
- ☐ मैं कभी दूध नहीं खाऊंगा।क्योंकि मुझे हर दिन एक ही प्रकार का भोजन खाना पसंद है।
- ☐ मुझे दूध पसंद नहीं है।
- ☐ दूध मेरी भूख मिटाने के लिए पर्याप्त नहीं है।
- ☐ मैं दूध खाने से दूसरों के सामने पिछड़ा महसूस करता हूँ।
- ☐ दूध कि खुराक का आकार मेरे लिए उपयुक्त नहीं है।
- ☐ दूध मूझे दिखने में अच्छा नहीं लगता है।
- ☐ क्योंकि दूध जैविक (आर्गेनिक) नहीं है।
- ☐ दूध मे कैलोरी की मात्रा बहुत अधिक होती है।
- ☐ दूध स्वास्थ्यय के लिए लाभदायक नहीं है।
- ☐ मेरी दूध खाने कि इच्छा नहीं होती है।
- ☐ मेने हाल ही में दूध खाया है। और अब मैं बार-बार दूध नहीं खाना चाहता हूँ।
- ☐ दूध मेरी भूख को तृप्त नहीं कर पाता है।
- ☐ मैं दूध नहीं खाना चाहता हूँ।
- ☐ दूध अन्य लोगों के आसपास खाने लायक भोजन नहीं है।
- ☐ दूध बनाना मुश्किल होता है।
- ☐ मुझे नहीं लगता कि दूध एक नाश्ता है।

आपके द्वारा चयनित "मुझे नहीं लगता कि दूध एक नाश्ता है।"

आपके लिए यह कथन कितना महत्वपूर्ण है।

- अत्यंत महत्वपूर्ण
- बहुत महत्वपूर्ण
- मामूली रूप से महत्वपूर्ण
- थोड़ा महत्वपूर्ण
- कतई महत्वपूर्ण नहीं

आपके द्वारा चयनित "मुझे दूध पसंद नहीं है।"

आपके लिए यह कथन कितना महत्वपूर्ण है।

- अत्यंत महत्वपूर्ण
- बहुत महत्वपूर्ण
- मामूली रूप से महत्वपूर्ण
- थोड़ा महत्वपूर्ण
- कतई महत्वपूर्ण नहीं

आपके द्वारा चयनित "मैं आमतौर पर दूध नहीं खाता हूँ।"

आपके लिए यह कथन कितना महत्वपूर्ण है।

- अत्यंत महत्वपूर्ण
- बहुत महत्वपूर्ण
- मामूली रूप से महत्वपूर्ण

- थोड़ा महत्वपूर्ण
- कतई महत्वपूर्ण नहीं

आपके द्वारा चयनित "दूध कि खुराक का आकार मेरे लिए उपयुक्त नहीं है।"

आपके लिए यह कथन कितना महत्वपूर्ण है।

- अत्यंत महत्वपूर्ण
- बहुत महत्वपूर्ण
- मामूली रूप से महत्वपूर्ण
- थोड़ा महत्वपूर्ण
- कतई महत्वपूर्ण नहीं

आपके द्वारा चयनित "दूध मेरी भूख मिटाने के लिए पर्याप्त नहीं है।"

आपके लिए यह कथन कितना महत्वपूर्ण है।

- अत्यंत महत्वपूर्ण
- बहुत महत्वपूर्ण
- मामूली रूप से महत्वपूर्ण
- थोड़ा महत्वपूर्ण
- कतई महत्वपूर्ण नहीं

आपके द्वारा चयनित "दूध स्वास्थ्यय के लिए लाभदायक नहीं है।"

आपके लिए यह कथन कितना महत्वपूर्ण है।

- अत्यंत महत्वपूर्ण
- बहुत महत्वपूर्ण
- मामूली रूप से महत्वपूर्ण
- थोड़ा महत्वपूर्ण
- कतई महत्वपूर्ण नहीं

आपके द्वारा चयनित "दूध बनाना मुश्किल होता है।"

आपके लिए यह कथन कितना महत्वपूर्ण है।

- अत्यंत महत्वपूर्ण
- बहुत महत्वपूर्ण
- मामूली रूप से महत्वपूर्ण
- थोड़ा महत्वपूर्ण
- कतई महत्वपूर्ण नहीं

आपके द्वारा चयनित "दूध मेरी भूख को तृप्त नहीं कर पाता है।"

आपके लिए यह कथन कितना महत्वपूर्ण है।

- अत्यंत महत्वपूर्ण
- बहुत महत्वपूर्ण
- मामूली रूप से महत्वपूर्ण
- थोड़ा महत्वपूर्ण
- कतई महत्वपूर्ण नहीं

आपके द्वारा चयनित "दूध स्थिति के लिए उपयुक्त नहीं था।"

आपके लिए यह कथन कितना महत्वपूर्ण है।

- अत्यंत महत्वपूर्ण
- बहुत महत्वपूर्ण
- मामूली रूप से महत्वपूर्ण
- थोड़ा महत्वपूर्ण
- कतई महत्वपूर्ण नहीं

आपके द्वारा चयनित "दूध अन्य लोगों के आसपास खाने लायक भोजन नहीं है।"

आपके लिए यह कथन कितना महत्वपूर्ण है।

- अत्यंत महत्वपूर्ण
- बहुत महत्वपूर्ण
- मामूली रूप से महत्वपूर्ण
- थोड़ा महत्वपूर्ण
- कतई महत्वपूर्ण नहीं

आपके द्वारा चयनित "दूध कि कीमत बहुत अधिक होती है।"

आपके लिए यह कथन कितना महत्वपूर्ण है।

- अत्यंत महत्वपूर्ण
- बहुत महत्वपूर्ण
- मामूली रूप से महत्वपूर्ण
- थोड़ा महत्वपूर्ण
- कतई महत्वपूर्ण नहीं

आपके द्वारा चयनित "दूध मूझे दिखने में अच्छा नहीं लगता है।"

आपके लिए यह कथन कितना महत्वपूर्ण है।

- अत्यंत महत्वपूर्ण
- बहुत महत्वपूर्ण
- मामूली रूप से महत्वपूर्ण
- थोड़ा महत्वपूर्ण
- कतई महत्वपूर्ण नहीं

आपके द्वारा चयनित "दूध मे कैलोरी की मात्रा बहुत अधिक होती है।"

आपके लिए यह कथन कितना महत्वपूर्ण है।

- अत्यंत महत्वपूर्ण
- बहुत महत्वपूर्ण
- मामूली रूप से महत्वपूर्ण
- थोड़ा महत्वपूर्ण
- कतई महत्वपूर्ण नहीं

आपके द्वारा चयनित "मैं दूध नहीं खाना चाहता हूँ।"

आपके लिए यह कथन कितना महत्वपूर्ण है।

- अत्यंत महत्वपूर्ण
- बहुत महत्वपूर्ण
- मामूली रूप से महत्वपूर्ण
- थोड़ा महत्वपूर्ण

- कतई महत्वपूर्ण नहीं

आपके द्वारा चयनित "मैं दूध खाने से दूसरों के सामने पिछड़ा महसूस करता हूँ।"

आपके लिए यह कथन कितना महत्वपूर्ण है।

- अत्यंत महत्वपूर्ण
- बहुत महत्वपूर्ण
- मामूली रूप से महत्वपूर्ण
- थोड़ा महत्वपूर्ण
- कतई महत्वपूर्ण नहीं

आपके द्वारा चयनित "मेरी दूध खाने कि इच्छा नहीं होती है।"

आपके लिए यह कथन कितना महत्वपूर्ण है।

- अत्यंत महत्वपूर्ण
- बहुत महत्वपूर्ण
- मामूली रूप से महत्वपूर्ण
- थोड़ा महत्वपूर्ण
- कतई महत्वपूर्ण नहीं

आपके द्वारा चयनित "मेने हाल ही में दूध खाया है। और अब मैं बार-बार दूध नहीं खाना चाहता हूँ।"

आपके लिए यह कथन कितना महत्वपूर्ण है।

- अत्यंत महत्वपूर्ण

- बहुत महत्वपूर्ण
- मामूली रूप से महत्वपूर्ण
- थोड़ा महत्वपूर्ण
- कतई महत्वपूर्ण नहीं

आपके द्वारा चयनित "मैं कभी दूध नहीं खाऊंगा।क्योंकि मुझे हर दिन एक ही प्रकार का भोजन खाना पसंद है।"

आपके लिए यह कथन कितना महत्वपूर्ण है।

- अत्यंत महत्वपूर्ण
- बहुत महत्वपूर्ण
- मामूली रूप से महत्वपूर्ण
- थोड़ा महत्वपूर्ण
- कतई महत्वपूर्ण नहीं

आपके द्वारा चयनित "दूध खाना मूझे दुखी, अकेला और कुंठित महसूस कराता है।"

आपके लिए यह कथन कितना महत्वपूर्ण है।

- अत्यंत महत्वपूर्ण
- बहुत महत्वपूर्ण
- मामूली रूप से महत्वपूर्ण
- थोड़ा महत्वपूर्ण
- कतई महत्वपूर्ण नहीं

आपके द्वारा चयनित "क्योंकि दूध जैविक (आर्गेनिक) नहीं है।"

आपके लिए यह कथन कितना महत्वपूर्ण है।

- अत्यंत महत्वपूर्ण
- बहुत महत्वपूर्ण
- मामूली रूप से महत्वपूर्ण
- थोड़ा महत्वपूर्ण
- कतई महत्वपूर्ण नहीं

Reasons for Fruit Choices(RATA)

क्या आप केले खाते हैं।

- हाँ
- नहीं

किन कारणों के लिए आप केले खातें हैं। (सभी विकल्प चुने)

- क्योंकि केले कुछ स्थितियों से संबंधित है।
- क्योंकि मैं अकेला महसूस करता हूँ।
- क्योंकि केले में वसा कि मात्रा कम होती है।
- केले खाना मेरी परंपरा का हिस्सा है।
- क्योंकि केले मेरे लिए आकर्षक भोजन है।
- क्योंकि मैं केले हर दिन खाना चाहता हूँ।
- क्योंकि केले प्राकृतिक है।
- क्योंकि केले का स्वाद अच्छा है।
- क्योंकि मैं उदास रहता हूँ।
- क्योंकि मैं निराश रहता हूँ।
- क्योंकि केले खाना सामाजिक है।
- क्योंकि केले ही एकमात्र विकल्प है।

- ☐ क्योंकि केले बनाना सुविधाजनक है।
- ☐ क्योंकि मैं बचपन से केले खाता रहा हूँ।
- ☐ क्योंकि केले विक्रय बिक्री पर होता है।
- ☐ क्योंकि मैं केले को टीवी या विज्ञापन पर देखता हूँ।
- ☐ क्योंकि केले खाना मूझे दूसरों के सामने अच्छा दिखाता है।
- ☐ क्योंकि मेरे भीतर केले खाने कि भूख रहती है।
- ☐ क्योंकि मैं अपने वजन पर निगरानी रखता हूँ।
- ☐ क्योंकि मैं केले खाने में आनंद महसूस करता हूँ।
- ☐ क्योंकि मैं केले से परिचित हूँ।
- ☐ संतुलित आहार बनाए रखने के लिए।
- ☐ क्योंकि मुझे केले पसंद है।
- ☐ क्योंकि केले या केले से सम्बंदित भोज कम समय में बन जाता है।
- ☐ क्योंकि मूझे ऊर्जा की जरूरत होती है।
- ☐ क्योंकि केले जैविक होते है।
- ☐ क्योंकि मैं केले खाने का आदी हूँ।
- ☐ दुसरो को निराश करने से बचने के लिए।
- ☐ क्योंकि मैं आमतौर पर केले खाता हूँ।
- ☐ क्योंकि केले सस्ते हैं।
- ☐ क्योंकि केले सामाजिक समारोहों को और अधिक आरामदायक बनाता है।
- ☐ क्योंकि केले खाना मुझे तृप्त करता है।
- ☐ क्योंकि केले मेरे शरीर को आकार में रखता है।
- ☐ क्योंकि केले खाना सुखदेय है।
- ☐ मैं अपने आपको पुरस्कृत करने के लिए केले खाता हूँ।
- ☐ ताकि मैं अन्य लोगों के साथ समय बिता सकूं।
- ☐ क्योंकि केले कि प्रस्तुति आकर्षक होती है।
- ☐ क्योंकि केले में कोई हानिकारक (कीटनाशक, प्रदूषक, एंटीबायोटिक) पदार्थ नहीं होता है।
- ☐ क्योंकि मूझे केले खाने पड़ते हैं।
- ☐ क्योंकि मैं भूखा रहता था।
- ☐ क्योंकि केले सस्ते है।

- क्योंकि केले में कम कैलोरीज होती है।
- क्योंकि केले नहीं खाना अशिष्ट होता है।
- क्योंकि केले खाना स्वास्थ्य के लिए लाभदायक है।
- क्योंकि केले बनाना आसान है।
- क्योंकि केले दूसरों को पसंद है।
- क्योंकि केले खाना फैशनेबल है।

आपके द्वारा चयनित "क्योंकि मैं केले खाने का आदी हूँ।"

आपके लिए यह कथन कितना महत्वपूर्ण है।

- अत्यंत महत्वपूर्ण
- बहुत महत्वपूर्ण
- मामूली रूप से महत्वपूर्ण
- थोड़ा महत्वपूर्ण
- कतई महत्वपूर्ण नहीं

आपके द्वारा चयनित "क्योंकि केले खाना स्वास्थ्य के लिए लाभदायक है।"

आपके लिए यह कथन कितना महत्वपूर्ण है।

- अत्यंत महत्वपूर्ण
- बहुत महत्वपूर्ण
- मामूली रूप से महत्वपूर्ण
- थोड़ा महत्वपूर्ण
- कतई महत्वपूर्ण नहीं

आपके द्वारा चयनित "क्योंकि केले या केले से सम्बंदित भोज कम समय में बन जाता है।"

आपके लिए यह कथन कितना महत्वपूर्ण है।

- अत्यंत महत्वपूर्ण
- बहुत महत्वपूर्ण
- मामूली रूप से महत्वपूर्ण
- थोड़ा महत्वपूर्ण
- कतई महत्वपूर्ण नहीं

आपके द्वारा चयनित "क्योंकि केले का स्वाद अच्छा है।"

आपके लिए यह कथन कितना महत्वपूर्ण है।

- अत्यंत महत्वपूर्ण
- बहुत महत्वपूर्ण
- मामूली रूप से महत्वपूर्ण
- थोड़ा महत्वपूर्ण
- कतई महत्वपूर्ण नहीं

आपके द्वारा चयनित "क्योंकि केले खाना मुझे तृप्त करता है।"

आपके लिए यह कथन कितना महत्वपूर्ण है।

- अत्यंत महत्वपूर्ण
- बहुत महत्वपूर्ण
- मामूली रूप से महत्वपूर्ण
- थोड़ा महत्वपूर्ण
- कतई महत्वपूर्ण नहीं

आपके द्वारा चयनित "क्योंकि केले प्राकृतिक है।"

आपके लिए यह कथन कितना महत्वपूर्ण है।

- अत्यंत महत्वपूर्ण
- बहुत महत्वपूर्ण
- मामूली रूप से महत्वपूर्ण
- थोड़ा महत्वपूर्ण
- कतई महत्वपूर्ण नहीं

आपके द्वारा चयनित "क्योंकि केले सस्ते है।"

आपके लिए यह कथन कितना महत्वपूर्ण है।

- अत्यंत महत्वपूर्ण
- बहुत महत्वपूर्ण
- मामूली रूप से महत्वपूर्ण
- थोड़ा महत्वपूर्ण
- कतई महत्वपूर्ण नहीं

आपके द्वारा चयनित "क्योंकि केले में कम कैलोरीज होती है।"

आपके लिए यह कथन कितना महत्वपूर्ण है।

- अत्यंत महत्वपूर्ण
- बहुत महत्वपूर्ण
- मामूली रूप से महत्वपूर्ण

- थोड़ा महत्वपूर्ण
- कतई महत्वपूर्ण नहीं

आपके द्वारा चयनित "क्योंकि मैं निराश रहता हूँ।"

आपके लिए यह कथन कितना महत्वपूर्ण है।

- अत्यंत महत्वपूर्ण
- बहुत महत्वपूर्ण
- मामूली रूप से महत्वपूर्ण
- थोड़ा महत्वपूर्ण
- कतई महत्वपूर्ण नहीं

आपके द्वारा चयनित "क्योंकि केले खाना मूझे दूसरों के सामने अच्छा दिखाता है।"

आपके लिए यह कथन कितना महत्वपूर्ण है।

- अत्यंत महत्वपूर्ण
- बहुत महत्वपूर्ण
- मामूली रूप से महत्वपूर्ण
- थोड़ा महत्वपूर्ण
- कतई महत्वपूर्ण नहीं

आपके द्वारा चयनित "क्योंकि केले नहीं खाना अशिष्ट होता है।"

आपके लिए यह कथन कितना महत्वपूर्ण है।

- अत्यंत महत्वपूर्ण
- बहुत महत्वपूर्ण
- मामूली रूप से महत्वपूर्ण
- थोड़ा महत्वपूर्ण
- कतई महत्वपूर्ण नहीं

आपके द्वारा चयनित "क्योंकि केले कि प्रस्तुति आकर्षक होती है।"

आपके लिए यह कथन कितना महत्वपूर्ण है।

- अत्यंत महत्वपूर्ण
- बहुत महत्वपूर्ण
- मामूली रूप से महत्वपूर्ण
- थोड़ा महत्वपूर्ण
- कतई महत्वपूर्ण नहीं

आपके द्वारा चयनित "ताकि मैं अन्य लोगों के साथ समय बिता सकूं।"

आपके लिए यह कथन कितना महत्वपूर्ण है।

- अत्यंत महत्वपूर्ण
- बहुत महत्वपूर्ण
- मामूली रूप से महत्वपूर्ण
- थोड़ा महत्वपूर्ण
- कतई महत्वपूर्ण नहीं

आपके द्वारा चयनित "क्योंकि केले जैविक होते है।"

आपके लिए यह कथन कितना महत्वपूर्ण है।

- अत्यंत महत्वपूर्ण
- बहुत महत्वपूर्ण
- मामूली रूप से महत्वपूर्ण
- थोड़ा महत्वपूर्ण
- कतई महत्वपूर्ण नहीं

आपके द्वारा चयनित "क्योंकि मैं बचपन से केले खाता रहा हूँ।"

आपके लिए यह कथन कितना महत्वपूर्ण है।

- अत्यंत महत्वपूर्ण
- बहुत महत्वपूर्ण
- मामूली रूप से महत्वपूर्ण
- थोड़ा महत्वपूर्ण
- कतई महत्वपूर्ण नहीं

आपके द्वारा चयनित "क्योंकि मैं केले खाने में आनंद महसूस करता हूँ।"

आपके लिए यह कथन कितना महत्वपूर्ण है।

- अत्यंत महत्वपूर्ण
- बहुत महत्वपूर्ण
- मामूली रूप से महत्वपूर्ण
- थोड़ा महत्वपूर्ण
- कतई महत्वपूर्ण नहीं

आपके द्वारा चयनित "क्योंकि मैं भूखा रहता था।"

आपके लिए यह कथन कितना महत्वपूर्ण है।

- अत्यंत महत्वपूर्ण
- बहुत महत्वपूर्ण
- मामूली रूप से महत्वपूर्ण
- थोड़ा महत्वपूर्ण
- कतई महत्वपूर्ण नहीं

आपके द्वारा चयनित "क्योंकि मैं आमतौर पर केले खाता हूँ।"

आपके लिए यह कथन कितना महत्वपूर्ण है।

- अत्यंत महत्वपूर्ण
- बहुत महत्वपूर्ण
- मामूली रूप से महत्वपूर्ण
- थोड़ा महत्वपूर्ण
- कतई महत्वपूर्ण नहीं

आपके द्वारा चयनित "क्योंकि मेरे भीतर केले खाने कि भूख रहती है।"

आपके लिए यह कथन कितना महत्वपूर्ण है।

- अत्यंत महत्वपूर्ण
- बहुत महत्वपूर्ण
- मामूली रूप से महत्वपूर्ण
- थोड़ा महत्वपूर्ण

- कतई महत्वपूर्ण नहीं

आपके द्वारा चयनित "क्योंकि केले बनाना आसान है।"

आपके लिए यह कथन कितना महत्वपूर्ण है।

- अत्यंत महत्वपूर्ण
- बहुत महत्वपूर्ण
- मामूली रूप से महत्वपूर्ण
- थोड़ा महत्वपूर्ण
- कतई महत्वपूर्ण नहीं

आपके द्वारा चयनित "क्योंकि मैं उदास रहता हूँ।"

आपके लिए यह कथन कितना महत्वपूर्ण है।

- अत्यंत महत्वपूर्ण
- बहुत महत्वपूर्ण
- मामूली रूप से महत्वपूर्ण
- थोड़ा महत्वपूर्ण
- कतई महत्वपूर्ण नहीं

आपके द्वारा चयनित "क्योंकि केले में वसा कि मात्रा कम होती है।"

आपके लिए यह कथन कितना महत्वपूर्ण है।

- अत्यंत महत्वपूर्ण

- बहुत महत्वपूर्ण
- मामूली रूप से महत्वपूर्ण
- थोड़ा महत्वपूर्ण
- कतई महत्वपूर्ण नहीं

आपके द्वारा चयनित "क्योंकि मैं केले को टीवी या विज्ञापन पर देखता हूँ।"

आपके लिए यह कथन कितना महत्वपूर्ण है।

- अत्यंत महत्वपूर्ण
- बहुत महत्वपूर्ण
- मामूली रूप से महत्वपूर्ण
- थोड़ा महत्वपूर्ण
- कतई महत्वपूर्ण नहीं

आपके द्वारा चयनित "क्योंकि केले सामाजिक समारोहों को और अधिक आरामदायक बनाता है।"

आपके लिए यह कथन कितना महत्वपूर्ण है।

- अत्यंत महत्वपूर्ण
- बहुत महत्वपूर्ण
- मामूली रूप से महत्वपूर्ण
- थोड़ा महत्वपूर्ण
- कतई महत्वपूर्ण नहीं

आपके द्वारा चयनित "क्योंकि मूझे केले खाने पड़ते हैं।"

आपके लिए यह कथन कितना महत्वपूर्ण है।

- अत्यंत महत्वपूर्ण
- बहुत महत्वपूर्ण
- मामूली रूप से महत्वपूर्ण
- थोड़ा महत्वपूर्ण
- कतई महत्वपूर्ण नहीं

आपके द्वारा चयनित "क्योंकि केले दूसरों को पसंद है।"

आपके लिए यह कथन कितना महत्वपूर्ण है।

- अत्यंत महत्वपूर्ण
- बहुत महत्वपूर्ण
- मामूली रूप से महत्वपूर्ण
- थोड़ा महत्वपूर्ण
- कतई महत्वपूर्ण नहीं

आपके द्वारा चयनित "क्योंकि मैं अकेला महसूस करता हूँ।"

आपके लिए यह कथन कितना महत्वपूर्ण है।

- अत्यंत महत्वपूर्ण
- बहुत महत्वपूर्ण
- मामूली रूप से महत्वपूर्ण
- थोड़ा महत्वपूर्ण
- कतई महत्वपूर्ण नहीं

आपके द्वारा चयनित "क्योंकि मैं अपने वजन पर निगरानी रखता हूँ।"

आपके लिए यह कथन कितना महत्वपूर्ण है।

- अत्यंत महत्वपूर्ण
- बहुत महत्वपूर्ण
- मामूली रूप से महत्वपूर्ण
- थोड़ा महत्वपूर्ण
- कतई महत्वपूर्ण नहीं

आपके द्वारा चयनित "क्योंकि केले में कोई हानिकारक (कीटनाशक, प्रदूषक, एंटीबायोटिक) पदार्थ नहीं होता है।"

आपके लिए यह कथन कितना महत्वपूर्ण है।

- अत्यंत महत्वपूर्ण
- बहुत महत्वपूर्ण
- मामूली रूप से महत्वपूर्ण
- थोड़ा महत्वपूर्ण
- कतई महत्वपूर्ण नहीं

आपके द्वारा चयनित "क्योंकि केले बनाना सुविधाजनक है।"

आपके लिए यह कथन कितना महत्वपूर्ण है।

- अत्यंत महत्वपूर्ण
- बहुत महत्वपूर्ण
- मामूली रूप से महत्वपूर्ण

- थोड़ा महत्वपूर्ण
- कतई महत्वपूर्ण नहीं

आपके द्वारा चयनित "क्योंकि मूझे ऊर्जा की जरूरत होती है।"

आपके लिए यह कथन कितना महत्वपूर्ण है।

- अत्यंत महत्वपूर्ण
- बहुत महत्वपूर्ण
- मामूली रूप से महत्वपूर्ण
- थोड़ा महत्वपूर्ण
- कतई महत्वपूर्ण नहीं

आपके द्वारा चयनित "क्योंकि मैं केले से परिचित हूँ।"

आपके लिए यह कथन कितना महत्वपूर्ण है।

- अत्यंत महत्वपूर्ण
- बहुत महत्वपूर्ण
- मामूली रूप से महत्वपूर्ण
- थोड़ा महत्वपूर्ण
- कतई महत्वपूर्ण नहीं

आपके द्वारा चयनित "क्योंकि मुझे केले पसंद है।"

आपके लिए यह कथन कितना महत्वपूर्ण है।

- अत्यंत महत्वपूर्ण
- बहुत महत्वपूर्ण
- मामूली रूप से महत्वपूर्ण
- थोड़ा महत्वपूर्ण
- कतई महत्वपूर्ण नहीं

आपके द्वारा चयनित "क्योंकि केले खाना सुखदेय है।"

आपके लिए यह कथन कितना महत्वपूर्ण है।

- अत्यंत महत्वपूर्ण
- बहुत महत्वपूर्ण
- मामूली रूप से महत्वपूर्ण
- थोड़ा महत्वपूर्ण
- कतई महत्वपूर्ण नहीं

आपके द्वारा चयनित "मैं अपने आपको पुरस्कृत करने के लिए केले खाता हूँ।"

आपके लिए यह कथन कितना महत्वपूर्ण है।

- अत्यंत महत्वपूर्ण
- बहुत महत्वपूर्ण
- मामूली रूप से महत्वपूर्ण
- थोड़ा महत्वपूर्ण
- कतई महत्वपूर्ण नहीं

आपके द्वारा चयनित "केले खाना मेरी परंपरा का हिस्सा है।"

आपके लिए यह कथन कितना महत्वपूर्ण है।

- अत्यंत महत्वपूर्ण
- बहुत महत्वपूर्ण
- मामूली रूप से महत्वपूर्ण
- थोड़ा महत्वपूर्ण
- कतई महत्वपूर्ण नहीं

आपके द्वारा चयनित "क्योंकि केले कुछ स्थितियों से संबंधित है।"

आपके लिए यह कथन कितना महत्वपूर्ण है।

- अत्यंत महत्वपूर्ण
- बहुत महत्वपूर्ण
- मामूली रूप से महत्वपूर्ण
- थोड़ा महत्वपूर्ण
- कतई महत्वपूर्ण नहीं

आपके द्वारा चयनित "संतुलित आहार बनाए रखने के लिए।"

आपके लिए यह कथन कितना महत्वपूर्ण है।

- अत्यंत महत्वपूर्ण
- बहुत महत्वपूर्ण
- मामूली रूप से महत्वपूर्ण
- थोड़ा महत्वपूर्ण
- कतई महत्वपूर्ण नहीं

आपके द्वारा चयनित "क्योंकि केले खाना सामाजिक है।"

आपके लिए यह कथन कितना महत्वपूर्ण है।

- अत्यंत महत्वपूर्ण
- बहुत महत्वपूर्ण
- मामूली रूप से महत्वपूर्ण
- थोड़ा महत्वपूर्ण
- कतई महत्वपूर्ण नहीं

आपके द्वारा चयनित "क्योंकि केले सस्ते हैं।"

आपके लिए यह कथन कितना महत्वपूर्ण है।

- अत्यंत महत्वपूर्ण
- बहुत महत्वपूर्ण
- मामूली रूप से महत्वपूर्ण
- थोड़ा महत्वपूर्ण
- कतई महत्वपूर्ण नहीं

आपके द्वारा चयनित "क्योंकि केले मेरे लिए आकर्षक भोजन है।"

आपके लिए यह कथन कितना महत्वपूर्ण है।

- अत्यंत महत्वपूर्ण
- बहुत महत्वपूर्ण
- मामूली रूप से महत्वपूर्ण
- थोड़ा महत्वपूर्ण

- कतई महत्वपूर्ण नहीं

आपके द्वारा चयनित "दुसरो को निराश करने से बचने के लिए।"

आपके लिए यह कथन कितना महत्वपूर्ण है।

- अत्यंत महत्वपूर्ण
- बहुत महत्वपूर्ण
- मामूली रूप से महत्वपूर्ण
- थोड़ा महत्वपूर्ण
- कतई महत्वपूर्ण नहीं

आपके द्वारा चयनित "क्योंकि केले खाना फैशनेबल है।"

आपके लिए यह कथन कितना महत्वपूर्ण है।

- अत्यंत महत्वपूर्ण
- बहुत महत्वपूर्ण
- मामूली रूप से महत्वपूर्ण
- थोड़ा महत्वपूर्ण
- कतई महत्वपूर्ण नहीं

आपके द्वारा चयनित "क्योंकि केले विक्रय बिक्री पर होता है।"

आपके लिए यह कथन कितना महत्वपूर्ण है।

- अत्यंत महत्वपूर्ण

- बहुत महत्वपूर्ण
- मामूली रूप से महत्वपूर्ण
- थोड़ा महत्वपूर्ण
- कतई महत्वपूर्ण नहीं

आपके द्वारा चयनित "क्योंकि केले मेरे शरीर को आकार में रखता है।"

आपके लिए यह कथन कितना महत्वपूर्ण है।

- अत्यंत महत्वपूर्ण
- बहुत महत्वपूर्ण
- मामूली रूप से महत्वपूर्ण
- थोड़ा महत्वपूर्ण
- कतई महत्वपूर्ण नहीं

आपके द्वारा चयनित "क्योंकि केले ही एकमात्र विकल्प है।"

आपके लिए यह कथन कितना महत्वपूर्ण है।

- अत्यंत महत्वपूर्ण
- बहुत महत्वपूर्ण
- मामूली रूप से महत्वपूर्ण
- थोड़ा महत्वपूर्ण
- कतई महत्वपूर्ण नहीं

आपके द्वारा चयनित "क्योंकि मैं केले हर दिन खाना चाहता हूँ।"

आपके लिए यह कथन कितना महत्वपूर्ण है।

- अत्यंत महत्वपूर्ण
- बहुत महत्वपूर्ण
- मामूली रूप से महत्वपूर्ण
- थोड़ा महत्वपूर्ण
- कतई महत्वपूर्ण नहीं

कृपया केले नहीं खाने के सभी कारण बताएं। (सभी विकल्प चुने)

- मैं कभी केले नहीं खाऊंगा।क्योंकि मुझे हर दिन एक ही प्रकार का भोजन खाना पसंद है।
- केले मूझे दिखने में अच्छा नहीं लगता है।
- केले कि कीमत बहुत अधिक होती है।
- मैं आमतौर पर केले नहीं खाता हूँ।
- केले स्वास्थ्यय के लिए लाभदायक नहीं है।
- केले मेरी भूख को तृप्त नहीं कर पाता है।
- केले खाना मूझे दुखी, अकेला और कुंठित महसूस कराता है।
- केले मेरी भूख मिटाने के लिए पर्याप्त नहीं है।
- मेरी केले खाने कि इच्छा नहीं होती है।
- केले अन्य लोगों के आसपास खाने लायक भोजन नहीं है।
- मुझे केले पसंद नहीं है।
- केले स्थिति के लिए उपयुक्त नहीं था।
- मुझे नहीं लगता कि केले एक नाश्ता है।
- केले बनाना मुश्किल होता है।
- केले मे कैलोरी की मात्रा बहुत अधिक होती है।
- मेने हाल ही में केले खाय है। और अब मैं बार-बार केले नहीं खाना चाहता हूँ।
- मैं केले खाने से दूसरों के सामने पिछड़ा महसूस करता हूँ।
- क्योंकि केले जैविक (आर्गेनिक) नहीं है।
- मैं केले नहीं खाना चाहता हूँ।
- केले कि खुराक का आकार मेरे लिए उपयुक्त नहीं है।

आपके द्वारा चयनित "मुझे नहीं लगता कि केले एक नाश्ता है।"

आपके लिए यह कथन कितना महत्वपूर्ण है।

- अत्यंत महत्वपूर्ण
- बहुत महत्वपूर्ण
- मामूली रूप से महत्वपूर्ण
- थोड़ा महत्वपूर्ण
- कतई महत्वपूर्ण नहीं

आपके द्वारा चयनित "मुझे केले पसंद नहीं है।"

आपके लिए यह कथन कितना महत्वपूर्ण है।

- अत्यंत महत्वपूर्ण
- बहुत महत्वपूर्ण
- मामूली रूप से महत्वपूर्ण
- थोड़ा महत्वपूर्ण
- कतई महत्वपूर्ण नहीं

आपके द्वारा चयनित "मैं आमतौर पर केले नहीं खाता हूँ।"

आपके लिए यह कथन कितना महत्वपूर्ण है।

- अत्यंत महत्वपूर्ण
- बहुत महत्वपूर्ण
- मामूली रूप से महत्वपूर्ण

- थोड़ा महत्वपूर्ण
- कतई महत्वपूर्ण नहीं

आपके द्वारा चयनित "केले कि खुराक का आकार मेरे लिए उपयुक्त नहीं है।"

आपके लिए यह कथन कितना महत्वपूर्ण है।

- अत्यंत महत्वपूर्ण
- बहुत महत्वपूर्ण
- मामूली रूप से महत्वपूर्ण
- थोड़ा महत्वपूर्ण
- कतई महत्वपूर्ण नहीं

आपके द्वारा चयनित "केले मेरी भूख मिटाने के लिए पर्याप्त नहीं है।"

आपके लिए यह कथन कितना महत्वपूर्ण है।

- अत्यंत महत्वपूर्ण
- बहुत महत्वपूर्ण
- मामूली रूप से महत्वपूर्ण
- थोड़ा महत्वपूर्ण
- कतई महत्वपूर्ण नहीं

आपके द्वारा चयनित "केले स्वास्थ्यय के लिए लाभदायक नहीं है।"

आपके लिए यह कथन कितना महत्वपूर्ण है।

- अत्यंत महत्वपूर्ण
- बहुत महत्वपूर्ण
- मामूली रूप से महत्वपूर्ण
- थोड़ा महत्वपूर्ण
- कतई महत्वपूर्ण नहीं

आपके द्वारा चयनित "केले बनाना मुश्किल होता है।"

आपके लिए यह कथन कितना महत्वपूर्ण है।

- अत्यंत महत्वपूर्ण
- बहुत महत्वपूर्ण
- मामूली रूप से महत्वपूर्ण
- थोड़ा महत्वपूर्ण
- कतई महत्वपूर्ण नहीं

आपके द्वारा चयनित "केले मेरी भूख को तृप्त नहीं कर पाता है।"

आपके लिए यह कथन कितना महत्वपूर्ण है।

- अत्यंत महत्वपूर्ण
- बहुत महत्वपूर्ण
- मामूली रूप से महत्वपूर्ण
- थोड़ा महत्वपूर्ण
- कतई महत्वपूर्ण नहीं

आपके द्वारा चयनित "केले स्थिति के लिए उपयुक्त नहीं था।"

आपके लिए यह कथन कितना महत्वपूर्ण है।

- अत्यंत महत्वपूर्ण
- बहुत महत्वपूर्ण
- मामूली रूप से महत्वपूर्ण
- थोड़ा महत्वपूर्ण
- कतई महत्वपूर्ण नहीं

आपके द्वारा चयनित "केले अन्य लोगों के आसपास खाने लायक भोजन नहीं है।"

आपके लिए यह कथन कितना महत्वपूर्ण है।

- अत्यंत महत्वपूर्ण
- बहुत महत्वपूर्ण
- मामूली रूप से महत्वपूर्ण
- थोड़ा महत्वपूर्ण
- कतई महत्वपूर्ण नहीं

आपके द्वारा चयनित "केले कि कीमत बहुत अधिक होती है।"

आपके लिए यह कथन कितना महत्वपूर्ण है।

- अत्यंत महत्वपूर्ण
- बहुत महत्वपूर्ण
- मामूली रूप से महत्वपूर्ण
- थोड़ा महत्वपूर्ण
- कतई महत्वपूर्ण नहीं

आपके द्वारा चयनित "केले मूझे दिखने में अच्छा नहीं लगता है।"

आपके लिए यह कथन कितना महत्वपूर्ण है।

- अत्यंत महत्वपूर्ण
- बहुत महत्वपूर्ण
- मामूली रूप से महत्वपूर्ण
- थोड़ा महत्वपूर्ण
- कतई महत्वपूर्ण नहीं

आपके द्वारा चयनित "केले मे कैलोरी की मात्रा बहुत अधिक होती है।"

आपके लिए यह कथन कितना महत्वपूर्ण है।

- अत्यंत महत्वपूर्ण
- बहुत महत्वपूर्ण
- मामूली रूप से महत्वपूर्ण
- थोड़ा महत्वपूर्ण
- कतई महत्वपूर्ण नहीं

आपके द्वारा चयनित "मैं केले नहीं खाना चाहता हूँ।"

आपके लिए यह कथन कितना महत्वपूर्ण है।

- अत्यंत महत्वपूर्ण
- बहुत महत्वपूर्ण
- मामूली रूप से महत्वपूर्ण
- थोड़ा महत्वपूर्ण

- कतई महत्वपूर्ण नहीं

आपके द्वारा चयनित "मैं केले खाने से दूसरों के सामने पिछड़ा महसूस करता हूँ।"

आपके लिए यह कथन कितना महत्वपूर्ण है।

- अत्यंत महत्वपूर्ण
- बहुत महत्वपूर्ण
- मामूली रूप से महत्वपूर्ण
- थोड़ा महत्वपूर्ण
- कतई महत्वपूर्ण नहीं

आपके द्वारा चयनित "मेरी केले खाने कि इच्छा नहीं होती है।"

आपके लिए यह कथन कितना महत्वपूर्ण है।

- अत्यंत महत्वपूर्ण
- बहुत महत्वपूर्ण
- मामूली रूप से महत्वपूर्ण
- थोड़ा महत्वपूर्ण
- कतई महत्वपूर्ण नहीं

आपके द्वारा चयनित "मेने हाल ही में केले खाय है। और अब मैं बार-बार केले नहीं खाना चाहता हूँ।"

आपके लिए यह कथन कितना महत्वपूर्ण है।

- अत्यंत महत्वपूर्ण

- बहुत महत्वपूर्ण
- मामूली रूप से महत्वपूर्ण
- थोड़ा महत्वपूर्ण
- कतई महत्वपूर्ण नहीं

आपके द्वारा चयनित "मैं कभी केले नहीं खाऊंगा।क्योंकि मुझे हर दिन एक ही प्रकार का भोजन खाना पसंद है।"

आपके लिए यह कथन कितना महत्वपूर्ण है।

- अत्यंत महत्वपूर्ण
- बहुत महत्वपूर्ण
- मामूली रूप से महत्वपूर्ण
- थोड़ा महत्वपूर्ण
- कतई महत्वपूर्ण नहीं

आपके द्वारा चयनित "केले खाना मूझे दुखी, अकेला और कुंठित महसूस कराता है।"

आपके लिए यह कथन कितना महत्वपूर्ण है।

- अत्यंत महत्वपूर्ण
- बहुत महत्वपूर्ण
- मामूली रूप से महत्वपूर्ण
- थोड़ा महत्वपूर्ण
- कतई महत्वपूर्ण नहीं

आपके द्वारा चयनित "क्योंकि केले जैविक (आर्गेनिक) नहीं है।"

आपके लिए यह कथन कितना महत्वपूर्ण है।

- अत्यंत महत्वपूर्ण
- बहुत महत्वपूर्ण
- मामूली रूप से महत्वपूर्ण
- थोड़ा महत्वपूर्ण
- कतई महत्वपूर्ण नहीं

Reasons for Sweet food Choices(RATA)

क्या आप गुलाब जामुन खाते हैं।

- हाँ
- नहीं

किन कारणों के लिए आप गुलाब जामुन खातें हैं। (सभी विकल्प चुने)

- क्योंकि मैं भूखा रहता था।
- क्योंकि गुलाब जामुन कि प्रस्तुति आकर्षक होती है।
- क्योंकि गुलाब जामुन बनाना आसान है।
- क्योंकि मैं गुलाब जामुन से परिचित हूँ।
- क्योंकि मैं निराश रहता हूँ।
- क्योंकि गुलाब जामुन खाना मूझे दूसरों के सामने अच्छा दिखाता है।
- क्योंकि मैं गुलाब जामुन को टीवी या विज्ञापन पर देखता हूँ।
- क्योंकि मेरे भीतर गुलाब जामुन खाने कि भूख रहती है।
- मैं अपने आपको पुरस्कृत करने के लिए गुलाब जामुन खाता हूँ।
- क्योंकि गुलाब जामुन सामाजिक समारोहों को और अधिक आरामदायक बनाता है।
- गुलाब जामुन खाना मेरी परंपरा का हिस्सा है।
- क्योंकि गुलाब जामुन खाना स्वास्थ्य के लिए लाभदायक है।

- ☐ क्योंकि गुलाब जामुन कुछ स्थितियों से संबंधित है।
- ☐ क्योंकि मैं आमतौर पर गुलाब जामुन खाता हूँ।
- ☐ क्योंकि गुलाब जामुन सस्ते हैं।
- ☐ क्योंकि गुलाब जामुन में कोई हानिकारक (कीटनाशक, प्रदूषक, एंटीबायोटिक) पदार्थ नहीं होता है।
- ☐ क्योंकि गुलाब जामुन में वसा कि मात्रा कम होती है।
- ☐ क्योंकि गुलाब जामुन मेरे शरीर को आकार में रखता है।
- ☐ क्योंकि गुलाब जामुन जैविक होते है।
- ☐ क्योंकि मैं अपने वजन पर निगरानी रखता हूँ।
- ☐ क्योंकि गुलाब जामुन में कम कैलोरीज होती है।
- ☐ क्योंकि गुलाब जामुन ही एकमात्र विकल्प है।
- ☐ क्योंकि गुलाब जामुन नहीं खाना अशिष्ट होता है।
- ☐ क्योंकि मूझे ऊर्जा की जरूरत होती है।
- ☐ संतुलित आहार बनाए रखने के लिए।
- ☐ क्योंकि मैं गुलाब जामुन खाने में आनंद महसूस करता हूँ।
- ☐ क्योंकि गुलाब जामुन खाना सुखदेय है।
- ☐ क्योंकि गुलाब जामुन का स्वाद अच्छा है।
- ☐ क्योंकि गुलाब जामुन दूसरों को पसंद है।
- ☐ क्योंकि गुलाब जामुन मेरे लिए आकर्षक भोजन है।
- ☐ दुसरो को निराश करने से बचने के लिए।
- ☐ क्योंकि मैं उदास रहता हूँ।
- ☐ क्योंकि मूझे गुलाब जामुन खाने पड़ते हैं।
- ☐ क्योंकि गुलाब जामुन प्राकृतिक है।
- ☐ क्योंकि गुलाब जामुन खाना सामाजिक है।
- ☐ क्योंकि मैं गुलाब जामुन खाने का आदी हूँ।
- ☐ क्योंकि मैं अकेला महसूस करता हूँ।
- ☐ क्योंकि गुलाब जामुन बनाना सुविधाजनक है।
- ☐ क्योंकि गुलाब जामुन खाना फैशनेबल है।
- ☐ क्योंकि गुलाब जामुन खाना मुझे तृप्त करता है।
- ☐ क्योंकि मैं गुलाब जामुन हर दिन खाना चाहता हूँ।

- ☐ क्योंकि गुलाब जामुन कम समय में बन जाता है।
- ☐ क्योंकि गुलाब जामुन सस्ते है।
- ☐ ताकि मैं अन्य लोगों के साथ समय बिता सकूं।
- ☐ क्योंकि मैं बचपन से गुलाब जामुन खाता रहा हूँ।
- ☐ क्योंकि मुझे गुलाब जामुन पसंद है।
- ☐ क्योंकि गुलाब जामुन विक्रय बिक्री पर होता है।

आपके द्वारा चयनित "क्योंकि मैं गुलाब जामुन खाने का आदी हूँ।"

आपके लिए यह कथन कितना महत्वपूर्ण है।

- ○ अत्यंत महत्वपूर्ण
- ○ बहुत महत्वपूर्ण
- ○ मामूली रूप से महत्वपूर्ण
- ○ थोड़ा महत्वपूर्ण
- ○ कतई महत्वपूर्ण नहीं

आपके द्वारा चयनित "क्योंकि गुलाब जामुन खाना स्वास्थ्य के लिए लाभदायक है।"

आपके लिए यह कथन कितना महत्वपूर्ण है।

- ○ अत्यंत महत्वपूर्ण
- ○ बहुत महत्वपूर्ण
- ○ मामूली रूप से महत्वपूर्ण
- ○ थोड़ा महत्वपूर्ण
- ○ कतई महत्वपूर्ण नहीं

आपके द्वारा चयनित "क्योंकि गुलाब जामुन कम समय में बन जाता है।"

आपके लिए यह कथन कितना महत्वपूर्ण है।

- अत्यंत महत्वपूर्ण
- बहुत महत्वपूर्ण
- मामूली रूप से महत्वपूर्ण
- थोड़ा महत्वपूर्ण
- कतई महत्वपूर्ण नहीं

आपके द्वारा चयनित "क्योंकि गुलाब जामुन का स्वाद अच्छा है।"

आपके लिए यह कथन कितना महत्वपूर्ण है।

- अत्यंत महत्वपूर्ण
- बहुत महत्वपूर्ण
- मामूली रूप से महत्वपूर्ण
- थोड़ा महत्वपूर्ण
- कतई महत्वपूर्ण नहीं

आपके द्वारा चयनित "क्योंकि गुलाब जामुन खाना मुझे तृप्त करता है।"

आपके लिए यह कथन कितना महत्वपूर्ण है।

- अत्यंत महत्वपूर्ण
- बहुत महत्वपूर्ण
- मामूली रूप से महत्वपूर्ण
- थोड़ा महत्वपूर्ण
- कतई महत्वपूर्ण नहीं

आपके द्वारा चयनित "क्योंकि गुलाब जामुन प्राकृतिक है।"

आपके लिए यह कथन कितना महत्वपूर्ण है।

- अत्यंत महत्वपूर्ण
- बहुत महत्वपूर्ण
- मामूली रूप से महत्वपूर्ण
- थोड़ा महत्वपूर्ण
- कतई महत्वपूर्ण नहीं

आपके द्वारा चयनित "क्योंकि गुलाब जामुन सस्ते है।"

आपके लिए यह कथन कितना महत्वपूर्ण है।

- अत्यंत महत्वपूर्ण
- बहुत महत्वपूर्ण
- मामूली रूप से महत्वपूर्ण
- थोड़ा महत्वपूर्ण
- कतई महत्वपूर्ण नहीं

आपके द्वारा चयनित "क्योंकि गुलाब जामुन में कम कैलोरीज होती है।"

आपके लिए यह कथन कितना महत्वपूर्ण है।

- अत्यंत महत्वपूर्ण
- बहुत महत्वपूर्ण
- मामूली रूप से महत्वपूर्ण

- थोड़ा महत्वपूर्ण
- कतई महत्वपूर्ण नहीं

आपके द्वारा चयनित "क्योंकि मैं निराश रहता हूँ।"

आपके लिए यह कथन कितना महत्वपूर्ण है।

- अत्यंत महत्वपूर्ण
- बहुत महत्वपूर्ण
- मामूली रूप से महत्वपूर्ण
- थोड़ा महत्वपूर्ण
- कतई महत्वपूर्ण नहीं

आपके द्वारा चयनित "क्योंकि गुलाब जामुन खाना मूझे दूसरों के सामने अच्छा दिखाता है।"

आपके लिए यह कथन कितना महत्वपूर्ण है।

- अत्यंत महत्वपूर्ण
- बहुत महत्वपूर्ण
- मामूली रूप से महत्वपूर्ण
- थोड़ा महत्वपूर्ण
- कतई महत्वपूर्ण नहीं

आपके द्वारा चयनित "क्योंकि गुलाब जामुन नहीं खाना अशिष्ट होता है।"

आपके लिए यह कथन कितना महत्वपूर्ण है।

- अत्यंत महत्वपूर्ण
- बहुत महत्वपूर्ण
- मामूली रूप से महत्वपूर्ण
- थोड़ा महत्वपूर्ण
- कतई महत्वपूर्ण नहीं

आपके द्वारा चयनित "क्योंकि गुलाब जामुन कि प्रस्तुति आकर्षक होती है।"

आपके लिए यह कथन कितना महत्वपूर्ण है।

- अत्यंत महत्वपूर्ण
- बहुत महत्वपूर्ण
- मामूली रूप से महत्वपूर्ण
- थोड़ा महत्वपूर्ण
- कतई महत्वपूर्ण नहीं

आपके द्वारा चयनित "ताकि मैं अन्य लोगों के साथ समय बिता सकूं।"

आपके लिए यह कथन कितना महत्वपूर्ण है।

- अत्यंत महत्वपूर्ण
- बहुत महत्वपूर्ण
- मामूली रूप से महत्वपूर्ण
- थोड़ा महत्वपूर्ण
- कतई महत्वपूर्ण नहीं

आपके द्वारा चयनित "क्योंकि गुलाब जामुन जैविक होते है।"

आपके लिए यह कथन कितना महत्वपूर्ण है।

- अत्यंत महत्वपूर्ण
- बहुत महत्वपूर्ण
- मामूली रूप से महत्वपूर्ण
- थोड़ा महत्वपूर्ण
- कतई महत्वपूर्ण नहीं

आपके द्वारा चयनित "क्योंकि मैं बचपन से गुलाब जामुन खाता रहा हूँ।"

आपके लिए यह कथन कितना महत्वपूर्ण है।

- अत्यंत महत्वपूर्ण
- बहुत महत्वपूर्ण
- मामूली रूप से महत्वपूर्ण
- थोड़ा महत्वपूर्ण
- कतई महत्वपूर्ण नहीं

आपके द्वारा चयनित "क्योंकि मैं गुलाब जामुन खाने में आनंद महसूस करता हूँ।"

आपके लिए यह कथन कितना महत्वपूर्ण है।

- अत्यंत महत्वपूर्ण
- बहुत महत्वपूर्ण
- मामूली रूप से महत्वपूर्ण
- थोड़ा महत्वपूर्ण
- कतई महत्वपूर्ण नहीं

आपके द्वारा चयनित "क्योंकि मैं भूखा रहता था।"

आपके लिए यह कथन कितना महत्वपूर्ण है।

- अत्यंत महत्वपूर्ण
- बहुत महत्वपूर्ण
- मामूली रूप से महत्वपूर्ण
- थोड़ा महत्वपूर्ण
- कतई महत्वपूर्ण नहीं

आपके द्वारा चयनित "क्योंकि मैं आमतौर पर गुलाब जामुन खाता हूँ।"

आपके लिए यह कथन कितना महत्वपूर्ण है।

- अत्यंत महत्वपूर्ण
- बहुत महत्वपूर्ण
- मामूली रूप से महत्वपूर्ण
- थोड़ा महत्वपूर्ण
- कतई महत्वपूर्ण नहीं

आपके द्वारा चयनित "क्योंकि मेरे भीतर गुलाब जामुन खाने कि भूख रहती है।"

आपके लिए यह कथन कितना महत्वपूर्ण है।

- अत्यंत महत्वपूर्ण
- बहुत महत्वपूर्ण
- मामूली रूप से महत्वपूर्ण
- थोड़ा महत्वपूर्ण

- कतई महत्वपूर्ण नहीं

आपके द्वारा चयनित "क्योंकि गुलाब जामुन बनाना आसान है।"

आपके लिए यह कथन कितना महत्वपूर्ण है।

- अत्यंत महत्वपूर्ण
- बहुत महत्वपूर्ण
- मामूली रूप से महत्वपूर्ण
- थोड़ा महत्वपूर्ण
- कतई महत्वपूर्ण नहीं

आपके द्वारा चयनित "क्योंकि मैं उदास रहता हूँ।"

आपके लिए यह कथन कितना महत्वपूर्ण है।

- अत्यंत महत्वपूर्ण
- बहुत महत्वपूर्ण
- मामूली रूप से महत्वपूर्ण
- थोड़ा महत्वपूर्ण
- कतई महत्वपूर्ण नहीं

आपके द्वारा चयनित "क्योंकि गुलाब जामुन में वसा कि मात्रा कम होती है।"

आपके लिए यह कथन कितना महत्वपूर्ण है।

- अत्यंत महत्वपूर्ण

- बहुत महत्वपूर्ण
- मामूली रूप से महत्वपूर्ण
- थोड़ा महत्वपूर्ण
- कतई महत्वपूर्ण नहीं

आपके द्वारा चयनित "क्योंकि मैं गुलाब जामुन को टीवी या विज्ञापन पर देखता हूँ।"

आपके लिए यह कथन कितना महत्वपूर्ण है।

- अत्यंत महत्वपूर्ण
- बहुत महत्वपूर्ण
- मामूली रूप से महत्वपूर्ण
- थोड़ा महत्वपूर्ण
- कतई महत्वपूर्ण नहीं

आपके द्वारा चयनित "क्योंकि गुलाब जामुन सामाजिक समारोहों को और अधिक आरामदायक बनाता है।"

आपके लिए यह कथन कितना महत्वपूर्ण है।

- अत्यंत महत्वपूर्ण
- बहुत महत्वपूर्ण
- मामूली रूप से महत्वपूर्ण
- थोड़ा महत्वपूर्ण
- कतई महत्वपूर्ण नहीं

आपके द्वारा चयनित "क्योंकि मूझे गुलाब जामुन खाने पड़ते हैं।"

आपके लिए यह कथन कितना महत्वपूर्ण है।

- अत्यंत महत्वपूर्ण
- बहुत महत्वपूर्ण
- मामूली रूप से महत्वपूर्ण
- थोड़ा महत्वपूर्ण
- कतई महत्वपूर्ण नहीं

आपके द्वारा चयनित "क्योंकि गुलाब जामुन दूसरों को पसंद है।"

आपके लिए यह कथन कितना महत्वपूर्ण है।

- अत्यंत महत्वपूर्ण
- बहुत महत्वपूर्ण
- मामूली रूप से महत्वपूर्ण
- थोड़ा महत्वपूर्ण
- कतई महत्वपूर्ण नहीं

आपके द्वारा चयनित "क्योंकि मैं अकेला महसूस करता हूँ।"

आपके लिए यह कथन कितना महत्वपूर्ण है।

- अत्यंत महत्वपूर्ण
- बहुत महत्वपूर्ण
- मामूली रूप से महत्वपूर्ण
- थोड़ा महत्वपूर्ण
- कतई महत्वपूर्ण नहीं

आपके द्वारा चयनित "क्योंकि मैं अपने वजन पर निगरानी रखता हूँ।"

आपके लिए यह कथन कितना महत्वपूर्ण है।

- अत्यंत महत्वपूर्ण
- बहुत महत्वपूर्ण
- मामूली रूप से महत्वपूर्ण
- थोड़ा महत्वपूर्ण
- कतई महत्वपूर्ण नहीं

आपके द्वारा चयनित "क्योंकि गुलाब जामुन में कोई हानिकारक (कीटनाशक, प्रदूषक, एंटीबायोटिक) पदार्थ नहीं होता है।"

आपके लिए यह कथन कितना महत्वपूर्ण है।

- अत्यंत महत्वपूर्ण
- बहुत महत्वपूर्ण
- मामूली रूप से महत्वपूर्ण
- थोड़ा महत्वपूर्ण
- कतई महत्वपूर्ण नहीं

आपके द्वारा चयनित "क्योंकि गुलाब जामुन बनाना सुविधाजनक है।"

आपके लिए यह कथन कितना महत्वपूर्ण है।

- अत्यंत महत्वपूर्ण
- बहुत महत्वपूर्ण
- मामूली रूप से महत्वपूर्ण

- थोड़ा महत्वपूर्ण
- कतई महत्वपूर्ण नहीं

आपके द्वारा चयनित "क्योंकि मूझे ऊर्जा की जरूरत होती है।"

आपके लिए यह कथन कितना महत्वपूर्ण है।

- अत्यंत महत्वपूर्ण
- बहुत महत्वपूर्ण
- मामूली रूप से महत्वपूर्ण
- थोड़ा महत्वपूर्ण
- कतई महत्वपूर्ण नहीं

आपके द्वारा चयनित "क्योंकि मैं गुलाब जामुन से परिचित हूँ।"

आपके लिए यह कथन कितना महत्वपूर्ण है।

- अत्यंत महत्वपूर्ण
- बहुत महत्वपूर्ण
- मामूली रूप से महत्वपूर्ण
- थोड़ा महत्वपूर्ण
- कतई महत्वपूर्ण नहीं

आपके द्वारा चयनित "क्योंकि मुझे गुलाब जामुन पसंद है।"

आपके लिए यह कथन कितना महत्वपूर्ण है।

- अत्यंत महत्वपूर्ण
- बहुत महत्वपूर्ण
- मामूली रूप से महत्वपूर्ण
- थोड़ा महत्वपूर्ण
- कतई महत्वपूर्ण नहीं

आपके द्वारा चयनित "क्योंकि गुलाब जामुन खाना सुखदेय है।"

आपके लिए यह कथन कितना महत्वपूर्ण है।

- अत्यंत महत्वपूर्ण
- बहुत महत्वपूर्ण
- मामूली रूप से महत्वपूर्ण
- थोड़ा महत्वपूर्ण
- कतई महत्वपूर्ण नहीं

आपके द्वारा चयनित "मैं अपने आपको पुरस्कृत करने के लिए गुलाब जामुन खाता हूँ।"

आपके लिए यह कथन कितना महत्वपूर्ण है।

- अत्यंत महत्वपूर्ण
- बहुत महत्वपूर्ण
- मामूली रूप से महत्वपूर्ण
- थोड़ा महत्वपूर्ण
- कतई महत्वपूर्ण नहीं

आपके द्वारा चयनित "गुलाब जामुन खाना मेरी परंपरा का हिस्सा है।"

आपके लिए यह कथन कितना महत्वपूर्ण है।

- अत्यंत महत्वपूर्ण
- बहुत महत्वपूर्ण
- मामूली रूप से महत्वपूर्ण
- थोड़ा महत्वपूर्ण
- कतई महत्वपूर्ण नहीं

आपके द्वारा चयनित "क्योंकि गुलाब जामुन कुछ स्थितियों से संबंधित है।"

आपके लिए यह कथन कितना महत्वपूर्ण है।

- अत्यंत महत्वपूर्ण
- बहुत महत्वपूर्ण
- मामूली रूप से महत्वपूर्ण
- थोड़ा महत्वपूर्ण
- कतई महत्वपूर्ण नहीं

आपके द्वारा चयनित "संतुलित आहार बनाए रखने के लिए।"

आपके लिए यह कथन कितना महत्वपूर्ण है।

- अत्यंत महत्वपूर्ण
- बहुत महत्वपूर्ण
- मामूली रूप से महत्वपूर्ण
- थोड़ा महत्वपूर्ण
- कतई महत्वपूर्ण नहीं

आपके द्वारा चयनित "क्योंकि गुलाब जामुन खाना सामाजिक है।"

आपके लिए यह कथन कितना महत्वपूर्ण है।

- अत्यंत महत्वपूर्ण
- बहुत महत्वपूर्ण
- मामूली रूप से महत्वपूर्ण
- थोड़ा महत्वपूर्ण
- कतई महत्वपूर्ण नहीं

आपके द्वारा चयनित "क्योंकि गुलाब जामुन सस्ते हैं।"

आपके लिए यह कथन कितना महत्वपूर्ण है।

- अत्यंत महत्वपूर्ण
- बहुत महत्वपूर्ण
- मामूली रूप से महत्वपूर्ण
- थोड़ा महत्वपूर्ण
- कतई महत्वपूर्ण नहीं

आपके द्वारा चयनित "क्योंकि गुलाब जामुन मेरे लिए आकर्षक भोजन है।"

आपके लिए यह कथन कितना महत्वपूर्ण है।

- अत्यंत महत्वपूर्ण
- बहुत महत्वपूर्ण
- मामूली रूप से महत्वपूर्ण
- थोड़ा महत्वपूर्ण

- कतई महत्वपूर्ण नहीं

आपके द्वारा चयनित "दुसरो को निराश करने से बचने के लिए।"

आपके लिए यह कथन कितना महत्वपूर्ण है।

- अत्यंत महत्वपूर्ण
- बहुत महत्वपूर्ण
- मामूली रूप से महत्वपूर्ण
- थोड़ा महत्वपूर्ण
- कतई महत्वपूर्ण नहीं

आपके द्वारा चयनित "क्योंकि गुलाब जामुन खाना फैशनेबल है।"

आपके लिए यह कथन कितना महत्वपूर्ण है।

- अत्यंत महत्वपूर्ण
- बहुत महत्वपूर्ण
- मामूली रूप से महत्वपूर्ण
- थोड़ा महत्वपूर्ण
- कतई महत्वपूर्ण नहीं

आपके द्वारा चयनित "क्योंकि गुलाब जामुन विक्रय बिक्री पर होता है।"

आपके लिए यह कथन कितना महत्वपूर्ण है।

- अत्यंत महत्वपूर्ण

- बहुत महत्वपूर्ण
- मामूली रूप से महत्वपूर्ण
- थोड़ा महत्वपूर्ण
- कतई महत्वपूर्ण नहीं

आपके द्वारा चयनित "क्योंकि गुलाब जामुन मेरे शरीर को आकार में रखता है।"

आपके लिए यह कथन कितना महत्वपूर्ण है।

- अत्यंत महत्वपूर्ण
- बहुत महत्वपूर्ण
- मामूली रूप से महत्वपूर्ण
- थोड़ा महत्वपूर्ण
- कतई महत्वपूर्ण नहीं

आपके द्वारा चयनित "क्योंकि गुलाब जामुन ही एकमात्र विकल्प है।"

आपके लिए यह कथन कितना महत्वपूर्ण है।

- अत्यंत महत्वपूर्ण
- बहुत महत्वपूर्ण
- मामूली रूप से महत्वपूर्ण
- थोड़ा महत्वपूर्ण
- कतई महत्वपूर्ण नहीं

आपके द्वारा चयनित "क्योंकि मैं गुलाब जामुन हर दिन खाना चाहता हूँ।"

आपके लिए यह कथन कितना महत्वपूर्ण है।

- अत्यंत महत्वपूर्ण
- बहुत महत्वपूर्ण
- मामूली रूप से महत्वपूर्ण
- थोड़ा महत्वपूर्ण
- कतई महत्वपूर्ण नहीं

कृपया गुलाब जामुन नहीं खाने के सभी कारण बताएं। (सभी विकल्प चुने)

- मुझे गुलाब जामुन पसंद नहीं है।
- मुझे नहीं लगता कि गुलाब जामुन एक नाश्ता है।
- गुलाब जामुन मेरी भूख मिटाने के लिए पर्याप्त नहीं है।
- गुलाब जामुन बनाना मुश्किल होता है।
- गुलाब जामुन स्थिति के लिए उपयुक्त नहीं था।
- मेने हाल ही में गुलाब जामुन खाय है। और अब मैं बार-बार गुलाब जामुन नहीं खाना चाहता हूँ।
- मैं गुलाब जामुन नहीं खाना चाहता हूँ।
- मेरी गुलाब जामुन खाने कि इच्छा नहीं होती है।
- गुलाब जामुन खाना मूझे दुखी, अकेला और कुंठित महसूस कराता है।
- गुलाब जामुन कि कीमत बहुत अधिक होती है।
- क्योंकि गुलाब जामुन जैविक (आर्गेनिक) नहीं है।
- गुलाब जामुन कि खुराक का आकार मेरे लिए उपयुक्त नहीं है।
- मैं गुलाब जामुन खाने से दूसरों के सामने पिछड़ा महसूस करता हूँ।
- गुलाब जामुन स्वास्थ्यय के लिए लाभदायक नहीं है।
- मैं आमतौर पर गुलाब जामुन नहीं खाता हूँ।
- गुलाब जामुन अन्य लोगों के आसपास खाने लायक भोजन नहीं है।
- मैं कभी गुलाब जामुननहीं खाऊंगा।क्योंकि मुझे हर दिन एक ही प्रकार का भोजन खाना पसंद है।
- गुलाब जामुन मेरी भूख को तृप्त नहीं कर पाता है।
- गुलाब जामुन मूझे दिखने में अच्छा नहीं लगता है।
- गुलाब जामुन मे कैलोरी की मात्रा बहुत अधिक होती है।

आपके द्वारा चयनित "मुझे नहीं लगता कि गुलाब जामुन एक नाश्ता है।"

आपके लिए यह कथन कितना महत्वपूर्ण है।

- अत्यंत महत्वपूर्ण
- बहुत महत्वपूर्ण
- मामूली रूप से महत्वपूर्ण
- थोड़ा महत्वपूर्ण
- कतई महत्वपूर्ण नहीं

आपके द्वारा चयनित "मुझे गुलाब जामुन पसंद नहीं है।"

आपके लिए यह कथन कितना महत्वपूर्ण है।

- अत्यंत महत्वपूर्ण
- बहुत महत्वपूर्ण
- मामूली रूप से महत्वपूर्ण
- थोड़ा महत्वपूर्ण
- कतई महत्वपूर्ण नहीं

आपके द्वारा चयनित "मैं आमतौर पर गुलाब जामुन नहीं खाता हूँ।"

आपके लिए यह कथन कितना महत्वपूर्ण है।

- अत्यंत महत्वपूर्ण
- बहुत महत्वपूर्ण
- मामूली रूप से महत्वपूर्ण

- थोड़ा महत्वपूर्ण
- कतई महत्वपूर्ण नहीं

आपके द्वारा चयनित "गुलाब जामुन कि खुराक का आकार मेरे लिए उपयुक्त नहीं है।"

आपके लिए यह कथन कितना महत्वपूर्ण है।

- अत्यंत महत्वपूर्ण
- बहुत महत्वपूर्ण
- मामूली रूप से महत्वपूर्ण
- थोड़ा महत्वपूर्ण
- कतई महत्वपूर्ण नहीं

आपके द्वारा चयनित "गुलाब जामुन मेरी भूख मिटाने के लिए पर्याप्त नहीं है।"

आपके लिए यह कथन कितना महत्वपूर्ण है।

- अत्यंत महत्वपूर्ण
- बहुत महत्वपूर्ण
- मामूली रूप से महत्वपूर्ण
- थोड़ा महत्वपूर्ण
- कतई महत्वपूर्ण नहीं

आपके द्वारा चयनित "गुलाब जामुन स्वास्थ्यय के लिए लाभदायक नहीं है।"

आपके लिए यह कथन कितना महत्वपूर्ण है।

- अत्यंत महत्वपूर्ण
- बहुत महत्वपूर्ण
- मामूली रूप से महत्वपूर्ण
- थोड़ा महत्वपूर्ण
- कतई महत्वपूर्ण नहीं

आपके द्वारा चयनित "गुलाब जामुन बनाना मुश्किल होता है।"

आपके लिए यह कथन कितना महत्वपूर्ण है।

- अत्यंत महत्वपूर्ण
- बहुत महत्वपूर्ण
- मामूली रूप से महत्वपूर्ण
- थोड़ा महत्वपूर्ण
- कतई महत्वपूर्ण नहीं

आपके द्वारा चयनित "गुलाब जामुन मेरी भूख को तृप्त नहीं कर पाता है।"

आपके लिए यह कथन कितना महत्वपूर्ण है।

- अत्यंत महत्वपूर्ण
- बहुत महत्वपूर्ण
- मामूली रूप से महत्वपूर्ण
- थोड़ा महत्वपूर्ण
- कतई महत्वपूर्ण नहीं

आपके द्वारा चयनित "गुलाब जामुन स्थिति के लिए उपयुक्त नहीं था।"

आपके लिए यह कथन कितना महत्वपूर्ण है।

- अत्यंत महत्वपूर्ण
- बहुत महत्वपूर्ण
- मामूली रूप से महत्वपूर्ण
- थोड़ा महत्वपूर्ण
- कतई महत्वपूर्ण नहीं

आपके द्वारा चयनित "गुलाब जामुन अन्य लोगों के आसपास खाने लायक भोजन नहीं है।"

आपके लिए यह कथन कितना महत्वपूर्ण है।

- अत्यंत महत्वपूर्ण
- बहुत महत्वपूर्ण
- मामूली रूप से महत्वपूर्ण
- थोड़ा महत्वपूर्ण
- कतई महत्वपूर्ण नहीं

आपके द्वारा चयनित "गुलाब जामुन कि कीमत बहुत अधिक होती है।"

आपके लिए यह कथन कितना महत्वपूर्ण है।

- अत्यंत महत्वपूर्ण
- बहुत महत्वपूर्ण
- मामूली रूप से महत्वपूर्ण
- थोड़ा महत्वपूर्ण
- कतई महत्वपूर्ण नहीं

आपके द्वारा चयनित "गुलाब जामुन मूझे दिखने में अच्छा नहीं लगता है।"

आपके लिए यह कथन कितना महत्वपूर्ण है।

- अत्यंत महत्वपूर्ण
- बहुत महत्वपूर्ण
- मामूली रूप से महत्वपूर्ण
- थोड़ा महत्वपूर्ण
- कतई महत्वपूर्ण नहीं

आपके द्वारा चयनित "गुलाब जामुन मे कैलोरी की मात्रा बहुत अधिक होती है।"

आपके लिए यह कथन कितना महत्वपूर्ण है।

- अत्यंत महत्वपूर्ण
- बहुत महत्वपूर्ण
- मामूली रूप से महत्वपूर्ण
- थोड़ा महत्वपूर्ण
- कतई महत्वपूर्ण नहीं

आपके द्वारा चयनित "मैं गुलाब जामुन नहीं खाना चाहता हूँ।"

आपके लिए यह कथन कितना महत्वपूर्ण है।

- अत्यंत महत्वपूर्ण
- बहुत महत्वपूर्ण
- मामूली रूप से महत्वपूर्ण
- थोड़ा महत्वपूर्ण

- कतई महत्वपूर्ण नहीं

आपके द्वारा चयनित "मैं गुलाब जामुन खाने से दूसरों के सामने पिछड़ा महसूस करता हूँ।"

आपके लिए यह कथन कितना महत्वपूर्ण है।

- अत्यंत महत्वपूर्ण
- बहुत महत्वपूर्ण
- मामूली रूप से महत्वपूर्ण
- थोड़ा महत्वपूर्ण
- कतई महत्वपूर्ण नहीं

आपके द्वारा चयनित "मेरी गुलाब जामुन खाने कि इच्छा नहीं होती है।"

आपके लिए यह कथन कितना महत्वपूर्ण है।

- अत्यंत महत्वपूर्ण
- बहुत महत्वपूर्ण
- मामूली रूप से महत्वपूर्ण
- थोड़ा महत्वपूर्ण
- कतई महत्वपूर्ण नहीं

आपके द्वारा चयनित "मेने हाल ही में गुलाब जामुन खाय है। और अब मैं बार-बार गुलाब जामुन नहीं खाना चाहता हूँ।"

आपके लिए यह कथन कितना महत्वपूर्ण है।

- अत्यंत महत्वपूर्ण
- बहुत महत्वपूर्ण
- मामूली रूप से महत्वपूर्ण
- थोड़ा महत्वपूर्ण
- कतई महत्वपूर्ण नहीं

आपके द्वारा चयनित "मैं कभी गुलाब जामुननहीं खाऊंगा।क्योंकि मुझे हर दिन एक ही प्रकार का भोजन खाना पसंद है।"

आपके लिए यह कथन कितना महत्वपूर्ण है।

- अत्यंत महत्वपूर्ण
- बहुत महत्वपूर्ण
- मामूली रूप से महत्वपूर्ण
- थोड़ा महत्वपूर्ण
- कतई महत्वपूर्ण नहीं

आपके द्वारा चयनित "गुलाब जामुन खाना मूझे दुखी, अकेला और कुंठित महसूस कराता है।"

आपके लिए यह कथन कितना महत्वपूर्ण है।

- अत्यंत महत्वपूर्ण
- बहुत महत्वपूर्ण
- मामूली रूप से महत्वपूर्ण
- थोड़ा महत्वपूर्ण
- कतई महत्वपूर्ण नहीं

आपके द्वारा चयनित "क्योंकि गुलाब जामुन जैविक (आर्गेनिक) नहीं है।"

आपके लिए यह कथन कितना महत्वपूर्ण है।

- अत्यंत महत्वपूर्ण
- बहुत महत्वपूर्ण
- मामूली रूप से महत्वपूर्ण
- थोड़ा महत्वपूर्ण
- कतई महत्वपूर्ण नहीं

Reasons for Starchy food choices(Full RATA)

क्या आप चावल खाते हैं।

- हाँ
- नहीं

जब आप चावल खाने के बारे में सोचते है।

क्योंकि मैं चावल खाने का आदी हूँ। आपके लिए यह कथन कितना महत्वपूर्ण है।

- अत्यंत महत्वपूर्ण
- बहुत महत्वपूर्ण
- मामूली रूप से महत्वपूर्ण
- थोड़ा महत्वपूर्ण
- कतई महत्वपूर्ण नहीं

जब आप चावल खाने के बारे में सोचते है।

क्योंकि चावल खाना स्वास्थ्य के लिए लाभदायक हैं। आपके लिए यह कथन कितना महत्वपूर्ण है।

- अत्यंत महत्वपूर्ण
- बहुत महत्वपूर्ण
- मामूली रूप से महत्वपूर्ण
- थोड़ा महत्वपूर्ण
- कतई महत्वपूर्ण नहीं

जब आप चावल खाने के बारे में सोचते है।

क्योंकि चावल कम समय में बन जाता है। आपके लिए यह कथन कितना महत्वपूर्ण है।

- अत्यंत महत्वपूर्ण
- बहुत महत्वपूर्ण
- मामूली रूप से महत्वपूर्ण
- थोड़ा महत्वपूर्ण
- कतई महत्वपूर्ण नहीं

जब आप चावल खाने के बारे में सोचते है।

क्योंकि चावल का स्वाद अच्छा है। आपके लिए यह कथन कितना महत्वपूर्ण है।

- अत्यंत महत्वपूर्ण
- बहुत महत्वपूर्ण
- मामूली रूप से महत्वपूर्ण
- थोड़ा महत्वपूर्ण
- कतई महत्वपूर्ण नहीं

जब आप चावल खाने के बारे में सोचते है।

क्योंकि चावल का स्वाद अच्छा है। आपके लिए यह कथन कितना महत्वपूर्ण है।

- अत्यंत महत्वपूर्ण

- बहुत महत्वपूर्ण
- मामूली रूप से महत्वपूर्ण
- थोड़ा महत्वपूर्ण
- कतई महत्वपूर्ण नहीं

जब आप चावल खाने के बारे में सोचते है।

क्योंकि चावल प्राकृतिक है। आपके लिए यह कथन कितना महत्वपूर्ण है।

- अत्यंत महत्वपूर्ण
- बहुत महत्वपूर्ण
- मामूली रूप से महत्वपूर्ण
- थोड़ा महत्वपूर्ण
- कतई महत्वपूर्ण नहीं

जब आप चावल खाने के बारे में सोचते है।

क्योंकि आप अधिक पैसे खर्च नहीं करना चाहते है। आपके लिए यह कथन कितना महत्वपूर्ण है।

- अत्यंत महत्वपूर्ण
- बहुत महत्वपूर्ण
- मामूली रूप से महत्वपूर्ण
- थोड़ा महत्वपूर्ण
- कतई महत्वपूर्ण नहीं

जब आप चावल खाने के बारे में सोचते है।

क्योंकि चावल में कम कैलोरीज होती हैं। आपके लिए यह कथन कितना महत्वपूर्ण है।

- अत्यंत महत्वपूर्ण
- बहुत महत्वपूर्ण

- मामूली रूप से महत्वपूर्ण
- थोड़ा महत्वपूर्ण
- कतई महत्वपूर्ण नहीं

जब आप चावल खाने के बारे में सोचते है।

क्योंकि आप निराश रहते है। आपके लिए यह कथन कितना महत्वपूर्ण है।

- अत्यंत महत्वपूर्ण
- बहुत महत्वपूर्ण
- मामूली रूप से महत्वपूर्ण
- थोड़ा महत्वपूर्ण
- कतई महत्वपूर्ण नहीं

जब आप चावल खाने के बारे में सोचते है।

क्योंकि चावल खाना आपको दूसरों के सामने अच्छा दिखाता हैं। आपके लिए यह कथन कितना महत्वपूर्ण है।

- अत्यंत महत्वपूर्ण
- बहुत महत्वपूर्ण
- मामूली रूप से महत्वपूर्ण
- थोड़ा महत्वपूर्ण
- कतई महत्वपूर्ण नहीं

जब आप चावल खाने के बारे में सोचते है।

क्योंकि चावल नहीं खाना अशिष्ट होता है। आपके लिए यह कथन कितना महत्वपूर्ण है।

- अत्यंत महत्वपूर्ण

- बहुत महत्वपूर्ण
- मामूली रूप से महत्वपूर्ण
- थोड़ा महत्वपूर्ण
- कतई महत्वपूर्ण नहीं

जब आप चावल खाने के बारे में सोचते है।

क्योंकि चावल कि प्रस्तुति आकर्षक होती है। आपके लिए यह कथन कितना महत्वपूर्ण है।

- अत्यंत महत्वपूर्ण
- बहुत महत्वपूर्ण
- मामूली रूप से महत्वपूर्ण
- थोड़ा महत्वपूर्ण
- कतई महत्वपूर्ण नहीं

जब आप चावल खाने के बारे में सोचते है।

ताकि आप अन्य लोगों के साथ समय बिता सके। आपके लिए यह कथन कितना महत्वपूर्ण है।

- अत्यंत महत्वपूर्ण
- बहुत महत्वपूर्ण
- मामूली रूप से महत्वपूर्ण
- थोड़ा महत्वपूर्ण
- कतई महत्वपूर्ण नहीं

जब आप चावल खाने के बारे में सोचते है।

क्योंकि चावल जैविक होता है। आपके लिए यह कथन कितना महत्वपूर्ण है।

- अत्यंत महत्वपूर्ण

- बहुत महत्वपूर्ण
- मामूली रूप से महत्वपूर्ण
- थोड़ा महत्वपूर्ण
- कतई महत्वपूर्ण नहीं

जब आप चावल खाने के बारे में सोचते है।

क्योंकि आप बचपन से चावल खाते रहे है। आपके लिए यह कथन कितना महत्वपूर्ण है।

- अत्यंत महत्वपूर्ण
- बहुत महत्वपूर्ण
- मामूली रूप से महत्वपूर्ण
- थोड़ा महत्वपूर्ण
- कतई महत्वपूर्ण नहीं

जब आप चावल खाने के बारे में सोचते है।

क्योंकि आप चावल खाने में आनंद महसूस करते है। आपके लिए यह कथन कितना महत्वपूर्ण है।

- अत्यंत महत्वपूर्ण
- बहुत महत्वपूर्ण
- मामूली रूप से महत्वपूर्ण
- थोड़ा महत्वपूर्ण
- कतई महत्वपूर्ण नहीं

जब आप चावल खाने के बारे में सोचते है।

क्योंकि आप भूखे रहते है। आपके लिए यह कथन कितना महत्वपूर्ण है।

- अत्यंत महत्वपूर्ण
- बहुत महत्वपूर्ण

- मामूली रूप से महत्वपूर्ण
- थोड़ा महत्वपूर्ण
- कतई महत्वपूर्ण नहीं

जब आप चावल खाने के बारे में सोचते है।

क्योंकि आप आमतौर पर चावल खाते है। आपके लिए यह कथन कितना महत्वपूर्ण है।

- अत्यंत महत्वपूर्ण
- बहुत महत्वपूर्ण
- मामूली रूप से महत्वपूर्ण
- थोड़ा महत्वपूर्ण
- कतई महत्वपूर्ण नहीं

जब आप चावल खाने के बारे में सोचते है।

क्योंकि आपके भीतर चावल खाने कि भूख रहती है। आपके लिए यह कथन कितना महत्वपूर्ण है।

- अत्यंत महत्वपूर्ण
- बहुत महत्वपूर्ण
- मामूली रूप से महत्वपूर्ण
- थोड़ा महत्वपूर्ण
- कतई महत्वपूर्ण नहीं

जब आप चावल खाने के बारे में सोचते है।

क्योंकि चावल तैयार करना आसान है। आपके लिए यह कथन कितना महत्वपूर्ण है।

- अत्यंत महत्वपूर्ण
- बहुत महत्वपूर्ण
- मामूली रूप से महत्वपूर्ण

- थोड़ा महत्वपूर्ण
- कतई महत्वपूर्ण नहीं

जब आप चावल खाने के बारे में सोचते है।

क्योंकि आप उदास रहते है। आपके लिए यह कथन कितना महत्वपूर्ण है।

- अत्यंत महत्वपूर्ण
- बहुत महत्वपूर्ण
- मामूली रूप से महत्वपूर्ण
- थोड़ा महत्वपूर्ण
- कतई महत्वपूर्ण नहीं

जब आप चावल खाने के बारे में सोचते है।

क्योंकि चावल में वसा कि मात्रा कम होती है। आपके लिए यह कथन कितना महत्वपूर्ण है।

- अत्यंत महत्वपूर्ण
- बहुत महत्वपूर्ण
- मामूली रूप से महत्वपूर्ण
- थोड़ा महत्वपूर्ण
- कतई महत्वपूर्ण नहीं

जब आप चावल खाने के बारे में सोचते है।

क्योंकि आप चावल को टीवी या विज्ञापन पर देखते है। आपके लिए यह कथन कितना महत्वपूर्ण है।

- अत्यंत महत्वपूर्ण
- बहुत महत्वपूर्ण
- मामूली रूप से महत्वपूर्ण

- थोड़ा महत्वपूर्ण
- कतई महत्वपूर्ण नहीं

जब आप चावल खाने के बारे में सोचते है।

क्योंकि चावल सामाजिक समारोहों को अधिक आरामदायक बनाता है। आपके लिए यह कथन कितना महत्वपूर्ण है।

- अत्यंत महत्वपूर्ण
- बहुत महत्वपूर्ण
- मामूली रूप से महत्वपूर्ण
- थोड़ा महत्वपूर्ण
- कतई महत्वपूर्ण नहीं

जब आप चावल खाने के बारे में सोचते है।

क्योंकि आपको चावल खाना पड़ता है। आपके लिए यह कथन कितना महत्वपूर्ण है।

- अत्यंत महत्वपूर्ण
- बहुत महत्वपूर्ण
- मामूली रूप से महत्वपूर्ण
- थोड़ा महत्वपूर्ण
- कतई महत्वपूर्ण नहीं

जब आप चावल खाने के बारे में सोचते है।

क्योंकि चावल दूसरों को पसंद है। आपके लिए यह कथन कितना महत्वपूर्ण है।

- अत्यंत महत्वपूर्ण
- बहुत महत्वपूर्ण

- मामूली रूप से महत्वपूर्ण
- थोड़ा महत्वपूर्ण
- कतई महत्वपूर्ण नहीं

जब आप चावल खाने के बारे में सोचते है।

क्योंकि आप अकेला महसूस करते है। आपके लिए यह कथन कितना महत्वपूर्ण है।

- अत्यंत महत्वपूर्ण
- बहुत महत्वपूर्ण
- मामूली रूप से महत्वपूर्ण
- थोड़ा महत्वपूर्ण
- कतई महत्वपूर्ण नहीं

जब आप चावल खाने के बारे में सोचते है।

क्योंकि आप अपने वजन पर निगरानी रखते है। आपके लिए यह कथन कितना महत्वपूर्ण है।

- अत्यंत महत्वपूर्ण
- बहुत महत्वपूर्ण
- मामूली रूप से महत्वपूर्ण
- थोड़ा महत्वपूर्ण
- कतई महत्वपूर्ण नहीं

जब आप चावल खाने के बारे में सोचते है।

क्योंकि चावल में कोई हानिकारक (कीटनाशक, प्रदूषक, एंटीबायोटिक) पदार्थ नहीं होता है। आपके लिए यह कथन कितना महत्वपूर्ण है।

- अत्यंत महत्वपूर्ण

- बहुत महत्वपूर्ण
- मामूली रूप से महत्वपूर्ण
- थोड़ा महत्वपूर्ण
- कतई महत्वपूर्ण नहीं

जब आप चावल खाने के बारे में सोचते है।

क्योंकि चावल बनाना सुविधाजनक है। आपके लिए यह कथन कितना महत्वपूर्ण है।

- अत्यंत महत्वपूर्ण
- बहुत महत्वपूर्ण
- मामूली रूप से महत्वपूर्ण
- थोड़ा महत्वपूर्ण
- कतई महत्वपूर्ण नहीं

जब आप चावल खाने के बारे में सोचते है।

क्योंकि मूझे ऊर्जा की जरूरत होती है। आपके लिए यह कथन कितना महत्वपूर्ण है।

- अत्यंत महत्वपूर्ण
- बहुत महत्वपूर्ण
- मामूली रूप से महत्वपूर्ण
- थोड़ा महत्वपूर्ण
- कतई महत्वपूर्ण नहीं

जब आप चावल खाने के बारे में सोचते है।

क्योंकि आप चावल से परिचित है। आपके लिए यह कथन कितना महत्वपूर्ण है।

- अत्यंत महत्वपूर्ण

- बहुत महत्वपूर्ण
- मामूली रूप से महत्वपूर्ण
- थोड़ा महत्वपूर्ण
- कतई महत्वपूर्ण नहीं

जब आप चावल खाने के बारे में सोचते है।

क्योंकि आपको चावल पसंद है। आपके लिए यह कथन कितना महत्वपूर्ण है।

- अत्यंत महत्वपूर्ण
- बहुत महत्वपूर्ण
- मामूली रूप से महत्वपूर्ण
- थोड़ा महत्वपूर्ण
- कतई महत्वपूर्ण नहीं

जब आप चावल खाने के बारे में सोचते है।

क्योंकि चावल खाना सुखदेय है। आपके लिए यह कथन कितना महत्वपूर्ण है।

- अत्यंत महत्वपूर्ण
- बहुत महत्वपूर्ण
- मामूली रूप से महत्वपूर्ण
- थोड़ा महत्वपूर्ण
- कतई महत्वपूर्ण नहीं

जब आप चावल खाने के बारे में सोचते है।

आप चावल अपने आपको पुरस्कृत करने के लिए चावल खाते हैं। आपके लिए यह कथन कितना महत्वपूर्ण है।

- अत्यंत महत्वपूर्ण
- बहुत महत्वपूर्ण

- मामूली रूप से महत्वपूर्ण
- थोड़ा महत्वपूर्ण
- कतई महत्वपूर्ण नहीं

जब आप चावल खाने के बारे में सोचते है।

क्योंकि चावल खाना आपकी परंपरा का हिस्सा है। आपके लिए यह कथन कितना महत्वपूर्ण है।

- अत्यंत महत्वपूर्ण
- बहुत महत्वपूर्ण
- मामूली रूप से महत्वपूर्ण
- थोड़ा महत्वपूर्ण
- कतई महत्वपूर्ण नहीं

जब आप चावल खाने के बारे में सोचते है।

क्योंकि यह कुछ स्थितियों से संबंधित है। आपके लिए यह कथन कितना महत्वपूर्ण है।

- अत्यंत महत्वपूर्ण
- बहुत महत्वपूर्ण
- मामूली रूप से महत्वपूर्ण
- थोड़ा महत्वपूर्ण
- कतई महत्वपूर्ण नहीं

जब आप चावल खाने के बारे में सोचते है।

आप चावल संतुलित आहार पाने के लिए खाते हैं। आपके लिए यह कथन कितना महत्वपूर्ण है।

- अत्यंत महत्वपूर्ण
- बहुत महत्वपूर्ण

- मामूली रूप से महत्वपूर्ण
- थोड़ा महत्वपूर्ण
- कतई महत्वपूर्ण नहीं

जब आप चावल खाने के बारे में सोचते है।

क्योंकि चावल खाना सामाजिक है। आपके लिए यह कथन कितना महत्वपूर्ण है।

- अत्यंत महत्वपूर्ण
- बहुत महत्वपूर्ण
- मामूली रूप से महत्वपूर्ण
- थोड़ा महत्वपूर्ण
- कतई महत्वपूर्ण नहीं

जब आप चावल खाने के बारे में सोचते है।

क्योंकि चावल सस्ता है। आपके लिए यह कथन कितना महत्वपूर्ण है।

- अत्यंत महत्वपूर्ण
- बहुत महत्वपूर्ण
- मामूली रूप से महत्वपूर्ण
- थोड़ा महत्वपूर्ण
- कतई महत्वपूर्ण नहीं

जब आप चावल खाने के बारे में सोचते है।

क्योंकि चावल आपके लिए आकर्षक भोजन है। आपके लिए यह कथन कितना महत्वपूर्ण है।

- अत्यंत महत्वपूर्ण
- बहुत महत्वपूर्ण

- मामूली रूप से महत्वपूर्ण
- थोड़ा महत्वपूर्ण
- कतई महत्वपूर्ण नहीं

जब आप चावल खाने के बारे में सोचते है।

दुसरो को निराश करने से बचने के लिए आप चावल खाते हैं। आपके लिए यह कथन कितना महत्वपूर्ण है।

- अत्यंत महत्वपूर्ण
- बहुत महत्वपूर्ण
- मामूली रूप से महत्वपूर्ण
- थोड़ा महत्वपूर्ण
- कतई महत्वपूर्ण नहीं

जब आप चावल खाने के बारे में सोचते है।

क्योंकि चावल खाना फैशनेबल है।   आपके लिए यह कथन कितना महत्वपूर्ण है।

- अत्यंत महत्वपूर्ण
- बहुत महत्वपूर्ण
- मामूली रूप से महत्वपूर्ण
- थोड़ा महत्वपूर्ण
- कतई महत्वपूर्ण नहीं

जब आप चावल खाने के बारे में सोचते है।

क्योंकि चावल सेल पर उपलब्ध है। आपके लिए यह कथन कितना महत्वपूर्ण है।

- अत्यंत महत्वपूर्ण

- बहुत महत्वपूर्ण
- मामूली रूप से महत्वपूर्ण
- थोड़ा महत्वपूर्ण
- कतई महत्वपूर्ण नहीं

जब आप चावल खाने के बारे में सोचते है।

क्योंकि यह आपके शरीर को आकार में रखता है। आपके लिए यह कथन कितना महत्वपूर्ण है।

- अत्यंत महत्वपूर्ण
- बहुत महत्वपूर्ण
- मामूली रूप से महत्वपूर्ण
- थोड़ा महत्वपूर्ण
- कतई महत्वपूर्ण नहीं

जब आप चावल खाने के बारे में सोचते है।

क्योंकि चावल ही एकमात्र विकल्प था। आपके लिए यह कथन कितना महत्वपूर्ण है।

- अत्यंत महत्वपूर्ण
- बहुत महत्वपूर्ण
- मामूली रूप से महत्वपूर्ण
- थोड़ा महत्वपूर्ण
- कतई महत्वपूर्ण नहीं

जब आप चावल खाने के बारे में सोचते है।

क्योंकि आप चावल हर दिन खाना चाहते है।   आपके लिए यह कथन कितना महत्वपूर्ण है।

- अत्यंत महत्वपूर्ण

- बहुत महत्वपूर्ण
- मामूली रूप से महत्वपूर्ण
- थोड़ा महत्वपूर्ण
- कतई महत्वपूर्ण नहीं

जब आप चावल नहीं खाने के बारे में सोचते है।

क्योंकि आपको नहीं लगता कि चावल एक नाश्ता है। आपके लिए यह कथन कितना महत्वपूर्ण है।

- अत्यंत महत्वपूर्ण
- बहुत महत्वपूर्ण
- मामूली रूप से महत्वपूर्ण
- थोड़ा महत्वपूर्ण
- कतई महत्वपूर्ण नहीं

जब आप चावल नहीं खाने के बारे में सोचते है।

क्योंकि आपको चावल पसंद नहीं है। आपके लिए यह कथन कितना महत्वपूर्ण है।

- अत्यंत महत्वपूर्ण
- बहुत महत्वपूर्ण
- मामूली रूप से महत्वपूर्ण
- थोड़ा महत्वपूर्ण
- कतई महत्वपूर्ण नहीं

जब आप चावल नहीं खाने के बारे में सोचते है।

क्योंकि आप आमतौर पर चावल नहीं खाते है। आपके लिए यह कथन कितना महत्वपूर्ण है।

- अत्यंत महत्वपूर्ण
- बहुत महत्वपूर्ण

- मामूली रूप से महत्वपूर्ण
- थोड़ा महत्वपूर्ण
- कतई महत्वपूर्ण नहीं

जब आप चावल नहीं खाने के बारे में सोचते है।

क्योंकि चावल कि खुराक का आकार मेरे लिए उपयुक्त नहीं होता है। आपके लिए यह कथन कितना महत्वपूर्ण है।

- अत्यंत महत्वपूर्ण
- बहुत महत्वपूर्ण
- मामूली रूप से महत्वपूर्ण
- थोड़ा महत्वपूर्ण
- कतई महत्वपूर्ण नहीं

जब आप चावल नहीं खाने के बारे में सोचते है।

क्योंकि चावल आपकी भूख मिटाने के लिए पर्याप्त नहीं होता है।   आपके लिए यह कथन कितना महत्वपूर्ण है।

- अत्यंत महत्वपूर्ण
- बहुत महत्वपूर्ण
- मामूली रूप से महत्वपूर्ण
- थोड़ा महत्वपूर्ण
- कतई महत्वपूर्ण नहीं

जब आप चावल नहीं खाने के बारे में सोचते है।

क्योंकि चावल स्वास्थ्यय के लिए लाभदायक नहीं है। आपके लिए यह कथन कितना महत्वपूर्ण है।

- अत्यंत महत्वपूर्ण

- बहुत महत्वपूर्ण
- मामूली रूप से महत्वपूर्ण
- थोड़ा महत्वपूर्ण
- कतई महत्वपूर्ण नहीं

जब आप चावल नहीं खाने के बारे में सोचते है।

क्योंकि चावल बनाना मुश्किल होता है। आपके लिए यह कथन कितना महत्वपूर्ण है।

- अत्यंत महत्वपूर्ण
- बहुत महत्वपूर्ण
- मामूली रूप से महत्वपूर्ण
- थोड़ा महत्वपूर्ण
- कतई महत्वपूर्ण नहीं

जब आप चावल नहीं खाने के बारे में सोचते है।

क्योंकि चावल आपकी भूख को तृप्त नहीं कर पाता है।   आपके लिए यह कथन कितना महत्वपूर्ण है।

- अत्यंत महत्वपूर्ण
- बहुत महत्वपूर्ण
- मामूली रूप से महत्वपूर्ण
- थोड़ा महत्वपूर्ण
- कतई महत्वपूर्ण नहीं

जब आप चावल नहीं खाने के बारे में सोचते है।

क्योंकि चावल स्थिति के लिए उपयुक्त नहीं होता है। आपके लिए यह कथन कितना महत्वपूर्ण है।

- अत्यंत महत्वपूर्ण

- बहुत महत्वपूर्ण
- मामूली रूप से महत्वपूर्ण
- थोड़ा महत्वपूर्ण
- कतई महत्वपूर्ण नहीं

जब आप चावल नहीं खाने के बारे में सोचते है।

क्योंकि चावल अन्य लोगों के आसपास खाने लायक भोजन नहीं है। आपके लिए यह कथन कितना महत्वपूर्ण है।

- अत्यंत महत्वपूर्ण
- बहुत महत्वपूर्ण
- मामूली रूप से महत्वपूर्ण
- थोड़ा महत्वपूर्ण
- कतई महत्वपूर्ण नहीं

जब आप चावल नहीं खाने के बारे में सोचते है।

क्योंकि चावल कि कीमत बहुत अधिक है। आपके लिए यह कथन कितना महत्वपूर्ण है।

- अत्यंत महत्वपूर्ण
- बहुत महत्वपूर्ण
- मामूली रूप से महत्वपूर्ण
- थोड़ा महत्वपूर्ण
- कतई महत्वपूर्ण नहीं

जब आप चावल नहीं खाने के बारे में सोचते है।

क्योंकि चावल आपको दिखने में अच्छा नहीं लगता है। आपके लिए यह कथन कितना महत्वपूर्ण है।

- अत्यंत महत्वपूर्ण
- बहुत महत्वपूर्ण

- मामूली रूप से महत्वपूर्ण
- थोड़ा महत्वपूर्ण
- कतई महत्वपूर्ण नहीं

जब आप चावल नहीं खाने के बारे में सोचते है।

क्योंकि चावल मे कैलोरी के मात्रा बहुत अधिक होती है। आपके लिए यह कथन कितना महत्वपूर्ण है।

- अत्यंत महत्वपूर्ण
- बहुत महत्वपूर्ण
- मामूली रूप से महत्वपूर्ण
- थोड़ा महत्वपूर्ण
- कतई महत्वपूर्ण नहीं

जब आप चावल नहीं खाने के बारे में सोचते है।

क्योंकि आप चावल नहीं खाना चाहते है। आपके लिए यह कथन कितना महत्वपूर्ण है।

- अत्यंत महत्वपूर्ण
- बहुत महत्वपूर्ण
- मामूली रूप से महत्वपूर्ण
- थोड़ा महत्वपूर्ण
- कतई महत्वपूर्ण नहीं

जब आप चावल नहीं खाने के बारे में सोचते है।

क्योंकि आप चावल खाने से दूसरों के सामने पिछड़ा महसूस करते है। आपके लिए यह कथन कितना महत्वपूर्ण है।

- अत्यंत महत्वपूर्ण

- बहुत महत्वपूर्ण
- मामूली रूप से महत्वपूर्ण
- थोड़ा महत्वपूर्ण
- कतई महत्वपूर्ण नहीं

जब आप चावल नहीं खाने के बारे में सोचते है।

क्योंकि आपकी चावल खाने कि इच्छा नहीं होती है। आपके लिए यह कथन कितना महत्वपूर्ण है।

- अत्यंत महत्वपूर्ण
- बहुत महत्वपूर्ण
- मामूली रूप से महत्वपूर्ण
- थोड़ा महत्वपूर्ण
- कतई महत्वपूर्ण नहीं

जब आप चावल नहीं खाने के बारे में सोचते है।

क्योंकि अपने हाल ही में चावल खाया था और अब आप बार-बार चावल नहीं खाना चाहते है। आपके लिए यह कथन कितना महत्वपूर्ण है।

- अत्यंत महत्वपूर्ण
- बहुत महत्वपूर्ण
- मामूली रूप से महत्वपूर्ण
- थोड़ा महत्वपूर्ण
- कतई महत्वपूर्ण नहीं

जब आप चावल नहीं खाने के बारे में सोचते है।

क्योंकि आपको हर दिन एक ही प्रकार का भोजन खाना पसंद है। आपके लिए यह कथन कितना महत्वपूर्ण है।

- अत्यंत महत्वपूर्ण
- बहुत महत्वपूर्ण
- मामूली रूप से महत्वपूर्ण
- थोड़ा महत्वपूर्ण
- कतई महत्वपूर्ण नहीं

जब आप चावल नहीं खाने के बारे में सोचते है।

क्योंकि चावल खाना आपको दुखी, अकेला और कुंठित महसूस कराता है। आपके लिए यह कथन कितना महत्वपूर्ण है।

- अत्यंत महत्वपूर्ण
- बहुत महत्वपूर्ण
- मामूली रूप से महत्वपूर्ण
- थोड़ा महत्वपूर्ण
- कतई महत्वपूर्ण नहीं

जब आप चावल नहीं खाने के बारे में सोचते है।

क्योंकि चावल जैविक (आर्गेनिक) नहीं है।   आपके लिए यह कथन कितना महत्वपूर्ण है।

- अत्यंत महत्वपूर्ण
- बहुत महत्वपूर्ण
- मामूली रूप से महत्वपूर्ण
- थोड़ा महत्वपूर्ण
- कतई महत्वपूर्ण नहीं

Reasons for Protein food choices(Full RATA)

क्या आप दाल खाते हैं ।

- हाँ
- नहीं

जब आप दाल खाने के बारे में सोचते है।

क्योंकि आप दाल खाने के आदी हैं। आपके लिए यह कथन कितना महत्वपूर्ण है।

- अत्यंत महत्वपूर्ण
- बहुत महत्वपूर्ण
- मामूली रूप से महत्वपूर्ण
- थोड़ा महत्वपूर्ण
- कतई महत्वपूर्ण नहीं

जब आप दाल खाने के बारे में सोचते है।

क्योंकि दाल खाना स्वास्थय के लिए लाभदायक हैं। आपके लिए यह कथन कितना महत्वपूर्ण है।

- अत्यंत महत्वपूर्ण
- बहुत महत्वपूर्ण
- मामूली रूप से महत्वपूर्ण
- थोड़ा महत्वपूर्ण
- कतई महत्वपूर्ण नहीं

जब आप दाल खाने के बारे में सोचते है।

क्योंकि दाल कम समय में बन जाती है। आपके लिए यह कथन कितना महत्वपूर्ण है।

- अत्यंत महत्वपूर्ण
- बहुत महत्वपूर्ण
- मामूली रूप से महत्वपूर्ण
- थोड़ा महत्वपूर्ण

- कतई महत्वपूर्ण नहीं

जब आप दाल खाने के बारे में सोचते है।

क्योंकि दाल का स्वाद अच्छा है। आपके लिए यह कथन कितना महत्वपूर्ण है।

- अत्यंत महत्वपूर्ण
- बहुत महत्वपूर्ण
- मामूली रूप से महत्वपूर्ण
- थोड़ा महत्वपूर्ण
- कतई महत्वपूर्ण नहीं

जब आप दाल खाने के बारे में सोचते है।

क्योंकि दाल मुझे तृप्त करती है। आपके लिए यह कथन कितना महत्वपूर्ण है।

- अत्यंत महत्वपूर्ण
- बहुत महत्वपूर्ण
- मामूली रूप से महत्वपूर्ण
- थोड़ा महत्वपूर्ण
- कतई महत्वपूर्ण नहीं

जब आप दाल खाने के बारे में सोचते है।

क्योंकि दाल प्राकृतिक है। आपके लिए यह कथन कितना महत्वपूर्ण है।

- अत्यंत महत्वपूर्ण
- बहुत महत्वपूर्ण
- मामूली रूप से महत्वपूर्ण
- थोड़ा महत्वपूर्ण
- कतई महत्वपूर्ण नहीं

जब आप दाल खाने के बारे में सोचते है।

क्योंकि आप अधिक पैसे खर्च नहीं करना चाहते। आपके लिए यह कथन कितना महत्वपूर्ण है।

- अत्यंत महत्वपूर्ण
- बहुत महत्वपूर्ण
- मामूली रूप से महत्वपूर्ण
- थोड़ा महत्वपूर्ण
- कतई महत्वपूर्ण नहीं

जब आप दाल खाने के बारे में सोचते है।

क्योंकि दाल में कम कैलोरीज होती हैं। आपके लिए यह कथन कितना महत्वपूर्ण है।

- अत्यंत महत्वपूर्ण
- बहुत महत्वपूर्ण
- मामूली रूप से महत्वपूर्ण
- थोड़ा महत्वपूर्ण
- कतई महत्वपूर्ण नहीं

जब आप दाल खाने के बारे में सोचते है।

क्योंकि आप निराश रहते हैं। आपके लिए यह कथन कितना महत्वपूर्ण है।

- अत्यंत महत्वपूर्ण
- बहुत महत्वपूर्ण
- मामूली रूप से महत्वपूर्ण
- थोड़ा महत्वपूर्ण
- कतई महत्वपूर्ण नहीं

जब आप दाल खाने के बारे में सोचते है।

क्योंकि दाल खाना आपको दूसरों के सामने अच्छा दिखाता हैं। आपके लिए यह कथन कितना महत्वपूर्ण है।

- अत्यंत महत्वपूर्ण
- बहुत महत्वपूर्ण
- मामूली रूप से महत्वपूर्ण
- थोड़ा महत्वपूर्ण
- कतई महत्वपूर्ण नहीं

जब आप दाल खाने के बारे में सोचते है।

क्योंकि दाल नहीं खाना अशिष्ट होगा। आपके लिए यह कथन कितना महत्वपूर्ण है।

- अत्यंत महत्वपूर्ण
- बहुत महत्वपूर्ण
- मामूली रूप से महत्वपूर्ण
- थोड़ा महत्वपूर्ण
- कतई महत्वपूर्ण नहीं

जब आप दाल खाने के बारे में सोचते है।

क्योंकि दाल कि प्रस्तुति आकर्षक होती है। आपके लिए यह कथन कितना महत्वपूर्ण है।

- अत्यंत महत्वपूर्ण
- बहुत महत्वपूर्ण
- मामूली रूप से महत्वपूर्ण
- थोड़ा महत्वपूर्ण
- कतई महत्वपूर्ण नहीं

जब आप दाल खाने के बारे में सोचते है।

ताकि आप अन्य लोगों के साथ समय बिता सके। आपके लिए यह कथन कितना महत्वपूर्ण है।

- अत्यंत महत्वपूर्ण
- बहुत महत्वपूर्ण
- मामूली रूप से महत्वपूर्ण
- थोड़ा महत्वपूर्ण
- कतई महत्वपूर्ण नहीं

जब आप दाल खाने के बारे में सोचते है।

क्योंकि दाल जैविक होता है। आपके लिए यह कथन कितना महत्वपूर्ण है।

- अत्यंत महत्वपूर्ण
- बहुत महत्वपूर्ण
- मामूली रूप से महत्वपूर्ण
- थोड़ा महत्वपूर्ण
- कतई महत्वपूर्ण नहीं

जब आप दाल खाने के बारे में सोचते है।

क्योंकि आप बचपन से दाल खाते रहे है। आपके लिए यह कथन कितना महत्वपूर्ण है।

- अत्यंत महत्वपूर्ण
- बहुत महत्वपूर्ण
- मामूली रूप से महत्वपूर्ण
- थोड़ा महत्वपूर्ण
- कतई महत्वपूर्ण नहीं

जब आप दाल खाने के बारे में सोचते है।

क्योंकि आप दाल खाने में आनंद महसूस करते है। आपके लिए यह कथन कितना महत्वपूर्ण है।

- अत्यंत महत्वपूर्ण
- बहुत महत्वपूर्ण
- मामूली रूप से महत्वपूर्ण
- थोड़ा महत्वपूर्ण
- कतई महत्वपूर्ण नहीं

जब आप दाल खाने के बारे में सोचते है।

क्योंकि आप भूखे रहते हैं। आपके लिए यह कथन कितना महत्वपूर्ण है।

- अत्यंत महत्वपूर्ण
- बहुत महत्वपूर्ण
- मामूली रूप से महत्वपूर्ण
- थोड़ा महत्वपूर्ण
- कतई महत्वपूर्ण नहीं

जब आप दाल खाने के बारे में सोचते है।

क्योंकि आप आमतौर पर दाल खाते हैं। आपके लिए यह कथन कितना महत्वपूर्ण है।

- अत्यंत महत्वपूर्ण
- बहुत महत्वपूर्ण
- मामूली रूप से महत्वपूर्ण
- थोड़ा महत्वपूर्ण
- कतई महत्वपूर्ण नहीं

जब आप दाल खाने के बारे में सोचते है।

क्योंकि आपके भीतर दाल खाने कि भूख रहती है। आपके लिए यह कथन कितना महत्वपूर्ण है।

- अत्यंत महत्वपूर्ण
- बहुत महत्वपूर्ण

- मामूली रूप से महत्वपूर्ण
- थोड़ा महत्वपूर्ण
- कतई महत्वपूर्ण नहीं

जब आप दाल खाने के बारे में सोचते है।

क्योंकि दाल बनाना आसान है। आपके लिए यह कथन कितना महत्वपूर्ण है।

- अत्यंत महत्वपूर्ण
- बहुत महत्वपूर्ण
- मामूली रूप से महत्वपूर्ण
- थोड़ा महत्वपूर्ण
- कतई महत्वपूर्ण नहीं

जब आप दाल खाने के बारे में सोचते है।

क्योंकि आप उदास रहते हैं। आपके लिए यह कथन कितना महत्वपूर्ण है।

- अत्यंत महत्वपूर्ण
- बहुत महत्वपूर्ण
- मामूली रूप से महत्वपूर्ण
- थोड़ा महत्वपूर्ण
- कतई महत्वपूर्ण नहीं

जब आप दाल खाने के बारे में सोचते है।

क्योंकि दाल में वसा कि मात्रा कम होती है। आपके लिए यह कथन कितना महत्वपूर्ण है।

- अत्यंत महत्वपूर्ण
- बहुत महत्वपूर्ण
- मामूली रूप से महत्वपूर्ण
- थोड़ा महत्वपूर्ण

- कतई महत्वपूर्ण नहीं

जब आप दाल खाने के बारे में सोचते है।

क्योंकि आप दाल को टीवी या विज्ञापन पर देखते हैं। आपके लिए यह कथन कितना महत्वपूर्ण है।

- अत्यंत महत्वपूर्ण
- बहुत महत्वपूर्ण
- मामूली रूप से महत्वपूर्ण
- थोड़ा महत्वपूर्ण
- कतई महत्वपूर्ण नहीं

जब आप दाल खाने के बारे में सोचते है।

क्योंकि दाल सामाजिक समारोहों को अधिक आरामदायक बनाता है। आपके लिए यह कथन कितना महत्वपूर्ण है।

- अत्यंत महत्वपूर्ण
- बहुत महत्वपूर्ण
- मामूली रूप से महत्वपूर्ण
- थोड़ा महत्वपूर्ण
- कतई महत्वपूर्ण नहीं

जब आप दाल खाने के बारे में सोचते है।

क्योंकि आपको दाल खानी पड़ती हैं। आपके लिए यह कथन कितना महत्वपूर्ण है।

- अत्यंत महत्वपूर्ण
- बहुत महत्वपूर्ण
- मामूली रूप से महत्वपूर्ण
- थोड़ा महत्वपूर्ण

- कतई महत्वपूर्ण नहीं

जब आप दाल खाने के बारे में सोचते है।

क्योंकि दाल दूसरों को पसंद है। आपके लिए यह कथन कितना महत्वपूर्ण है।

- अत्यंत महत्वपूर्ण
- बहुत महत्वपूर्ण
- मामूली रूप से महत्वपूर्ण
- थोड़ा महत्वपूर्ण
- कतई महत्वपूर्ण नहीं

जब आप दाल खाने के बारे में सोचते है।

क्योंकि आप अकेला महसूस करते है। आपके लिए यह कथन कितना महत्वपूर्ण है।

- अत्यंत महत्वपूर्ण
- बहुत महत्वपूर्ण
- मामूली रूप से महत्वपूर्ण
- थोड़ा महत्वपूर्ण
- कतई महत्वपूर्ण नहीं

जब आप दाल खाने के बारे में सोचते है।

क्योंकि आप अपने वजन पर निगरानी रखते है। आपके लिए यह कथन कितना महत्वपूर्ण है।

- अत्यंत महत्वपूर्ण
- बहुत महत्वपूर्ण
- मामूली रूप से महत्वपूर्ण
- थोड़ा महत्वपूर्ण
- कतई महत्वपूर्ण नहीं

जब आप दाल खाने के बारे में सोचते है।

क्योंकि दाल में कोई हानिकारक (कीटनाशक, प्रदूषक, एंटीबायोटिक) पदार्थ नहीं होता है। आपके लिए यह कथन कितना महत्वपूर्ण है।

- अत्यंत महत्वपूर्ण
- बहुत महत्वपूर्ण
- मामूली रूप से महत्वपूर्ण
- थोड़ा महत्वपूर्ण
- कतई महत्वपूर्ण नहीं

जब आप दाल खाने के बारे में सोचते है।

क्योंकि दाल बनाना सुविधाजनक है। आपके लिए यह कथन कितना महत्वपूर्ण है।

- अत्यंत महत्वपूर्ण
- बहुत महत्वपूर्ण
- मामूली रूप से महत्वपूर्ण
- थोड़ा महत्वपूर्ण
- कतई महत्वपूर्ण नहीं

जब आप दाल खाने के बारे में सोचते है।

क्योंकि मूझे ऊर्जा की जरूरत होती है। आपके लिए यह कथन कितना महत्वपूर्ण है।

- अत्यंत महत्वपूर्ण
- बहुत महत्वपूर्ण
- मामूली रूप से महत्वपूर्ण
- थोड़ा महत्वपूर्ण
- कतई महत्वपूर्ण नहीं

जब आप दाल खाने के बारे में सोचते है।

क्योंकि आप दाल से परिचित है। आपके लिए यह कथन कितना महत्वपूर्ण है।

- अत्यंत महत्वपूर्ण
- बहुत महत्वपूर्ण
- मामूली रूप से महत्वपूर्ण
- थोड़ा महत्वपूर्ण
- कतई महत्वपूर्ण नहीं

जब आप दाल खाने के बारे में सोचते है।

क्योंकि आपको दाल पसंद है। आपके लिए यह कथन कितना महत्वपूर्ण है।

- अत्यंत महत्वपूर्ण
- बहुत महत्वपूर्ण
- मामूली रूप से महत्वपूर्ण
- थोड़ा महत्वपूर्ण
- कतई महत्वपूर्ण नहीं

जब आप दाल खाने के बारे में सोचते है।

क्योंकि दाल खाना सुखदेय है। आपके लिए यह कथन कितना महत्वपूर्ण है।

- अत्यंत महत्वपूर्ण
- बहुत महत्वपूर्ण
- मामूली रूप से महत्वपूर्ण
- थोड़ा महत्वपूर्ण
- कतई महत्वपूर्ण नहीं

जब आप दाल खाने के बारे में सोचते है।

आप दाल अपने आपको पुरस्कृत करने के लिए दाल खाते हैं। आपके लिए यह कथन कितना महत्वपूर्ण

- अत्यंत महत्वपूर्ण
- बहुत महत्वपूर्ण
- मामूली रूप से महत्वपूर्ण
- थोड़ा महत्वपूर्ण
- कतई महत्वपूर्ण नहीं

जब आप दाल खाने के बारे में सोचते है।

क्योंकि दाल खाना आपकी परंपरा का हिस्सा है। आपके लिए यह कथन कितना महत्वपूर्ण है।

- अत्यंत महत्वपूर्ण
- बहुत महत्वपूर्ण
- मामूली रूप से महत्वपूर्ण
- थोड़ा महत्वपूर्ण
- कतई महत्वपूर्ण नहीं

जब आप दाल खाने के बारे में सोचते है।

क्योंकि यह कुछ स्थितियों से संबंधित है। आपके लिए यह कथन कितना महत्वपूर्ण है।

- अत्यंत महत्वपूर्ण
- बहुत महत्वपूर्ण
- मामूली रूप से महत्वपूर्ण
- थोड़ा महत्वपूर्ण
- कतई महत्वपूर्ण नहीं

जब आप दाल खाने के बारे में सोचते है।

आप दाल संतुलित आहार पाने के लिए खाते हैं। आपके लिए यह कथन कितना महत्वपूर्ण है।

- अत्यंत महत्वपूर्ण
- बहुत महत्वपूर्ण
- मामूली रूप से महत्वपूर्ण
- थोड़ा महत्वपूर्ण
- कतई महत्वपूर्ण नहीं

जब आप दाल खाने के बारे में सोचते है।

क्योंकि दाल खाना सामाजिक है। आपके लिए यह कथन कितना महत्वपूर्ण है।

- अत्यंत महत्वपूर्ण
- बहुत महत्वपूर्ण
- मामूली रूप से महत्वपूर्ण
- थोड़ा महत्वपूर्ण
- कतई महत्वपूर्ण नहीं

जब आप दाल खाने के बारे में सोचते है।

क्योंकि दाल सस्ती है। आपके लिए यह कथन कितना महत्वपूर्ण है।

- अत्यंत महत्वपूर्ण
- बहुत महत्वपूर्ण
- मामूली रूप से महत्वपूर्ण
- थोड़ा महत्वपूर्ण
- कतई महत्वपूर्ण नहीं

जब आप दाल खाने के बारे में सोचते है।

क्योंकि दाल आपके लिए आकर्षक भोजन है। आपके लिए यह कथन कितना महत्वपूर्ण है।

- अत्यंत महत्वपूर्ण
- बहुत महत्वपूर्ण
- मामूली रूप से महत्वपूर्ण
- थोड़ा महत्वपूर्ण
- कतई महत्वपूर्ण नहीं

जब आप दाल खाने के बारे में सोचते है।

दुसरो को निराश करने से बचने के लिए आप दाल खाते हैं। आपके लिए यह कथन कितना महत्वपूर्ण है।

- अत्यंत महत्वपूर्ण
- बहुत महत्वपूर्ण
- मामूली रूप से महत्वपूर्ण
- थोड़ा महत्वपूर्ण
- कतई महत्वपूर्ण नहीं

जब आप दाल खाने के बारे में सोचते है।

क्योंकि दाल खाना फैशनेबल है।   आपके लिए यह कथन कितना महत्वपूर्ण है।

- अत्यंत महत्वपूर्ण
- बहुत महत्वपूर्ण
- मामूली रूप से महत्वपूर्ण
- थोड़ा महत्वपूर्ण
- कतई महत्वपूर्ण नहीं

जब आप दाल खाने के बारे में सोचते है।

क्योंकि दाल सेल पर उपलब्ध है। आपके लिए यह कथन कितना महत्वपूर्ण है।

- अत्यंत महत्वपूर्ण
- बहुत महत्वपूर्ण
- मामूली रूप से महत्वपूर्ण
- थोड़ा महत्वपूर्ण
- कतई महत्वपूर्ण नहीं

जब आप दाल खाने के बारे में सोचते है।

क्योंकि यह आपके शरीर को आकार में रखता है। आपके लिए यह कथन कितना महत्वपूर्ण है।

- अत्यंत महत्वपूर्ण
- बहुत महत्वपूर्ण
- मामूली रूप से महत्वपूर्ण
- थोड़ा महत्वपूर्ण
- कतई महत्वपूर्ण नहीं

जब आप दाल खाने के बारे में सोचते है।

क्योंकि दाल ही एकमात्र विकल्प था। आपके लिए यह कथन कितना महत्वपूर्ण है।

- अत्यंत महत्वपूर्ण
- बहुत महत्वपूर्ण
- मामूली रूप से महत्वपूर्ण
- थोड़ा महत्वपूर्ण
- कतई महत्वपूर्ण नहीं

जब आप दाल खाने के बारे में सोचते है।

क्योंकि आप दाल हर दिन खाना चाहते है। आपके लिए यह कथन कितना महत्वपूर्ण है।

- अत्यंत महत्वपूर्ण

- बहुत महत्वपूर्ण
- मामूली रूप से महत्वपूर्ण
- थोड़ा महत्वपूर्ण
- कतई महत्वपूर्ण नहीं

जब आप दाल नहीं खाने के बारे में सोचते है।

क्योंकि आपको नहीं लगता कि दाल एक नाश्ता है। आपके लिए यह कथन कितना महत्वपूर्ण है।

- अत्यंत महत्वपूर्ण
- बहुत महत्वपूर्ण
- मामूली रूप से महत्वपूर्ण
- थोड़ा महत्वपूर्ण
- कतई महत्वपूर्ण नहीं

जब आप दाल नहीं खाने के बारे में सोचते है।

क्योंकि आपको दाल पसंद नहीं है। आपके लिए यह कथन कितना महत्वपूर्ण है।

- अत्यंत महत्वपूर्ण
- बहुत महत्वपूर्ण
- मामूली रूप से महत्वपूर्ण
- थोड़ा महत्वपूर्ण
- कतई महत्वपूर्ण नहीं

जब आप दाल नहीं खाने के बारे में सोचते है।

क्योंकि आप आमतौर पर दाल नहीं खाते है। आपके लिए यह कथन कितना महत्वपूर्ण है।

- अत्यंत महत्वपूर्ण
- बहुत महत्वपूर्ण

- मामूली रूप से महत्वपूर्ण
- थोड़ा महत्वपूर्ण
- कतई महत्वपूर्ण नहीं

जब आप दाल नहीं खाने के बारे में सोचते है।

क्योंकि दाल कि खुराक का आकार मेरे लिए उपयुक्त नहीं होता है। आपके लिए यह कथन कितना महत्वपूर्ण है।

- अत्यंत महत्वपूर्ण
- बहुत महत्वपूर्ण
- मामूली रूप से महत्वपूर्ण
- थोड़ा महत्वपूर्ण
- कतई महत्वपूर्ण नहीं

जब आप दाल नहीं खाने के बारे में सोचते है।

क्योंकि दाल आपकी भूख मिटाने के लिए पर्याप्त नहीं होता है। आपके लिए यह कथन कितना महत्वपूर्ण है।

- अत्यंत महत्वपूर्ण
- बहुत महत्वपूर्ण
- मामूली रूप से महत्वपूर्ण
- थोड़ा महत्वपूर्ण
- कतई महत्वपूर्ण नहीं

जब आप दाल नहीं खाने के बारे में सोचते है।

क्योंकि दाल स्वास्थ्यय के लिए लाभदायक नहीं है। आपके लिए यह कथन कितना महत्वपूर्ण है।

- अत्यंत महत्वपूर्ण

- बहुत महत्वपूर्ण
- मामूली रूप से महत्वपूर्ण
- थोड़ा महत्वपूर्ण
- कतई महत्वपूर्ण नहीं

जब आप दाल नहीं खाने के बारे में सोचते है।

क्योंकि दाल बनाना मुश्किल होता है। आपके लिए यह कथन कितना महत्वपूर्ण है।

- अत्यंत महत्वपूर्ण
- बहुत महत्वपूर्ण
- मामूली रूप से महत्वपूर्ण
- थोड़ा महत्वपूर्ण
- कतई महत्वपूर्ण नहीं

जब आप दाल नहीं खाने के बारे में सोचते है।

क्योंकि दाल आपकी भूख को तृप्त नहीं कर पाती है। आपके लिए यह कथन कितना महत्वपूर्ण है।

- अत्यंत महत्वपूर्ण
- बहुत महत्वपूर्ण
- मामूली रूप से महत्वपूर्ण
- थोड़ा महत्वपूर्ण
- कतई महत्वपूर्ण नहीं

जब आप दाल नहीं खाने के बारे में सोचते है।

क्योंकि दाल स्थिति के लिए उपयुक्त नहीं होता है। आपके लिए यह कथन कितना महत्वपूर्ण है।

- अत्यंत महत्वपूर्ण
- बहुत महत्वपूर्ण

- मामूली रूप से महत्वपूर्ण
- थोड़ा महत्वपूर्ण
- कतई महत्वपूर्ण नहीं

जब आप दाल नहीं खाने के बारे में सोचते है।

क्योंकि दाल अन्य लोगों के आसपास खाने लायक भोजन नहीं है। आपके लिए यह कथन कितना महत्वपूर्ण है।

- अत्यंत महत्वपूर्ण
- बहुत महत्वपूर्ण
- मामूली रूप से महत्वपूर्ण
- थोड़ा महत्वपूर्ण
- कतई महत्वपूर्ण नहीं

जब आप दाल नहीं खाने के बारे में सोचते है।

क्योंकि दाल कि कीमत बहुत अधिक है। आपके लिए यह कथन कितना महत्वपूर्ण है।

- अत्यंत महत्वपूर्ण
- बहुत महत्वपूर्ण
- मामूली रूप से महत्वपूर्ण
- थोड़ा महत्वपूर्ण
- कतई महत्वपूर्ण नहीं

जब आप दाल नहीं खाने के बारे में सोचते है।

क्योंकि दाल आपको दिखने में अच्छा नहीं लगता है। आपके लिए यह कथन कितना महत्वपूर्ण है।

- अत्यंत महत्वपूर्ण
- बहुत महत्वपूर्ण
- मामूली रूप से महत्वपूर्ण
- थोड़ा महत्वपूर्ण

- कतई महत्वपूर्ण नहीं

जब आप दाल नहीं खाने के बारे में सोचते है।

क्योंकि दाल मे कैलोरी के मात्रा बहुत अधिक होती है। आपके लिए यह कथन कितना महत्वपूर्ण है।

- अत्यंत महत्वपूर्ण
- बहुत महत्वपूर्ण
- मामूली रूप से महत्वपूर्ण
- थोड़ा महत्वपूर्ण
- कतई महत्वपूर्ण नहीं

जब आप दाल नहीं खाने के बारे में सोचते है।

क्योंकि आप दाल नहीं खाना चाहते है। आपके लिए यह कथन कितना महत्वपूर्ण है।

- अत्यंत महत्वपूर्ण
- बहुत महत्वपूर्ण
- मामूली रूप से महत्वपूर्ण
- थोड़ा महत्वपूर्ण
- कतई महत्वपूर्ण नहीं

जब आप दाल नहीं खाने के बारे में सोचते है।

क्योंकि आप दाल खाने से दूसरों के सामने पिछड़ा महसूस करते हैं। आपके लिए यह कथन कितना महत्वपूर्ण है।

- अत्यंत महत्वपूर्ण
- बहुत महत्वपूर्ण
- मामूली रूप से महत्वपूर्ण
- थोड़ा महत्वपूर्ण
- कतई महत्वपूर्ण नहीं

जब आप दाल नहीं खाने के बारे में सोचते है।

क्योंकि आपकी दाल खाने कि इच्छा नहीं होती हैं। आपके लिए यह कथन कितना महत्वपूर्ण है।

- अत्यंत महत्वपूर्ण
- बहुत महत्वपूर्ण
- मामूली रूप से महत्वपूर्ण
- थोड़ा महत्वपूर्ण
- कतई महत्वपूर्ण नहीं

जब आप दाल नहीं खाने के बारे में सोचते है।

क्योंकि अपने हाल ही में दाल खाया था और अब आप बार-बार दाल नहीं खाना चाहते है। आपके लिए यह कथन कितना महत्वपूर्ण है।

- अत्यंत महत्वपूर्ण
- बहुत महत्वपूर्ण
- मामूली रूप से महत्वपूर्ण
- थोड़ा महत्वपूर्ण
- कतई महत्वपूर्ण नहीं

जब आप दाल नहीं खाने के बारे में सोचते है।

क्योंकि आपको हर दिन एक ही प्रकार का भोजन खाना पसंद है। आपके लिए यह कथन कितना महत्वपूर्ण है।

- अत्यंत महत्वपूर्ण
- बहुत महत्वपूर्ण
- मामूली रूप से महत्वपूर्ण
- थोड़ा महत्वपूर्ण
- कतई महत्वपूर्ण नहीं

जब आप दाल नहीं खाने के बारे में सोचते है।

क्योंकि दाल खाना आपको दुखी, अकेला और कुंठित महसूस कराता है। आपके लिए यह कथन कितना महत्वपूर्ण है।

- अत्यंत महत्वपूर्ण
- बहुत महत्वपूर्ण
- मामूली रूप से महत्वपूर्ण
- थोड़ा महत्वपूर्ण
- कतई महत्वपूर्ण नहीं

जब आप दाल नहीं खाने के बारे में सोचते है।

क्योंकि दाल जैविक (आर्गेनिक) नहीं है। आपके लिए यह कथन कितना महत्वपूर्ण है।

- अत्यंत महत्वपूर्ण
- बहुत महत्वपूर्ण
- मामूली रूप से महत्वपूर्ण
- थोड़ा महत्वपूर्ण
- कतई महत्वपूर्ण नहीं

Reasons for Dairy food choices(Full RATA)

क्या आप दूध खाते हैं।

- हाँ
- नहीं

जब आप दूध खाने के बारे में सोचते हैं।

क्योंकि आप दूध खाने के आदी हैं। आपके लिए यह कथन कितना महत्वपूर्ण है।

- अत्यंत महत्वपूर्ण
- बहुत महत्वपूर्ण
- मामूली रूप से महत्वपूर्ण
- थोड़ा महत्वपूर्ण

- कतई महत्वपूर्ण नहीं

जब आप दूध खाने के बारे में सोचते हैं।

क्योंकि दूध खाना स्वास्थ्य के लिए लाभदायक हैं। आपके लिए यह कथन कितना महत्वपूर्ण है।

- अत्यंत महत्वपूर्ण
- बहुत महत्वपूर्ण
- मामूली रूप से महत्वपूर्ण
- थोड़ा महत्वपूर्ण
- कतई महत्वपूर्ण नहीं

जब आप दूध खाने के बारे में सोचते हैं।

क्योंकि दूध कम समय में बन जाता है। आपके लिए यह कथन कितना महत्वपूर्ण है।

- अत्यंत महत्वपूर्ण
- बहुत महत्वपूर्ण
- मामूली रूप से महत्वपूर्ण
- थोड़ा महत्वपूर्ण
- कतई महत्वपूर्ण नहीं

जब आप दूध खाने के बारे में सोचते हैं।

क्योंकि दूध का स्वाद अच्छा है। आपके लिए यह कथन कितना महत्वपूर्ण है।

- अत्यंत महत्वपूर्ण
- बहुत महत्वपूर्ण
- मामूली रूप से महत्वपूर्ण
- थोड़ा महत्वपूर्ण
- कतई महत्वपूर्ण नहीं

जब आप दूध खाने के बारे में सोचते हैं।

क्योंकि दूध मुझे तृप्त करता है। आपके लिए यह कथन कितना महत्वपूर्ण है।

- अत्यंत महत्वपूर्ण
- बहुत महत्वपूर्ण
- मामूली रूप से महत्वपूर्ण
- थोड़ा महत्वपूर्ण
- कतई महत्वपूर्ण नहीं

जब आप दूध खाने के बारे में सोचते हैं।

क्योंकि दूध प्राकृतिक है। आपके लिए यह कथन कितना महत्वपूर्ण है।

- अत्यंत महत्वपूर्ण
- बहुत महत्वपूर्ण
- मामूली रूप से महत्वपूर्ण
- थोड़ा महत्वपूर्ण
- कतई महत्वपूर्ण नहीं

जब आप दूध खाने के बारे में सोचते हैं।

क्योंकि आप अधिक पैसे खर्च नहीं करना चाहते है। आपके लिए यह कथन कितना महत्वपूर्ण है।

- अत्यंत महत्वपूर्ण
- बहुत महत्वपूर्ण
- मामूली रूप से महत्वपूर्ण
- थोड़ा महत्वपूर्ण
- कतई महत्वपूर्ण नहीं

जब आप दूध खाने के बारे में सोचते हैं।

क्योंकि दूध में कम कैलोरीज होती हैं। आपके लिए यह कथन कितना महत्वपूर्ण है।

- अत्यंत महत्वपूर्ण
- बहुत महत्वपूर्ण
- मामूली रूप से महत्वपूर्ण
- थोड़ा महत्वपूर्ण
- कतई महत्वपूर्ण नहीं

जब आप दूध खाने के बारे में सोचते हैं।

क्योंकि आप निराश रहते हैं। आपके लिए यह कथन कितना महत्वपूर्ण है।

- अत्यंत महत्वपूर्ण
- बहुत महत्वपूर्ण
- मामूली रूप से महत्वपूर्ण
- थोड़ा महत्वपूर्ण
- कतई महत्वपूर्ण नहीं

जब आप दूध खाने के बारे में सोचते हैं।

क्योंकि दूध खाना आपको दूसरों के सामने अच्छा दिखाता हैं। आपके लिए यह कथन कितना महत्वपूर्ण है।

- अत्यंत महत्वपूर्ण
- बहुत महत्वपूर्ण
- मामूली रूप से महत्वपूर्ण
- थोड़ा महत्वपूर्ण
- कतई महत्वपूर्ण नहीं

जब आप दूध खाने के बारे में सोचते हैं।

क्योंकि दूध नहीं खाना अशिष्ट होता है। आपके लिए यह कथन कितना महत्वपूर्ण है।

- अत्यंत महत्वपूर्ण
- बहुत महत्वपूर्ण
- मामूली रूप से महत्वपूर्ण
- थोड़ा महत्वपूर्ण
- कतई महत्वपूर्ण नहीं

जब आप दूध खाने के बारे में सोचते हैं।

क्योंकि दूध कि प्रस्तुति आकर्षक होती है। आपके लिए यह कथन कितना महत्वपूर्ण है।

- अत्यंत महत्वपूर्ण
- बहुत महत्वपूर्ण
- मामूली रूप से महत्वपूर्ण
- थोड़ा महत्वपूर्ण
- कतई महत्वपूर्ण नहीं

जब आप दूध खाने के बारे में सोचते हैं।

ताकि आप अन्य लोगों के साथ समय बिता सके। आपके लिए यह कथन कितना महत्वपूर्ण है।

- अत्यंत महत्वपूर्ण
- बहुत महत्वपूर्ण
- मामूली रूप से महत्वपूर्ण
- थोड़ा महत्वपूर्ण
- कतई महत्वपूर्ण नहीं

जब आप दूध खाने के बारे में सोचते हैं।

क्योंकि दूध जैविक होता है। आपके लिए यह कथन कितना महत्वपूर्ण है।

- अत्यंत महत्वपूर्ण
- बहुत महत्वपूर्ण
- मामूली रूप से महत्वपूर्ण
- थोड़ा महत्वपूर्ण
- कतई महत्वपूर्ण नहीं

जब आप दूध खाने के बारे में सोचते हैं।

क्योंकि आप बचपन से दूध खाते रहे है। आपके लिए यह कथन कितना महत्वपूर्ण है।

- अत्यंत महत्वपूर्ण
- बहुत महत्वपूर्ण
- मामूली रूप से महत्वपूर्ण
- थोड़ा महत्वपूर्ण
- कतई महत्वपूर्ण नहीं

जब आप दूध खाने के बारे में सोचते हैं।

क्योंकि आप दूध खाने में आनंद महसूस करते है। आपके लिए यह कथन कितना महत्वपूर्ण है।

- अत्यंत महत्वपूर्ण
- बहुत महत्वपूर्ण
- मामूली रूप से महत्वपूर्ण
- थोड़ा महत्वपूर्ण
- कतई महत्वपूर्ण नहीं

जब आप दूध खाने के बारे में सोचते हैं।

क्योंकि आप भूखे रहते हैं। आपके लिए यह कथन कितना महत्वपूर्ण है।

- अत्यंत महत्वपूर्ण

- बहुत महत्वपूर्ण
- मामूली रूप से महत्वपूर्ण
- थोड़ा महत्वपूर्ण
- कतई महत्वपूर्ण नहीं

जब आप दूध खाने के बारे में सोचते हैं।

क्योंकि आप आमतौर पर दूध खाते है। आपके लिए यह कथन कितना महत्वपूर्ण है।

- अत्यंत महत्वपूर्ण
- बहुत महत्वपूर्ण
- मामूली रूप से महत्वपूर्ण
- थोड़ा महत्वपूर्ण
- कतई महत्वपूर्ण नहीं

जब आप दूध खाने के बारे में सोचते हैं।

क्योंकि आपके भीतर दूध खाने कि भूख रहती है। आपके लिए यह कथन कितना महत्वपूर्ण है।

- अत्यंत महत्वपूर्ण
- बहुत महत्वपूर्ण
- मामूली रूप से महत्वपूर्ण
- थोड़ा महत्वपूर्ण
- कतई महत्वपूर्ण नहीं

जब आप दूध खाने के बारे में सोचते हैं।

क्योंकि दूध तैयार करना आसान है। आपके लिए यह कथन कितना महत्वपूर्ण है।

- अत्यंत महत्वपूर्ण
- बहुत महत्वपूर्ण

- मामूली रूप से महत्वपूर्ण
- थोड़ा महत्वपूर्ण
- कतई महत्वपूर्ण नहीं

जब आप दूध खाने के बारे में सोचते हैं।

क्योंकि आप उदास रहते है। आपके लिए यह कथन कितना महत्वपूर्ण है।

- अत्यंत महत्वपूर्ण
- बहुत महत्वपूर्ण
- मामूली रूप से महत्वपूर्ण
- थोड़ा महत्वपूर्ण
- कतई महत्वपूर्ण नहीं

जब आप दूध खाने के बारे में सोचते हैं।

क्योंकि दूध में वसा कि मात्रा कम होती है। आपके लिए यह कथन कितना महत्वपूर्ण है।

- अत्यंत महत्वपूर्ण
- बहुत महत्वपूर्ण
- मामूली रूप से महत्वपूर्ण
- थोड़ा महत्वपूर्ण
- कतई महत्वपूर्ण नहीं

जब आप दूध खाने के बारे में सोचते हैं।

क्योंकि आप दूध को टीवी या विज्ञापन पर देखते है। आपके लिए यह कथन कितना महत्वपूर्ण है।

- अत्यंत महत्वपूर्ण
- बहुत महत्वपूर्ण
- मामूली रूप से महत्वपूर्ण
- थोड़ा महत्वपूर्ण

- कतई महत्वपूर्ण नहीं

जब आप दूध खाने के बारे में सोचते हैं।

क्योंकि दूध सामाजिक समारोहों को और अधिक आरामदायक बनाता है। आपके लिए यह कथन कितना महत्वपूर्ण है।

- अत्यंत महत्वपूर्ण
- बहुत महत्वपूर्ण
- मामूली रूप से महत्वपूर्ण
- थोड़ा महत्वपूर्ण
- कतई महत्वपूर्ण नहीं

जब आप दूध खाने के बारे में सोचते हैं।

क्योंकि आपको दूध खाना पड़ता है। आपके लिए यह कथन कितना महत्वपूर्ण है।

- अत्यंत महत्वपूर्ण
- बहुत महत्वपूर्ण
- मामूली रूप से महत्वपूर्ण
- थोड़ा महत्वपूर्ण
- कतई महत्वपूर्ण नहीं

जब आप दूध खाने के बारे में सोचते हैं।

क्योंकि दूध दूसरों को पसंद है। आपके लिए यह कथन कितना महत्वपूर्ण है।

- अत्यंत महत्वपूर्ण
- बहुत महत्वपूर्ण
- मामूली रूप से महत्वपूर्ण
- थोड़ा महत्वपूर्ण
- कतई महत्वपूर्ण नहीं

जब आप दूध खाने के बारे में सोचते हैं।

क्योंकि आप अकेला महसूस करते हैं। आपके लिए यह कथन कितना महत्वपूर्ण है।

- अत्यंत महत्वपूर्ण
- बहुत महत्वपूर्ण
- मामूली रूप से महत्वपूर्ण
- थोड़ा महत्वपूर्ण
- कतई महत्वपूर्ण नहीं

जब आप दूध खाने के बारे में सोचते हैं।

क्योंकि आप अपने वजन पर निगरानी रखते हैं।   आपके लिए यह कथन कितना महत्वपूर्ण है।

- अत्यंत महत्वपूर्ण
- बहुत महत्वपूर्ण
- मामूली रूप से महत्वपूर्ण
- थोड़ा महत्वपूर्ण
- कतई महत्वपूर्ण नहीं

जब आप दूध खाने के बारे में सोचते हैं।

क्योंकि दूध में कोई हानिकारक (कीटनाशक, प्रदूषक, एंटीबायोटिक) पदार्थ नहीं होता है।   आपके लिए यह कथन कितना महत्वपूर्ण है।

- अत्यंत महत्वपूर्ण
- बहुत महत्वपूर्ण
- मामूली रूप से महत्वपूर्ण
- थोड़ा महत्वपूर्ण
- कतई महत्वपूर्ण नहीं

जब आप दूध खाने के बारे में सोचते हैं।

क्योंकि दूध बनाना सुविधाजनक है। आपके लिए यह कथन कितना महत्वपूर्ण है।

- अत्यंत महत्वपूर्ण
- बहुत महत्वपूर्ण
- मामूली रूप से महत्वपूर्ण
- थोड़ा महत्वपूर्ण
- कतई महत्वपूर्ण नहीं

जब आप दूध खाने के बारे में सोचते हैं।

क्योंकि मूझे ऊर्जा की जरूरत होती है। आपके लिए यह कथन कितना महत्वपूर्ण है।

- अत्यंत महत्वपूर्ण
- बहुत महत्वपूर्ण
- मामूली रूप से महत्वपूर्ण
- थोड़ा महत्वपूर्ण
- कतई महत्वपूर्ण नहीं

जब आप दूध खाने के बारे में सोचते हैं।

क्योंकि आप दूध से परिचित है। आपके लिए यह कथन कितना महत्वपूर्ण है।

- अत्यंत महत्वपूर्ण
- बहुत महत्वपूर्ण
- मामूली रूप से महत्वपूर्ण
- थोड़ा महत्वपूर्ण
- कतई महत्वपूर्ण नहीं

जब आप दूध खाने के बारे में सोचते हैं।

क्योंकि आपको दूध पसंद है। आपके लिए यह कथन कितना महत्वपूर्ण है।

- अत्यंत महत्वपूर्ण
- बहुत महत्वपूर्ण
- मामूली रूप से महत्वपूर्ण
- थोड़ा महत्वपूर्ण
- कतई महत्वपूर्ण नहीं

जब आप दूध खाने के बारे में सोचते हैं।

क्योंकि दूध खाना सुखदेय है। आपके लिए यह कथन कितना महत्वपूर्ण है।

- अत्यंत महत्वपूर्ण
- बहुत महत्वपूर्ण
- मामूली रूप से महत्वपूर्ण
- थोड़ा महत्वपूर्ण
- कतई महत्वपूर्ण नहीं

जब आप दूध खाने के बारे में सोचते हैं।

आप दूध अपने आपको पुरस्कृत करने के लिए दूध खाते हैं। आपके लिए यह कथन कितना महत्वपूर्ण है।

- अत्यंत महत्वपूर्ण
- बहुत महत्वपूर्ण
- मामूली रूप से महत्वपूर्ण
- थोड़ा महत्वपूर्ण
- कतई महत्वपूर्ण नहीं

जब आप दूध खाने के बारे में सोचते हैं।

क्योंकि दूध खाना आपकी परंपरा का हिस्सा है। आपके लिए यह कथन कितना महत्वपूर्ण है।

- अत्यंत महत्वपूर्ण
- बहुत महत्वपूर्ण
- मामूली रूप से महत्वपूर्ण
- थोड़ा महत्वपूर्ण
- कतई महत्वपूर्ण नहीं

जब आप दूध खाने के बारे में सोचते हैं।

क्योंकि यह कुछ स्थितियों से संबंधित है।   आपके लिए यह कथन कितना महत्वपूर्ण है।

- अत्यंत महत्वपूर्ण
- बहुत महत्वपूर्ण
- मामूली रूप से महत्वपूर्ण
- थोड़ा महत्वपूर्ण
- कतई महत्वपूर्ण नहीं

जब आप दूध खाने के बारे में सोचते हैं।

आप दूध संतुलित आहार पाने के लिए खाते हैं। आपके लिए यह कथन कितना महत्वपूर्ण है।

- अत्यंत महत्वपूर्ण
- बहुत महत्वपूर्ण
- मामूली रूप से महत्वपूर्ण
- थोड़ा महत्वपूर्ण
- कतई महत्वपूर्ण नहीं

जब आप दूध खाने के बारे में सोचते हैं।

क्योंकि दूध खाना सामाजिक है। आपके लिए यह कथन कितना महत्वपूर्ण है।

- अत्यंत महत्वपूर्ण

- बहुत महत्वपूर्ण
- मामूली रूप से महत्वपूर्ण
- थोड़ा महत्वपूर्ण
- कतई महत्वपूर्ण नहीं

जब आप दूध खाने के बारे में सोचते हैं।

क्योंकि दूध सस्ता है। आपके लिए यह कथन कितना महत्वपूर्ण है।

- अत्यंत महत्वपूर्ण
- बहुत महत्वपूर्ण
- मामूली रूप से महत्वपूर्ण
- थोड़ा महत्वपूर्ण
- कतई महत्वपूर्ण नहीं

जब आप दूध खाने के बारे में सोचते हैं।

क्योंकि दूध आपके लिए आकर्षक भोजन है। आपके लिए यह कथन कितना महत्वपूर्ण है।

- अत्यंत महत्वपूर्ण
- बहुत महत्वपूर्ण
- मामूली रूप से महत्वपूर्ण
- थोड़ा महत्वपूर्ण
- कतई महत्वपूर्ण नहीं

जब आप दूध खाने के बारे में सोचते हैं।

दुसरो को निराश करने से बचने के लिए आप दूध खाते हैं। आपके लिए यह कथन कितना महत्वपूर्ण है।

- अत्यंत महत्वपूर्ण
- बहुत महत्वपूर्ण

- मामूली रूप से महत्वपूर्ण
- थोड़ा महत्वपूर्ण
- कतई महत्वपूर्ण नहीं

जब आप दूध खाने के बारे में सोचते हैं।

क्योंकि दूध खाना फैशनेबल है। आपके लिए यह कथन कितना महत्वपूर्ण है।

- अत्यंत महत्वपूर्ण
- बहुत महत्वपूर्ण
- मामूली रूप से महत्वपूर्ण
- थोड़ा महत्वपूर्ण
- कतई महत्वपूर्ण नहीं

जब आप दूध खाने के बारे में सोचते हैं।

क्योंकि दूध सेल पर उपलब्ध है। आपके लिए यह कथन कितना महत्वपूर्ण है।

- अत्यंत महत्वपूर्ण
- बहुत महत्वपूर्ण
- मामूली रूप से महत्वपूर्ण
- थोड़ा महत्वपूर्ण
- कतई महत्वपूर्ण नहीं

जब आप दूध खाने के बारे में सोचते हैं।

क्योंकि दूध आपके शरीर को आकार में रखता है। आपके लिए यह कथन कितना महत्वपूर्ण है।

- अत्यंत महत्वपूर्ण
- बहुत महत्वपूर्ण
- मामूली रूप से महत्वपूर्ण

- थोड़ा महत्वपूर्ण
- कतई महत्वपूर्ण नहीं

जब आप दूध खाने के बारे में सोचते हैं।

क्योंकि दूध ही एकमात्र विकल्प था। आपके लिए यह कथन कितना महत्वपूर्ण है।

- अत्यंत महत्वपूर्ण
- बहुत महत्वपूर्ण
- मामूली रूप से महत्वपूर्ण
- थोड़ा महत्वपूर्ण
- कतई महत्वपूर्ण नहीं

जब आप दूध खाने के बारे में सोचते हैं।

क्योंकि आप दूध हर दिन खाना चाहते है। आपके लिए यह कथन कितना महत्वपूर्ण है।

- अत्यंत महत्वपूर्ण
- बहुत महत्वपूर्ण
- मामूली रूप से महत्वपूर्ण
- थोड़ा महत्वपूर्ण
- कतई महत्वपूर्ण नहीं

जब आप दूध नहीं खाने के बारे में सोचते हैं।

क्योंकि आपको नहीं लगता कि दूध एक नाश्ता है। आपके लिए यह कथन कितना महत्वपूर्ण है।

- अत्यंत महत्वपूर्ण
- बहुत महत्वपूर्ण
- मामूली रूप से महत्वपूर्ण
- थोड़ा महत्वपूर्ण

- कतई महत्वपूर्ण नहीं

जब आप दूध नहीं खाने के बारे में सोचते हैं।

क्योंकि आपको दूध पसंद नहीं है। आपके लिए यह कथन कितना महत्वपूर्ण है।

- अत्यंत महत्वपूर्ण
- बहुत महत्वपूर्ण
- मामूली रूप से महत्वपूर्ण
- थोड़ा महत्वपूर्ण
- कतई महत्वपूर्ण नहीं

जब आप दूध नहीं खाने के बारे में सोचते हैं।

क्योंकि आप आमतौर पर दूध नहीं खाते है। आपके लिए यह कथन कितना महत्वपूर्ण है।

- अत्यंत महत्वपूर्ण
- बहुत महत्वपूर्ण
- मामूली रूप से महत्वपूर्ण
- थोड़ा महत्वपूर्ण
- कतई महत्वपूर्ण नहीं

जब आप दूध नहीं खाने के बारे में सोचते हैं।

क्योंकि दूध कि खुराक का आकार मेरे लिए उपयुक्त नहीं होता है। आपके लिए यह कथन कितना महत्वपूर्ण है।

- अत्यंत महत्वपूर्ण
- बहुत महत्वपूर्ण
- मामूली रूप से महत्वपूर्ण
- थोड़ा महत्वपूर्ण
- कतई महत्वपूर्ण नहीं

जब आप दूध नहीं खाने के बारे में सोचते हैं।

क्योंकि दूध आपकी भूख मिटाने के लिए पर्याप्त नहीं होता है। आपके लिए यह कथन कितना महत्वपूर्ण है।

- अत्यंत महत्वपूर्ण
- बहुत महत्वपूर्ण
- मामूली रूप से महत्वपूर्ण
- थोड़ा महत्वपूर्ण
- कतई महत्वपूर्ण नहीं

जब आप दूध नहीं खाने के बारे में सोचते हैं।

क्योंकि दूध स्वास्थ्यय के लिए लाभदायक नहीं है। आपके लिए यह कथन कितना महत्वपूर्ण है।

- अत्यंत महत्वपूर्ण
- बहुत महत्वपूर्ण
- मामूली रूप से महत्वपूर्ण
- थोड़ा महत्वपूर्ण
- कतई महत्वपूर्ण नहीं

जब आप दूध नहीं खाने के बारे में सोचते हैं।

क्योंकि दूध बनाना मुश्किल होता है। आपके लिए यह कथन कितना महत्वपूर्ण है।

- अत्यंत महत्वपूर्ण
- बहुत महत्वपूर्ण
- मामूली रूप से महत्वपूर्ण
- थोड़ा महत्वपूर्ण
- कतई महत्वपूर्ण नहीं

जब आप दूध नहीं खाने के बारे में सोचते हैं।

क्योंकि दूध आपकी भूख को तृप्त नहीं कर पाता है। आपके लिए यह कथन कितना महत्वपूर्ण है।

- अत्यंत महत्वपूर्ण
- बहुत महत्वपूर्ण
- मामूली रूप से महत्वपूर्ण
- थोड़ा महत्वपूर्ण
- कतई महत्वपूर्ण नहीं

जब आप दूध नहीं खाने के बारे में सोचते हैं।

क्योंकि दूध स्थिति के लिए उपयुक्त नहीं होता है। आपके लिए यह कथन कितना महत्वपूर्ण है।

- अत्यंत महत्वपूर्ण
- बहुत महत्वपूर्ण
- मामूली रूप से महत्वपूर्ण
- थोड़ा महत्वपूर्ण
- कतई महत्वपूर्ण नहीं

जब आप दूध नहीं खाने के बारे में सोचते हैं।

क्योंकि दूध अन्य लोगों के आसपास खाने लायक भोजन नहीं है। आपके लिए यह कथन कितना महत्वपूर्ण है।

- अत्यंत महत्वपूर्ण
- बहुत महत्वपूर्ण
- मामूली रूप से महत्वपूर्ण
- थोड़ा महत्वपूर्ण
- कतई महत्वपूर्ण नहीं

जब आप दूध नहीं खाने के बारे में सोचते हैं।

क्योंकि दूध कि कीमत बहुत अधिक है। आपके लिए यह कथन कितना महत्वपूर्ण है।

- अत्यंत महत्वपूर्ण
- बहुत महत्वपूर्ण
- मामूली रूप से महत्वपूर्ण
- थोड़ा महत्वपूर्ण
- कतई महत्वपूर्ण नहीं

जब आप दूध नहीं खाने के बारे में सोचते हैं।

क्योंकि दूध आपको दिखने में अच्छा नहीं लगता है। आपके लिए यह कथन कितना महत्वपूर्ण है।

- अत्यंत महत्वपूर्ण
- बहुत महत्वपूर्ण
- मामूली रूप से महत्वपूर्ण
- थोड़ा महत्वपूर्ण
- कतई महत्वपूर्ण नहीं

जब आप दूध नहीं खाने के बारे में सोचते हैं।

क्योंकि दूध मे कैलोरी के मात्रा बहुत अधिक होती है। आपके लिए यह कथन कितना महत्वपूर्ण है।

- अत्यंत महत्वपूर्ण
- बहुत महत्वपूर्ण
- मामूली रूप से महत्वपूर्ण
- थोड़ा महत्वपूर्ण
- कतई महत्वपूर्ण नहीं

जब आप दूध नहीं खाने के बारे में सोचते हैं।

क्योंकि आप दूध नहीं खाना चाहते है। आपके लिए यह कथन कितना महत्वपूर्ण है।

- अत्यंत महत्वपूर्ण
- बहुत महत्वपूर्ण
- मामूली रूप से महत्वपूर्ण
- थोड़ा महत्वपूर्ण
- कतई महत्वपूर्ण नहीं

जब आप दूध नहीं खाने के बारे में सोचते हैं।

क्योंकि आप दूध खाने से दूसरों के सामने पिछड़ा महसूस करते है। आपके लिए यह कथन कितना महत्वपूर्ण है।

- अत्यंत महत्वपूर्ण
- बहुत महत्वपूर्ण
- मामूली रूप से महत्वपूर्ण
- थोड़ा महत्वपूर्ण
- कतई महत्वपूर्ण नहीं

जब आप दूध नहीं खाने के बारे में सोचते हैं।

क्योंकि आपकी दूध खाने कि इच्छा नहीं होती है। आपके लिए यह कथन कितना महत्वपूर्ण है।

- अत्यंत महत्वपूर्ण
- बहुत महत्वपूर्ण
- मामूली रूप से महत्वपूर्ण
- थोड़ा महत्वपूर्ण
- कतई महत्वपूर्ण नहीं

जब आप दूध नहीं खाने के बारे में सोचते हैं।

क्योंकि अपने हाल ही में दूध खाया था और अब आप बार-बार दूध नहीं खाना चाहते है। आपके लिए यह कथन कितना महत्वपूर्ण है।

- अत्यंत महत्वपूर्ण

- बहुत महत्वपूर्ण
- मामूली रूप से महत्वपूर्ण
- थोड़ा महत्वपूर्ण
- कतई महत्वपूर्ण नहीं

जब आप दूध नहीं खाने के बारे में सोचते हैं।

क्योंकि आपको हर दिन एक ही प्रकार का भोजन खाना पसंद है। आपके लिए यह कथन कितना महत्वपूर्ण है।

- अत्यंत महत्वपूर्ण
- बहुत महत्वपूर्ण
- मामूली रूप से महत्वपूर्ण
- थोड़ा महत्वपूर्ण
- कतई महत्वपूर्ण नहीं

जब आप दूध नहीं खाने के बारे में सोचते हैं।

क्योंकि दूध खाना आपको दुखी, अकेला और कुंठित महसूस कराता है। आपके लिए यह कथन कितना महत्वपूर्ण है।

- अत्यंत महत्वपूर्ण
- बहुत महत्वपूर्ण
- मामूली रूप से महत्वपूर्ण
- थोड़ा महत्वपूर्ण
- कतई महत्वपूर्ण नहीं

जब आप दूध नहीं खाने के बारे में सोचते हैं।

क्योंकि दूध जैविक (आर्गेनिक) नहीं है। आपके लिए यह कथन कितना महत्वपूर्ण है।

- अत्यंत महत्वपूर्ण
- बहुत महत्वपूर्ण

- मामूली रूप से महत्वपूर्ण
- थोड़ा महत्वपूर्ण
- कतई महत्वपूर्ण नहीं

Reasons for Fruit Choices(Full RATA)

क्या आप केले खाते हैं।

- हाँ
- नहीं

जब आप केले खाने के बारे में सोचते हैं।

क्योंकि आप केले खाने के आदी हैं। आपके लिए यह कथन कितना महत्वपूर्ण है।

- अत्यंत महत्वपूर्ण
- बहुत महत्वपूर्ण
- मामूली रूप से महत्वपूर्ण
- थोड़ा महत्वपूर्ण
- कतई महत्वपूर्ण नहीं

जब आप केले खाने के बारे में सोचते हैं।

क्योंकि केले खाना स्वास्थ्य के लिए लाभदायक हैं। आपके लिए यह कथन कितना महत्वपूर्ण है।

- अत्यंत महत्वपूर्ण
- बहुत महत्वपूर्ण
- मामूली रूप से महत्वपूर्ण
- थोड़ा महत्वपूर्ण
- कतई महत्वपूर्ण नहीं

जब आप केले खाने के बारे में सोचते हैं।

क्योंकि केले कम समय में बन जाता है। आपके लिए यह कथन कितना महत्वपूर्ण है।

- अत्यंत महत्वपूर्ण
- बहुत महत्वपूर्ण
- मामूली रूप से महत्वपूर्ण
- थोड़ा महत्वपूर्ण
- कतई महत्वपूर्ण नहीं

जब आप केले खाने के बारे में सोचते हैं।

क्योंकि केले का स्वाद अच्छा है। आपके लिए यह कथन कितना महत्वपूर्ण है।

- अत्यंत महत्वपूर्ण
- बहुत महत्वपूर्ण
- मामूली रूप से महत्वपूर्ण
- थोड़ा महत्वपूर्ण
- कतई महत्वपूर्ण नहीं

जब आप केले खाने के बारे में सोचते हैं।

क्योंकि केले मुझे तृप्त करते है। आपके लिए यह कथन कितना महत्वपूर्ण है।

- अत्यंत महत्वपूर्ण
- बहुत महत्वपूर्ण
- मामूली रूप से महत्वपूर्ण
- थोड़ा महत्वपूर्ण
- कतई महत्वपूर्ण नहीं

जब आप केले खाने के बारे में सोचते हैं।

क्योंकि केले प्राकृतिक है। आपके लिए यह कथन कितना महत्वपूर्ण है।

- अत्यंत महत्वपूर्ण
- बहुत महत्वपूर्ण
- मामूली रूप से महत्वपूर्ण
- थोड़ा महत्वपूर्ण
- कतई महत्वपूर्ण नहीं

जब आप केले खाने के बारे में सोचते हैं।

क्योंकि आप अधिक पैसे खर्च नहीं करना चाहते। आपके लिए यह कथन कितना महत्वपूर्ण है।

- अत्यंत महत्वपूर्ण
- बहुत महत्वपूर्ण
- मामूली रूप से महत्वपूर्ण
- थोड़ा महत्वपूर्ण
- कतई महत्वपूर्ण नहीं

जब आप केले खाने के बारे में सोचते हैं।

क्योंकि केले में कम कैलोरीज होती हैं। आपके लिए यह कथन कितना महत्वपूर्ण है।

- अत्यंत महत्वपूर्ण
- बहुत महत्वपूर्ण
- मामूली रूप से महत्वपूर्ण
- थोड़ा महत्वपूर्ण
- कतई महत्वपूर्ण नहीं

जब आप केले खाने के बारे में सोचते हैं।

[illegible]

क्योंकि आप निराश रहते हैं। आपके लिए यह कथन कितना महत्वपूर्ण है।

- अत्यंत महत्वपूर्ण
- बहुत महत्वपूर्ण
- मामूली रूप से महत्वपूर्ण
- थोड़ा महत्वपूर्ण
- कतई महत्वपूर्ण नहीं

जब आप केले खाने के बारे में सोचते हैं।

क्योंकि केले खाना आपको दूसरों के सामने अच्छा दिखाता हैं। आपके लिए यह कथन कितना महत्वपूर्ण है।

- अत्यंत महत्वपूर्ण
- बहुत महत्वपूर्ण
- मामूली रूप से महत्वपूर्ण
- थोड़ा महत्वपूर्ण
- कतई महत्वपूर्ण नहीं

जब आप केले खाने के बारे में सोचते हैं।

क्योंकि केले नहीं खाना अशिष्ट होता है। आपके लिए यह कथन कितना महत्वपूर्ण है।

- अत्यंत महत्वपूर्ण
- बहुत महत्वपूर्ण
- मामूली रूप से महत्वपूर्ण
- थोड़ा महत्वपूर्ण
- कतई महत्वपूर्ण नहीं

जब आप केले खाने के बारे में सोचते हैं।

[illegible]

क्योंकि केले कि प्रस्तुति आकर्षक होती है। आपके लिए यह कथन कितना महत्वपूर्ण है।

- अत्यंत महत्वपूर्ण
- बहुत महत्वपूर्ण
- मामूली रूप से महत्वपूर्ण
- थोड़ा महत्वपूर्ण
- कतई महत्वपूर्ण नहीं

जब आप केले खाने के बारे में सोचते हैं।

ताकि आप अन्य लोगों के साथ समय बिता सके। आपके लिए यह कथन कितना महत्वपूर्ण है।

- अत्यंत महत्वपूर्ण
- बहुत महत्वपूर्ण
- मामूली रूप से महत्वपूर्ण
- थोड़ा महत्वपूर्ण
- कतई महत्वपूर्ण नहीं

जब आप केले खाने के बारे में सोचते हैं।

क्योंकि केले जैविक होते है। आपके लिए यह कथन कितना महत्वपूर्ण है।

- अत्यंत महत्वपूर्ण
- बहुत महत्वपूर्ण
- मामूली रूप से महत्वपूर्ण
- थोड़ा महत्वपूर्ण
- कतई महत्वपूर्ण नहीं

जब आप केले खाने के बारे में सोचते हैं।

[illegible]

क्योंकि आप बचपन से केले खाते रहे हैं। आपके लिए यह कथन कितना महत्वपूर्ण है।

- अत्यंत महत्वपूर्ण
- बहुत महत्वपूर्ण
- मामूली रूप से महत्वपूर्ण
- थोड़ा महत्वपूर्ण
- कतई महत्वपूर्ण नहीं

जब आप केले खाने के बारे में सोचते हैं।

क्योंकि आप केले खाने में आनंद महसूस करते है। आपके लिए यह कथन कितना महत्वपूर्ण है।

- अत्यंत महत्वपूर्ण
- बहुत महत्वपूर्ण
- मामूली रूप से महत्वपूर्ण
- थोड़ा महत्वपूर्ण
- कतई महत्वपूर्ण नहीं

जब आप केले खाने के बारे में सोचते हैं।

क्योंकि आप भूखे रहते है। आपके लिए यह कथन कितना महत्वपूर्ण है।

- अत्यंत महत्वपूर्ण
- बहुत महत्वपूर्ण
- मामूली रूप से महत्वपूर्ण
- थोड़ा महत्वपूर्ण
- कतई महत्वपूर्ण नहीं

जब आप केले खाने के बारे में सोचते हैं।

क्योंकि आप आमतौर पर केले खाते है। आपके लिए यह कथन कितना महत्वपूर्ण है।

- अत्यंत महत्वपूर्ण
- बहुत महत्वपूर्ण
- मामूली रूप से महत्वपूर्ण
- थोड़ा महत्वपूर्ण
- कतई महत्वपूर्ण नहीं

जब आप केले खाने के बारे में सोचते हैं।

क्योंकि आपके भीतर केले खाने कि भूख रहती है। आपके लिए यह कथन कितना महत्वपूर्ण है।

- अत्यंत महत्वपूर्ण
- बहुत महत्वपूर्ण
- मामूली रूप से महत्वपूर्ण
- थोड़ा महत्वपूर्ण
- कतई महत्वपूर्ण नहीं

जब आप केले खाने के बारे में सोचते हैं।

क्योंकि केले तैयार करना आसान है। आपके लिए यह कथन कितना महत्वपूर्ण है।

- अत्यंत महत्वपूर्ण
- बहुत महत्वपूर्ण
- मामूली रूप से महत्वपूर्ण
- थोड़ा महत्वपूर्ण
- कतई महत्वपूर्ण नहीं

जब आप केले खाने के बारे में सोचते हैं।

क्योंकि आप उदास रहते है। आपके लिए यह कथन कितना महत्वपूर्ण है।

- अत्यंत महत्वपूर्ण

- बहुत महत्वपूर्ण
- मामूली रूप से महत्वपूर्ण
- थोड़ा महत्वपूर्ण
- कतई महत्वपूर्ण नहीं

जब आप केले खाने के बारे में सोचते हैं।

क्योंकि केले में वसा कि मात्रा कम होती है। आपके लिए यह कथन कितना महत्वपूर्ण है।

- अत्यंत महत्वपूर्ण
- बहुत महत्वपूर्ण
- मामूली रूप से महत्वपूर्ण
- थोड़ा महत्वपूर्ण
- कतई महत्वपूर्ण नहीं

जब आप केले खाने के बारे में सोचते हैं।

क्योंकि आप केले को टीवी या विज्ञापन पर देखते है। आपके लिए यह कथन कितना महत्वपूर्ण है।

- अत्यंत महत्वपूर्ण
- बहुत महत्वपूर्ण
- मामूली रूप से महत्वपूर्ण
- थोड़ा महत्वपूर्ण
- कतई महत्वपूर्ण नहीं

जब आप केले खाने के बारे में सोचते हैं।

क्योंकि केले सामाजिक समारोहों को अधिक आरामदायक बनाता है। आपके लिए यह कथन कितना महत्वपूर्ण है।

- अत्यंत महत्वपूर्ण
- बहुत महत्वपूर्ण

- मामूली रूप से महत्वपूर्ण
- थोड़ा महत्वपूर्ण
- कतई महत्वपूर्ण नहीं

जब आप केले खाने के बारे में सोचते हैं।

क्योंकि आपको केले खाने पड़ते है। आपके लिए यह कथन कितना महत्वपूर्ण है।

- अत्यंत महत्वपूर्ण
- बहुत महत्वपूर्ण
- मामूली रूप से महत्वपूर्ण
- थोड़ा महत्वपूर्ण
- कतई महत्वपूर्ण नहीं

जब आप केले खाने के बारे में सोचते हैं।

क्योंकि केले दूसरों को पसंद है। आपके लिए यह कथन कितना महत्वपूर्ण है।

- अत्यंत महत्वपूर्ण
- बहुत महत्वपूर्ण
- मामूली रूप से महत्वपूर्ण
- थोड़ा महत्वपूर्ण
- कतई महत्वपूर्ण नहीं

जब आप केले खाने के बारे में सोचते हैं।

क्योंकि आप अकेला महसूस करते हैं। आपके लिए यह कथन कितना महत्वपूर्ण है।

- अत्यंत महत्वपूर्ण
- बहुत महत्वपूर्ण
- मामूली रूप से महत्वपूर्ण

- थोड़ा महत्वपूर्ण
- कतई महत्वपूर्ण नहीं

जब आप केले खाने के बारे में सोचते हैं।

क्योंकि आप अपने वजन पर निगरानी रखते हैं। आपके लिए यह कथन कितना महत्वपूर्ण है।

- अत्यंत महत्वपूर्ण
- बहुत महत्वपूर्ण
- मामूली रूप से महत्वपूर्ण
- थोड़ा महत्वपूर्ण
- कतई महत्वपूर्ण नहीं

जब आप केले खाने के बारे में सोचते हैं।

क्योंकि केले में कोई हानिकारक (कीटनाशक, प्रदूषक, एंटीबायोटिक) पदार्थ नहीं होता है। आपके लिए यह कथन कितना महत्वपूर्ण है।

- अत्यंत महत्वपूर्ण
- बहुत महत्वपूर्ण
- मामूली रूप से महत्वपूर्ण
- थोड़ा महत्वपूर्ण
- कतई महत्वपूर्ण नहीं

जब आप केले खाने के बारे में सोचते हैं।

क्योंकि केले बनाना सुविधाजनक है। आपके लिए यह कथन कितना महत्वपूर्ण है।

- अत्यंत महत्वपूर्ण
- बहुत महत्वपूर्ण
- मामूली रूप से महत्वपूर्ण
- थोड़ा महत्वपूर्ण

- कतई महत्वपूर्ण नहीं

जब आप केले खाने के बारे में सोचते हैं।

क्योंकि मूझे ऊर्जा की जरूरत होती है। आपके लिए यह कथन कितना महत्वपूर्ण है।

- अत्यंत महत्वपूर्ण
- बहुत महत्वपूर्ण
- मामूली रूप से महत्वपूर्ण
- थोड़ा महत्वपूर्ण
- कतई महत्वपूर्ण नहीं

जब आप केले खाने के बारे में सोचते हैं।

क्योंकि आप केले से परिचित है। आपके लिए यह कथन कितना महत्वपूर्ण है।

- अत्यंत महत्वपूर्ण
- बहुत महत्वपूर्ण
- मामूली रूप से महत्वपूर्ण
- थोड़ा महत्वपूर्ण
- कतई महत्वपूर्ण नहीं

जब आप केले खाने के बारे में सोचते हैं।

क्योंकि आपको केले पसंद है। आपके लिए यह कथन कितना महत्वपूर्ण है।

- अत्यंत महत्वपूर्ण
- बहुत महत्वपूर्ण
- मामूली रूप से महत्वपूर्ण
- थोड़ा महत्वपूर्ण
- कतई महत्वपूर्ण नहीं

जब आप केले खाने के बारे में सोचते हैं।

क्योंकि केले खाना सुखदेय है। आपके लिए यह कथन कितना महत्वपूर्ण है।

- अत्यंत महत्वपूर्ण
- बहुत महत्वपूर्ण
- मामूली रूप से महत्वपूर्ण
- थोड़ा महत्वपूर्ण
- कतई महत्वपूर्ण नहीं

जब आप केले खाने के बारे में सोचते हैं।

आप केले अपने आपको पुरस्कृत करने के लिए केले खाते हैं। आपके लिए यह कथन कितना महत्वपूर्ण है।

- अत्यंत महत्वपूर्ण
- बहुत महत्वपूर्ण
- मामूली रूप से महत्वपूर्ण
- थोड़ा महत्वपूर्ण
- कतई महत्वपूर्ण नहीं

जब आप केले खाने के बारे में सोचते हैं।

क्योंकि केले खाना आपकी परंपरा का हिस्सा है। आपके लिए यह कथन कितना महत्वपूर्ण है।

- अत्यंत महत्वपूर्ण
- बहुत महत्वपूर्ण
- मामूली रूप से महत्वपूर्ण
- थोड़ा महत्वपूर्ण
- कतई महत्वपूर्ण नहीं

जब आप केले खाने के बारे में सोचते हैं।

क्योंकि यह कुछ स्थितियों से संबंधित है। आपके लिए यह कथन कितना महत्वपूर्ण है।

- अत्यंत महत्वपूर्ण
- बहुत महत्वपूर्ण
- मामूली रूप से महत्वपूर्ण
- थोड़ा महत्वपूर्ण
- कतई महत्वपूर्ण नहीं

जब आप केले खाने के बारे में सोचते हैं।

क्योंकि केले संतुलित आहार बनाए रखने के लिए मह्त्वपूर्ण है। आपके लिए यह कथन कितना महत्वपूर्ण है।

- अत्यंत महत्वपूर्ण
- बहुत महत्वपूर्ण
- मामूली रूप से महत्वपूर्ण
- थोड़ा महत्वपूर्ण
- कतई महत्वपूर्ण नहीं

जब आप केले खाने के बारे में सोचते हैं।

क्योंकि केले खाना सामाजिक है। आपके लिए यह कथन कितना महत्वपूर्ण है।

- अत्यंत महत्वपूर्ण
- बहुत महत्वपूर्ण
- मामूली रूप से महत्वपूर्ण
- थोड़ा महत्वपूर्ण
- कतई महत्वपूर्ण नहीं

जब आप केले खाने के बारे में सोचते हैं।

क्योंकि केले सस्ते है। आपके लिए यह कथन कितना महत्वपूर्ण है।

- अत्यंत महत्वपूर्ण
- बहुत महत्वपूर्ण
- मामूली रूप से महत्वपूर्ण
- थोड़ा महत्वपूर्ण
- कतई महत्वपूर्ण नहीं

जब आप केले खाने के बारे में सोचते हैं।

क्योंकि केले आपके लिए आकर्षक भोजन है। आपके लिए यह कथन कितना महत्वपूर्ण है।

- अत्यंत महत्वपूर्ण
- बहुत महत्वपूर्ण
- मामूली रूप से महत्वपूर्ण
- थोड़ा महत्वपूर्ण
- कतई महत्वपूर्ण नहीं

जब आप केले खाने के बारे में सोचते हैं।

दुसरो को निराश करने से बचने के लिए आप केले खाते हैं। आपके लिए यह कथन कितना महत्वपूर्ण है।

- अत्यंत महत्वपूर्ण
- बहुत महत्वपूर्ण
- मामूली रूप से महत्वपूर्ण
- थोड़ा महत्वपूर्ण
- कतई महत्वपूर्ण नहीं

जब आप केले खाने के बारे में सोचते हैं।

[illegible]

हैं। क्योंकि केले खाना फैशनेबल है। आपके लिए यह कथन कितना महत्वपूर्ण है।

- अत्यंत महत्वपूर्ण
- बहुत महत्वपूर्ण
- मामूली रूप से महत्वपूर्ण
- थोड़ा महत्वपूर्ण
- कतई महत्वपूर्ण नहीं

जब आप केले खाने के बारे में सोचते हैं।

क्योंकि केले सेल पर उपलब्ध है। आपके लिए यह कथन कितना महत्वपूर्ण है।

- अत्यंत महत्वपूर्ण
- बहुत महत्वपूर्ण
- मामूली रूप से महत्वपूर्ण
- थोड़ा महत्वपूर्ण
- कतई महत्वपूर्ण नहीं

जब आप केले खाने के बारे में सोचते हैं।

क्योंकि केले आपके शरीर को आकार में रखता है। आपके लिए यह कथन कितना महत्वपूर्ण है।

- अत्यंत महत्वपूर्ण
- बहुत महत्वपूर्ण
- मामूली रूप से महत्वपूर्ण
- थोड़ा महत्वपूर्ण
- कतई महत्वपूर्ण नहीं

जब आप केले खाने के बारे में सोचते हैं।

क्योंकि केले ही एकमात्र विकल्प था। आपके लिए यह कथन कितना महत्वपूर्ण है।

- अत्यंत महत्वपूर्ण
- बहुत महत्वपूर्ण
- मामूली रूप से महत्वपूर्ण
- थोड़ा महत्वपूर्ण
- कतई महत्वपूर्ण नहीं

जब आप केले खाने के बारे में सोचते हैं।

क्योंकि आप केले हर दिन खाना चाहते है। आपके लिए यह कथन कितना महत्वपूर्ण है।

- अत्यंत महत्वपूर्ण
- बहुत महत्वपूर्ण
- मामूली रूप से महत्वपूर्ण
- थोड़ा महत्वपूर्ण
- कतई महत्वपूर्ण नहीं

जब आप केले नहीं खाने के बारे में सोचते हैं।

क्योंकि आपको नहीं लगता कि केले एक नाश्ता है। आपके लिए यह कथन कितना महत्वपूर्ण है।

- अत्यंत महत्वपूर्ण
- बहुत महत्वपूर्ण
- मामूली रूप से महत्वपूर्ण
- थोड़ा महत्वपूर्ण
- कतई महत्वपूर्ण नहीं

जब आप केले नहीं खाने के बारे में सोचते हैं।

क्योंकि आपको केले पसंद नहीं है। आपके लिए यह कथन कितना महत्वपूर्ण है।

- अत्यंत महत्वपूर्ण

- बहुत महत्वपूर्ण
- मामूली रूप से महत्वपूर्ण
- थोड़ा महत्वपूर्ण
- कतई महत्वपूर्ण नहीं

जब आप केले नहीं खाने के बारे में सोचते हैं।

क्योंकि आप आमतौर पर केले नहीं खाते है। आपके लिए यह कथन कितना महत्वपूर्ण है।

- अत्यंत महत्वपूर्ण
- बहुत महत्वपूर्ण
- मामूली रूप से महत्वपूर्ण
- थोड़ा महत्वपूर्ण
- कतई महत्वपूर्ण नहीं

जब आप केले नहीं खाने के बारे में सोचते हैं।

क्योंकि केले कि खुराक का आकार मेरे लिए उपयुक्त नहीं होता है। आपके लिए यह कथन कितना महत्वपूर्ण है।

- अत्यंत महत्वपूर्ण
- बहुत महत्वपूर्ण
- मामूली रूप से महत्वपूर्ण
- थोड़ा महत्वपूर्ण
- कतई महत्वपूर्ण नहीं

जब आप केले नहीं खाने के बारे में सोचते हैं।

क्योंकि केले आपकी भूख मिटाने के लिए पर्याप्त नहीं होता है। आपके लिए यह कथन कितना महत्वपूर्ण है।

- अत्यंत महत्वपूर्ण
- बहुत महत्वपूर्ण

- मामूली रूप से महत्वपूर्ण
- थोड़ा महत्वपूर्ण
- कतई महत्वपूर्ण नहीं

जब आप केले नहीं खाने के बारे में सोचते हैं।

क्योंकि केले स्वास्थ्यय के लिए लाभदायक नहीं है। आपके लिए यह कथन कितना महत्वपूर्ण है।

- अत्यंत महत्वपूर्ण
- बहुत महत्वपूर्ण
- मामूली रूप से महत्वपूर्ण
- थोड़ा महत्वपूर्ण
- कतई महत्वपूर्ण नहीं

जब आप केले नहीं खाने के बारे में सोचते हैं।

क्योंकि केले बनाना मुश्किल होता है। आपके लिए यह कथन कितना महत्वपूर्ण है।

- अत्यंत महत्वपूर्ण
- बहुत महत्वपूर्ण
- मामूली रूप से महत्वपूर्ण
- थोड़ा महत्वपूर्ण
- कतई महत्वपूर्ण नहीं

जब आप केले नहीं खाने के बारे में सोचते हैं।

क्योंकि केले आपकी भूख को तृप्त नहीं कर पाता है। आपके लिए यह कथन कितना महत्वपूर्ण है।

- अत्यंत महत्वपूर्ण
- बहुत महत्वपूर्ण
- मामूली रूप से महत्वपूर्ण

- थोड़ा महत्वपूर्ण
- कतई महत्वपूर्ण नहीं

जब आप केले नहीं खाने के बारे में सोचते हैं।

क्योंकि केले स्थिति के लिए उपयुक्त नहीं होता है। आपके लिए यह कथन कितना महत्वपूर्ण है।

- अत्यंत महत्वपूर्ण
- बहुत महत्वपूर्ण
- मामूली रूप से महत्वपूर्ण
- थोड़ा महत्वपूर्ण
- कतई महत्वपूर्ण नहीं

जब आप केले नहीं खाने के बारे में सोचते हैं।

क्योंकि केले अन्य लोगों के आसपास खाने लायक भोजन नहीं है। आपके लिए यह कथन कितना महत्वपूर्ण है।

- अत्यंत महत्वपूर्ण
- बहुत महत्वपूर्ण
- मामूली रूप से महत्वपूर्ण
- थोड़ा महत्वपूर्ण
- कतई महत्वपूर्ण नहीं

जब आप केले नहीं खाने के बारे में सोचते हैं।

क्योंकि केले कि कीमत बहुत अधिक है। आपके लिए यह कथन कितना महत्वपूर्ण है।

- अत्यंत महत्वपूर्ण
- बहुत महत्वपूर्ण
- मामूली रूप से महत्वपूर्ण
- थोड़ा महत्वपूर्ण

- कतई महत्वपूर्ण नहीं

जब आप केले नहीं खाने के बारे में सोचते हैं।

क्योंकि केले आपको दिखने में अच्छा नहीं लगता है। आपके लिए यह कथन कितना महत्वपूर्ण है।

- अत्यंत महत्वपूर्ण
- बहुत महत्वपूर्ण
- मामूली रूप से महत्वपूर्ण
- थोड़ा महत्वपूर्ण
- कतई महत्वपूर्ण नहीं

जब आप केले नहीं खाने के बारे में सोचते हैं।

क्योंकि केले मे कैलोरी के मात्रा बहुत अधिक होती है। आपके लिए यह कथन कितना महत्वपूर्ण है।

- अत्यंत महत्वपूर्ण
- बहुत महत्वपूर्ण
- मामूली रूप से महत्वपूर्ण
- थोड़ा महत्वपूर्ण
- कतई महत्वपूर्ण नहीं

जब आप केले नहीं खाने के बारे में सोचते हैं।

क्योंकि आप केले नहीं खाना चाहते है। आपके लिए यह कथन कितना महत्वपूर्ण है।

- अत्यंत महत्वपूर्ण
- बहुत महत्वपूर्ण
- मामूली रूप से महत्वपूर्ण
- थोड़ा महत्वपूर्ण
- कतई महत्वपूर्ण नहीं

जब आप केले नहीं खाने के बारे में सोचते हैं।

क्योंकि आप केले खाने से दूसरों के सामने पिछड़ा महसूस करते है। आपके लिए यह कथन कितना महत्वपूर्ण है।

- अत्यंत महत्वपूर्ण
- बहुत महत्वपूर्ण
- मामूली रूप से महत्वपूर्ण
- थोड़ा महत्वपूर्ण
- कतई महत्वपूर्ण नहीं

जब आप केले नहीं खाने के बारे में सोचते हैं।

क्योंकि आपकी केले खाने कि इच्छा नहीं होती है। आपके लिए यह कथन कितना महत्वपूर्ण है।

- अत्यंत महत्वपूर्ण
- बहुत महत्वपूर्ण
- मामूली रूप से महत्वपूर्ण
- थोड़ा महत्वपूर्ण
- कतई महत्वपूर्ण नहीं

जब आप केले नहीं खाने के बारे में सोचते हैं।

क्योंकि अपने हाल ही में केले खाया था और अब आप बार-बार केले नहीं खाना चाहते है। आपके लिए यह कथन कितना महत्वपूर्ण है।

- अत्यंत महत्वपूर्ण
- बहुत महत्वपूर्ण
- मामूली रूप से महत्वपूर्ण
- थोड़ा महत्वपूर्ण
- कतई महत्वपूर्ण नहीं

जब आप केले नहीं खाने के बारे में सोचते हैं।

क्योंकि आपको हर दिन एक ही प्रकार का भोजन खाना पसंद है। आपके लिए यह कथन कितना महत्वपूर्ण है।

- अत्यंत महत्वपूर्ण
- बहुत महत्वपूर्ण
- मामूली रूप से महत्वपूर्ण
- थोड़ा महत्वपूर्ण
- कतई महत्वपूर्ण नहीं

जब आप केले नहीं खाने के बारे में सोचते हैं।

क्योंकि केले खाना आपको दुखी, अकेला और कुंठित महसूस कराता है। आपके लिए यह कथन कितना महत्वपूर्ण है।

- अत्यंत महत्वपूर्ण
- बहुत महत्वपूर्ण
- मामूली रूप से महत्वपूर्ण
- थोड़ा महत्वपूर्ण
- कतई महत्वपूर्ण नहीं

जब आप केले नहीं खाने के बारे में सोचते हैं।

क्योंकि केले जैविक (आर्गेनिक) नहीं है। आपके लिए यह कथन कितना महत्वपूर्ण है।

- अत्यंत महत्वपूर्ण
- बहुत महत्वपूर्ण
- मामूली रूप से महत्वपूर्ण
- थोड़ा महत्वपूर्ण
- कतई महत्वपूर्ण नहीं

Reasons for Sweet food choices(Full RATA)

क्या आप गुलाब जामुन खाते हैं।

- हाँ
- नहीं

जब आप गुलाब जामुन खाने के बारे में सोचते हैं।

क्योंकि आप गुलाब जामुन खाने के आदी हैं। आपके लिए यह कथन कितना महत्वपूर्ण है।

- अत्यंत महत्वपूर्ण
- बहुत महत्वपूर्ण
- मामूली रूप से महत्वपूर्ण
- थोड़ा महत्वपूर्ण
- कतई महत्वपूर्ण नहीं

जब आप गुलाब जामुन खाने के बारे में सोचते हैं।

क्योंकि गुलाब जामुन खाना स्वास्थ्य के लिए लाभदायक हैं। आपके लिए यह कथन कितना महत्वपूर्ण है।

- अत्यंत महत्वपूर्ण
- बहुत महत्वपूर्ण
- मामूली रूप से महत्वपूर्ण
- थोड़ा महत्वपूर्ण
- कतई महत्वपूर्ण नहीं

जब आप गुलाब जामुन खाने के बारे में सोचते हैं।

क्योंकि गुलाब जामुन कम समय में बन जाता है। आपके लिए यह कथन कितना महत्वपूर्ण है।

- अत्यंत महत्वपूर्ण
- बहुत महत्वपूर्ण

- मामूली रूप से महत्वपूर्ण
- थोड़ा महत्वपूर्ण
- कतई महत्वपूर्ण नहीं

जब आप गुलाब जामुन खाने के बारे में सोचते हैं।

क्योंकि गुलाब जामुन का स्वाद अच्छा है। आपके लिए यह कथन कितना महत्वपूर्ण है।

- अत्यंत महत्वपूर्ण
- बहुत महत्वपूर्ण
- मामूली रूप से महत्वपूर्ण
- थोड़ा महत्वपूर्ण
- कतई महत्वपूर्ण नहीं

जब आप गुलाब जामुन खाने के बारे में सोचते हैं।

क्योंकि गुलाब जामुन मुझे तृप्त करता है। आपके लिए यह कथन कितना महत्वपूर्ण है।

- अत्यंत महत्वपूर्ण
- बहुत महत्वपूर्ण
- मामूली रूप से महत्वपूर्ण
- थोड़ा महत्वपूर्ण
- कतई महत्वपूर्ण नहीं

जब आप गुलाब जामुन खाने के बारे में सोचते हैं।

क्योंकि गुलाब जामुन प्राकृतिक है। आपके लिए यह कथन कितना महत्वपूर्ण है।

- अत्यंत महत्वपूर्ण
- बहुत महत्वपूर्ण
- मामूली रूप से महत्वपूर्ण

- थोड़ा महत्वपूर्ण
- कतई महत्वपूर्ण नहीं

जब आप गुलाब जामुन खाने के बारे में सोचते हैं।

क्योंकि आप अधिक पैसे खर्च नहीं करना चाहते है। आपके लिए यह कथन कितना महत्वपूर्ण है।

- अत्यंत महत्वपूर्ण
- बहुत महत्वपूर्ण
- मामूली रूप से महत्वपूर्ण
- थोड़ा महत्वपूर्ण
- कतई महत्वपूर्ण नहीं

जब आप गुलाब जामुन खाने के बारे में सोचते हैं।

क्योंकि गुलाब जामुन में कम कैलोरीज होती हैं। आपके लिए यह कथन कितना महत्वपूर्ण है।

- अत्यंत महत्वपूर्ण
- बहुत महत्वपूर्ण
- मामूली रूप से महत्वपूर्ण
- थोड़ा महत्वपूर्ण
- कतई महत्वपूर्ण नहीं

जब आप गुलाब जामुन खाने के बारे में सोचते हैं।

क्योंकि आप निराश रहते हैं। आपके लिए यह कथन कितना महत्वपूर्ण है।

- अत्यंत महत्वपूर्ण
- बहुत महत्वपूर्ण
- मामूली रूप से महत्वपूर्ण
- थोड़ा महत्वपूर्ण

- कतई महत्वपूर्ण नहीं

जब आप गुलाब जामुन खाने के बारे में सोचते हैं।

क्योंकि गुलाब जामुन खाना आपको दूसरों के सामने अच्छा दिखाता हैं। आपके लिए यह कथन कितना महत्वपूर्ण है।

- अत्यंत महत्वपूर्ण
- बहुत महत्वपूर्ण
- मामूली रूप से महत्वपूर्ण
- थोड़ा महत्वपूर्ण
- कतई महत्वपूर्ण नहीं

जब आप गुलाब जामुन खाने के बारे में सोचते हैं।

क्योंकि गुलाब जामुन नहीं खाना अशिष्ट होता है। आपके लिए यह कथन कितना महत्वपूर्ण है।

- अत्यंत महत्वपूर्ण
- बहुत महत्वपूर्ण
- मामूली रूप से महत्वपूर्ण
- थोड़ा महत्वपूर्ण
- कतई महत्वपूर्ण नहीं

जब आप गुलाब जामुन खाने के बारे में सोचते हैं।

क्योंकि गुलाब जामुन कि प्रस्तुति आकर्षक होती है। आपके लिए यह कथन कितना महत्वपूर्ण है।

- अत्यंत महत्वपूर्ण
- बहुत महत्वपूर्ण
- मामूली रूप से महत्वपूर्ण
- थोड़ा महत्वपूर्ण
- कतई महत्वपूर्ण नहीं

जब आप गुलाब जामुन खाने के बारे में सोचते हैं।

ताकि आप अन्य लोगों के साथ समय बिता सके। आपके लिए यह कथन कितना महत्वपूर्ण है।

- अत्यंत महत्वपूर्ण
- बहुत महत्वपूर्ण
- मामूली रूप से महत्वपूर्ण
- थोड़ा महत्वपूर्ण
- कतई महत्वपूर्ण नहीं

जब आप गुलाब जामुन खाने के बारे में सोचते हैं।

क्योंकि गुलाब जामुन जैविक होता है। आपके लिए यह कथन कितना महत्वपूर्ण है।

- अत्यंत महत्वपूर्ण
- बहुत महत्वपूर्ण
- मामूली रूप से महत्वपूर्ण
- थोड़ा महत्वपूर्ण
- कतई महत्वपूर्ण नहीं

जब आप गुलाब जामुन खाने के बारे में सोचते हैं।

क्योंकि आप बचपन से गुलाब जामुन खाते रहे हैं। आपके लिए यह कथन कितना महत्वपूर्ण है।

- अत्यंत महत्वपूर्ण
- बहुत महत्वपूर्ण
- मामूली रूप से महत्वपूर्ण
- थोड़ा महत्वपूर्ण
- कतई महत्वपूर्ण नहीं

जब आप गुलाब जामुन खाने के बारे में सोचते हैं।

क्योंकि आप गुलाब जामुन खाने में आनंद महसूस करते हैं। आपके लिए यह कथन कितना महत्वपूर्ण है।

- अत्यंत महत्वपूर्ण
- बहुत महत्वपूर्ण
- मामूली रूप से महत्वपूर्ण
- थोड़ा महत्वपूर्ण
- कतई महत्वपूर्ण नहीं

जब आप गुलाब जामुन खाने के बारे में सोचते हैं।

क्योंकि आप भूखे रहते हैं। आपके लिए यह कथन कितना महत्वपूर्ण है।

- अत्यंत महत्वपूर्ण
- बहुत महत्वपूर्ण
- मामूली रूप से महत्वपूर्ण
- थोड़ा महत्वपूर्ण
- कतई महत्वपूर्ण नहीं

जब आप गुलाब जामुन खाने के बारे में सोचते हैं।

क्योंकि आप आमतौर पर गुलाब जामुन खाते हैं। आपके लिए यह कथन कितना महत्वपूर्ण है।

- अत्यंत महत्वपूर्ण
- बहुत महत्वपूर्ण
- मामूली रूप से महत्वपूर्ण
- थोड़ा महत्वपूर्ण
- कतई महत्वपूर्ण नहीं

जब आप गुलाब जामुन खाने के बारे में सोचते हैं।

क्योंकि आपके भीतर गुलाब जामुन खाने कि भूख रहती है। आपके लिए यह कथन कितना महत्वपूर्ण है।

- अत्यंत महत्वपूर्ण
- बहुत महत्वपूर्ण
- मामूली रूप से महत्वपूर्ण
- थोड़ा महत्वपूर्ण
- कतई महत्वपूर्ण नहीं

जब आप गुलाब जामुन खाने के बारे में सोचते हैं।

क्योंकि गुलाब जामुन तैयार करना आसान है। आपके लिए यह कथन कितना महत्वपूर्ण है।

- अत्यंत महत्वपूर्ण
- बहुत महत्वपूर्ण
- मामूली रूप से महत्वपूर्ण
- थोड़ा महत्वपूर्ण
- कतई महत्वपूर्ण नहीं

जब आप गुलाब जामुन खाने के बारे में सोचते हैं।

क्योंकि आप उदास रहते है। आपके लिए यह कथन कितना महत्वपूर्ण है।

- अत्यंत महत्वपूर्ण
- बहुत महत्वपूर्ण
- मामूली रूप से महत्वपूर्ण
- थोड़ा महत्वपूर्ण
- कतई महत्वपूर्ण नहीं

जब आप गुलाब जामुन खाने के बारे में सोचते हैं।

[illegible]

क्योंकि गुलाब जामुन में वसा कि मात्रा कम होती है। आपके लिए यह कथन कितना महत्वपूर्ण है।

- अत्यंत महत्वपूर्ण
- बहुत महत्वपूर्ण
- मामूली रूप से महत्वपूर्ण
- थोड़ा महत्वपूर्ण
- कतई महत्वपूर्ण नहीं

जब आप गुलाब जामुन खाने के बारे में सोचते हैं।

क्योंकि आप गुलाब जामुन को टीवी या विज्ञापन पर देखते है। आपके लिए यह कथन कितना महत्वपूर्ण है।

- अत्यंत महत्वपूर्ण
- बहुत महत्वपूर्ण
- मामूली रूप से महत्वपूर्ण
- थोड़ा महत्वपूर्ण
- कतई महत्वपूर्ण नहीं

जब आप गुलाब जामुन खाने के बारे में सोचते हैं।

क्योंकि गुलाब जामुन सामाजिक समारोहों को अधिक आरामदायक बनाता है। आपके लिए यह कथन कितना महत्वपूर्ण है।

- अत्यंत महत्वपूर्ण
- बहुत महत्वपूर्ण
- मामूली रूप से महत्वपूर्ण
- थोड़ा महत्वपूर्ण
- कतई महत्वपूर्ण नहीं

जब आप गुलाब जामुन खाने के बारे में सोचते हैं।

क्योंकि आपको गुलाब जामुन खाना पड़ता है। आपके लिए यह कथन कितना महत्वपूर्ण है।

- अत्यंत महत्वपूर्ण
- बहुत महत्वपूर्ण
- मामूली रूप से महत्वपूर्ण
- थोड़ा महत्वपूर्ण
- कतई महत्वपूर्ण नहीं

जब आप गुलाब जामुन खाने के बारे में सोचते हैं।

क्योंकि गुलाब जामुन दूसरों को पसंद है। आपके लिए यह कथन कितना महत्वपूर्ण है।

- अत्यंत महत्वपूर्ण
- बहुत महत्वपूर्ण
- मामूली रूप से महत्वपूर्ण
- थोड़ा महत्वपूर्ण
- कतई महत्वपूर्ण नहीं

जब आप गुलाब जामुन खाने के बारे में सोचते हैं।

क्योंकि आप अकेला महसूस करते है। आपके लिए यह कथन कितना महत्वपूर्ण है।

- अत्यंत महत्वपूर्ण
- बहुत महत्वपूर्ण
- मामूली रूप से महत्वपूर्ण
- थोड़ा महत्वपूर्ण
- कतई महत्वपूर्ण नहीं

जब आप गुलाब जामुन खाने के बारे में सोचते हैं।

क्योंकि आप अपने वजन पर निगरानी रखते है। आपके लिए यह कथन कितना महत्वपूर्ण है।

- अत्यंत महत्वपूर्ण

- बहुत महत्वपूर्ण
- मामूली रूप से महत्वपूर्ण
- थोड़ा महत्वपूर्ण
- कतई महत्वपूर्ण नहीं

जब आप गुलाब जामुन खाने के बारे में सोचते हैं।

क्योंकि गुलाब जामुन में कोई हानिकारक (कीटनाशक, प्रदूषक, एंटीबायोटिक) पदार्थ नहीं होता है। आपके लिए यह कथन कितना महत्वपूर्ण है।

- अत्यंत महत्वपूर्ण
- बहुत महत्वपूर्ण
- मामूली रूप से महत्वपूर्ण
- थोड़ा महत्वपूर्ण
- कतई महत्वपूर्ण नहीं

जब आप गुलाब जामुन खाने के बारे में सोचते हैं।

क्योंकि गुलाब जामुन बनाना आसान है। आपके लिए यह कथन कितना महत्वपूर्ण है।

- अत्यंत महत्वपूर्ण
- बहुत महत्वपूर्ण
- मामूली रूप से महत्वपूर्ण
- थोड़ा महत्वपूर्ण
- कतई महत्वपूर्ण नहीं

जब आप गुलाब जामुन खाने के बारे में सोचते हैं।

क्योंकि मूझे ऊर्जा की जरूरत होती है। आपके लिए यह कथन कितना महत्वपूर्ण है।

- अत्यंत महत्वपूर्ण
- बहुत महत्वपूर्ण

- मामूली रूप से महत्वपूर्ण
- थोड़ा महत्वपूर्ण
- कतई महत्वपूर्ण नहीं

जब आप गुलाब जामुन खाने के बारे में सोचते हैं।

क्योंकि आप गुलाब जामुन से परिचित है। आपके लिए यह कथन कितना महत्वपूर्ण है।

- अत्यंत महत्वपूर्ण
- बहुत महत्वपूर्ण
- मामूली रूप से महत्वपूर्ण
- थोड़ा महत्वपूर्ण
- कतई महत्वपूर्ण नहीं

जब आप गुलाब जामुन खाने के बारे में सोचते हैं।

क्योंकि आपको गुलाब जामुन पसंद है। आपके लिए यह कथन कितना महत्वपूर्ण है।

- अत्यंत महत्वपूर्ण
- बहुत महत्वपूर्ण
- मामूली रूप से महत्वपूर्ण
- थोड़ा महत्वपूर्ण
- कतई महत्वपूर्ण नहीं

जब आप गुलाब जामुन खाने के बारे में सोचते हैं।

क्योंकि गुलाब जामुन खाना सुखदेय है। आपके लिए यह कथन कितना महत्वपूर्ण है।

- अत्यंत महत्वपूर्ण
- बहुत महत्वपूर्ण
- मामूली रूप से महत्वपूर्ण

- थोड़ा महत्वपूर्ण
- कतई महत्वपूर्ण नहीं

जब आप गुलाब जामुन खाने के बारे में सोचते हैं।

आप गुलाब जामुन अपने आपको पुरस्कृत करने के लिए गुलाब जामुन खाते हैं। आपके लिए यह कथन कितना महत्वपूर्ण है।

- अत्यंत महत्वपूर्ण
- बहुत महत्वपूर्ण
- मामूली रूप से महत्वपूर्ण
- थोड़ा महत्वपूर्ण
- कतई महत्वपूर्ण नहीं

जब आप गुलाब जामुन खाने के बारे में सोचते हैं।

क्योंकि गुलाब जामुन खाना आपकी परंपरा का हिस्सा है। आपके लिए यह कथन कितना महत्वपूर्ण है।

- अत्यंत महत्वपूर्ण
- बहुत महत्वपूर्ण
- मामूली रूप से महत्वपूर्ण
- थोड़ा महत्वपूर्ण
- कतई महत्वपूर्ण नहीं

जब आप गुलाब जामुन खाने के बारे में सोचते हैं।

क्योंकि यह कुछ स्थितियों से संबंधित है। आपके लिए यह कथन कितना महत्वपूर्ण है।

- अत्यंत महत्वपूर्ण
- बहुत महत्वपूर्ण
- मामूली रूप से महत्वपूर्ण
- थोड़ा महत्वपूर्ण

- कतई महत्वपूर्ण नहीं

जब आप गुलाब जामुन खाने के बारे में सोचते हैं।

आप गुलाब जामुन संतुलित आहार पाने के लिए खाते हैं। आपके लिए यह कथन कितना महत्वपूर्ण है।

- अत्यंत महत्वपूर्ण
- बहुत महत्वपूर्ण
- मामूली रूप से महत्वपूर्ण
- थोड़ा महत्वपूर्ण
- कतई महत्वपूर्ण नहीं

जब आप गुलाब जामुन खाने के बारे में सोचते हैं।

क्योंकि गुलाब जामुन खाना सामाजिक है। आपके लिए यह कथन कितना महत्वपूर्ण है।

- अत्यंत महत्वपूर्ण
- बहुत महत्वपूर्ण
- मामूली रूप से महत्वपूर्ण
- थोड़ा महत्वपूर्ण
- कतई महत्वपूर्ण नहीं

जब आप गुलाब जामुन खाने के बारे में सोचते हैं।

क्योंकि गुलाब जामुन सस्ता है। आपके लिए यह कथन कितना महत्वपूर्ण है।

- अत्यंत महत्वपूर्ण
- बहुत महत्वपूर्ण
- मामूली रूप से महत्वपूर्ण
- थोड़ा महत्वपूर्ण
- कतई महत्वपूर्ण नहीं

जब आप गुलाब जामुन खाने के बारे में सोचते हैं।

क्योंकि गुलाब जामुन आपके लिए आकर्षक भोजन है। आपके लिए यह कथन कितना महत्वपूर्ण है।

- अत्यंत महत्वपूर्ण
- बहुत महत्वपूर्ण
- मामूली रूप से महत्वपूर्ण
- थोड़ा महत्वपूर्ण
- कतई महत्वपूर्ण नहीं

जब आप गुलाब जामुन खाने के बारे में सोचते हैं।

दुसरो को निराश करने से बचने के लिए आप गुलाब जामुन खाते हैं। आपके लिए यह कथन कितना महत्वपूर्ण है।

- अत्यंत महत्वपूर्ण
- बहुत महत्वपूर्ण
- मामूली रूप से महत्वपूर्ण
- थोड़ा महत्वपूर्ण
- कतई महत्वपूर्ण नहीं

जब आप गुलाब जामुन खाने के बारे में सोचते हैं।

क्योंकि गुलाब जामुन खाना फैशनेबल है। आपके लिए यह कथन कितना महत्वपूर्ण है।

- अत्यंत महत्वपूर्ण
- बहुत महत्वपूर्ण
- मामूली रूप से महत्वपूर्ण
- थोड़ा महत्वपूर्ण
- कतई महत्वपूर्ण नहीं

जब आप गुलाब जामुन खाने के बारे में सोचते हैं।

क्योंकि गुलाब जामुन सेल पर उपलब्ध हैं। आपके लिए यह कथन कितना महत्वपूर्ण है।

- अत्यंत महत्वपूर्ण
- बहुत महत्वपूर्ण
- मामूली रूप से महत्वपूर्ण
- थोड़ा महत्वपूर्ण
- कतई महत्वपूर्ण नहीं

जब आप गुलाब जामुन खाने के बारे में सोचते हैं।

क्योंकि यह आपके शरीर को आकार में रखता है। आपके लिए यह कथन कितना महत्वपूर्ण है।

- अत्यंत महत्वपूर्ण
- बहुत महत्वपूर्ण
- मामूली रूप से महत्वपूर्ण
- थोड़ा महत्वपूर्ण
- कतई महत्वपूर्ण नहीं

जब आप गुलाब जामुन खाने के बारे में सोचते हैं।

क्योंकि गुलाब जामुन ही एकमात्र विकल्प था। आपके लिए यह कथन कितना महत्वपूर्ण है।

- अत्यंत महत्वपूर्ण
- बहुत महत्वपूर्ण
- मामूली रूप से महत्वपूर्ण
- थोड़ा महत्वपूर्ण
- कतई महत्वपूर्ण नहीं

जब आप गुलाब जामुन खाने के बारे में सोचते हैं।

क्योंकि आप गुलाब जामुन हर दिन खाना चाहते है। आपके लिए यह कथन कितना महत्वपूर्ण है।

- अत्यंत महत्वपूर्ण
- बहुत महत्वपूर्ण
- मामूली रूप से महत्वपूर्ण
- थोड़ा महत्वपूर्ण
- कतई महत्वपूर्ण नहीं

जब आप गुलाब जामुन नहीं खाने के बारे में सोचते हैं।

क्योंकि आपको नहीं लगता कि गुलाब जामुन एक नाश्ता है। आपके लिए यह कथन कितना महत्वपूर्ण है।

- अत्यंत महत्वपूर्ण
- बहुत महत्वपूर्ण
- मामूली रूप से महत्वपूर्ण
- थोड़ा महत्वपूर्ण
- कतई महत्वपूर्ण नहीं

जब आप गुलाब जामुन नहीं खाने के बारे में सोचते हैं।

क्योंकि आपको गुलाब जामुन पसंद नहीं है। आपके लिए यह कथन कितना महत्वपूर्ण है।

- अत्यंत महत्वपूर्ण
- बहुत महत्वपूर्ण
- मामूली रूप से महत्वपूर्ण
- थोड़ा महत्वपूर्ण
- कतई महत्वपूर्ण नहीं

जब आप गुलाब जामुन नहीं खाने के बारे में सोचते हैं।

क्योंकि आप आमतौर पर गुलाब जामुन नहीं खाते है। आपके लिए यह कथन कितना महत्वपूर्ण है।

- अत्यंत महत्वपूर्ण
- बहुत महत्वपूर्ण
- मामूली रूप से महत्वपूर्ण
- थोड़ा महत्वपूर्ण
- कतई महत्वपूर्ण नहीं

जब आप गुलाब जामुन नहीं खाने के बारे में सोचते हैं।

क्योंकि गुलाब जामुन कि खुराक का आकार मेरे लिए उपयुक्त नहीं होता है। आपके लिए यह कथन कितना महत्वपूर्ण है।

- अत्यंत महत्वपूर्ण
- बहुत महत्वपूर्ण
- मामूली रूप से महत्वपूर्ण
- थोड़ा महत्वपूर्ण
- कतई महत्वपूर्ण नहीं

जब आप गुलाब जामुन नहीं खाने के बारे में सोचते हैं।

क्योंकि गुलाब जामुन आपकी भूख मिटाने के लिए पर्याप्त नहीं होता है। आपके लिए यह कथन कितना महत्वपूर्ण है।

- अत्यंत महत्वपूर्ण
- बहुत महत्वपूर्ण
- मामूली रूप से महत्वपूर्ण
- थोड़ा महत्वपूर्ण
- कतई महत्वपूर्ण नहीं

जब आप गुलाब जामुन नहीं खाने के बारे में सोचते हैं।

क्योंकि गुलाब जामुन स्वास्थ्यय के लिए लाभदायक नहीं है। आपके लिए यह कथन कितना महत्वपूर्ण है।

- अत्यंत महत्वपूर्ण
- बहुत महत्वपूर्ण
- मामूली रूप से महत्वपूर्ण
- थोड़ा महत्वपूर्ण
- कतई महत्वपूर्ण नहीं

जब आप गुलाब जामुन नहीं खाने के बारे में सोचते हैं।

क्योंकि गुलाब जामुन बनाना मुश्किल होता है। आपके लिए यह कथन कितना महत्वपूर्ण है।

- अत्यंत महत्वपूर्ण
- बहुत महत्वपूर्ण
- मामूली रूप से महत्वपूर्ण
- थोड़ा महत्वपूर्ण
- कतई महत्वपूर्ण नहीं

जब आप गुलाब जामुन नहीं खाने के बारे में सोचते हैं।

क्योंकि आप गुलाब जामुन नहीं खाना चाहते है। आपके लिए यह कथन कितना महत्वपूर्ण है।

- अत्यंत महत्वपूर्ण
- बहुत महत्वपूर्ण
- मामूली रूप से महत्वपूर्ण
- थोड़ा महत्वपूर्ण
- कतई महत्वपूर्ण नहीं

जब आप गुलाब जामुन नहीं खाने के बारे में सोचते हैं।

क्योंकि गुलाब जामुन स्थिति के लिए उपयुक्त नहीं होता है। आपके लिए यह कथन कितना महत्वपूर्ण है।

- अत्यंत महत्वपूर्ण
- बहुत महत्वपूर्ण
- मामूली रूप से महत्वपूर्ण
- थोड़ा महत्वपूर्ण
- कतई महत्वपूर्ण नहीं

जब आप गुलाब जामुन नहीं खाने के बारे में सोचते हैं।

क्योंकि गुलाब जामुन अन्य लोगों के आसपास खाने लायक भोजन नहीं है। आपके लिए यह कथन कितना महत्वपूर्ण है।

- अत्यंत महत्वपूर्ण
- बहुत महत्वपूर्ण
- मामूली रूप से महत्वपूर्ण
- थोड़ा महत्वपूर्ण
- कतई महत्वपूर्ण नहीं

जब आप गुलाब जामुन नहीं खाने के बारे में सोचते हैं।

क्योंकि गुलाब जामुन कि कीमत बहुत अधिक है। आपके लिए यह कथन कितना महत्वपूर्ण है।

- अत्यंत महत्वपूर्ण
- बहुत महत्वपूर्ण
- मामूली रूप से महत्वपूर्ण
- थोड़ा महत्वपूर्ण
- कतई महत्वपूर्ण नहीं

जब आप गुलाब जामुन नहीं खाने के बारे में सोचते हैं।

क्योंकि गुलाब जामुन आपको दिखने में अच्छा नहीं लगता है। आपके लिए यह कथन कितना महत्वपूर्ण है।

- अत्यंत महत्वपूर्ण
- बहुत महत्वपूर्ण
- मामूली रूप से महत्वपूर्ण
- थोड़ा महत्वपूर्ण
- कतई महत्वपूर्ण नहीं

जब आप गुलाब जामुन नहीं खाने के बारे में सोचते हैं।

क्योंकि गुलाब जामुन मे कैलोरी के मात्रा बहुत अधिक होती है। आपके लिए यह कथन कितना महत्वपूर्ण है।

- अत्यंत महत्वपूर्ण
- बहुत महत्वपूर्ण
- मामूली रूप से महत्वपूर्ण
- थोड़ा महत्वपूर्ण
- कतई महत्वपूर्ण नहीं

जब आप गुलाब जामुन नहीं खाने के बारे में सोचते हैं।

क्योंकि आप गुलाब जामुन नहीं खाना चाहते है। आपके लिए यह कथन कितना महत्वपूर्ण है।

- अत्यंत महत्वपूर्ण
- बहुत महत्वपूर्ण
- मामूली रूप से महत्वपूर्ण
- थोड़ा महत्वपूर्ण
- कतई महत्वपूर्ण नहीं

जब आप गुलाब जामुन नहीं खाने के बारे में सोचते हैं।

क्योंकि आप गुलाब जामुन खाने से दूसरों के सामने पिछड़ा महसूस करते है। आपके लिए यह कथन कितना महत्वपूर्ण है।

- अत्यंत महत्वपूर्ण
- बहुत महत्वपूर्ण
- मामूली रूप से महत्वपूर्ण
- थोड़ा महत्वपूर्ण
- कतई महत्वपूर्ण नहीं

जब आप गुलाब जामुन नहीं खाने के बारे में सोचते हैं।

क्योंकि आपकी गुलाब जामुन खाने कि इच्छा नहीं होती है। आपके लिए यह कथन कितना महत्वपूर्ण है।

- अत्यंत महत्वपूर्ण
- बहुत महत्वपूर्ण
- मामूली रूप से महत्वपूर्ण
- थोड़ा महत्वपूर्ण
- कतई महत्वपूर्ण नहीं

जब आप गुलाब जामुन नहीं खाने के बारे में सोचते हैं।

क्योंकि अपने हाल ही में गुलाब जामुन खाया था और अब आप बार-बार गुलाब जामुन नहीं खाना चाहते हैं।
आपके लिए यह कथन कितना महत्वपूर्ण है।

- अत्यंत महत्वपूर्ण
- बहुत महत्वपूर्ण
- मामूली रूप से महत्वपूर्ण
- थोड़ा महत्वपूर्ण
- कतई महत्वपूर्ण नहीं

जब आप गुलाब जामुन नहीं खाने के बारे में सोचते हैं।

क्योंकि आपको हर दिन एक ही प्रकार का भोजन खाना पसंद है। आपके लिए यह कथन कितना महत्वपूर्ण है।

- अत्यंत महत्वपूर्ण
- बहुत महत्वपूर्ण
- मामूली रूप से महत्वपूर्ण
- थोड़ा महत्वपूर्ण
- कतई महत्वपूर्ण नहीं

जब आप गुलाब जामुन नहीं खाने के बारे में सोचते हैं।

क्योंकि गुलाब जामुन खाना आपको दुखी, अकेला और कुंठित महसूस कराता है। आपके लिए यह कथन कितना महत्वपूर्ण है।

- अत्यंत महत्वपूर्ण
- बहुत महत्वपूर्ण
- मामूली रूप से महत्वपूर्ण
- थोड़ा महत्वपूर्ण
- कतई महत्वपूर्ण नहीं

जब आप गुलाब जामुन नहीं खाने के बारे में सोचते हैं।

क्योंकि गुलाब जामुन जैविक (आर्गेनिक) नहीं है। आपके लिए यह कथन कितना महत्वपूर्ण है।

- अत्यंत महत्वपूर्ण
- बहुत महत्वपूर्ण
- मामूली रूप से महत्वपूर्ण
- थोड़ा महत्वपूर्ण
- कतई महत्वपूर्ण नहीं

Powered by Qualtrics
